# Macrocyclisation of small peptides enabled by oxetane incorporation†
†Electronic supplementary information (ESI) available: Experimental procedures for the synthesis of peptide precursors, macrocyclisations, kinetic studies, molecular dynamics simulations and bioassays; characterisation data for all new compounds and copies of ^1^H and ^13^C NMR spectra. See DOI: 10.1039/c8sc05474f


**DOI:** 10.1039/c8sc05474f

**Published:** 2019-01-03

**Authors:** Stefan Roesner, George J. Saunders, Ina Wilkening, Eleanor Jayawant, Joanna V. Geden, Paul Kerby, Ann M. Dixon, Rebecca Notman, Michael Shipman

**Affiliations:** a Department of Chemistry , University of Warwick , Gibbet Hill Road , Coventry , CV4 7AL , UK . Email: m.shipman@warwick.ac.uk

## Abstract

Cyclic peptides are an important source of new drugs but are challenging to produce synthetically. We show that head-to-tail peptide macrocyclisations are greatly improved, as measured by isolated yields, reaction rates and product distribution, by substitution of one of the backbone amide C=O bonds with an oxetane ring. The cyclisation precursors are easily made by standard solution- or solid-phase peptide synthesis techniques. Macrocyclisations across a range of challenging ring sizes (tetra-, penta- and hexapeptides) are enabled by incorporation of this turn-inducing element. Oxetane incorporation is shown to be superior to other established amino acid modifications such as *N*-methylation. The positional dependence of the modification on cyclisation efficiency is mapped using a cyclic peptide of sequence LAGAY. We provide the first direct experimental evidence that oxetane modification induces a turn in linear peptide backbones, through the observation of *d*_NN_ (*i*, *i* + 2) and *d*_αN_ (*i*, *i* + 2) NOEs, which offers an explanation for these improvements. For cyclic peptide, cLAGAY, a combination of NMR derived distance restraints and molecular dynamics simulations are used to show that this modification alters the backbone conformation in proximity to the oxetane, with the flexibility of the ring reduced and a new intramolecular H-bond established. Finally, we incorporated an oxetane into a cyclic pentapeptide inhibitor of Aminopeptidase N, a transmembrane metalloprotease overexpressed on the surface of cancer cells. The inhibitor, cCNGRC, displayed similar IC_50_ values in the presence or absence of an oxetane at the glycine residue, indicating that bioactivity is fully retained upon amide C=O bond replacement.

## Abstract 2

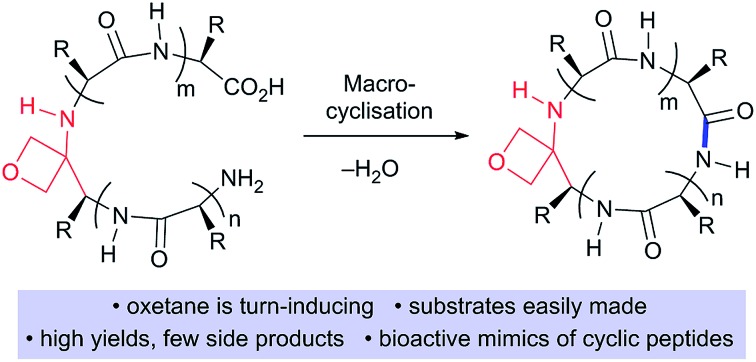
Head-to-tail peptide macrocyclisations are significantly improved, as measured by isolated yields, reaction rates and product distribution, by substitution of one of the backbone amide C=O bonds with an oxetane ring.

## Introduction

**Scheme 1:**
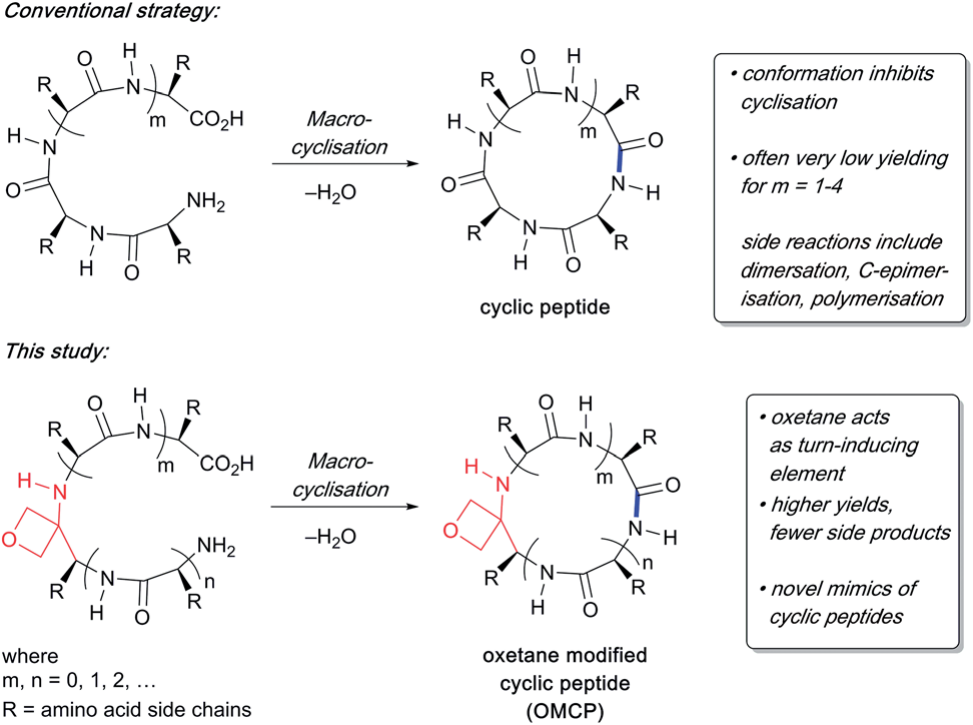
Difficulties associated with making small cyclic peptides and improvements arising from oxetane introduction.

Currently more than 40 cyclic peptide drugs are in clinical use with the vast majority derived from natural products (*e.g.* cyclosporine, vancomycin).^1^ Compared to their linear counterparts, cyclic peptides benefit from enhanced cell permeability, increased target affinity, and resistance to proteolytic degradation.^2^ Moreover, they are capable of acting as inhibitors against some of the most challenging targets, including protein–protein interactions (PPIs).^3^ One major obstacle to the discovery and development of new cyclic peptide drugs relates to the difficulties associated with their synthesis.^4^ The head-to-tail cyclisation of short peptides containing seven or fewer amino acids is especially challenging. Common problems encountered during cyclisation are C-terminal epimerisation, cyclooligomerisation and the appearance of side products arising from polymerisation (Scheme 1). Consequently, there is a pressing need to discover new macrocyclisation strategies that can provide easy access to a variety of small cyclic peptide scaffolds suitable for use in drug discovery programmes.^5^

The incorporation of turn-inducing elements such as a pseudoproline, an *N*-alkylated or a d-amino acid into a linear peptide often enhances peptide macrocyclisations.4c,^6^ Recently we have shown that substitution of a backbone amide C=O with an oxetane ring induced turns in tripeptides,^7^ suggesting that oxetane introduction into the backbone of a linear peptide might offer a general method to improve macrocyclisation efficiency by promoting conformational preorganisation of the linear peptide such that the C- and N-termini are brought into close proximity (Scheme 1).

Importantly, the use of oxetanes as bioisosteres in small-molecule drug discovery is well established,^8^ with emerging applications in peptide science.^9^,^10^ For example, the grafting of oxetanes onto the cysteine side chains of proteins and antibodies can significantly improve their stability and activity.^9^ Additionally, the serum half-life and *in vivo* analgesic properties of *linear* peptides such as Leu-enkephalin can be improved by introduction of an oxetane.^10^

In this Edge Article, we show that many peptide macrocyclisations can be greatly improved by oxetane incorporation, and that the resulting modified peptides are biologically active, supporting the hypothesis that oxetane modified cyclic peptides (OMCPs) represent a powerful new class of peptidomimetic for drug discovery.

## Results and discussion

### Synthesis of oxetane modified cyclisation substrates

To study the impact of oxetane modification on peptide macrocyclisation efficiency, a range of linear peptide precursors were synthesised. Many of the substrates were made in solution using Bn ester/Z-protection of the C- and N-termini to allow the synthesis of salt-free precursors by use of a final hydrogenolysis step. This enabled accurate comparisons in product yields to be made across different substrates without having to correct for the impact of salts on the cyclisations. The synthesis of oxetane modified peptides was also achieved using the more practical approach of solid-phase peptide synthesis (SPPS). In most cases, control peptides without the oxetane modification were made for comparison purposes (see ESI†).

**Scheme 2:**
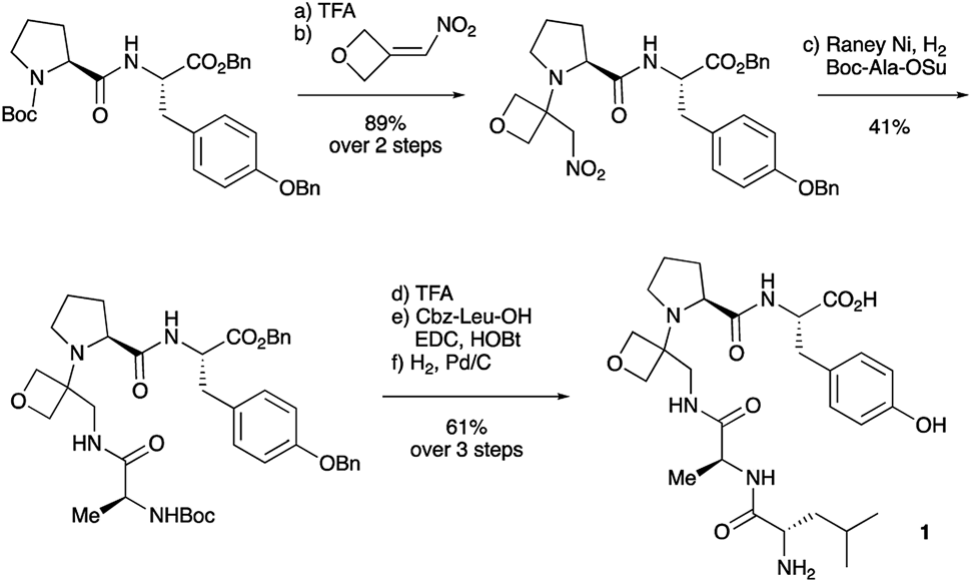
Synthesis of oxetane modified pentapeptide **1**. Reagents and conditions: (a) TFA/CH_2_Cl_2_ (1 : 1), 1 h; (b) (i) oxetan-3-one (2.0 equiv.), MeNO_2_ (2.8 equiv.), Et_3_N (0.4 equiv.), 0 °C to RT, 1 h; (ii) MeSO_2_Cl (2.0 equiv.), Et_3_N (4.0 equiv.), CH_2_Cl_2_, –78 °C, 1.5 h; (iii) amine from (a), Et_3_N (1.5 equiv.), –78 °C to RT, 16 h; (c) RANEY® Ni, H_2_, Boc-Ala-OSu (1.5 equiv.), THF, 4 h; (d) TFA/CH_2_Cl_2_ (1 : 1), 1 h; (e) Cbz-Leu-OH (1.1 equiv.), EDC·HCl (1.1 equiv.), HOBt·H_2_O (1.1 equiv.), NMM (4.0 equiv.), CH_2_Cl_2_, 24 h; (f) 10% Pd/C, H_2_, MeOH, 16 h.

**Scheme 3:**
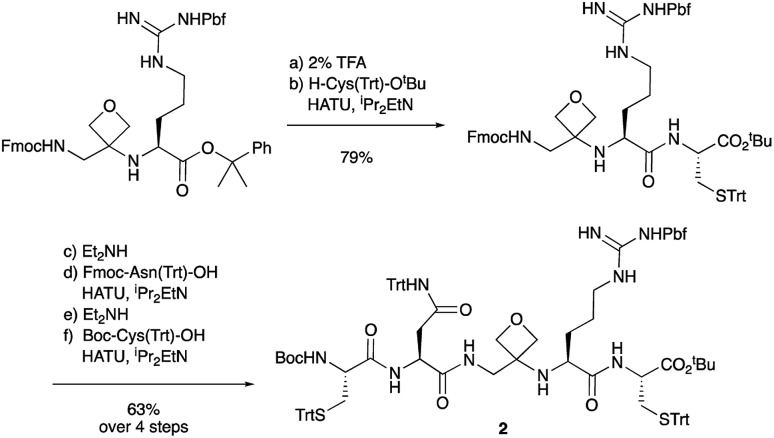
Synthesis of pentapeptide **2** using oxetane containing dipeptide building block. Reagents and conditions: (a) 2% TFA/CH_2_Cl_2_, 2 h; (b) H-Cys(Trt)-O^*t*^Bu (1.5 equiv.), HATU (1.1 equiv.), ^i^Pr_2_EtN (4.0 equiv.), DMF, 48 h; (c) Et_2_NH/CH_2_Cl_2_ (1 : 1), 1 h; (d) Fmoc-Asn(Trt)-OH (1.5 equiv), HATU (1.5 equiv.), ^i^Pr_2_EtN (3.0 equiv.), CH_2_Cl_2_, 16 h; (e) Et_2_NH/CH_2_Cl_2_ (1 : 1), 1 h; (f) Boc-Cys(Trt)-OH (1.4 equiv.), HATU (1.4 equiv.), ^i^Pr_2_EtN (3.0 equiv.), CH_2_Cl_2_, 16 h.

**Scheme 4:**
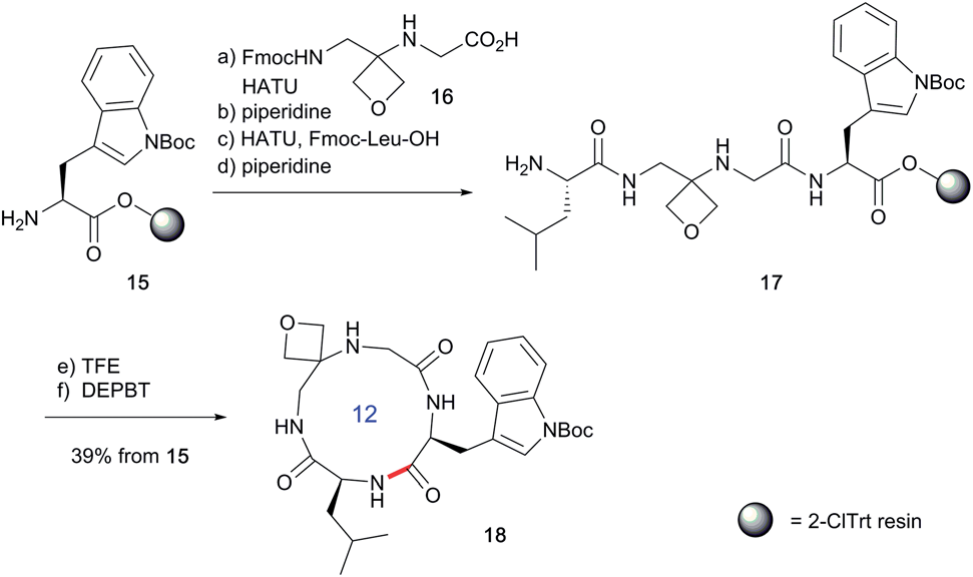
Synthesis of oxetane modified cyclic peptide **18** by SPPS. Reaction conditions: (a) **16** (2 equiv.), HATU (1.9 equiv.), ^i^Pr_2_EtN (4.0 equiv.), DMF, 2–4 h, double coupling; (b) 20% piperidine, DMF, 20 min; (c) Fmoc-Leu-OH (5.0 equiv.), HATU (4.9 equiv.), ^i^Pr_2_EtN (10 equiv.), DMF, 1 h; (d) 20% piperidine, DMF, 20 min; (e) TFE/CH_2_Cl_2_ (1 : 4), 3 × 1 h; (f) DEPBT (2.0 equiv.), ^i^Pr_2_EtN (2.0 equiv.), DMF (0.001 M), 64 h.

Two distinct methods were used to make the linear peptides. In the first, oxetane introduction was achieved by: (i) conjugate addition of the N-terminus of the growing peptide to 3-(nitromethylene)oxetane; (ii) nitro group reduction and *in situ* coupling of the resulting amine to the next protected amino acid, preactivated as its succinyl ester.^7^,^11^ This method proved very convenient for the solution-phase synthesis of oxetane modified glycine residues as illustrated by the preparation of pentapeptide **1** (Scheme 2). Alternatively, preformed Fmoc-protected dipeptide building blocks containing the oxetane modification were incorporated into the growing peptide chain as shown for the synthesis of pentapeptide **2** (Scheme 3). This second method is suitable for the introduction of oxetane modified derivatives of other l-amino acids,^10^ is fully compatible with standard Fmoc/^*t*^Bu SPPS,11*a* and avoids the need to subject the growing peptide chain to strongly reductive conditions. The assembly of resin-bound tetrapeptide **17** by SPPS provides an illustration of the utility of this method (Scheme 4), with additional examples in the ESI.†

### Macrocyclisation of oxetane modified pentapeptides

To study the impact of oxetane introduction on the efficiency of macrocyclisation, ring closure of the pentapeptide LAGPY in the presence and absence of oxetane modified glycine was studied under a variety of conditions. As shown in Table 1 (entries 6–8), this substrate was chosen because cyclisation of the corresponding unmodified pentapeptide is very low yielding (13–23%) even under high dilution.^12^ Upon introduction of the oxetane modification, a 3-fold yield improvement was observed. The best yield was obtained using PyBOP (Table 1, entry 3), although DEPBT and HATU were also efficient with similar levels of improvement seen. Later studies exploring the wider scope of this methodology led us to favour DEPBT as the reagent of choice in most cases. Of practical importance, the reaction can be conducted on a larger scale (0.5 mmol) under less dilute conditions (0.005 M) without loss of yield (Table 1, entry 1).

**Table 1:** Impact of oxetane modification on the cyclisation efficiency of a pentapeptide

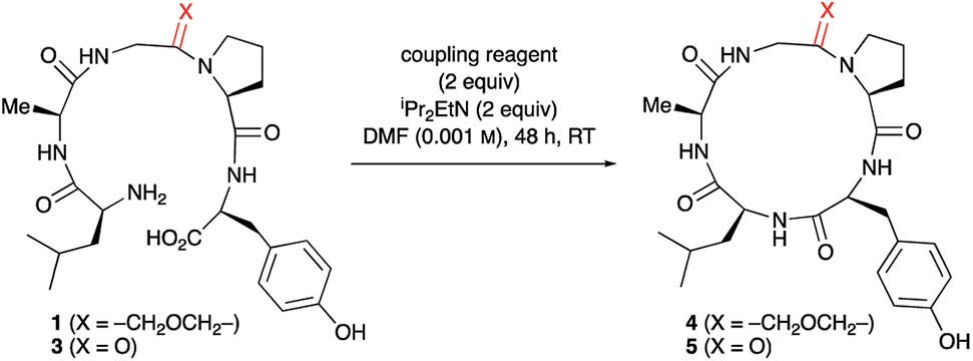				
Entry	Substrate^*a*^	Coupling reagent^*b*^	Product	Yield^*c*^ (%)
1	**1**	DEPBT	**4**	48 (50)^*d*^
2	**1**	DEPBT^*e*^	**4**	33
3	**1**	PyBOP	**4**	60
4	**1**	HATU	**4**	53
5	**1**	T3P	**4**	28
6	**3**	DEPBT	**5**	13^*f*^
7	**3**	PyBOP	**5**	23
8	**3**	HATU	**5**	15

^*a*^All reactions run on 0.1 mmol scale.

^*b*^3-(Diethoxyphosphoryloxy)-1,2,3-benzotriazin-4(3*H*)-one (DEPBT); *O*-(7-azabenzotriazol-1-yl)-*N*,*N*,*N*′,*N*′-tetramethyluronium hexafluorophosphate (HATU); (benzotriazol-1-yloxy)tripyrrolidinophosphonium hexafluorophosphate (PyBOP); propylphosphonic anhydride (T3P).

^*c*^Isolated yield after column chromatography.

^*d*^Yield in parenthesis relates to larger scale reaction (0.5 mmol) run at higher concentration (0.005 M).

^*e*^ ^i^Pr_2_EtN omitted.

^*f*^Taken from ref. 12.

**Fig. 1:**
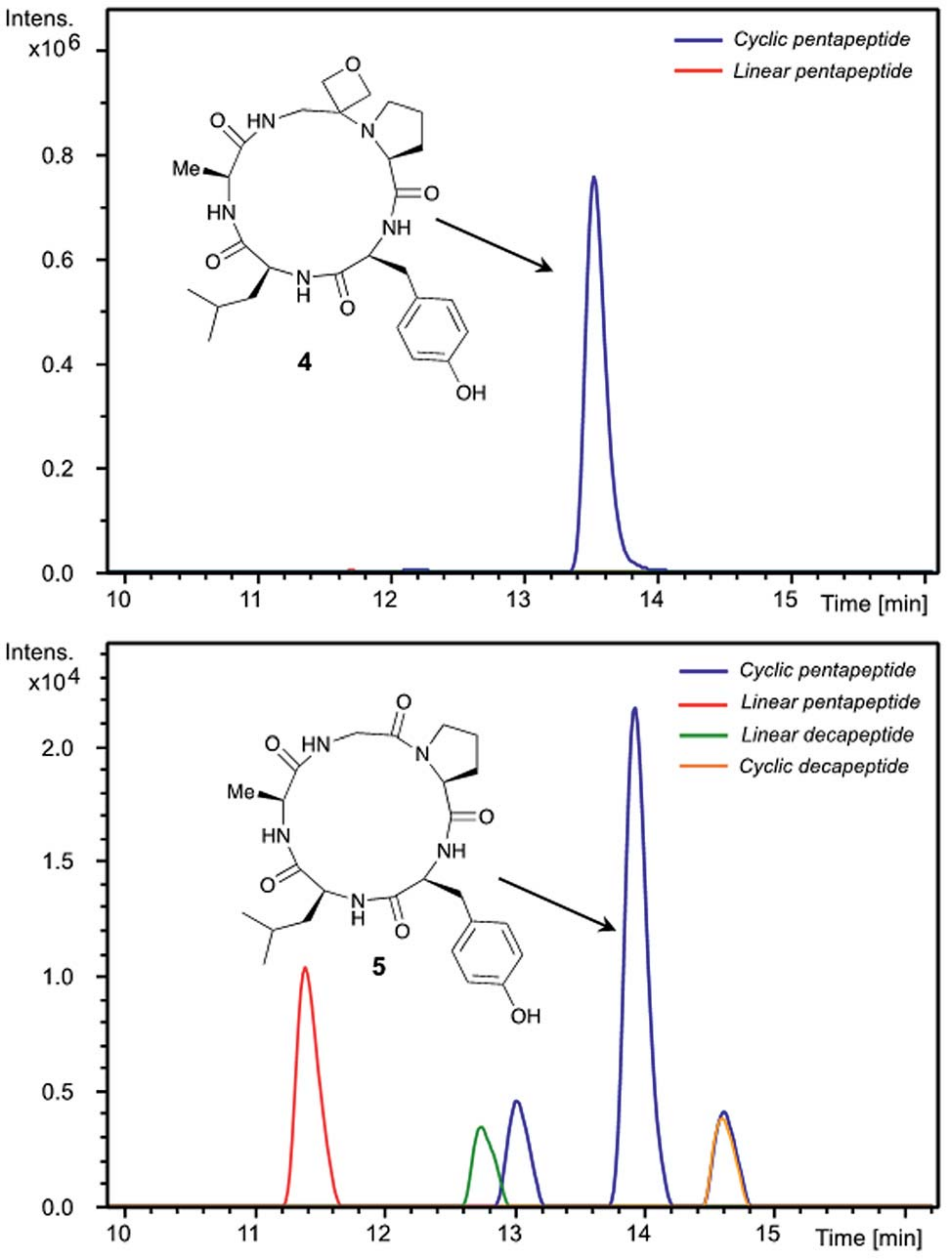
Extracted ion chromatograms of peptide macrocyclisations [DEPBT (2 equiv.), ^i^Pr_2_EtN (2 equiv.), DMF (0.001 M), 48 h, RT] for **1** → **4** (top) and **3** → **5** (bottom).

Insights into the impact of oxetane introduction on the efficiency of this macrocyclisation were revealed by LC-MS analysis (Fig. 1). In the cyclisation of the unmodified peptide, there are significant quantities of unreacted linear pentapeptide **3** left even after 48 h. Additionally, linear and cyclic decapeptides arising from substrate dimerisation were evident alongside a second cyclic pentapeptide arising from C-terminal epimerisation (Fig. 1, bottom). In contrast, for the oxetane containing precursor, essentially complete consumption of starting material is seen on this timescale. The reaction is also much cleaner with the corresponding by-products seen in only trace quantities (Fig. 1, top). Thus, significant improvements in this difficult macrocyclisation are realised through introduction of the oxetane motif.

**Fig. 2:**
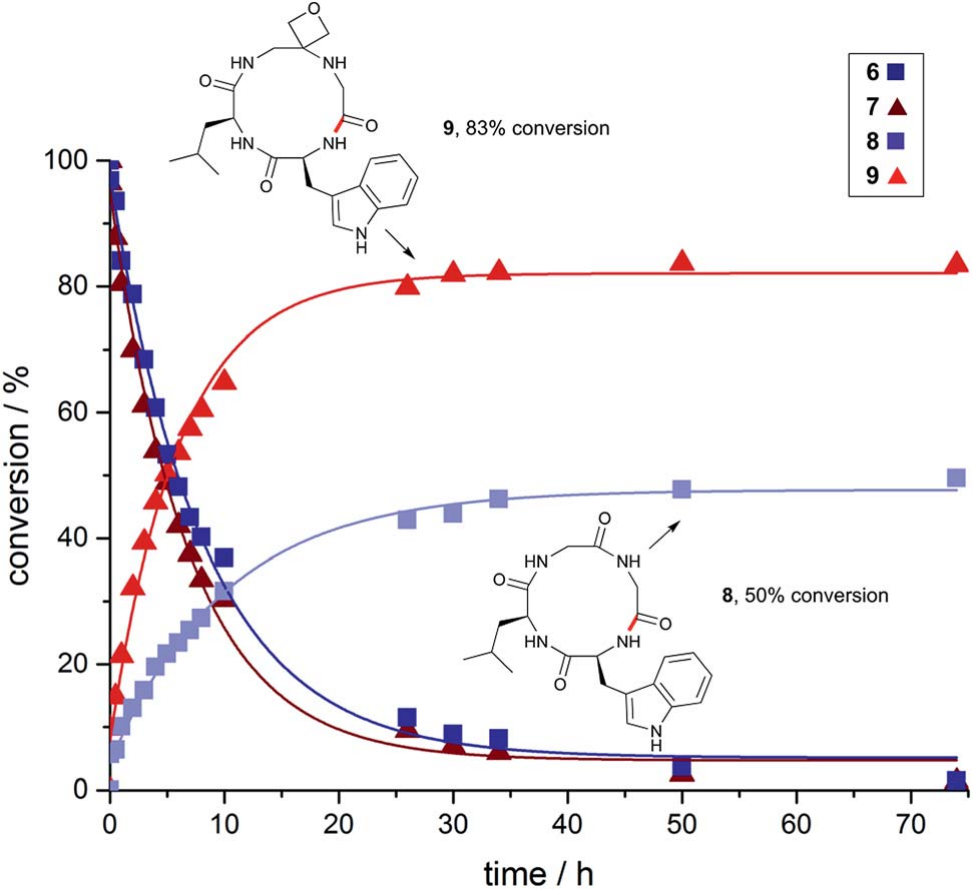
Impact of oxetane modification on macrocyclisation rates: Cyclisation of WLGG (**6**) and oxetane modified WLGOxG (**7**) monitored over 74 h using DEPBT (2 equiv.), ^i^Pr_2_EtN (2 equiv.) in DMF (0.001 M), RT. Bond formed in macrocyclisation indicated in red; peptides were quantified by analytical HPLC by integration of the corresponding UV signal at 280 nm (see ESI†).

**Fig. 3:**
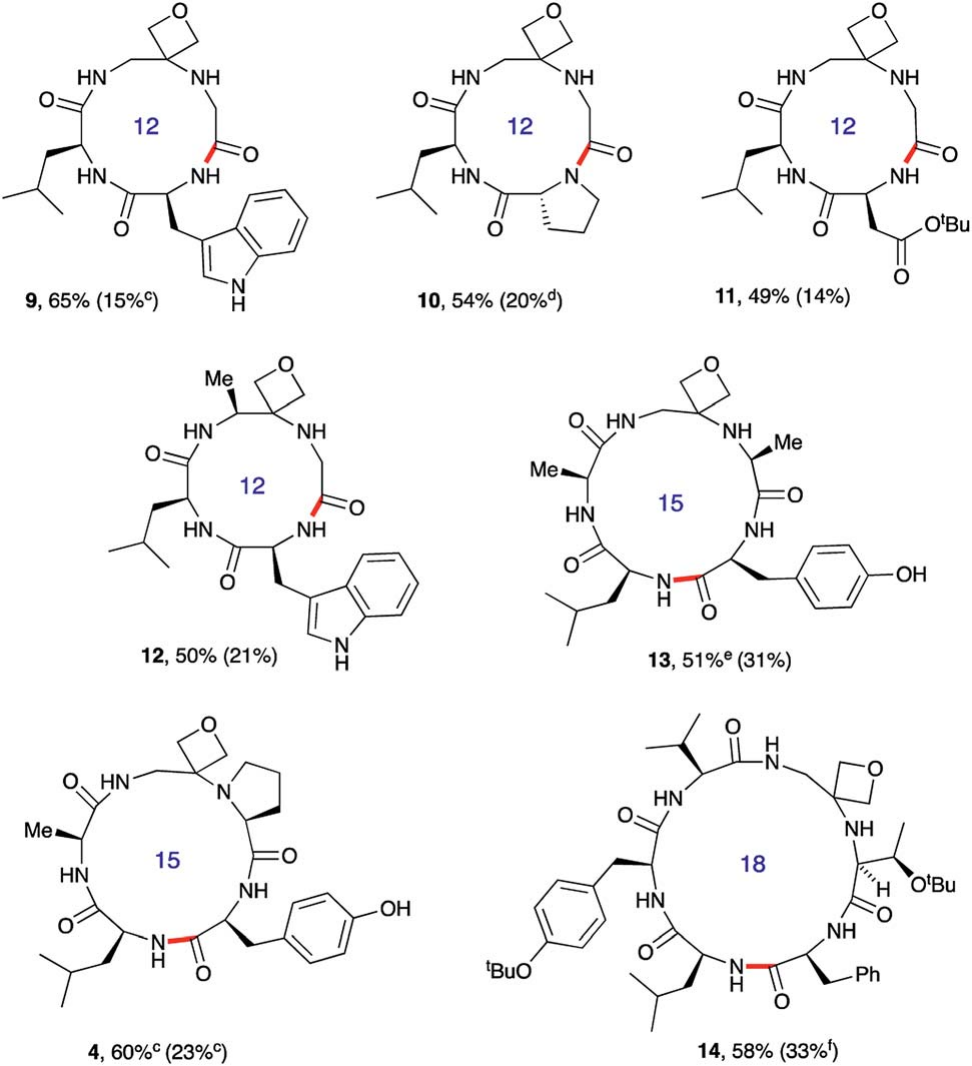
Oxetane modified cyclic peptides by head-to-tail macrocyclisation.^*a,b a*^Reaction conditions: DEPBT (2 equiv.), ^i^Pr_2_EtN (2 equiv.), DMF (0.001 M), RT. ^*b*^Bond formed in macrocyclisation indicated in red; yield in parenthesis for the cyclisation of linear peptide with C=O rather than oxetane in backbone. ^*c*^PyBOP as activator. ^*d*^Product isolated as the dimeric octapeptide. ^*e*^DEPBT: 51%, d.r. >95 : 5; PyBOP: 64%, d.r. 3 : 1. ^*f*^DMTMM tetrafluoroborate as activator. Data taken from ref. 13.

To gain a deeper understanding of reaction rates and the products formed, time-course studies of an inherently difficult to cyclise tetrapeptide WLGG (**6**) and corresponding oxetane modified WLGOxG (**7**) (where GOx = oxetane modified glycine) were undertaken. For this investigation, substrates containing a tryptophan residue were used to allow quantitative monitoring by UV spectroscopy. Both substrates were subjected to the DEPBT method and conversions monitored over 74 h. From these data, it is clear that the initial rate of formation of oxetane containing cyclic peptide **9** is considerably faster than **8**, even though both linear precursors **6** and **7** are consumed at similar rates (Fig. 2). The isolated yields for these macrocyclisations are given in Fig. 3 and again demonstrate the benefits of the oxetane motif. Appreciable quantities of the unwanted dimer and cyclodimer were produced in the cyclisation of **8**, explaining the lower conversion and isolated yield (see ESI†). In contrast, for the oxetane-modified peptide **7** clean conversion to the cyclic product **9** was observed. Taken together, these studies establish that head-to-tail ring closures to form small cyclic peptides proceed more quickly, give higher yields and produce less side products when one of the backbone carbonyl groups is replaced by an oxetane ring.

### Oxetane modified cyclic peptides: scope and limitations

To test the generality of these initial findings, the head-to-tail cyclisation of a variety of oxetane modified substrates was studied and the isolated yields compared with those for the unmodified systems (Fig. 3). Oxetane introduction clearly leads to appreciable improvements across a range of ring sizes as illustrated for tetrapeptides **9–12**, pentapeptides **4** and **13** and hexapeptide **14**. OMCPs were obtained in yields ranging from 49–65% whereas all yields for the control systems were substantially lower (0–33%). In a striking illustration of the power of this new method, cyclisation to tetrapeptide **10** was achieved in 54% yield in the presence of the oxetane modification, however cyclodimerisation to octapeptide was the predominant reaction pathway (20%) for the unmodified system. The successful formation of **12** containing an alanine modified residue confirms that this methodology is not simply limited to glycine substitution. Generally, DEPBT was favoured as the activating agent as it gave efficient cyclisations and minimised formation of difficult to separate diastereomers (*e.g.***13**: DEPBT: 51%, d.r. >95 : 5; PyBOP: 64%, d.r. 3 : 1) arising from epimerisation at the C-terminus prior to cyclisation. In some instances, impurities derived from the DEPBT reagent proved hard to remove, in which case PyBOP was used as an alternative.

**Fig. 4:**
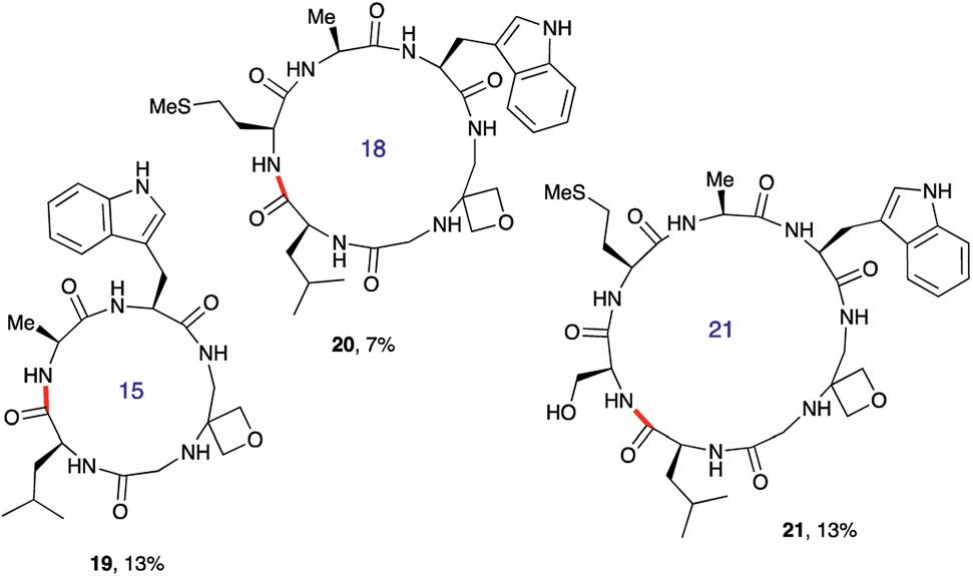
Additional oxetane modified macrocycles made using SPPS.^*a*,*b a*^Overall yield from first resin-bound amino acid including final RP-HPLC purification. For full details, see ESI.† ^*b*^Bond formed in macrocyclisation indicated in red.

Next, the synthesis of OMCPs by SPPS techniques was examined. In this way, it is possible to circumvent the need to purify any of the intermediates, simplifying and accelerating the process and potentially enabling automation. This was realised through the synthesis of cyclic tetrapeptide **18** in an impressive 39% yield from commercial H-Trp(Boc)-2-ClTrt (**15**) (Scheme 4). First, the linear peptide **17** was prepared from **15**, **16** and Fmoc-Leu-OH using Fmoc/^*t*^Bu SPPS,11*a* cleaved from the resin using TFE then cyclised to **18** using DEPBT conditions and purified by column chromatography. Macrocycles **19–21** based on different ring sizes were readily made through further generalisation of this SPPS approach (Fig. 4 and ESI†).

**Scheme 5:**
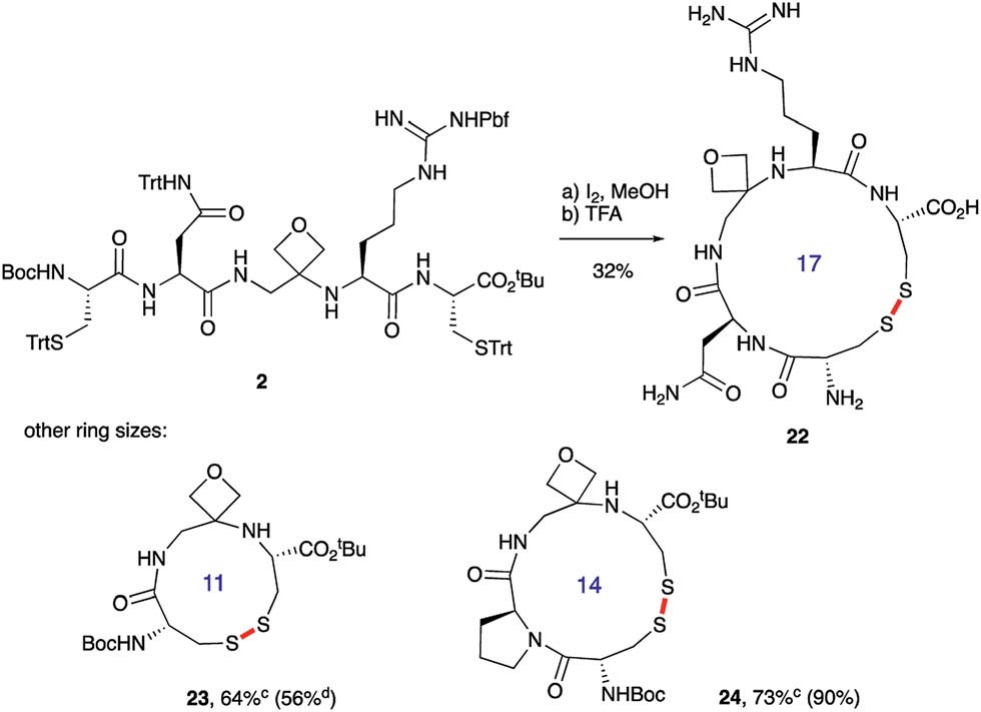
Oxetane modified cyclic peptides by disulphide bond formation.*^a^*^,^*^b^*^*a*^Reagents and conditions: (a) I_2_ (3.0 equiv.), MeOH, 1 h; (b) 70% TFA/20% CH_2_Cl_2_/10% TIS, 2.5 h. ^*b*^Bond formed in macrocyclisation indicated in red. ^*c*^TFA step omitted; yield in parenthesis for the cyclisation of linear peptide with C=O rather than oxetane in backbone. ^*d*^Data taken from ref. 14.

Macrocyclisations not involving head-to-tail ring closure have also been examined. Specifically, the formation of disulphide containing macrocycles through oxidative cyclisation of cysteine side chains has been explored (Scheme 5). Treatment of **2**, whose synthesis was described in Scheme 3, with iodine provided the 17-membered macrocycle by way of trityl deprotection and disulphide bond formation. Further reaction with TFA in the presence of the cation scavenger triisopropylsilane (TIS) facilitated removal of all the side chain protecting groups providing **22** after reverse-phase HPLC purification. Importantly, the four-membered oxetane ring survives the strongly acid conditions required to globally deprotect the side-chains including the arginine Pbf group. Smaller 11- and 14-membered macrocycles **23** and **24** were also conveniently produced in good yields using this methodology.

### Comparison with other amino acid modifications

**Scheme 6:**
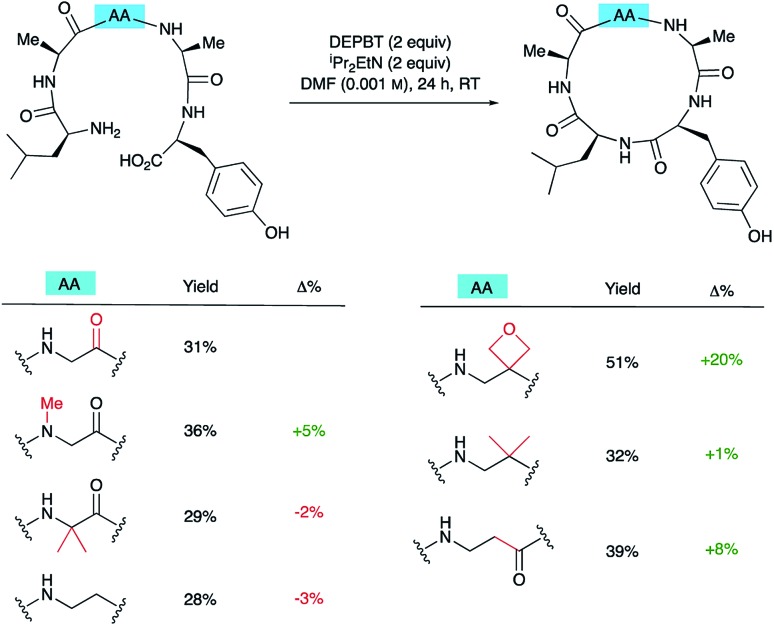
Synthesis of cLAGAY and impact of modifications on cyclisation.

To understand how significant these improvements are in the broader context of peptide macrocyclisation, a series of alternative modifications were made to the central G residue of LAGAY and the efficiencies of the ring closures compared. *N*-Methyl glycine, 2-methylalanine (Aib), ethylenediamine, dimethylethylenediamine and β-alanine were all introduced in place of the glycine (Scheme 6). These modifications could improve cyclisation efficiency by: (i) increasing the conformational flexibility of the peptide backbone by deletion of one of the amide bonds; (ii) enlarging the size of the macrocycle by introduction of an additional methylene group; (iii) bringing the reacting ends closer together by favouring the *cis*-amide conformation; or (iv) introducing a potentially beneficial Thorpe–Ingold effect. In fact, only the oxetane modification led to marked improvement in isolated yield of the derived cyclic peptides, suggesting that oxetane introduction is particularly beneficial.

**Fig. 5:**
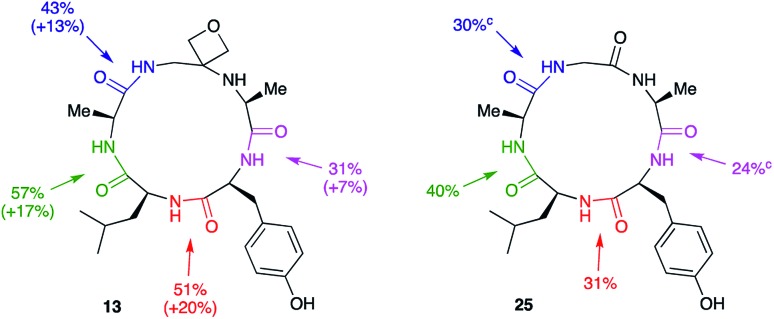
Macrocyclisation efficiency as a function of location of oxetane relative to forming amide bond.^*a*,*b a*^Reaction conditions: DEPBT (2 equiv.), ^i^Pr_2_EtN (2 equiv.), DMF (0.001 M), RT, 24 h. ^*b*^Average of two runs. ^*c*^Taken from ref. 12.

Macrocyclisation to pentapeptide **13** by formation of all four possible amide bonds was examined to see whether the location of the oxetane relative to the amide bond being formed is important (Fig. 5). The yields were compared with those obtained making pentapeptide **25** by the same disconnections. This approach removes inherent differences in cyclisation efficiency associated with amide bond formation between different amino acid residues. For all four pairs of cyclisation studied, the oxetane modified system outperformed the C=O system leading to higher product yields. However, larger improvements are seen when the modification is more centrally located along the precursor backbone.

### Structural impact of oxetane modification

**Fig. 6:**
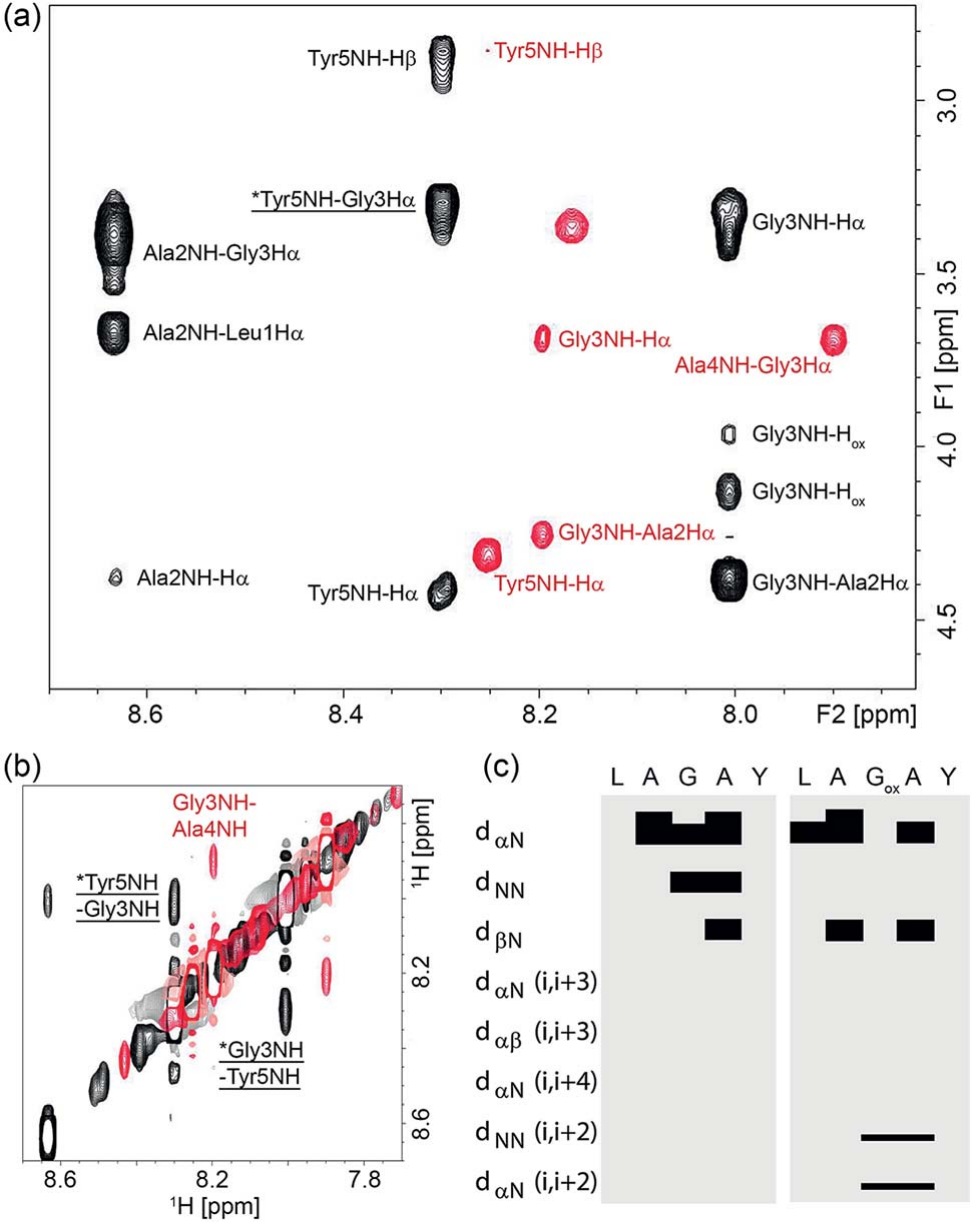
NOESY spectra of LAGOxAY-OMe (black) and LAGAY-OMe (red) collected with a mixing time of 400 ms for samples containing 30 mM peptide solubilised in *d*_6_-DMSO. Both spectra were collected at 298 K at a field strength of 700 MHz. Panel (a) shows the amide to Hα Hβ dipolar coupling in the fingerprint region, and panel (b) shows the sequential and medium-range amide proton coupling. Medium range NOEs are indicated by an asterisk and underlined. (c) NOE-connectivity maps of LAGOxAY-OMe and LAGAY-OMe based on NOE spectra collected with 250–800 ms mixing times. Each sequential and medium-range coupling is indicated on the left, and a bar is used to join the coupled residues if a coupling is observed.

To understand why oxetane incorporation improves the macrocyclisations, we undertook solution-state NMR studies on the linear cyclisation precursor LAGAY and its oxetane modified variant. In order to accurately mimic the active ester intermediate without the effect of zwitterion formation, which may alter the solution conformation, we chose to study the corresponding methyl esters: LAGAY-OMe and LAGOxAY-OMe. 2D ^1^H–^1^H TOCSY and NOESY spectra were acquired as detailed in the ESI† in order to assign the ^1^H signals in both peptides and probe changes in conformation, especially near the site of modification. As shown in Fig. 6a and b, oxetane modified LAGOxAY-OMe yielded a larger number of nuclear Overhauser enhancements (NOEs) than unmodified LAGAY-OMe. The observed NOEs were used to construct an NOE-connectivity map (Fig. 6c) which summarises sequential (*i.e.* residue *i* to residue *i* + 1) and medium-range NOEs that are commonly observed in α-helices, β-sheets, and turns.^15^ While sequential NOEs are observable in both peptides (*d*_αN_, *d*_NN_, *d*_βN_), medium-range NOEs are *only* observed in peptide LAGOxAY-OMe containing the modification. Specifically, *d*_NN_ (*i*, *i* + 2) and *d*_αN_ (*i*, *i* + 2) NOEs are clearly observed in the oxetane modified peptide between residues Gly3 and Tyr5 indicating their close proximity, data consistent with the formation of a turn. These NMR studies provide the first direct experimental evidence that the oxetane modification is indeed turn-inducing, and brings the peptide termini closer in space to facilitate efficient cyclisation (Scheme 6).

To gain insights into how oxetane modification impacts the structure of the derived cyclic peptides, molecular dynamics (MD) computer simulations with distance restraints derived from NMR experiments were carried out on cLAGAY (**25**) and cLAGOxAY (**13**) in DMSO. 2D ^1^H–^1^H NOESY spectra were used to compile a set of nuclear Overhauser enhancement (NOE) – derived experimental distance restraints that were incorporated into MD simulations using the CHARMM forcefield^16^ with modifications for the oxetane ring.^7^ All simulations were performed using the Gromacs 5.1.4 simulation package.^17^ Each cyclic peptide was simulated for 100 ns in DMSO at 500 K and from this trajectory five distinct conformations of each peptide were selected for a further 100 ns at 300 K. For these small, relatively rigid peptides, 500 ns total simulation time was sufficient for the simulations to converge, given that no new structural information was obtained after ∼320 ns.

**Fig. 7:**
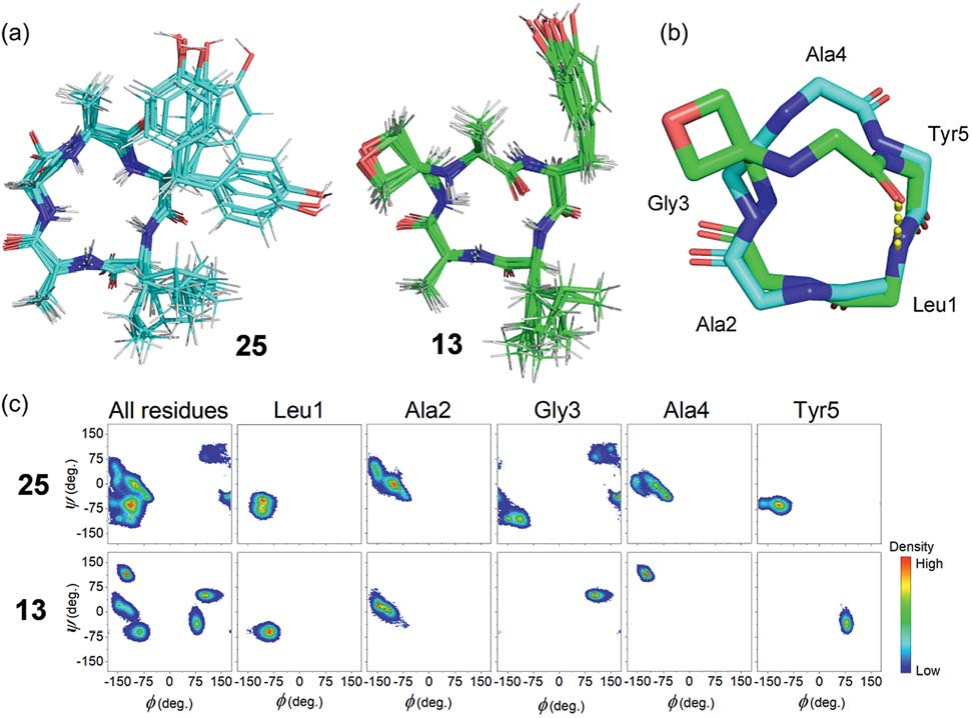
(a) NOE-restrained Molecular Dynamics simulations of cLAGAY (**25** in cyan) and oxetane modified cLAGOxAY (**13** in green). For each, a total of 10 representative structures are overlaid. (b) Peptide backbones of **13** and **25** with side-chains omitted for clarity. For **13**, intramolecular H-bond is indicated (yellow dots). (c) Ramachandran plots showing dihedral angles *φ* against *Φ* for the amino acid residues within **13** and **25**.

Cluster analysis was performed on 12 500 structures extracted from the trajectories at 40 ps intervals. Five and four distinct structures were found for peptides **25** and **13** respectively, with the first three clusters representing ≥99.9% of the entire trajectory for both peptides. The trajectories were dominated by conformations belonging to a single cluster that represented 93.7% and 99.1% of the total population of **25** and **13**, respectively (see ESI†). Ten representative structures from the dominant cluster of each peptide are overlaid in Fig. 7a. The cluster analysis indicates that the backbones of both structures have little conformational flexibility (as expected for small cyclic peptides), but that the OMCP is slightly more rigid. H-bond analysis revealed that the unmodified cyclic peptide **25** did not form any significant intra-peptide H-bonds whereas OMCP **13** was characterised by a H-bond with 86.3% occupancy between the NH group of Leu1 and the O atom of Ala4 (see Fig. 7b). The backbones of **25** and **13** in their dominant configurations are shown overlaid in Fig. 7b. Oxetane introduction only alters the backbone conformation of residues in proximity to the modification itself. In this case, an inversion of the amide bond between Ala4 and Tyr5 arises on oxetane introduction, which allows the structure to be stabilised by an intra-peptide H-bond across the macrocycle. Ramachandran plots, showing the distribution of the *Φ* and *φ* backbone dihedral angles of each residue and the entire peptide (averaged over the entire simulation trajectory), are presented in Fig. 7c. The Ramachandran plots show that **13** explores less of the *Φ*/*φ* space than **25**, which supports our observation above that **13** is more rigid. The Ramachandran plots also highlight the effect of the oxetane introduction on the structure of the residues in proximity to the modification, with Gly3, Ala4 and Tyr5 shifting to different regions of *Φ*/*φ* space following the introduction of the oxetane, which is in agreement with the visual representation of the backbone in Fig. 7b.

### Metalloprotease inhibition studies

Studies to explore the impact of oxetane modification on the bioactivity of the derived cyclic peptides were undertaken. cCNGRC (**26**) is known to target Aminopeptidase N (APN), a transmembrane zinc-dependent metalloprotease involved in a variety of processes, including blood pressure regulation, cell migration, viral uptake, cell survival, and angiogenesis.^18^,^19^ Both oxetane modified derivative **22** and cCNGRC (**26**) were examined for their inhibitory activity towards porcine APN using a spectrophotometric assay (Table 2).^19^ Although both compounds are rather weak inhibitors, the fact that similar IC_50_ values were observed for **22** and **26** suggests that the oxetane motif is an excellent bioisostere of the amide bond it replaces.

**Table 2:** Relative inhibitory effects of **22** and cCNGRC (**26**) against porcine Aminopeptidase N (APN)^*a*^

Entry	Compound	IC_50_ (μM)
1	**22**	175
2	cCNGRC (**26**)	212^*b*^
3	bestatin^*c*^	4.1^*b*^

^*a*^APN and the substrate l-leucine-*p*-nitroanilide were incubated for 1 h in the presence of gradient concentrations of inhibitors and formation of the product *p*-nitroaniline measured reading absorbance at 405 nm. IC_50_ values were calculated from concentration–response curves (see ESI).

^*b*^Values slightly lower than those reported in ref. 19.

^*c*^Positive control.

## Conclusions

A new way to make small macrocyclic peptides has been discovered and evaluated. The approach relies on the introduction of an oxetane into the backbone of the cyclisation precursor. Two strategies for the assembly of these molecules were developed involving either chain elongation of the peptide by reaction with 3-(nitromethylene)oxetane, or alternatively through coupling preformed Fmoc-protected dipeptide building blocks containing this modification. The first is convenient for the insertion of glycine modifications, whereas the second is more general, being suitable for introduction of oxetane modified derivatives of other l-amino acids,^10^ and for integration with conventional SPPS methods.11*a*

Oxetane incorporation led to improvements in all the head-to-tail cyclisations studied as assessed by isolated yields, reaction rates and analysis of product distributions. The method works across a range of ring sizes and enabled the synthesis of a range of cyclic tetra-, penta-, hexa- and heptapeptides. Major improvements in yields are seen using glycine modified residues, with levels of epimerisation and dimerisation significantly reduced. The extension of this method to other modified l-amino acids was demonstrated. It can also be applied to side-chain to side-chain macrocyclisations, specifically oxidative ring closure of cysteine residues by disulphide bond formation. As a tool to improve head-to-tail macrocyclisations, our evidence suggests that oxetane incorporation is superior to a number of other common backbone modifications. Importantly, OMCPs are compatible with the harsh acidic conditions needed to deprotect amino acid side chains.

A key question we sought to address in this study was why does oxetane introduction improve macrocyclisations? For maximum benefit, we have shown that the oxetane is best located centrally along the peptide backbone, an observation consistent with the hypothesis that this modification is turn-inducing.^7^ Moreover, using solution-state NMR spectroscopy, we have obtained the first direct experimental evidence that oxetane introduction induces a turn in the peptide backbone, through the observation of *d*_NN_ (*i*, *i* + 2) and *d*_αN_ (*i*, *i* + 2) NOEs. The formation of a turn presumably helps bring the termini closer together enabling the macrocyclisation.

To be useful in drug discovery programmes as well as being easy to make, OMCPs must also have useful properties. Intuitively, one might expect oxetane introduction to only alter the backbone configuration of the cyclic peptide close to the site of modification. Indeed, this was borne out in detailed NOE-restrained MD simulations conducted on **13**. The full retention of bioactivity of **22** relative to cCNGRC suggests that the replacement of a C=O amide bond by an oxetane ring has little impact on binding, making it a viable bioisosteric replacement. Next steps will involve a fuller assessment of the properties of OMCPs and their application in drug discovery programmes.

## Conflicts of interest

There are no conflicts to declare.

## Supplementary Material

Supplementary information
