# Supplementary material for: Macrocyclisation of small peptides enabled by oxetane incorporation
Source: Chem Sci. 2019 Jan 3;10(8):2465–72. doi: 10.1039/c8sc05474f (PMC6385813; doi:10.1039/c8sc05474f)

Electronic Supplementary Material (ESI) for Chemical Science.
This journal is © The Royal Society of Chemistry 2019

Macrocyclisation of Small Peptides Enabled by Oxetane Incorporation

Stefan Roesner, George J. Saunders, Ina Wilkening, Eleanor Jayawant, Joanna V. Geden, Paul Kerby, Ann M. Dixon, Rebecca Notman and Michael Shipman*

Department of Chemistry, University of Warwick, Gibbet Hill Road, Coventry, CV4 7AL, U.K.

E-mail: M.Shipman@warwick.ac.uk

Supporting Information

Table of Contents

1. General experimental information S2
2. Detailed procedures and analytical data S3
   2.1 Preparation of cyclic pentapeptide **4** S3
   2.2 Preparation of cyclic pentapeptide **5** S7
   2.3 Preparation of cyclic tetrapeptides **9**, **10**, and **11** S11
   2.4 Preparation of cyclic tetrapeptides **8** and **48** and cyclic octapeptide **42** and **45** S17
   2.5 Preparation of cyclic tetrapeptide **12** and **58** S23
   2.6 Preparation of cyclic pentapeptide **13** S29
   2.7 Preparation of cyclic pentapeptide **25** S44
   2.8 Preparation of cyclic pentapeptide **92** S50
   2.9 Preparation of cyclic pentapeptide **98** S54
   2.10 Preparation of cyclic pentapeptide **102** S58
   2.11 Preparation of cyclic pentapeptide **107** S61
   2.12 Preparation of cyclic pentapeptide **112** S64
   2.13 Preparation of cyclic hexapeptide **14** S68
   2.14 Preparation of cyclic peptides **18-21** via SPPS S74
   2.15 Preparation of cyclic disulfide-bridged pentapeptide **22** S78
   2.16 Preparation of cyclic disulfide-bridged tripeptide **23** S84
   2.17 Preparation of cyclic disulfide-bridged tetrapeptide **24** S86
   2.18 Preparation of cyclic disulfide-bridged tetrapeptide **136** S89
   2.19 Preparation of pentapeptides **137** and **138** S91
3. Kinetic measurement of the cyclization reaction S93
4. *In vitro* inhibition assay of Aminopeptidase N with oxetane modified peptide **22** and parent peptide **26** S99
5. NMR analysis of pentapeptides **137** and **138** S100
6. NMR analysis of cyclic pentapeptides **13** and **25** S102
7. Molecular dynamics simulations S106
8. References S110
9. ^{1}H NMR and ^{13}C NMR spectra S111

1. General experimental information

Reaction mixtures were stirred magnetically. All chemicals were purchased from Acros Organics, Alfa Aesar, Fluorochem or Sigma-Aldrich and used as received unless otherwise mentioned. TNBS test kit picrylsulfonic acid (ca. 1% in DMF) 10 mL/*N*,*N*-diisopropylethylamine (ca. 10% in DMF) 10 mL for detection of primary amines was purchased from TCI Chemicals. Anhydrous solvents were purchased from Sigma-Aldrich or Acros Organics in Sure-Seal™ bottles. All other solvents were reagent grade and used as received. Petroleum ether refers to the fraction that boils in the range of 40–60 °C. Boc-Tyr(Bn)-OBn,[1] Boc-Ala-OBn,[1] Boc-Leu-OBn,[1] NO_2-GOx-Gly-OBn,[2] TsOH·H-Gly-Gly-OBn,[3] NO_2-AOx-(*R*)-CH(Me)Ph,[4] and Boc-Ala-Gly-OBn,[5] were synthesised according to known literature procedures.

^{1}H Nuclear Magnetic Resonance (NMR) spectra were recorded in $CDCl_3$, CD_2Cl_2, CD_3OD, DMSO-*d6*, CD_3CN, toluene-*d8*, or D_2O on a Bruker HD400 (400 MHz), AV500 (500 MHz) or AV600 (600 MHz) Fourier transform spectrometer. Chemical shifts (δ_H) are quoted in parts per million (ppm) and referred to the residual protic solvent signals of $CDCl_3$ (7.26 ppm), CD_2Cl_2 (5.32 ppm), CD_3OD (3.31 ppm), DMSO-*d6* (2.50 ppm), CD_3CN (1.94 ppm), toluene-*d8* (2.09 ppm) or D_2O (4.79 ppm). ^{1}H NMR coupling constants are reported in hertz and refer to apparent multiplicities. Data are reported as follows: chemical shift, multiplicity (s = singlet, br. s = broad singlet, d = doublet, t = triplet, q = quartet, quint = quintet, sext = sextet, sept = septet, m = multiplet, dd = doublet of doublet, etc.), coupling constant, integration, and assignment. ^{13}C NMR spectra were recorded at 101, 126 or 151 MHz. Chemical shifts (δ_C) are quoted in ppm referenced to $CHCl_3$ (77.16 ppm), CH_2Cl_2 (54.00 ppm), CD_3OD (49.00 ppm), DMSO-*d6* (39.52 ppm), CD_3CN (1.32 ppm) or toluene-*d8* (20.40 ppm). NMR assignments were deduced using 2D experiments (COSY, HSQC and HMBC). NH and OH are not visible in protic solvents (CD_3OD, D_2O).

Two-dimensional NMR spectra were collected on an Avance 700 MHz spectrometer (Bruker Biospin, UK) equipped with a triple resonance inverse cryoprobe with Z-gradients. Data were acquired for peptide samples in 3 mm tubes at 25 °C. Peptides **137** and **138** were measured in DMSO-d_6 at 30 mM and cyclic peptides **13** and **25** were dissolved in DMSO-d_6 to final peptide concentrations ranging from 2–60 mM. ^{1}H 1D spectra were collected to determine if any self-association was occurring at high concentration (Figure S5). For peptide samples at concentrations of ca. 60 mM, ^{1}H-^{1}H TOCSY and NOESY spectra were recorded with 4096 × 256 data points and mixing times ranging from 70–140 ms and 100–800 ms, respectively. All data were processed using TopSpin 3.2, and analysed using CcpNmr Analysis 2.4.2 software. ^{1}H assignment was completed (peak assignments and representative 2D spectra are shown in Figures S6-S7) and NOE buildup curves were plotted for 4 inter-residue NOE peaks (after normalisation to the volumes of the Tyr aryl peaks, which have a fixed distance) to ensure that NOE restraints were estimated from a spectrum in the linear region of the curve, and not from a spectrum experiencing spin-diffusion (see Figure S8). NOESY spectra with mixing times of 250 ms were selected for estimation of distance restraints in MD simulations. The inter-residue NOE restraints input into the simulations are shown in Table S1, and an evaluation of goodness-of-fit to these restraints in the resulting structures are shown in Table S2. Secondary structure plots were prepared using CcpNmr Analysis 2.4.2, using data from 250, 400, 600 and 800 ms NOESY experiments.

Low-resolution mass spectra were recorded on an Agilent 6130B single Quad (ESI) instrument. High-resolution mass spectra were recorded using a Bruker MaXis Impact. All infrared spectra were recorded on the neat compounds using a Bruker ALPHA-Platinum FTIR spectrometer, irradiating between 4000 cm^{-1} and 600 cm^{-1}. Only strong and selected absorbances (ν_{max}) are reported. Analytical TLC was performed on aluminium backed silica plates (Merck, Silica Gel 60 F_{254}, 0.25 mm). Compounds were visualised by fluorescence quenching or by staining the plates with 5% solution of phosphomolybdic

acid ($H_3PMo_{12}O_{40}$) in EtOH or 1% solution of potassium permanganate ($KMnO_4$) in water followed by heating. Flash column chromatography was performed on silica gel (Aldrich, Silica Gel 60, 40–63 µm). All mixed solvent eluents are reported as v/v solutions. Optical rotations were obtained using an AA-1000 polarimeter at 589 nm (Na D-line) in a cell with a path length of 2 dm. Specific rotation values are given in (deg mL)/(g dm). Melting points were measured with a Gallenkamp melting point apparatus.

LC-MS analysis were conducted on a Bruker Amazon X or Bruker HCT Ultra ETD instrument with a PLRP-S column from Agilent (100 Å, 8 µm, 150 × 4.6 mm) and UV detection at 210 nm. A binary gradient of acetonitrile (0.1% formic acid) and water (0.1% formic acid) was used at a flow rate of 1 mL/min. Peptides **19**–**22** and **26** were purified by preparative HPLC on an Agilent PLRP-S RP (100 Å, 8 µm, 150 × 25mm) column on an Agilent Infinity 1260 HPLC system. The mobile phase consisted of a gradient of water and acetonitrile (HPLC grade) at a flow rate of 10 mL/min, with UV detection at 210, 254 and 280 nm.

2. Detailed procedures and analytical data

2.1 Preparation of cyclic pentapeptide ***4***

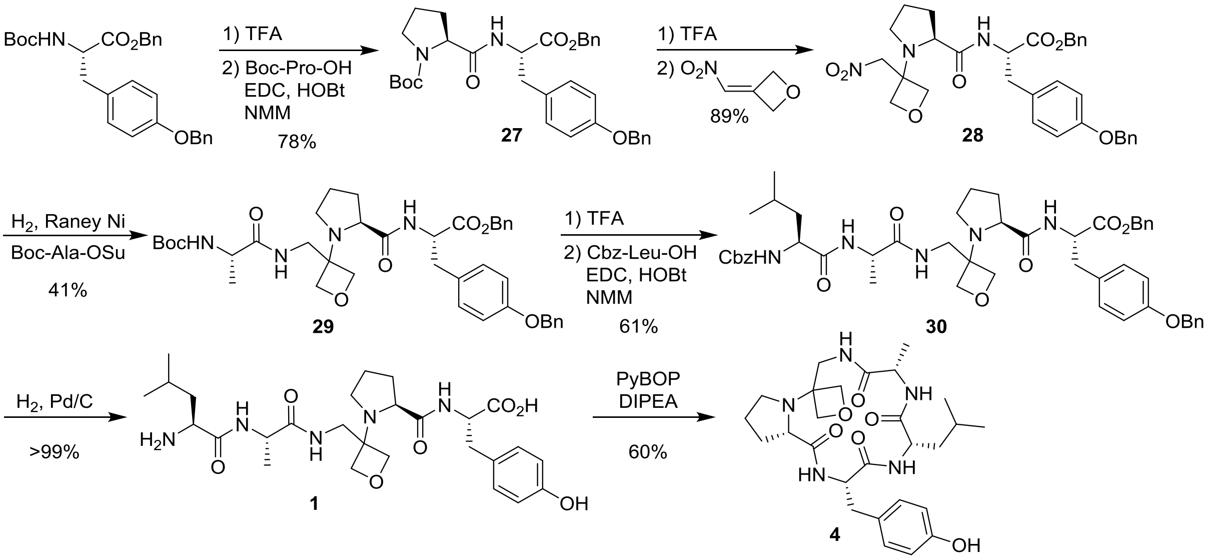

Boc-Pro-Tyr(Bn)-OBn (27)

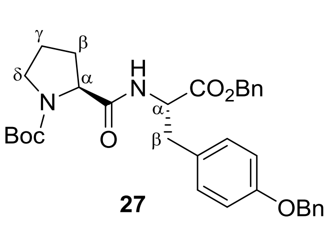

27

To a solution of Boc-Tyr(Bn)-OBn[1] (11.4 g, 24.8 mmol, 1.0 equiv) in CH_2Cl_2 (25 mL) was added TFA (25 mL) and the mixture was stirred at room temperature for 1 h *(Caution – gas evolution!)*. The reaction mixture was concentrated under reduced pressure and the resulting residue repeatedly dissolved in CH_2Cl_2 (3 × 250 mL) and concentrated under reduced pressure to give the crude amine. The residue was dissolved in CH_2Cl_2 (250 mL), Boc-Pro-OH (5.88 g, 27.3 mmol, 1.1 equiv), EDC·HCl (5.23 g, 27.3 mmol, 1.1 equiv), HOBt·H_2O (3.69 g, 27.3 mmol, 1.1 equiv) and NMM (10.9 mL, 99.2 mmol, 4.0 equiv) were added subsequently, and the reaction mixture was stirred at room temperature for 24 h. The reaction mixture was diluted with EtOAc (250 mL) and washed with brine (250 mL), dried over Na_2SO_4, filtered and concentrated under reduced pressure. The residue was purified by column chromatography (SiO_2, PE/EtOAc 4:1) to give dipeptide Boc-Pro-Tyr(Bn)-OBn (**27**) (10.8 g, 19.3 mmol, 78%) as a white foam. $\mathbf{R_f}$ (PE/EtOAc 4:1) 0.30; **mp** 105–108 °C; ^{1}**H NMR** (600 MHz, DMSO-*d6*) δ_H 7.70 (d, *J* = 7.5 Hz, 1H, NH), 7.44–7.27 (m, 10H, ArH), 7.11 (d, *J* = 8.4 Hz, 2H, ArH), 6.89 (d, *J* = 8.4 Hz, 2H, ArH), 5.10 (s, 2H, CH_2Ph), 5.07 (s, 2H, CH_2Ph), 4.61–4.56 (m, 1H, CHα-Tyr), 4.13 (dd, *J* = 8.5, 3.0 Hz, 1H, CHα-

Pro), 3.35–3.26 (m, 2H, CH_2δ-Pro), 3.04 (dd, J = 14.1, 6.0 Hz, 1H, C*H*Hβ-Tyr), 2.97–2.92 (dd, J = 18.0, 8.0 Hz, 1H, CH*H*β-Tyr), 2.00 (dq, J = 16.3, 8.3 Hz, 1H, C*H*Hβ-Pro or C*H*Hγ-Pro), 1.78–1.66 (m, 3H, CH*H*β-Pro or CH*H*γ-Pro, CH_2β-Pro or CH_2γ-Pro), 1.32 (s, 9H, 3 × CH_3, Boc); **^{13}C NMR** (101 MHz, DMSO-*d6*) δ_C 172.7 (C=O), 171.5 (C=O), 157.1 (C=O, Boc), 153.3 (C), 137.2 (C), 135.7 (C), 130.0 (CH), 129.2 (C), 128.41 (CH), 128.35 (CH), 128.1 (CH), 128.0 (CH), 127.8 (CH), 127.5 (CH), 114.6 (CH), 78.3 (C, Boc), 69.1 (CH_2, Bn), 66.0 (CH_2, Bn), 59.3 (CH, α-Pro), 53.9 (CH, α-Tyr), 46.4 (CH_2, δ-Pro), 35.7 (CH_2, β-Tyr), 30.7 (CH_2, β-Pro or CH_2, γ-Pro), 28.1 (CH_3, Boc, minor rotamer), 27.8 (CH_3, Boc, major rotamer), 22.9 (CH_2, β-Pro or CH_2, γ-Pro); **ν_{max}** (neat) = 2977, 1734, 1686, 1669, 1508, 1160, 734 cm^{-1}; **MS** (ESI^+) *m/z* 581 $[M+Na]^+$; **HRMS** (ESI^+) calcd. for $C_{33}H_{38}N_2NaO_6$ $[M+Na]^+$ 581.2622, found 581.2624; $[\boldsymbol{\alpha}]_D^{27}$ –50.0 (*c* 0.10, $CHCl_3$).

NO_2-GOx-Pro-Tyr(Bn)-OBn (28)

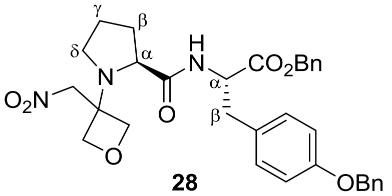

To a solution of Boc-Pro-Tyr(Bn)-OBn (**27**) (6.72 g, 12.0 mmol, 1.0 equiv) in CH_2Cl_2 (12.0 mL) was added TFA (12.0 mL) and the mixture was stirred at room temperature for 1 h. The mixture was concentrated under reduced pressure and the resulting residue repeatedly dissolved in CH_2Cl_2 (3 × 50 mL) and concentrated under reduced pressure to give the crude amine. In a second reaction vessel, oxetane-3-one (1.41 mL, 24.1 mmol, 2.0 equiv), nitromethane (1.82 mL, 33.7 mmol, 2.8 equiv) and triethylamine (672 μL, 4.82 mmol, 0.4 equiv) were combined at 0 °C and stirred for 1 h at room temperature. The mixture was dissolved in anhydrous CH_2Cl_2 (92 mL), cooled to –78 °C, and triethylamine (6.72 mL, 24.1 mmol, 4.0 equiv) was added followed by dropwise addition of methanesulfonyl chloride (1.86 mL, 24.1 mmol, 2.0 equiv). The reaction mixture was stirred at –78 °C for 1.5 h and a solution of the crude amine and triethylamine (2.52 mL, 18.1 mmol, 1.5 equiv) in anhydrous CH_2Cl_2 (20 mL) was added slowly *via* syringe. The reaction mixture was allowed to warm to room temperature and stirred for 16 h. A saturated solution of NH_4Cl (100 mL) was added and stirred for 10 min. The layers were separated and the aqueous one extracted with CH_2Cl_2 (2 × 60 mL) and EtOAc (2 × 60 mL). The combined organic phases were washed with saturated aqueous $NaHCO_3$ solution (100 mL), brine (100 mL), dried over Na_2SO_4, filtered and concentrated under reduced pressure. The residue was purified by column chromatography (SiO_2, EtOAc/PE 4:1) to yield NO_2-GOx-Pro-Tyr(Bn)-OBn (**28**) (6.10 g, 10.6 mmol, 89%) as an orange oil. **R_f** (EtOAc/PE 4:1) 0.31; **^{1}H NMR** (500 MHz, $CDCl_3$) δ_H 7.44–7.28 (m, 10H, ArH), 7.25 (m, 1H, NH), 7.04 (d, J = 8.5 Hz, 2H, ArH), 6.85 (d, J = 8.5 Hz, 2H, ArH), 5.21–5.12 (m, 2H, CH_2Ph), 5.04 (s, 2H, CH_2Ph), 4.94 (d, J = 13.1 Hz, 1H, OC*H*H-Ox), 4.73 (d, J = 13.1 Hz, 1H, OCH*H*-Ox), 4.70–4.63 (m, 2H, OC*H*H-Ox, CHα-Tyr), 4.62 (d, J = 7.4 Hz, 1H, C*H*HGOx), 4.56 (d, J = 8.5 Hz, 1H, OCH*H*-Ox) 4.35 (d, J = 7.4 Hz, 1H, CH*H*GOx), 4.01 (dd, J = 10.0, 1.8 Hz, 1H, CHα-Pro), 3.22 (dd, J = 14.0, 5.1 Hz, 1H, C*H*Hβ-Tyr), 3.01 (t, J = 7.3 Hz, 1H, C*H*Hδ-Pro), 2.86 (dd, J = 14.0, 9.9 Hz, 1H, CH*H*β-Tyr), 2.37 (ddd, J = 11.3, 8.0, 5.9 Hz, 1H, CH*H*δ-Pro), 2.05–1.95 (m, 1H, C*H*Hβ-Pro or C*H*Hγ-Pro), 1.94–1.87 (m, 1H, C*H*Hβ-Pro or C*H*Hγ-Pro), 1.63 (dt, J = 12.1, 5.9 Hz, 1H, C*H*Hβ-Pro or C*H*Hγ-Pro), 1.25–1.13 (m, 1H, C*H*Hβ-Pro or C*H*Hγ-Pro); **^{13}C NMR** (126 MHz, $CDCl_3$) δ_C 174.2 (C=O), 171.2 (C=O), 157.9 (C), 137.1 (C), 135.5 (C), 130.2 (CH), 128.8 (C), 128.7 (CH), 128.5 (CH), 128.0 (CH), 127.5 (CH), 115.1 (CH), 79.2 (OCH_2), 79.1 (OCH_2), 75.3 (CH_2, GOx), 70.1 (CH_2, Bn), 67.3 (CH_2, Bn), 62.8 (C, Ox), 61.4 (CH, α-Pro), 53.6 (CH, α-Tyr), 49.2 (CH_2, δ-Pro), 36.6 (CH_2, β-Tyr), 31.4 (CH_2, β-Pro or CH_2, γ-Pro), 24.3 (CH_2, β-Pro or CH_2, γ-Pro). *N.B.* One aromatic CH signal not observed; **ν_{max}** (neat) = 3032, 2873, 1740, 1668, 1549, 1509, 1121, 982, 666 cm^{-1}; **MS** (ESI^+) *m/z* 596 $[M+Na]^+$; **HRMS:** (ESI^+) calcd. for $C_{32}H_{35}N_3NaO_7$ $[M+Na]^+$ 596.2367, found 596.2370; $[\boldsymbol{\alpha}]_D^{27}$ +13.5 (*c* 0.10, $CHCl_3$).

Boc-Ala-GOx-Pro-Tyr(Bn)-OBn (29)

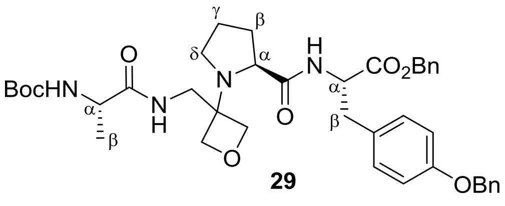

To a solution of NO_2-GOx-Pro-Tyr(Bn)-OBn (**28**) (7.80 g, 13.6 mmol, 1.0 equiv) in THF (136 mL) was added Boc-Ala-OSu (5.84 g, 20.4 mmol, 1.5 equiv) and Raney Ni (slurry in H_2O, 14 mL). The solution was placed under an atmosphere of nitrogen, evacuated and filled with hydrogen (balloon). The reaction mixture was stirred vigorously for 4.0 h at room temperature. Then, the mixture was filtered through a plug of Celite eluting with EtOAc, concentrated under reduced pressure, the filtrate was suspended in EtOAc (100 mL), washed with saturated Na_2CO_3 (3 × 100 mL), dried over Na_2SO_4 and concentrated under reduced pressure. Boc-Ala-GOx-Pro-Tyr(Bn)-OBn (**29**) was afforded after purification by column chromatography (SiO_2, CH_2Cl_2/EtOAc 7:3) as an off-white solid (3.99 g, 5.59 mmol, 41%). **R_f** (CH_2Cl_2/EtOAc 7:3) 0.22; **mp** 72–74 °C; **^{1}H NMR** (500 MHz, $CDCl_3$) δ_H 7.73 (d, *J* = 9.1 Hz, 1H, NH), 7.44–7.29 (m, 10H, ArH), 7.02 (d, *J* = 7.8 Hz, 2H, ArH), 6.86 (s, 1H, NH), 6.82 (d, *J* = 8.4 Hz, 2H, ArH), 5.26 (d, *J* = 12.1 Hz, 1H, C*H*HPh), 5.19–5.10 (m, 1H, CH*H*Ph), 5.01 (s, 2H, CH_2Ph), 4.92 (td, *J* = 8.8, 5.3 Hz, 1H, CHα-Tyr), 4.63 (d, *J* = 6.9 Hz, 1H, OC*H*H-Ox), 4.39 (d, *J* = 7.2 Hz, 1H, OC*H*H-Ox), 4.34 (d, *J* = 6.9 Hz, 1H, OCH*H*-Ox), 4.30 (d, *J* = 7.2 Hz, 1H, OCH*H*-Ox), 4.11 (quint, *J* = 6.9 Hz, 1H, CHα-Ala), 3.94 (dd, *J* = 14.0, 6.8 Hz, 1H, C*H*HGOx), 3.85–3.80 (m, 1H, CHα-Pro), 3.56 (dd, *J* = 14.1, 3.8 Hz, 1H, CH*H*GOx), 3.19 (dd, *J* = 14.0, 5.0 Hz, 1H, C*H*Hβ-Tyr), 3.05 (t, *J* = 7.6 Hz, 1H, C*H*Hδ-Pro), 2.99 (dd, *J* = 14.0, 8.6 Hz, 1H, CH*H*β-Tyr), 2.47 (ddd, *J* = 10.8, 8.8, 5.9 Hz, 1H, CH*H*δ-Pro), 2.08–1.97 (m, 1H, C*H*Hβ-Pro or C*H*Hγ-Pro), 1.77–1.64 (m, 2H, C*H*Hβ-Pro or C*H*Hγ-Pro), 1.59–1.48 (m, 1H, C*H*Hβ-Pro or C*H*Hγ-Pro), 1.42 (s, 9H, 3 × CH_3, Boc), 1.26 (d, *J* = 8.1 Hz, 3H, CH_3β-Ala). *N.B.* Peak at 1.77–1.64 overlaps with residual H_2O peak, Boc NH not observed; **^{13}C NMR** (126 MHz, $CDCl_3$) δ_C 175.0 (C=O), 173.9 (C=O), 173.4 (C=O), 157.8 (C), 155.7 (C=O, Boc), 137.1 (C), 135.0 (C), 130.4 (CH), 128.72 (CH), 128.68 (CH), 128.7 (CH), 128.6 (CH), 128.1 (CH), 127.6 (CH), 114.9 (CH), 79.8 (C, Boc), 78.3 (OCH_2), 76.5 (OCH_2), 70.1 (CH_2, Bn), 67.8 (CH_2, Bn), 62.8 (C, Ox), 61.7 (CH, α-Pro), 52.7 (CH, α-Tyr), 50.0 (CH, α-Ala), 48.8 (CH_2, δ-Pro), 45.1 (CH_2, GOx), 37.1 (CH_2, β-Tyr), 31.6 (CH_2, β-Pro or CH_2, γ-Pro), 28.5 (CH_3, Boc), 24.5 (CH_2, β-Pro or CH_2, γ-Pro), 18.4 (CH_3, β-Ala). *N.B.* One aromatic CH signal not observed; **ν_{max}** (neat) = 3323, 2970, 2935, 1736, 1661, 1510, 1163, 734 cm^{-1}; **MS** (ESI^+) *m/z* 715 $[M+H]^+$, 737 $[M+Na]^+$; **HRMS** (ESI^+) calcd. for $C_{40}H_{50}N_4NaO_8$ $[M+Na]^+$ 737.3521, found 737.3517; $[\alpha]_D^{27}$ –10.0 (*c* 0.10, $CHCl_3$).

Cbz-Leu-Ala-GOx-Pro-Tyr(Bn)-OBn (30)

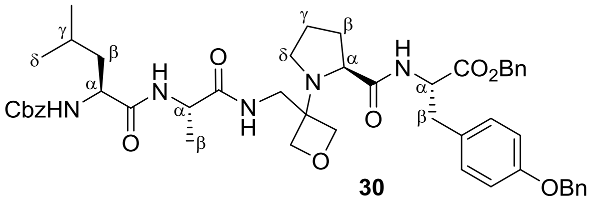

To a solution of Boc-Ala-GOx-Pro-Tyr(Bn)-OBn (**29**) (3.99 g, 5.58 mmol, 1.0 equiv) in CH_2Cl_2 (6.0 mL) was added TFA (6.0 mL) and the mixture was stirred at room temperature for 1 h *(Gas evolution!)*. The reaction mixture was concentrated under reduced pressure and the resulting residue repeatedly dissolved in CH_2Cl_2 (3 × 60 mL) and concentrated *in vacuo* to give the crude amine. The residue was dissolved in CH_2Cl_2 (60 mL), Cbz-Leu-OH (1.63 g, 6.14 mmol, 1.1 equiv), EDC·HCl (1.18 g, 6.14 mmol, 1.1 equiv), HOBt·H_2O (0.83 g, 6.14 mmol, 1.1 equiv) and NMM (2.45 mL, 22.3 mmol, 4.0 equiv) were added subsequently, and the mixture was stirred at room temperature for 24 h. The reaction mixture was diluted with EtOAc (50 mL) and washed with brine (3 × 50 mL), dried over Na_2SO_4, filtered and concentrated *in vacuo*. The residue was purified by column chromatography (SiO_2, CH_2Cl_2/MeOH 19:1) to give Cbz-Leu-Ala-GOx-Pro-Tyr(Bn)-OBn (**30**) (2.93 g, 3.40 mmol, 61%) as a white solid. **R_f** (CH_2Cl_2/MeOH 19:1) 0.32; **mp** 62–64 °C; **^{1}H NMR** (400 MHz, $CDCl_3$) δ_H

7.82 (d, J = 9.3 Hz, 1H, NH), 7.45–7.27 (m, 16H, ArH, NH), 7.04 (d, J = 8.5 Hz, 2H, ArH), 6.81 (d, J = 8.5 Hz, 2H, ArH), 6.66 (d, J = 7.0 Hz, 1H, NH), 5.30–5.18 (d, J = 12.2 Hz, 1H, C*H*HPh), 5.14–5.05 (m, 3H, CH*H*Ph, CH_2Ph), 5.03–4.91 (m, 4H, CH_2Ph, CHα-Tyr, NH), 4.62 (d, J = 6.8 Hz, 1H, OC*H*H-Ox), 4.36 (m, 4H, OCH*H*-Ox, OCH_2-Ox, CHα-Ala), 4.16 (m, 1H, CHα-Leu), 3.86 (m, 2H, C*H*HGOx, CHα-Pro), 3.59 (dd, J = 14.1, 4.2 Hz, 1H, CH*H*GOx), 3.17 (dd, J = 13.9, 5.0 Hz, 1H, C*H*Hβ-Tyr), 3.08–2.92 (m, 2H, CH*H*β-Tyr, C*H*Hδ-Pro), 2.43 (m, 1H, CH*H*δ-Pro), 2.09–1.91 (m, 1H, CH_2β-Pro or CH_2γ-Pro), 1.78–1.41 (m, 5H, CHγ-Leu, CH_2β-Leu, CH_2β-Pro or CH_2γ-Pro), 1.28 (d, J = 7.0 Hz, 3H, CH_3β-Ala), 0.92 (d, J = 5.6 Hz, 6H, 2 × CH_3δ-Leu); **^{13}C NMR** (126 MHz, $CDCl_3$) δ_C 175.1 (C=O), 173.3 (C=O), 173.0 (C=O), 172.2 (C=O), 157.9 (C), 156.3 (C=O, Cbz), 137.1 (C), 136.0 (C), 135.0 (C), 130.4 (CH), 128.84 (CH), 128.76 (CH), 128.72 (CH), 128.70 (CH), 128.5 (CH), 128.4 (CH), 128.3 (CH), 128.1 (CH), 127.6 (CH), 114.9 (CH), 78.6 (OCH_2), 76.5 (OCH_2), 70.0 (CH_2, Bn), 67.7 (CH_2, Bn), 67.4 (CH_2, Bn), 62.7 (C, Ox), 61.8 (CH, α-Pro), 53.8 (CH, α-Leu), 52.6 (CH, α-Tyr), 49.1 (CH, α-Ala), 48.8 (CH_2, δ-Pro), 45.2 (CH_2, GOx), 41.6 (CH_2, β-Leu), 36.9 (CH_2, β-Tyr), 31.6 (CH_2, β-Pro or CH_2, γ-Pro), 24.8 (CH, γ-Leu), 24.5 (CH_2, β-Pro or CH_2, γ-Pro), 23.1 (CH_3, δ-Leu), 21.9 (CH_3, δ-Leu), 17.6 (CH_3, β-Ala). *N.B.* One aromatic CH signal not observed; ν_{max} (neat) = 2955, 2879, 1720, 1649, 1509, 1347, 1119 cm^{-1}; **MS** (ESI$^-$) *m/z* 860 [M–H]$^-$; **HRMS** (ESI$^-$) calcd. for $C_{49}H_{58}N_5O_9$ [M–H]$^-$ 860.4240, found 860.4222; $[\alpha]_D^{27}$ –18.5 (*c* 0.10, $CHCl_3$).

H-Leu-Ala-GOx-Pro-Tyr-OH (1)

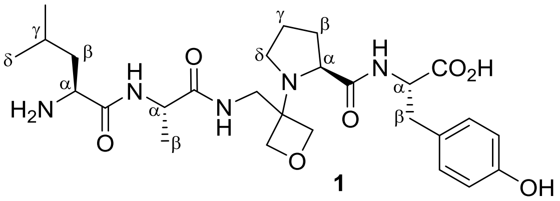

To a solution of pentapeptide Cbz-Leu-Ala-GOx-Pro-Tyr(Bn)-OBn (**30**) (0.48 g, 0.55 mmol, 1.0 equiv) in MeOH (6.0 mL) was added 10 wt% Pd/C (5.0 mg, 10 wt%) and the reaction flask was evacuated, filled with nitrogen, evacuated, and placed under an atmosphere of hydrogen (balloon). The reaction mixture was stirred at room temperature for 16 h, placed under nitrogen and filtered through a plug of Celite, which was washed with MeOH (3×). The filtrate was concentrated *in vacuo* to give H-Leu-Ala-GOx-Pro-Tyr-OH (**1**) as a white solid (30 mg, 0.55 mmol, quant. yield); **mp** 161–163 °C; **^{1}H NMR** (500 MHz, CD_3OD) δ_H 7.06 (d, J = 8.3 Hz, 2H, ArH), 6.70 (d, J = 8.3 Hz, 2H, ArH), 4.70 (d, J = 7.0 Hz, 1H, OC*H*H-Ox), 4.61 (dd, J = 8.0, 4.8 Hz, 1H, CHα-Tyr), 4.48–4.38 (m, 4H, OCH_2-Ox, OCH*H*-Ox, CHα-Ala), 3.95 (dd, J = 8.1, 6.2 Hz, 1H, CHα-Leu), 3.79 (d, J = 9.0 Hz, 1H, CHα-Pro), 3.70 (d, J = 14.2 Hz, 1H, C*H*HGOx), 3.60 (d, J = 14.2 Hz, 1H, CH*H*GOx), 3.18 (dd, J = 13.9, 4.7 Hz, 1H, C*H*Hβ-Tyr), 3.11 (t, J = 7.4 Hz, 1H, C*H*Hδ-Pro), 3.00 (dd, J = 13.9, 8.4 Hz, 1H, CH*H*β-Tyr), 2.64 (dt, J = 8.7, 5.9 Hz, 1H, CH*H*δ-Pro), 2.18–2.06 (m, 1H, C*H*Hβ-Pro or C*H*Hγ-Pro), 1.83–1.54 (m, 5H, CH_2β-Leu, CHγ-Leu, CH_2β-Pro or CH_2γ-Pro), 1.36 (d, J = 7.1 Hz, 3H, CH_3β-Ala), 0.99 (d, J = 4.0 Hz, 3H, CH_3δ-Leu), 0.98 (d, J = 4.0 Hz, 3H, CH_3δ-Leu); **^{13}C NMR** (126 MHz, CD_3OD) δ_C 177.5 (C=O), 175.9 (C=O), 175.4 (C=O), 170.3 (C=O), 157.4 (C), 131.5 (CH), 129.2 (C), 116.2 (CH), 79.0 (OCH_2), 77.6 (OCH_2), 64.4 (C, Ox), 62.9 (CH, α-Pro), 54.9 (CH, α-Tyr), 52.9 (CH, α-Leu), 50.9 (CH, α-Ala), 49.7 (CH_2, δ-Pro), 45.1 (CH_2, GOx), 41.6 (CH_2, β-Leu), 37.8 (CH_2, β-Tyr), 32.4 (CH_2, β-Pro or CH_2, γ-Pro), 25.4 (CH_2, β-Pro or CH_2, γ-Pro), 25.3 (CH, γ-Leu), 23.1 (CH_3, δ-Leu), 22.2 (CH_3, δ-Leu), 18.2 (CH_3, β-Ala); ν_{max} (neat) = 3267, 2989, 2900, 1644, 1513, 1234 cm^{-1}; **MS** (ESI$^-$) *m/z* 546 [M–H]$^-$; **HRMS** (ESI$^-$) calcd. for $C_{27}H_{40}N_5O_7$ [M–H]$^-$ 546.2933, found 546.2933; $[\alpha]_D^{27}$ –1.00 (*c* 0.20, MeOH).

Cyclo(Leu-Ala-GOx-Pro-Tyr) (4)

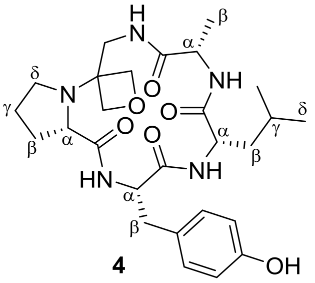

To a solution of pentapeptide H-Leu-Ala-GOx-Pro-Tyr-OH (**1**) (55 mg, 0.10 mmol, 1.0 equiv) in anhydrous DMF (100 mL, 0.001 M) under an atmosphere of nitrogen was added PyBOP (104 mg, 0.20 mmol, 2.0 equiv) and DIPEA (35 µL, 0.20 mmol, 2.0 equiv) and the reaction mixture was stirred for 48 h at room temperature. The solvent was removed under reduced pressure at 60 °C over 30 min, and the residue was dried under reduced pressure. The residue was analysed by LCMS and purified twice by column chromatography (SiO_2, CH_2Cl_2/MeOH 19:1→9:1) to give the cyclic pentapeptide (**4**) as a colourless glassy solid (32 mg, 60 µmol, 60%). $\mathbf{R_f}$ (CH_2Cl_2/MeOH 19:1) 0.15; **mp** 174–176 °C; **^{1}H NMR** (500 MHz, CD_3OD) δ_H 7.10 (d, *J* = 8.4 Hz, 2H, ArH), 6.73 (d, *J* = 8.4 Hz, 2H, ArH), 4.69 (d, *J* = 6.9 Hz, 1H, OCH*H*-Ox), 4.54–4.45 (m, 2H, CHα-Tyr, CHα-Ala), 4.43 (d, *J* = 7.5 Hz, 1H, OC*H*H-Ox), 4.35 (d, *J* = 6.8 Hz, 1H, OCH*H*-Ox), 4.27 (d, *J* = 7.5 Hz, 1H, OCH*H*-Ox), 4.13 (dd, *J* = 10.8, 4.9 Hz, 1H, CHα-Leu), 3.93 (dd, *J* = 9.8, 3.2 Hz, 1H, CHα-Pro), 3.79 (d, *J* = 14.0 Hz, 1H, C*H*HGOx), 3.58 (d, *J* = 14.0 Hz, 1H, CH*H*GOx), 3.28–3.21 (m, 1H, C*H*Hδ-Pro), 3.16–3.04 (m, 2H, CH_2β-Tyr), 2.58 (q, *J* = 8.3 Hz, 1H, CH*H*δ-Pro), 2.19–2.09 (m, 1H C*H*Hβ-Pro or C*H*Hγ-Pro), 1.94–1.80 (m, 3H, C*H*Hβ-Leu, CH_2β-Pro or CH_2γ-Pro), 1.75–1.68 (m, 1H, CH*H*β-Pro or CH*H*γ-Pro), 1.63–1.55 (m, 1H, CH*H*β-Leu), 1.49–1.40 (m, 4H, CHγ-Leu, CH_3β-Ala), 0.94 (d, *J* = 6.6 Hz, 3H, CH_3δ-Leu), 0.85 (d, *J* = 6.5 Hz, 3H, CH_3δ-Leu); **^{13}C NMR** (126 MHz, CD_3OD) δ_C 178.4 (C=O), 176.0 (C=O), 174.3 (C=O), 173.8 (C=O), 157.4 (C), 131.3 (CH), 128.8 (C), 116.3 (CH), 78.8 (OCH_2), 77.9 (OCH_2), 63.8 (C, Ox), 62.9 (CH, α-Pro), 57.0 (CH, α-Tyr), 56.1 (CH, α-Leu), 51.5 (CH, α-Ala), 49.3 (CH_2, δ-Pro), 46.2 (CH_2, GOx), 40.6 (CH_2, β-Leu), 36.5 (CH_2, β-Tyr), 32.7 (CH_2, β-Pro or CH_2, γ-Pro), 25.9 (CH_2, β-Pro or CH_2, γ-Pro), 25.7 (CH, γ-Leu), 23.4 (CH_3, δ-Leu), 21.5 (CH_3, δ-Leu), 18.5 (CH_3, β-Ala); $\mathbf{\nu_{max}}$ (neat) = 3271, 2956, 2872, 1642, 1512, 1241 cm^{-1}; **MS** (ESI^+) *m/z* 552 $[M+Na]^+$; **HRMS** (ESI^+) calcd. for $C_{27}H_{39}N_5NaO_6$ $[M+Na]^+$ 552.2793, found 552.2796; $[\boldsymbol{\alpha}]_D^{27}$ –76.5 (*c* 0.10, MeOH).

*2.2 Preparation of cyclic pentapeptide **5***

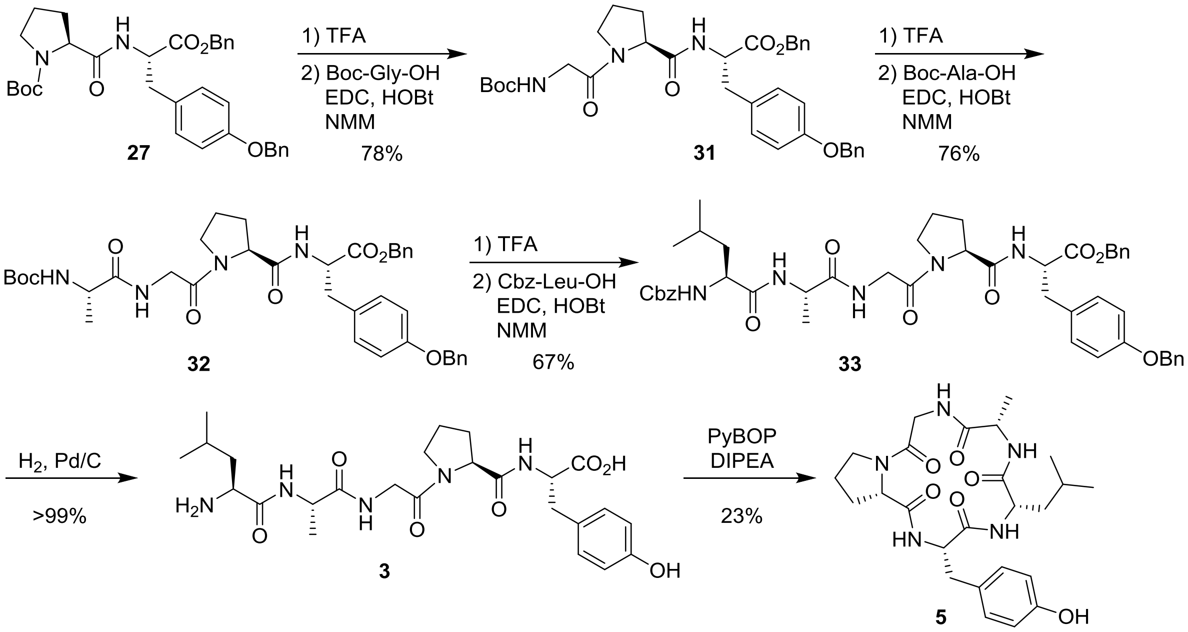

Boc-Gly-Pro-Tyr(Bn)-OBn (31)

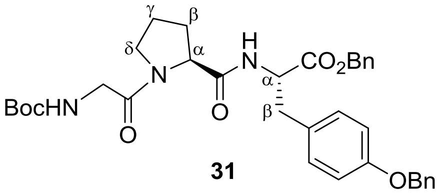

31

To a solution of dipeptide **27** (5.06 g, 9.08 mmol, 1.0 equiv) in CH_2Cl_2 (9.0 mL) was added TFA (9.0 mL) and the mixture was stirred at room temperature for 1 h *(Caution – gas evolution!)*. The reaction mixture was concentrated under reduced pressure and the resulting residue repeatedly dissolved in CH_2Cl_2 (3 × 100 mL) and concentrated under reduced pressure to give the crude amine. The residue was dissolved in CH_2Cl_2 (90 mL), Boc-Gly-OH (1.75 g, 9.98 mmol, 1.1 equiv), EDC·HCl (1.91 g, 9.98 mmol, 1.1 equiv), HOBt·H_2O (1.35 g, 9.98 mmol, 1.1 equiv) and NMM (4.39 mL, 39.9 mmol, 4.0 equiv) were added subsequently, and the mixture was stirred at room temperature for 24 h. The reaction mixture was diluted with EtOAc (100 mL) and washed with brine (3 × 100 mL), dried over Na_2SO_4, filtered and concentrated under reduced pressure. The residue was purified by column chromatography (SiO_2, PE/EtOAc 1:1) to give tripeptide Boc-Gly-Pro-Tyr(Bn)-OBn (**31**) (4.36 g, 7.09 mmol, 78%) as a white solid. **R_f** (PE/EtOAc 1:1) 0.23; **mp** 52–55 °C; **^{1}H NMR** (500 MHz, $CDCl_3$) δ_H 7.46–7.29 (m, 10H, ArH), 7.23 (d, *J* = 7.8 Hz, 1H, NH), 6.94 (d, *J* = 8.4 Hz, 2H, ArH), 6.81 (d, *J* = 8.4 Hz, 2H, ArH), 5.39 (s, 1H, NH), 5.15 (d, *J* = 12.2 Hz, 2H, CH_2Ph), 5.02 (s, 2H, CH_2Ph), 4.82 (dd, *J* = 13.4, 7.0 Hz, 1H, CHα-Tyr), 4.54 (d, *J* = 7.0 Hz, 1H, CHα-Pro), 3.94 (dd, *J* = 17.4, 4.9 Hz, 1H, C*H*HGly), 3.72 (dd, *J* = 17.4, 3.8 Hz, 1H, CH*H*Gly), 3.26 (t, *J* = 6.9 Hz, 2H, CH_2δ-Pro), 3.11 (dd, *J* = 14.1, 5.6 Hz, 1H, C*H*Hβ-Tyr), 2.94 (dd, *J* = 14.1, 7.0 Hz, 1H, CH*H*β-Tyr), 2.35–2.29 (m, 1H, C*H*Hβ-Pro or C*H*Hγ-Pro), 1.96–1.89 (m, 2H, CH_2β-Pro or CH_2γ-Pro), 1.82–1.73 (m, 1H, CH*H*β-Pro or CH*H*γ-Pro), 1.45 (s, 9H, 3 × CH_3, Boc); **^{13}C NMR** (126 MHz, $CDCl_3$) δ_C 171.4 (C=O), 170.4 (C=O), 168.7 (C=O), 157.9 (C), 155.9 (C=O, Boc), 137.2 (C), 135.4 (C), 130.5 (CH), 128.8 (CH), 128.7 (CH), 128.6 (CH), 128.3 (C), 128.1 (CH), 127.7 (CH), 127.6 (CH), 114.8 (CH), 79.9 (C, Boc), 70.0 (CH_2Ph), 67.3 (CH_2Ph), 60.0 (CH, α-Pro), 53.5 (CH, α-Tyr), 46.2 (CH_2, δ-Pro), 43.2 (CH_2, Gly), 37.1 (CH_2, β-Tyr), 28.5 (CH_3, Boc), 27.1 (CH_2, β-Pro or CH_2, γ-Pro), 24.9 (CH_2, β-Pro or CH_2, γ-Pro); **ν_{max}** (neat) = 3307, 2974, 1739, 1709, 1646, 1509, 1238, 1161, 695 cm^{-1}; **MS** (ESI^+) *m/z* 638 $[M+Na]^+$; **HRMS** (ESI^+) calcd. for $C_{35}H_{41}N_3NaO_7$ $[M+Na]^+$ 638.2837, found 638.2833; $[\boldsymbol{\alpha}]_D^{22}$ –66.0 (*c* 0.27, $CHCl_3$).

Boc-Ala-Gly-Pro-Tyr(Bn)-OBn (32)

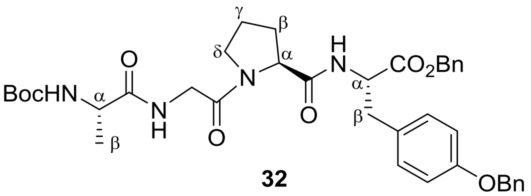

32

To a solution of tripeptide **31** (4.21 g, 6.84 mmol, 1.0 equiv) in CH_2Cl_2 (7.0 mL) was added TFA (7.0 mL) and the mixture was stirred at room temperature for 1 h *(Gas evolution!)*. The reaction mixture was concentrated under reduced pressure and the resulting residue repeatedly dissolved in CH_2Cl_2 (3 × 70 mL) and concentrated under reduced pressure to give the crude amine. The residue was dissolved in CH_2Cl_2 (70 mL), Boc-Ala-OH (1.42 g, 7.52 mmol, 1.1 equiv), EDC·HCl (1.44 g, 7.52 mmol, 1.1 equiv), HOBt·H_2O (1.02 g, 7.52 mmol, 1.1 equiv) and NMM (3.0 mL, 27.4 mmol, 4.0 equiv) were added subsequently, and the mixture was stirred at room temperature for 24 h. The reaction mixture was diluted with EtOAc (70 mL) and washed with brine (3 × 70 mL), dried over Na_2SO_4, filtered and concentrated *in vacuo*. The residue was purified by column chromatography (SiO_2, EtOAc/PE 8:2) to give tetrapeptide Boc-Ala-Gly-Pro-Tyr(Bn)-OBn (**32**) (3.58 g, 4.71 mmol, 76%) as a white solid. **R_f** (EtOAc/PE 8:2) 0.26; **mp** 62–64 °C; **^{1}H NMR** (500 MHz, $CDCl_3$) δ_H 7.44–7.30 (m, 11H, ArH, NH), 7.11 (d, *J* = 7.2 Hz, 1H, NH), 6.94 (d, *J* = 8.5 Hz, 2H, ArH), 6.81 (d, *J* = 8.5 Hz, 2H, ArH), 5.22–5.09 (m, 3H, CH_2Ph, NH), 5.04–4.99 (m, 2H, CH_2Ph), 4.84 (dd, *J* = 13.6, 7.1 Hz, 1H, CHα-Tyr), 4.52 (d, *J* = 6.8 Hz, 1H, CHα-Pro), 4.28–4.19 (m, 1H, CHα-Ala), 3.98 (dd, *J* = 17.6, 4.3 Hz, 1H, C*H*HGly), 3.86 (dd, *J* = 17.6, 3.9 Hz, 1H, CH*H*Gly), 3.34–3.24 (m, 2H, CH_2δ-Pro), 3.11 (dd, *J* = 14.1, 5.7 Hz, 1H, C*H*Hβ-Tyr), 2.96 (dd, *J* = 14.1,

7.0 Hz, 1H, CH*H*β-Tyr), 2.31–2.25 (m, 1H, C*H*Hβ-Pro or C*H*Hγ-Pro), 1.96–1.88 (m, 2H, CH_2β-Pro or CH_2γ-Pro), 1.85–1.76 (m, 1H, CH*H*β-Pro or CH*H*γ-Pro), 1.44 (s, 9H, 3 × CH_3, Boc), 1.33 (d, *J* = 7.1 Hz, 3H, CH_3β-Ala); **^{13}C NMR** (126 MHz, $CDCl_3$) δ_C 172.9 (C=O), 171.5 (C=O), 170.3 (C=O), 167.9 (C=O), 157.8 (C), 155.5 (C=O, Boc), 137.1 (C), 135.4 (C), 130.5 (CH), 128.75 (CH), 128.73 (CH), 128.70 (CH), 128.66 (CH), 128.64 (CH), 128.3 (C), 128.1 (CH), 127.7 (CH), 114.9 (CH), 80.2 (C, Boc), 70.1 (CH_2Ph), 67.3 (CH_2Ph), 60.1 (CH, α-Pro), 53.3 (CH, α-Tyr), 50.3 (CH, α-Ala), 46.4 (CH_2, δ-Pro), 42.2 (CH_2, Gly), 37.0 (CH_2, β-Tyr), 28.5 (CH_3, Boc), 27.5 (CH_2, β-Pro or CH_2, γ-Pro), 24.8 (CH_2, β-Pro or CH_2, γ-Pro), 18.7 (CH_3, β-Ala); **ν_{max}** (neat) = 3293, 2974, 1739, 1639, 1509, 1239, 1163, 1023, 696 cm^{-1}; **MS** (ESI^+) *m/z* 709 $[M+Na]^+$; **HRMS** (ESI^+) calcd. for $C_{38}H_{46}N_4NaO_8$ $[M+Na]^+$ 709.3208, found 709.3206; $[\alpha]_D^{22}$ –46.1 (*c* 0.27, $CHCl_3$).

Cbz-Leu-Ala-Gly-Pro-Tyr(Bn)-OBn (33)

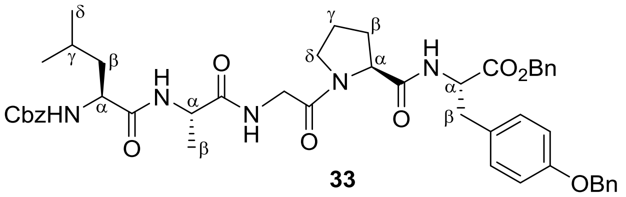

To a solution of tetrapeptide **32** (3.23 g, 4.71 mmol, 1.0 equiv) in CH_2Cl_2 (5.0 mL) was added TFA (5.0 mL) and the mixture was stirred at room temperature for 1 h *(Gas evolution!)*. The reaction mixture was concentrated under reduced pressure and the resulting residue repeatedly dissolved in CH_2Cl_2 (3 × 50 mL) and concentrated *in vacuo* to give the crude amine. The residue was dissolved in CH_2Cl_2 (47 mL), Cbz-Leu-OH (1.38 g, 5.18 mmol, 1.1 equiv), EDC·HCl (0.99 g, 5.18 mmol, 1.1 equiv), HOBt·H_2O (0.70 g, 5.18 mmol, 1.1 equiv) and NMM (2.07 mL, 18.9 mmol, 4.0 equiv) were added subsequently, and the mixture was stirred at room temperature for 24 h. The reaction mixture was diluted with EtOAc (50 mL) and washed with brine (3 × 50 mL), dried over Na_2SO_4, filtered and concentrated under reduced pressure. The residue was purified by column chromatography (SiO_2, CH_2Cl_2/MeOH 97:3) to give pentapeptide Cbz-Leu-Ala-Gly-Pro-Tyr(Bn)-OBn (**33**) (2.63 g, 3.16 mmol, 67%) as a white solid. **R_f** (CH_2Cl_2/MeOH 97:3) 0.18; **mp** 69–72 °C; NMR data reported for the major rotamer: **^{1}H NMR** (600 MHz, DMSO-*d6* @ 373 K) δ_H 7.65 (d, *J* = 7.2 Hz, 1H, NH), 7.57 (s, 1H, NH), 7.46–7.25 (m, 15H, ArH), 7.11 (d, *J* = 8.4 Hz, 2H, ArH), 6.90 (d, *J* = 8.4 Hz, 2H, ArH), 5.12–5.04 (m, 6H, 3 × CH_2Ph), 4.61–4.53 (m, 1H, CHα-Tyr), 4.42 (dd, *J* = 8.5, 2.6 Hz, 1H, CHα-Pro), 4.40–4.35 (m, 1H, CHα-Ala), 4.08 (td, *J* = 8.9, 5.3 Hz, 1H, CHα-Leu), 3.85 (br. s, 2H, CH_2Gly), 3.44 (m, 2H, CH_2δ-Pro), 3.08–3.00 (m, 2H, CH_2β-Tyr), 1.86–1.47 (m, 7H, CH_2β-Leu, CHγ-Leu, CH_2β-Pro, CH_2γ-Pro), 1.26 (d, *J* = 7.0 Hz, 3H, CH_3β-Ala), 0.89 (d, *J* = 6.6 Hz, 3H, CH_3δ-Leu), 0.87 (d, *J* = 6.6 Hz, 3H, CH_3δ-Leu). *N.B.* Two NH signals not observed; **^{13}C NMR** (151 MHz, DMSO-*d6 @* 373 K) δ_C 171.5 (C=O), 171.3 (C=O), 170.8 (C=O), 170.5 (C=O), 166.6 (C=O), 156.9 (C=O, Cbz), 155.2 (C),136.9 (C), 136.6 (C), 135.3 (C), 129.5 (CH), 128.9 (C), 127.6 (CH), 127.7 (CH), 127.4 (CH), 127.3 (CH), 127.1 (CH), 126.9 (CH), 126.8 (CH), 114.5 (CH), 69.2 (CH_2Ph), 65.6 (CH_2Ph), 65.1 (CH_2Ph), 58.9 (CH, α-Pro), 53.2 (CH α-Leu or CH, α-Tyr), 53.1 (CH α-Leu or CH, α-Tyr), 47.8 (CH, α-Ala), 45.3 (CH_2, δ-Pro), 42.2 (CH_2, Gly), 40.9 (CH_2, β-Leu), 35.6 (CH_2, β-Tyr), 32.5 (CH_2, β-Pro or CH_2, γ-Pro), 28.5 (CH_2, β-Pro or CH_2, γ-Pro), 23.8 (CH, γ-Leu), 22.3 (CH_3, δ-Leu), 21.1 (CH_3, δ-Leu), 17.6 (CH_3, β-Ala). *N.B.* CH_2, β-Tyr, β-Pro and γ-Pro peaks assigned by HSQC correlations; two aromatic CH signals not observed; **ν_{max}** (neat) = 3285, 3064, 2955, 1717, 1268, 1509, 1174, 695 cm^{-1}; **MS** (ESI^+) *m/z* 856 $[M+Na]^+$; **HRMS** (ESI^+) calcd. for $C_{47}H_{55}N_5NaO_9$ $[M+Na]^+$ 856.3892, found 856.3903; $[\alpha]_D^{22}$ –52.6 (*c* 0.30, $CHCl_3$).

H-Leu-Ala-Gly-Pro-Tyr-OH (3)

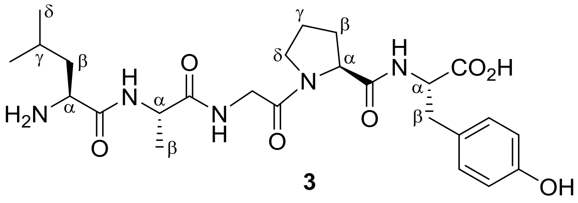

3

To a solution of pentapeptide **33** (1.00 g, 1.19 mmol, 1.0 equiv) in MeOH (120 ml) was added 10 wt% Pd/C (100 mg, 10 wt%) and the reaction flask was evacuated, filled with nitrogen, evacuated, and placed under an atmosphere of hydrogen (balloon). The reaction mixture was stirred at room temperature for 16 h, placed under nitrogen and filtered through a plug of Celite, which was washed with MeOH (3×). The filtrate was concentrated *in vacuo* to give H-Leu-Ala-Gly-Pro-Tyr-OH (**3**) as a pale pink solid (622 mg, 1.19 mmol, quant. yield). **mp** 172–175 °C; **^{1}H NMR** (600 MHz, DMSO-*d6* @ 373 K) δ_H 7.02 (d, *J* = 8.2 Hz, 2H, ArH), 6.70 (d, *J* = 8.1 Hz, 2H, ArH), 4.51–4.35 (m, 4H, CH α-Tyr, CHα-Pro, CHα-Ala, C*H*Hα-Gly), 3.87 (dd, *J* = 16.0, 4.3 Hz, 1H, CH*H*α-Gly), 3.77 (t, *J* = 6.9 Hz, 1H, CHα-Leu), 3.52–3.41 (m, 2H, CH_2δ-Pro), 2.99 (dd, *J* = 13.0, 3.8 Hz, 1H, C*H*Hβ-Tyr), 2.91–2.83 (m, 1H, CH*H*β-Tyr), 1.96–1.73 (m, 5H, CHγ-Leu, CH_2β-Pro, CH_2γ-Pro), 1.72–1.64 (m, 1H, C*H*Hβ-Leu), 1.64–1.56 (m, 1H, CH*H*β-Leu), 1.34 (d, *J* = 7.0 Hz, 3H, CH_3β-Ala), 0.96–0.90 (d, *J* = 6.6 Hz, 6H, 2 × δ-CH_3 Leu); **^{13}C NMR** (151 MHz, DMSO-*d6* @ 373 K) δ_C 171.8 (C=O), 171.0 (C=O), 170.5 (C=O), 168.0 (C=O), 166.6 (C=O), 155.5 (C), 129.3 (CH), 127.0 (C), 114.7 (CH), 58.9 (CH, α-Pro), 53.2 (CH, α-Tyr), 51.0 (CH, α-Leu), 48.2 (CH, α-Ala), 45.6 (CH_2, δ-Pro) 40.9 (CH_2, Gly), 39.6 (CH_2, β-Leu), 35.7 (CH_2, β-Tyr), 23.1 (CH, γ-Leu), 21.9 (CH_3, δ-Leu), 21.5 (CH_3, δ-Leu), 17.3 (CH_3, β-Ala). *N.B.* β-CH_2, Pro and γ-CH_2, Pro signals are not observed; **ν_{max}** (neat) = 3217, 2961, 1639, 1513, 1445, 1336, 1224 cm^{-1}; **MS** (ESI$^+$) *m/z* 520 $[M+H]^+$, 542 $[M+Na]^+$; **HRMS** (ESI$^+$) calcd. for $C_{25}H_{38}N_5O_7$ $[M+H]^+$ 520.2766, found 520.2768; $[\alpha]_D^{22}$ –49.5 (*c* 0.09, MeOH).

Cyclo(Leu-Ala-Gly-Pro-Tyr) (5)

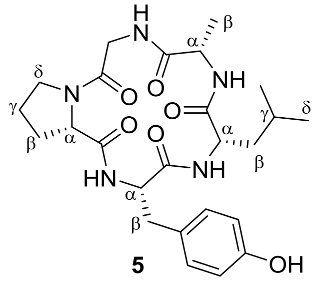

5

To a solution of H-Leu-Ala-Gly-Pro-Tyr-OH (**3**) (52 mg, 0.10 mmol, 1.0 equiv) in anhydrous DMF (100 mL, 0.001 M) under an atmosphere of nitrogen was added PyBOP (104 mg, 0.20 mmol, 2.0 equiv) and DIPEA (35 μL, 0.20 mmol, 2.0 equiv) and the reaction mixture was stirred for 48 h at room temperature. The solvent was removed under reduced pressure at 60 °C over 30 min, and the residue was dried under reduced pressure. The residue was analysed by LCMS and purified twice by column chromatography (SiO_2, CH_2Cl_2/MeOH 9:1→4:1) to give the cyclic pentapeptide (**5**) as a white solid (12 mg, 23 μmol, 23%). **R_f** (CH_2Cl_2/MeOH 9:1) 0.28; **mp** 178–181 °C; **^{1}H NMR** (500 MHz, CD_3OD) δ_H 7.09 (d, *J* = 8.4 Hz, 2H, ArH), 6.72 (d, *J* = 8.4 Hz, 2H, ArH), 4.65 (t, *J* = 8.1 Hz, 1H, CHα-Tyr), 4.46 (q, *J* = 7.0 Hz, 1H, CHα-Ala), 4.30 (dt, *J* = 8.7, 4.3 Hz, 1H, CHα-Pro), 4.16 (d, *J* = 14.6 Hz, 1H, C*H*HGly), 4.09–4.03 (m, 1H, C*H*Hδ-Pro), 3.90 (dd, *J* = 10.2, 5.9 Hz, 1H, CHα-Leu), 3.69–3.62 (m, 1H, C*H*Hδ-Pro), 3.57 (d, *J* = 14.6 Hz, 1H, CH*H*Gly), 3.07–2.97 (m, 2H, CH_2β-Tyr), 2.22–2.13 (m, 1H, C*H*Hγ-Pro), 2.02–1.88 (m, 3H, C*H*Hβ-Leu, CH_2β-Pro), 1.79–1.71 (m, 1H, CH*H*γ-Pro), 1.68–1.64 (m, 1H, CHγ-Leu), 1.54–1.51 (m, 1H, C*H*Hβ-Leu), 1.37 (d, *J* = 7.1 Hz, 3H, CH_3β-Ala), 0.92 (d, *J* = 6.6 Hz, 3H, CH_3δ-Leu), 0.83 (d, *J* = 6.5 Hz, 3H, CH_3δ-Leu); **^{13}C NMR** (126 MHz, CD_3OD) δ_C 176.1 (C=O), 175.0 (C=O), 174.1 (C=O), 173.4 (C=O), 170.7 (C=O), 157.4 (C), 131.3 (CH), 128.8 (C), 116.2 (CH), 62.9 (CH, α-Pro), 57.5 (CH, α-Leu), 56.5 (CH, α-Tyr), 50.1 (CH, α-Ala), 48.5 (CH_2, δ-Pro), 43.0 (CH_2, Gly), 39.7 (CH_2, β-Leu), 37.3 (CH_2, β-Tyr), 30.7 (CH_2, β-Pro or CH_2, γ-Pro), 25.8 (CH, γ-Leu), 25.4 (CH_2, β-Pro or CH_2, γ-Pro), 23.3 (CH_3, δ-Leu), 21.7 (CH_3 δ-Leu), 18.3 (CH_3, β-Ala); **ν_{max}** (neat) = 3268, 2956, 2930, 1641, 1514, 1233 cm^{-1}; **MS** (ESI$^+$) *m/z* 524 $[M+Na]^+$; **HRMS** (ESI$^+$) calcd. for $C_{25}H_{35}N_5NaO_6$ $[M+Na]^+$ 524.2480, found 524.2479; $[\alpha]_D^{22}$ –88.1 (*c* 0.08, MeOH).

*2.3 Preparation of cyclic tetrapeptides **9**, **10** and **11***

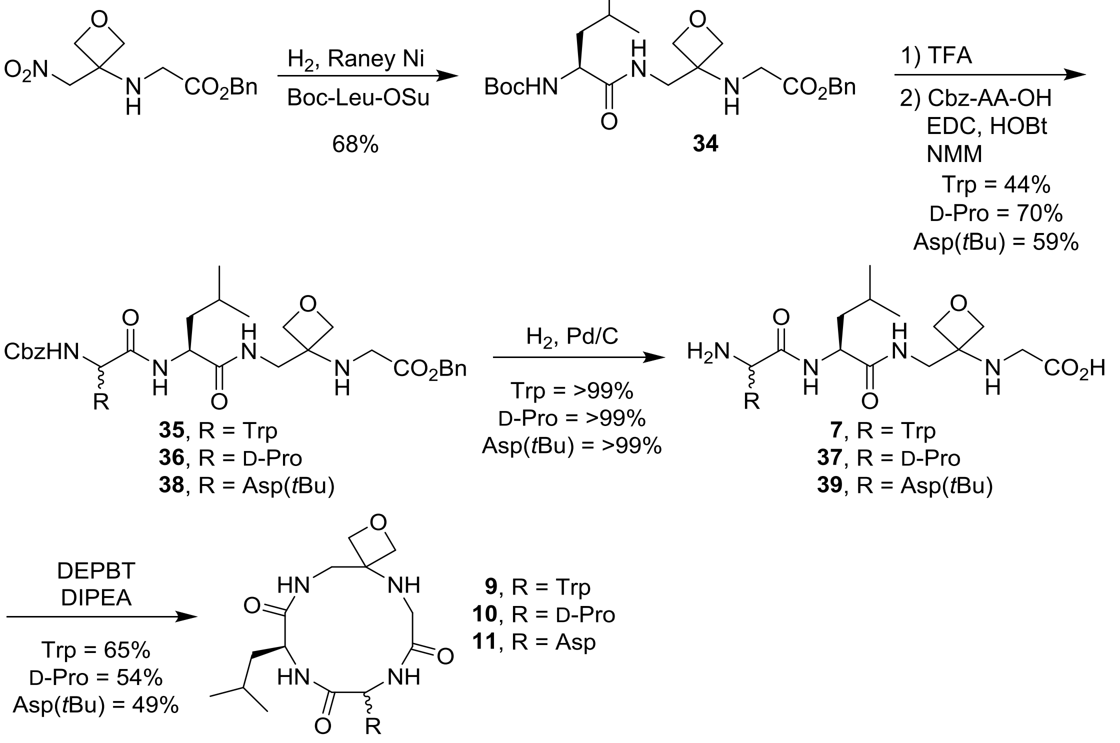

Boc-Leu-GOx-Gly-OBn (34)

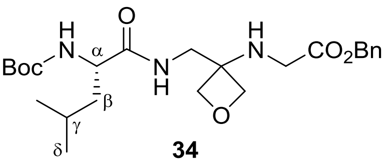

To a solution of NO_2-GOx-Gly-OBn[2] (1.70 g, 6.06 mmol, 1.0 equiv) in THF (60 mL) was added Boc-Leu-OSu (2.98 g, 9.09 mmol, 1.5 equiv) and Raney Ni (slurry in H_2O, 6.0 mL). The solution was placed under an atmosphere of nitrogen, evacuated and filled with hydrogen (balloon). The reaction mixture was stirred vigorously for 4.0 h at room temperature. Then, the mixture was filtered through a plug of Celite eluting with EtOAc, concentrated under reduced pressure, the filtrate was suspended in EtOAc (50 mL), washed with saturated Na_2CO_3 (3 × 50 mL), dried over Na_2SO_4 and concentrated *in vavuo*. Boc-Leu-GOx-Gly-OBn (**34**) was afforded after purification by column chromatography (SiO_2, EtOAc/PE 3:2) as a colourless viscous oil (1.91 g, 4.13 mmol, 68%). **R_f** (EtOAc/PE 3:2) 0.28; **^{1}H NMR** (500 MHz, $CDCl_3$) δ_H 7.40–7.32 (m, 5H, ArH), 6.73 (br. t, J = 5.0 Hz, 1H, NH), 5.17 (s, 2H, CH_2Ph), 4.93 (br. s, 1H, NH), 4.44 (m, 2H, OCH_2-Ox), 4.36 (d, J = 7.0 Hz, 2H, OCH_2-Ox), 4.07 (br. m, 1H, CHα-Leu), 3.65–3.57 (m, 2H, CH_2GOx), 3.54–3.48 (m, 2H, CH_2Gly), 2.00 (br. s, 1H, NH), 1.70–1.61 (m, 2H, C*H*Hβ-Leu, CHγ-Leu), 1.48–1.44 (1H, m, CH*H*β-Leu), 1.42 (s, 9H, 3 × CH_3, Boc), 0.94 (d, J = 1.7 Hz, 3H, CH_3δ-Leu), 0.93 (d, J = 1.7 Hz, 3H, CH_3δ-Leu); **^{13}C NMR** (125 MHz, $CDCl_3$) δ_C 173.4 (C=O), 172.5 (C=O), 155.7 (C=O, Boc), 135.2 (C), 128.7 (CH), 128.6 (CH), 128.5 (CH), 79.3 (2 × OCH_2), 67.1 (CH_2, Bn), 59.6 (C, Ox), 53.3 (CH, α-Leu), 44.0 (CH_2, Gly), 43.2 (CH_2, GOx), 41.3 (CH_2, β-Leu), 28.3 (CH_3, Boc), 24.9 (CH, γ-Leu), 20.8 (2 x CH_3, δ-Leu); **ν_{max}** (neat) = 2956, 2871, 1739, 1656, 1520, 1164 cm^{-1}; **MS** (ESI$^+$) *m/z* 464 $[M+H]^+$, 486 $[M+Na]^+$; **HRMS** (ESI$^+$) calcd. for $C_{24}H_{37}N_3NaO_6$ $[M+Na]^+$ 486.2575, found 486.2572; $[\alpha]_D^{26}$ +4.4 (c 0.14, $CHCl_3$).

Cbz-Trp-Leu-GOx-Gly-OBn (35)

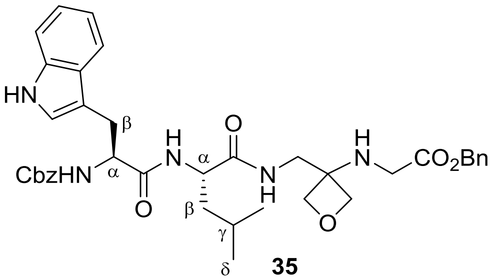

35

To a solution of tripeptide Boc-Leu-GOx-Gly-OBn **34** (1.29 g, 2.79 mmol, 1.0 equiv) in CH_2Cl_2 (3.0 mL) was added TFA (3.0 mL) and the mixture was stirred at room temperature for 1 h *(Caution – gas evolution!)*. The reaction mixture was concentrated under reduced pressure and the resulting residue repeatedly dissolved in CH_2Cl_2 (3 × 25 mL) and concentrated under reduced pressure to give the crude amine. The residue was dissolved in a mixture of CH_2Cl_2 (30 mL), Cbz-Trp-OH (0.94 g, 2.79 mmol, 1.0 equiv), EDC·HCl (0.53 g, 2.79 mmol, 1.0 equiv), HOBt·H_2O (0.38 g, 2.79 mmol, 1.0 equiv) and NMM (1.23 mL, 11.2 mmol, 4.0 equiv) were added subsequently, and the reaction mixture was stirred at room temperature for 24 h. The reaction mixture was diluted with EtOAc (30 mL) and washed with brine (30 mL), dried over Na_2SO_4, filtered and concentrated under reduced pressure. The residue was purified by column chromatography (SiO_2, 49:1 CH_2Cl_2/MeOH) to give tetrapeptide Cbz-Trp-Leu-GOx-Gly-OBn (**35**) (834 mg, 1.22 mmol, 44%) as a colourless viscous oil. $\mathbf{R_f}$ (CH_2Cl_2/MeOH 49:1) 0.31; **^{1}H NMR** (500 MHz, $CDCl_3$) δ_H ppm 8.45 (s, 1H, NH), 7.62 (d, *J* = 7.3 Hz, 1H, ArH), 7.41–7.27 (m, 11H, ArH), 7.17 (t, *J* = 7.5 Hz, 1H, ArH), 7.08 (t, *J* = 7.1 Hz, 1H, ArH), 7.02 (s, 1H, ArH), 6.76 (t, *J* = 5.5 Hz, 1H, NH), 6.39 (d, *J* = 6.9 Hz, 1H, NH), 5.59 (d, *J* = 6.1 Hz, 1H, NH), 5.13 (s, 2H, CH_2Ph), 5.06 (s, 2H, CH_2Ph), 4.53 (q, *J* = 6.5 Hz, 1H, CHα-Trp), 4.40–4.32 (m, 3H, CHα-Leu, OCH_2-Ox), 4.31–4.25 (m, 2H, OCH_2-Ox), 3.54–3.41 (m, 4H, CH_2Gly, CH_2GOx), 3.30–3.16 (m, 2H, CH_2β-Trp), 1.64–1.52 (m, 1H, C*H*Hβ-Leu), 1.47–1.29 (m, 2H, CH*H*β-Leu, CHγ-Leu), 0.82 (d, *J* = 6.3 Hz, 3H, CH_3δ-Leu), 0.81 (d, *J* = 6.3 Hz, 3H, CH_3δ-Leu); **^{13}C NMR** (126 MHz, $CDCl_3$) δ_C ppm 172.7 (C=O), 172.5 (C=O), 171.5 (C=O), 156.3 (C=O, Cbz), 136.3 (C), 136.1 (C), 135.3 (C), 128.7 (CH), 128.6 (CH), 128.5 (CH), 128.3 (CH), 128.1 (CH), 127.3 (C), 123.5 (CH), 122.3 (CH), 119.8 (CH), 118.8 (CH), 111.4 (CH), 110.0 (C), 79.1 (OCH_2), 79.0 (OCH_2), 67.2 (CH_2, Bn), 67.1 (CH_2, Bn), 59.6 (C, Ox), 55.6 (CH, α-Trp), 52.2 (CH, α-Leu), 44.7 (CH_2, GOx or CH_2, Gly), 43.4 (CH_2, GOx or CH_2, Gly), 40.7 (CH_2, β-Leu), 28.1 (CH_2, β-Trp), 24.7 (CH, γ-Leu), 22.8 (CH_3, δ-Leu), 21.9 (CH_3, δ-Leu); $\mathbf{\nu_{max}}$ (neat) = 3316, 2952, 1707, 1645, 1510, 1173, 738 cm^{-1}; **MS** (ESI^+) *m/z* 684 $[M+H]^+$, 706 $[M+Na]^+$; **HRMS** (ESI^+) calcd. for $C_{38}H_{45}N_5NaO_7$ $[M+Na]^+$ 706.3211, found 706.3211; $[\boldsymbol{\alpha}]_D^{26}$ –23.6 (*c* 0.14, $CHCl_3$).

H-Trp-Leu-GOx-Gly-OH (7)

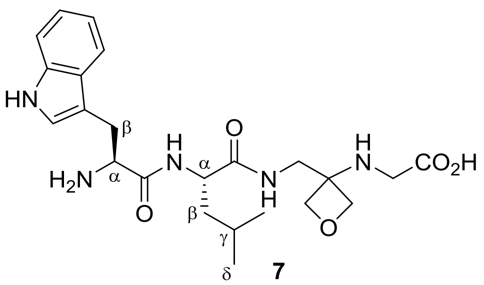

7

To a solution of tetrapeptide Cbz-Trp-Leu-GOx-Gly-OBn (**35**) (683 mg, 1.00 mmol, 1.0 equiv) in MeOH (10 mL) was added 10 wt% Pd/C (68 mg, 10 wt%) and the reaction flask was evacuated, filled with nitrogen, evacuated, and placed under an atmosphere of hydrogen (balloon). The reaction mixture was stirred at room temperature for 16 h, placed under nitrogen and filtered through a plug of Celite, which was washed with MeOH (3×). The filtrate was concentrated *in vacuo* to give tetrapeptide H-Trp-Leu-GOx-Gly-OH (**7**) as a yellow solid (458 mg, 1.00 mmol) in quantitative yield; **mp** 260–263 °C; **^{1}H NMR** (500 MHz, D_2O) δ_H 7.64 (d, *J* = 7.9 Hz, 1H, ArH), 7.53 (d, *J* = 8.2 Hz, 1H, ArH), 7.32 (s, 1H, ArH), 7.28 (t, *J* = 7.6 Hz, 1H, ArH), 7.18 (t, *J* = 7.5 Hz, 1H, ArH), 4.72–4.67 (m, 2H, OCH_2-Ox), 4.56 (d, *J* = 8.2 Hz, 1H, OC*H*H-Ox), 4.52 (d, *J* = 8.2 Hz, 1H, OCH*H*-Ox), 4.32 (t, *J* = 7.3 Hz, 1H, CHα-Trp), 4.27 (dd, *J* = 9.0, 5.8 Hz, 1H, CHα-Leu), 3.63–3.53 (m, 4H, CH_2Gly, CH_2GOx), 3.43 (dd, *J* = 14.7, 7.9 Hz, 1H, C*H*Hβ-Trp), 3.36 (dd, *J* = 15.0, 6.5 Hz, 1H, CH*H*β-Trp), 1.61–1.34 (m, 3H, CH_2β-Leu, CHγ-Leu), 0.87 (d, *J* = 6.4 Hz, 3H, CH_3, δ-Leu), 0.83 (d, *J* = 6.4 Hz,

3H, CH_3, δ-Leu); **^{13}C NMR** (126 MHz, D_2O) δ_C 174.5 (C=O), 169.3 (C=O), 136.2 (C), 126.5 (C), 125.2 (CH), 122.1 (CH), 119.5 (CH), 118.0 (CH), 112.0 (CH), 106.3 (C), 75.9 (2 × OCH_2), 60.6 (C, Ox), 53.5 (CH, α-Trp), 52.6 (CH, α-Leu), 45.1 (CH_2, GOx or CH_2, Gly), 40.6 (CH_2, GOx or CH_2, Gly), 40.0 (CH_2, β-Leu), 26.7 (CH_2, β-Trp), 24.1 (CH, γ-Leu), 21.9 (CH_3, δ-Leu), 20.8 (CH_3, δ-Leu). *N.B.* One carbonyl signal not observed; **ν_{max}** (neat) = 3231, 2951, 1653, 1522, 1167, 741 cm^{-1}; **MS** (ESI^+) *m/z* 460 $[M+H]^+$, 482 $[M+Na]^+$; **HRMS** (ESI^+) calcd. for $C_{23}H_{34}N_5O_5$ $[M+H]^+$ 460.2554, found 460.2557; $[\alpha]_D^{27}$ +9.7 (*c* 0.06, MeOH).

Cyclo(Trp-Leu-GOx-Gly) (9)

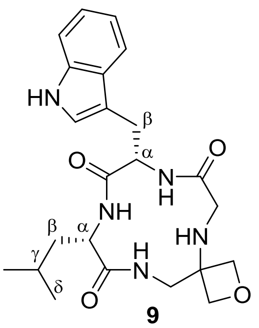

9

To a solution of tetrapeptide H-Trp-Leu-GOx-Gly-OH (**7**) (46 mg, 0.10 mmol, 1.0 equiv) in anhydrous DMF (100 mL, 0.001 M) under an atmosphere of nitrogen was added DEPBT (60 mg, 0.10 mmol, 2.0 equiv) and DIPEA (35 µL, 0.10 mmol, 2.0 equiv) and the reaction mixture was stirred for 48 h at room temperature. The solvent was removed under reduced pressure at 60 °C over 30 min, and the residue was dried *in vacuo*. The residue was analysed by LCMS and purified twice by column chromatography (SiO_2, CH_2Cl_2/MeOH 19:1→9:1) to give cyclic tetrapeptide (**9**) as a yellow solid (29 mg, 65 µmol, 65%). **R_f** (CH_2Cl_2/MeOH 9:1) 0.41; **mp** 200–203 °C; **^{1}H NMR** (500 MHz, DMSO-*d6*) δ_H 10.87 (s, 1H, NH), 8.25 (d, *J* = 10.4 Hz, 1H, NH), 7.97 (d, *J* = 9.1 Hz, 1H, NH), 7.56–7.50 (m, 2H, NH, ArH), 7.34 (d, *J* = 8.0 Hz, 1H, ArH), 7.11 (s, 1H, ArH), 7.08 (t, *J* = 7.5 Hz, 1H, ArH), 7.00 (t, *J* = 7.4 Hz, 1H, ArH), 4.59 (q, *J* = 9.3 Hz, 1H, CHα-Trp), 4.41 (d, *J* = 6.3 Hz, 1H, OC*H*H-Ox), 4.18 (d, *J* = 6.9 Hz, 1H, OC*H*H-Ox), 4.15 (d, *J* = 6.3 Hz, 1H, OCH*H*-Ox), 4.04–3.97 (m, 1H, CHα-Leu), 3.90 (d, *J* = 6.9 Hz, 1H, OCH*H*-Ox), 3.81 (dd, *J* = 13.3, 7.7 Hz, 1H, C*H*HGly or C*H*HGOx), 3.43–3.37 (m, 1H, C*H*HGly or C*H*HGOx), 3.26–3.18 (m, 2H, C*H*HGly or C*H*HGOx, C*H*Hβ-Trp), 3.07–2.97 (m, 2H, C*H*HGly or C*H*HGOx, CH*H*β-Trp), 1.65–1.57 (m, 2H, C*H*Hβ-Leu, CHγ-Leu), 1.53–1.45 (m, 1H, CH*H*β-Leu), 0.92 (d, *J* = 6.1 Hz, 3H, CH_3δ-Leu), 0.79 (d, *J* = 6.1 Hz, 3H, CH_3δ-Leu). *N.B.* Secondary amine NH not observed; **^{13}C NMR** (126 MHz, DMSO-*d6*) δ_C 173.2 (C=O), 172.9 (C=O), 171.4 (C=O), 136.1 (C), 127.1 (C), 123.0 (CH), 121.0 (CH), 118.3 (CH), 118.1 (CH), 111.4 (CH), 109.5 (C), 78.1 (OCH_2), 76.3 (OCH_2), 60.3 (C, Ox), 56.0 (CH, α-Trp), 54.0 (CH, α-Leu), 47.3 (CH_2, GOx or CH_2, Gly), 44.2 (CH_2, GOx or CH_2, Gly), 39.2 (CH_2, β-Leu), 26.5 (CH_2, β-Trp), 24.6 (CH, γ-Leu), 22.8 (CH_3, δ-Leu), 21.3 (CH_3, δ-Leu); **ν_{max}** (neat) = 3278, 2954, 1660, 1516, 740 cm^{-1}; **MS** (ESI^+) *m/z* 464 $[M+Na]^+$; **HRMS** (ESI^+) calcd. for $C_{23}H_{31}N_5NaO_4$ $[M+Na]^+$ 464.2268, found 464.2270; $[\alpha]_D^{26}$ –60.3 (*c* 0.01, MeOH).

Cbz-D-Pro-Leu-GOx-Gly-OBn (36)

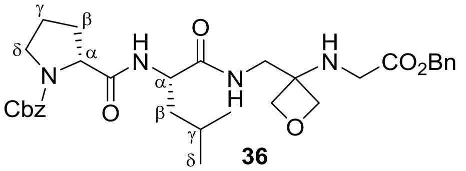

36

To a solution of tripeptide Boc-Leu-GOx-Gly-OBn (**34**) (1.15 g, 2.48 mmol, 1.0 equiv) in CH_2Cl_2 (10.0 mL) was added TFA (10.0 mL) and the mixture was stirred at room temperature for 1 h *(Caution – gas evolution!)*. The reaction mixture was concentrated under reduced pressure and the resulting residue repeatedly dissolved in CH_2Cl_2 (3 × 25 mL) and concentrated under reduced pressure to give the crude amine. The residue was dissolved in CH_2Cl_2 (25 mL), Cbz-D-Pro-OH (0.68 g, 2.73 mmol, 1.1 equiv), EDC·HCl (0.52 g, 2.73 mmol, 1.1 equiv), HOBt·H_2O (0.37 g, 2.73 mmol, 1.1 equiv) and NMM (1.64 mL, 14.9 mmol, 6.0 equiv) were added subsequently, and the reaction mixture was stirred at room temperature for 24 h. The reaction mixture was diluted with EtOAc (50 mL) and washed with brine (50 mL), dried over Na_2SO_4, filtered and concentrated *in vacuo*. The residue was

purified by column chromatography (SiO_2, CH_2Cl_2/MeOH, 40:1) to give tetrapeptide Cbz-D-Pro-Leu-GOx-Gly-OBn (**36**) (1.03 g, 1.73 mmol, 70%) as a colourless viscous oil. **R_f** (CH_2Cl_2/MeOH 49:1) 0.22; **^{1}H NMR** (600 MHz, DMSO-*d6* @ 373 K) δ_H 7.69 (s, 1H, NH), 7.48 (s, 1H, NH), 7.39–7.27 (m, 10H, ArH), 5.15 (s, 2H, CH_2Ph), 5.11–5.02 (m, 2H, CH_2Ph), 4.35 (d, *J* = 6.2 Hz, 2H, OCH_2-Ox), 4.30 (td, *J* = 8.9, 5.3 Hz, 1H, CHα-Leu), 4.28–4.24 (m, 3H, OCH_2-Ox, CHα-Pro), 3.51–3.42 (m, 6H, CH_2δ-Pro, CH_2GOx, CH_2Gly), 2.16–2.08 (m, 1H, C*H*Hβ-Pro or C*H*Hγ-Pro), 1.92–1.78 (m, 3H, CH*H*β-Pro or CH*H*γ-Pro, CH_2β-Pro or CH_2γ-Pro), 1.65–1.54 (m, 2H, C*H*Hβ-Leu, CHγ-Leu), 1.52–1.47 (m, 1H, CH*H*β-Leu), 0.88 (d, *J* = 6.4 Hz, 3H, CH_3δ-Leu), 0.84 (d, *J* = 6.4 Hz, 3H, CH_3δ-Leu). *N.B.* Secondary amine NH not observed; **^{13}C NMR** (151 MHz, DMSO-*d6* @ 373 K) δ_C 172.0 (C=O), 171.5 (C=O), 171.3 (C=O), 153.8 (C=O, Cbz), 136.5 (C), 135.6 (C), 127.8 (CH), 127.7 (CH), 127.4 (CH), 127.3 (CH), 127.0 (CH), 126.7 (CH), 77.7 (2 × OCH_2), 65.6 (CH_2, Bn), 65.2 (CH_2, Bn), 59.7 (CH, α-Pro), 59.3 (C, Ox), 51.1 (CH, α-Leu), 46.4 (CH_2, δ-Pro or CH_2, Gly), 44.2 (CH_2, δ-Pro or CH_2, Gly), 42.5 (CH_2, GOx), 40.3 (CH_2, β-Leu), 29.8 (CH_2, β-Pro or CH_2, γ-Pro), 23.9 (CH, γ-Leu), 23.0 (CH_2, β-Pro or CH_2, γ-Pro), 22.3 (CH_3, δ-Leu), 21.1 (CH_3, δ-Leu); **ν_{max}** (neat) = 3307, 2954, 1738, 1655, 1529, 1171, 737 cm^{-1}; **MS** (ESI$^+$) *m/z* 595 $[M+H]^+$, 617 $[M+Na]^+$; **HRMS** (ESI$^+$) calcd. for $C_{32}H_{42}N_4NaO_7$ $[M+Na]^+$ 617.2946, found 617.2955; $[\alpha]_D^{25}$ +6.7 (*c* 0.11, $CHCl_3$).

H-D-Pro-Leu-GOx-Gly-OH (37)

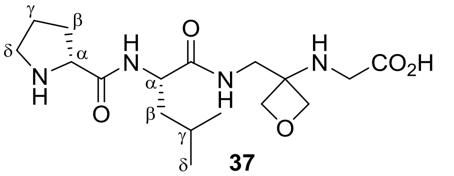

37

To a solution of Cbz-D-Pro-Leu-GOx-Gly-OBn (**36**) (440 mg, 0.74 mmol, 1.0 equiv) in MeOH (10 mL) was added 10 wt% Pd/C (44 mg, 10 wt%) and the reaction flask was evacuated, filled with nitrogen, evacuated, and placed under an atmosphere of hydrogen (balloon). The mixture was stirred at room temperature for 16 h, placed under nitrogen and filtered through a plug of Celite, which was washed with MeOH (3×). The filtrate was concentrated *in vacuo* to give **37** as a white solid (276 mg, 0.74 mmol, quant. yield), which required no further purification; **mp** 67–70 °C; **^{1}H NMR** (500 MHz, D_2O) δ_H 4.68 (d, *J* = 7.6 Hz, 1H, OC*H*H-Ox), 4.67 (d, *J* = 7.6 Hz, 1H, OCH*H*-Ox), 4.59 (d, *J* = 7.7 Hz, 1H, OC*H*H-Ox), 4.56 (d, *J* = 7.7 Hz, 1H, OCH*H*-Ox), 4.46–4.42 (m, 1H, CHα-Pro), 4.39–4.35 (m, 1H, CHα-Leu), 3.78 (d, *J* = 14.7 Hz, 1H, C*H*HGly or C*H*HGOx), 3.69 (d, *J* = 14.7 Hz, 1H, CH*H*Gly or CH*H*GOx), 3.47–3.38 (m, 4H, CH_2Gly or CH_2GOx, CH_2δ-Pro), 2.52–2.43 (m, 1H, C*H*Hβ-Pro or C*H*Hγ-Pro), 2.12–2.00 (m, 3H, CH_2β-Pro or CH_2γ-Pro, CH*H*β-Pro or CH*H*γ-Pro), 1.72–1.61 (m, 3H, CH_2β-Leu, CHγ-Leu), 0.94 (d, *J* = 5.5 Hz, 3H, CH_3δ-Leu), 0.90 (d, *J* = 5.5 Hz, 3H, CH_3δ-Leu); **^{13}C NMR** (126 MHz, D_2O) δ_C 175.6 (C=O), 175.3 (C=O), 169.9 (C=O), 77.4 (OCH_2), 77.2 (OCH_2), 60.1 (C, Ox), 59.7 (CH, α-Pro), 53.0 (CH, α-Leu), 46.5 (CH_2, GOx or CH_2, Gly), 45.5 (CH_2, GOx or CH_2, Gly), 41.6 (CH_2, δ-Pro), 39.6 (CH_2, β-Leu), 29.8 (CH_2, β-Pro or CH_2, γ-Pro), 24.4 (CH, γ-Leu), 23.8 (CH_2, β-Pro or CH_2, γ-Pro), 22.1 (CH_3, δ-Leu), 20.4 (CH_3, δ-Leu); **ν_{max}** (neat) = 3258, 2956, 1655, 1561, 1174, 719 cm^{-1}; **MS** (ESI$^+$) *m/z* 371 $[M+H]^+$, 393 $[M+Na]^+$; **HRMS** (ESI$^+$) calcd. for $C_{17}H_{31}N_4O_5$ $[M+H]^+$ 371.2289, found 371.2283; $[\alpha]_D^{25}$ +162 (*c* 0.03, MeOH).

Cyclo(D-Pro-Leu-GOx-Gly) (10)

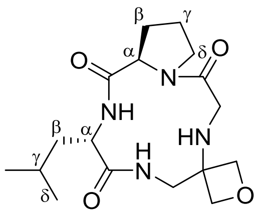

10

To a solution of H-D-Pro-Leu-GOx-Gly-OH (**37**) (74 mg, 0.20 mmol, 1.0 equiv) in anhydrous DMF (200 mL, 0.001 M) under an atmosphere of nitrogen was added DEPBT (120 mg, 0.40 mmol, 2.0 equiv) and DIPEA (70 μL, 0.40 mmol, 2.0 equiv) and the mixture was stirred for 48 h at room temperature. The solvent was removed *in vacuo* and the residue was purified twice by column chromatography (SiO_2, CH_2Cl_2/MeOH 19:1→9:1) to give the cyclic tetrapeptide **10** as a white solid (38 mg, 108 μmol, 54%). $\mathbf{R_f}$ (CH_2Cl_2/MeOH 19:1) 0.28; **mp** 71–73 °C; **^{1}H NMR** (500 MHz, CD_3OD) δ_H 4.72 (d, *J* = 6.7 Hz, 1H, OC*H*H-Ox), 4.57 (d, *J* = 6.9 Hz, 1H, OC*H*H-Ox), 4.46–4.37 (m, 3H, OCH*H*-Ox, CHα-Pro, CHα-Leu), 4.33 (d, *J* = 6.7 Hz, 1H, OCH*H*-Ox), 3.78 (d, *J* = 13.9 Hz, 1H, C*H*HGOx), 3.66–3.52 (m, 2H, CH_2δ-Pro), 3.50–3.38 (m, 3H, CH*H*GOx, CH_2Gly), 2.35–2.24 (m, 1H, C*H*Hβ-Pro or C*H*Hγ-Pro), 2.05–1.86 (m, 3H, CH_2β-Pro or CH_2γ-Pro, CH*H*β-Pro or CH*H*γ-Pro), 1.72–1.63 (m, 2H, C*H*Hβ-Leu, CHγ-Leu), 1.60–1.51 (m, 1H, CH*H*β-Leu), 0.98 (d, *J* = 6.2 Hz, 3H, CH_3δ-Leu), 0.93 (d, *J* = 6.1 Hz, 3H, CH_3δ-Leu); **^{13}C NMR** (126 MHz, CD_3OD) δ_C 176.3 (C=O), 175.8 (C=O), 173.1 (C=O), 80.3 (OCH_2), 79.2 (OCH_2), 62.1 (CH, α-Pro), 62.0 (C, Ox), 53.9 (CH, α-Leu), 48.8 (CH_2, δ-Pro), 48.5 (CH_2, Gly), 47.2 (CH_2, GOx), 38.3 (CH_2, β-Leu), 33.0 (CH_2, γ-Pro), 26.1 (CH, γ-Leu), 23.4 (CH_2, β-Pro), 23.0 (CH_3, δ-Leu), 22.6 (CH_3, δ-Leu); $\mathbf{\nu_{max}}$ (neat) = 3274, 2954, 1657, 1539, 1173, 970 cm^{-1}; **MS** (ESI$^+$) *m/z* 353 [M+H]$^+$, 375 [M+Na]$^+$; **HRMS** (ESI$^+$) calcd. for $C_{17}H_{28}N_4NaO_4$ [M+Na]$^+$ 375.2003, found 375.2006; $[\boldsymbol{\alpha}]_D^{27}$ –132 (*c* 0.06, MeOH).

Cbz-Asp(*t*Bu)-Leu-GOx-Gly-OBn (38)

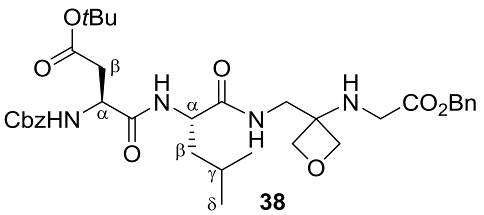

38

To a solution of Boc-Leu-GOx-Gly-OBn (**34**) (487 mg, 1.05 mmol, 1.0 equiv) in CH_2Cl_2 (1.0 mL) was added TFA (1.0 mL) and the mixture was stirred at room temperature for 1 h *(Gas evolution!)*. The reaction mixture was concentrated under reduced pressure and the resulting residue repeatedly dissolved in CH_2Cl_2 (3 × 10 mL) and concentrated *in vacuo* to give the crude amine. The residue was dissolved in CH_2Cl_2 (10.5 mL), Cbz-Asp(*t*Bu)-OH (340 mg, 1.05 mmol, 1.0 equiv), EDC·HCl (202 mg, 1.05 mmol, 1.0 equiv), HOBt·H_2O (142 mg, 1.05 mmol, 1.0 equiv) and NMM (0.46 mL, 4.20 mmol, 4.0 equiv) were added, and the reaction mixture was stirred at room temperature for 24 h. The mixture was diluted with EtOAc (30 mL) and washed with brine (3 × 30 mL), dried over Na_2SO_4, filtered and concentrated *in vacuo*. The residue was purified by column chromatography (SiO_2, CH_2Cl_2/MeOH 25:1) to give tetrapeptide **38** (413 mg, 0.62 mmol, 59%) as a colourless oil; $\mathbf{R_f}$ (CH_2Cl_2/MeOH 25:1) 0.41; **^{1}H NMR** (500 MHz, $CDCl_3$) δ_H ppm 7.39–7.31 (m, 10H, ArH), 6.86 (br. t, 1H, NH), 6.79 (d, *J* = 8.0 Hz, 1H, NH), 5.93 (d, *J* = 8.0 Hz, 1H, NH), 5.16 (s, 2H, CH_2Ph), 5.11 (s, 2H, CH_2Ph), 4.50 (dd, *J* = 13.0, 6.1 Hz, 1H, CHα-Asp), 4.46–4.33 (m, 5H, CH α-Leu, 2 × OCH_2-Ox), 3.68–3.59 (m, 1H, C*H*HGOx), 3.51 (m, 3H, CH*H*GOx, CH_2Gly), 2.86 (dd, *J* = 17.0, 4.5 Hz, 1H, C*H*Hβ-Asp), 2.67 (dd, *J* = 17.0, 6.5 Hz, 1H, CH*H*β-Asp), 1.77–1.69 (m, 1H, C*H*Hβ-Leu), 1.66–1.58 (m, 1H, CHγ-Leu), 1.56–1.49 (m, 1H, CH*H*β-Leu), 1.42 (s, 9H, 3 × CH_3, *t*Bu), 0.91 (d, *J* = 6.5 Hz, 3H, CH_3δ-Leu), 0.88 (d, *J* = 6.4 Hz, 3H, CH_3δ-Leu). *N.B.* Amine NH not observed; **^{13}C NMR** (126 MHz, $CDCl_3$) δ_C ppm 172.8 (C=O), 172.4 (C=O), 171.2 (C=O), 170.7 (C=O), 156.2 (C=O, Cbz), 135.9 (C), 135.3 (C), 128.7 (CH), 128.64 (CH), 128.57 (CH), 128.5 (CH), 128.4 (CH), 128.2 (CH), 82.1 (C, *t*Bu), 79.2 (2 × OCH_2), 67.4 (CH_2, Bn), 67.1 (CH_2, Bn), 59.6 (C, Ox), 52.1 (CH, α-Asp), 51.5 (CH, α-Leu), 44.8 (CH_2, Gly), 43.3 (CH_2, GOx), 40.6 (CH_2, β-Leu), 36.9 (CH_2, β-Asp), 28.0 (CH_3, *t*Bu), 24.7 (CH, γ-Leu), 23.1 (CH_3, δ-Leu), 21.7 (CH_3, δ-Leu); $\mathbf{\nu_{max}}$ (neat) = 3310, 2955, 1716, 1649, 1526, 1150, 696 cm^{-1}; **MS** (ESI$^+$) *m/z* 669 [M+H]$^+$, 691 [M+Na]$^+$; **HRMS** (ESI$^+$) calcd. for $C_{35}H_{48}N_4NaO_9$ [M+Na]$^+$ 691.3313, found 691.3301; $[\boldsymbol{\alpha}]_D^{26}$ –23.0 (*c* 0.12, MeOH).

H-Asp(*t*Bu)-Leu-GOx-Gly-OH (39)

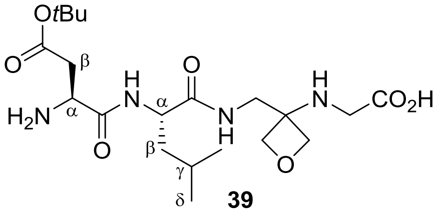

39

To a solution of Cbz-Asp(*t*Bu)-Leu-GOx-Gly-OBn (**38**) (384 mg, 0.57 mmol, 1.0 equiv) in MeOH (6.0 mL) was added 10 wt% Pd/C (40 mg, 10 wt%) and the reaction flask was evacuated, filled with nitrogen, evacuated, and placed under an atmosphere of hydrogen (balloon). The reaction mixture was stirred at room temperature for 16 h, placed under nitrogen and filtered through a plug of Celite, which was washed with MeOH (3×). The filtrate was concentrated *in vacuo* to give H-Asp(*t*Bu)-Leu-GOx-Gly-OH (**39**) as a white solid (255 mg, 0.57 mmol) in quantitative yield; **mp** 92–94 °C; **^{1}H NMR** (500 MHz, CD_3OD) δ_H ppm 4.56–4.51 (m, 2H, OCH_2-Ox), 4.47–4.42 (m, 2H, OCH_2-Ox), 4.32 (dd, *J* = 9.2, 5.2 Hz, 1H, CHα-Leu), 4.10–4.03 (m, 1H, CHα-Asp), 3.70 (d, *J* = 14.2 Hz, 1H, C*H*HGOx), 3.60 (d, *J* = 14.2 Hz, 1H, *C*H*H*GOx), 3.34–3.31 (m, 2H, CH_2Gly), 2.99 (dd, *J* = 17.6, 4.3 Hz, 1H, C*H*Hβ-Asp), 2.78 (dd, *J* = 17.6, 7.6 Hz, 1H, CH*H*β-Asp), 1.71–1.63 (m, 3H, CH_2β-Leu, CHγ-Leu), 1.49 (s, 9H, 3 × CH_3, *t*Bu), 0.97 (d, *J* = 5.9 Hz, 3H, CH_3δ-Leu), 0.93 (d, *J* = 5.9 Hz, 3H, CH_3δ-Leu); **^{13}C NMR** (126 MHz, CD_3OD) δ_C ppm 177.1 (C=O), 175.0 (C=O), 171.4 (C=O), 171.3 (C=O), 83.4 (C, *t*Bu), 79.6 (OCH_2), 79.5 (OCH_2), 61.7 (C, Ox), 54.1 (CH, α-Leu), 51.6 (CH, α-Asp), 47.0 (CH_2, Gly), 43.4 (CH_2, GOx), 41.3 (CH_2, β-Leu), 37.9 (CH_2, β-Asp), 28.3 (CH_3, *t*Bu), 25.9 (CH, γ-Leu), 23.5 (CH_3, δ-Leu), 21.7 (CH_3, δ-Leu); **ν_{max}** (neat) = 3302, 1721, 1648, 1533, 1367, 1152, 975 cm^{-1}; **MS** (ESI^+) *m/z* 445 $[M+H]^+$, 467 $[M+Na]^+$; **HRMS** (ESI^+) calcd. for $C_{20}H_{37}N_4O_7$ $[M+H]^+$ 445.2657, found 445.2657; $[\alpha]_D^{26}$ +6.8 (*c* 0.11, MeOH).

Cyclo(Asp(*t*Bu)-Leu-GOx-Gly) (11)

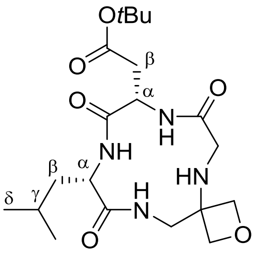

11

To a solution of tetrapeptide H-Asp(*t*Bu)-Leu-GOx-Gly-OH (**39**) (89 mg, 0.20 mmol, 1.0 equiv) in anhydrous DMF (200 mL, 0.001 M) under an atmosphere of nitrogen was added DEPBT (120 mg, 0.40 mmol, 2.0 equiv) and DIPEA (70 µL, 0.40 mmol, 2.0 equiv) and the reaction mixture was stirred for 48 h at room temperature. The DMF was removed under reduced pressure at 60 °C over 30 min, and the residue was dried *in vacuo*. The residue was analysed by LCMS and purified twice by column chromatography (SiO_2, CH_2Cl_2/MeOH 19:1→9:1) to give the cyclic tetrapeptide (**11**) as a white solid (1st run: 41.5 mg, 97 µmol, 49%; 2nd run: 42.7 mg, 100 µmol, 50%). **R_f** (CH_2Cl_2/MeOH 12:1) 0.33; **mp** 134–137 °C; ^{1}H and ^{13}C NMR data reported for the major conformer: **^{1}H NMR** (500 MHz, DMSO-*d6*) δ_H ppm 8.02 (d, *J* = 10.6 Hz, 1H, NH), 7.57 (d, *J* = 8.7 Hz, 1H, NH), 7.37–7.33 (m, 1H, NH), 4.54 (dd, *J* = 18.7, 8.7 Hz, 1H, CHα-Asp), 4.39–4.37 (m, 1H, OC*H*H-Ox), 4.16–4.12 (m, 2H, OC*H*H-Ox, OCH*H*-Ox), 4.00 (td, *J* = 10.1, 5.1 Hz, 1H, CHα-Leu), 3.88 (d, *J* = 7.0 Hz, 1H, OCH*H*-Ox), 3.82 (dd, *J* = 13.5, 8.0 Hz, 1H, C*H*HGOx), 3.37–3.34 (m, 1H, C*H*HGly), 3.24 (d, *J* = 14.4 Hz, 1H, CH*H*Gly), 2.94 (d, *J* = 13.5 Hz, 1H, CH*H*GOx), 2.78 (dd, *J* = 15.8, 8.9 Hz, 1H, C*H*Hβ-Asp), 2.62 (dd, *J* = 15.8, 8.2 Hz, 1H, CH*H*β-Asp), 1.65–1.59 (m, 1H, CHγ-Leu), 1.56–1.50 (m, 1H, C*H*Hβ-Leu), 1.49–1.43 (m, 1H, CH*H*β-Leu), 1.36 (s, 9H, 3 × CH_3, *t*Bu), 0.91 (d, *J* = 6.5 Hz, 3H, CH_3δ-Leu), 0.84–0.81 (m, 3H, CH_3δ-Leu). *N.B.* Amine NH not observed; **^{13}C NMR** (126 MHz, DMSO-*d6*) δ_C ppm 173.0 (C=O), 172.8 (C=O), 170.0 (C=O), 168.6 (C=O), 80.5 (C, *t*Bu), 78.0 (OCH_2), 76.0 (OCH_2), 60.3 (C, Ox), 54.1 (CH, α-Asp), 51.8 (CH, α-Leu), 47.1 (CH_2, Gly), 44.2 (CH_2, GOx), 39.2 (CH_2, β-Leu), 36.7 (CH_2, β-Asp), 27.6 (CH_3, *t*Bu), 24.6 (CH, γ-Leu), 22.8 (CH_3, δ-Leu), 21.3 (CH_3, δ-Leu). *N.B.* CH_2, β-Leu overlaps with solvent peak; **ν_{max}** (neat) = 3308, 2956, 1726, 1656, 1520, 1153, 971 cm^{-1}; **MS** (ESI^+) *m/z* 449 $[M+Na]^+$, 875 $[2M+Na]^+$; **HRMS** (ESI^+) calcd. for $C_{20}H_{34}N_4NaO_6$ $[M+Na]^+$ 449.2371, found 449.2369; $[\alpha]_D^{26}$ –74.5 (*c* 0.07, MeOH).

*2.4 Preparation of cyclic tetrapeptides **8** and **48**, and cyclic octapeptide **42** and **45***

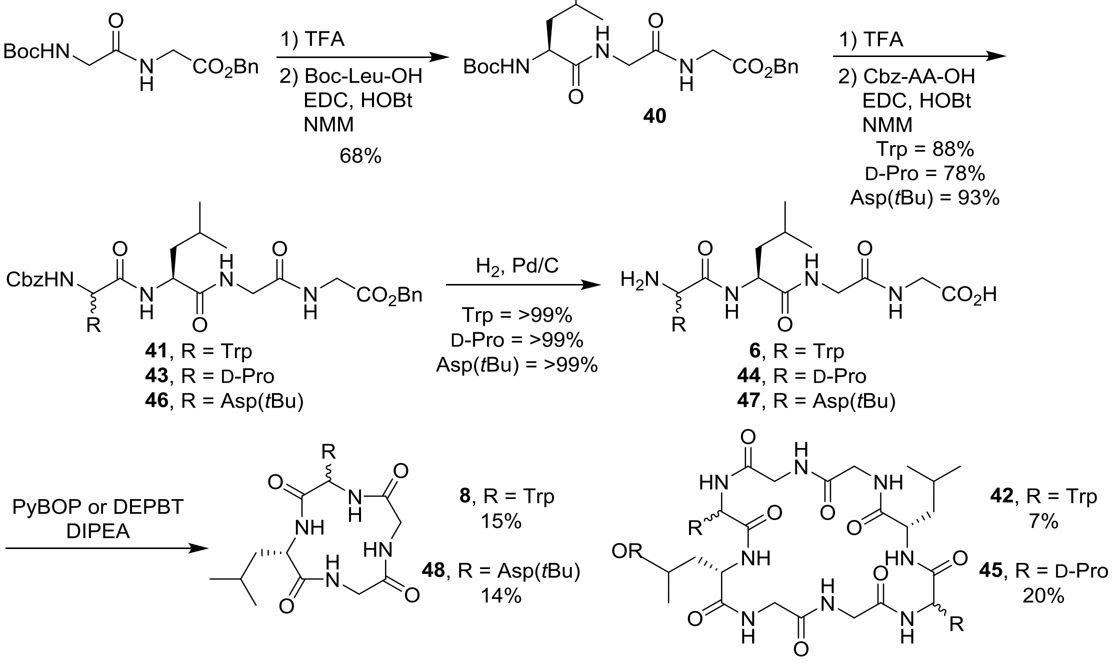

Boc-Leu-Gly-Gly-OBn (40)

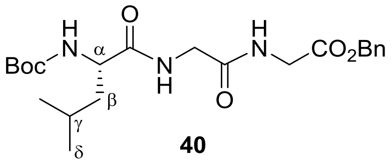

To a solution of dipeptide TsOH·H-Gly-Gly-OBn[3] (3.94 g, 10.0 mmol, 1.0 equiv) in CH_2Cl_2 (100 mL) was added Boc-Leu-OH (2.43 g, 10.5 mmol, 1.05 equiv), EDC·HCl (2.01 g, 10.5 mmol, 1.05 equiv), HOBt·H_2O (1.42 g, 10.5 mmol, 1.05 equiv) and NMM (4.40 mL, 40.0 mmol, 4.0 equiv), and the mixture was stirred at room temperature for 24 h. The mixture was diluted with EtOAc (100 mL) and washed with brine (3 × 100 mL), dried over Na_2SO_4, filtered and concentrated *in vacuo*. The residue was purified by column chromatography (SiO_2, CH_2Cl_2/EtOAc 1:1→EtOAc) to give Boc-Leu-Gly-Gly-OBn (**40**) (2.98 g, 6.84 mmol, 68%) as a white solid. $\mathbf{R_f}$ (CH_2Cl_2/EtOAc 1:1) 0.26; **mp** 53–57 °C; **^{1}H NMR** (500 MHz, $CDCl_3$) δ_H 7.38–7.30 (m, 5H, ArH), 7.24 (s, 1H, NH), 7.06 (s, 1H, NH), 5.16 (s, 2H, CH_2Ph), 5.09 (d, *J* = 5.7 Hz, 1H, NH), 4.15–4.04 (m, 3H, CH_2Gly, CHα-Leu), 4.00 (dd, *J* = 18.1, 5.0 Hz, 1H, C*H*HGly), 3.94 (dd, *J* = 16.8, 5.4 Hz, 1H, CH*H*Gly), 1.72–1.62 (m, 2H, C*H*Hβ-Leu, CHγ-Leu), 1.55–1.46 (m, 1H, CH*H*β-Leu), 1.40 (s, 9H, 3 × CH_3, Boc), 0.94 (d, *J* = 6.4 Hz, 3H, CH_3δ-Leu), 0.92 (d, *J* = 6.4 Hz, 3H, CH_3δ-Leu); **^{13}C NMR** (126 MHz, $CDCl_3$) δ_C 173.5 (C=O), 169.7 (C=O), 169.6 (C=O), 156.3 (C=O, Boc), 135.3 (C), 128.8 (CH), 128.6 (CH), 128.5 (CH), 80.6 (C, Boc), 67.3 (CH_2, Bn), 53.8 (CH, α-Leu), 43.1 (CH_2, Gly), 41.4 (CH_2, Gly), 41.0 (CH_2, β-Leu), 28.4 (CH_3, Boc), 24.9 (CH, γ-Leu), 23.1 (CH_3, δ-Leu), 22.0 (CH_3, δ-Leu); $\mathbf{\nu_{max}}$ (neat) = 3306, 2957, 1746, 1654, 1518, 1163, 954, 736 cm^{-1}; **MS** (ESI^+) *m/z* 436 $[M+H]^+$, 458 $[M+Na]^+$; **HRMS** (ESI^+) calcd. for $C_{22}H_{33}N_3NaO_6$ $[M+Na]^+$ 458.2268, found 458.2264; $[\alpha]_D^{25}$ +2.4 (*c* 0.12, $CHCl_3$).

Cbz-Trp-Leu-Gly-Gly-OBn (41)

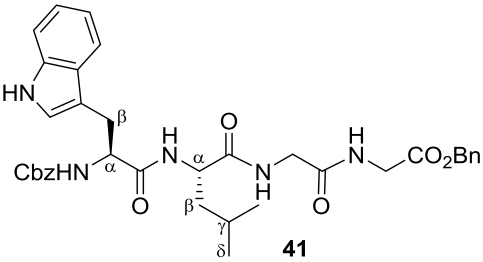

41

To a solution of Boc-Leu-Gly-Gly-OBn (**40**) (0.93 g, 2.15 mmol, 1.0 equiv) in CH_2Cl_2 (2.5 mL) was added TFA (2.5 mL) and the mixture was stirred at room temperature for 1 h *(Gas evolution!)*. The mixture was concentrated under reduced pressure and the resulting residue repeatedly dissolved in CH_2Cl_2 (3 × 25 mL) and concentrated *in vacuo* to give the crude amine. The residue was dissolved in CH_2Cl_2 (22 mL), Cbz-Trp-OH (0.73 g, 2.15 mmol, 1.0 equiv), EDC·HCl (0.41 g, 2.15 mmol, 1.0 equiv), HOBt·H_2O (0.29 g, 2.15 mmol, 1.0 equiv) and NMM (0.95 mL, 8.60 mmol, 4.0 equiv) were added, and the mixture was stirred at room temperature for 24 h. The mixture was diluted with EtOAc (25 mL) and washed with brine (3 × 25 mL), dried over Na_2SO_4, filtered and concentrated *in vacuo*. The residue was purified by column chromatography (SiO_2, CH_2Cl_2/MeOH 40:1) to give Cbz-Trp-Leu-Gly-Gly-OBn (**41**) (1.21 g, 1.89 mmol, 88%) as a white solid. $\mathbf{R_f}$ (CH_2Cl_2/MeOH 40:1) 0.13; **mp** 170–173 °C; **^{1}H NMR** (500 MHz, CD_3CN) δ_H 9.28 (s, 1H, NH), 7.60 (d, *J* = 7.8 Hz, 1H, ArH), 7.40–7.27 (m, 11H, ArH), 7.21 (t, *J* = 5.5 Hz, 1H, NH), 7.13–7.09 (m, 2H, ArH), 7.06–7.03 (m, 1H, ArH), 6.97 (s, 1H, NH), 6.01 (d, *J* = 6.8 Hz, 1H, NH), 5.12 (s, 2H, CH_2Ph), 5.06–4.97 (m, 2H, CH_2Ph), 4.42 (q, *J* = 7.0 Hz, 1H, CHα-Trp), 4.18 (m, 1H, CHα-Leu), 3.92 (dd, *J* = 5.8, 1.4 Hz, 2H, CH_2Gly), 3.74 (dd, *J* = 17.0, 6.3 Hz, 1H, C*H*HGly), 3.68 (dd, *J* = 17.0, 5.9 Hz, 1H, CH*H*Gly), 3.24 (dd, *J* = 14.7, 6.0 Hz, 1H, C*H*Hβ-Trp), 3.08 (dd, *J* = 14.6, 7.7 Hz, 1H, CH*H*β-Trp), 1.56–1.45 (m, 3H, CH_2β-Leu, CHγ-Leu), 0.87 (d, *J* = 6.0 Hz, 3H, CH_3δ-Leu), 0.82 (d, *J* = 6.0 Hz, 3H, CH_3δ-Leu); **^{13}C NMR** (126 MHz, CD_3CN) δ_C 173.7 (C=O), 173.4 (C=O), 170.8 (C=O), 170.7 (C=O), 157.4 (C=O, Cbz), 138.0 (C), 137.4 (C), 137.1 (C), 129.52 (CH), 129.46 (CH), 129.2 (CH), 129.1 (CH), 128.9 (CH), 128.7 (CH), 128.4 (C), 124.9 (CH), 122.6 (CH), 120.1 (CH), 119.4 (CH), 112.4 (CH), 110.8 (C), 67.5 (CH_2, Bn), 67.3 (CH_2, Bn), 56.9 (CH, α-Trp), 53.5 (CH, α-Leu), 43.3 (CH_2, Gly), 41.9 (CH_2, Gly), 40.7 (CH_2, β-Leu), 28.4 (CH_2, β-Trp), 25.3 (CH, γ-Leu), 23.3 (CH_3, δ-Leu), 21.8 (CH_3, δ-Leu); $\mathbf{\nu_{max}}$ (neat) = 3285, 2954, 1667, 1226, 740 cm^{-1}; **MS** (ESI$^+$) *m/z* 678 [M+Na]$^+$; **HRMS** (ESI$^+$) calcd. for $C_{36}H_{41}N_5NaO_7$ [M+Na]$^+$ 678.2898, found 678.2901; $[\boldsymbol{\alpha}]_D^{26}$ −1.5 (*c* 0.24, MeOH).

H-Trp-Leu-Gly-Gly-OH (6)

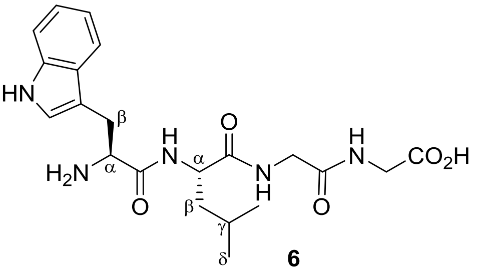

6

To a solution of tetrapeptide Cbz-Trp-Leu-Gly-Gly-OBn (**41**) (900 mg, 1.37 mmol, 1.0 equiv) in MeOH (15 mL) was added 10 wt% Pd/C (90 mg, 10 wt%) and the reaction flask was evacuated, filled with nitrogen, evacuated, and placed under an atmosphere of hydrogen (balloon). The reaction mixture was stirred at room temperature for 16 h, placed under nitrogen and filtered through a plug of Celite, which was washed with MeOH (3×). The filtrate was concentrated *in vacuo* to give H-Trp-Leu-Gly-Gly-OH (**6**) as a white solid (590 mg, 1.37 mmol, quant. yield), which required no further purification. **mp** 210–213 °C; **^{1}H NMR** (500 MHz, D_2O) δ_H 7.63 (d, *J* = 8.0 Hz, 1H, ArH), 7.52 (d, *J* = 8.2 Hz, 1H, ArH), 7.29 (s, *J* = 7.3 Hz, 1H, ArH), 7.26 (t, *J* = 7.6 Hz, 1H, ArH), 7.16 (t, *J* = 7.5 Hz, 1H, ArH), 4.34 (t, *J* = 7.1 Hz, 1H, CHα-Leu or CHα-Trp), 4.30 (t, *J* = 7.3 Hz, 1H, CHα-Leu or CHα-Trp), 3.89–3.70 (m, 4H, 2 × CH_2Gly), 3.44–3.35 (m, 2H, CH_2β-Trp), 1.57–1.47 (m, 3H, CH_2β-Leu, CHγ-Leu), 0.88 (d, *J* = 6.2 Hz, 3H, CH_3δ-Leu), 0.84 (d, *J* = 6.2 Hz, 3H, CH_3δ-Leu); **^{13}C NMR** (126 MHz, D_2O) δ_C 174.9 (C=O), 173.8 (C=O), 170.9 (C=O), 169.4 (C=O), 136.1 (C), 126.5 (C), 125.2 (CH), 122.2 (CH), 119.6 (CH), 118.0 (CH), 112.0 (CH), 106.1 (C), 53.4 (CH, α-Leu or CH, α-Trp), 52.6 (CH, α-Leu or CH, α-Trp), 42.23

(CH$_2$, Gly), 42.16 (CH$_2$, Gly), 39.8 (CH$_2$, β-Leu), 26.7 (CH$_2$, β-Trp), 24.1 (CH, γ-Leu), 21.9 (CH$_3$, δ-Leu), 20.9 (CH$_3$, δ-Leu); **ν$_{max}$** (neat) = 3276, 2956, 1630, 1529, 741 cm^{-1}; **MS** (ESI$^+$) *m/z* 432 [M+H]$^+$, 454 [M+Na]$^+$; **HRMS** (ESI$^+$) calcd. for $C_{21}H_{29}N_5NaO_5$ [M+Na]$^+$ 454.2061, found 454.2054; $[\boldsymbol{\alpha}]_D^{27}$ +14.3 (*c* 0.07, MeOH).

Cyclo(Trp-Leu-Gly-Gly) (8)

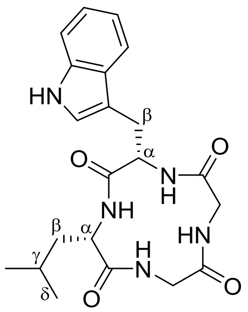

8

To a solution of H-Trp-Leu-Gly-Gly-OH (**6**) (86 mg, 0.20 mmol, 1.0 equiv) in anhydrous DMF (200 mL, 0.001 M) under an atmosphere of nitrogen was added DEPBT (120 mg, 0.40 mmol, 2.0 equiv) and DIPEA (70 μL, 0.40 mmol, 2.0 equiv) and the mixture was stirred for 48 h at room temperature. The solvent was removed under reduced pressure, and the residue was purified twice by column chromatography (SiO$_2$, CH$_2$Cl$_2$/MeOH 92.5:7.5→4:1) to give cyclic tetrapeptide **8** as a yellow solid (1st run: 10.3 mg, 25 μmol, 13%; 2nd run: 13.0 mg, 31 μmol, 16%) and cyclic octapeptide **42** as a yellow solid (11.3 mg, 14 μmol, 7%). Analytical data for cyclic tetrapeptide **8**: **R$_f$** (CH$_2$Cl$_2$/MeOH 9:1) 0.24; **mp** 255–257 °C; NMR data reported for the major conformational isomer at 298 K. **^{1}H NMR** (500 MHz, DMSO-*d6*) δ$_H$ 10.89 (s, 1H, NH), 8.53 (dd, *J* = 7.9, 4.3 Hz, 1H, NH), 8.38 (t, *J* = 9.8 Hz, 2H, 2 × NH), 8.18 (s, 1H, NH), 7.56 (d, *J* = 7.8 Hz, 1H, ArH), 7.33 (d, *J* = 8.0 Hz, 1H, ArH), 7.12 (s, 1H, ArH), 7.07 (t, *J* = 7.5 Hz, 1H, ArH), 6.98 (t, *J* = 7.4 Hz, 1H, ArH), 4.43 (dd, *J* = 17.2, 8.6 Hz, 1H, CHα-Trp), 4.21 (dd, *J* = 17.1, 8.4 Hz, 1H, CHα-Leu), 3.77 (dd, *J* = 14.2, 8.8 Hz, 1H, C*H*HGly), 3.74 (dd, *J* = 14.2, 8.8 Hz, 1H, CH*H*Gly), 3.58 (dd, *J* = 14.0, 4.0 Hz, 1H, C*H*HGly), 3.44 (dd, *J* = 14.0, 4.0 Hz, 1H, CH*H*Gly), 3.18 (dd, *J* = 14.6, 7.5 Hz, 1H, C*H*Hβ-Trp), 3.07 (dd, *J* = 14.6, 8.8 Hz, 1H, CH*H*β-Trp), 1.62–1.54 (m, 3H, CHγ-Leu, CH$_2$β-Leu), 0.90 (d, *J* = 6.2 Hz, 3H, CH$_3$δ-Leu), 0.83 (d, *J* = 6.1 Hz, 3H, CH$_3$δ-Leu). *N.B.* CH$_2$β-Leu overlaps with DMSO solvent peak. **^{13}C NMR** (126 MHz, DMSO-*d6*) δ$_H$ 172.7 (C=O), 172.0 (C=O), 169.9 (C=O), 169.4 (C=O), 136.1 (C), 127.1 (C), 123.3 (CH), 120.9 (CH), 118.3 (CH), 118.1 (CH), 111.4 (CH), 109.7 (C), 56.8 (CH, α-Trp), 53.7 (CH, α-Leu), 43.8 (CH$_2$, Gly), 43.5 (CH$_2$, Gly), 39.6 (CH$_2$, β-Leu), 26.8 (CH$_2$, β-Trp), 24.6 (CH, γ-Leu), 22.4 (CH$_3$, δ-Leu), 22.0 (CH$_3$, δ-Leu); **ν$_{max}$** (neat) = 3286, 3057, 1650, 1530, 739 cm^{-1}; **MS** (ESI$^+$) *m/z* 436 [M+Na]$^+$; **HRMS** (ESI$^+$) calcd. for $C_{21}H_{27}N_5NaO_4$ [M+Na]$^+$ 436.1955, found 436.1954; $[\boldsymbol{\alpha}]_D^{30}$ –207 (*c* 0.05, DMF).

Cyclo(Trp-Leu-Gly-Gly-Trp-Leu-Gly-Gly) (42)

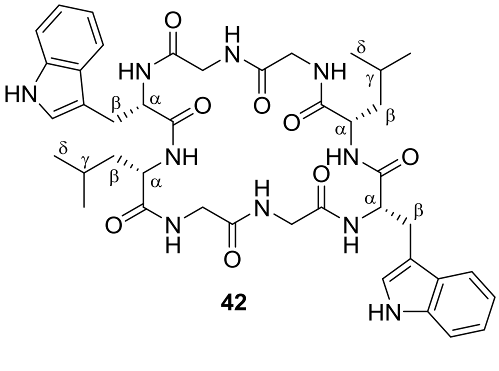

42

Analytical data for cyclic octapeptide **42**: **R$_f$** (CH$_2$Cl$_2$/MeOH 9:1) 0.20; **mp** 216–219 °C; **^{1}H NMR** (500 MHz, CD$_3$OD) δ$_H$ 7.59 (d, *J* = 7.9 Hz, 2H, ArH), 7.36 (d, *J* = 8.1 Hz, 2H, ArH), 7.20 (s, 2H, ArH), 7.11 (t, *J* = 7.5 Hz, 2H, ArH), 7.02 (t, *J* = 7.5 Hz, 2H, ArH), 4.55 (dd, *J* = 8.2, 5.2 Hz, 2H, CHα-Trp), 4.20 (dd, *J* = 10.6, 4.4 Hz, 2H, CHα-Leu), 4.01 (d, *J* = 16.8 Hz, 2H, C*H*HGly), 3.86 (d, *J* = 16.1 Hz, 2H, C*H*HGly), 3.74 (d, *J* = 16.1 Hz, 2H, CH*H*Gly), 3.65 (d, *J* = 16.8 Hz, 2H, CH*H*Gly), 3.39–3.34 (m, 2H, C*H*Hβ-Trp), 3.23 (dd, *J* = 14.9, 8.3 Hz, 2H, CH*H*β-Trp), 1.73–1.65 (m, 2H, C*H*Hβ-Leu), 1.54 (ddd, *J* = 13.9, 9.6, 4.5 Hz, 2H, CH*H*β-Leu), 1.36–1.26 (m, 2H, CHγ-Leu), 0.84 (d, *J* = 6.6 Hz, 6H, CH$_3$δ-Leu), 0.78 (d, *J* = 6.6 Hz, 6H, CH$_3$δ-Leu); **^{13}C NMR** (126 MHz, CD$_3$OD) δ$_C$ 175.7 (C=O), 174.9 (C=O), 173.0 (C=O), 172.6 (C=O), 138.1 (C), 128.6 (C), 124.7 (CH), 122.6 (CH), 120.0 (CH), 119.2 (CH), 112.4 (CH), 110.5 (C), 56.8

(CH, α-Trp), 54.0 (CH, α-Leu), 44.1 (CH_2, Gly), 43.4 (CH_2, Gly), 40.6 (CH_2, β-Leu), 27.9 (CH_2, β-Trp), 25.7 (CH, γ-Leu), 23.7 (CH_3, δ-Leu), 21.6 (CH_3, δ-Leu); **ν_{max}** (neat) = 3298, 2869, 1645, 1522, 1234, 742 cm^{-1}; **MS** (ESI^+) *m/z* 849 $[M+Na]^+$; **HRMS** (ESI^+) calcd. for $C_{42}H_{54}N_{10}NaO_8$ $[M+Na]^+$ 849.4018, found 849.4020; $[\alpha]_D^{27}$ –16.6 (*c* 0.04, MeOH).

Cbz-D-Pro-Leu-Gly-Gly-OBn (43)

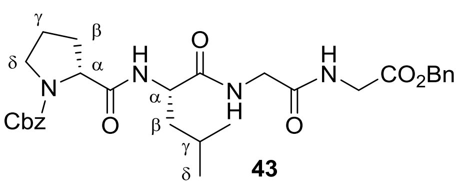

To a solution of Boc-Leu-Gly-Gly-OBn (**40**) (1.23 g, 2.84 mmol, 1.0 equiv) in CH_2Cl_2 (3.0 mL) was added TFA (3.0 mL) and the mixture was stirred at room temperature for 1 h *(Gas evolution!)*. The mixture was concentrated under reduced pressure and the resulting residue repeatedly dissolved in CH_2Cl_2 (3 × 25 mL) and concentrated *in vacuo* to give the crude amine. The residue was dissolved in CH_2Cl_2 (28 mL), Cbz-D-Pro-OH (0.71 g, 2.84 mmol, 1.0 equiv), EDC·HCl (0.54 g, 2.84 mmol, 1.0 equiv), HOBt·H_2O (0.38 g, 2.84 mmol, 1.0 equiv) and NMM (1.15 mL, 11.4 mmol, 4.0 equiv) were added, and the reaction mixture was stirred at room temperature for 24 h. The mixture was diluted with EtOAc (25 mL) and washed with brine (3 × 25 mL), dried over Na_2SO_4, filtered and concentrated *in vacuo*. The residue was purified by column chromatography (SiO_2, CH_2Cl_2/MeOH 38:1) to give Cbz-D-Pro-Leu-Gly-Gly-OBn (**43**) (1.25 g, 2.21 mmol, 78%) as a white solid. **R_f** (CH_2Cl_2/MeOH 38:1) 0.20; **mp** 146–149 °C; **^{1}H NMR** (500 MHz, DMSO-*d6*) δ_H ppm 7.84 (s, 1H, NH), 7.79–7.65 (m, 2H, 2 × NH), 5.16 (s, 2H, CH_2Ph), 5.10 (d, *J* = 12.8 Hz, 1H, C*H*HPh), 5.05 (d, *J* = 12.8 Hz, 1H, CH*H*Ph), 4.38–4.25 (m, 2H, CHα-Pro, CHα-Leu), 4.00–3.88 (m, 2H, CH_2Gly), 3.83–3.72 (m, 2H, CH_2Gly), 3.54–3.43 (m, 2H, CH_2δ-Pro), 2.21–2.11 (m, 1H, C*H*Hγ-Pro), 1.96–1.87 (m, 2H, CH*H*γ-Pro, C*H*Hβ-Pro), 1.87–1.78 (m, 1H, CH*H*β-Pro), 1.70–1.50 (m, 3H, CH_2β-Leu, CHγ-Leu), 0.94–0.83 (d, 6H, *J* = 6.3 Hz, 2 × CH_3δ-Leu); **^{13}C NMR** (126 MHz, DMSO-*d6*) δ_C ppm 172.96 (C=O), 172.94 (C=O), 170.2 (C=O), 170.0 (C=O), 155.3 (C=O, Cbz), 137.9 (C), 136.9 (C), 129.2 (CH), 129.1 (CH), 128.8 (CH), 128.5 (CH), 128.4 (CH), 128.1 (CH), 67.0 (CH_2, Bn), 66.8 (CH_2, Bn), 61.1 (CH, α-Pro), 52.6 (CH_2, α-Leu), 47.8 (CH_2, δ-Pro), 43.1 (CH_2, Gly), 41.8 (CH_2, Gly), 41.5 (CH_2, β-Leu), 31.2 (CH_2, γ-Pro), 25.2 (CH, γ-Leu), 24.4 (CH_2, β-Pro), 23.7 (CH_3, δ-Leu), 22.5 (CH_3, δ-Leu); **ν_{max}** (neat) = 3066, 3010, 1745, 1706, 1649, 1420, 1171, 694 cm^{-1}; **MS** (ESI^+) *m/z* 589 $[M+Na]^+$; **HRMS** (ESI^+) calcd. for $C_{30}H_{38}N_4NaO_7$ $[M+Na]^+$ 589.2633, found 589.2637; $[\alpha]_D^{26}$ +7.3 (*c* 0.16, MeOH).

H-D-Pro-Leu-Gly-Gly-OH (44)

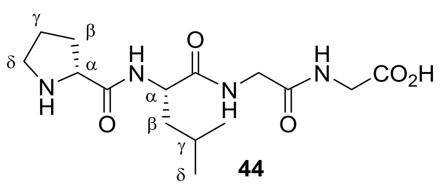

To a solution of Cbz-D-Pro-Leu-Gly-Gly-OBn (**43**) (1.00 g, 1.77 mmol, 1.0 equiv) in MeOH (18 ml) was added 10 wt% Pd/C (100 mg, 10 wt%) and the reaction flask was evacuated, filled with nitrogen, evacuated, and placed under an atmosphere of hydrogen (balloon). The mixture was stirred at room temperature for 16 h, placed under nitrogen and filtered through a plug of Celite, which was washed with MeOH (3×). The filtrate was concentrated *in vacuo* to give H-D-Pro-Leu-Gly-Gly-OH (**44**) as a white solid (604 mg, 1.77 mmol) in quantitative yield. **mp** 137–140 °C; **^{1}H NMR** (500 MHz, D_2O) δ_H ppm 4.47–4.38 (m, 2H, CHα-Pro, CHα-Leu), 4.00 (d, *J* = 17.0 Hz, 1H, C*H*HGly), 3.94 (d, *J* = 17.0 Hz, 1H, CH*H*Gly), 3.83–3.75 (m, 2H, CH_2Gly), 3.49–3.37 (m, 2H, CH_2δ-Pro), 2.54–2.43 (m, 1H, C*H*Hγ-Pro), 2.11–2.03 (m, 3H, CH*H*γ-Pro, CH_2β-Pro), 1.72–1.63 (m, 3H, CH_2β-Leu, CHγ-Leu), 0.95 (d, *J* = 5.6 Hz, 3H, CH_3δ-Leu), 0.90 (d, *J* = 5.6 Hz, 3H, CH_3δ-Leu); **^{13}C NMR** (126 MHz, D_2O) δ_C ppm 176.3 (C=O), 175.0 (C=O), 170.9 (C=O), 170.0 (C=O), 59.7 (CH, α-Pro), 52.9 (CH, α-Leu), 46.5 (CH_2, δ-Pro), 43.1 (CH_2, Gly), 42.4 (CH_2, Gly), 39.5 (CH_2, β-Leu), 29.8 (CH_2, γ-Pro), 24.4 (CH, γ-Leu), 23.7 (CH_2, β-Pro),

22.1 (CH_3, δ-Leu), 20.5 (CH_3, δ-Leu); **ν_{max}** (neat) = 3637, 3256, 1654, 1523, 1383, 1249, 668 cm^{-1}; **MS** (ESI^+) *m/z* 365 $[M+Na]^+$; **HRMS** (ESI^+) calcd. for $C_{15}H_{26}N_4NaO_5$ $[M+Na]^+$ 365.1795, found 365.1797; $[\boldsymbol{\alpha}]_D^{26}$ –9.3 (*c* 0.20, MeOH).

Cyclo(D-Pro-Leu-Gly-Gly-D-Pro-Leu-Gly-Gly) (45)

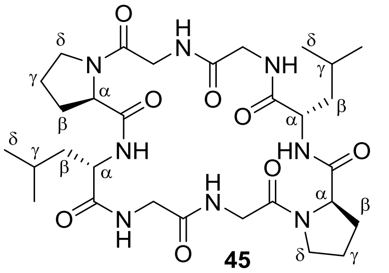

To a solution of tetrapeptide H-D-Pro-Leu-Gly-Gly-OH (**44**) (68 mg, 0.20 mmol, 1.0 equiv) in anhydrous DMF (200 mL, 0.001 M) under an atmosphere of nitrogen was added DEPBT (120 mg, 0.40 mmol, 2.0 equiv) and DIPEA (70 μL, 0.40 mmol, 2.0 equiv) and the reaction mixture was stirred for 48 h at room temperature. The DMF was removed under reduced pressure at 60 °C over 30 min and the residue was purified twice by column chromatography (SiO_2, CH_2Cl_2/MeOH 6:1→4:1) to give the cyclic octapeptide **45** as a white solid (1st run: 26.2 mg, 40 μmol, 20%; 2nd run (0.10 mmol scale): 12.9 mg, 20 μmol, 20%). **R_f** (CH_2Cl_2/MeOH 4:1) 0.31; **mp** 206–207 °C (decomposition); **^{1}H NMR** (500 MHz, DMSO-*d6*) δ_H ppm 8.60 (d, *J* = 8.0 Hz, 2H, NH), 8.18 (t, *J* = 6.3 Hz, 2H, NH), 7.59 (t, *J* = 6.1 Hz, 2H, NH), 4.22 (t, *J* = 6.8 Hz, 2H, CHα-Pro), 4.06 (ddd, *J* = 11.4, 8.0, 3.2 Hz, 2H, CHα-Leu), 3.98 (dd, *J* = 17.0, 6.6 Hz, 2H, C*H*HGly), 3.92 (dd, *J* = 16.6, 7.5 Hz, 2H, C*H*HGly), 3.78 (dd, *J* = 17.0, 5.5 Hz, 2H, CH*H*Gly), 3.60–3.54 (m, 2H, C*H*Hδ-Pro), 3.52–3.46 (m, 4H, CH*H*δ-Pro, CH*H*Gly), 2.11–2.01 (m, 4H, C*H*Hβ-Pro, C*H*Hγ-Pro), 1.92–1.84 (m, 2H, CH*H*γ-Pro), 1.82–1.74 (m, 2H, CH*H*β-Pro), 1.68–1.52 (m, 6H, CH_2β-Leu, CHγ-Leu), 0.89 (d, *J* = 6.0 Hz, 6H, CH_3δ-Leu), 0.81 (d, *J* = 5.9 Hz, 6H, CH_3δ-Leu); **^{13}C NMR** (126 MHz, DMSO-*d6*) δ_C ppm 172.4 (C=O), 172.2 (C=O), 169.9 (C=O), 167.4 (C=O), 60.3 (CH, α-Pro), 51.6 (CH, α-Leu), 46.0 (CH_2, δ-Pro), 41.9 (CH_2, Gly), 41.1 (CH_2, Gly), 28.5 (CH_2, γ-Pro), 25.1 (CH_2, β-Pro), 24.4 (CH, γ-Leu), 23.1 (CH_3, δ-Leu), 20.9 (CH_3, δ-Leu). *N.B.* CH_2, β-Leu overlaps with DMSO solvent peak; **ν_{max}** (neat) = 2955, 1738, 1681, 1630, 1544, 1241, 1160, 650 cm^{-1}; **MS** (ESI^+) *m/z* 671 $[M+Na]^+$; **HRMS** (ESI^+) calcd. for $C_{30}H_{48}N_8NaO_8$ $[M+Na]^+$ 671.3487, found 671.3490.

Cbz-Asp(*t*Bu)-Leu-Gly-Gly-OBn (46)

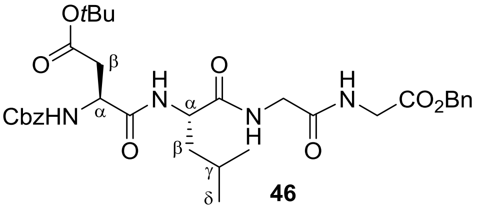

To a solution of Boc-Leu-Gly-Gly-OBn (**40**) (1.09 g, 2.50 mmol, 1.0 equiv) in CH_2Cl_2 (2.5 mL) was added TFA (2.5 mL) and the mixture was stirred at room temperature for 1 h *(Gas evolution!)*. The mixture was concentrated under reduced pressure and the resulting residue repeatedly dissolved in CH_2Cl_2 (3 x 25 mL) and concentrated *in vacuo* to give the crude amine. The residue was dissolved in CH_2Cl_2 (25 mL), Cbz-Asp(*t*Bu)-OH·H_2O (0.81 g, 2.50 mmol, 1.0 equiv), EDC·HCl (0.48 g, 2.50 mmol, 1.0 equiv), HOBt·H_2O (0.34 g, 2.50 mmol, 1.0 equiv) and NMM (1.10 mL, 10.0 mmol, 4.0 equiv) were added, and the reaction mixture was stirred at room temperature for 24 h. The mixture was diluted with EtOAc (25 mL) and washed with brine (3 × 25 mL), dried over Na_2SO_4, filtered and concentrated under reduced pressure. The residue was purified by column chromatography (SiO_2, CH_2Cl_2/MeOH 32:1) to give tetrapeptide **46** (1.49 g, 2.33 mmol, 93%) as a white solid; **R_f** (CH_2Cl_2/MeOH 32:1) 0.35; **mp** 136–139 °C; **^{1}H NMR** (500 MHz, $CDCl_3$) δ_H ppm 7.39–7.28 (m, 11H, ArH, NH), 7.08 (t, *J* = 5.3 Hz, 1H, NH), 6.92 (d, *J* = 6.7 Hz, 1H, NH), 5.92 (d, *J* = 8.1 Hz, 1H, NH), 5.13 (s, 2H, CH_2Ph), 5.11 (s, 2H, CH_2Ph), 4.49 (dd, *J* = 12.3, 7.1 Hz, 1H, CHα-Asp), 4.35–4.28 (m, 1H, CHα-Leu), 4.09 (dd, *J* = 18.0, 5.6 Hz, 1H, C*H*HGly), 4.01 (dd, *J* = 18.0, 5.3 Hz, 1H, CH*H*Gly), 3.98–3.91 (m, 2H, CH_2Gly), 2.80 (dd, *J* = 17.0, 4.4 Hz, 1H, C*H*Hβ-Asp), 2.74 (dd, *J* = 17.0, 6.9 Hz, 1H, CH*H*β-Asp), 1.77–1.53 (m, 3H, CH_2β-Leu, CHγ-Leu), 1.40 (s, 9H, 3 × CH_3, *t*Bu),

0.93 (d, *J* = 6.4 Hz, 3H, CH_3δ-Leu), 0.88 (d, *J* = 6.4 Hz, 3H, CH_3δ-Leu); **^{13}C NMR** (126 MHz, $CDCl_3$) δ_C ppm 172.3 (C=O), 171.7 (C=O), 171.4 (C=O), 169.8 (C=O), 169.5 (C=O), 156.4 (C=O, Cbz), 136.0 (C), 135.4 (C), 128.74 (CH), 128.73 (CH), 128.60 (CH), 128.58 (CH), 128.5 (CH), 128.3 (CH), 82.4 (C, *t*Bu), 67.6 (CH_2, Bn), 67.2 (CH_2, Bn), 53.0 (CH, α-Asp), 51.6 (CH, α-Leu), 43.1 (CH_2, Gly), 41.3 (CH_2, Gly), 40.2 (CH_2, β-Leu), 37.1 (CH_2, β-Asp), 28.1 (CH_3, *t*Bu), 24.9 (CH, γ-Leu), 23.1 (CH_3, δ-Leu), 21.7 (CH_3, δ-Leu); **ν_{max}** (neat) = 3283, 2955, 1736, 1714, 1631, 1522, 1204, 697 cm^{-1}; **MS** (ESI^+) *m/z* 663 $[M+Na]^+$; **HRMS** (ESI^+) calcd. for $C_{33}H_{44}N_4NaO_9$ $[M+Na]^+$ 663.3000, found 663.3006; $[\alpha]_D^{26}$ –16.3 (*c* 0.16, MeOH).

H-Asp(*t*Bu)-Leu-Gly-Gly-OH (47)

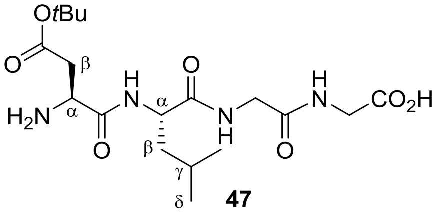

47

To a solution of tetrapeptide Cbz-Asp(*t*Bu)-Leu-Gly-Gly-OBn (**46**) (1.07 g, 1.67 mmol, 1.0 equiv) in MeOH (17 mL) was added 10 wt% Pd/C (106 mg, 10 wt%) and the reaction flask was evacuated, filled with nitrogen, evacuated, and placed under an atmosphere of hydrogen (balloon). The reaction mixture was stirred at room temperature for 16 h, placed under nitrogen and filtered through a plug of Celite, which was washed with MeOH (3×). The filtrate was concentrated *in vacuo* to give H-Asp(*t*Bu)-Leu-Gly-Gly-OH (**47**) as a white solid (692 mg, 1.67 mmol) in quantitative yield. **mp** 172–175 °C; **^{1}H NMR** (500 MHz, CD_3OD) δ_H ppm 4.32 (t, *J* = 7.4 Hz, 1H, CHα-Leu), 4.15 (dd, *J* = 8.3, 4.1 Hz, 1H, CHα-Asp), 3.99 (d, *J* = 16.9 Hz, 1H, C*H*HGly), 3.83–3.75 (m, 3H, CH_2Gly, CH*H*Gly), 3.01 (dd, *J* = 18.0, 4.1 Hz, 1H, C*H*Hβ-Asp), 2.84 (dd, *J* = 18.0, 8.4 Hz, 1H, CH*H*β-Asp), 1.77–1.59 (m, 3H, CH_2β-Leu, CHγ-Leu), 1.48 (s, 9H, 3 × CH_3, *t*Bu), 0.98 (d, *J* = 6.3 Hz, 3H, CH_3δ-Leu), 0.95 (d, *J* = 6.2 Hz, 3H, CH_3δ-Leu); **^{13}C NMR** (126 MHz, CD_3OD) δ_C ppm 175.6 (C=O), 174.7 (C=O), 171.3 (C=O), 171.1 (C=O), 170.9 (C=O), 83.6 (C, *t*Bu), 54.3 (CH, α-Leu), 51.1 (CH, α-Asp), 44.0 (CH_2, Gly), 43.5 (CH_2, Gly), 41.1 (CH_2, β-Leu), 37.5 (CH_2, β-Asp), 28.3 (CH_3, *t*Bu), 25.9 (CH, γ-Leu), 23.3 (CH_3, δ-Leu), 22.0 (CH_3, δ-Leu); **ν_{max}** (neat) = 3279, 2957, 1724, 1630, 1520, 1153, 674 cm^{-1}; **MS** (ESI^+) *m/z* 417 $[M+H]^+$, 439 $[M+Na]^+$; **HRMS** (ESI^+) calcd. for $C_{18}H_{32}N_4NaO_7$ $[M+Na]^+$ 439.2163, found 439.2167; $[\alpha]_D^{26}$ +15.9 (*c* 0.07, MeOH).

Cyclo(Asp(*t*Bu)-Leu-Gly-Gly) (48)

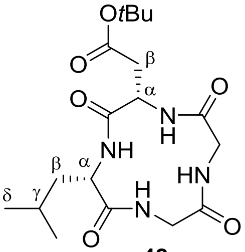

48

To a solution of H-Asp(*t*Bu)-Leu-Gly-Gly-OH (**47**) (208 mg, 0.50 mmol, 1.0 equiv) in anhydrous DMF (500 mL, 0.001 M) under an atmosphere of nitrogen was added DEPBT (299 mg, 1.00 mmol, 2.0 equiv) and DIPEA (169 µL, 1.00 mmol, 2.0 equiv) and the mixture was stirred for 48 h at room temperature. The DMF was removed *in vacuo* and the residue was purified twice by column chromatography (SiO_2, CH_2Cl_2/MeOH 9:1→ 4:1) to give the cyclic tetrapeptide **48** as a white solid (1st run: 27.2 mg, 68 µmol, 14%; 2nd run (0.20 mmol scale): 10.5 mg, 26 µmol, 13%); **R_f** (CH_2Cl_2/MeOH 9:1) 0.24; **mp** 202–205 °C; **^{1}H NMR** (500 MHz, CD_3OD) δ_H ppm 4.63 (dd, *J* = 8.4, 4.4 Hz, 1H, CHα-Asp), 4.36 (dd, *J* = 11.5, 3.6 Hz, 1H, CHα-Leu), 4.04 (d, *J* = 16.7 Hz, 1H, C*H*HGly), 3.90 (d, *J* = 15.8 Hz, 1H, C*H*HGly), 3.73 (d, *J* = 15.8 Hz, 1H, CH*H*Gly), 3.69 (d, *J* = 16.7 Hz, 1H, CH*H*Gly), 2.85 (dd, *J* = 16.7, 4.4 Hz, 1H, C*H*Hβ-Asp), 2.72 (dd, *J* = 16.8, 8.4 Hz, 1H, CH*H*β-Asp), 1.96–1.87 (m, 1H, C*H*Hβ-Leu), 1.81–1.70 (m, 1H, CHγ-Leu), 1.60–1.51 (m, 1H, CH*H*β-Leu), 1.45 (s, 9H, 3 × CH_3, *t*Bu), 0.95 (d, *J* = 6.6 Hz, 3H, CH_3δ-Leu), 0.89 (d, *J* = 6.5 Hz, 3H, CH_3δ-Leu); **^{13}C NMR** (126 MHz, CD_3OD) δ_C ppm 174.5 (C=O), 171.9 (C=O), 171.3 (C=O), 171.2 (C=O), 169.9 (C=O), 81.2 (C, *t*Bu), 52.7 (CH, α-Asp), 50.7 (CH, α-Leu), 43.1 (CH_2, Gly), 41.8 (CH_2, Gly), 40.3 (CH_2, β-Leu), 36.1 (CH_2, β-Asp), 26.9 (CH_3, *t*Bu), 24.5 (CH, γ-Leu), 22.6 (CH_3,

δ-Leu), 20.1 (CH_3, δ-Leu); **ν$_{max}$** (neat) = 3292, 2956, 2930, 1647, 1526, 1151 cm^{-1}; **MS** (ESI$^+$) *m/z* 421 $[M+Na]^+$; **HRMS** (ESI$^+$) calcd. for $C_{18}H_{30}N_4NaO_6$ $[M+Na]^+$ 421.2058, found 421.2061; $[\alpha]_D^{26}$ +15.9 (*c* 0.09, MeOH).

*2.5 Preparation of cyclic tetrapeptide **12** and **58***

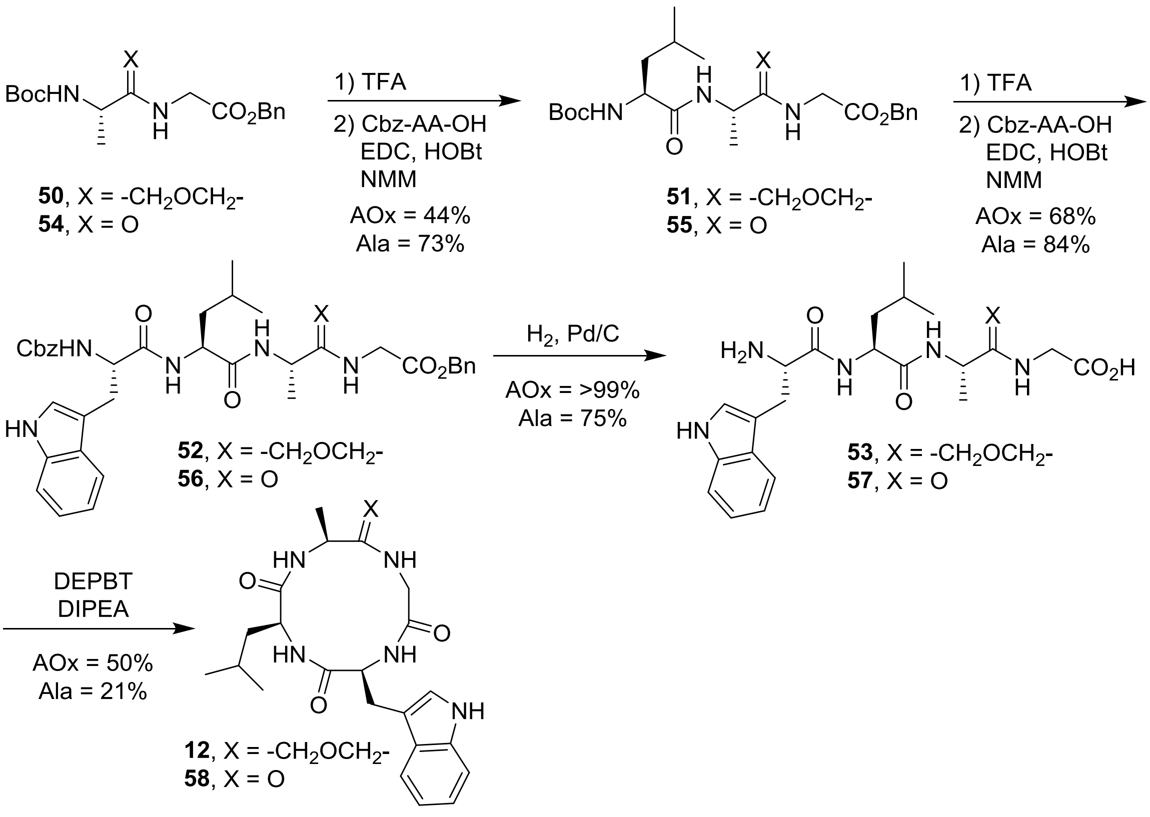

Boc-AOx-(*R*)-CH(Me)Ph (49)

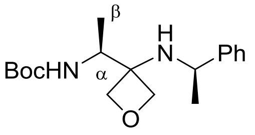

49

To a solution of NO_2-AOx-(*R*)-CH(Me)Ph[4] (1.23 g, 3.83 mmol, 1.0 equiv) in THF (40 mL) was added Boc_2O (1.67 g, 7.65 mmol, 2.0 equiv) and Raney Ni (slurry in H_2O, 8.0 mL). The solution was placed under an atmosphere of nitrogen, evacuated and filled with hydrogen (balloon). The reaction mixture was stirred vigorously for 6 h at room temperature. Then, the mixture was filtered through a plug of Celite eluting with EtOAc, the filtrate was concentrated under reduced pressure, EtOAc (40 mL) was added, the mixture was washed with saturated Na_2CO_3 (3 × 40 mL), dried over Na_2SO_4 and concentrated under reduced pressure. Boc-AOx-(*R*)-CH(Me)Ph (**49**) was afforded after purification by column chromatography (SiO_2, EtOAc/PE 3:7) as a colourless oil (1.06 g, 3.30 mmol, 86%). **R$_f$** (EtOAc /PE 3:7) 0.44; **mp** 100–103 ºC; **^{1}H NMR** (500 MHz, $CDCl_3$) δ_H 7.36–7.29 (m, 4H, ArH), 7.23 (t, *J* = 7.0 Hz, 1H, ArH), 4.82 (s, 1H, NH Boc), 4.51–4.56 (m, 1H, OC*H*H-Ox), 4.41–4.37 (m, 2H, OC*H*H-Ox, OCH*H*-Ox), 4.33–4.28 (m, 1H, OCH*H*-Ox), 4.14 (q, *J* = 6.6 Hz, 1H, C*H*CH_3), 4.10–4.01 (m, 1H, CHα-AOx), 1.47 (s, 3 × 9H, CH_3, Boc), 1.37 (d, *J* = 6.7 Hz, 3H, CHC*H*$_3$), 1.11 (d, *J* = 6.6 Hz, 3H, CH_3β-AOx); **^{13}C NMR** (126 MHz, $CDCl_3$) δ_C 156.0 (C=O, Boc), 147.3 (C), 128.8 (CH), 127.1 (CH), 126.3 (CH), 78.9 (OCH_2), 78.5 (C, Boc), 63.3 (C, Ox), 52.9 (*C*HCH_3), 49.7 (CH, α-AOx), 28.5 (CH_3, Boc), 26.5 (CH*C*H$_3$), 15.3 (CH_3, β-AOx). **ν$_{max}$** (neat) = 3301, 2970, 2879, 1708, 1541, 1167 cm^{-1}; **MS** (ESI$^+$) *m/z* 321 $[M+H]^+$, 343 $[M+Na]^+$; **HRMS** (ESI$^+$) calcd. for $C_{18}H_{29}N_2O_3$ $[M+H]^+$ 321.2173, found 321.2175; $[\alpha]_D^{29}$ +23.6 (*c* 0.18, $CHCl_3$).

Boc-AOx-Gly-OBn (50)

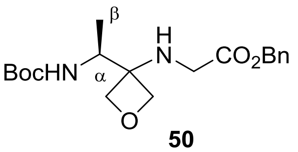

50

To a solution of Boc-AOx-(*R*)-CH(Me)Ph (**49**) (1.62 g, 5.08 mmol, 1.0 equiv) in MeOH (51 mL) was added 31 wt% $Pd(OH)_2/C$ (508 mg, 10 wt%) and the reaction flask was evacuated, filled with nitrogen, evacuated, and placed under an atmosphere of hydrogen (balloon). The reaction mixture was stirred at room temperature for 16 h, placed under nitrogen, and filtered through a plug of Celite, which was washed with MeOH (3×). The filtrate was concentrated *in vacuo* to a pale yellow solid, which was re-dissolved in anhydrous CH_3CN (100 mL) and $BrCH_2CO_2Bn$ (1.68 mL, 10.2 mmol, 2.0 equiv) and DIPEA (1.85 mL, 10.2 mmol, 2.0 equiv) were added. The reaction mixture was stirred at 35 °C under an atmosphere of nitrogen for 3 d, at which time the solvent was removed under reduced pressure. The residue was purified by column chromatography (SiO_2, EtOAc/PE 4:6) to give Boc-AOx-Gly-OBn (**50**) as a yellow solid (1.27 g, 3.50 mmol, 69%). $\mathbf{R_f}$ (EtOAc/PE 4:6) 0.22; **mp** 79–81 ºC; **^{1}H NMR** (500 MHz, $CDCl_3$) δ_H 7.40–7.32 (m, 5H, ArH), 5.19 (s, 2H, CH_2Ph), 4.93 (s, 1H, NH Boc), 4.51–4.37 (m, 4H, 2 × OCH_2-Ox), 4.12 (d, *J* = 6.9 Hz, 1H, CHα-AOx), 3.73 (d, *J* = 17.6 Hz, 1H, C*H*HGly), 3.65 (d, *J* = 17.6 Hz, 1H, CH*H*Gly), 1.94 (s, 1H, NH), 1.44 (s, 9H, 3 × CH_3, Boc), 1.14 (d, *J* = 6.8 Hz, 3H, CH_3β-AOx); **^{13}C NMR** (126 MHz, $CDCl_3$) δ_C 172.7 (C=O), 155.9 (C=O, Boc), 135.4 (C), 128.8 (CH), 128.7 (CH), 128.6 (CH), 78.2 (C, Boc), 77.3 (OCH_2), 67.2 (CH_2, Bn), 62.8 (C, Ox), 50.6 (CH, α-AOx), 45.0 (CH_2, Gly), 28.5 (CH_3, Boc), 14.5 (CH_3, β-AOx); $\mathbf{\nu_{max}}$ (neat) = 3342, 2884, 1746, 1716, 1248 cm^{-1}; **MS** (ESI^+) *m/z* 365 $[M+H]^+$, 387 $[M+Na]^+$; **HRMS** (ESI^+) calcd. for $C_{19}H_{28}N_2NaO_5$ $[M+Na]^+$ 387.1890, found 387.1894; $[\boldsymbol{\alpha}]_D^{29}$ –6.0 (*c* 0.12, MeOH).

Boc-Leu-AOx-Gly-OBn (51)

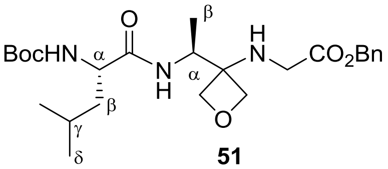

51

To a solution of Boc-AOx-Gly-OBn (**50**) (574 mg, 1.58 mmol, 1.0 equiv) in CH_2Cl_2 (2.0 mL) was added TFA (2.0 mL) and the mixture was stirred at room temperature for 1 h *(Gas evolution!)*. The mixture was concentrated under reduced pressure and the resulting residue repeatedly dissolved in CH_2Cl_2 (3 × 25 mL) and concentrated *in vacuo* to give the crude amine. The residue was dissolved in CH_2Cl_2 (16 mL), Boc-Leu-OH (364 mg, 1.58 mmol, 1.0 equiv), EDC·HCl (302 mg, 1.58 mmol, 1.0 equiv), HOBt·H_2O (213 mg, 1.58 mmol, 1.0 equiv) and NMM (700 µL, 6.30 mmol, 4.0 equiv) were added, and the mixture was stirred at room temperature for 24 h. The reaction mixture was diluted with EtOAc (20 mL) and washed with brine (3 × 25 mL), dried over Na_2SO_4, filtered, concentrated *in vacuo* and purified by column chromatography (SiO_2, CH_2Cl_2/EtOAc 6:4) to give Boc-Leu-AOx-Gly-OBn (**51**) (328 mg, 0.69 mmol, 44%) as a colourless oil. $\mathbf{R_f}$ (CH_2Cl_2/EtOAc 6:4) 0.29; **^{1}H NMR** (500 MHz, $CDCl_3$) δ_H 7.41–7.30 (m, 5H, ArH), 6.64 (d, *J* = 8.3 Hz, 1H, NH), 5.19 (s, 2H, CH_2Ph), 4.91 (s, 1H, Boc NH), 4.52–4.30 (m, 5H, 2 × OCH_2-Ox, CHα-Leu), 4.07–3.97 (m, 1H, CHα-AOx), 3.72 (d, *J* = 17.7 Hz, 1H, C*H*HGly), 3.65 (d, *J* = 17.7 Hz, 1H, CH*H*Gly), 1.92 (s, 1H, NH), 1.68–1.55 (m, 2H, CHγ-Leu, C*H*Hβ-Leu), 1.49–1.38 (m, 10H, CH*H*β-Leu, 3 × CH_3, Boc), 1.14 (d, *J* = 6.8 Hz, 3H, CH_3β-AOx), 0.92 (d, *J* = 4.8 Hz, 6H, 2 × CH_3δ-Leu); **^{13}C NMR** (126 MHz, $CDCl_3$) δ_C 172.8 (C=O), 172.7 (C=O), 155.8 (C=O, Boc) 135.4 (C), 128.8 (CH), 128.7 (CH), 128.6 (CH), 78.2 (C, Boc), 77.4 (OCH_2), 67.3 (CH_2, Bn), 62.5 (C, Ox), 53.5 (CH, α-Leu), 49.7 (CH, α-AOx), 45.0 (CH_2, Gly), 41.4 (CH_2, β-Leu), 28.4 (CH_3, Boc), 25.0 (CH, γ-Leu), 23.0 (CH_3, δ-Leu), 22.2 (CH_3, δ-Leu), 14.5 (CH_3, β-AOx); $\mathbf{\nu_{max}}$ (neat) = 3329, 2975, 1737, 1682, 1649, 1117 cm^{-1}; **MS** (ESI^+) *m/z* 478 $[M+H]^+$, 500 $[M+Na]^+$; **HRMS** (ESI^+) calcd. for $C_{25}H_{40}N_3O_6$ $[M+H]^+$ 478.2912, found 478.2915; $[\boldsymbol{\alpha}]_D^{29}$ –25.0 (*c* 0.20, MeOH).

Cbz-Trp-Leu-AOx-Gly-OBn (52)

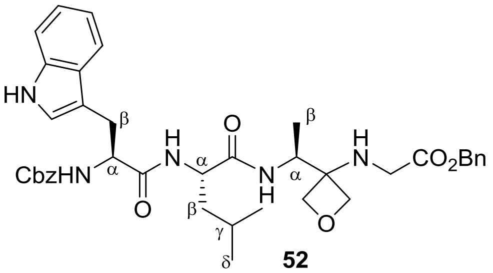

52

To a solution of Boc-Leu-AOx-Gly-OBn (**51**) (460 mg, 0.97 mmol, 1.0 equiv) in CH_2Cl_2 (2.0 mL) was added TFA (2.0 mL) and the mixture was stirred at room temperature for 1 h *(Gas evolution!)*. The mixture was concentrated under reduced pressure and the resulting residue repeatedly dissolved in CH_2Cl_2 (3 × 25 mL) and concentrated *in vacuo* to give the crude amine. The residue was dissolved in CH_2Cl_2 (10 mL), Cbz-Trp-OH (326 mg, 0.97 mmol, 1.0 equiv), EDC·HCl (185 mg, 0.97 mmol, 1.0 equiv), HOBt·H_2O (130 mg, 0.97 mmol, 1.0 equiv) and NMM (477 µL, 4.37 mmol, 4.5 equiv) were added, and the mixture was stirred at room temperature for 24 h. The reaction mixture was diluted with EtOAc (25 mL) and washed with brine (3 × 25 mL), dried over Na_2SO_4, filtered and concentrated *in vacuo*. The residue was purified by column chromatography (SiO_2, CH_2Cl_2/MeOH 19:1) to give Cbz-Trp-Leu-AOx-Gly-OBn (**52**) (456 mg, 0.65 mmol, 68%) as a white solid. $\mathbf{R_f}$ (CH_2Cl_2/MeOH 19:1) 0.20; **mp** 81–84 °C; ^{1}**H NMR** (500 MHz, $CDCl_3$) δ_H 8.30 (s, 1H, NH), 7.65 (d, *J* = 7.1 Hz, 1H, ArH), 7.39–7.27 (m, 11H, ArH), 7.19 (t, *J* = 7.5 Hz, 1H, ArH), 7.11 (t, *J* = 6.8 Hz, 1H, ArH), 7.04 (s, 1H, ArH), 6.72 (d, *J* = 8.0 Hz, 1H, NH), 6.25 (d, *J* = 6.7 Hz, 1H, NH), 5.48 (d, *J* = 6.9 Hz, 1H, NH), 5.17 (s, *J* = 5.8 Hz, 2H, CH_2Ph), 5.12–5.04 (m, 2H, CH_2Ph), 4.56–4.24 (m, 7H, 2 × OCH_2-Ox, CHα-Leu, CHα-Ala, CHα-Trp), 3.67 (s, 2H, CH_2Gly), 3.28 (dd, *J* = 14.6, 5.7 Hz, 1H, C*H*Hβ-Trp), 3.20 (dd, *J* = 14.6, 6.7 Hz, 1H, CH*H*β-Trp), 1.97 (s, 1H, NH), 1.61–1.51 (m, 1H, C*H*Hβ-Leu), 1.42–1.28 (m, 2H, CH*H*β-Leu, CHγ-Leu), 1.09 (d, *J* = 6.6 Hz, 3H, CH_3β-AOx), 0.81 (d, *J* = 6.4 Hz, 6H, 2 × CH_3δ-Leu); ^{13}**C NMR** (126 MHz, $CDCl_3$) δ_C 172.9 (C=O), 171.7 (C=O), 171.5 (C=O), 156.4 (C=O, Cbz), 136.4 (C), 136.1 (C), 135.4 (C), 128.8 (CH), 128.72 (CH), 128.67 (CH), 128.5 (CH), 128.4 (CH), 128.3 (CH), 127.4 (C), 123.5 (CH), 122.5 (CH), 120.0 (CH), 118.9 (CH), 111.5 (CH), 110.2 (C), 78.1 (OCH_2), 77.5 (OCH_2), 67.3 (CH_2, Bn), 67.2 (CH_2, Bn), 62.6 (C, Ox), 55.6 (CH, α-Trp), 52.5 (CH, α-Leu), 49.5 (CH, α-AOx), 45.0 (CH_2, Gly), 40.8 (CH_2, β-Leu), 28.1 (CH_2, β-Trp), 24.9 (CH, γ-Leu), 22.9 (CH_3, δ-Leu), 22.1 (CH_3, δ-Leu), 14.5 (CH_3, β-AOx); ν_{max} (neat) = 3282, 2952, 1703, 1643, 1342 cm^{-1}; **MS** (ESI$^+$) *m/z* 698 [M+H]$^+$, 720 [M+Na]$^+$; **HRMS** (ESI$^+$) calcd. for $C_{39}H_{47}N_5NaO_7$ [M+Na]$^+$ 720.3368, found 720.3369; $[\alpha]_D^{29}$ –29.0 (*c* 0.05, MeOH).

H-Trp-Leu-AOx-Gly-OH (53)

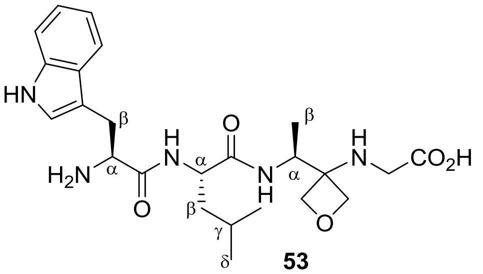

53

To a solution of Cbz-Trp-Leu-AOx-Gly-OBn (**52**) (404 mg, 0.58 mmol, 1.0 equiv) in MeOH (6.0 mL) was added 10 wt% Pd/C (40 mg, 10 wt%) and the reaction flask was evacuated, filled with nitrogen, evacuated, and placed under an atmosphere of hydrogen (balloon). The reaction mixture was stirred at room temperature for 16 h, placed under nitrogen and filtered through a plug of Celite, which was washed with MeOH (3×). The filtrate was concentrated *in vacuo* to give H-Trp-Leu-AOx-Gly-OH (**53**) as a yellow solid (273 mg, 0.58 mmol, quant. yield), which required no further purification. **mp** 170–173 °C; ^{1}**H NMR** (500 MHz, CD_3OD) δ_H 7.73 (d, *J* = 7.9 Hz, 1H, ArH), 7.41 (d, *J* = 8.1 Hz, 1H, ArH), 7.26 (s, 1H, ArH), 7.16 (t, *J* = 7.5 Hz, 1H, ArH), 7.09 (t, *J* = 7.4 Hz, 1H, ArH), 4.62–4.57 (m, 2H, OCH_2-Ox), 4.56–4.50 (m, 2H, OCH_2-Ox), 4.46–4.37 (m, 2H, CHα-Leu, CHα-AOx), 4.24 (dd, *J* = 8.3, 5.6 Hz, 1H, CHα-Trp), 3.63 (d, *J* = 16.9 Hz, 1H, C*H*HGly), 3.58 (d, *J* = 16.9 Hz, 1H, CH*H*Gly), 3.51 (dd, *J* = 15.0, 5.3 Hz, 1H, C*H*Hβ-Trp), 3.23 (dd, *J* = 15.0, 8.5 Hz, 1H, CH*H*β-Trp), 1.71–1.60 (m, 3H, CH_2β-Leu, CHγ-Leu), 1.28 (d, *J* = 6.8 Hz, 3H, CH_3β-AOx), 0.97 (d, *J* = 5.7 Hz, 3H, CH_3δ-Leu), 0.95 (d, *J* = 5.7 Hz, 3H, CH_3δ-Leu); ^{13}**C NMR** (126 MHz, CD_3OD) δ_C 174.9 (C=O),

174.7 (C=O), 170.3 (C=O), 138.3 (C), 128.4 (C), 125.8 (CH), 123.0 (CH), 120.3 (CH), 119.2 (CH), 112.6 (CH), 107.9 (C), 77.5 (OCH_2), 77.4 (OCH_2), 64.6 (C, Ox), 54.8 (CH, α-Trp), 54.0 (CH, α-Leu), 49.9 (CHα-AOx), 46.0 (CH_2, Gly), 42.0 (CH_2, β-Leu), 28.7 (CH_2, β-Trp), 25.9 (CH, γ-Leu), 23.4 (CH_3, δ-Leu), 22.0 (CH_3, δ-Leu), 14.7 (CH_3, β-AOx); **ν_{max}** (neat) = 3228, 2954, 1643, 1537, 1237 cm^{-1}; **MS** (ESI^+) *m/z* 474 $[M+H]^+$, 496 $[M+Na]^+$; **HRMS** (ESI^+) calcd. for $C_{24}H_{35}N_5NaO_5$ $[M+Na]^+$ 496.2530, found 496.2533; $[\alpha]_D^{29}$ –37.0 (*c* 0.05, MeOH).

Cyclo(Trp-Leu-AOx-Gly) (12)

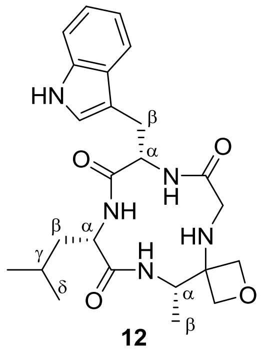

12

To a solution of H-Trp-Leu-AOx-Gly-OH (**53**) (47 mg, 0.10 mmol, 1.0 equiv) in anhydrous DMF (100 mL, 0.001 M) under an atmosphere of nitrogen was added DEPBT (60 mg, 0.20 mmol, 2.0 equiv) and DIPEA (35 µL, 0.20 mmol, 2.0 equiv) and the mixture was stirred for 48 h at room temperature. The solvent was removed under reduced pressure, and the residue was purified twice by column chromatography (SiO_2, CH_2Cl_2/MeOH 9:1→85:15) to give cyclic tetrapeptide **12** as a white solid (1st run: 22.9 mg, 50 µmol, 50%; 2nd run (289 µmol scale): 64.4 mg, 142 µmol, 49%). **R_f** (CH_2Cl_2/MeOH 9:1) 0.19; **mp** 220–223 °C; **^{1}H NMR** (500 MHz, CD_3OD) δ_H 7.62 (d, *J* = 7.9 Hz, 1H, ArH), 7.32 (d, *J* = 8.1 Hz, 1H, ArH), 7.12 (s, 1H, ArH), 7.10 (t, *J* = 7.7 Hz, 1H, ArH), 7.03 (t, *J* = 7.4 Hz, 1H, ArH), 4.93 (dd, *J* = 10.2, 7.2 Hz, 1H, CHα-Trp), 4.57 (q, *J* = 7.2 Hz, 1H, CHα-AOx), 4.50 (d, *J* = 6.8 Hz, 1H, OC*H*H-Ox), 4.40 (d, *J* = 6.8 Hz, 1H, OCH*H*-Ox), 4.38 (d, *J* = 7.8 Hz, 1H, OC*H*H-Ox), 4.20 (dd, *J* = 10.9, 4.2 Hz, 1H, CHα-Leu), 4.04 (d, *J* = 7.8 Hz, 1H, OCH*H*-Ox), 3.67 (d, *J* = 16.6 Hz, 1H, C*H*HGly), 3.57 (d, *J* = 16.6 Hz, 1H, CH*H*Gly), 3.37–3.27 (m, 1H, C*H*Hβ-Trp), 3.21 (dd, *J* = 15.1, 7.2 Hz, 1H, CH*H*β-Trp), 1.72–1.63 (m, 1H, C*H*Hβ-Leu), 1.59–1.48 (m, 2H, CH*H*β-Leu, CHγ-Leu), 1.12 (d, *J* = 6.9 Hz, 3H, CH_3β-AOx), 0.90 (d, *J* = 6.0 Hz, 3H, CH_3δ-Leu), 0.73 (d, *J* = 6.0 Hz, 3H, CH_3δ-Leu). *N.B.* C*H*Hβ-Trp overlaps with solvent peak; **^{13}C NMR** (126 MHz, CD_3OD) δ_C 176.4 (C=O), 175.3 (C=O), 173.7 (C=O), 138.0 (C), 128.5 (C), 123.8 (CH), 122.6 (CH), 119.9 (CH), 119.1 (CH), 112.3 (CH), 110.3 (C), 79.2 (OCH_2), 77.3 (OCH_2), 64.7 (C, Ox), 57.7 (CH, α-Trp), 55.3 (CH, α-Leu), 51.6 (CH, α-AOx), 48.8 (CH_2, Gly), 40.6 (CH_2, β-Leu), 27.6 (CH_2, β-Trp), 26.2 (CH, γ-Leu), 23.3 (CH_3, δ-Leu), 21.2 (CH_3, δ-Leu), 13.5 (CH_3, AOx). *N.B.* CH_2, Gly signal overlaps with solvent peak; **ν_{max}** (neat) = 3256, 2956, 1659, 1532, 740 cm^{-1}; **MS** (ESI^+) *m/z* 478 $[M+Na]^+$; **HRMS** (ESI^+) calcd. for $C_{24}H_{33}N_5NaO_4$ $[M+Na]^+$ 478.2425, found 478.2423; $[\alpha]_D^{29}$ –108 (*c* 0.06, MeOH).

Boc-Leu-Ala-Gly-OBn (55)

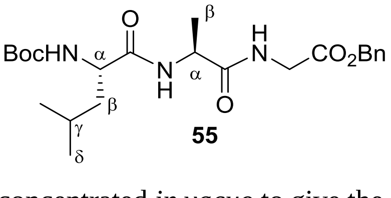

To a solution of Boc-Ala-Gly-OBn[5] (**54**) (885 mg, 2.63 mmol, 1.0 equiv) in CH_2Cl_2 (3.0 mL) was added TFA (3.0 mL) and the mixture was stirred at room temperature for 1 h *(Gas evolution!)*. The mixture was concentrated under reduced pressure and the resulting residue repeatedly dissolved in CH_2Cl_2 (3 × 25 mL) and concentrated *in vacuo* to give the crude amine. The residue was dissolved in CH_2Cl_2 (27 mL), Boc-Leu-OH (608 mg, 2.63 mmol, 1.0 equiv), EDC·HCl (504 mg, 2.63 mmol, 1.0 equiv), HOBt·H_2O (355 mg, 2.63 mmol, 1.0 equiv) and NMM (1.20 mL, 10.5 mmol, 4.0 equiv) were added, and the mixture was stirred at room temperature for 24 h. The mixture was diluted with EtOAc (30 mL) and washed with brine (3 × 30 mL), dried over Na_2SO_4, filtered and concentrated *in vacuo*. The residue was purified by column chromatography (SiO_2, EtOAc/CH_2Cl_2 4:6) to give Boc-Leu-Ala-Gly-OBn (**55**) as a white foam (863 mg, 1.92 mmol, 73%). **R_f** (EtOAc/CH_2Cl_2 4:6) 0.29; **mp** 60–63 °C; **^{1}H NMR** (500 MHz, $CDCl_3$) δ_H 7.38–7.30 (m, 5H, ArH), 6.97 (s, 1H, NH), 6.71 (d, *J* = 7.4 Hz, 1H, NH), 5.16 (s, 2H, CH_2Ph), 4.96

(d, *J* = 6.7 Hz, 1H, Boc NH), 4.54 (quint, *J* = 7.1 Hz, 1H, CHα-Ala), 4.15–3.98 (m, 3H, CHα-Leu, CH_2Gly), 1.71–1.59 (m, 2H, CHγ-Leu, C*H*Hβ-Leu), 1.52–1.44 (m, 1H, CH*H*β-Leu), 1.42 (s, 9H, 3 × CH_3, Boc), 1.39 (d, *J* = 7.1 Hz, 3H, CH_3β-Ala), 0.93 (d, *J* = 6.5 Hz, 6H, 2 × CH_3δ-Leu); **^{13}C NMR** (126 MHz, $CDCl_3$) δ_C 172.8 (C=O), 172.5 (C=O), 169.6 (C=O), 156.1 (C=O, Boc), 135.3 (C), 128.8 (CH), 128.7 (CH), 128.5 (CH), 80.6 (C, Boc), 67.3 (CH_2, Bn), 53.5 (CH, α-Leu), 48.8 (CH, α-Ala), 41.5 (CH_2, Gly), 41.1 (CH_2, β-Leu), 28.4 (CH_3, Boc), 24.9 (CH, γ-Leu), 23.1 (CH_3, δ-Leu), 21.9 (CH_3, δ-Leu), 18.0 (CH_3, β-Ala); **ν_{max}** (neat) = 3297, 2932, 1748, 1645, 1453, 1162 cm^{-1}; **MS** (ESI^+) *m/z* 472 $[M+Na]^+$; **HRMS** (ESI^+) calcd. for $C_{23}H_{35}N_3NaO_6$ $[M+Na]^+$ 472.2418, found 472.2420; $[\alpha]_D^{29}$ –45.0 (*c* 0.15, MeOH).

Cbz-Trp-Leu-Ala-Gly-OBn (56)

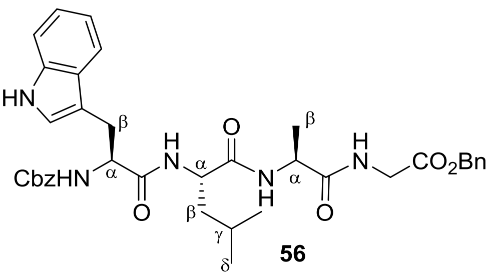

56

To a solution of Boc-Leu-Ala-Gly-OBn (**55**) (760 mg, 1.69 mmol, 1.0 equiv) in CH_2Cl_2 (2.5 mL) was added TFA (2.5 mL) and the mixture was stirred at room temperature for 1 h *(Gas evolution!)*. The mixture was concentrated under reduced pressure and the resulting residue repeatedly dissolved in CH_2Cl_2 (3 × 25 mL) and concentrated *in vacuo* to give the crude amine. The residue was dissolved in CH_2Cl_2 (22 mL), Cbz-Trp-OH (572 mg, 1.69 mmol, 1.0 equiv), EDC·HCl (324 mg, 1.69 mmol, 1.0 equiv), HOBt·H_2O (228 mg, 1.69 mmol, 1.0 equiv) and NMM (750 µL, 6.76 mmol, 4.0 equiv) were added, and the mixture was stirred at room temperature for 24 h. The mixture was diluted with EtOAc (25 mL) and washed with brine (3 × 25 mL), dried over Na_2SO_4, filtered and concentrated *in vacuo*. The residue was purified by column chromatography (SiO_2, CH_2Cl_2/MeOH 24:1) to give Cbz-Trp-Leu-Ala-Gly-OBn (**56**) (945 mg, 1.41 mmol, 84%) as a white solid. **R_f** (CH_2Cl_2/MeOH 24:1) 0.41; **mp** 93–96 ºC; **^{1}H NMR** (500 MHz, CD_3OD) δ_H 7.59 (d, *J* = 7.9 Hz, 1H, ArH), 7.37–7.23 (m, 11H, ArH), 7.13–7.05 (m, 2H, ArH), 6.99 (t, *J* = 7.4 Hz, 1H, ArH), 5.13 (s, 2H, CH_2Ph), 5.02 (d, *J* = 5.3 Hz, 2H, CH_2Ph), 4.49–4.43 (m, 1H, CHα-Trp), 4.37–4.30 (m, 2H, CHα-Ala, CHα-Leu), 4.01 (d, *J* = 17.5 Hz, 1H, C*H*HGly), 3.94 (d, *J* = 17.5 Hz, 1H, CH*H*Gly), 3.27 (dd, *J* = 14.6, 5.6 Hz, 1H, C*H*Hβ-Trp), 3.11 (dd, *J* = 14.6, 7.9 Hz, 1H, CH*H*β-Trp), 1.57–1.46 (m, 3H, CHγ-Leu, CH_2β-Leu), 1.31 (d, *J* = 7.2 Hz, 3H, CH_3β-Ala), 0.87 (d, *J* = 6.0 Hz, 3H, CH_3δ-Leu), 0.82 (d, *J* = 6.0 Hz, 3H, CH_3δ-Leu); **^{13}C NMR** (126 MHz, CD_3OD) δ_C 175.3 (C=O), 175.0 (C=O), 174.4 (C=O), 170.9 (C=O), 158.5 (C=O, Cbz), 138.1 (C), 138.0 (C), 137.1 (C), 129.53 (CH), 129.45 (CH), 129.3 (CH), 129.0 (CH), 128.8 (CH), 124.7 (CH), 122.5 (CH), 119.9 (CH), 119.4 (CH), 112.3 (CH), 110.7 (C), 67.9 (CH_2, Bn), 67.7 (CH_2, Bn), 57.5 (CH, α-Trp), 53.4 (CH, α-Leu), 50.3 (CH, α-Ala), 42.1 (CH_2, Gly), 41.4 (CH_2, β-Leu), 28.7 (CH_2, β-Trp), 25.6 (CH, γ-Leu), 23.5 (CH_3, δ-Leu), 21.9 (CH_3, δ-Leu), 17.9 (CH_3, β-Ala). *N.B.* One quaternary aromatic C and one CH not observed; **ν_{max}** (neat) = 3270, 2952, 1703, 1634, 1191 cm^{-1}; **MS** (ESI^+) *m/z* 692 $[M+Na]^+$; **HRMS** (ESI^+) calcd. for $C_{37}H_{43}N_5NaO_7$ $[M+Na]^+$ 692.3055, found 692.3061; $[\alpha]_D^{29}$ –37.0 (*c* 0.17, MeOH).

H-Trp-Leu-Ala-Gly-OH (57)

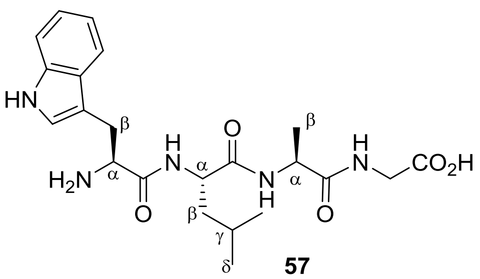

57

To a solution of Cbz-Trp-Leu-Ala-Gly-OBn (**56**) (854 mg, 1.28 mmol, 1.0 equiv) in MeOH (13 mL) was added 10 wt% Pd/C (86 mg, 10 wt%) and the reaction flask was evacuated, filled with nitrogen, evacuated, and placed under an atmosphere of hydrogen (balloon). The reaction mixture was stirred at room temperature for 16 h, placed under nitrogen, and DMF (10 mL) was added. The solution was filtered through a plug of Celite and the solids were washed with MeOH (3×). The filtrate was concentrated *in vacuo* to give H-Trp-Leu-Ala-Gly-OH (**57**) as a DMF adduct as a white solid (426 mg, 0.96 mmol, 75%), which required no further purification. **mp** 198–201 ºC; **^{1}H NMR** (500 MHz, D_2O) δ_H 7.59 (d, *J* = 8.0 Hz, 1H, ArH), 7.52 (d, *J* = 8.2 Hz, 1H, ArH), 7.31–7.25 (m, 2H, ArH), 7.16 (t, *J* = 7.4 Hz, 1H, ArH), 4.32–4.26 (m, 2H, CHα-Trp, CHα-Leu), 4.15 (q, *J* = 7.2 Hz, 1H, CHα-Ala), 3.75 (s, 2H, CH_2Gly), 3.44–3.33 (m, 2H, CH_2β-Trp), 1.53–1.47 (m, 3H, CHγ-Leu, CH_2β-Leu), 1.37 (d, *J* = 7.2 Hz, 3H, CH_3β-Ala), 0.87 (t, *J* = 5.8 Hz, 3H, CH_3δ-Leu), 0.85 (d, *J* = 5.8 Hz, 3H, CH_3δ-Leu); **^{13}C NMR** (126 MHz, D_2O) δ_C 176.0 (C=O), 174.2 (C=O), 172.8 (C=O), 169.0 (C=O), 136.2 (C), 126.6 (C), 125.1 (CH), 122.1 (CH), 119.4 (CH), 117.9 (CH), 111.9 (CH), 106.2 (C), 53.5 (CH, α-Trp), 52.0 (CH, α-Leu), 49.6 (CH, α-Ala), 43.1 (CH_2, Gly), 40.5 (CH_2, β-Leu), 26.7 (CH_2, β-Trp), 24.0 (CH, γ-Leu), 21.9 (CH_3, δ-Leu), 21.1 (CH_3, δ-Leu), 16.5 (CH_3, β-Ala); **ν_{max}** (neat) = 3270, 2956, 1626, 1525, 1438 cm^{-1}; **MS** (ESI^+) *m/z* 468 $[M+Na]^+$; **HRMS** (ESI^+) calcd. for $C_{22}H_{31}N_5NaO_5$ $[M+Na]^+$ 468.2217, found 468.2220; $[\alpha]_D^{29}$ –23.0 (*c* 0.04, DMF).

Cyclo(Trp-Leu-Ala-Gly) (58)

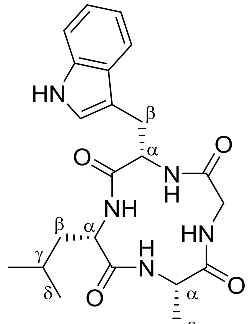

58

To a solution of H-Trp-Leu-Ala-Gly-OH (**57**) (89 mg, 0.20 mmol, 1.0 equiv) in anhydrous DMF (200 mL, 0.001 M) under an atmosphere of nitrogen was added DEPBT (120 mg, 0.40 mmol, 2.0 equiv) and DIPEA (70 µL, 0.40 mmol, 2.0 equiv) and the mixture was stirred for 48 h at room temperature. The solvent was removed under reduced pressure, and the residue was purified twice by column chromatography (SiO_2, CH_2Cl_2/MeOH 19:1→9:1) to give cyclic tetrapeptide **58** as a white solid (1st run: 17.4 mg, 40 µmol, 20%; 2nd run: 18.4 mg, 43 µmol, 22%). **R_f** (CH_2Cl_2/MeOH 92.5:7.5) 0.34; **mp** 296–297 ºC (decomposition); The data reported for this compound are for the major conformational isomer at 298 K. **^{1}H NMR** (500 MHz, DMSO-*d6*) δ_H 10.88 (s, 1H, NH), 8.29 (t, *J* = 5.6 Hz, 1H, NH), 7.80 (d, *J* = 9.4 Hz, 1H, NH), 7.73 (d, *J* = 9.1 Hz, 1H, NH), 7.54 (d, *J* = 7.9 Hz, 1H, ArH), 7.40 (d, *J* = 9.3 Hz, 1H, NH), 7.34 (d, *J* = 8.0 Hz, 1H, ArH), 7.13 (s, 1H, ArH), 7.08 (t, *J* = 7.4 Hz, 1H, ArH), 7.00 (t, *J* = 7.4 Hz, 1H, ArH), 4.52 (m, 1H, CHα-Trp), 4.32–4.25 (m, 1H, CHα-Ala), 4.12 (dd, *J* = 15.6, 9.4 Hz, 1H, CHα-Leu), 3.85 (dd, *J* = 14.0, 6.0 Hz, 1H, C*H*HGly), 3.43 (dd, *J* = 14.0, 6.0 Hz, 1H, CH*H*Gly), 3.13 (dd, *J* = 14.8, 8.2 Hz, 1H, C*H*Hβ-Trp), 3.03 (dd, *J* = 14.8, 8.2 Hz, 1H, CH*H*β-Trp), 1.63–1.44 (m, 3H, CHγ-Leu, CH_2β-Leu), 1.26 (d, *J* = 7.1 Hz, 3H, CH_3β-Ala), 0.89 (d, *J* = 6.1 Hz, 3H, CH_3δ-Leu), 0.80 (d, *J* = 6.1 Hz, 3H, CH_3δ-Leu); **^{13}C NMR** (126 MHz, DMSO-*d6*) δ_C 172.8 (C=O), 172.5 (C=O), 171.6 (C=O), 169.7 (C=O), 136.1 (C), 127.0 (C), 123.1 (CH), 121.0 (CH), 118.4 (CH), 118.1 (CH), 111.4 (CH), 109.4 (C), 56.2 (CH, α-Trp), 54.0 (CH, α-Leu), 49.6 (CH, α-Ala), 43.6 (CH_2, Gly), 39.0 (CH_2, β-Leu) 26.5 (CH_2, β-Trp), 24.6 (CH, γ-Leu), 22.6 (CH_3, δ-Leu), 21.6 (CH_3, δ-Leu), 16.2 (CH_3, β-Ala); **ν_{max}** (neat) = 3291, 2956, 1649, 1529, 737 cm^{-1}; **MS** (ESI^+) *m/z* 450 $[M+Na]^+$, 877 $[2M+Na]^+$; **HRMS** (ESI^+) calcd. for $C_{22}H_{29}N_5NaO_4$ $[M+Na]^+$ 450.2112, found 450.2115; $[\alpha]_D^{29}$ –112 (*c* 0.04, DMF).

*2.6 Preparation of cyclic pentapeptide **13***

*Preparation of pentapeptide **63***

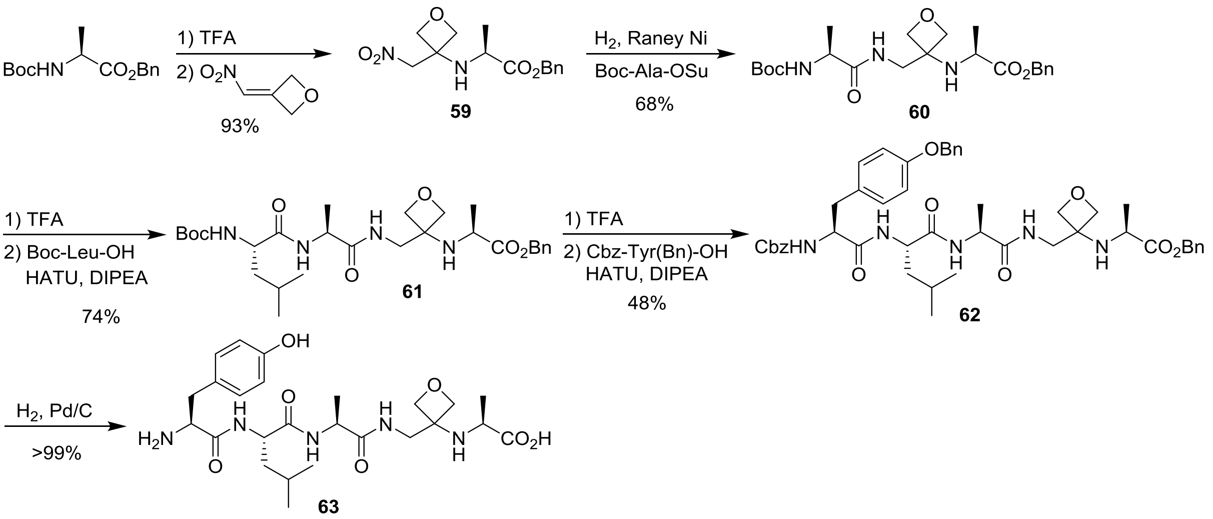

NO_2-GOx-Ala-OBn (59)

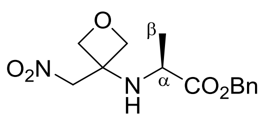

59

To a solution of Boc-Ala-OBn[1] (3.36 g, 12.0 mmol, 1.0 equiv) in CH_2Cl_2 (12 mL) was added TFA (12 mL) and the mixture was stirred at room temperature for 1 h. The mixture was concentrated under reduced pressure and the resulting residue repeatedly dissolved in CH_2Cl_2 (3 × 20 mL) and concentrated *in vacuo* to give the crude amine. In a second reaction vessel, oxetane-3-one (1.54 mL, 24.0 mmol, 2.0 equiv), nitromethane (1.82 mL, 33.6 mmol, 2.8 equiv) and triethylamine (670 µL, 4.80 mmol, 0.4 equiv) were combined at 0 °C and stirred for 1 h at room temperature. The mixture was dissolved in anhydrous CH_2Cl_2 (40 mL), cooled to –78 °C, and triethylamine (6.70 mL, 48.0 mmol, 4.0 equiv) was added followed by dropwise addition of a solution of methanesulfonyl chloride (1.86 mL, 24.0 mmol, 2.0 equiv) in anhydrous CH_2Cl_2 (12 mL). The reaction mixture was stirred at –78 °C for 1.5 h and a solution of the crude amine and triethylamine (2.52 mL, 18.0 mmol, 1.5 equiv) in anhydrous CH_2Cl_2 (40 mL) was added slowly *via* syringe. The reaction mixture was allowed to warm to room temperature and stirred for 16 h. A saturated solution of NH_4Cl (50 mL) was added and stirred for 10 min. The layers were separated and the aqueous one extracted with CH_2Cl_2 (2 × 30 mL) and EtOAc (2 × 30 mL). The combined organic phases were washed with sat. $NaHCO_3$ solution (50 mL), brine (50 mL), dried over $MgSO_4$, filtered and concentrated *in vacuo*. The residue was purified by column chromatography (SiO_2, PE/EtOAc 2:1→1:1) to give **59** (3.28 g, 11.1 mmol, 93%) as an orange oil. **R_f** (PE/EtOAc 1:1) 0.40; **^{1}H NMR** (400 MHz, $CDCl_3$) δ_H ppm 7.41–7.26 (m, 5H, ArH), 5.16 (d, *J* = 12.1 Hz, 1H, C*H*HPh), 5.12 (d, *J* = 12.1 Hz, 1H, CH*H*Ph), 4.83 (d, *J* = 12.8 Hz, 1H, C*H*HGOx), 4.78 (d, *J* = 12.8 Hz, 1H, CH*H*GOx), 4.59 (d, *J* = 7.2 Hz, 1H, OC*H*H-Ox), 4.55 (d, *J* = 7.1 Hz, 1H, OC*H*H-Ox), 4.49 (d, *J* = 7.2 Hz, 1H, OCH*H*-Ox), 4.31 (d, *J* = 7.1 Hz, 1H, OCH*H*-Ox), 3.62 (q, *J* = 7.0 Hz, 1H, CHα-Ala), 2.36 (br. s, 1H, NH), 1.32 (d, *J* = 7.0 Hz, 3H, CH_3β-Ala); **^{13}C NMR** (101 MHz, $CDCl_3$) δ_C ppm 175.6 (C=O), 135.3 (C), 128.83 (CH), 128.78 (CH), 128.6 (CH), 79.2 (OCH_2), 79.1 (OCH_2), 78.3 (CH_2, GOx), 67.3 (CH_2, Bn), 59.7 (C, Ox), 51.5 (CH, α-Ala), 20.4 (CH_3, β-Ala); **ν_{max}** (neat) = 3330, 2967, 1729, 1552, 1454, 1378, 1165, 1050, 975, 902, 738, 698 cm^{-1}; **MS** (ESI^+) *m/z* 295 $[M+H]^+$, 317 $[M+Na]^+$; **HRMS** (ESI^+) calcd. for $C_{14}H_{18}N_2NaO_5$ $[M+Na]^+$ 317.1108, found 317.1107; $[\alpha]_D^{26}$ –1.19 (*c* 1.22, $CHCl_3$).

Boc-Ala-GOx-Ala-OBn (60)

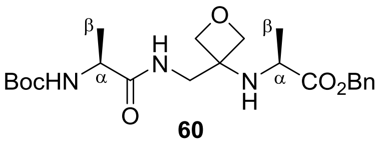

60

To a solution of NO_2-GOx-Ala-OBn (**59**) (3.18 g, 10.8 mmol, 1.0 equiv) in THF (108 mL) was added Boc-Ala-OSu (6.18 g, 21.6 mmol, 2.0 equiv), $NaHCO_3$ (3.63 g, 43.2 mmol, 4.0 equiv) and Raney Ni (slurry in H_2O, 22 mL). The solution was placed under an atmosphere of nitrogen, evacuated and filled with hydrogen (balloon). The reaction mixture was stirred vigorously for 4.0 h at room temperature. Then, the mixture was filtered through a plug of Celite eluting with EtOAc, the filtrate was washed with saturated Na_2CO_3 (3 × 50 mL) and concentrated *in vacuo*. Boc-Ala-GOx-Ala-OBn (**60**) was afforded after purification by column chromatography (SiO_2, PE/EtOAc 1:1→EtOAc) as a pale-yellow oil (3.20 g, 7.34 mmol, 68%). **R_f** (EtOAc) 0.35; **^{1}H NMR** (400 MHz, $CDCl_3$) δ_H ppm 7.38–7.30 (m, 5H, ArH), 6.67 (br. s, 1H, NH), 5.12 (s, 2H, CH_2Ph), 5.08 (br. s, 1H, NH), 4.39 (d, J = 6.5 Hz, 1H, OC*H*H-Ox), 4.34 (d, J = 6.6 Hz, 1H, OC*H*H-Ox), 4.27 (d, J = 6.6 Hz, 1H, OCH*H*-Ox), 4.22 (d, J = 6.5 Hz, 1H, OCH*H*-Ox), 4.16–4.08 (m, 1H, CHα-Ala), 3.75 (dd, J = 14.0, 5.8 Hz, 1H, C*H*HGOx), 3.48 (q, J = 6.8 Hz, 1H, CHα-Ala), 3.42 (dd, J = 14.0, 4.7 Hz, 1H, CH*H*GOx), 2.12 (br. s, 1H, NH), 1.42 (s, 9H, 3 × CH_3, Boc), 1.33 (d, J = 7.1 Hz, 3H, CH_3β-Ala), 1.32 (d, J = 6.8 Hz, 3H, CH_3β-Ala); **^{13}C NMR** (101 MHz, $CDCl_3$) δ_C ppm 176.3 (C=O), 173.4 (C=O), 155.4 (C=O, Boc), 135.3 (C), 128.7 (CH), 128.6 (CH), 128.4 (CH), 80.1 (OCH_2), 80.0 (C, Boc), 79.7 (OCH_2), 67.2 (CH_2, Bn), 59.4 (C, Ox), 51.2 (CH, α-Ala), 50.2 (CH, α-Ala), 43.3 (CH_2, GOx), 28.3 (CH_3, Boc), 20.6 (CH_3, β-Ala), 18.4 (CH_3, β-Ala); **ν_{max}** (neat) = 3309, 2967, 1663, 1499, 1453, 1366, 1247, 1162, 1057, 972, 733, 698 cm^{-1}; **MS** (ESI$^+$) *m/z* 436 $[M+H]^+$, 458 $[M+Na]^+$; **HRMS** (ESI$^+$) calcd. for $C_{22}H_{33}N_3NaO_6$ $[M+Na]^+$ 458.2262, found 458.2263; $[\alpha]_D^{27}$ –10.5 (c 1.00, $CHCl_3$).

Boc-Leu-Ala-GOx-Ala-OBn (61)

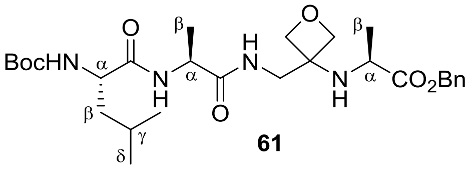

To a solution of Boc-Ala-GOx-Ala-OBn (**60**) (915 mg, 2.10 mmol, 1.0 equiv) in CH_2Cl_2 (2.5 mL) was added TFA (2.5 mL) and the mixture was stirred at room temperature for 1 h. The reaction mixture was concentrated under reduced pressure and the resulting residue repeatedly dissolved in CH_2Cl_2 (3 × 10 mL) and concentrated under reduced pressure to give the crude amine. The residue was dissolved in CH_2Cl_2 (21 mL), Boc-Leu-OH (583 mg, 2.50 mmol, 1.2 equiv), HATU (958 mg, 2.50 mmol, 1.2 equiv) and DIPEA (1.46 mL, 8.40 mmol, 4.0 equiv) were added subsequently, and the reaction mixture was stirred at room temperature for 16 h. The reaction mixture was diluted with CH_2Cl_2 (30 mL) and washed with 10% citric acid solution (2 × 50 mL) and saturated $NaHCO_3$ solution (2 × 50 mL), dried over $MgSO_4$, filtered and concentrated under reduced pressure. The residue was purified by flash column chromatography (SiO_2, EtOAc) to give tetrapeptide **61** (855 mg, 1.56 mmol, 74%) as an off-white solid. **R_f** (EtOAc) 0.30; **mp** 134–136 °C; **^{1}H NMR** (400 MHz, $CDCl_3$) δ_H ppm 7.42–7.28 (m, 5H, ArH), 6.72 (d, J = 7.1 Hz, 2H, NH), 5.13 (s, 2H, CH_2Ph), 4.92 (br. d, J = 7.2 Hz, 1H, NH), 4.45–4.41 (m, 1H, CHα-Ala), 4.40 (d, J = 6.5 Hz, 1H, OC*H*H-Ox), 4.35 (d, J = 6.6 Hz, 1H, OC*H*H-Ox), 4.28 (d, J = 6.6 Hz, 1H, OCH*H*-Ox), 4.22 (d, J = 6.5 Hz, 1H, OCH*H*-Ox), 4.14–4.04 (m, 1H, CH, α-Leu), 3.71 (dd, J = 14.1, 5.8 Hz, 1H, C*H*HGOx), 3.52–3.37 (m, 2H, CHα-Ala, CH*H*GOx), 1.84 (br. s, 1H, NH), 1.69–1.61 (m, 2H, C*H*Hβ-Leu, CHγ-Leu), 1.51–1.45 (m, 1H, CH*H*β-Leu), 1.43 (s, 9H, 3 × CH_3, Boc), 1.36 (d, J = 7.0 Hz, 3H, CH_3β-Ala), 1.33 (d, J = 7.0 Hz, 3H, CH_3β-Ala), 0.93 (d, J = 6.2 Hz, 3H, CH_3δ-Leu), 0.92 (d, J = 6.0 Hz, 3H, CH_3δ-Leu); **^{13}C NMR** (101 MHz, $CDCl_3$) δ_C ppm 176.4 (C=O), 172.8 (C=O), 172.6 (C=O), 155.9 (C=O, Boc), 135.4 (C), 128.8 (CH), 128.7 (CH), 128.5 (CH), 80.4 (C, Boc), 80.2 (OCH_2), 79.8 (OCH_2), 67.2 (CH_2, Bn), 59.5 (C, Ox), 53.3 (CH, α-Leu), 51.3 (CH, α-Ala), 49.1 (CH, α-Ala), 43.5 (CH_2, GOx), 41.3 (CH_2, β-Leu), 28.4 (CH_3, Boc), 24.8 (CH, γ-Leu), 23.2 (CH_3, δ-Leu), 21.9 (CH_3, δ-Leu), 20.6 (CH_3, β-Ala), 18.3 (CH_3, β-Ala); **ν_{max}** (neat) = 3296, 2967, 1710, 1629,

1516, 1453, 1366, 1250, 1163, 1047, 975, 732 cm^{-1}; **MS** (ESI^+) *m/z* 549 $[M+H]^+$, 571 $[M+Na]^+$; **HRMS** (ESI^+) calcd. for $C_{28}H_{45}N_4NaO_7$ $[M+Na]^+$ 549.3283, found 549.3285; $[\boldsymbol{\alpha}]_D^{27}$ –35.5 (*c* 1.00, $CHCl_3$).

Cbz-Tyr(Bn)-Leu-Ala-GOx-Ala-OBn (62)

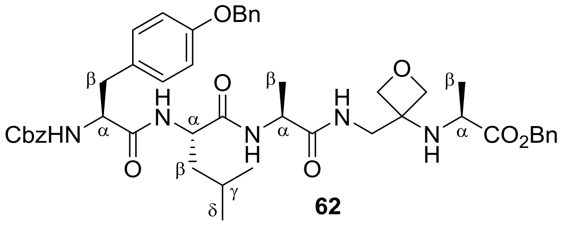

To a solution of Boc-Leu-Ala-GOx-Ala-OBn (**61**) (637 mg, 1.16 mmol, 1.0 equiv) in CH_2Cl_2 (2.0 mL) was added TFA (2.0 mL) and the mixture was stirred at room temperature for 1 h. The mixture was concentrated under reduced pressure and the resulting residue repeatedly dissolved in CH_2Cl_2 (3 × 10 mL) and concentrated under reduced pressure to give the crude amine. The residue was dissolved in CH_2Cl_2 (12 mL), Cbz-Tyr(Bn)-OH (565 mg, 1.39 mmol, 1.2 equiv), HATU (565 mg, 1.39 mmol, 1.2 equiv) and DIPEA (808 μL, 4.64 mmol, 4.0 equiv) were added, and the reaction mixture was stirred at room temperature for 16 h. The reaction mixture was diluted with CH_2Cl_2 (20 mL) and washed with 10% citric acid solution (2 × 30 mL) and saturated $NaHCO_3$ solution (2 × 30 mL), dried over $MgSO_4$, filtered and concentrated under reduced pressure. The residue was purified by column chromatography (SiO_2, CH_2Cl_2/MeOH 19:1→9:1) to give pentapeptide **62** (463 mg, 0.55 mmol, 48%) as an off-white solid. $\mathbf{R_f}$ (CH_2Cl_2/MeOH 9:1) 0.47; **mp** 134–136 °C; **^{1}H NMR** (400 MHz, $CDCl_3$) δ_H ppm 7.33–7.19 (m, 15H, ArH), 6.98 (d, *J* = 8.1 Hz, 2H, ArH), 6.83 (br. s, 1H, NH), 6.79 (d, *J* = 8.1 Hz, 2H, ArH), 6.59 (br. s, 1H, NH), 5.37 (br. s, 1H, NH), 5.04 (s, 2H, CH_2Ph), 4.98 (s, 2H, CH_2Ph), 4.91 (s, 2H, CH_2Ph), 4.33 (m, 6H, CHα-Tyr, CHα-Leu, CHα-Ala, OCH_2-Ox, OC*H*H-Ox), 4.19 (d, *J* = 6.1 Hz, 1H, OCH*H*-Ox), 3.77–3.67 (m, 1H, C*H*HGOx), 3.51–3.38 (m, 2H, CH*H*GOx, CHα-Ala), 2.96 (dd, *J* = 14.0, 5.4 Hz, 1H, C*H*Hβ-Tyr), 2.88 (dd, *J* = 14.0, 7.2 Hz, 1H, CH*H*β-Tyr), 2.09 (br. s, 2H, 2 × NH), 1.61–1.52 (m, 1H, C*H*Hβ-Leu), 1.44–1.34 (m, 2H, CHγ-Leu, CH*H*β-Leu), 1.30 (d, *J* = 6.5 Hz, 3H, CH_3β-Ala), 1.22 (d, *J* = 6.9 Hz, 3H, CH_3β-Ala), 0.80 (d, *J* = 6.2 Hz, 3H, CH_3δ-Leu), 0.79 (d, *J* = 6.2 Hz, 3H, CH_3δ-Leu); **^{13}C NMR** (126 MHz, $CDCl_3$) δ_C ppm 176.3 (C=O), 173.0 (C=O), 171.7 (C=O), 171.6 (C=O), 158.2 (C), 156.6 (C=O, Cbz), 136.9 (C), 135.9 (C), 135.5 (C), 130.4 (CH), 128.8 (CH), 128.7 (CH), 128.6 (CH), 128.5 (CH), 128.43 (CH), 128.41 (CH), 128.2 (CH), 128.1 (CH), 128.0 (C), 127.6 (CH), 115.3 (CH), 80.0 (OCH_2), 79.9 (OCH_2), 70.1 (CH_2, Bn), 67.5 (CH_2, Bn), 67.1 (CH_2, Bn), 59.6 (C, Ox), 56.7 (CH, α-Tyr), 52.4 (CH, α-Leu), 51.2 (CH, α-Ala), 49.2 (CH, α-Ala), 43.6 (CH_2, GOx), 40.9 (CH_2, β-Leu), 37.2 (CH_2, β-Tyr), 24.9 (CH, γ-Leu), 23.1 (CH_3, δ-Leu), 21.9 (CH_3, δ-Leu), 20.6 (CH_3, β-Ala), 18.1 (CH_3, β-Ala); $\mathbf{\nu_{max}}$ (neat) = 3285, 2955, 1635, 1510, 1453, 1381, 1232, 1046, 971, 736, 695 cm^{-1}; **MS** (ESI^+) *m/z* 836 $[M+H]^+$, 858 $[M+Na]^+$; **HRMS** (ESI^+) calcd. for $C_{47}H_{57}N_5NaO_9$ $[M+Na]^+$ 858.4048, found 858.4047; $[\boldsymbol{\alpha}]_D^{27}$ –14.6 (*c* 0.86, $CHCl_3$).

H-Tyr-Leu-Ala-GOx-Ala-OH (63)

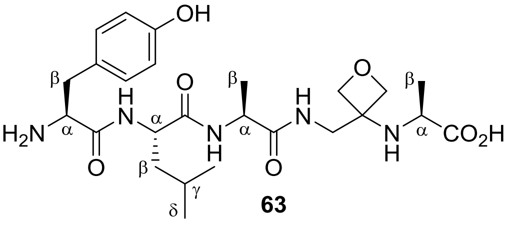

To a solution of pentapeptide **62** (348 mg, 0.42 mmol) in anhydrous MeOH (4.0 mL) was added 10 wt% Pd/C (35 mg, 10 wt%) and the reaction flask was evacuated, filled with nitrogen, evacuated, and placed under an atmosphere of hydrogen (balloon). The reaction mixture was stirred at room temperature for 16 h, placed under nitrogen and filtered through a plug of Celite, which was washed with MeOH (3×). The filtrate was concentrated *in vacuo* to give **63** as an off-white solid (273 mg, >99%), which required no further purification. **mp** 167–169 °C (decomposition); **^{1}H NMR** (400 MHz, CD_3OD) δ_H ppm 7.10 (d, *J* = 8.0 Hz, 2H, ArH), 6.76 (d, *J* = 8.0 Hz, 2H, ArH), 4.61 (d, *J* = 7.4 Hz, 1H, OC*H*H-Ox), 4.57 (d,

J = 6.9 Hz, 1H, OC*H*H-Ox), 4.48–4.33 (m, 3H, 2 × OCH*H*-Ox, CHα-Leu), 4.31 (q, J = 6.7 Hz, 1H, CHα-Ala), 4.05 (t, J = 6.5 Hz, 1H, CHα-Tyr), 3.81 (d, J = 13.8 Hz, 1H, C*H*HGOx), 3.52 (d, J = 13.8 Hz, 1H, CH*H*GOx), 3.45 (q, J = 6.5 Hz, 1H, CHα-Ala), 3.15 (dd, J = 14.0, 4.8 Hz, 1H, C*H*Hβ-Tyr), 2.94 (dd, J = 14.0, 8.2 Hz, 1H, CH*H*β-Tyr), 1.70–1.55 (m, 3H, CH_2β-Leu, CHγ-Leu), 1.39 (d, J = 7.0 Hz, 3H, CH_3β-Ala), 1.36 (d, J = 6.8 Hz, 3H, CH_3β-Ala), 0.96 (d, J = 4.6 Hz, 3H, CH_3δ-Leu), 0.94 (d, J = 4.6 Hz, 3H, CH_3δ-Leu); **^{13}C NMR** (101 MHz, CD_3OD) δ_C ppm 175.6 (C=O), 175.5 (C=O), 173.8 (C=O), 170.6 (C=O), 158.2 (C), 131.7 (CH), 126.3 (C), 116.8 (CH), 80.1 (OCH_2), 79.5 (OCH_2), 61.8 (C, Ox), 56.0 (CH, α-Tyr), 54.9 (CH, α-Ala), 53.5 (CH, α-Leu), 50.9 (CH, α-Ala), 43.6 (CH_2, GOx), 41.7 (CH_2, β-Leu), 38.1 (CH_2, β-Tyr), 25.8 (CH, γ-Leu), 23.4 (CH_3, δ-Leu), 22.1 (CH_3, δ-Leu), 20.4 (CH_3, β-Ala), 17.9 (CH_3, β-Ala); **ν_{max}** (neat) = 3248, 2957, 1643, 1515, 1452, 1366, 1233, 1171, 1102, 979, 829 cm^{-1}; **MS** (ESI^+) *m/z* 522 $[M+H]^+$, 544 $[M+Na]^+$; **HRMS** (ESI^+) calcd. for $C_{25}H_{39}N_5NaO_7$ $[M+Na]^+$ 544.2742, found 544.2753; $[\alpha]_D^{28}$ –18.5 (*c* 0.55, MeOH).

*Preparation of pentapeptide **68***

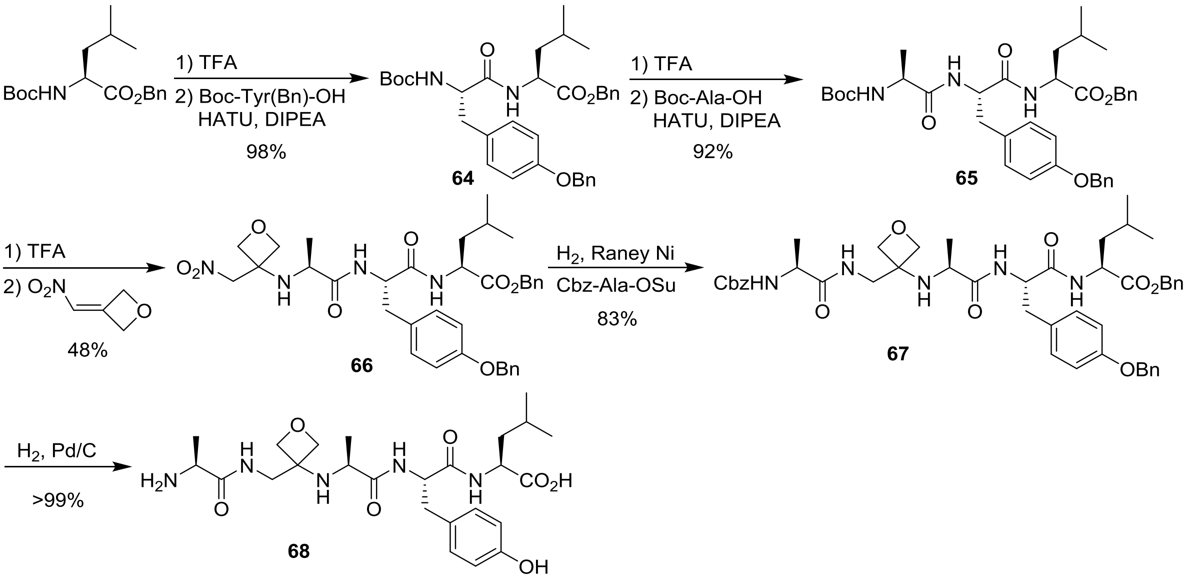

Boc-Tyr(Bn)-Leu-OBn (64)

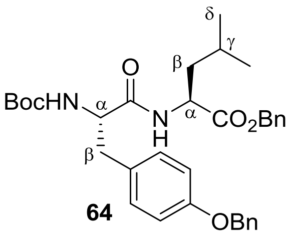

To a solution of Boc-Leu-OBn[1] (8.04 g, 25.0 mmol, 1.0 equiv) in CH_2Cl_2 (25 mL) was added TFA (25 mL) and the mixture was stirred at room temperature for 1 h. The reaction mixture was concentrated under reduced pressure and the resulting residue repeatedly dissolved in CH_2Cl_2 (3 × 20 mL) and concentrated under reduced pressure to give the crude amine. The residue was dissolved in CH_2Cl_2 (250 mL), Boc-Tyr(Bn)-OH (11.1 g, 30.0 mmol, 1.2 equiv), HATU (11.4 g, 30.0 mmol, 1.2 equiv) and DIPEA (17.4 mL, 100 mmol, 4.0 equiv) were added subsequently, and the reaction mixture was stirred at room temperature for 16 h. The reaction mixture was washed with 10% citric acid solution (2 × 100 mL) and saturated $NaHCO_3$ solution (2 × 100 mL), dried over $MgSO_4$, filtered and concentrated under reduced pressure. The residue was purified by column chromatography (SiO_2, PE/EtOAc 2:1→1:1) to give dipeptide **64** (14.1 g, 24.5 mmol, 98%) as a white solid. **R_f** (PE/EtOAc 1:1) 0.57; **mp** 116–117 °C; **^{1}H NMR** (400 MHz, $CDCl_3$) δ_H ppm 7.44–7.29 (m, 10H, ArH), 7.11 (d, J = 8.4 Hz, 2H, ArH), 6.88 (d, J = 8.4 Hz, 2H, ArH), 6.28 (d, J = 8.2 Hz, 1H, NH), 5.15 (d, J = 12.3 Hz, 1H, C*H*HPh), 5.11 (d, J = 12.3 Hz, 1H, CH*H*Ph), 5.02 (s, 2H, CH_2Ph), 5.00 (br. s, 1H, NH), 4.62 (td, J = 8.4, 5.3 Hz, 1H, CHα-Leu), 4.31 (q, J = 6.4 Hz, 1H, CHα-Tyr), 3.02 (dd, J = 12.6, 5.1 Hz, 1H, C*H*Hβ-Tyr), 2.98 (dd, J = 12.6,

5.3 Hz, 1H, CH*H*β-Tyr), 1.62–1.46 (m, 3H, CH_2β-Leu, CHγ-Leu), 1.41 (s, 9H, 3 × CH_3, Boc), 0.89 (d, *J* = 6.6 Hz, 3H, CH_3δ-Leu), 0.87 (d, *J* = 6.5 Hz, 3H, CH_3δ-Leu); **^{13}C NMR** (101 MHz, $CDCl_3$) δ_C ppm 172.4 (C=O), 171.1 (C=O), 158.0 (C), 155.5 (C=O, Boc), 137.1 (C), 135.5 (C), 130.6 (CH), 128.9 (C), 128.73 (CH), 128.71 (CH), 128.5 (CH), 128.3 (CH), 128.1 (CH), 127.6 (CH), 115.1 (CH), 80.3 (C, Boc), 70.1 (CH_2, Bn), 67.1 (CH_2, Bn), 55.9 (CH, α-Tyr), 51.0 (CH, α-Leu), 41.7 (CH_2, β-Leu), 37.4 (CH_2, β-Tyr), 28.4 (CH_3, Boc), 24.8 (CH, γ-Leu), 22.9 (CH_3, δ-Leu), 22.0 (CH_3, δ-Leu); **ν_{max}** (neat) = 3325, 2957, 1734, 1658, 1510, 1367, 1299, 1238, 1164, 1021, 862, 741, 697 cm^{-1}; **MS** (ESI$^+$) *m/z* 575 $[M+H]^+$, 597 $[M+Na]^+$; **HRMS** (ESI$^+$) calcd. for $C_{34}H_{42}N_2NaO_6$ $[M+Na]^+$ 597.2935, found 597.2939; $[\alpha]_D^{25}$ –3.98 (*c* 1.00, $CHCl_3$).

Boc-Ala-Tyr(Bn)-Leu-OBn (65)

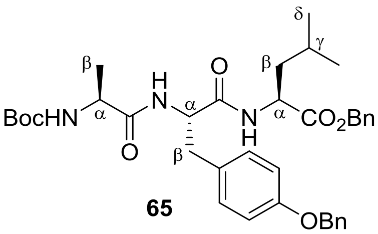

65

To a solution of Boc-Tyr(Bn)-Leu-OBn (**64**) (12.9 g, 22.5 mmol, 1.0 equiv) in CH_2Cl_2 (22.5 mL) was added TFA (22.5 mL) and the mixture was stirred at room temperature for 1 h. The reaction mixture was concentrated under reduced pressure and the resulting residue repeatedly dissolved in CH_2Cl_2 (3 × 20 mL) and concentrated *in vacuo* to give the crude amine. The residue was dissolved in CH_2Cl_2 (225 mL), Boc-Ala-OH (5.11 g, 27.0 mmol, 1.2 equiv), HATU (10.3 g, 27.0 mmol, 1.2 equiv) and DIPEA (15.7 mL, 90.0 mmol, 4.0 equiv) were added, and the mixture was stirred at room temperature for 16 h. The reaction mixture was washed with 10% citric acid solution (2 × 100 mL) and saturated $NaHCO_3$ solution (2 × 100 mL), dried over $MgSO_4$, filtered and concentrated under reduced pressure. The residue was purified by column chromatography (SiO_2, PE/EtOAc 1:1) to give tripeptide **65** (13.4 g, 20.7 mmol, 92%) as a white foam. **R_f** (EtOAc) 0.72; **mp** 119–121 °C; **^{1}H NMR** (400 MHz, $CDCl_3$) δ_H ppm 7.45–7.28 (m, 10H, ArH), 7.09 (d, *J* = 8.2 Hz, 2H, ArH), 6.86 (d, *J* = 8.2 Hz, 2H, ArH), 6.72 (br. s, 1H, NH), 6.53 (br. s, 1H, NH), 5.15 (d, *J* = 12.4 Hz, 1H, C*H*HPh), 5.11 (d, *J* = 12.4 Hz, 1H, CH*H*Ph), 4.99 (d, *J* = 3.2 Hz, 2H, CH_2Ph), 4.89 (br. s, 1H, NH), 4.71–4.53 (m, 2H, CHα-Tyr, CHα-Leu), 4.23–3.93 (m, 1H, CHα-Ala), 3.07 (dd, *J* = 13.6, 4.8 Hz, 1H, C*H*Hβ-Tyr), 2.98 (dd, *J* = 13.6, 6.8 Hz, 1H, CH*H*β-Tyr), 1.61–1.48 (m, 3H, CH_2β-Leu, CHγ-Leu), 1.41 (s, 9H, 3 × CH_3, Boc), 1.29 (d, *J* = 7.0 Hz, 3H, CH_3β-Ala), 0.87 (d, *J* = 5.9 Hz, 3H, CH_3δ-Leu), 0.86 (d, *J* = 5.9 Hz, 3H, CH_3δ-Leu); **^{13}C NMR** (101 MHz, $CDCl_3$) δ_C ppm 172.5 (C=O), 172.2 (C=O), 170.5 (C=O), 158.0 (C), 155.6 (C=O, Boc), 137.1 (C), 135.6 (C), 130.6 (CH), 129.6 (CH), 128.70 (CH), 128.66 (C), 128.5 (CH), 128.3 (CH), 128.1 (CH), 127.6 (CH), 115.1 (CH), 80.4 (C, Boc), 70.1 (CH_2, Bn), 67.1 (CH_2, Bn), 54.3 (CH, α-Tyr), 51.1 (CH, α-Leu), 50.7 (CH, α-Ala), 41.3 (CH_2, β-Leu), 37.0 (CH_2, β-Tyr), 28.4 (CH_3, Boc), 24.8 (CH, γ-Leu), 22.8 (CH_3, δ-Leu), 22.0 (CH_3, δ-Leu), 18.3 (CH_3, β-Ala); **ν_{max}** (neat) = 3294, 2962, 1733, 1649, 1511, 1453, 1367, 1264, 1164, 1025, 738, 696 cm^{-1}; **MS** (ESI$^+$) *m/z* 646 $[M+H]^+$, 668 $[M+Na]^+$; **HRMS** (ESI$^+$) calcd. for $C_{37}H_{47}N_3NaO_7$ $[M+Na]^+$ 668.3306, found 668.3303; $[\alpha]_D^{27}$ –24.4 (*c* 0.76, $CHCl_3$).

NO_2-GOx-Ala-Tyr(Bn)-Leu-OBn (66)

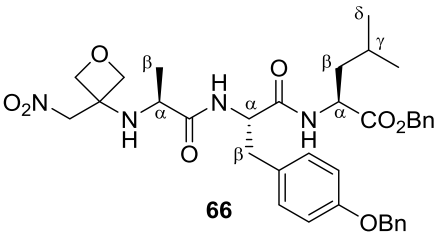

66

To a solution of Boc-Ala-Tyr(Bn)-Leu-OBn (**65**) (3.87 g, 6.00 mmol, 1.0 equiv) in CH_2Cl_2 (6.0 mL) was added TFA (6.0 mL) and the mixture was stirred at room temperature for 1 h. The reaction mixture was concentrated under reduced pressure and the resulting residue repeatedly dissolved in CH_2Cl_2 (3 × 10 mL) and concentrated under reduced pressure to give the crude amine. In a second reaction vessel, oxetane-3-one

(770 µL, 12.0 mmol, 2.0 equiv), nitromethane (910 µL, 16.8 mmol, 2.8 equiv) and triethylamine (335 µL, 2.40 mmol, 0.4 equiv) were combined at 0 °C and stirred for 1 h at room temperature. The mixture was dissolved in anhydrous CH_2Cl_2 (20 mL), cooled to –78 °C, and triethylamine (3.35 mL, 24.0 mmol, 4.0 equiv) was added followed by dropwise addition of a solution of methanesulfonyl chloride (930 µL, 12.0 mmol, 2.0 equiv) in anhydrous CH_2Cl_2 (6.0 mL). The reaction mixture was stirred at –78 °C for 1.5 h and a solution of the crude amine and triethylamine (1.26 mL, 9.0 mmol, 1.5 equiv) in anhydrous CH_2Cl_2 (20 mL) was added slowly *via* syringe. The reaction mixture was allowed to warm to room temperature and stirred for 16 h. A saturated solution of NH_4Cl (30 mL) was added and stirred for 10 min. The layers were separated and the aqueous one extracted with CH_2Cl_2 (2 × 20 mL) and EtOAc (2 × 20 mL). The combined organic phases were washed with saturated aqueous $NaHCO_3$ solution (30 mL), brine (30 mL), dried over $MgSO_4$, filtered and concentrated under reduced pressure. The residue was purified by column chromatography (SiO_2, PE/EtOAc 1:1→EtOAc) to give **66** (1.90 g, 2.88 mmol, 48%) as an off-white solid. $\mathbf{R_f}$ (EtOAc) 0.61; **mp** 48–50 °C; **^{1}H NMR** (400 MHz, $CDCl_3$) δ_H ppm 7.46–7.30 (m, 11H, ArH, NH), 7.09 (d, *J* = 8.5 Hz, 2H, ArH), 6.87 (d, *J* = 8.5 Hz, 2H, ArH), 6.39 (dd, *J* = 12.4, 8.3 Hz, 1H, NH), 5.14 (s, 2H, CH_2Ph), 5.02 (s, 2H, CH_2Ph), 4.73 (s, 2H, CH_2GOx), 4.62–4.51 (m, 3H, CHα-Tyr, CHα-Leu, OC*H*H-Ox), 4.47 (d, *J* = 7.5 Hz, 1H, OC*H*H-Ox), 4.43 (d, *J* = 7.5 Hz, 1H, OCH*H*-Ox), 4.39 (d, *J* = 7.3 Hz, 1H, OCH*H*-Ox), 3.47 (q, *J* = 7.0 Hz, 1H, CHα-Ala), 3.04 (dd, *J* = 14.1, 6.4 Hz, 1H, C*H*Hβ-Tyr), 2.95 (dd, *J* = 14.1, 7.9 Hz, 1H, CH*H*β-Tyr), 1.81 (br. s, 1H, NH), 1.64–1.48 (m, 3H, CH_2β-Leu, CHγ-Leu), 1.21 (d, *J* = 7.0 Hz, 3H, CH_3β-Ala), 0.87 (d, *J* = 6.0 Hz, 6H, 2 × CH_3δ-Leu); **^{13}C NMR** (101 MHz, $CDCl_3$) δ_C ppm 174.9 (C=O), 172.4 (C=O), 170.8 (C=O), 158.0 (C), 137.1 (C), 135.5 (C), 130.5 (CH), 129.1 (C), 128.71 (CH), 128.68 (CH), 128.5 (CH), 128.3 (CH), 128.1 (CH), 127.5 (CH), 115.1 (CH), 79.2 (CH_2, GOx), 77.9 (OCH_2), 77.6 (OCH_2), 70.1 (CH_2, Bn), 67.2 (CH_2, Bn), 59.3 (C, Ox), 54.2 (CH, α-Tyr), 52.8 (CH, α-Ala), 51.1 (CH, α-Leu), 41.3 (CH_2, β-Leu), 37.3 (CH_2, β-Tyr), 24.9 (CH, γ-Leu), 22.8 (CH_3, δ-Leu), 21.9 (CH_3, δ-Leu), 20.7 (CH_3, β-Ala); $\mathbf{\nu_{max}}$ (neat) = 3290, 2960, 1738, 1643, 1551, 1510, 1379, 1239, 1177, 976, 735, 696 cm^{-1}; **MS** (ESI^+) *m/z* 661 $[M+H]^+$, 683 $[M+Na]^+$; **HRMS** (ESI^+) calcd. for $C_{36}H_{44}N_4NaO_8$ $[M+Na]^+$ 683.3051, found 683.3056; $[\boldsymbol{\alpha}]_D^{27}$ –10.0 (*c* 0.93, $CHCl_3$).

Cbz-Ala-GOx-Ala-Tyr(Bn)-Leu-OBn (67)

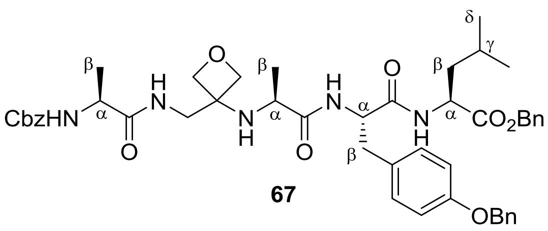

67

To a solution of NO_2-GOx-Ala-Tyr(Bn)-Leu-OBn (**66**) (1.26 g, 1.90 mmol, 1.0 equiv) in THF (20 mL) was added Cbz-Ala-OSu (1.22 g, 3.80 mmol, 2.0 equiv), $NaHCO_3$ (638 mg, 7.60 mmol, 4.0 equiv) and Raney Ni (slurry in H_2O, 4.0 mL). The solution was placed under an atmosphere of nitrogen, evacuated and filled with hydrogen (balloon). The mixture was stirred for 2.5 h at room temperature, filtered through a plug of Celite eluting with EtOAc, and the filtrate was concentrated *in vacuo*. Pentapeptide **67** was afforded after purification by column chromatography (SiO_2, EtOAc→CH_2Cl_2/MeOH 9:1) as an off-white foam (989 mg, 1.18 mmol, 83%). $\mathbf{R_f}$ (CH_2Cl_2/MeOH 9:1) 0.34; **mp** 64–66 °C; **^{1}H NMR** (400 MHz, $CDCl_3$) δ_H ppm 7.87 (d, *J* = 7.9 Hz, 1H, NH), 7.41–7.29 (m, 16H, ArH, NH), 7.10 (d, *J* = 8.4 Hz, 2H, ArH), 6.85 (d, *J* = 8.4 Hz, 2H, ArH), 6.27 (br. s, 1H, NH), 5.79 (t, *J* = 7.4 Hz, 1H, NH), 5.14 (s, 2H, CH_2Ph), 5.06 (s, 2H, CH_2Ph), 5.00 (s, 2H, CH_2Ph), 4.69–4.60 (m, 1H, CHα-Tyr), 4.55–4.47 (m, 1H, CHα-Ala), 4.39 (d, *J* = 6.7 Hz, 1H, OC*H*H-Ox), 4.37–4.28 (m, 3H, OC*H*H-Ox, OCH*H*-Ox, CHα-Leu,), 4.26 (d, *J* = 6.8 Hz, 1H, OCH*H*-Ox), 3.94 (dd, *J* = 14.0, 8.2 Hz, 1H, C*H*HGOx), 3.33 (q, *J* = 6.7 Hz, 1H, CHα-Ala), 3.17 (dd, *J* = 14.0, 3.1 Hz, 1H, CH*H*GOx), 3.01–2.89 (m, 2H, CH_2β-Tyr), 1.77 (br. s, 1H, NH), 1.57–1.40 (m, 3H, CH_2β-Leu, CHγ-Leu), 1.32 (d, *J* = 7.0 Hz, 3H, CH_3β-Ala), 1.22 (d, *J* = 7.0 Hz, 3H, CH_3β-Ala), 0.83 (d, *J* = 6.1 Hz, 3H, CH_3δ-Leu), 0.80 (d,

J = 6.1 Hz, 3H, CH_3δ-Leu); **^{13}C NMR** (126 MHz, $CDCl_3$) $δ_C$ ppm 175.5 (C=O), 174.2 (C=O), 172.1 (C=O), 171.8 (C=O), 158.0 (C), 156.1 (C=O, Cbz), 137.0 (C), 136.6 (C), 135.4 (C), 130.5 (CH), 128.8 (CH), 128.72 (CH), 128.68 (CH), 128.64 (CH), 128.60 (C), 128.4 (CH), 128.3 (CH), 128.12 (CH), 128.11 (CH), 127.6 (CH), 115.1 (CH), 79.5 (OCH_2), 78.5 (OCH_2), 70.1 (CH_2, Bn), 67.3 (CH_2, Bn), 66.9 (CH_2, Bn), 61.1 (C, Ox), 53.8 (CH, α-Tyr), 53.2 (CH, α-Ala), 51.4 (CH, α-Leu), 50.7 (CH, α-Ala), 44.3 (CH_2, GOx), 41.1 (CH_2, β-Leu), 38.0 (CH_2, β-Tyr), 24.8 (CH, γ-Leu), 22.8 (CH_3, δ-Leu), 21.9 (CH_3, δ-Leu), 21.1 (CH_3, β-Ala), 18.7 (CH_3, β-Ala); **$ν_{max}$** (neat) = 3287, 2958, 1736, 1645, 1515, 1453, 1232, 1026, 735, 695 cm^{-1}; **MS** (ESI^+) *m/z* 836 $[M+H]^+$, 858 $[M+Na]^+$; **HRMS** (ESI^+) calcd. for $C_{47}H_{57}N_5NaO_9$ $[M+Na]^+$ 858.4048, found 858.4049; $[\boldsymbol{\alpha}]_D^{28}$ +17.1 (*c* 0.44, $CHCl_3$).

H-Ala-GOx-Ala-Tyr-Leu-OH (68)

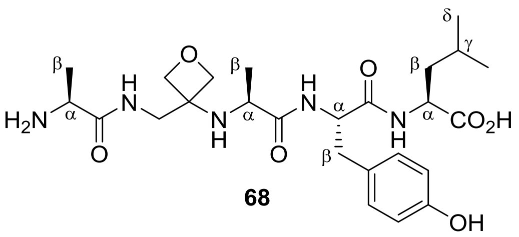

68

To a solution of pentapeptide **67** (1.75 g, 2.09 mmol) in anhydrous MeOH (21 mL) was added 10 wt% Pd/C (175 mg, 10 wt%) and the reaction flask was evacuated, filled with nitrogen, evacuated, and placed under an atmosphere of hydrogen (balloon). The reaction mixture was stirred at room temperature for 16 h, placed under nitrogen and filtered through a plug of Celite, which was washed with MeOH (3×). The filtrate was concentrated *in vacuo* to give **68** as a white solid (1.14 g, >99%), which required no further purification. **mp** 186–188 °C (decomposition); **^{1}H NMR** (400 MHz, CD_3OD) $δ_H$ ppm 7.08 (d, J = 8.3 Hz, 2H, ArH), 6.69 (d, J = 8.3 Hz, 2H, ArH), 4.66 (dd, J = 8.8, 4.7 Hz, 1H, CHα-Tyr), 4.45 (d, J = 6.6 Hz, 1H, OC*H*H-Ox), 4.39 (t, J = 7.3 Hz, 1H, CHα-Leu), 4.31 (s, 2H, OCH_2-Ox), 4.24 (d, J = 6.6 Hz, 1H, OCH*H*-Ox), 3.95 (q, J = 7.0 Hz, 1H, CHα-Ala), 3.58 (d, J = 14.1 Hz, 1H, C*H*HGOx), 3.35–3.31 (m, 2H, CH*H*GOx, CHα-Ala), 3.11 (dd, J = 14.1, 4.6 Hz, 1H, C*H*Hβ-Tyr), 2.85 (dd, J = 14.1, 8.9 Hz, 1H, CH*H*β-Tyr), 1.77–1.62 (m, 3H, CH_2β-Leu, CHγ-Leu), 1.49 (d, J = 7.0 Hz, 3H, CH_3β-Ala), 1.20 (d, J = 7.0 Hz, 3H, CH_3β-Ala), 0.96 (d, J = 6.1 Hz, 3H, CH_3δ-Leu), 0.91 (d, J = 6.0 Hz, 3H, CH_3δ-Leu); **^{13}C NMR** (101 MHz, CD_3OD) $δ_C$ ppm 177.9 (C=O), 176.0 (C=O), 173.8 (C=O), 172.0 (C=O), 157.4 (C), 131.6 (CH), 128.7 (C), 116.2 (CH), 80.2 (OCH_2), 80.0 (OCH_2), 61.6 (C, Ox), 54.8 (CH, α-Tyr), 53.9 (CH, α-Ala), 52.5 (CH, α-Leu), 50.5 (CH, α-Ala), 44.9 (CH_2, GOx), 41.6 (CH_2, β-Leu), 38.4 (CH_2, β-Tyr), 26.0 (CH, γ-Leu), 23.4 (CH_3, δ-Leu), 21.9 (CH_3, δ-Leu), 20.8 (CH_3, β-Ala), 17.8 (CH_3, β-Ala); **$ν_{max}$** (neat) = 3240, 2957, 1646, 1515, 1448, 1223, 1147, 969, 830 cm^{-1}; **MS** (ESI^+) *m/z* 522 $[M+H]^+$, 544 $[M+Na]^+$; **HRMS** (ESI^+) calcd. for $C_{25}H_{40}N_5O_7$ $[M+H]^+$ 522.2922, found 522.2919; $[\boldsymbol{\alpha}]_D^{28}$ +14.8 (*c* 0.12, MeOH).

*Preparation of pentapeptide **73***

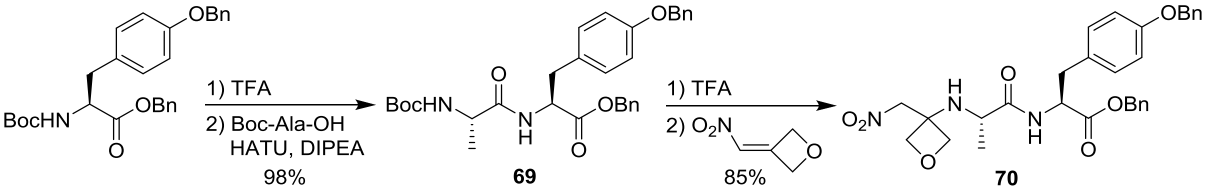

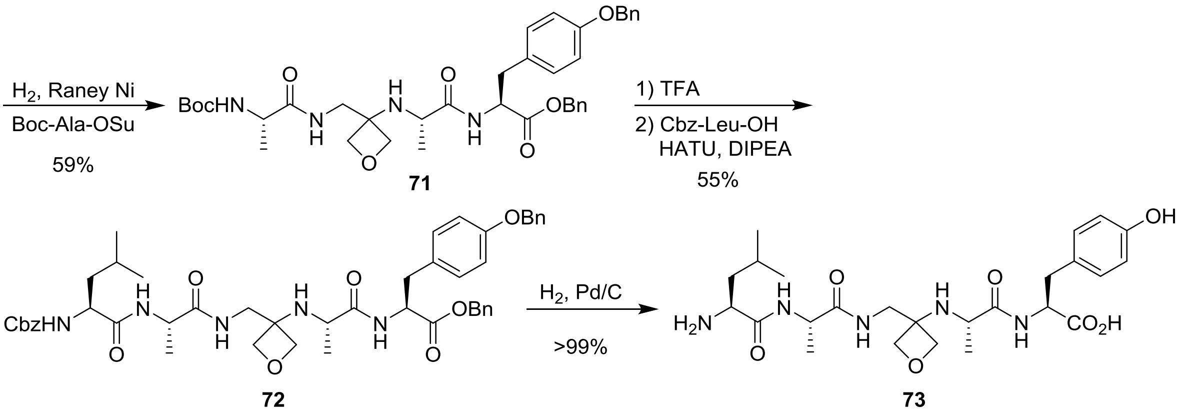

Boc-Ala-Tyr(Bn)-OBn (69)

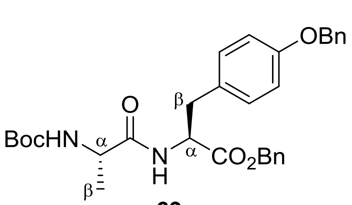

69

To a solution of Boc-Tyr(Bn)-OBn[1] (11.3 g, 25.0 mmol, 1.0 equiv) in CH_2Cl_2 (25 mL) was added TFA (25 mL) and the mixture was stirred at room temperature for 30 min (*Caution – gas evolution!*). The reaction mixture was concentrated under reduced pressure and the resulting residue repeatedly dissolved in CH_2Cl_2 (3 × 20 mL) and concentrated under reduced pressure to give the crude amine. The residue was dissolved in CH_2Cl_2 (250 mL), Boc-Ala-OH (5.68 g, 30.0 mmol, 1.2 equiv), HATU (11.4 g, 30.0 mmol, 1.2 equiv) and DIPEA (17.4 mL, 100 mmol, 4.0 equiv) were added subsequently, and the reaction mixture was stirred at room temperature for 16 h. The reaction mixture was washed with 10% citric acid solution (2 × 100 mL) and saturated $NaHCO_3$ solution (2 × 100 mL), dried over $MgSO_4$, filtered and concentrated under reduced pressure. The residue was purified by column chromatography (SiO_2, PE/EtOAc 2:1→1:1) to give dipeptide **69** (13.1 g, 24.5 mmol, 98%) as a white solid. **R$_f$** (EtOAc) 0.67; **mp** 111–113 °C; **^{1}H NMR** (400 MHz, $CDCl_3$) δ_H ppm 7.44–7.28 (m, 10H, ArH), 6.92 (d, *J* = 8.5 Hz, 2H, ArH), 6.81 (d, *J* = 8.5 Hz, 2H, ArH), 6.53 (d, *J* = 7.7 Hz, 1H, NH), 5.17 (d, *J* = 12.1 Hz, 1H, C*H*HPh), 5.10 (d, *J* = 12.1 Hz, 1H, CH*H*Ph), 5.01 (s, 2H, CH_2Ph), 4.98 (br. s, 1H, NH), 4.85 (dd, *J* = 13.3, 5.8 Hz, 1H, CHα-Tyr), 4.15 (br. s, 1H, CHα-Ala), 3.08 (dd, *J* = 13.7, 5.6 Hz, 1H, C*H*Hβ-Tyr), 3.04 (dd, *J* = 13.8, 5.4 Hz, 1H, CH*H*β-Tyr), 1.44 (s, 9H, 3 × CH_3, Boc), 1.30 (d, *J* = 7.0 Hz, 3H, CH_3β-Ala); **^{13}C NMR** (101 MHz, $CDCl_3$) δ_C ppm 172.3 (C=O), 171.3 (C=O), 158.0 (C), 155.5 (C=O, Boc), 137.1 (C), 135.2 (C), 130.5 (CH), 128.74 (CH), 128.73 (CH), 128.71 (CH), 128.66 (CH), 128.1 (CH), 127.9 (C), 127.6 (CH), 115.0 (CH), 80.2 (C, Boc), 70.1 (CH_2, Bn), 67.4 (CH_2, Bn), 53.4 (CH, α-Tyr), 50.2 (CH, α-Ala), 37.1 (CH_2, β-Tyr), 28.4 (CH_3, Boc), 18.5 (CH_3, β-Ala); **ν_{max}** (neat) = 3339, 1730, 1666, 1511, 1321, 1243, 1163, 1070, 1027, 823, 750, 733, 695 cm^{-1}; **MS** (ESI$^+$) *m/z* 555 $[M+Na]^+$; **HRMS** (ESI$^+$) calcd. for $C_{31}H_{36}N_2NaO_6$ $[M+Na]^+$ 555.2466, found 555.2464; $[\boldsymbol{\alpha}]_D^{26}$ –7.2 (*c* 0.27, $CHCl_3$).

O_2N-GOx-Ala-Tyr(Bn)-OBn (70)

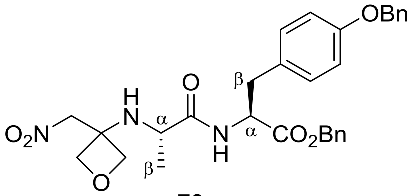

70

To a solution of Boc-Ala-Tyr(Bn)-OBn (**69**) (3.20 g, 6.00 mmol, 1.0 equiv) in CH_2Cl_2 (6.0 mL) was added TFA (6.0 mL) and the mixture was stirred at room temperature for 1 h. The reaction mixture was concentrated under reduced pressure and the resulting residue repeatedly dissolved in CH_2Cl_2 (3 × 10 mL) and concentrated under reduced pressure to give the crude amine. In a second reaction vessel, oxetane-3-one (770 µL, 22.0 mmol, 2.0 equiv), nitromethane (910 µL, 16.8 mmol, 2.8 equiv) and triethylamine (335 µL, 2.40 mmol, 0.4 equiv) were combined at 0 °C and

stirred for 1 h at room temperature. The mixture was dissolved in anhydrous CH_2Cl_2 (40 mL), cooled to –78 °C, and triethylamine (3.35 mL, 24.0 mmol, 4.0 equiv) was added followed by dropwise addition of a solution of methanesulfonyl chloride (930 μL, 12.0 mmol, 2.0 equiv) in anhydrous CH_2Cl_2 (12 mL). The reaction mixture was stirred at –78 °C for 1.5 h and a solution of the crude amine and triethylamine (1.26 mL, 9.00 mmol, 1.5 equiv) in anhydrous CH_2Cl_2 (20 mL) was added slowly *via* syringe. The reaction mixture was allowed to warm to room temperature and stirred for 16 h. A saturated solution of NH_4Cl (50 mL) was added and stirred for 10 min. The layers were separated and the aqueous one extracted with CH_2Cl_2 (2 × 30 mL) and EtOAc (2 × 30 mL). The combined organic phases were washed with saturated aqueous $NaHCO_3$ solution (50 mL), brine (50 mL), dried over $MgSO_4$, filtered and concentrated under reduced pressure. The residue was purified by column chromatography (SiO_2, PE/EtOAc 1:1→EtOAc) to give **70** (2.79 g, 5.09 mmol, 85%) as an orange wax-like solid. $\mathbf{R_f}$ (EtOAc) 0.57; ^{1}**H NMR** (400 MHz, $CDCl_3$) δ_H ppm 7.46–7.29 (m, 11H, ArH, NH), 6.95 (d, *J* = 8.2 Hz, 2H, ArH), 6.85 (d, *J* = 8.2 Hz, 2H, ArH), 5.18 (d, *J* = 12.1 Hz, 1H, C*H*HPh), 5.12 (d, *J* = 12.1 Hz, 1H, CH*H*Ph), 5.03 (s, 2H, CH_2Ph), 4.79 (dd, *J* = 13.7, 6.0 Hz, 1H, CHα-Tyr), 4.70 (d, *J* = 13.7 Hz, 1H, C*H*HGOx), 4.63–4.57 (m, 2H, CH*H*GOx, OC*H*H-Ox), 4.48 (d, *J* = 7.6 Hz, 1H, OC*H*H-Ox), 4.36 (d, *J* = 7.3 Hz, 1H, OCH*H*-Ox), 4.28 (d, *J* = 7.6 Hz, 1H, OCH*H*-Ox), 3.49 (q, *J* = 6.9 Hz, 1H, CHα-Ala), 3.10 (dd, *J* = 14.2, 5.6 Hz, 1H, C*H*Hβ-Tyr), 3.02 (dd, *J* = 14.2, 6.8 Hz, 1H, CH*H*β-Tyr), 1.88 (br. s, 1H, NH), 1.26 (d, *J* = 7.0 Hz, 3H, CH_3β-Ala); ^{13}**C NMR** (101 MHz, $CDCl_3$) δ_C ppm δ 174.4 (C=O), 171.3 (C=O), 158.0 (C), 137.0 (C), 135.2 (C), 130.4 (CH), 128.78 (CH), 128.75 (CH), 128.70 (CH), 128.69 (CH), 128.6 (CH), 128.1 (C), 128.0 (CH), 127.6 (CH), 115.0 (CH), 79.4 (CH_2, GOx), 77.6 (OCH_2), 77.3 (OCH_2), 70.0 (CH_2, Bn), 67.4 (CH_2, Bn), 59.1 (C, Ox), 52.9 (CH, α-Tyr), 52.8 (CH, α-Ala), 36.9 (CH_2, β-Tyr), 20.7 (CH_3, β-Ala); **MS** (ESI^+) *m/z* 548 $[M+H]^+$, 570 $[M+Na]^+$, 586 $[M+K]^+$; **HRMS** (ESI^+) calcd. for $C_{30}H_{33}N_3NaO_7$ $[M+Na]^+$ 570.2211, found 570.2215; $[\boldsymbol{\alpha}]^{25}_{D}$ –9.6 (*c* 0.24, $CHCl_3$).

Boc-Ala-GOx-Ala-Tyr(Bn)-OBn (71)

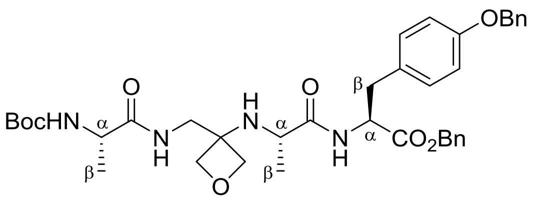

71

To a solution of NO_2-GOx-Ala-Tyr(Bn)-OBn (**70**) (2.66 g, 4.86 mmol, 1.0 equiv) in THF (48 mL) was added Boc-Ala-OSu (2.78 g, 9.71 mmol, 2.0 equiv), $NaHCO_3$ (1.63 g, 19.4 mmol, 4.0 equiv) and Raney Ni (slurry in H_2O, 10 mL). The solution was placed under an atmosphere of nitrogen, evacuated and filled with hydrogen (balloon). The reaction mixture was stirred vigorously for 4.0 h at room temperature. Then, the mixture was filtered through a plug of Celite eluting with EtOAc, the filtrate was washed with saturated Na_2CO_3 (3 × 50 mL) and concentrated under reduced pressure. Boc-Ala-GOx-Ala-Tyr(Bn)-OBn (**71**) was afforded after purification by column chromatography (SiO_2, EtOAc) as an off-white foam (1.96 g, 2.85 mmol, 59%). $\mathbf{R_f}$ (EtOAc) 0.34; **mp** 53–55 °C; ^{1}**H NMR** (400 MHz, $CDCl_3$) δ_H ppm 7.69 (d, *J* = 8.3 Hz, 1H, NH), 7.43–7.29 (m, 10H, ArH), 7.03 (br. s, 1H, NH), 6.95 (d, *J* = 7.8 Hz, 2H, ArH), 6.82 (d, *J* = 8.5 Hz, 2H, ArH), 5.25 (d, *J* = 12.0 Hz, 1H, C*H*HPh), 5.22 (br. s, 1H, NH), 5.13 (d, *J* = 12.0 Hz, 1H, CH*H*Ph), 5.01 (s, 2H, CH_2Ph), 4.90 (dt, *J* = 7.9, 5.8 Hz, 1H, CHα-Tyr), 4.44 (d, *J* = 6.9 Hz, 1H, OC*H*H-Ox), 4.34 (d, *J* = 6.9 Hz, 1H, OCH*H*-Ox), 4.33 (d, *J* = 6.8 Hz, 1H, OC*H*H-Ox), 4.30 (d, *J* = 6.8 Hz, 1H, OCH*H*-Ox), 4.21–4.12 (m, 1H, CHα-Ala), 3.88 (dd, *J* = 14.0, 7.7 Hz, 1H, C*H*HGOx), 3.27 (q, *J* = 6.7 Hz, 1H, CHα-Ala), 3.22–3.11 (m, 2H, CH*H*GOx, C*H*Hβ-Tyr), 3.01 (dd, *J* = 14.1, 7.6 Hz, 1H, CH*H*β-Tyr), 2.04 (br. s, 1H, NH), 1.43 (s, 9H, 3 × CH_3, Boc), 1.26 (d, *J* = 6.4 Hz, 3H, CH_3β-Ala), 1.20 (d, *J* = 6.9 Hz, 3H, CH_3β-Ala); ^{13}**C NMR** (101 MHz, $CDCl_3$) δ_C ppm 175.2 (C=O), 174.2 (C=O), 172.8 (C=O), 157.9 (C), 155.5 (C=O, Boc), 137.0 (C), 134.9 (C), 130.3 (CH), 128.8 (CH), 128.7 (CH), 128.2 (C), 128.1 (CH), 127.5 (CH), 115.0 (CH), 79.9 (C, Boc), 79.5 (OCH_2), 78.9 (OCH_2), 70.0 (CH_2, Bn), 67.7 (CH_2, Bn), 61.0 (C, Ox), 53.3 (CH, α-

Ala), 52.7 (CH, α-Tyr), 50.2 (CH, α-Ala), 43.9 (CH_2, GOx), 37.0 (CH_2, β-Tyr), 28.4 (CH_3, Boc), 21.3 (CH_3, β-Ala), 18.4 (CH_3, β-Ala). *N.B.* Two aromatic CH signals not observed; **ν_{max}** (neat) = 3308, 2974, 1657, 1510, 1453, 1366, 1241, 1164, 1021, 972, 735, 696 cm^{-1}; **MS** (ESI^+) *m/z* 689 $[M+H]^+$, 711 $[M+Na]^+$, 727 $[M+K]^+$; **HRMS** (ESI^+) calcd. for $C_{38}H_{48}N_4NaO_8$ $[M+Na]^+$ 711.3364, found 711.3367; $[\boldsymbol{\alpha}]_D^{27}$ –8.8 (*c* 1.18, $CHCl_3$).

Cbz-Leu-Ala-GOx-Ala-Tyr(Bn)-OBn (72)

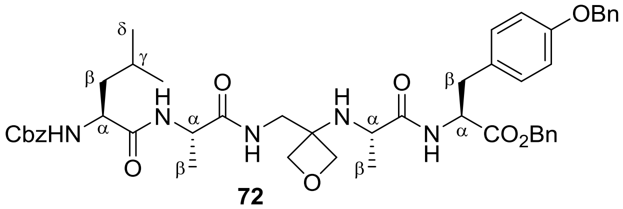

72

To a solution of Boc-Ala-GOx-Ala-Tyr(Bn)-OBn (**71**) (1.81 g, 2.63 mmol, 1.0 equiv) in CH_2Cl_2 (3.0 mL) was added TFA (3.0 mL) and the mixture was stirred at room temperature for 10 min. The mixture was concentrated under reduced pressure and the resulting residue repeatedly dissolved in CH_2Cl_2 (3 × 10 mL) and concentrated *in vacuo* to give the crude amine. The residue was dissolved in a mixture of CH_2Cl_2 (26 mL) and DMF (5.0 mL), Cbz-Leu-OH (837 mg, 3.15 mmol, 1.2 equiv), HATU (1.20 g, 3.15 mmol, 1.2 equiv) and DIPEA (1.83 mL, 10.5 mmol, 4.0 equiv) were added subsequently, and the reaction mixture was stirred at room temperature for 16 h. The mixture was diluted with CH_2Cl_2 (30 mL) and washed with 10% citric acid solution (2 × 50 mL) and saturated $NaHCO_3$ solution (2 × 50 mL), dried over $MgSO_4$, filtered and concentrated under reduced pressure. The residue was purified by flash column chromatography (SiO_2, CH_2Cl_2/MeOH 98:2→97:3→96:4) to give pentapeptide **72** (1.21 g, 1.45 mmol, 55%) as a white foam. **R_f** (CH_2Cl_2/MeOH 9:1) 0.33; **mp** 59–61 °C; **^{1}H NMR** (400 MHz, CD_3OD) δ_H ppm 7.41 (d, *J* = 7.5 Hz, 2H, ArH), 7.38–7.26 (m, 13H, ArH), 7.07 (d, *J* = 8.2 Hz, 2H, ArH), 6.86 (d, *J* = 8.2 Hz, 2H, ArH), 5.18 (d, *J* = 12.2 Hz, 1H, C*H*HPh), 5.15–5.04 (m, 3H, CH*H*Ph, CH_2Ph), 5.02 (s, 2H, CH_2Ph), 4.73 (dd, *J* = 9.2, 5.1 Hz, 1H, CHα-Tyr), 4.41 (d, *J* = 6.4 Hz, 1H, OC*H*H-Ox), 4.31 (q, *J* = 7.1 Hz, 1H, CHα-Ala), 4.27 (d, *J* = 6.8 Hz, 1H, OC*H*H-Ox), 4.22–4.11 (m, 3H, 2 × OCH*H*-Ox, CHα-Leu), 3.50 (d, *J* = 14.1 Hz, 1H, C*H*HGOx), 3.37–3.31 (m, 1H, CHα-Ala), 3.21 (d, *J* = 14.1 Hz, 1H, CH*H*GOx), 3.15 (dd, *J* = 13.8, 4.8 Hz, 1H, C*H*Hβ-Tyr), 2.94 (dd, *J* = 13.8, 9.4 Hz, 1H, CH*H*β-Tyr), 1.71 (nonet, *J* = 6.6 Hz, 1H, CHγ-Leu), 1.62–1.50 (m, 2H, CH_2β-Leu), 1.32 (d, *J* = 7.1 Hz, 3H, CH_3β-Ala), 1.15 (d, *J* = 6.9 Hz, 3H, CH_3β-Ala), 0.94 (d, *J* = 7.2 Hz, 3H, CH_3δ-Leu), 0.92 (d, *J* = 7.4 Hz, 3H, CH_3δ-Leu); **^{13}C NMR** (101 MHz, CD_3OD) δ_C ppm 178.6 (C=O), 175.4 (C=O), 175.3 (C=O), 172.9 (C=O), 159.3 (C=O), 158.7 (C), 138.8 (C), 138.1 (C), 136.0 (C), 131.4 (CH), 130.2 (C), 129.61 (CH), 129.56 (CH), 129.50 (CH), 129.46 (CH), 129.0 (CH), 128.83 (CH), 128.79 (CH), 128.6 (CH), 116.0 (CH), 80.1 (OCH_2), 80.0 (OCH_2), 71.0 (CH_2, Bn), 68.2 (CH_2, Bn), 67.8 (CH_2, Bn), 61.2 (C, Ox), 55.1 (CH, α-Leu), 54.8 (CH, α-Tyr), 53.5 (CH, α-Ala), 50.7 (CH, α-Ala), 44.7 (CH_2, GOx), 41.9 (CH_2, β-Leu), 37.4 (CH_2, β-Tyr), 25.9 (CH, γ-Leu), 23.5 (CH_3, δ-Leu), 21.8 (CH_3, δ-Leu), 20.9 (CH_3, β-Ala), 17.8 (CH_3, β-Ala). *N.B.* One aromatic CH signal not observed; **ν_{max}** (neat) = 3295, 1712, 1650, 1537, 1493, 1214, 1121, 969, 744, 700 cm^{-1}; **MS** (ESI^+) *m/z* 836 $[M+H]^+$, 858 $[M+Na]^+$, 874 $[M+K]^+$; **HRMS** (ESI^+) calcd. for $C_{47}H_{57}N_5NaO_9$ $[M+Na]^+$ 858.4048, found 858.4043; $[\boldsymbol{\alpha}]_D^{26}$ –19.1 (*c* 0.25, $CHCl_3$).

H-Leu-Ala-GOx-Ala-Tyr-OH (73)

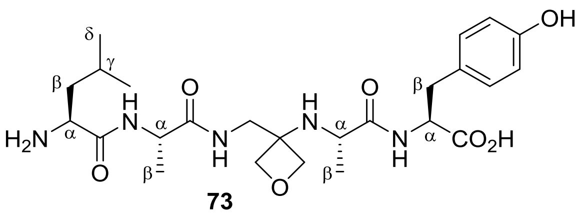

73

To a solution of pentapeptide **72** (1.14 g, 1.36 mmol) in anhydrous MeOH (14 mL) was added 10 wt% Pd/C (114 mg, 10 wt%) and the reaction flask was evacuated, filled with nitrogen, evacuated, and placed under an atmosphere of H_2 (balloon). The reaction mixture was stirred at room temperature for 16 h, placed under N_2 and filtered through a plug of Celite, which was washed with MeOH (3×). The filtrate was concentrated *in vacuo* to give **73** as an off-white solid (742 mg) in quantitative yield. **mp** 170–172 °C; **^{1}H NMR** (400 MHz, CD_3OD) δ_H ppm 7.02 (d, *J* = 8.4 Hz, 2H, ArH), 6.66 (d, *J* = 8.4 Hz, 2H, ArH), 4.48–4.35 (m, 5H, OC*H*H-Ox, CHα-Tyr, CHα-Ala, OCH_2-Ox), 4.31 (d, *J* = 6.5 Hz, 1H, OCH*H*-Ox), 3.92 (t, *J* = 7.0 Hz, 1H, CHα-Leu), 3.67 (d, *J* = 14.1 Hz, 1H, C*H*HGOx), 3.28–3.20 (m, 2H, CH*H*GOx, CHα-Ala), 3.10 (dd, *J* = 14.1, 4.7 Hz, 1H, C*H*Hβ-Tyr), 2.98 (dd, *J* = 14.1, 6.3 Hz, 1H, CH*H*β-Tyr), 1.78–1.58 (m, 3H, CH_2β-Leu, CHγ-Leu), 1.29 (d, *J* = 7.1 Hz, 3H, CH_3β-Ala), 1.25 (d, *J* = 7.0 Hz, 3H, CH_3β-Ala), 0.98 (d, *J* = 5.8 Hz, 3H, CH_3δ-Leu), 0.97 (d, *J* = 5.9 Hz, 3H, CH_3δ-Leu); **^{13}C NMR** (101 MHz, CD_3OD) δ_C ppm 177.8 (C=O), 177.6 (C=O), 175.7 (C=O), 170.5 (C=O), 157.1 (C), 131.6 (CH), 129.8 (C), 116.0 (CH), 80.5 (OCH_2), 80.3 (OCH_2), 61.6 (C, Ox), 56.2 (CH, α-Tyr), 54.3 (CH, α-Ala), 53.1 (CH, α-Leu), 51.3 (CH, α-Ala), 45.2 (CH_2, GOx), 41.6 (CH_2, β-Leu), 38.3 (CH_2, β-Tyr), 25.5 (CH_3, γ-Leu), 23.1 (CH_3, δ-Leu), 22.3 (CH_3, δ-Leu), 21.0 (CH_3, β-Ala), 18.0 (CH_3, β-Ala); **ν_{max}** (neat) = 3272, 2961, 1649, 1513, 1388, 1236, 1186, 968, 828 cm^{-1}; **MS** (ESI^+) *m/z* 522 $[M+H]^+$, 544 $[M+Na]^+$, 560 $[M+K]^+$; **HRMS** (ESI^+) calcd. for $C_{25}H_{40}N_5O_7$ $[M+H]^+$ 522.2922, found 522.2921; $[\alpha]_D^{24}$ +4.5 (*c* 0.20, MeOH).

*Preparation of pentapeptide **80***

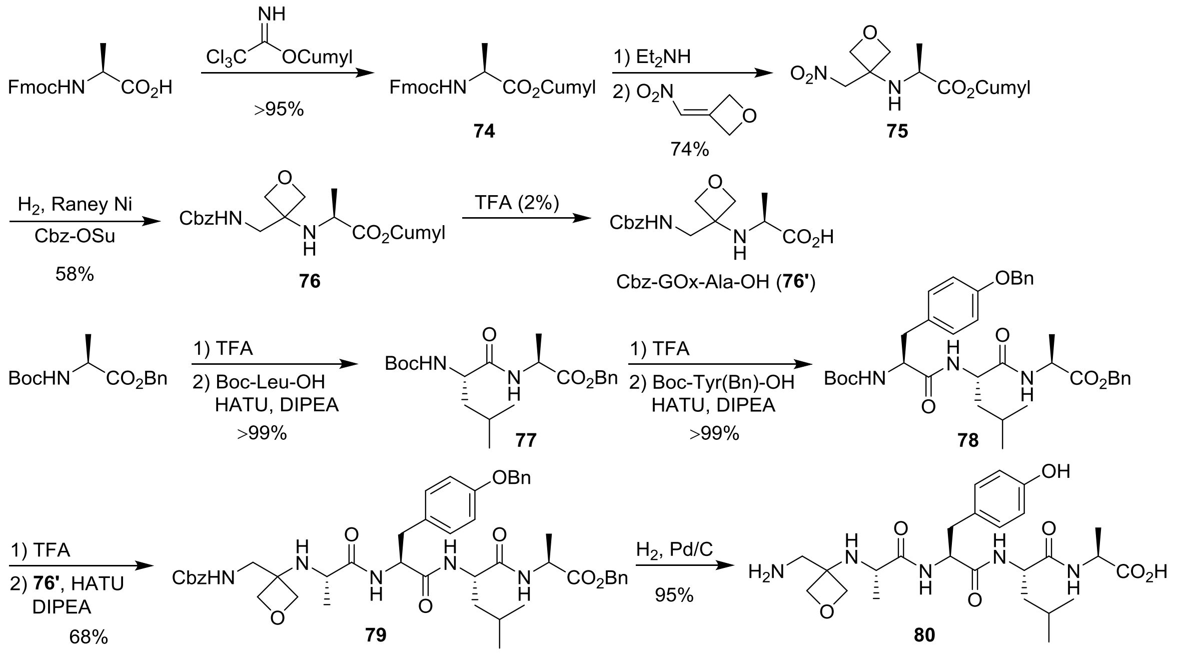

Fmoc-Ala-OCumyl (74)

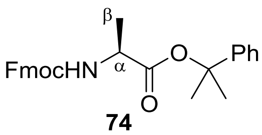

74

To sodium hydride (60% dispersion in mineral oil, 280 mg, 7.00 mmol, 0.5 equiv) in anhydrous diethyl ether (28 mL) was added freshly distilled 2-phenyl-2-propanol (4.20 g, 30.8 mmol, 2.2 equiv) at 0 °C and the mixture was stirred for 1 h at room temperature. The reaction mixture was cooled to 0 °C, 2,2,2-trichloroacetonitrile (2.80 mL, 28.0 mmol, 2.0 equiv) were added slowly and stirring was continued for 3 h at ambient temperature. The solvent was removed under reduced pressure and the residue re-dissolved in PE (7.0 mL), anhydrous MeOH (283 µL, 7.00 mmol, 0.5 equiv) was added and the solution was stirred for 10 min at room temperature. The mixture was filtered through a plug of Celite eluting with PE and the filtrate was concentrated *in vacuo* to give the crude imidate. To a suspension of Fmoc-Ala-OH (4.36 g, 14.0 mmol, 1.0 equiv) in CH_2Cl_2 (80 mL) was added a solution of the imidate in CH_2Cl_2 (15 mL) and the mixture was stirred for 16 h at room temperature. The reaction mixture was filtered through a plug of Celite eluting with CH_2Cl_2, the solvent was removed *in vacuo*, and the residue was purified by column chromatograph (SiO_2, PE/EtOAc 4:1) to give Fmoc-Ala-OCumyl (**74**) (6.00 g, 14.0 mmol, quant. yield) contaminated with small amounts of 2-phenyl-2-propanol (~85:15 by ^{1}H NMR) as a pale-yellow oil. **R$_f$** (PE/EtOAc 4:1) 0.31; **^{1}H NMR** (400 MHz, $CDCl_3$) δ_H ppm 7.76 (d, *J* = 7.5 Hz, 2H, ArH), 7.59 (d, *J* = 7.5 Hz, 2H, ArH), 7.43–7.25 (m, 9H, ArH), 5.35 (d, *J* = 7.1 Hz, 1H, NH), 4.44–4.34 (m, 3H, CHα-Ala, CH_2-Fmoc), 4.21 (t, *J* = 7.0 Hz, 1H, CH-Fmoc), 1.83 (s, 3H, CH_3, cumyl), 1.79 (s, 3H, CH_3, cumyl), 1.47 (d, *J* = 7.0 Hz, 3H, CH_2β-Ala); **^{13}C NMR** (101 MHz, $CDCl_3$) δ_C ppm 171.7 (C=O), 155.7 (C=O, Fmoc), 145.2 (C), 145.0 (C), 144.1 (C), 143.9 (C), 141.4 (C), 128.3 (CH), 127.8 (CH), 127.5 (CH), 127.2 (CH), 125.3 (CH), 124.3 (CH), 120.1 (CH), 83.3 (C, cumyl), 67.1 (CH_2, Fmoc), 50.1 (CH, α-Ala), 47.3 (CH, Fmoc), 28.9 (CH_3, cumyl), 28.2 (CH_3, cumyl), 19.0 (CH_3, β-Ala); **ν_{max}** (neat) = 3330, 2979, 1702, 1512, 1448, 1249, 1137, 1071, 951, 759, 737, 698 cm^{-1}; **MS** (ESI$^+$) *m/z* 452 $[M+Na]^+$, 467 $[M+K]^+$; **HRMS** (ESI$^+$) calcd. for $C_{27}H_{27}NNaO_4$ $[M+Na]^+$ 452.1832, found 452.1834; $[\alpha]_D^{27}$ –7.7 (*c* 1.00, $CHCl_3$).

O_2N-GOx-Ala-OCumyl (75)

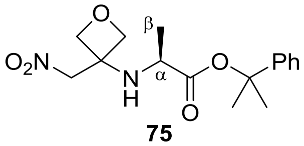

75

To a solution of Fmoc-Ala-OCumyl (**74**) (3.70 g, 8.60 mmol, 1.0 equiv) in CH_2Cl_2 (9.0 mL) was added diethylamine (9.0 mL) and the mixture was stirred at room temperature for 1 h. The reaction mixture was concentrated under reduced pressure and the resulting residue repeatedly dissolved in CH_2Cl_2 (3 × 15 mL) and concentrated under reduced pressure to give the crude amine. In a second reaction vessel, oxetane-3-one (1.10 mL, 17.2 mmol, 2.0 equiv), nitromethane (1.30 mL, 24.1 mmol, 2.8 equiv) and triethylamine (480 µL, 3.44 mmol, 0.4 equiv) were combined at 0 °C and stirred for 1 h at room temperature. The mixture was dissolved in anhydrous CH_2Cl_2 (60 mL), cooled to –78 °C, and triethylamine (4.80 mL, 34.4 mmol, 4.0 equiv) was added followed by dropwise addition of a solution of methanesulfonyl chloride (1.33 mL, 17.2 mmol, 2.0 equiv) in anhydrous CH_2Cl_2 (18 mL). The reaction mixture was stirred at –78 °C for 1.5 h and the solution of the crude amine in anhydrous CH_2Cl_2 (30 mL) was added slowly *via* syringe. The reaction mixture was allowed to warm to room temperature and stirred for 16 h. A saturated solution of NH_4Cl (50 mL) was added and stirred for 10 min. The layers were separated and the aqueous one extracted with CH_2Cl_2 (2 × 30 mL) and EtOAc (2 × 30 mL). The combined organic phases were washed with saturated aqueous $NaHCO_3$ solution (50 mL), brine (50 mL), dried over $MgSO_4$, filtered, and concentrated *in vacuo*. The residue was purified by column chromatography (SiO_2, PE/EtOAc 2:1→1:1) to give **75** (2.04 g, 6.33 mmol, 74%) as a pale-yellow oil. **R$_f$** (PE/EtOAc 1:1) 0.39; **^{1}H NMR** (400 MHz, $CDCl_3$) δ_H ppm 7.36–7.31 (m, 4H, ArH), 7.29–7.26 (m, 1H, ArH), 4.80 (d, *J* = 12.6 Hz, 1H, C*H*HGOx), 4.76 (d, *J* = 12.6 Hz, 1H, CH*H*GOx), 4.57 (d, *J* = 7.0 Hz, 1H, OC*H*H-Ox), 4.56 (d, *J* = 6.8 Hz, 1H, OC*H*H-Ox), 4.48 (d,

J = 7.0 Hz, 1H, OCH*H*-Ox), 4.38 (d, J = 6.8 Hz, 1H, OCH*H*-Ox), 3.57 (q, J = 6.8 Hz, 1H, CHα-Ala), 2.33 (s, 1H, NH), 1.79 (s, 6H, 2 × CH_3, cumyl), 1.32 (d, J = 7.0 Hz, 3H, CH_3β-Ala); **^{13}C NMR** (101 MHz, $CDCl_3$) δ_C ppm 174.5 (C=O), 145.0 (C), 128.5 (CH), 127.6 (CH), 124.4 (CH), 82.9 (C, cumyl), 79.1 (OCH_2), 79.0 (OCH_2), 78.8 (CH_2, GOx), 59.7 (C, Ox), 51.9 (CH, α-Ala), 28.39 (CH_3, cumyl), 28.37 (CH_3, cumyl), 20.7 (CH_3, β-Ala); **ν_{max}** (neat) = 3336, 2980, 1728, 1552, 1448, 1378, 1272, 1199, 1332, 1101, 976, 831, 764, 699 cm^{-1}; **MS** (ESI^+) *m/z* 345 $[M+Na]^+$, 361 $[M+K]^+$; **HRMS** (ESI^+) calcd. for $C_{16}H_{22}N_2NaO_5$ $[M+Na]^+$ 345.1421 found 345.1414; $[\alpha]_D^{27}$ –9.4 (c 0.88, $CHCl_3$).

Cbz-GOx-Ala-OCumyl (76)

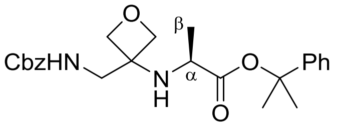

76

To a solution of NO_2-GOx-Ala-OCumyl (**75**) (2.04 g, 6.32 mmol, 1.0 equiv) in THF (65 mL) was added *N*-(benzyloxycarbonyloxy) succinimide (3.15 g, 12.6 mmol, 2.0 equiv), $NaHCO_3$ (2.12 g, 25.2 mmol, 4.0 equiv) and Raney Ni (slurry in H_2O, 6.3 mL). The mixture was placed under an atmosphere of nitrogen, evacuated and filled with hydrogen (balloon). The reaction mixture was stirred vigorously for 2.5 h at room temperature. Then, the mixture was filtered through a plug of Celite eluting with EtOAc, the filtrate was washed with sat. Na_2CO_3 (3 × 50 mL) and concentrated under reduced pressure. Cbz-GOx-Ala-OCumyl (**76**) was afforded after purification by column chromatography (SiO_2, PE/EtOAc 2:1→1:1→EtOAc) as a pale yellow oil (1.55 g, 3.63 mmol, 58%). **R_f** (PE/EtOAc 1:1) 0.19; **^{1}H NMR** (400 MHz, $CDCl_3$) δ_H ppm 7.38–7.29 (m, 9H, ArH), 7.26–7.21 (m, 1H, ArH), 5.20 (br. s, 1H, NH), 5.10 (s, 2H, CH_2Ph), 4.40 (d, J = 6.6 Hz, 1H, OC*H*H-Ox), 4.34 (d, J = 6.6 Hz, 1H, OCH*H*-Ox), 4.29 (d, J = 5.6 Hz, 1H, OC*H*H-Ox), 4.28 (d, J = 5.6 Hz, 1H, OCH*H*-Ox), 3.69 (dd, J = 13.7, 6.2 Hz, 1H, C*H*HGOx), 3.46–3.36 (m, 2H, CH*H*GOx, CHα-Ala), 1.77 (d, J = 4.9 Hz, 6H, 2 × CH_3, cumyl), 1.76–1.60 (br. s, 1H, NH), 1.31 (d, J = 7.0 Hz, 3H, CH_3β-Ala); **^{13}C NMR** (101 MHz, $CDCl_3$) δ_C ppm 175.2 (C=O), 156.9 (C=O, Fmoc), 145.0 (C), 136.6 (C), 128.7 (CH), 128.5 (CH), 128.3 (CH), 127.5 (CH), 124.4 (CH), 82.8 (C, cumyl), 80.0 (OCH_2), 79.8 (OCH_2), 67.0 (CH_2Ph), 59.5 (C, Ox), 51.6 (CH, α-Ala), 45.5 (CH_2, GOx), 28.5 (CH_3, cumyl), 28.3 (CH_3, cumyl), 20.9 (CH_3, β-Ala). *N.B.* One aromatic CH signal not observed; **ν_{max}** (neat) = 3329, 2977, 1717, 1515, 1450, 1277, 1130, 1101, 973, 763, 697 cm^{-1}; **MS** (ESI^+) *m/z* 427 $[M+H]^+$, 449 $[M+Na]^+$; **HRMS** (ESI^+) calcd. for $C_{24}H_{31}N_2O_5$ $[M+H]^+$ 427.2227, found 427.2229; $[\alpha]_D^{27}$ –9.4 (c 0.77, $CHCl_3$).

Boc-Leu-Ala-OBn (77)

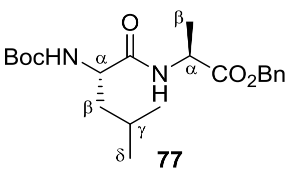

77

To a solution of Boc-Ala-OBn[1] (4.56 g, 16.3 mmol, 1.0 equiv) in CH_2Cl_2 (16 mL) was added TFA (16 mL) and the mixture was stirred at room temperature for 1 h. The reaction mixture was concentrated under reduced pressure and the resulting residue repeatedly dissolved in CH_2Cl_2 (3 × 20 mL) and concentrated under reduced pressure to give the crude amine. The residue was dissolved in CH_2Cl_2 (160 mL), Boc-Leu-OH (4.53 g, 19.6 mmol, 1.2 equiv), HATU (7.45 g, 19.6 mmol, 1.2 equiv) and DIPEA (11.4 mL, 65.2 mmol, 4.0 equiv) were added subsequently, and the reaction mixture was stirred at room temperature for 16 h. The reaction mixture was diluted with CH_2Cl_2 (50 mL) and washed with 10% citric acid solution (2 × 100 mL) and sat. $NaHCO_3$ solution (2 × 100 mL), dried over $MgSO_4$, filtered and concentrated under reduced pressure. The residue was purified by flash column chromatography (SiO_2, PE/EtOAc 2:1→1:1) to give dipeptide **77** (6.40 g, 16.3 mmol, >99%) as a colourless viscous oil. **R_f** (PE/EtOAc 1:1) 0.53; **^{1}H NMR** (400 MHz, $CDCl_3$) δ_H ppm 7.39–7.30 (m, 5H, ArH), 6.60 (br. m, 1H, NH), 5.19 (d, J = 12.3 Hz, 1H, C*H*HPh), 5.14 (d, J = 12.3 Hz, 1H, CH*H*Ph), 4.88 (br. s, 1H, NH), 4.61 (quint, J = 7.2 Hz, 1H, CHα-Ala), 4.11 (br. m, 1H, CHα-Leu), 1.71–1.60 (m,

2H, CH_2β-Leu), 1.50–1.44 (m, 1H, CHγ-Leu), 1.43 (s, 9H, 3 × CH_3, Boc), 1.41 (d, *J* = 7.3 Hz, 3H, CH_3β-Ala), 0.92 (d, *J* = 6.1 Hz, 6H, 2 × CH_3δ-Leu); **^{13}C NMR** (101 MHz, $CDCl_3$) δ_C ppm δ 172.7 (C=O), 172.3 (C=O), 155.8 (C=O, Boc), 135.5 (C), 128.7 (CH), 128.5 (CH), 128.3 (CH), 80.2 (C, Boc), 67.2 (CH_2, Bn), 53.0 (CH, α-Leu), 48.2 (CH, α-Ala), 41.5 (CH_2, β-Leu), 28.4 (CH_3, Boc), 24.8 (CH, γ-Leu), 23.0 (CH_3, δ-Leu), 22.1 (CH_3, δ-Leu), 18.3 (CH_3, β-Ala); **ν_{max}** (neat) = 3294, 2957, 1744, 1656, 1527, 1454, 1366, 1317, 1160, 1046, 751, 697 cm^{-1}; **MS** (ESI^+) *m/z* 415 $[M+Na]^+$, 431 $[M+K]^+$; **HRMS** (ESI^+) calcd. for $C_{21}H_{32}N_2NaO_5$ $[M+Na]^+$ 415.2203, found 415.2203; $[\alpha]_D^{25}$ –29.8 (*c* 0.91, $CHCl_3$).

Boc-Tyr(Bn)-Leu-Ala-OBn (78)

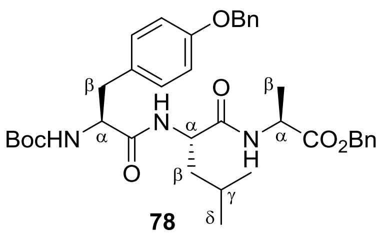

78

To a solution of Boc-Leu-Ala-OBn (**77**) (5.85 g, 14.9 mmol, 1.0 equiv) in CH_2Cl_2 (15 mL) was added TFA (15 mL) and the mixture was stirred at room temperature for 1 h. The reaction mixture was concentrated under reduced pressure and the resulting residue repeatedly dissolved in CH_2Cl_2 (3 x 20 mL) and concentrated under reduced pressure to give the crude amine. The residue was dissolved in CH_2Cl_2 (150 mL), Boc-Tyr(Bn)-OH (6.40 g, 17.2 mmol, 1.2 equiv), HATU (6.54 g, 17.2 mmol, 1.2 equiv) and DIPEA (10.4 mL, 59.6 mmol, 4.0 equiv) were added subsequently, and the reaction mixture was stirred at room temperature for 16 h. The mixture was diluted with CH_2Cl_2 (50 mL) and washed with 10% citric acid solution (2 × 100 mL) and saturated $NaHCO_3$ solution (2 × 100 mL), dried over $MgSO_4$, filtered and concentrated under reduced pressure. The residue was purified by flash column chromatography (SiO_2, PE/EtOAc 2:1→1:1) to give tripeptide **78** (9.60 g, 14.9 mmol, >99%) as a white solid. **R_f** (PE/EtOAc 1:1) 0.49; **mp** 59–61 °C; **^{1}H NMR** (400 MHz, $CDCl_3$) δ_H ppm 7.43–7.29 (m, 10H, ArH), 7.10 (d, *J* = 8.4 Hz, 2H, ArH), 6.88 (d, *J* = 8.4 Hz, 2H, ArH), 6.68 (br. s, 1H, NH), 6.43 (t, *J* = 8.8 Hz, 1H, NH), 5.19 (d, *J* = 12.3 Hz, 1H, C*H*HPh), 5.13 (d, *J* = 12.3 Hz, 1H, CH*H*Ph), 5.01 (s, 2H, CH_2Ph), 4.98 (br. s, 1H, NH), 4.55 (quint, *J* = 7.2 Hz, 1H, CHα-Ala), 4.48–4.39 (m, 1H, CHα-Leu), 4.35–4.24 (m, 1H, CHα-Tyr), 3.02 (dd, *J* = 13.5, 6.3 Hz, 1H, C*H*Hβ-Tyr), 2.97 (dd, *J* = 13.5, 6.5 Hz, 1H, CH*H*β-Tyr), 1.81 (br. s, 1H, NH), 1.66–1.42 (m, 3H, CH_2β-Leu, CHγ-Leu), 1.40 (s, 9H, 3 × CH_3, Boc), 1.38 (d, *J* = 7.7 Hz, 3H, CH_3β-Ala), 0.88 (d, *J* = 6.3 Hz, 6H, 2 × CH_3δ-Leu); **^{13}C NMR** (101 MHz, $CDCl_3$) δ_C ppm 172.5 (C=O), 171.5 (C=O), 171.3 (C=O), 158.0 (C), 155.7 (C=O, Boc), 137.1 (C), 135.5 (C), 130.5 (CH), 130.2 (C), 128.73 (CH), 128.72 (CH), 128.5 (CH), 128.3 (CH), 128.1 (CH), 127.6 (CH), 115.2 (CH), 80.5 (C, Boc), 70.1 (CH_2, Bn), 67.3 (CH_2, Bn), 56.0 (CH, α-Tyr), 51.7 (CH, α-Leu), 48.3 (CH, α-Ala), 41.1 (CH_2, β-Ala), 37.1 (CH_2, β-Tyr), 28.4 (CH_3, Boc), 24.6 (CH, γ-Leu), 23.0 (CH_3, δ-Leu), 22.1 (CH_3, δ-Leu), 18.1 (CH_3, β-Ala); **ν_{max}** (neat) = 3273, 2957, 1643, 1510, 1454, 1366, 1237, 1159, 1020, 735, 695 cm^{-1}; **MS** (ESI^+) *m/z* 668 $[M+Na]^+$, 684 $[M+K]^+$; **HRMS** (ESI^+) calcd. for $C_{37}H_{47}N_3NaO_7$ $[M+Na]^+$ 668.3306, found 668.3311; $[\alpha]_D^{26}$ –16.4 (*c* 0.50, $CHCl_3$).

Cbz-GOx-Ala-Tyr(Bn)-Leu-Ala-OBn (79)

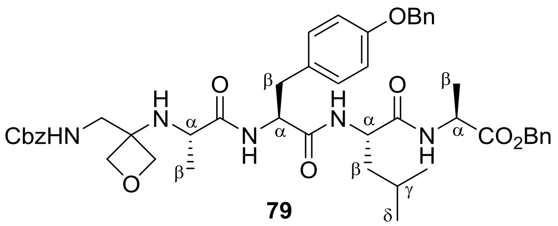

79

Cbz-GOx-Ala-OCumyl (**76**) (853 mg, 2.00 mmol, 1.0 equiv) was stirred in 2% TFA/CH_2Cl_2 (40 mL) at room temperature for 90 min. The reaction mixture was concentrated under reduced pressure and the resulting residue repeatedly dissolved in CH_2Cl_2 (3 × 20 mL) and concentrated under reduced pressure to give the crude acid **76'**. In a separate reaction flask Boc-Tyr(Bn)-Leu-Ala-OBn (**78**) (1.29 g, 2.00 mmol, 1.0 equiv) was dissolved in CH_2Cl_2 (2.0 mL), TFA (2.0 mL) was added and the mixture was stirred at

room temperature for 1 h. The reaction mixture was concentrated under reduced pressure and the resulting residue repeatedly dissolved in CH_2Cl_2 (3 × 20 mL) and concentrated under reduced pressure to give the crude amine. The residue was dissolved in a mixture of CH_2Cl_2 (20 mL) and DMF (1.0 mL) and added to the crude acid. HATU (760 mg, 2.00 mmol, 1.0 equiv) and DIPEA (1.39 mL, 8.00 mmol, 4.0 equiv) were added subsequently, and the reaction mixture was stirred at room temperature for 16 h. The reaction mixture was diluted with EtOAc (50 mL), washed with brine (2 × 50 mL), 1.0 M HCl (3 × 50 mL), saturated $NaHCO_3$ solution (3 × 50 mL), brine (50 mL), dried over $MgSO_4$, filtered and concentrated under reduced pressure. The residue was purified by flash column chromatography (SiO_2, EtOAc) to give pentapeptide **79** (1.15 g, 1.37 mmol, 68%) as a white foam. **R_f** (CH_2Cl_2/MeOH 96:4) 0.31; **mp** 75–77 °C; **^{1}H NMR** (400 MHz, CD_3OD) δ_H ppm 7.44–7.24 (m, 15H, ArH), 7.11 (d, *J* = 8.4 Hz, 2H, ArH), 6.86 (d, *J* = 8.4 Hz, 2H, ArH), 5.16 (d, *J* = 12.6 Hz, 1H, C*H*HPh), 5.11 (d, *J* = 12.6 Hz, 1H, CH*H*Ph), 5.09 (s, 2H, CH_2Ph), 5.01 (s, 2H, CH_2Ph), 4.60 (dd, *J* = 9.5, 5.1 Hz, 1H, CHα-Tyr), 4.46–4.37 (m, 3H, OC*H*H-Ox, CHα-Ala, CHα-Leu), 4.26 (s, 1H, OCH_2-Ox), 4.16 (d, *J* = 6.5 Hz, 1H, OCH*H*-Ox), 3.42 (d, *J* = 14.2 Hz, 1H, C*H*HGOx), 3.37–3.32 (m, 1H, CHα-Ala), 3.22 (d, *J* = 14.2 Hz, 1H, CH*H*GOx), 3.05 (dd, *J* = 14.0, 5.0 Hz, 1H, C*H*Hβ-Tyr), 2.78 (dd, *J* = 13.9, 9.7 Hz, 1H, CH*H*β-Tyr), 1.62 (nonet, *J* = 6.8 Hz, 1H, CHγ-Leu), 1.51 (t, *J* = 7.3 Hz, 2H, CH_2β-Leu), 1.37 (d, *J* = 7.3 Hz, 3H, CH_3β-Ala), 1.14 (d, *J* = 6.8 Hz, 3H, CH_3β-Ala), 0.88 (d, *J* = 6.6 Hz, 1H, CH_3δ-Leu), 0.85 (d, *J* = 6.5 Hz, 1H, CH_3δ-Leu); **^{13}C NMR** (101 MHz, CD_3OD) δ_C 178.3 (C=O), 174.3 (C=O), 173.8 (C=O), 173.3 (C=O), 159.2 (C), 157.0 (C=O, Cbz), 138.8 (C), 138.3 (C), 137.2 (C), 131.4 (CH), 130.4 (CH), 129.6 (CH), 129.5 (CH), 129.4 (CH), 129.3 (C), 129.0 (CH), 128.9 (CH), 128.8 (CH), 128.6 (CH), 115.9 (CH), 80.1 (OCH_2), 79.9 (OCH_2), 71.0 (CH_2, Bn), 68.0 (CH_2, Bn), 67.5 (CH_2, Bn), 61.4 (C, Ox), 55.4 (CH, α-Tyr), 53.4 (CH, α-Ala), 52.7 (CH, α-Leu), 49.6 (CH, α-Ala), 46.4 (CH_2, GOx), 42.2 (CH_2, β-Leu), 38.2 (CH_2, β-Tyr), 25.7 (CH, γ-Leu), 23.4 (CH_3, δ-Leu), 22.1 (CH_3, δ-Leu), 20.7 (CH_3, β-Ala), 17.3 (CH_3, β-Ala). *N.B.* One aromatic CH signal not observed; **ν_{max}** (neat) = 3281, 2955, 1638, 1543, 1510, 1453, 1235, 1156, 1024, 971, 735, 695 cm^{-1}; **MS** (ESI$^+$) *m/z* 836 [M+H]$^+$, 858 [M+Na]$^+$, 874 [M+K]$^+$; **HRMS** (ESI$^+$) calcd. for $C_{47}H_{57}N_5NaO_9$ [M+Na]$^+$ 858.4048, found 858.4050; $[\alpha]_D^{27}$ –6.8 (*c* 0.3, $CHCl_3$).

H-GOx-Ala-Tyr-Leu-Ala-OH (80)

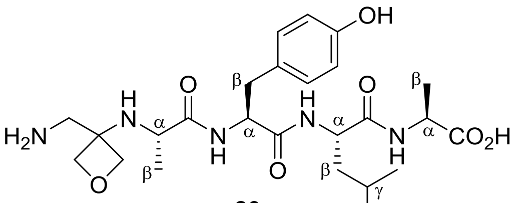

80

To a solution of pentapeptide **79** (316 mg, 0.38 mmol) in anhydrous MeOH (4.0 mL) was added 10 wt% Pd/C (32 mg, 10 wt%) and the reaction flask was evacuated, filled with nitrogen, evacuated, and placed under an atmosphere of hydrogen (balloon). The reaction mixture was stirred at room temperature for 5 h, placed under nitrogen and filtered through a plug of Celite, which was washed with MeOH (3×). The filtrate was concentrated *in vacuo* to give **80** as an off-white solid (189 mg, 0.36 mmol, 95%), which required no further purification. **mp** 165–168 °C; **^{1}H NMR** (400 MHz, CD_3OD) δ_H ppm 7.12 (d, *J* = 8.3 Hz, 2H, ArH), 6.72 (d, *J* = 8.3 Hz, 2H, ArH), 4.66 (dd, *J* = 9.8, 3.4 Hz, 1H, CHα-Tyr), 4.53 (d, *J* = 5.2 Hz, 1H, OC*H*H-Ox), 4.42–4.34 (m, 2H, OC*H*H-Ox, CHα-Leu), 4.27 (d, *J* = 6.6 Hz, 1H, OCH*H*-Ox), 4.23–4.14 (m, 2H, OCH*H*-Ox, CHα-Ala), 3.46–3.38 (m, 1H, CHα-Ala), 3.25 (d, *J* = 13.2 Hz, 1H, C*H*HGOx), 3.15 (dd, *J* = 13.4, 3.7 Hz, 1H, C*H*Hβ-Tyr), 3.04 (d, *J* = 13.2 Hz, 1H, CH*H*GOx), 2.86 (dd, *J* = 13.4, 11.1 Hz, 1H, CH*H*β-Tyr), 1.70–1.59 (m, 3H, CH_2β-Leu, CHγ-Leu), 1.36 (d, *J* = 7.0 Hz, 3H, CH_3β-Ala), 1.20 (d, *J* = 5.4 Hz, 3H, CH_3β-Ala), 0.95 (d, *J* = 5.7 Hz, 3H, CH_3δ-Leu), 0.90 (d, *J* = 5.4 Hz, 3H, CH_3δ-Leu); **^{13}C NMR** (101 MHz, CD_3OD) δ_C ppm 178.5 (C=O), 177.8 (C=O), 174.2 (C=O), 173.6 (C=O), 157.4 (C), 131.5 (CH), 129.2 (C), 116.3 (CH), 79.6 (OCH_2), 78.9 (OCH_2), 59.6 (C, Ox), 55.7 (CH, α-Tyr), 53.8 (CH, α-Ala), 53.7 (CH, α-Leu), 51.7 (CH, α-Ala), 44.6 (CH_2, GOx), 41.5 (CH_2, β-Leu), 38.0 (CH_2, β-Tyr), 25.9 (CH, γ-Leu), 23.6 (CH_3, δ-Leu), 21.9 (CH_3, δ-

Leu), 20.6 (CH_3, β-Ala), 19.1 (CH_3, β-Ala); **ν_{max}** (neat) = 3282, 2957, 1639, 1550, 1511, 1452, 1384, 1233, 1158, 973, 735, 696 cm^{-1}; **MS** (ESI^+) *m/z* 522 $[M+H]^+$, 544 $[M+Na]^+$; **HRMS** (ESI^+) calcd. for $C_{25}H_{40}N_5O_7$ $[M+H]^+$ 522.2922, found 522.2924; $[\alpha]_D^{25}$ –31.2 (*c* 0.30, MeOH).

Cyclo(Ala-GOx-Ala-Tyr-Leu) (13)

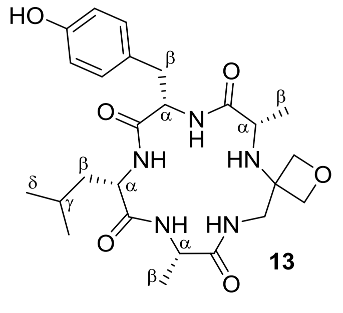

13

Representative example for cyclisation using DEPBT

To a solution of H-Ala-GOx-Ala-Tyr-Leu-OH (**68**) (52 mg, 0.10 mmol, 1.0 equiv) in anhydrous DMF (100 mL, 0.001 M) under an atmosphere of nitrogen was added DEPBT (60 mg, 0.20 mmol, 2.0 equiv) and DIPEA (35 μL, 0.20 mmol, 2.0 equiv) and the reaction mixture was stirred for 24 h at room temperature. The solvent was removed under reduced pressure, and the residue was purified twice by column chromatography (SiO_2, CH_2Cl_2/MeOH 9:1→4:1) to give the cyclic pentapeptide (**13**) as a white solid (27 mg, 54 μmol, 54%). **R_f** (CH_2Cl_2/MeOH 4:1) 0.57; **mp** 213–217 °C; **^{1}H NMR** (400 MHz, CD_3OD) δ_H ppm 7.06 (d, *J* = 8.3 Hz, 2H, ArH), 6.72 (d, *J* = 8.3 Hz, 2H, ArH), 4.53 (d, *J* = 7.8 Hz, 1H, OC*H*H-Ox), 4.49 (q, *J* = 7.1 Hz, 1H, CHα-Ala), 4.46 (d, *J* = 6.2 Hz, 1H, OC*H*H-Ox), 4.32–4.25 (m, 3H, 2 × OCH*H*-Ox, CHα-Leu), 4.19 (dd, *J* = 9.9, 6.1 Hz, 1H, CHα-Tyr), 3.70 (d, *J* = 14.0 Hz, 1H, C*H*HGOx), 3.49 (q, *J* = 6.9 Hz, 1H, CHα-Ala), 3.43 (d, *J* = 14.0 Hz, 1H, CH*H*GOx), 3.20 (dd, *J* = 13.4, 10.4 Hz, 1H, C*H*Hβ-Tyr), 3.07 (dd, *J* = 13.4, 6.1 Hz, 1H, CH*H*β-Tyr), 1.79 (ddd, *J* = 13.9, 11.2, 4.7 Hz, 1H, C*H*Hβ-Leu), 1.70–1.62 (m, 1H, CH*H*β-Leu), 1.57–1.48 (m, 1H, CHγ-Leu), 1.38 (d, *J* = 7.1 Hz, 3H, CH_3β-Ala), 1.20 (d, *J* = 6.9 Hz, 3H, CH_3β-Ala), 0.94 (d, *J* = 6.6 Hz, 3H, CH_3δ-Leu), 0.86 (d, *J* = 6.5 Hz, 3H, CH_3δ-Leu); **^{13}C NMR** (101 MHz, CD_3OD) δ_C ppm 178.7 (C=O), 175.6 (C=O), 174.3 (C=O), 173.9 (C=O), 157.4 (C), 131.3 (CH), 129.1 (C), 116.3 (CH), 81.6 (OCH_2), 79.0 (OCH_2), 62.0 (C, Ox), 57.9 (CH, α-Tyr), 55.2 (CH, α-Leu), 53.7 (CH, α-Ala), 50.7 (CH, α-Ala), 46.2 (CH_2, GOx), 40.7 (CH_2, β-Leu), 35.7 (CH_2, β-Tyr), 25.9 (CH, γ-Leu), 23.5 (CH_3, δ-Leu), 21.5 (CH_3, β-Ala), 21.2 (CH_3, δ-Leu), 17.9 (CH_3, β-Ala); **ν_{max}** (neat) = 3260, 2958, 1647, 1513, 1232, 965, 828 cm^{-1}; **MS** (ESI^+) *m/z* 504 $[M+H]^+$, 526 $[M+Na]^+$; **HRMS** (ESI^+) calcd. for $C_{25}H_{38}N_5O_6$ $[M+H]^+$ 504.2817, found 504.2818; $[\alpha]_D^{28}$ –69.7 (*c* 0.06, MeOH).

*2.7 Preparation of cyclic pentapeptide **25***

*Preparation of pentapeptide **83***

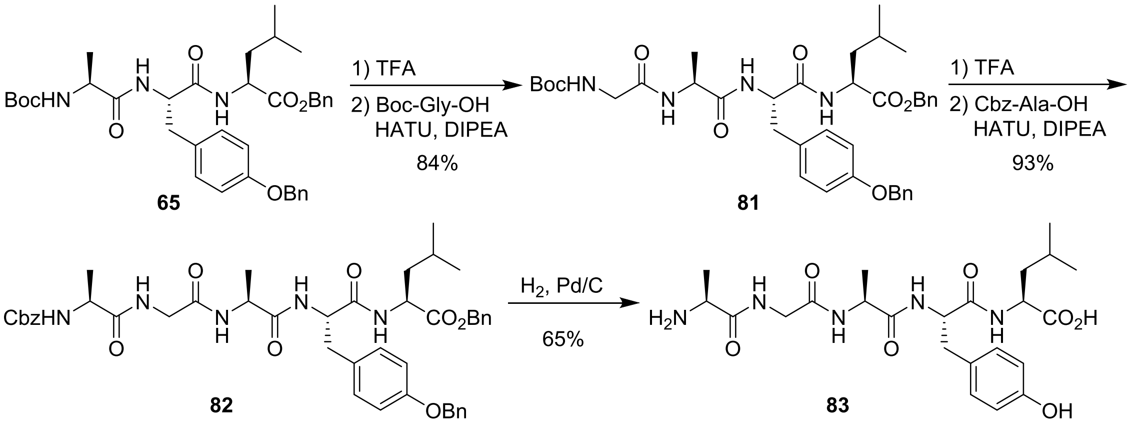

Boc-Gly-Ala-Tyr(Bn)-Leu-OBn (81)

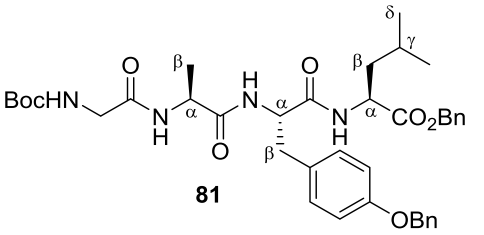

81

To a solution of Boc-Ala-Tyr(Bn)-Leu-OBn (**65**) 1.00 g, 1.55 mmol, 1.0 equiv) in CH_2Cl_2 (10 mL) was added TFA (2.0 mL) and the mixture was stirred at room temperature for 1 h. The mixture was concentrated under reduced pressure and the resulting residue repeatedly dissolved in CH_2Cl_2 (3 × 10 mL) and concentrated under reduced pressure to give the crude amine. The residue was dissolved in CH_2Cl_2 (16 mL), Boc-Gly-OH (326 mg, 1.86 mmol, 1.2 equiv), HATU (707 mg, 1.86 mmol, 1.2 equiv) and DIPEA (1.08 mL, 6.20 mmol, 4.0 equiv) were added subsequently, and the mixture was stirred at room temperature for 16 h. The reaction mixture was diluted with CH_2Cl_2 (20 mL) and washed with 10% citric acid solution (2 × 20 mL) and saturated $NaHCO_3$ solution (2 × 20 mL), dried over $MgSO_4$, filtered and concentrated under reduced pressure. The residue was purified by column chromatography (SiO_2, PE/EtOAc 1:1→EtOAc) to give tetrapeptide **81** (912 mg, 1.30 mmol, 84%) as a white solid. $\mathbf{R_f}$ (EtOAc) 0.56; **mp** 77–79 °C; ^{1}**H NMR** (400 MHz, $CDCl_3$) δ_H ppm 7.42–7.28 (m, 10H, ArH), 7.06 (d, *J* = 8.6 Hz, 2H, ArH), 7.02–6.68 (m, 5H, ArH, 3 × NH), 5.32 (br. s, 1H, NH), 5.14 (s, 2H, CH_2Ph), 4.99 (d, *J* = 4.2 Hz, 2H, CH_2Ph), 4.71 (dt, *J* = 16.3, 8.2 Hz, 1H, CHα-Tyr), 4.59 (dt, *J* = 7.9, 5.3 Hz, 1H, CHα-Leu), 4.48–4.34 (m, 1H, CHα-Ala), 3.77 (dd, *J* = 7.5, 4.5 Hz, 2H, CH_2Gly), 3.07 (dd, *J* = 14.1, 5.3 Hz, 1H, C*H*Hβ-Tyr), 2.94 (dd, *J* = 14.1, 7.4 Hz, 1H, CH*H*β-Tyr), 1.65–1.50 (m, 3H, CH_2β-Leu, CHγ-Leu), 1.43 (s, 9H, 3 × CH_3, Boc), 1.29 (d, *J* = 7.0 Hz, 3H, CH_3β-Ala), 0.87 (d, *J* = 5.9 Hz, 3H, CH_3δ-Leu), 0.86 (d, *J* = 5.8 Hz, 3H, CH_3δ-Leu); ^{13}**C NMR** (101 MHz, $CDCl_3$) δ_C ppm 172.6 (C=O), 172.2 (C=O), 170.8 (C=O), 169.7 (C=O), 157.9 (C), 156.3 (C=O, Boc), 137.1 (C), 135.5 (C), 130.5 (CH), 128.8 (C), 128.72 (CH), 128.71 (CH), 128.5 (CH), 128.3 (CH), 128.1 (CH), 127.6 (CH), 115.0 (CH), 80.7 (C, Boc), 70.1 (CH_2, Bn), 67.2 (CH_2, Bn), 54.5 (CH, α-Tyr), 51.2 (CH, α-Leu), 49.3 (CH, α-Ala), 44.4 (CH_2, Gly), 41.2 (CH_2, β-Leu), 37.3 (CH_2, β-Tyr), 28.5 (CH_3, Boc), 24.9 (CH, γ-Tyr), 22.9 (CH_3, δ-Leu), 22.0 (CH_3, δ-Leu), 18.4 (CH_3, β-Ala); $\boldsymbol{\nu_{max}}$ (neat) = 3280, 2962, 1636, 1509, 1452, 1366, 1240, 1149, 1025, 845, 734, 695 cm^{-1}; **MS** (ESI^+) *m/z* 725 $[M+Na]^+$; **HRMS** (ESI^+) calcd. for $C_{39}H_{50}N_4NaO_8$ $[M+Na]^+$ 725.3521, found 725.3523; $[\boldsymbol{\alpha}]_D^{27}$ –21.7 (*c* 0.27, $CHCl_3$).

Cbz-Ala-Gly-Ala-Tyr(Bn)-Leu-OBn (82)

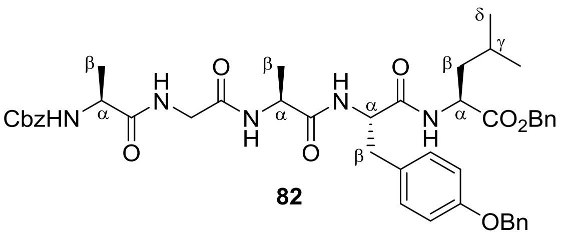

82

To a solution of Boc-Gly-Ala-Tyr(Bn)-Leu-OBn (**81**) (861 mg, 1.23 mmol, 1.0 equiv) in CH_2Cl_2 (10 mL) was added TFA (1.5 mL) and the mixture was stirred at room temperature for 1 h. The reaction mixture was concentrated under reduced pressure and the resulting residue repeatedly dissolved in CH_2Cl_2 (3 × 10 mL) and concentrated under reduced pressure to give the crude amine. The residue was dissolved in CH_2Cl_2 (13 mL), Cbz-Ala-OH (328 mg, 1.47 mmol, 1.2 equiv), HATU (559 mg, 1.47 mmol, 1.2 equiv) and DIPEA (853 μL, 4.90 mmol, 4.0 equiv) were added subsequently, and the mixture was stirred at room temperature for 16 h. The reaction mixture was diluted with CH_2Cl_2 (20 mL) and washed with 10% citric acid solution (2 × 20 mL) and saturated $NaHCO_3$ solution (2 × 20 mL), dried over $MgSO_4$, filtered and concentrated under reduced pressure. The residue was purified by column chromatography (SiO_2, CH_2Cl_2/MeOH 19:1→9:1) to give pentapeptide **82** (928 mg, 1.15 mmol, 93%) as a white solid. $\mathbf{R_f}$ (CH_2Cl_2/MeOH 9:1) 0.43; **mp** 219–221 °C; ^{1}**H NMR** (400 MHz, DMSO-*d6*) δ_H ppm 8.28 (d, *J* = 7.3 Hz, 1H, NH), 8.14 (t, *J* = 5.3 Hz, 1H, NH), 7.94 (d, *J* = 8.2 Hz, 1H, NH), 7.83 (d, *J* = 7.2 Hz, 1H, NH), 7.53 (d, *J* = 6.9 Hz, 1H, NH), 7.45–7.26 (m, 15H, ArH), 7.13 (d, *J* = 8.5 Hz, 2H, ArH), 6.87 (d, *J* = 8.5 Hz, 2H, ArH), 5.11 (s, 2H, CH_2Ph),

5.06–5.01 (m, 3H, CH_2Ph, C*H*HPh), 4.98 (d, *J* = 12.5 Hz, 1H, CH*H*Ph), 4.45 (td, *J* = 9.0, 4.4 Hz, 1H, CHα-Tyr), 4.33 (td, *J* = 8.8, 5.9 Hz, 1H, CHα-Leu), 4.24 (quint, *J* = 7.4 Hz, 1H, CHα-Ala), 4.04 (quint, *J* = 7.1 Hz, 1H, CHα-Ala), 3.68 (t, *J* = 6.3 Hz, 2H, CH_2Gly), 2.91 (dd, *J* = 13.6, 3.9 Hz, 1H, C*H*Hβ-Tyr), 2.69 (dd, *J* = 13.6, 9.8 Hz, 1H, CH*H*β-Tyr), 1.64–1.47 (m, 3H, CH_2β-Leu, CHγ-Leu), 1.21 (d, *J* = 7.1 Hz, 3H, CH_3β-Ala), 1.11 (d, *J* = 7.0 Hz, 3H, CH_3β-Ala), 0.88 (d, *J* = 6.1 Hz, 3H, CH_3δ-Leu), 0.82 (d, *J* = 6.1 Hz, 3H, CH_3δ-Leu); **^{13}C NMR** (101 MHz, $CDCl_3$) δ_C ppm 172.9 (C=O), 172.1 (C=O), 171.8 (C=O), 171.2 (C=O), 168.4 (C=O), 156.9 (C), 155.8 (C=O, Cbz), 137.2 (C), 136.9 (C), 135.9 (C), 130.2 (CH), 129.8 (C), 128.41 (CH), 128.39 (CH), 128.3 (CH), 128.0 (CH), 127.80 (CH), 127.76 (CH), 127.75 (CH), 127.6 (CH), 114.3 (CH), 69.1 (CH_2, Bn), 65.9 (CH_2, Bn), 65.5 (CH_2, Bn), 53.8 (CH, α-Tyr), 50.4 (CH, α-Leu), 50.2 (CH, α-Ala), 48.0 (CH, α-Ala), 42.1 (CH_2, Gly), 39.6 (CH_2, β-Leu), 36.4 (CH_2, β-Tyr), 24.1 (CH, γ-Tyr), 22.7 (CH_3, δ-Leu), 21.3 (CH_3, δ-Leu), 18.1 (CH_3, β-Ala), 17.9 (CH_3, β-Ala); **ν_{max}** (neat) = 3263, 1708, 1645, 1541, 1236, 1176, 1079, 745, 695 cm^{-1}; **MS** (ESI^+) *m/z* 830 $[M+Na]^+$; **HRMS** (ESI^+) calcd. for $C_{45}H_{53}N_5NaO_9$ $[M+Na]^+$ 830.3735, found 830.3742; $[\alpha]_D^{26}$ –7.3 (*c* 0.20, DMF).

H-Ala-Gly-Ala-Tyr-Leu-OH (83)

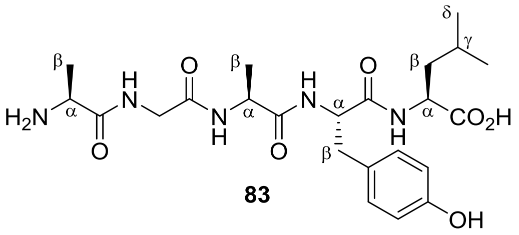

83

To a solution of pentapeptide **82** (820 mg, 1.01 mmol) in anhydrous DMF (20 mL) was added 10 wt% Pd/C (82 mg, 10 wt%) and the reaction flask was evacuated, filled with nitrogen, evacuated, and placed under an atmosphere of hydrogen (balloon). The reaction mixture was stirred at room temperature for 24 h, placed under nitrogen and filtered through a plug of Celite, which was washed with MeOH (3×). The filtrate was concentrated *in vacuo* to give **83** as a tan-coloured solid (418 mg, 65%), which required no further purification. *N.B.* The product was isolated as a complex with 2.0 equiv of DMF. **mp** 215–217 °C (decomposition); **^{1}H NMR** (400 MHz, D_2O) δ_H ppm 7.15 (d, *J* = 8.2 Hz, 2H, ArH), 6.84 (d, *J* = 8.2 Hz, 2H, ArH), 4.59 (dd, *J* = 8.7, 5.6 Hz, 1H, CHα-Tyr), 4.26 (q, *J* = 7.1 Hz, 1H, CHα-Ala), 4.20–4.15 (m, 1H, CHα-Leu), 4.12 (q, *J* = 7.2 Hz, 1H, CHα-Ala), 3.94 (s, 2H, CH_2Gly), 3.12 (dd, *J* = 14.1, 5.3 Hz, 1H, C*H*Hβ-Tyr), 2.92 (dd, *J* = 11.2, 6.0 Hz, 1H, CH*H*β-Tyr), 1.60–1.47 (m, 6H, CH_2β-Leu, CHγ-Leu, CH_3β-Ala), 1.25 (d, *J* = 7.1 Hz, 3H, CH_3β-Ala), 0.89 (d, *J* = 5.9 Hz, 3H, CH_3δ-Leu), 0.86 (d, *J* = 5.8 Hz, 3H, CH_3δ-Leu); **^{13}C NMR** (101 MHz, D_2O) δ_C ppm 179.4 (C=O), 174.3 (C=O), 171.9 (C=O), 171.7 (C=O), 170.5 (C=O), 154.3 (C), 130.6 (CH), 128.4 (C), 115.4 (CH), 54.7 (CH, α-Tyr), 54.0 (CH, α-Leu), 49.6 (CH, α-Ala), 49.1 (CH, α-Ala), 42.3 (CH_2, Gly), 40.8 (CH_2, β-Leu), 35.9 (CH_2, β-Tyr), 31.3 (CH, γ-Tyr), 24.5 (CH_3, δ-Leu), 22.4 (CH_3, δ-Leu), 20.9 (CH_3, β-Ala), 16.5 (CH_3, β-Ala); **ν_{max}** (neat) = 3275, 1628, 1512, 1386, 1236, 1086, 845 cm^{-1}; **MS** (ESI^+) *m/z* 494 $[M+H]^+$, 516 $[M+Na]^+$; **HRMS** (ESI^+) calcd. for $C_{23}H_{36}N_5O_7$ $[M+H]^+$ 494.2609, found 494.2608; $[\alpha]_D^{27}$ –14.2 (*c* 0.30, MeOH).

*Preparation of pentapeptide **87***

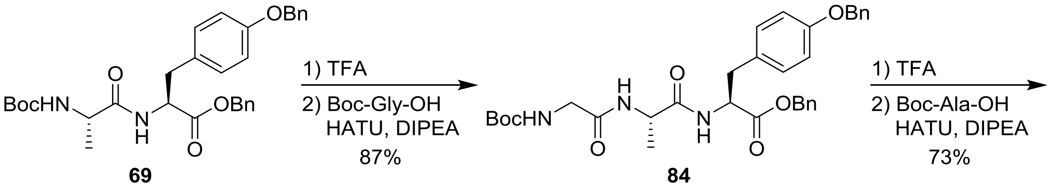

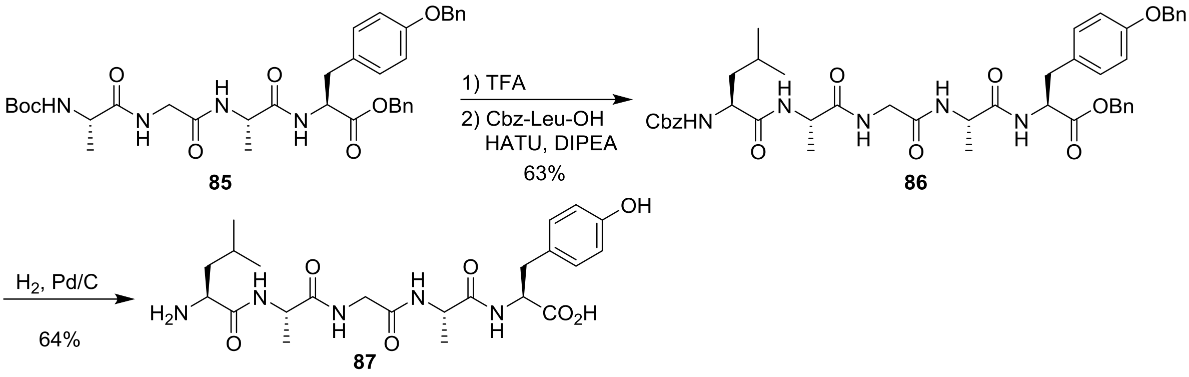

Boc-Gly-Ala-Tyr(Bn)-OBn (84)

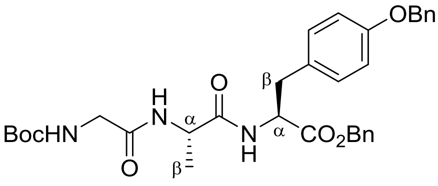

84

To a solution of Boc-Ala-Tyr(Bn)-OBn (**69**) (2.66 g, 5.00 mmol, 1.0 equiv) in CH_2Cl_2 (5.0 mL) was added TFA (5.0 mL) and the mixture was stirred at room temperature for 30 min (*Caution – gas evolution!*). The reaction mixture was concentrated under reduced pressure and the resulting residue repeatedly dissolved in CH_2Cl_2 (3 × 10 mL) and concentrated under reduced pressure to give the crude amine. The residue was dissolved in a mixture of CH_2Cl_2 (50 mL) and DMF (10 mL), Boc-Gly-OH (1.05 g, 6.00 mmol, 1.2 equiv), HATU (2.28 g, 6.00 mmol, 1.2 equiv) and DIPEA (3.48 mL, 20.0 mmol, 4.0 equiv) were added subsequently, and the mixture was stirred at room temperature for 16 h. The reaction mixture was washed with 10% citric acid solution (2 × 50 mL) and saturated $NaHCO_3$ solution (2 × 50 mL), dried over $MgSO_4$, filtered and concentrated under reduced pressure. The residue was purified by column chromatography (SiO_2, PE/EtOAc 1:1→EtOAc) to give tripeptide Boc-Gly-Ala-Tyr(Bn)-OBn (**84**) (2.57 g, 4.36 mmol, 87%) as a white foam. $\mathbf{R_f}$ (EtOAc) 0.56; **mp** 52–55 °C; **^{1}H NMR** (400 MHz, $CDCl_3$) δ_H ppm 7.44–7.28 (m, 10H, ArH), 6.91 (d, *J* = 8.4 Hz, 2H, ArH), 6.82 (d, *J* = 8.4 Hz, 2H, ArH), 6.59 (d, *J* = 7.3 Hz, 1H, NH), 6.51 (d, *J* = 7.1 Hz, 1H, NH), 5.18 (d, *J* = 12.1 Hz, 1H, C*H*HPh), 5.09 (d, *J* = 12.1 Hz, 2H, CH*H*Ph), 5.01 (s, 2H, CH_2Ph), 4.83 (dd, *J* = 13.7, 6.1 Hz, 1H, CHα-Tyr), 4.44 (quint, *J* = 7.0 Hz, 1H, CHα-Ala), 3.78 (dd, *J* = 16.8, 5.7 Hz, 1H, C*H*HGly), 3.70 (dd, *J* = 16.8, 5.7 Hz, 1H, CH*H*Gly), 3.07 (dd, *J* = 14.0, 6.0 Hz, 1H, C*H*Hβ-Tyr), 3.01 (dd, *J* = 14.0, 6.2 Hz, 1H, CH*H*β-Tyr), 1.66 (br. s, 1H, NH), 1.45 (s, 9H, 3 × CH_3, Boc), 1.32 (d, *J* = 7.0 Hz, 3H, CH_3β-Ala); **^{13}C NMR** (101 MHz, $CDCl_3$) δ_C ppm 171.7 (C=O), 171.3 (C=O), 169.5 (C=O), 158.0 (C), 156.2 (C=O, Boc), 137.1 (C), 135.2 (C), 130.5 (CH), 128.74 (CH), 128.71 (CH), 128.69 (CH), 128.1 (CH), 127.9 (C), 127.6 (CH), 115.0 (CH), 80.5 (C, Boc), 70.1 (CH_2, Bn), 67.4 (CH_2, Bn), 53.5 (CH, α-Tyr), 48.8 (CH, α-Ala), 44.3 (CH_2, Gly), 37.0 (CH_2, β-Tyr), 28.4 (CH_3, Boc), 18.2 (CH_3, β-Ala). *N.B.* One aromatic CH signal not observed; $\mathbf{\nu_{max}}$ (neat) = 3299, 1648, 1509, 1453, 1367, 1239, 1163, 1025, 841, 735, 696 cm^{-1}; **MS** (ESI$^+$) *m/z* 590 $[M+H]^+$, 612 $[M+Na]^+$, 628 $[M+K]^+$; **HRMS** (ESI$^+$) calcd. for $C_{33}H_{39}N_3NaO_7$ $[M+Na]^+$ 612.2680, found 612.2681; $[\boldsymbol{\alpha}]_D^{27}$ –7.7 (*c* 0.88, $CHCl_3$).

Boc-Ala-Gly-Ala-Tyr(Bn)-OBn (85)

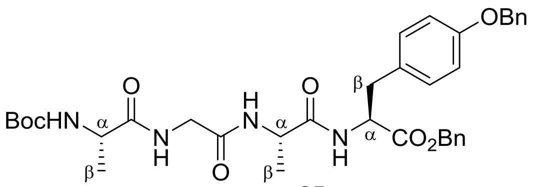

85

To a solution of Boc-Gly-Ala-Tyr(Bn)-OBn (**84**) (887 mg, 1.50 mmol, 1.0 equiv) in CH_2Cl_2 (2.0 mL) was added TFA (2.0 mL) and the mixture was stirred at room temperature for 30 min (*Caution – gas evolution!*). The reaction mixture was concentrated under reduced pressure and the resulting residue repeatedly dissolved in CH_2Cl_2 (3 × 10 mL) and concentrated under reduced pressure to give the crude amine. The residue

was dissolved in a mixture of CH_2Cl_2 (15 mL) and DMF (5.0 mL), Boc-Ala-OH (341 mg, 1.80 mmol, 1.2 equiv), HATU (684 mg, 1.80 mmol, 1.2 equiv) and DIPEA (1.05 mL, 6.00 mmol, 4.0 equiv) were added subsequently, and the mixture was stirred at room temperature for 16 h. The reaction mixture was washed with 10% citric acid solution (2 × 50 mL) and saturated $NaHCO_3$ solution (2 × 50 mL), dried over $MgSO_4$, filtered and concentrated under reduced pressure. The residue was purified by column chromatography (SiO_2, EtOAc) to give tetrapeptide Boc-Ala-Gly-Ala-Tyr(Bn)-OBn (**85**) (725 mg, 1.10 mmol, 73%) as a white solid. **R_f** (EtOAc) 0.40; **mp** 137–143 °C; **^{1}H NMR** (400 MHz, DMSO-*d6*) δ_H 8.32 (d, *J* = 5.1 Hz, 1H, NH), 8.02 (t, *J* = 5.1 Hz, 1H, NH), 7.86 (d, *J* = 7.4 Hz, 1H, NH), 7.46–7.29 (m, 8H, ArH), 7.28–7.23 (m, 2H, ArH), 7.12 (d, *J* = 8.4 Hz, 2H, ArH), 7.02 (d, *J* = 6.8 Hz, 1H, NH), 6.89 (d, *J* = 8.4 Hz, 2H, ArH), 5.05 (s, 4H, 2 × CH_2Ph), 4.45 (dd, *J* = 14.6, 7.5 Hz, 1H, CHα-Tyr), 4.32 (quint, *J* = 6.8 Hz, 1H, CHα-Ala), 3.96 (quint, *J* = 6.9 Hz, 1H, CHα-Ala), 3.72 (dd, *J* = 16.8, 5.6 Hz, 1H, C*H*HGly), 3.66 (dd, *J* = 16.8, 5.6 Hz, 1H, CH*H*Gly), 2.98 (dd, *J* = 13.9, 6.3 Hz, 1H, C*H*Hβ-Tyr), 2.90 (dd, *J* = 13.9, 8.3 Hz, 1H, CH*H*β-Tyr), 1.37 (s, 9H, 3 × CH_3, Boc), 1.18 (d, *J* = 7.1 Hz, 3H, CH_3β-Ala), 1.14 (d, *J* = 7.0 Hz, 3H, CH_3β-Ala); **^{13}C NMR** (101 MHz, DMSO-*d6*) δ_C ppm 173.2 (C=O), 172.2 (C=O), 171.2 (C=O), 168.2 (C=O), 157.2 (C), 155.2 (C=O, Boc), 137.2 (C), 135.7 (C), 130.2 (CH), 129.1 (C), 128.4 (CH), 128.3 (CH), 128.0 (CH), 127.9 (CH), 127.8 (CH), 127.6 (CH), 114.6 (CH), 78.2 (C, Boc), 69.1 (CH_2, Bn), 66.0 (CH_2, Bn), 54.0 (CH, α-Tyr), 49.9 (CH, α-Ala), 47.7 (CH, α-Ala), 42.0 (CH_2, Gly), 35.8 (CH_2, β-Tyr), 28.2 (CH_3, Boc), 18.3 (CH_3, β-Ala), 18.0 (CH_3, β-Ala); **ν_{max}** (neat) = 3280, 1638, 1509, 1449, 1387, 1242, 1166, 842, 732, 695 cm^{-1}; **MS** (ESI$^+$) *m/z* 683 $[M+Na]^+$, 699 $[M+K]^+$; **HRMS** (ESI$^+$) calcd. for $C_{36}H_{44}N_4NaO_8$ $[M+Na]^+$ 683.3051, found 683.3054; $[\alpha]_D^{27}$ +5.4 (*c* 0.72, $CHCl_3$).

Cbz-Leu-Ala-Gly-Ala-Tyr(Bn)-OBn (86)

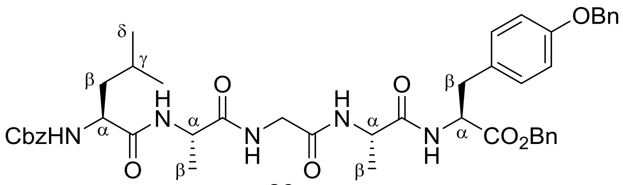

To a solution of Boc-Ala-Gly-Ala-Tyr(Bn)-OBn (**85**) (642 mg, 0.97 mmol, 1.0 equiv) in CH_2Cl_2 (2.0 mL) was added TFA (1.0 mL) and the mixture was stirred at room temperature for 30 min (*Caution – gas evolution!*). The reaction mixture was concentrated under reduced pressure and the resulting residue repeatedly dissolved in CH_2Cl_2 (3 × 10 mL) and concentrated under reduced pressure to give the crude amine. The residue was dissolved in a mixture of CH_2Cl_2 (5.0 mL) and DMF (5.0 mL), Cbz-Leu-OH (309 mg, 1.16 mmol, 1.2 equiv), HATU (441 mg, 1.16 mmol, 1.2 equiv) and DIPEA (696 µL, 3.88 mmol, 4.0 equiv) were added subsequently, and the mixture was stirred at room temperature for 16 h. The reaction mixture was washed with 10% citric acid solution (2 × 50 mL) and saturated $NaHCO_3$ solution (2 × 50 mL), dried over $MgSO_4$, filtered and concentrated under reduced pressure. The residue was purified by column chromatography (SiO_2, CH_2Cl_2/MeOH 98:2→96:4) to give pentapeptide Cbz-Leu-Ala-Gly-Ala-Tyr(Bn)-OBn (**86**) (496 mg, 0.61 mmol, 63%) as a white solid. **R_f** (CH_2Cl_2/MeOH 9:1) 0.37; **mp** 173–175 °C; **^{1}H NMR** (400 MHz, DMSO-*d6*) δ_H ppm 8.35 (d, *J* = 7.3 Hz, 1H, NH), 8.09 (t, *J* = 5.2 Hz, 1H, NH), 8.04 (d, *J* = 6.7 Hz, 1H, NH), 7.89 (d, *J* = 7.6 Hz, 1H, NH), 7.45–7.23 (m, 16H, ArH, NH), 7.11 (d, *J* = 8.3 Hz, 2H, ArH), 6.89 (d, *J* = 8.3 Hz, 2H, ArH), 5.05 (s, 4H, 2 × CH_2Ph), 5.01 (s, 2H, CH_2Ph), 4.45 (q, *J* = 7.2 Hz, 1H, CHα-Tyr), 4.33 (quint, *J* = 7.1 Hz, 1H, CHα-Ala), 4.26 (quint, *J* = 6.9 Hz, 1H, CHα-Ala), 4.06 (q, *J* = 7.2 Hz, 1H, CHα-Leu), 3.73 (dd, *J* = 16.8, 5.4 Hz, 1H, C*H*HGly), 3.66 (dd, *J* = 16.8, 5.4 Hz, 1H, CH*H*Gly), 2.97 (dd, *J* = 13.6, 6.4 Hz, 1H, C*H*Hβ-Tyr), 2.90 (dd, *J* = 13.6, 8.3 Hz, 1H, CH*H*β-Tyr), 1.62 (nonet, *J* = 6.4 Hz, 1H, CHγ-Leu), 1.43 (t, *J* = 6.9 Hz, 2H, CH_2β-Leu), 1.22 (d, *J* = 6.9 Hz, 3H, CH_3β-Ala), 1.15 (d, *J* = 6.8 Hz, 3H, CH_3β-Ala), 0.86 (d, *J* = 6.3 Hz, 3H, CH_3δ-Leu), 0.84 (d, *J* = 6.2 Hz, 3H, CH_3δ-Leu); **^{13}C NMR** (101 MHz, DMSO-*d6*) δ_C ppm δ 172.5 (C=O), 172.24 (C=O), 172.18 (C=O), 171.2 (C=O), 168.1 (C=O),

157.3 (C), 156.0 (C=O, Cbz), 137.2 (C), 137.1 (C), 135.7 (C), 130.2 (CH), 129.0 (C), 128.4 (CH), 128.3 (CH), 128.0 (CH), 127.9 (CH), 127.79 (CH), 127.76 (CH), 127.7 (CH), 127.6 (CH), 114.6 (CH), 69.2 (CH_2, Bn), 66.0 (CH_2, Bn), 65.4 (CH_2, Bn), 54.0 (CH, α-Tyr), 53.0 (CH, α-Leu), 48.3 (CH, α-Ala), 47.7 (CH, α-Ala), 41.9 (CH_2, Gly), 40.6 (CH_2, β-Leu), 35.8 (CH_2, β-Tyr), 24.2 (CH, γ-Leu), 23.1 (CH_3, δ-Leu), 21.4 (CH_3, δ-Leu), 18.3 (CH_3, β-Ala), 18.1 (CH_3, β-Ala). *N.B.* One aromatic CH signal not observed; $\mathbf{\nu_{max}}$ (neat) = 3286, 1631, 1510, 1453, 1172, 1026, 840, 734, 695 cm^{-1}; **MS** (ESI^+) *m/z* 830 $[M+Na]^+$, 846 $[M+K]^+$; **HRMS** (ESI^+) calcd. for $C_{45}H_{53}N_5NaO_9$ $[M+Na]^+$ 830.3735, found 830.3741; $[\boldsymbol{\alpha}]_D^{25}$ –23.3 (*c* 0.20, MeOH).

H-Leu-Ala-Gly-Ala-Tyr-OH (87)

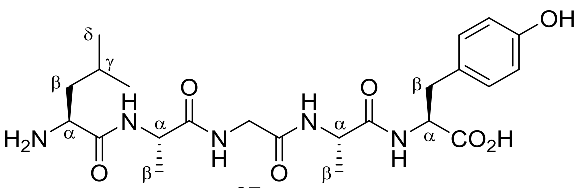

87

To a solution of pentapeptide **86** (402 mg, 0.50 mmol) in a mixture of anhydrous MeOH (15 mL) and DMF (5.0 mL) was added 10 wt% Pd/C (40 mg, 10 wt%) and the reaction flask was evacuated, filled with nitrogen, evacuated, and placed under an atmosphere of hydrogen (balloon). The reaction mixture was stirred at room temperature for 16 h, placed under nitrogen and filtered through a plug of Celite, which was washed with MeOH (3×). The filtrate was concentrated *in vacuo* to give **87** as an off-white solid (199 mg) in 64% yield. *N.B.* The product was obtained as a complex with 2.0 equiv of DMF. **mp** 109–113 °C; **^{1}H NMR** (400 MHz, DMSO-*d6*) δ_H ppm 8.60 (br. s, 1H, OH), 8.22 (t, *J* = 4.9 Hz, 1H, NH), 8.04 (d, *J* = 7.2 Hz, 1H, NH), 7.68 (d, *J* = 7.2 Hz, 1H, NH), 6.95 (d, *J* = 8.2 Hz, 2H, ArH), 6.60 (d, *J* = 8.2 Hz, 2H, ArH), 4.33–4.24 (m, 2H, CHα-Ala, NH), 4.21 (quint, *J* = 7.3 Hz, 1H, CHα-Ala), 4.13 (dd, *J* = 12.6, 6.8 Hz, 1H, CHα-Tyr), 3.69 (d, *J* = 5.2 Hz, 2H, CH_2Gly), 3.50 (dd, *J* = 7.5, 6.6 Hz, 1H, CHα-Leu), 2.93 (dd, *J* = 13.6, 5.0 Hz, 1H, C*H*Hβ-Tyr), 2.79 (dd, *J* = 13.6, 7.0 Hz, 1H, CH*H*β-Tyr), 1.68 (nonet, *J* = 6.7 Hz, 1H, CHγ-Leu), 1.56–1.47 (m, 1H, C*H*Hβ-Leu), 1.43–1.34 (m, 1H, CH*H*β-Leu), 1.23 (d, *J* = 7.0 Hz, 3H, CH_3β-Ala), 1.17 (d, *J* = 7.0 Hz, 3H, CH_3β-Ala), 0.88 (d, *J* = 6.5 Hz, 3H, CH_3δ-Leu), 0.85 (d, *J* = 6.5 Hz, 3H, CH_3δ-Leu). *N.B.* Three protic NH/OH signals not observed; **^{13}C NMR** (101 MHz, DMSO-*d6*) δ_C ppm 172.2 (C=O), 171.84 (C=O), 171.75 (C=O), 171.3 (C=O), 168.3 (C=O), 155.7 (C), 130.3 (CH), 128.2 (C), 114.8 (CH), 54.8 (CH, α-Tyr), 52.0 (CH, α-Leu), 48.5 (CH, α-Ala), 48.4 (CH, α-Ala), 42.1 (CH_2, Gly), 41.7 (CH_2, β-Leu), 36.2 (CH_2, β-Tyr), 23.8 (CH, γ-Leu), 22.9 (CH_3, δ-Leu), 21.9 (CH_3, δ-Leu), 18.12 (CH_3, β-Ala), 18.07 (CH_3, β-Ala); $\mathbf{\nu_{max}}$ (neat) = 3279, 1631, 1511, 1450, 1385, 1237, 1172, 833, 733, 695 cm^{-1}; **MS** (ESI^+) *m/z* 494 $[M+H]^+$, 516 $[M+Na]^+$; **HRMS** (ESI^+) calcd. for $C_{23}H_{36}N_5O_7$ $[M+H]^+$ 494.2609, found 494.2609; $[\boldsymbol{\alpha}]_D^{26}$ +8.7 (*c* 0.30, MeOH).

Cyclo(Ala-Gly-Ala-Tyr-Leu) (25)

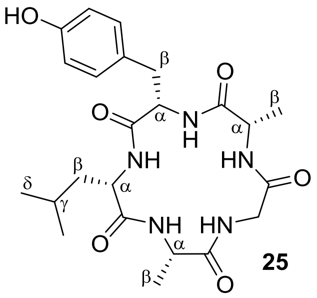

25

Representative example for cyclisation using DEPBT

To a solution of H-Ala-Gly-Ala-Tyr-Leu-OH (**83**) (50 mg, 0.10 mmol, 1.0 equiv) in anhydrous DMF (100 mL, 0.001 M) under an atmosphere of nitrogen was added DEPBT (60 mg, 0.20 mmol, 2.0 equiv) and DIPEA (35 μL, 0.20 mmol, 2.0 equiv) and the mixture was stirred for 24 h at room temperature. The solvent was removed *in vacuo* and the residue was purified twice by column chromatography (SiO_2, CH_2Cl_2/MeOH 9:1→4:1) to give the cyclic pentapeptide **25** as a white solid (20 mg, 42 μmol, 42%). $\mathbf{R_f}$ (CH_2Cl_2/MeOH 4:1) 0.48; **mp** 294–296 °C (decomposition). Lit. 284–286 °C;[6] **^{1}H NMR** (400 MHz, CD_3OD) δ_H ppm 7.08 (d, *J* = 8.4 Hz, 2H, ArH), 6.70 (d, *J* = 8.4 Hz, 2H, ArH), 4.51

(t, J = 8.0 Hz, 1H, CHα-Tyr), 4.34 (q, J = 7.0 Hz, 1H, CHα-Ala), 4.19 (q, J = 7.3 Hz, 1H, CHα-Ala), 4.04 (dd, J = 10.5, 5.2 Hz, 1H, CHα-Leu), 3.98 (d, J = 14.7 Hz, 1H, C*H*H-Gly), 3.59 (d, J = 14.7 Hz, 1H, CH*H*-Gly), 3.06 (dd, J = 12.3, 6.1 Hz, 1H, C*H*Hβ-Tyr), 3.01 (dd, J = 12.3, 7.5 Hz, 1H, CH*H*β-Tyr), 1.86 (ddd, J = 13.5, 10.7, 4.8 Hz, 1H, C*H*Hβ-Leu), 1.57–1.48 (m, 1H, CH*H*β-Leu), 1.47–1.40 (m, 1H, CHγ-Leu), 1.37 (d, J = 7.1 Hz, 3H, CH_3β-Ala), 1.28 (d, J = 7.3 Hz, 3H, CH_3β-Ala), 0.93 (d, J = 6.5 Hz, 3H, CH_3δ-Leu), 0.86 (d, J = 6.4 Hz, 3H, CH_3δ-Leu); **^{13}C NMR** (101 MHz, CD_3OD) δ_C ppm 175.5 (C=O), 175.03 (C=O), 174.98 (C=O), 173.7 (C=O), 172.0 (C=O), 157.4 (C), 131.3 (CH), 128.8 (C), 116.2 (CH), 57.2 (CH, α-Tyr), 56.2 (CH, α-Leu), 51.8 (CH, α-Ala), 50.6 (CH, α-Ala), 44.4 (CH_2, Gly), 40.4 (CH_2, β-Leu), 37.0 (CH_2, β-Tyr), 25.9 (CH, γ-Leu), 23.4 (CH_3, δ-Leu), 21.7 (CH_3, δ-Leu), 17.7 (CH_3, β-Ala), 17.1 (CH_3, β-Ala); **ν_{max}** (neat) = 3279, 1648, 1631, 1530, 1514, 1440, 1384, 1226, 1087 cm^{-1}; **MS** (ESI$^+$) *m/z* 498 [M+Na]$^+$; **HRMS** (ESI$^+$) calcd. for $C_{23}H_{33}N_5NaO_6$ [M+Na]$^+$ 498.2323, found 498.2322; $[\alpha]_D^{26}$ –82.8 (*c* 0.20, MeOH). Lit. $[\alpha]_D^{20}$ –104 (*c* 0.10, C_2H_5OH).[6]

*Preparation of cyclic pentapeptides **13** and **25** by different bond formations, to be read in conjunction with Table 3 in paper.*

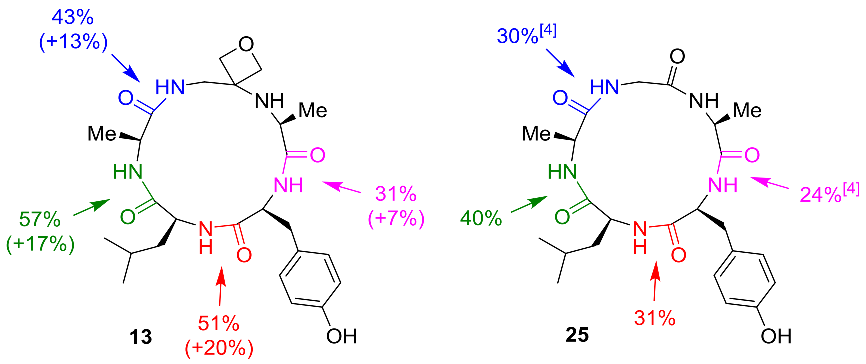

Linear pentapeptide	Cyclic peptide	1st run	2nd run	Average yield
H-GOx-Ala-Tyr-Leu-Ala-OH (**80**)	**13**	43%	43%	**43%**
H-Ala-GOx-Ala-Tyr-Leu-OH (**68**)	**13**	54%	60%	**57%**
H-Leu-Ala-GOx-Ala-Tyr-OH (**73**)	**13**	56%	46%	**51%**
H-Tyr-Leu-Ala-GOx-Ala-OH (**63**)	**13**	32%	30%	**31%**
H-Ala-Gly-Ala-Tyr-Leu-OH (**83**)	**25**	42%	38%	**40%**
H-Leu-Ala-Gly-Ala-Tyr-OH (**87**)	**25**	33%	29%	**31%**

*2.8 Preparation of cyclic pentapeptide **92***

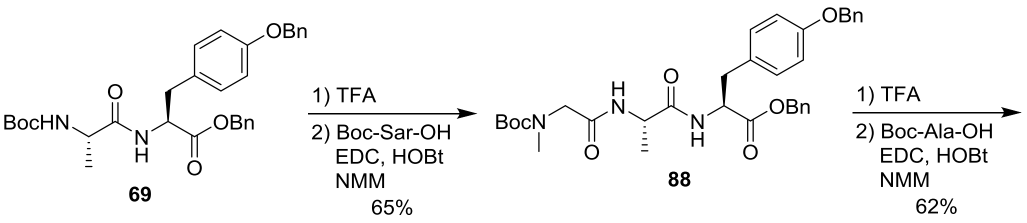

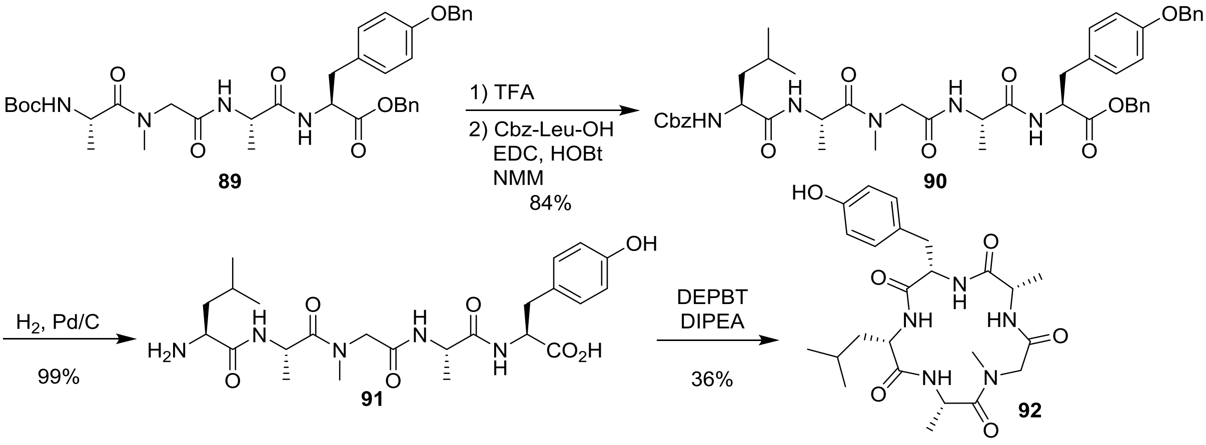

Boc-Sar-Ala-Tyr(Bn)-OBn (88)

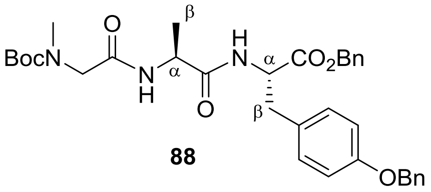

88

To a solution of Boc-Ala-Tyr(Bn)-OBn (**69**) (3.19 g, 6.00 mmol, 1.0 equiv) in CH_2Cl_2 (6.0 mL) was added TFA (6.0 mL) and the mixture was stirred at room temperature for 1 h *(Gas evolution!)*. The mixture was concentrated under reduced pressure and the resulting residue repeatedly dissolved in CH_2Cl_2 (3 × 25 mL) and concentrated *in vacuo* to give the crude amine. The residue was dissolved in CH_2Cl_2 (60 mL), Boc-Sar-OH (1.14 g, 6.00 mmol, 1.0 equiv), EDC·HCl (1.15 g, 6.00 mmol, 1.0 equiv), HOBt·H_2O (0.81 g, 6.00 mmol, 1.0 equiv) and NMM (2.64 mL, 24.0 mmol, 4.0 equiv) were added, and the mixture was stirred at room temperature for 24 h. The mixture was diluted with EtOAc (60 mL) and washed with brine (3 × 100 mL), dried over Na_2SO_4, filtered and concentrated *in vacuo*. The residue was purified by column chromatography (SiO_2, EtOAc/PE 1:1) to give Boc-Sar-Ala-Tyr(Bn)-OBn (**88**) (2.64 g, 3.91 mmol, 65%) as a white foam. **R_f** (EtOAc/PE 1:1) 0.17; **mp** 118–121 °C; **^{1}H NMR** (500 MHz, $CDCl_3$) δ_H 7.44–7.27 (m, 10H, ArH), 6.92 (d, J = 8.4 Hz, 2H, ArH), 6.82 (d, J = 8.4 Hz, 2H, ArH), 6.70–6.38 (m, 2H, 2 × NH), 5.17 (d, J = 12.1 Hz, 1H, C*H*HPh), 5.09 (d, J = 12.1 Hz, 1H, CH*H*Ph), 5.00 (s, 2H, CH_2Ph), 4.82 (dd, J = 13.2, 6.1 Hz, 1H, CHα-Tyr), 4.47 (s, 1H CHα-Ala), 3.90–3.70 (m, 2H, CH_2Sar), 3.09–3.00 (m, 2H, CH_2βTyr), 2.90 (s, 3H, NCH_3), 1.46 (s, 9H, 3 × CH_3, Boc), 1.32 (d, J = 6.9 Hz, 3H, CH_3β-Ala); **^{13}C NMR** (126 MHz, $CDCl_3$) δ_C 171.6 (C=O), 171.2 (C=O), 158.1 (C), 156.5 (C=O, Boc), 137.1 (C), 135.2 (C), 130.4 (CH), 128.73 (CH), 128.70 (CH), 128.66 (CH), 128.1 (CH), 127.8 (C), 127.6 (CH), 115.0 (CH), 80.9 (C, Boc), 70.1 (CH_2, Bn), 67.4 (CH_2, Bn), 53.5 (CH, α-Tyr), 53.0 (CH_2, Sar), 48.7 (CH, α-Ala), 37.0 (CH_2, β-Tyr), 35.9 (NCH_3), 28.4 (CH_3, Boc), 18.1 (CH_3, β-Ala). *N.B.* C=O Boc, CH_2, Sar and CH_3, β-Ala are broad and not seen clearly, assignment by HSQC and HMBC correlations; one aromatic CH not observed; **ν_{max}** (neat) = 3282, 2974, 1731, 1699, 1638, 1117 cm^{-1}; **MS** (ESI$^+$) *m/z* 626 [M+Na]$^+$; **HRMS** (ESI$^+$) calcd. for $C_{34}H_{41}N_3NaO_7$ [M+Na]$^+$ 626.2837, found 626.2835; $[\alpha]_D^{29}$ –38.0 (*c* 0.12, MeOH).

Boc-Ala-Sar-Ala-Tyr(Bn)-OBn (89)

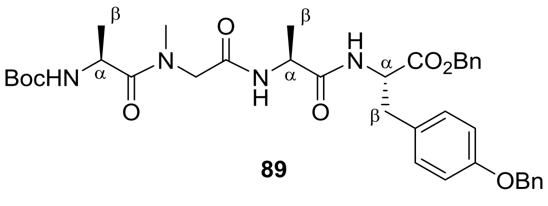

89

To a solution of Boc-Sar-Ala-Tyr(Bn)-OBn (**88**) (2.05 g, 3.40 mmol, 1.0 equiv) in CH_2Cl_2 (5.0 mL) was added TFA (5.0 mL) and the mixture was stirred at room temperature for 1 h *(Gas evolution!)*. The mixture was concentrated *in vacuo* and the residue repeatedly dissolved in CH_2Cl_2 (3 × 25 mL) and concentrated *in vacuo* to give the crude amine. The residue was dissolved in CH_2Cl_2 (34 mL), Boc-Ala-OH (0.64 g, 3.40 mmol, 1.0 equiv), EDC·HCl (0.65 g, 3.40 mmol, 1.0 equiv), HOBt·H_2O (0.46 g,

3.40 mmol, 1.0 equiv) and NMM (1.50 mL, 13.6 mmol, 4.0 equiv) were added, and the mixture was stirred at room temperature for 24 h. The mixture was diluted with EtOAc (40 mL) and washed with brine (3 × 60 mL), dried over Na_2SO_4, filtered and concentrated *in vacuo*. The residue was purified by column chromatography (SiO_2, CH_2Cl_2/MeOH 97:3) to give Boc-Ala-Sar-Ala-Tyr(Bn)-OBn (**89**) (1.42 g, 2.11 mmol, 62%) as a white foam. **R_f** (CH_2Cl_2/MeOH 97:3) 0.32; **mp** 69–72 °C; **^{1}H NMR** (500 MHz, DMSO-*d6*) δ_H 7.86 (d, *J* = 5.7 Hz, 1H, NH), 7.47–7.24 (m, 10H, ArH), 7.10 (d, *J* = 8.5 Hz, 2H, ArH), 6.90 (d, *J* = 8.5 Hz, 2H, ArH), 6.23 (s, 1H, NH), 5.11–5.04 (m, 4H, 2 × CH_2Ph), 4.56 (q, *J* = 7.2 Hz, 1H, CHα-Tyr), 4.46–4.30 (m, 2H, 2 × CHα-Ala), 4.07–3.78 (m, 2H, CH_2Sar), 3.03 (dd, *J* = 14.1, 6.3 Hz, 1H, C*H*HβTyr), 2.98–2.94 (m, 1H, CH*H*βTyr), 1.39 (s, 9H, 3 × CH_3, Boc), 1.23–1.17 (m, 6H, 2 × CH_3β-Ala). *N.B.* NCH_3 and one NH signal not observed; **^{13}C NMR** (126 MHz, DMSO-*d6*) δ_C 172.3 (C=O), 171.4 (C=O), 170.3 (C=O), 167.1 (C=O), 156.9 (C), 154.2 (C=O, Boc), 136.9 (C), 135.3 (C), 129.5 (CH), 128.8 (C), 127.7 (CH), 127.6 (CH), 127.3 (CH), 127.2 (CH), 127.0 (CH), 126.8 (CH), 114.4 (CH), 77.7 (C, Boc), 69.2 (CH_2, Bn), 65.5 (CH_2, Bn), 53.3 (CH, α-Tyr), 47.6 (CH, α-Ala), 45.7 (CH, α-Ala), 35.6 (CH_2, β-Tyr), 27.6 (CH_3, Boc), 17.3 (CH_3β-Ala), 17.1 (CH_3β-Ala). *N.B.* NCH_3 and CH_2, Sar not observed, assignment by HSQC and HMBC correlations; **ν_{max}** (neat) = 2986, 1744, 1714, 1654, 1164, 1013 cm^{-1}; **MS** (ESI^+) *m/z* 697 $[M+Na]^+$; **HRMS** (ESI^+) calcd. for $C_{37}H_{46}N_4NaO_8$ $[M+Na]^+$ 697.3208, found 697.3211; $[\alpha]_D^{29}$ –43.0 (*c* 0.11, MeOH).

Cbz-Leu-Ala-Sar-Ala-Tyr(Bn)-OBn (90)

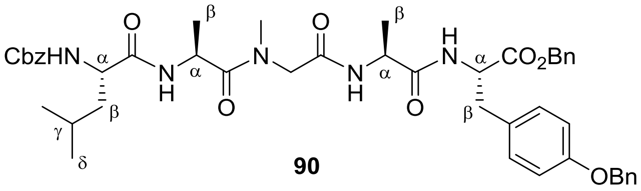

90

To a solution of **89** (1.33 g, 2.06 mmol, 1.0 equiv) in CH_2Cl_2 (3.0 mL) was added TFA (3.0 mL) and the mixture was stirred at room temperature for 1 h *(Gas evolution!)*. The mixture was concentrated under reduced pressure and the resulting residue repeatedly dissolved in CH_2Cl_2 (3 × 25 mL) and concentrated *in vacuo* to give the crude amine. The residue was dissolved in CH_2Cl_2 (21 mL), Cbz-Leu-OH (0.55 g, 2.06 mmol, 1.0 equiv), EDC·HCl (0.39 g, 2.06 mmol, 1.0 equiv), HOBt·H_2O (0.28 g, 2.06 mmol, 1.0 equiv) and NMM (0.91 mL, 8.22 mmol, 4.0 equiv) were added, and the mixture was stirred at room temperature for 24 h. The mixture was diluted with EtOAc (25 mL) and washed with brine (3 × 40 mL), dried over Na_2SO_4, filtered and concentrated *in vacuo*. The residue was purified by column chromatography (SiO_2, CH_2Cl_2/MeOH 19:1) to give pentapeptide **90** (1.43 g, 1.73 mmol, 84%) as a white foam. **R_f** (CH_2Cl_2/MeOH 19:1) 0.35; **mp** 77–80 °C; **^{1}H NMR** (500 MHz, DMSO-*d6*) δ_H 7.79 (s, 1H, NH), 7.55 (d, *J* = 6.4 Hz, 1H, NH), 7.38–7.19 (m, 16H, ArH, NH), 7.03 (d, *J* = 8.3 Hz, 2H, ArH), 6.82 (d, *J* = 8.3 Hz, 3H, ArH, Cbz NH), 5.12–4.87 (m, 6H, 3 × CH_2Ph), 4.68–4.59 (m, 1H, CHα-Ala), 4.50 (q, *J* = 7.3 Hz, 1H, CHα-Tyr), 4.29 (quint, *J* = 6.9 Hz, 1H, CHα-Ala), 4.13–3.65 (m, 3H, CHα-Leu, CH_2Sar), 2.95 (dd, *J* = 14.1, 6.3 Hz, 1H, C*H*HβTyr), 2.91–2.87 (m, 1H, CH*H*βTyr), 1.60 (m, 1H, CHγ-Leu), 1.49–1.37 (m, 2H, CH_2β-Leu), 1.14 (d, *J* = 6.9 Hz, 6H, 2 × CH_3β-Ala), 0.82 (d, *J* = 6.8 Hz, 3H, CH_3δ-Leu), 0.80 (d, *J* = 6.6 Hz, 3H, CH_3δ-Leu). *N.B.* NCH_3 signal not observed; **^{13}C NMR** (126 MHz, DMSO-*d6*) δ_C 171.8 (C=O), 171.4 (C=O), 170.9 (C=O), 170.4 (C=O), 167.0 (C=O), 156.9 (C), 155.2 (C=O, Cbz), 136.9 (C), 136.6 (C), 135.3 (C), 129.5 (CH), 128.8 (C), 127.69 (CH), 127.66 (CH), 127.6 (CH), 127.3 (CH), 127.2 (CH), 127.0 (CH), 126.83 (CH), 126.77 (CH), 114.5 (CH), 69.2 (CH_2, Bn), 65.5 (CH_2, Bn), 65.1 (CH_2, Bn), 53.3 (CH, α-Tyr), 53.1 (CH, α-Leu), 50.7 (CH_2, Sar), 47.7 (CH, α-Ala), 44.2 (CH, α-Ala), 40.4 (CH_2, β-Leu), 35.6 (CH_2, β-Tyr), 23.8 (CH, γ-Leu), 22.2 (CH_3, δ-Leu), 21.0 (CH_3, δ-Leu), 17.3 (CH_3β-Ala), 17.0 (CH_3β-Ala). *N.B.* NCH_3 and one aromatic CH not observed; **ν_{max}** (neat) = 3277, 2955, 1719, 1632, 1452, 1174 cm^{-1}; **MS** (ESI^+) *m/z* 844 $[M+Na]^+$; **HRMS** (ESI^+) calcd. for $C_{46}H_{55}N_5NaO_9$ $[M+Na]^+$ 844.3892, found 844.3894; $[\alpha]_D^{29}$ –39.0 (*c* 0.12, MeOH).

H-Leu-Ala-Sar-Ala-Tyr-OH (91)

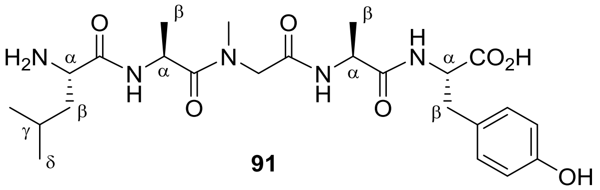

To a solution of pentapeptide **90** (821 mg, 1.0 mmol, 1.0 equiv) in MeOH (10 mL) was added 10 wt% Pd/C (82 mg, 10 wt%) and the reaction flask was evacuated, filled with nitrogen, evacuated, and placed under an atmosphere of hydrogen (balloon). The reaction mixture was stirred at room temperature for 16 h, placed under nitrogen and filtered through a plug of Celite, which was washed with MeOH (3×). The filtrate was concentrated *in vacuo* to give H-Leu-Ala-Sar-Ala-Tyr-OH (**91**) as a white solid (506 mg, 0.99 mmol, 99%), which required no further purification. **mp** 179–182 °C; **^{1}H NMR** (600 MHz, DMSO-*d6* @ 373 K) δ_H 7.00 (d, *J* = 8.2 Hz, 2H, ArH), 6.67 (d, *J* = 8.2 Hz, 2H, ArH), 4.80–4.66 (m, 1H, CHα-Ala), 4.40 (d, *J* = 5.7 Hz, 1H, CHα-Tyr), 4.36–4.29 (m, 1H, CHα-Ala), 4.05–3.97 (m, 1H, C*H*HSar), 3.57–3.46 (m, 1H, CHα-Leu), 2.97 (dd, *J* = 14.0, 5.5 Hz, 1H, C*H*Hβ-Tyr), 2.85 (dd, *J* = 14.0, 7.7 Hz, 1H, CH*H*β-Tyr), 1.79–1.69 (m, 1H, CHγ-Leu), 1.61–1.51 (m, 1H C*H*Hβ-Leu), 1.47–1.39 (m, 1H, CH*H*β-Leu), 1.24 (d, *J* = 6.5 Hz, 3H, CH_3β-Ala), 1.22 (d, *J* = 7.0 Hz, 3H, CH_3β-Ala), 0.91 (d, *J* = 6.6 Hz, 3H, CH_3δ-Leu), 0.89 (d, *J* = 6.6 Hz, 3H, CH_3δ-Leu). *N.B.* CH_2β-Tyr peaks overlap with water signal; NH protons, NCH_3 and CH*H*Sar broad and not visible; Data reported are for the major conformational isomer: **^{13}C NMR** (151 MHz, DMSO-*d6* @ 373 K) δ_C 171.9 (C=O), 171.7 (C=O), 171.2 (2 × C=O), 167.0 (C=O), 155.5 (C), 129.4 (CH), 127.1 (C), 114.7 (CH), 53.3 (CH, α-Tyr), 51.6 (CH, α-Leu), 50.3 (CH_2, Sar) 47.9 (CH, α-Ala), 44.3 (CH, α-Ala), 41.5 (CH_2, β-Leu), 35.8 (CH_2, β-Tyr), 23.4 (CH, γ-Leu), 22.2 (CH_3, δ-Leu), 21.5 (CH_3, δ-Leu), 17.4 (CH_3β-Ala). *N.B.* Both CH_3β-Ala peaks overlap, NCH_3 not observed; **ν_{max}** (neat) = 3223, 2955, 1637, 1512, 1226 cm^{-1}; **MS** (ESI^+) *m/z* 508 $[M+H]^+$, 530 $[M+Na]^+$; **HRMS** (ESI^+) calcd. for $C_{24}H_{37}N_5NaO_7$ $[M+Na]^+$ 530.2585, found 530.2587; $[\alpha]_D^{29}$ –14.0 (*c* 0.07, MeOH).

Cyclo(Leu-Ala-Sar-Ala-Tyr) (92)

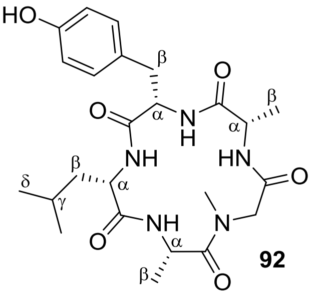

To a solution of H-Leu-Ala-Sar-Ala-Tyr-OH (**91**) (51 mg, 0.10 mmol, 1.0 equiv) in anhydrous DMF (100 mL, 0.001 M) under an atmosphere of nitrogen was added DEPBT (60 mg, 0.20 mmol, 2.0 equiv) and DIPEA (35 µL, 0.20 mmol, 2.0 equiv) and the mixture was stirred for 24 h at room temperature. The solvent was removed under reduced pressure, and the residue was purified twice by column chromatography (SiO_2, CH_2Cl_2/MeOH 92.5:7.5→9:1) to give cyclic pentapeptide **92** as a white solid (1st run: 19.0 mg, 39 µmol, 39%; 2nd run: 15.8 mg, 33 µmol, 33%). **R_f** (CH_2Cl_2/MeOH 9:1) 0.36; **mp** 179–182 °C; **^{1}H NMR** (500 MHz, CD_3OD) δ_H 7.07 (d, *J* = 8.3 Hz, 2H, ArH), 6.71 (d, *J* = 8.3 Hz, 2H, ArH), 4.85–4.83 (m, 1H, CHα-Ala), 4.49 (d, *J* = 14.0 Hz, 1H, C*H*HSar), 4.35 (t, *J* = 8.0 Hz, 1H, CHα-Tyr), 4.23 (q, *J* = 7.3 Hz, 1H, CHα-Ala), 3.83 (dd, *J* = 11.3, 4.9 Hz, 1H, CHα-Leu), 3.29–3.27 (m, 1H, CH*H*Sar), 3.16 (s, 3H, NCH_3), 3.03 (dd, *J* = 13.3, 7.8 Hz, 1H, C*H*Hβ-Tyr), 2.97 (dd, *J* = 13.3, 8.3 Hz, 1H, CH*H*β-Tyr), 2.00 (ddd, *J* = 13.8, 11.5, 4.5 Hz, 1H, C*H*Hβ-Leu), 1.50 (ddd, *J* = 14.5, 10.0, 5.0 Hz, 1H, CH*H*β-Leu), 1.32 (d, *J* = 6.7 Hz, 3H, CH_3β-Ala), 1.30 (d, *J* = 7.4 Hz, 3H, CH_3β-Ala), 1.28–1.22 (m, 1H, CHγ-Leu), 0.88 (d, *J* = 6.6 Hz, 3H, CH_3δ-Leu), 0.77 (d, *J* = 6.6 Hz, 3H, CH_3δ-Leu). *N.B.* CH*H*Sar and one CHα-Ala signal overlaps with MeOH and H_2O solvent signal respectively, assignment by HMBC & HSQC; **^{13}C NMR** (126 MHz, CD_3OD) δ_C 175.0 (C=O), 174.91 (C=O), 174.89 (C=O), 173.3 (C=O), 171.9 (C=O), 157.4 (C), 131.4 (CH), 128.9 (C), 116.2 (CH), 57.7 (CH, α-Tyr), 57.5 (CH, α-Leu), 54.4 (CH_2, Sar), 51.0 (CH, α-Ala), 46.8 (CH, α-Ala), 39.7 (CH_2, β-Leu), 37.9 (NCH_3), 36.6 (CH_2, β-Tyr), 25.7 (CH, γ-Leu), 23.5 (CH_3, δ-Leu), 21.2 (CH_3, δ-Leu), 18.3 (CH_3, β-Ala), 17.6 (CH_3, β-Ala); **ν_{max}** (neat) = 3275, 2957, 1631, 1513, 1170 cm^{-1}; **MS** (ESI^+) *m/z* 512

[M+Na]+; **HRMS** (ESI+) calcd. for $C_{24}H_{35}N_5NaO_6$ [M+Na]+ 512.2480, found 512.2484; $[\alpha]_D^{29}$ –119 (*c* 0.06, MeOH).

*2.9 Preparation of cyclic pentapeptide **98***

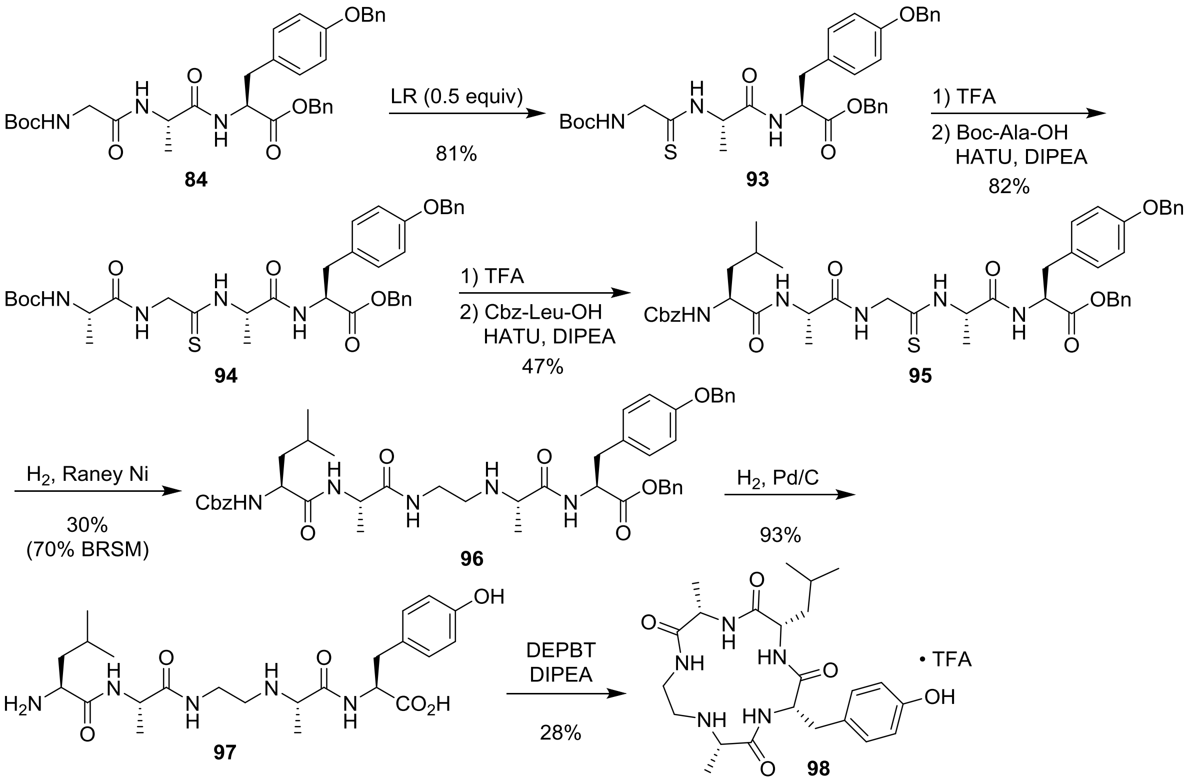

Boc-Gly-ψ[CSNH]Ala-Tyr(Bn)-OBn (93)

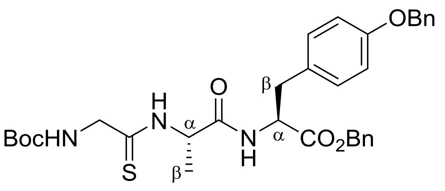

According to a modified procedure by Lawesson,[7] to a solution of Boc-Gly-Ala-Tyr(Bn)-OBn (**84**) (5.39 g, 9.14 mmol, 1.0 equiv) in anhydrous THF (5.0 mL) was added Lawesson's reagent (1.85 g, 4.57 mmol, 0.5 equiv) and the mixture was stirred at room temperature overnight. The reaction mixture was concentrated under reduced pressure and the residue was purified by column chromatography (SiO_2, PE/EtOAc 2:1→1:1) to give tripeptide **93** (4.65 g, 7.67 mmol, 84%) as a white solid. $\mathbf{R_f}$ (PE/EtOAc 1:1) 0.39; **mp** 53–55 °C; **^{1}H NMR** (400 MHz, $CDCl_3$) δ_H ppm 8.52 (s, 1H, NH), 7.45–7.28 (m, 10H, ArH), 6.92 (d, *J* = 7.8 Hz, 2H, ArH), 6.83 (d, *J* = 7.8 Hz, 2H, ArH), 6.41 (d, *J* = 7.4 Hz, 1H, NH), 5.19 (d, *J* = 12.2 Hz, 1H, C*H*HPh), 5.14 (br. s, 1H, NH), 5.11 (d, *J* = 12.2 Hz, 1H, CH*H*Ph), 5.02 (s, 2H, CH_2Ph), 4.98 (q, *J* = 6.9 Hz, 1H, CHα-Ala), 4.86 (dd, *J* = 12.9, 6.4 Hz, 1H, CHα-Tyr), 4.18 (dd, *J* = 17.1, 6.1 Hz, 1H, C*H*HGly), 4.09 (dd, *J* = 17.1, 5.6 Hz, 1H, CH*H*Gly), 3.11–3.00 (m, 2H, CH_2β-Tyr), 1.46 (s, 9H, 3 × CH_3, Boc), 1.43 (d, *J* = 6.9 Hz, 3H, CH_3β-Ala); **^{13}C NMR** (101 MHz, $CDCl_3$) δ_C ppm 199.6 (C=S), 171.1 (C=O), 170.5 (C=O), 158.1 (C), 137.1 (C), 135.1 (C), 130.6 (CH), 128.80 (CH), 128.78 (CH), 128.75 (CH), 128.7 (CH), 128.1 (CH), 127.7 (C), 127.6 (CH), 115.1 (CH), 80.7 (C, Boc), 70.1 (CH_2, Bn), 67.5 (CH_2, Bn), 53.9 (CH, α-Ala), 53.6 (CH, α-Tyr), 52.3 (CH_2, Gly), 37.1 (CH_2, β-Tyr), 28.4 (CH_3, Boc), 17.3 (CH_3, β-Ala). *N.B.* C=O, Boc not observed; $\mathbf{\nu_{max}}$ (neat) = 3308, 2977, 1667, 1509, 1239, 1159, 1023, 734, 695 cm^{-1}; **MS** (ESI+) *m/z* 628 [M+Na]+; **HRMS** (ESI+) calcd. for $C_{33}H_{39}N_3NaO_6S$ [M+Na]+ 628.2452, found 628.2455; $[\alpha]_D^{27}$ –53.8 (*c* 0.50, $CHCl_3$).

Boc-Ala-Gly-ψ[CSNH]Ala-Tyr(Bn)-OBn (94)

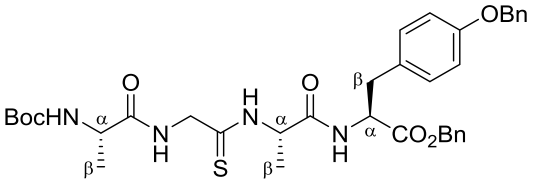

94

To a solution of Boc-Gly-ψ[CSNH]Ala-Tyr(Bn)-OBn (**93**) (4.42 g, 7.30 mmol, 1.0 equiv) in CH_2Cl_2 (8.0 mL) was added TFA (8.0 mL) and the mixture was stirred at room temperature for 1 h (*Caution – gas evolution!*). The reaction mixture was concentrated under reduced pressure and the resulting residue repeatedly dissolved in CH_2Cl_2 (3 × 15 mL) and concentrated under reduced pressure to give the crude amine. The residue was dissolved in CH_2Cl_2 (73 mL), Boc-Ala-OH (1.66 g, 8.76 mmol, 1.2 equiv), HATU (3.33 g, 8.76 mmol, 1.2 equiv) and diisopropylethylamine (5.09 mL, 29.2 mmol, 4.0 equiv) were added subsequently, and the reaction mixture was stirred at room temperature for 16 h. The mixture was washed with 10% citric acid solution (2 × 50 ml) and saturated $NaHCO_3$ solution (2 × 50 ml), dried over $MgSO_4$, filtered and concentrated under reduced pressure. The residue was purified by column chromatography (SiO_2, PE/EtOAc 1:1→EtOAc) to give tetrapeptide **94** (4.04 g, 5.96 mmol, 82%) as a white solid. **R_f** (EtOAc) 0.60; **mp** 69–71 °C; **^{1}H NMR** (400 MHz, $CDCl_3$) δ_H 8.69 (d, *J* = 6.9 Hz, 1H, NH), 7.44–7.28 (m, 10H, ArH), 7.06 (br. s, 1H, NH), 6.94 (d, *J* = 8.0 Hz, 2H, ArH), 6.81 (d, *J* = 8.0 Hz, 2H, ArH), 6.58 (br. d, *J* = 6.8 Hz, 1H, NH), 5.17 (d, *J* = 12.1 Hz, 1H, C*H*HPh), 5.12 (br. s, 1H, NH), 5.09 (d, *J* = 12.1 Hz, 1H, CH*H*Ph), 5.02–4.95 (m, 3H, CH_2Ph, CHα-Ala), 4.84 (q, *J* = 6.6 Hz, 1H, CHα-Tyr), 4.33 (dd, *J* = 17.0, 6.0 Hz, 1H, C*H*HGly), 4.23 (dd, *J* = 17.0, 4.9 Hz, 1H, CH*H*Gly), 4.08 (q, *J* = 6.8 Hz, 1H, CHα-Ala), 3.11–2.99 (m, 2H, CH_2β-Tyr), 1.46–1.42 (m, 12H, CH_3β-Ala, 3 × CH_3, Boc), 1.32 (d, *J* = 7.1 Hz, 3H, CH_3β-Ala); **^{13}C NMR** (101 MHz, $CDCl_3$) δ_C ppm 199.2 (C=S), 174.0 (C=O), 171.3 (C=O), 170.6 (C=O), 158.0 (C), 156.0 (C=O, Boc), 137.1 (C), 135.1 (C), 130.6 (CH), 128.72 (CH), 128.65 (CH), 128.1 (CH), 128.0 (C), 127.6 (CH), 115.0 (CH), 80.8 (C, Boc), 70.1 (CH_2, Bn), 67.4 (CH_2, Bn), 54.6 (CH, α-Ala), 53.6 (CH, α-Tyr), 51.0 (CH, α-Ala), 50.6 (CH_2, Gly), 37.0 (CH_2, β-Tyr), 28.5 (CH_3, Boc), 17.8 (CH_3, β-Ala), 17.1 (CH_3, β-Ala); **ν_{max}** (neat) = 3272, 2977, 1660, 1510, 1241, 1163, 1022, 841, 735, 696 cm^{-1}; **MS** (ESI^+) *m/z* 677 $[M+H]^+$, 699 $[M+Na]^+$; **HRMS** (ESI^+) calcd. for $C_{36}H_{44}N_4NaO_7S$ $[M+Na]^+$ 699.2823, found 699.2821; $[\boldsymbol{\alpha}]_D^{29}$ –29.0 (*c* 0.10, $CHCl_3$).

Cbz-Leu-Ala-Gly-ψ[CSNH]Ala-Tyr(Bn)-OBn (95)

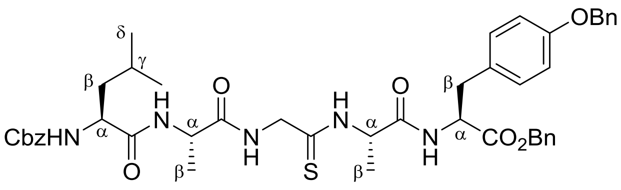

95

To a solution of Boc-Ala-Gly- ψ[CSNH]Ala-Tyr(Bn)-OBn (**94**) (676 mg, 1.00 mmol, 1.0 equiv) in CH_2Cl_2 (2.0 mL) was added TFA (2.0 mL) and the mixture was stirred at room temperature for 1 h (*Caution – gas evolution!*). The reaction mixture was concentrated under reduced pressure and the resulting residue repeatedly dissolved in CH_2Cl_2 (3 × 10 mL) and concentrated under reduced pressure to give the crude amine. The residue was dissolved in a mixture of CH_2Cl_2 (8.0 mL) and DMF (2.0 mL), Cbz-Leu-OH (531 mg, 2.00 mmol, 2.0 equiv), HATU (760 mg, 2.00 mmol, 2.0 equiv) and diisopropylethylamine (1.05 mL, 6.00 mmol, 6.0 equiv) were added subsequently, and the reaction mixture was stirred at room temperature for 16 h. The mixture was washed with 10% citric acid solution (2 × 50 ml) and saturated $NaHCO_3$ solution (2 × 50 ml), dried over $MgSO_4$, filtered and concentrated under reduced pressure. The residue was purified by column chromatography (SiO_2, PE/EtOAc 1:1→EtOAc) to give pentapeptide Cbz-Leu-Ala-Gly-ψ[CSNH]Ala-Tyr(Bn)-OBn (**95**) (388 mg, 0.47 mmol, 47%) as a white solid. **R_f** (EtOAc) 0.48; **mp** 71–73 °C; **^{1}H NMR** (400 MHz, $CDCl_3$) δ_H 8.78 (d, *J* = 6.4 Hz, 1H, NH), 7.43–7.26 (m, 16H, ArH, NH), 6.93 (d, *J* = 8.3 Hz, 2H, ArH), 6.81 (br. s, 1H, NH), 6.80 (d, *J* = 8.3 Hz, 2H, ArH), 6.64 (d, *J* = 7.8 Hz, 1H, NH), 5.51 (d, *J* = 4.8 Hz, 1H, NH), 5.17–4.98 (m, 7H, 3 × CH_2Ph, CHα-Ala), 4.82 (d, *J* = 6.8 Hz, 1H, CHα-

Tyr), 4.33–4.15 (m, 3H, CHα-Ala, CH_2Gly), 4.11 (dd, *J* = 9.3, 5.1 Hz, 1H, CHα-Leu), 3.01 (d, *J* = 5.6 Hz, 2H, CH_2β-Tyr), 1.69–1.51 (m, 3H, CH_2β-Leu, CHγ-Leu), 1.44 (d, *J* = 6.7 Hz, 3H, CH_3β-Ala), 1.29 (d, *J* = 7.0 Hz, 3H, CH_3β-Ala), 0.93 (d, *J* = 6.6 Hz, 3H, CH_3δ-Leu), 0.91 (d, *J* = 6.6 Hz, 3H, CH_3δ-Leu); **^{13}C NMR** (101 MHz, $CDCl_3$) δ_C ppm 199.4 (C=S), 173.4 (C=O), 173.1 (C=O), 171.3 (C=O), 170.8 (C=O), 158.0 (C), 137.1 (C), 136.1 (C), 135.1 (C), 130.7 (CH), 128.8 (CH), 128.74 (CH), 128.72 (CH), 128.71 (CH), 128.67 (CH), 128.6 (CH), 128.3 (CH), 128.1 (CH), 128.0 (C), 127.6 (CH), 115.0 (CH), 70.1 (CH_2, Bn), 67.7 (CH_2, Bn), 67.4 (CH_2, Bn), 54.7 (CH, α-Ala), 54.4 (CH, α-Leu), 53.7 (CH, α-Tyr), 50.8 (CH_2, Gly), 50.1 (CH, α-Ala), 40.8 (CH_2, β-Leu), 37.0 (CH_2, β-Tyr), 24.9 (CH, γ-Leu), 23.0 (CH_3, δ-Leu), 21.9 (CH_3, δ-Leu), 17.2 (CH_3, β-Ala), 17.0 (CH_3, β-Ala). *N.B.* C=O, Cbz not observed; **ν_{max}** (neat) = 3287, 2955, 1654, 1510, 1236, 1175, 1026, 735, 695 cm^{-1}; **MS** (ESI^+) *m/z* 846 $[M+Na]^+$; **HRMS** (ESI^+) calcd. for $C_{45}H_{53}N_5NaO_8S$ $[M+Na]^+$ 846.3507, found 846.3508; $[\boldsymbol{\alpha}]_D^{28}$ –33.0 (*c* 0.20, $CHCl_3$).

Cbz-Leu-Ala-NHCH_2CH_2-Ala-Tyr(Bn)-OBn (96)

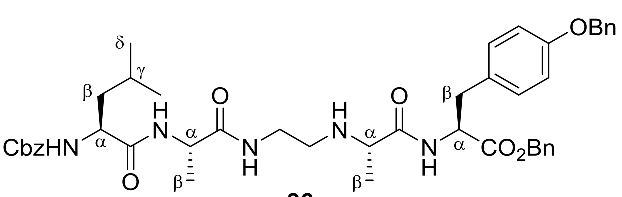

96

To a solution of Cbz-Leu-Ala-Gly-*ψ*[CSNH]Ala-Tyr(Bn)-OBn (**95**) (681 mg, 0.83 mmol, 1.0 equiv) in THF (10 mL) was added Raney Ni (slurry in H_2O, 2.0 mL). The mixture was placed under an atmosphere of nitrogen, evacuated and filled with hydrogen (balloon). The reaction mixture was stirred vigorously for 5 h at room temperature. Then, the mixture was filtered through a plug of Celite eluting with EtOAc and the filtrate was concentrated under reduced pressure. Cbz-Leu-Ala-NHCH_2CH_2-Ala-Tyr(Bn)-OBn (**96**) was afforded after purification by column chromatography (SiO_2, EtOAc→CH_2Cl_2/MeOH 19:1→9:1) as a pale-yellow waxy solid (198 mg, 0.25 mmol, 30%, 70% BRSM). **R_f** (CH_2Cl_2/MeOH 9:1) 0.29; **mp** 53–56 °C; **^{1}H NMR** (400 MHz, CD_3OD) δ_H 7.42–7.23 (m, 15H, ArH), 7.04 (d, *J* = 7.8 Hz, 2H, ArH), 6.85 (d, *J* = 7.8 Hz, 2H, ArH), 5.18–5.03 (m, 4H, 2 × CH_2Ph), 5.00 (s, 2H, CH_2Ph), 4.74 (dd, *J* = 8.1, 5.6 Hz, 1H, CHα-Tyr), 4.25 (q, *J* = 6.7 Hz, 1H, CHα-Ala), 4.13 (t, *J* = 6.4 Hz, 1H, CHα-Leu), 3.29–3.18 (m, 2H, CHα-Ala, CH_2, CONHC*H*H), 3.16–3.02 (m, 2H, CONHCH*H*, C*H*Hβ-Tyr), 2.95 (dd, *J* = 13.6, 9.0 Hz, 1H, CH*H*β-Tyr), 2.56 (dd, *J* = 11.6, 5.2 Hz, 1H, NHC*H*H), 2.43 (dd, *J* = 11.6, 5.7 Hz, 1H, NHCH*H*), 1.74–1.65 (m, 1H, CHγ-Leu), 1.60–1.49 (m, 2H, CH_2β-Leu), 1.32 (d, *J* = 6.9 Hz, 3H, CH_3β-Ala), 1.18 (d, *J* = 6.5 Hz, 3H, CH_3β-Ala), 0.93 (d, *J* = 7.4 Hz, 3H, CH_3δ-Leu), 0.91 (d, *J* = 7.4 Hz, 3H, CH_3δ-Leu); **^{13}C NMR** (101 MHz, CD_3OD) δ_C ppm 176.1 (C=O), 175.4 (C=O), 175.1 (C=O), 172.7 (C=O), 159.2 (C), 158.7 (C=O, Cbz), 138.7 (C), 138.1 (C), 136.9 (C), 131.4 (CH), 130.0 (C), 129.6 (CH), 129.54 (CH), 129.48 (CH), 129.4 (CH), 129.0 (CH), 128.8 (CH), 128.8 (CH), 128.52 (CH), 128.47 (CH), 115.9 (CH), 70.9 (CH_2, Bn), 68.2 (CH_2, Bn), 67.8 (CH_2, Bn), 58.5 (CH, α-Ala), 55.2 (CH, α-Leu), 54.8 (CH, α-Tyr), 50.7 (CH, α-Ala), 47.7 ($NHCH_2$), 41.8 (CH_2, β-Leu), 39.4 (CONHC*H*$_2$), 37.5 (CH_2, β-Tyr), 25.8 (CH, γ-Leu), 23.5 (CH_3, δ-Leu), 21.9 (CH_3, δ-Leu), 19.2 (CH_3, β-Ala), 17.7 (CH_3, β-Ala); **ν_{max}** (neat) = 3292, 2956, 1648, 1510, 1237, 1081, 1026, 735, 695 cm^{-1}; **MS** (ESI^+) *m/z* 794 $[M+H]^+$, 816 $[M+Na]^+$; **HRMS** (ESI^+) calcd. for $C_{45}H_{55}N_5NaO_8$ $[M+Na]^+$ 816.3943, found 816.3947; $[\boldsymbol{\alpha}]_D^{26}$ –23.4 (*c* 0.10, MeOH).

H-Leu-Ala-NHCH$_2$CH$_2$-Ala-Tyr-OH (97)

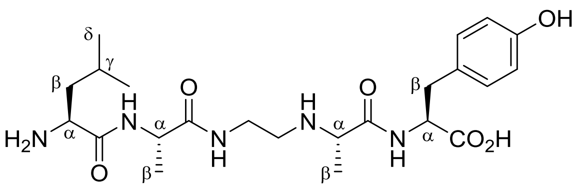

97

To a solution of Cbz-Leu-Ala-NHCH$_2$CH$_2$-Ala-Tyr(Bn)-OBn (**96**) (191 mg, 0.24 mmol, 1.0 equiv) in anhydrous MeOH (4.0 mL) was added 50 wt% Pd/C (96 mg, 10 wt%) and the reaction flask was evacuated, filled with nitrogen, evacuated, and placed under an atmosphere of hydrogen (balloon). The reaction mixture was stirred at room temperature for 18 h, placed under nitrogen and filtered through a plug of Celite, which was washed with MeOH (3×). The filtrate was concentrated *in vacuo* to give **97** as an off-white solid (110 mg, 0.22 mmol, 93%), which required no further purification. **mp** 154–158 °C; **^{1}H NMR** (400 MHz, CD$_3$OD) δ_H 7.02 (d, *J* = 7.4 Hz, 2H, ArH), 6.66 (d, *J* = 7.4 Hz, 2H, ArH), 4.51–4.40 (m, 1H, CHα-Tyr), 4.34 (q, *J* = 6.4 Hz, 1H, CHα-Ala), 3.89–3.78 (m, 1H, CHα-Leu), 3.43–3.35 (m, 1H, CONHC*H*H), 3.23–3.03 (m, 3H, CONHCH*H*, CHα-Ala, C*H*Hβ-Tyr), 2.93 (dd, *J* = 12.7, 7.7 Hz, 1H, CH*H*β-Tyr), 2.72 (dd, *J* = 12.6, 5.1 Hz, 1H, NHC*H*H), 2.48 (dd, *J* = 12.6, 6.3 Hz, 1H, NHCH*H*), 1.79–1.66 (m, 2H, CHγ-Leu, C*H*Hβ-Leu), 1.65–1.51 (m, 1H, CH*H*β-Leu), 1.31 (dd, *J* = 7.1 Hz, 3H, CH$_3$β-Ala), 1.20 (d, *J* = 6.4 Hz, 3H, CH$_3$β-Ala), 0.97 (br. s, 6H, 2 × CH$_3$δ-Leu); **^{13}C NMR** (101 MHz, CD$_3$OD) δ_C ppm 178.1 (C=O), 175.8 (C=O), 175.1 (C=O), 172.2 (C=O), 157.0 (C), 131.7 (CH), 130.0 (C), 115.9 (CH), 59.4 (CH, α-Ala), 56.7 (CH, α-Tyr), 53.4 (CH, α-Leu), 51.0 (CH, α-Ala), 48.0 (NHCH$_2$), 42.4 (CH$_2$, β-Leu), 40.2 (CONHCH), 38.6 (CH$_2$, β-Tyr), 25.5 (CH, γ-Leu), 23.2 (CH$_3$, δ-Leu), 22.4 (CH$_3$, δ-Leu), 19.5 (CH$_3$, β-Ala), 18.1 (CH$_3$, β-Ala); **ν_{max}** (neat) = 3272, 2953, 1643, 1513, 1385, 1239, 1171 cm^{-1}; **MS** (ESI$^-$) *m/z* 478 [M–H]$^-$; **HRMS** (ESI$^-$) calcd. for $C_{23}H_{36}N_5O_8$ [M–H]$^-$ 478.2671, found 478.2663; $[\boldsymbol{\alpha}]_D^{29}$ +28.8 (*c* 0.08, MeOH).

Cyclo(Leu-Ala-NHCH$_2$CH$_2$-Ala-Tyr) · TFA (98)

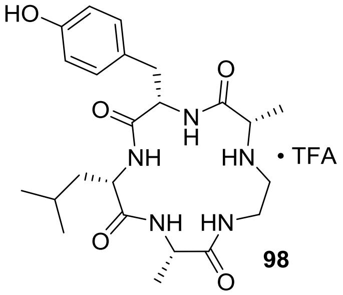

98

To a solution of **97** (49 mg, 0.10 mmol, 1.0 equiv) in anhydrous DMF (100 mL, 0.001 M) under an atmosphere of nitrogen was added DEPBT (60 mg, 0.20 mmol, 2.0 equiv) and DIPEA (35 µL, 0.20 mmol, 2.0 equiv) and the mixture was stirred for 24 h at room temperature. The solvent was removed *in vacuo*, the residue was filtered through a plug of silica eluting with CH$_2$Cl$_2$/MeOH (4:1→7:3), and the solution was concentrated *in vacuo*. The residue was purified by reverse-phase HPLC to give the TFA salt of cyclic pentapeptide **98** as a white solid (16 mg, 28 µmol, 28%). The TFA content was determined by ^{19}F NMR with 2,2,2-trifluoroethanol as internal standard. **mp** 67–69 °C; **^{1}H NMR** (500 MHz, CD$_3$OD) δ_H 6.94 (d, *J* = 8.3 Hz, 2H, ArH), 6.66 (d, *J* = 8.3 Hz, 2H, ArH), 4.48 (q, *J* = 6.9 Hz, 1H, CHα-Ala), 4.26 (dd, *J* = 10.0, 5.4 Hz, 1H, CHα-Leu), 3.89 (dd, *J* = 11.4, 5.0 Hz, 1H, CHα-Tyr), 3.68 (dd, *J* = 15.2, 11.8 Hz, 1H, CONHC*H*H), 3.60 (q, *J* = 6.8 Hz, 1H, CHα-Ala), 3.36 (dd, *J* = 12.9, 1.8 Hz, 1H, NHC*H*H), 3.08 (dd, *J* = 13.2, 4.7 Hz, 1H, CH*H*β-Tyr), 3.02 (dd, *J* = 15.2, 1.6 Hz, 1H, CONHCH*H*), 2.58 (t, *J* = 12.0 Hz, 1H, NHCH*H*), 1.76–1.60 (m, 2H, CH$_2$β-Leu), 1.59–1.49 (m, 1H, CHγ-Leu), 1.29 (d, *J* = 8.0 Hz, 3H, CH$_3$β-Ala), 1.28 (d, *J* = 7.3 Hz, 3H, CH$_3$β-Ala), 0.88 (d, *J* = 6.5 Hz, 3H, CH$_3$δ-Leu), 0.81 (d, *J* = 6.5 Hz, 3H, CH$_3$δ-Leu). *N.B.* C*H*Hβ-Tyr signal overlapping with solvent signal at 3.31 ppm; **^{13}C NMR** (126 MHz, CD$_3$OD) δ_C ppm 176.1 (C=O), 174.2 (C=O), 173.9 (C=O), 170.9 (C=O), 157.7 (C), 131.2 (CH), 129.0 (C), 116.6 (CH), 59.1 (CH, α-Tyr), 58.3 (CH, α-Ala), 55.1 (CH, α-Leu), 51.2 (NHCH$_2$), 49.6 (CH, α-Ala), 40.3 (CH$_2$, β-Leu), 37.6 (CONHCH$_2$), 34.2 (CH$_2$, β-Tyr), 26.0 (CH, γ-Leu), 23.5 (CH$_3$, δ-Leu), 21.3 (CH$_3$, δ-Leu), 17.2 (CH$_3$, β-Ala), 16.9 (CH$_3$, β-Ala); **ν_{max}** (neat) = 3307, 2920, 1651, 1515, 1182, 1131, 799, 721 cm^{-1}; **MS** (ESI$^+$) *m/z* 462 [M+H]$^+$, 484 [M+Na]$^+$; **HRMS** (ESI$^+$) calcd. for $C_{23}H_{35}N_5NaO_5$ [M+Na]$^+$ 484.2530, found 484.2534; $[\boldsymbol{\alpha}]_D^{29}$ –178 (*c* 0.04, MeOH).

*2.10 Preparation of cyclic pentapeptide **102***

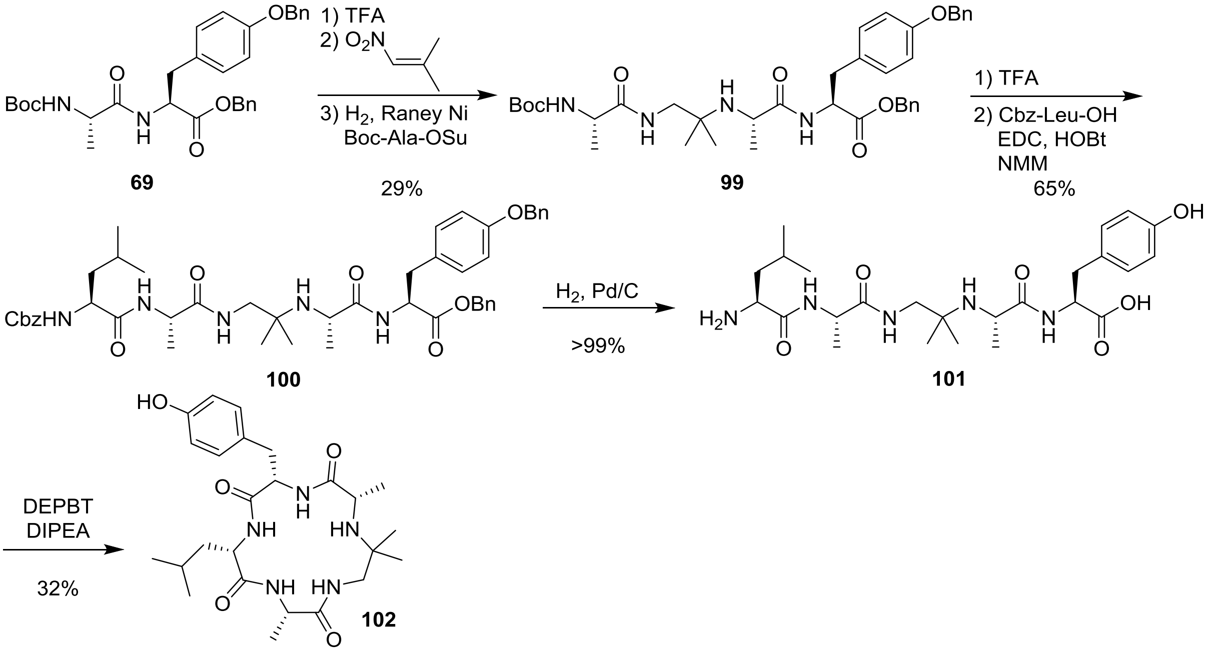

Boc-Ala-NHCH$_2$C(Me)$_2$-Ala-Tyr(Bn)-OBn (99)

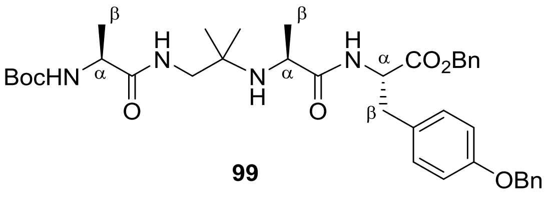

To a solution of Boc-Ala-Tyr(Bn)-OBn (**69**) (1.39 g, 2.60 mmol, 1.0 equiv) in CH_2Cl_2 (2.0 mL) was added TFA (2.0 mL) and the mixture was stirred for 40 min *(Caution – gas evolution!)*. The reaction mixture was concentrated under reduced pressure and the resulting residue repeatedly dissolved in CH_2Cl_2 (3 × 25 mL) and concentrated under reduced pressure to give the crude amine. The residue was dissolved in CH_2Cl_2 (26 mL) and NEt_3 (1.09 mL, 7.80 mmol, 3.0 equiv) and 2-methyl-1-nitroprop-1-ene (252 µL, 2.60 mmol, 1.0 equiv) were added subsequently, and the reaction mixture was stirred at room temperature for 24 h. The solvent was removed under reduced pressure to give a yellow oil which was dissolved in EtOAc/PE 1:1 and filtered through a plug of silica gel, eluting with EtOAc/PE 1:1. The eluent was concentrated under reduced pressure to reveal a colourless oil, which was suspended in THF (15 mL). Boc-Ala-OSu (842 mg, 3.00 mmol, 1.2 equiv) and Raney-Ni (slurry in H_2O, 3.0 mL) were added subsequently, the solution was placed under an atmosphere of nitrogen, evacuated and filled with hydrogen (balloon). The reaction mixture was stirred vigorously for 5 h at room temperature. Then, the mixture was filtered through a plug of Celite eluting with EtOAc, the filtrate was concentrated under reduced pressure, EtOAc (30 mL) was added, the mixture was washed with saturated Na_2CO_3 (3 × 30 mL), dried over Na_2SO_4 and concentrated under reduced pressure. Boc-Ala-NHCH$_2$C(Me)$_2$-Ala-Tyr(Bn)-OBn (**99**) was afforded after purification by column chromatography (SiO_2, EtOAc/CH_2Cl_2 1:1) as a colourless oil (502 mg, 0.75 mmol, 29%). **R$_f$** (EtOAc/CH_2Cl_2 1:1) 0.19; **^{1}H NMR** (500 MHz, $CDCl_3$) δ_H 8.01 (d, *J* = 8.0 Hz, 1H, NH), 7.45–7.28 (m, 10H, ArH), 6.92 (d, *J* = 7.7 Hz, 2H, ArH), 6.80 (d, *J* = 8.4 Hz, 2H, ArH), 6.68 (s, 1H, NH), 5.31–5.24 (m, 2H, C*H*HPh, NH Boc), 5.12 (d, *J* = 12.1 Hz, 1H, CH*H*Ph), 5.00 (s, 2H, CH$_2$Ph), 4.88 (dd, *J* = 14.5, 6.3 Hz, 1H, CHα-Tyr), 4.24–4.15 (m, 1H, CHα-Ala), 3.28 (dd, *J* = 13.9, 7.3 Hz, 1H, C*H*HGly(Me)$_2$), 3.22 (q, *J* = 7.0 Hz, 1H, CHα-Ala), 3.12 (dd, *J* = 14.0, 5.5 Hz, 1H, C*H*Hβ-Tyr), 3.04 (dd, *J* = 14.0, 6.7 Hz, 1H, CH*H*β-Tyr), 2.87 (dd, *J* = 13.9, 5.1 Hz, 1H, CH*H*Gly(Me)$_2$), 1.44 (s, 9H, 3 × CH$_3$, Boc), 1.25 (d, *J* = 6.9 Hz, 3H, CH$_3$β-Ala), 1.16 (d, *J* = 6.9 Hz, 3H, CH$_3$β-Ala), 1.00 (s, 3H, CH$_3$Gly(Me)$_2$), 0.94 (s, 3H, CH$_3$Gly(Me)$_2$). *N.B.* Amine NH signal not observed; **^{13}C NMR** (126 MHz, $CDCl_3$) δ_C 176.1 (C=O), 173.4 (C=O), 172.6 (C=O), 158.0

(C), 155.6 (C=O, Boc), 137.1 (C), 135.1 (C), 130.5 (CH), 128.83 (CH), 128.79 (CH), 128.76 (CH), 128.7 (CH), 128.3 (C), 128.1 (CH), 127.6 (CH), 114.9 (CH), 79.9 (C, Boc), 70.1 (CH_2, Bn), 67.6 (CH_2, Bn), 54.5 (C, Gly(Me)$_2$), 52.6 (CH, α-Tyr), 52.0 (CH, α-Ala), 50.3 (CH, α-Ala), 48.2 (CH_2, Gly(Me)$_2$), 37.2 (CH_2, β-Tyr), 28.5 (CH_3, Boc), 25.9 (CH_3, Gly(Me)$_2$), 23.9 (CH_3, Gly(Me)$_2$), 21.9 (CH_3, β-Ala), 18.5 (CH_3, β-Ala); **ν_{max}** (neat) = 3314, 2972, 1737, 1656, 1509, 1165 cm^{-1}; **MS** (ESI$^+$) *m/z* 675 [M+H]$^+$, 697 [M+Na]$^+$; **HRMS** (ESI$^+$) calcd. for $C_{38}H_{51}N_4O_7$ [M+H]$^+$ 675.3752, found 675.3756; $[\boldsymbol{\alpha}]_D^{29}$ –26.0 (*c* 0.07, MeOH).

Cbz-Leu-Ala-NHCH$_2$C(Me)$_2$-Ala-Tyr(Bn)-OBn (100)

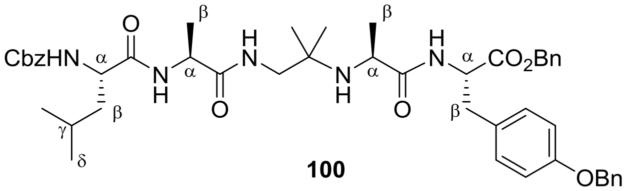

100

To a solution of Boc-Ala-NHCH$_2$C(Me)$_2$-Ala-Tyr(Bn)-OBn (**99**) (472 mg, 0.70 mmol, 1.0 equiv) in CH_2Cl_2 (1.0 mL) was added TFA (1.0 mL) and the mixture was stirred at room temperature for 1 h *(Gas evolution!)*. The reaction mixture was concentrated *in vacuo* and the resulting residue repeatedly dissolved in CH_2Cl_2 (3 × 15 mL) and concentrated under reduced pressure to give the crude amine. The residue was dissolved in CH_2Cl_2 (7.0 mL), Cbz-Leu-OH (186 mg, 0.70 mmol, 1.0 equiv), EDC·HCl (134 mg, 0.70 mmol, 1.0 equiv), HOBt·H_2O (95 mg, 0.70 mmol, 1.0 equiv) and NMM (310 µL, 2.80 mmol, 4.0 equiv) were added subsequently, and the mixture was stirred at room temperature for 24 h. The reaction mixture was diluted with EtOAc (10 mL) and washed with brine (3 × 15 mL), dried over Na_2SO_4, filtered and concentrated *in vacuo*. The residue was purified by column chromatography (SiO_2, CH_2Cl_2/MeOH 96.5:3.5) to give Cbz-Leu-Ala-NHCH$_2$C(Me)$_2$-Ala-Tyr(Bn)-OBn (**100**) (373 mg, 0.45 mmol, 65%) as a white foam. **R_f** (CH_2Cl_2/MeOH 19:1) 0.45; **mp** 67–70 ºC; **^{1}H NMR** (500 MHz, $CDCl_3$) δ_H 8.02 (d, *J* = 8.4 Hz, 1H, NH), 7.43–7.28 (m, 15H, ArH), 6.93 (d, *J* = 8.4 Hz, 2H, ArH), 6.86–6.78 (m, 4H, ArH, 2 × NH), 5.24 (d, *J* = 12.1 Hz, 1H, C*H*HPh), 5.20–4.98 (m, 6H, NH Cbz, CH*H*Ph, 2 × CH_2Ph), 4.92–4.85 (m, 1H, CHα-Tyr), 4.49 (quint, *J* = 7.1 Hz, 1H, CHα-Ala), 4.18 (m, 1H, CHα-Leu), 3.28–3.17 (m, 2H, CHα-Ala, C*H*HGly(Me)$_2$), 3.12 (dd, *J* = 14.0, 5.6 Hz, 1H, C*H*Hβ-Tyr), 3.03 (dd, *J* = 14.0, 6.8 Hz, 1H, CH*H*β-Tyr), 2.91 (dd, *J* = 13.9, 5.3 Hz, 1H, CH*H*Gly(Me)$_2$), 1.71–1.58 (m, 2H, CHγ-Leu, C*H*Hβ-Leu), 1.55–1.46 (m, 1H, CH*H*β-Leu), 1.25 (d, *J* = 7.0 Hz, 3H, CH_3β-Ala), 1.16 (d, *J* = 7.0 Hz, 3H, CH_3β-Ala), 1.00 (s, 3H, CH_3Gly(Me)$_2$), 0.97–0.86 (m, 9H, CH_3Gly(Me)$_2$, 2 × CH_3δ-Leu). *N.B.* Amine signal NH not observed; **^{13}C NMR** (126 MHz, $CDCl_3$) δ_C 176.2 (C=O), 172.8 (C=O), 172.6 (C=O), 172.0 (C=O), 158.0 (C), 156.4 (C=O, Cbz), 137.1 (C), 136.1 (C), 135.0 (C), 130.5 (CH), 128.81 (CH), 128.75 (CH), 128.73 (CH), 128.71 (CH), 128.69 (CH), 128.5 (CH), 128.3 (C), 128.2 (CH), 128.1 (CH), 127.6 (CH), 114.9 (CH), 70.1 (CH_2, Bn), 67.7 (CH_2, Bn), 67.3 (CH_2, Bn), 54.5 (C, Gly(Me)$_2$), 54.0 (CH, α-Leu), 52.6 (CH, α-Tyr), 52.0 (CH, α-Ala), 49.1 (CH, α-Ala), 48.7 (CH_2, Gly(Me)$_2$), 41.7 (CH_2, β-Leu), 37.2 (CH_2, β-Tyr), 25.9 (CH, γ-Leu), 24.9 (CH_3, Gly(Me)$_2$), 23.7 (CH_3, Gly(Me)$_2$), 23.1 (CH_3, δ-Leu), 21.9 (CH_3, δ-Leu), 21.8 (CH_3, β-Ala), 18.1 (CH_3, β-Ala); **ν_{max}** (neat) = 3293, 2961, 1705, 1645, 1509, 1236 cm^{-1}; **MS** (ESI$^+$) *m/z* 822 [M+H]$^+$, 844 [M+Na]$^+$; **HRMS** (ESI$^+$) calcd. for $C_{47}H_{60}N_5O_8$ [M+H]$^+$ 822.4436, found 822.4428; $[\boldsymbol{\alpha}]_D^{29}$ –37.0 (*c* 0.11, MeOH).

H-Leu-Ala-NHCH$_2$C(Me)$_2$-Ala-Tyr-OH (101)

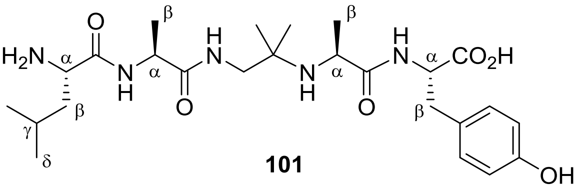

101

To a solution of pentapeptide **100** (329 mg, 0.40 mmol, 1.0 equiv) in MeOH (4.0 mL) was added 10 wt% Pd/C (33 mg, 10 wt%) and the reaction flask was evacuated, filled with nitrogen, evacuated, and placed under an atmosphere of hydrogen (balloon). The mixture was stirred at room temperature for 16 h, placed under nitrogen and filtered through a plug of Celite, which was washed with MeOH (3×). The filtrate was concentrated *in vacuo* to give pentapeptide **101** as a white solid (203 mg, 0.40 mmol, quant. yield). **mp** 144–147 ºC; **^{1}H NMR** (500 MHz, CD$_3$OD) δ_H 7.03 (d, *J* = 8.3 Hz, 2H, ArH), 6.65 (d, *J* = 8.3 Hz, 2H, ArH), 4.46 (dd, *J* = 8.0, 4.7 Hz, 1H, CHα-Tyr), 4.38 (q, *J* = 7.1 Hz, 1H, CHα-Ala), 3.99–3.93 (m, 1H, CHα-Leu), 3.55 (q, *J* = 6.6 Hz, 1H, CHα-Ala), 3.31–3.28 (m, 1H, C*H*HGly(Me)$_2$), 3.12 (dd, *J* = 13.9, 4.6 Hz, 1H, C*H*Hβ-Tyr), 2.90 (dd, *J* = 13.9, 8.2 Hz, 1H, CH*H*β-Tyr), 2.74 (d, *J* = 14.0 Hz, 1H, CH*H*Gly(Me)$_2$), 1.81–1.62 (m, 3H, CHγ-Leu, CH$_2$β-Leu), 1.34 (d, *J* = 7.2 Hz, 3H, CH$_3$β-Ala), 1.31 (d, *J* = 7.0 Hz, 3H, CH$_3$β-Ala), 1.05 (s, 3H, CH$_3$Gly(Me)$_2$), 1.01 (s, 3H, CH$_3$Gly(Me)$_2$), 0.99 (d, *J* = 5.8 Hz, 3H, CH$_3$δ-Leu), 0.97 (d, *J* = 5.8 Hz, 3H, CH$_3$δ-Leu). *N.B.* C*H*HGly(Me)$_2$ signal partially overlaps with solvent peak; **^{13}C NMR** (126 MHz, CD$_3$OD) δ_C 177.0 (C=O), 175.3 (2 × C=O), 170.8 (C=O), 157.2 (C), 131.5 (CH), 129.8 (C), 116.1 (CH), 58.1 (C, Gly(Me)$_2$), 56.7 (CH, α-Tyr), 53.1 (CH, α-Leu), 53.0 (CH, α-Ala), 51.2 (CH, α-Ala), 48.3 (CH$_2$, Gly(Me)$_2$), 41.7 (CH$_2$, β-Leu), 38.4 (CH$_2$, β-Tyr), 25.4 (CH, γ-Leu), 24.4 (CH$_3$, Gly(Me)$_2$), 23.3 (CH$_3$, Gly(Me)$_2$), 23.2 (CH$_3$, δ-Leu), 22.2 (CH$_3$, δ-Leu), 20.4 (CH$_3$, β-Ala), 18.0 (CH$_3$, β-Ala); **ν_{max}** (neat) = 3226, 1655, 1512, 1449, 1234 cm^{-1}; **MS** (ESI$^+$) *m/z* 508 [M+H]$^+$, 530 [M+Na]$^+$; **HRMS** (ESI$^+$) calcd. for $C_{25}H_{42}N_5O_6$ [M+H]$^+$ 508.3130, found 508.3133; $[\alpha]_D^{29}$ +4.0 (*c* 0.07, MeOH).

Cyclo(Leu-Ala-NHCH$_2$C(Me)$_2$-Ala-Tyr) (102)

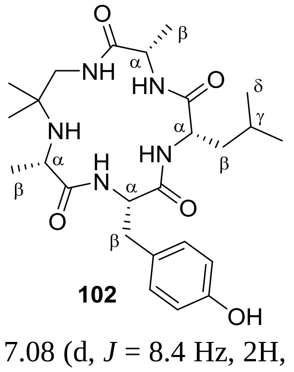

102

To a solution of H-Leu-Ala-NHCH$_2$C(Me)$_2$-Ala-Tyr-OH (**101**) (50.7 mg, 0.10 mmol, 1.0 equiv) in anhydrous DMF (100 mL, 0.001 M) under an atmosphere of nitrogen was added DEPBT (60 mg, 0.20 mmol, 2.0 equiv) and DIPEA (35 μL, 0.20 mmol, 2.0 equiv) and the reaction mixture was stirred for 24 h at room temperature. The solvent was removed under reduced pressure and the residue was purified twice by column chromatography (SiO$_2$, CH$_2$Cl$_2$/MeOH 19:1→9:1) to give the cyclic pentapeptide (**102**) as a white solid (1st run: 15.9 mg, 33 μmol, 33%; 2nd run: 15.4 mg, 31 μmol, 31%). **R$_f$** (CH$_2$Cl$_2$/MeOH 9:1) 0.38; **mp** 178–180 ºC; **^{1}H NMR** (500 MHz, CD$_3$OD) δ_H 7.08 (d, *J* = 8.4 Hz, 2H, ArH), 6.71 (d, *J* = 8.4 Hz, 2H, ArH), 4.41 (q, *J* = 7.2 Hz, 1H, CHα-Ala), 4.35 (t, *J* = 7.9 Hz, 1H, CHα-Tyr), 4.14 (dd, *J* = 10.5, 4.9 Hz, 1H, CHα-Leu), 3.28 (q, *J* = 7.0 Hz, 1H, CHα-Ala), 3.16 (d, *J* = 13.9 Hz, 1H, C*H*HGly(Me)$_2$), 3.12 (dd, *J* = 13.6, 9.3 Hz, 1H, C*H*Hβ-Tyr), 3.06 (dd, *J* = 13.6, 7.5 Hz, 1H, CH*H*β-Tyr), 2.97 (d, *J* = 13.9 Hz, 1H, CH*H*Gly(Me)$_2$), 1.85 (ddd, *J* = 14.0, 10.9, 4.9 Hz, 1H, C*H*Hβ-Leu), 1.61–1.52 (m, 1H, CH*H*β-Leu), 1.43 (d, *J* = 7.2 Hz, 3H, CH$_3$β-Ala), 1.41–1.33 (m, 1H, CHγ-Leu), 1.17 (d, *J* = 7.0 Hz, 3H, CH$_3$β-Ala), 1.06 (s, 3H, CH$_3$Gly(Me)$_2$), 0.95–0.89 (m, 6H, CH$_3$Gly(Me)$_2$, CH$_3$δ-Leu), 0.83 (d, *J* = 6.5 Hz, 3H, CH$_3$δ-Leu). *N.B.* CHα-Ala signal partially overlaps with solvent signal; **^{13}C NMR** (126 MHz, CD$_3$OD) δ_C 179.3 (C=O), 175.3 (C=O), 174.6 (C=O), 173.7 (C=O), 157.4 (C), 131.3 (C), 128.9 (CH), 116.3 (CH), 57.6 (CH, α-Tyr), 56.2 (CH, α-Leu), 55.9 (C, Gly(Me)$_2$), 52.8 (CH, α-Ala), 51.4 (CH, α-Ala), 49.2 (CH$_2$, Gly(Me)$_2$), 40.8 (CH$_2$, β-Leu), 36.3 (CH$_2$, β-Tyr), 26.6 (CH$_3$, Gly(Me)$_2$), 25.8 (CH, γ-Leu), 23.4 (CH$_3$, δ-Leu), 22.8 (CH$_3$, Gly(Me)$_2$), 21.9 (CH$_3$, β-Ala), 21.6 (CH$_3$, δ-Leu), 18.3 (CH$_3$, β-Ala); **ν_{max}** (neat) = 3259, 2960, 1649, 1513, 1448, 1302 cm^{-1}; **MS** (ESI$^+$) *m/z* 490 [M+H]$^+$, 512 [M+Na]$^+$; **HRMS** (ESI$^+$) calcd. for $C_{25}H_{40}N_5O_5$ [M+H]$^+$ 490.3024, found 490.3027; $[\alpha]_D^{29}$ –71.0 (*c* 0.04, MeOH).

*2.11 Preparation of cyclic pentapeptide **107***

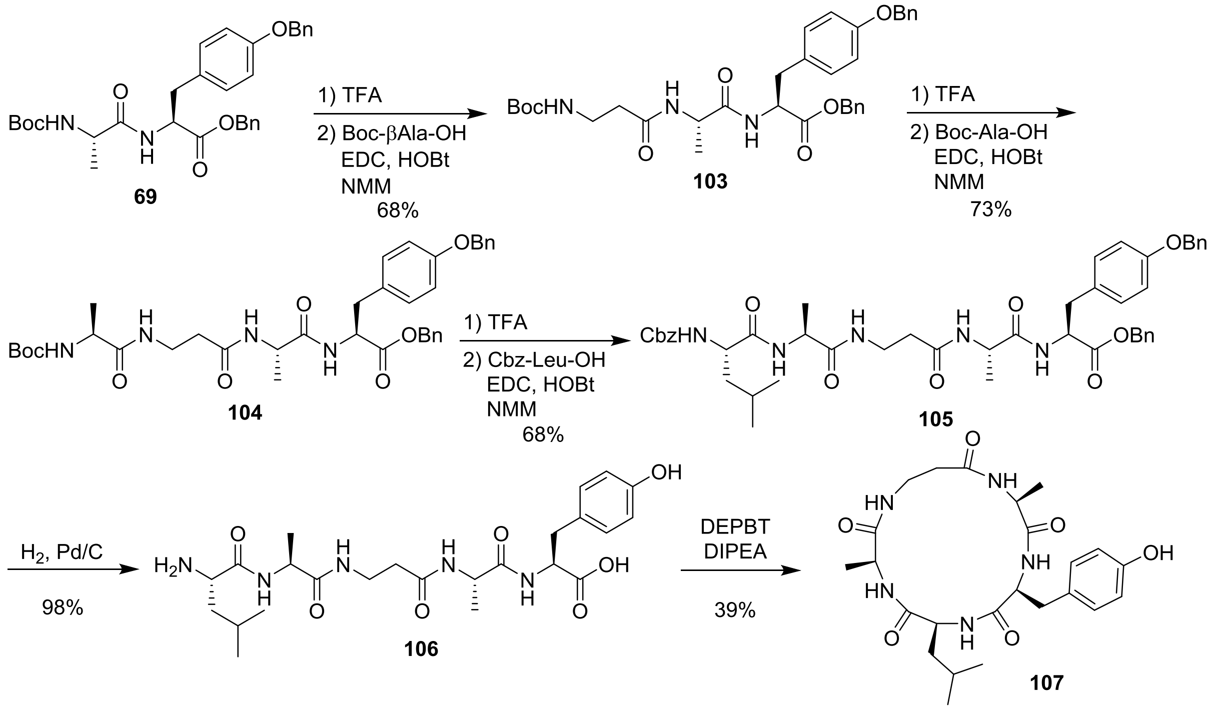

Boc-βAla-Ala-Tyr(Bn)-OBn (103)

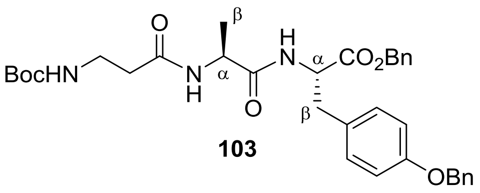

To a solution of Boc-Ala-Tyr(Bn)-OBn (**69**) (3.19 g, 6.00 mmol, 1.0 equiv) in CH_2Cl_2 (6.0 mL) was added TFA (6.0 mL) and the mixture was stirred at room temperature for 1 h *(Gas evolution!)*. The mixture was concentrated under reduced pressure and the resulting residue repeatedly dissolved in CH_2Cl_2 (3 × 25 mL) and concentrated *in vacuo* to give the crude amine. The residue was dissolved in CH_2Cl_2 (60 mL), Boc-βAla-OH (1.14 g, 6.00 mmol, 1.0 equiv), EDC·HCl (1.15 g, 6.00 mmol, 1.0 equiv), HOBt·H_2O (0.81 g, 6.00 mmol, 1.0 equiv) and NMM (2.64 mL, 24.0 mmol, 4.0 equiv) were added, and the mixture was stirred at room temperature for 24 h. The mixture was diluted with EtOAc (60 mL) and washed with brine (3 × 100 mL), dried over Na_2SO_4, filtered and concentrated *in vacuo*. The residue was purified by column chromatography (SiO_2, EtOAc/PE 7:3) to give Boc-βAla-Ala-Tyr(Bn)-OBn (**103**) (2.47 g, 4.10 mmol, 68%) as a white solid. $\mathbf{R_f}$ (EtOAc/PE 7:3) 0.20; **mp** 154–157 °C; ^{1}**H NMR** (500 MHz, DMSO-*d6*) δ_H 8.28 (d, *J* = 7.4 Hz, 1H, NH), 7.99 (d, *J* = 7.7 Hz, 1H, NH), 7.46–7.23 (m, 10H, ArH), 7.11 (d, *J* = 8.5 Hz, 2H, ArH), 6.89 (d, *J* = 8.5 Hz, 2H, ArH), 6.71 (t, *J* = 5.3 Hz, 1H, NH), 5.05 (s, 4H, 2 × CH_2Ph), 4.44 (dd, *J* = 14.5, 7.4 Hz, 1H, CHα-Tyr), 4.30 (quint, *J* = 7.1 Hz, 1H, CHα-Ala), 3.10 (dd, *J* = 13.4, 6.8 Hz, 2H, C*H*$_2$NHBoc), 2.97 (dd, *J* = 13.8, 6.2 Hz, 1H, C*H*Hβ-Tyr), 2.89 (dd, *J* = 13.8, 8.4 Hz, 1H, CH*H*β-Tyr), 2.24 (t, *J* = 7.3 Hz, 2H, CH_2CONH), 1.36 (s, 9H, 3 × CH_3, Boc), 1.11 (d, *J* = 7.1 Hz, 3H, CH_3β-Ala); ^{13}**C NMR** (126 MHz, DMSO-*d6*) δ_C 173.0 (C=O), 171.7 (C=O), 170.5 (C=O), 157.6 (C), 155.9 (C=O, Boc), 137.6 (C), 136.2 (C), 130.7 (CH), 129.5 (C), 128.9 (CH), 128.8 (CH), 128.5 (CH), 128.4 (CH), 128.3 (CH), 128.1 (CH), 115.0 (CH), 78.0 (C, Boc), 69.6 (CH_2, Bn), 66.4 (CH_2, Bn), 54.4 (CH, α-Tyr), 48.1 (CH, α-Ala), 37.1 (CH_2NHBoc), 36.2 (CH_2, β-Tyr), 36.0 (*C*H_2CONH), 28.7 (CH_3, Boc), 18.6 (CH_3, β-Ala); $\mathbf{\nu_{max}}$ (neat) = 3342, 3300, 1728, 1687, 1632, 1176 cm^{-1}; **MS** (ESI^+) *m/z* 626 $[M+Na]^+$; **HRMS** (ESI^+) calcd. for $C_{34}H_{41}N_3NaO_7$ $[M+Na]^+$ 626.2837, found 626.2841; $[\alpha]_D^{29}$ –10.0 (*c* 0.08, DMF).

Boc-Ala-βAla-Ala-Tyr(Bn)-OBn (104)

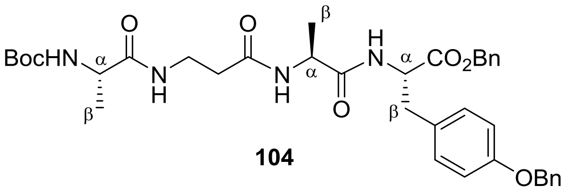

104

To a solution of Boc-βAla-Ala-Tyr(Bn)-OBn (**103**) (2.32 g, 3.86 mmol, 1.0 equiv) in CH_2Cl_2 (10 mL) was added TFA (10 mL) and the mixture was stirred at room temperature for 1 h *(Gas evolution!)*. The mixture was concentrated under reduced pressure and the resulting residue repeatedly dissolved in CH_2Cl_2 (3 × 25 mL) and concentrated *in vacuo* to give the crude amine. The residue was dissolved in CH_2Cl_2 (40 mL), Boc-Ala-OH (0.73 g, 3.86 mmol, 1.0 equiv), EDC·HCl (0.74 g, 3.86 mmol, 1.0 equiv), HOBt·H_2O (0.52 g, 3.86 mmol, 1.0 equiv) and NMM (1.70 mL, 15.4 mmol, 4.0 equiv) were added, and the mixture was stirred at room temperature for 24 h. The mixture was diluted with EtOAc (40 mL) and washed with brine (3 × 60 mL), dried over Na_2SO_4, filtered and concentrated *in vacuo*. The residue was purified by column chromatography (SiO_2, CH_2Cl_2/MeOH 24:1) to give Boc-Ala-βAla-Ala-Tyr(Bn)-OBn (**104**) (1.84 g, 2.72 mmol, 73%) as a white solid. $\mathbf{R_f}$ (CH_2Cl_2/MeOH 24:1) 0.16; **mp** 158–161 ºC; **^{1}H NMR** (500 MHz, CD_2Cl_2) δ_H 7.45–7.31 (m, 11H, ArH, NH), 6.97–6.90 (m, 3H, ArH, NH), 6.84–6.78 (m, 3H, ArH, NH), 5.27 (d, *J* = 6.7 Hz, 1H, Boc NH), 5.20 (d, *J* = 12.1 Hz, 1H, C*H*HPh), 5.11 (d, *J* = 12.1 Hz, 1H, CH*H*Ph), 5.04–4.97 (m, 2H, CH_2Ph), 4.88 (dd, *J* = 12.8, 5.9 Hz, 1H, CHα-Tyr), 4.38–4.29 (m, 1H, CHα-Ala), 3.80–3.72 (m, 1H, CHα-Ala), 3.72–3.62 (m, 1H, C*H*HNH), 3.25–3.15 (m, 1H, CH*H*NH), 3.07 (dd, *J* = 14.0, 5.7 Hz, 1H, C*H*Hβ-Tyr), 3.01 (dd, *J* = 14.0, 6.2 Hz, 1H, CH*H*β-Tyr), 2.47–2.36 (m, 1H, C*H*HCONH), 2.24 (m, 1H, CH*H*CONH), 1.43 (s, 9H, 3 × CH_3, Boc), 1.33 (d, *J* = 7.2 Hz, 3H, CH_3β-Ala), 1.23 (d, *J* = 6.9 Hz, 3H, CH_3β-Ala); **^{13}C NMR** (126 MHz, CD_2Cl_2) δ_C 174.1 (C=O), 173.3 (C=O), 172.7 (C=O), 172.6 (C=O), 158.4 (C), 156.5 (C=O, Boc), 137.7 (C), 135.7 (C), 131.0 (CH), 129.2 (CH), 129.13 (CH), 129.11 (CH), 129.07 (CH), 128.6 (C) 128.5 (CH), 128.1 (CH), 115.2 (CH), 80.5 (C, Boc), 70.4 (CH_2, Bn), 68.0 (CH_2, Bn), 53.9 (CH, α-Tyr), 51.2 (CH, α-Ala), 50.7 (CH, α-Ala), 37.5 (CH_2, β-Tyr), 37.2 (CH_2NH), 36.9 (*C*H_2CONH), 28.7 (CH_3, Boc), 18.2 (CH_3, β-Ala), 18.0 (CH_3, β-Ala); $\mathbf{\nu_{max}}$ (neat) = 3288, 2975, 1732, 1646, 1632, 1163 cm^{-1}; **MS** (ESI^+) *m/z* 697 $[M+Na]^+$; **HRMS** (ESI^+) calcd. for $C_{37}H_{46}N_4NaO_8$ $[M+Na]^+$ 697.3208, found 697.3210; $[\boldsymbol{\alpha}]_D^{29}$ –9.0 (*c* 0.14, DMF).

Cbz-Leu-Ala-βAla-Ala-Tyr(Bn)-OBn (105)

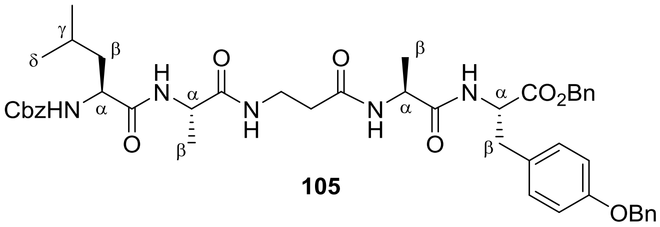

105

To a solution of Boc-Ala-βAla-Ala-Tyr(Bn)-OBn (**104**) (1.54 g, 2.28 mmol, 1.0 equiv) in CH_2Cl_2 (5.0 mL) was added TFA (5.0 mL) and the mixture was stirred at room temperature for 1 h *(Gas evolution!)*. The mixture was concentrated under reduced pressure and the resulting residue repeatedly dissolved in CH_2Cl_2 (3 × 25 mL) and concentrated *in vacuo* to give the crude amine. The residue was dissolved in CH_2Cl_2 (23 mL), Cbz-Leu-OH (0.60 g, 2.28 mmol, 1.0 equiv), EDC·HCl (0.44 g, 2.28 mmol, 1.0 equiv), HOBt·H_2O (0.31 g, 2.28 mmol, 1.0 equiv) and NMM (1.60 mL, 9.11 mmol, 4.0 equiv) were added, and the mixture was stirred at room temperature for 24 h. The mixture was diluted with EtOAc (25 mL) and washed with brine (3 × 40 mL), dried over Na_2SO_4, filtered and concentrated *in vacuo*. The residue was purified by column chromatography (SiO_2, CH_2Cl_2/MeOH 19:1) to give Cbz-Leu-Ala-βAla-Ala-Tyr(Bn)-OBn (**105**) (1.27 g, 1.55 mmol, 68%) as a white solid. $\mathbf{R_f}$ (CH_2Cl_2/MeOH 19:1) 0.46; **mp** 176–179 ºC; **^{1}H NMR** (500 MHz, DMSO-*d6*) δ_H 8.31 (d, *J* = 7.4 Hz, 1H, NH), 8.03 (d, *J* = 7.7 Hz, 1H, NH), 7.91 (d, *J* = 7.4 Hz, 1H, NH), 7.86 (t, *J* = 5.5 Hz, 1H, NH), 7.48–7.24 (m, 16H, NH, ArH), 7.12 (d, *J* = 8.4 Hz, 2H, ArH), 6.90 (d, *J* = 8.5 Hz, 2H, ArH), 5.11–5.01 (m, 6H, CH_2Ph), 4.45 (dd, *J* = 14.6, 7.4 Hz, 1H, CHα-Tyr), 4.33 (quint, *J* = 7.1 Hz, 1H, CHα-

Ala), 4.21 (quint, *J* = 7.0 Hz, 1H, CHα-Ala), 4.04 (dd, *J* = 14.8, 8.5 Hz, 1H, CHα-Leu), 3.31–3.17 (m, 2H, C*H*$_2$NH), 2.97 (dd, *J* = 13.8, 6.3 Hz, 1H, C*H*Hβ-Tyr), 2.91 (dd, *J* = 13.8, 8.4 Hz, 1H, CH*H*β-Tyr), 2.32–2.20 (m, 2H, C*H*$_2$CONH), 1.67–1.56 (m, 1H, CHγ-Leu), 1.48–1.38 (m, 2H, CH$_2$β-Leu), 1.17 (d, *J* = 7.0 Hz, 3H, CH$_3$β-Ala), 1.13 (d, *J* = 7.0 Hz, 3H, CH$_3$β-Ala), 0.87 (d, *J* = 7.4 Hz, 3H, CH$_3$δ-Leu), 0.85 (d, *J* = 7.4 Hz, 3H, CH$_3$δ-Leu); **^{13}C NMR** (126 MHz, DMSO-*d6*) δ_C 172.6 (C=O), 171.9 (C=O), 171.9 (C=O), 171.3 (C=O), 170.0 (C=O), 157.2 (C=O, Cbz), 156.0 (C), 137.2 (C), 137.1 (C), 135.7 (C), 130.2 (CH), 129.0 (C), 128.4 (CH), 128.3 (CH), 128.0 (CH), 127.9 (CH), 127.8 (CH), 127.8 (CH), 127.6 (CH), 127.6 (CH), 114.5 (CH), 69.1 (CH$_2$, Bn), 66.0 (CH$_2$, Bn), 65.4 (CH$_2$, Bn), 54.0 (CH, α-Tyr), 53.1 (CH, α-Leu), 48.1 (CH, α-Ala), 47.7 (CH, α-Ala), 40.6 (CH$_2$, β-Leu), 35.7 (CH$_2$, β-Tyr), 35.3 (CH$_2$NH), 35.0 (*C*H$_2$CONH), 24.2 (CH, γ-Leu), 23.1 (CH$_3$, δ-Leu), 21.3 (CH$_3$, δ-Leu), 18.5 (CH$_3$, β-Ala), 18.2 (CH$_3$, β-Ala). *N.B.* One aromatic CH signal not observed due to overlapping; **ν_{max}** (neat) = 3279, 3065, 1635, 1385, 693 cm^{-1}; **MS** (ESI$^+$) *m/z* 844 [M+Na]$^+$; **HRMS** (ESI$^+$) calcd. for $C_{46}H_{55}N_5NaO_9$ [M+Na]$^+$ 844.3892, found 844.3899; $[\alpha]_D^{29}$ +3.0 (*c* 0.13, DMF).

H-Leu-Ala-βAla-Ala-Tyr-OH (106)

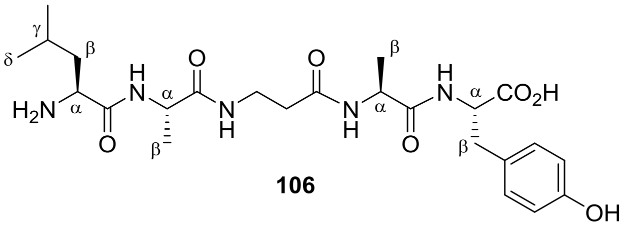

106

To a solution of Cbz-Leu-Ala-βAla-Ala-Tyr(Bn)-OBn (**105**) (1.07 g, 1.31 mmol, 1.0 equiv) in MeOH/DMF (1:1, 15 mL) was added 10 wt% Pd/C (110 mg, 10 wt%) and the reaction flask was evacuated, filled with nitrogen, evacuated, and placed under an atmosphere of hydrogen (balloon). The reaction mixture was stirred at room temperature for 16 h, placed under nitrogen and filtered through a plug of Celite, which was washed with MeOH (3×). The filtrate was concentrated *in vacuo* to give H-Leu-Ala-βAla-Ala-Tyr-OH (**106**) as a DMF adduct as a white solid (647 mg, 1.28 mmol, 98%), which required no further purification. **mp** 89–92 ºC; **^{1}H NMR** (500 MHz, CD$_3$OD) δ_H 7.02 (d, *J* = 8.3 Hz, 2H, ArH), 6.67 (d, *J* = 8.3 Hz, 2H, ArH), 4.43 (t, *J* = 5.5 Hz, 1H, CHα-Tyr), 4.29 (q, *J* = 7.2 Hz, 1H, CHα-Ala), 4.26 (q, *J* = 7.3 Hz, 1H, CHα-Ala), 3.97 (dd, *J* = 8.4, 6.0 Hz, 1H, CHα-Leu), 3.56 (ddd, *J* = 13.4, 6.1, 4.7 Hz, 1H, C*H*HNH), 3.38–3.34 (1H, m, CH*H*NH), 3.10 (dd, *J* = 13.6, 5.0 Hz, 1H, C*H*HCONH), 3.00 (dd, *J* = 13.6, 6.0 Hz, 1H, CH*H*bCONH), 2.45 (ddd, *J* = 13.4, 8.9, 4.3 Hz, 1H, C*H*Hβ-Tyr), 2.36 (ddd, *J* = 14.8, 5.9, 4.3 Hz, 1H, CH*H*β-Tyr), 1.84–1.63 (m, 3H, CHγ-Leu, CH$_2$β-Leu), 1.35 (d, *J* = 7.2 Hz, 3H, CH$_3$β-Ala), 1.31 (d, *J* = 7.2 Hz, 3H, CH$_3$β-Ala), 1.02 (d, *J* = 6.4 Hz, 3H, CH$_3$δ-Leu), 1.00 (d, *J* = 6.4 Hz, 3H, CH$_3$δ-Leu); **^{13}C NMR** (126 MHz, CD$_3$OD) δ_C 177.0 (C=O), 174.6 (C=O), 174.0 (C=O), 173.9 (C=O), 170.6 (C=O), 157.0 (C), 131.7 (CH), 129.5 (C), 115.9 (CH), 56.4 (CH, α-Tyr), 53.2 (CH, α-Leu), 51.2 (CH, α-Ala), 50.9 (CH, α-Ala), 41.6 (CH$_2$, β-Leu), 37.9 (CH$_2$NH), 36.8 (*C*H$_2$CONH), 36.4 (CH$_2$, β-Tyr), 25.5 (CH, γ-Leu), 23.1 (CH$_3$, δ-Leu), 22.1 (CH$_3$, δ-Leu), 18.1 (CH$_3$, β-Ala), 17.7 (CH$_3$, β-Ala); **ν_{max}** (neat) = 3263, 2932, 1644, 1512, 1189 cm^{-1}; **MS** (ESI$^+$) *m/z* 508 [M+H]$^+$, 530 [M+Na]$^+$; **HRMS** (ESI$^+$) calcd. for $C_{24}H_{37}N_5NaO_7$ [M+Na]$^+$ 530.2585, found 530.2589; $[\alpha]_D^{29}$ +3.0 (*c* 0.07, DMF).

Cyclo(Leu-Ala-βAla-Ala-Tyr) (107)

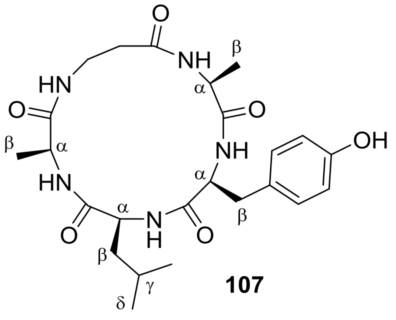

To a solution of H-Leu-Ala-βAla-Ala-Tyr-OH (**106**) (51 mg, 0.10 mmol, 1.0 equiv) in anhydrous DMF (100 mL, 0.001 M) under an atmosphere of nitrogen was added DEPBT (60 mg, 0.20 mmol, 2.0 equiv) and DIPEA (35 μL, 0.20 mmol, 2.0 equiv) and the mixture was stirred for 24 h at room temperature. The solvent was removed under reduced pressure, and the residue was purified twice by column chromatography (SiO_2, CH_2Cl_2/MeOH 9:1) to give cyclic pentapeptide **107** as a white solid (1st run: 20 mg, 41 μmol, 41%; 2nd run: 18 mg, 37 μmol, 37%). $\mathbf{R_f}$ (CH_2Cl_2/MeOH 9:1) 0.28; **mp** 288–290 ºC (decomposition); **^{1}H NMR** (500 MHz, CD_3OD) δ_H 7.05 (d, *J* = 8.4 Hz, 2H, ArH), 6.75 (d, *J* = 8.4 Hz, 2H, ArH), 4.41 (q, *J* = 7.3 Hz, 1H, CHα-Ala), 4.23 (q, *J* = 7.2 Hz, 1H, CHα-Ala), 4.16 (dd, *J* = 11.4, 3.9 Hz, 1H, CHα-Leu), 4.00 (dd, *J* = 10.2, 5.7 Hz, 1H, CHα-Tyr), 3.73 (dt, *J* = 13.3, 3.9 Hz, 1H, C*H*HNH), 3.28–3.09 (m, 3H, CH*H*NH, CH_2β-Tyr), 2.51 (ddd, *J* = 13.6, 11.7, 4.3 Hz, 1H, C*H*HCONH), 2.19 (dt, *J* = 13.6, 3.4 Hz, 1H, CH*H*CONH), 1.88 (ddd, *J* = 14.4, 11.7, 4.2 Hz, 1H, C*H*Hβ-Leu), 1.71 (ddd, *J* = 13.9, 10.2, 3.9 Hz, 1H, CH*H*β-Leu), 1.59–1.46 (m, 4H, CH_3β-Ala, CHγ-Leu), 1.27 (d, *J* = 7.2 Hz, 3H, CH_3β-Ala), 0.96 (d, *J* = 6.6 Hz, 3H, CH_3δ-Leu), 0.90 (d, *J* = 6.6 Hz, 3H, CH_3δ-Leu); **^{13}C NMR** (126 MHz, CD_3OD) δ_C 175.7 (C=O), 174.8 (C=O), 174.4 (C=O), 174.3 (2 × C=O), 157.4 (C), 131.3 (CH), 129.5 (C), 116.4 (CH), 58.0 (CH, α-Tyr), 55.0 (CH, α-Leu), 50.6 (CH, α-Ala), 50.4 (CH, α-Ala), 39.7 (CH_2, β-Leu), 37.6 (CH_2NH), 36.1 (*C*H_2CONH), 35.2 (CH_2, β-Tyr), 26.0 (CH, γ-Leu), 23.7 (CH_3, δ-Leu), 21.2 (CH_3, δ-Leu), 17.8 (CH_3, β-Ala), 17.1 (CH_3, β-Ala); $\mathbf{\nu_{max}}$ (neat) = 3281, 2956, 1642, 1513, 1237 cm^{-1}; **MS** (ESI^+) *m/z* 512 $[M+Na]^+$; **HRMS** (ESI^+) calcd. for $C_{24}H_{35}N_5NaO_6$ $[M+Na]^+$ 512.2480, found 512.2483; $[\alpha]_D^{29}$ –102 (*c* 0.05, MeOH).

*2.12 Preparation of cyclic pentapeptide **112***

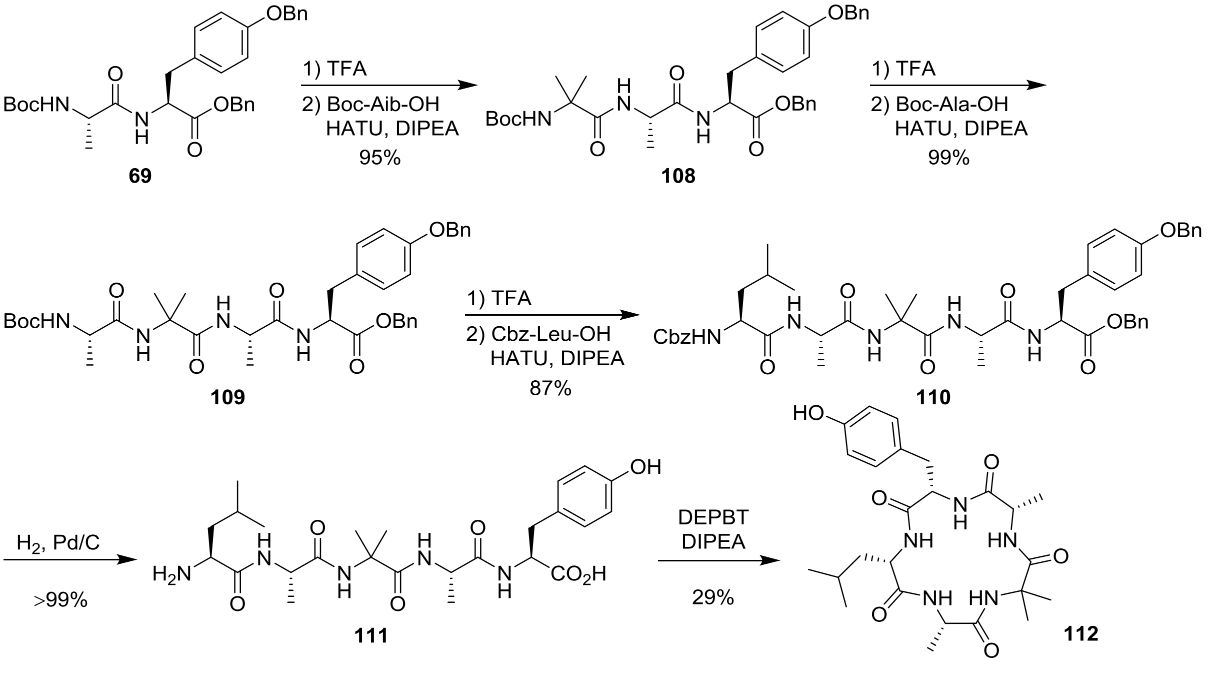

Boc-Aib-Ala-Tyr(Bn)-OBn (108)

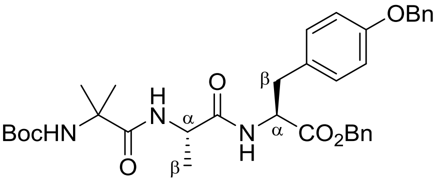

108

To a solution of Boc-Ala-Tyr(Bn)-OBn (**69**) (5.33 g, 10.0 mmol, 1.0 equiv) in CH_2Cl_2 (10 mL) was added TFA (10 mL) and the mixture was stirred at room temperature for 1 h (*Caution – gas evolution!*). The reaction mixture was concentrated *in vacuo* and the resulting residue repeatedly dissolved in CH_2Cl_2 (3 × 10 mL) and concentrated under reduced pressure to give the crude amine. The residue was dissolved in CH_2Cl_2 (100 mL), Boc-Aib-OH (2.44 g, 12.0 mmol, 1.2 equiv), HATU (4.56 g, 12.0 mmol, 1.2 equiv) and DIPEA (6.97 mL, 40.0 mmol, 4.0 equiv) were added subsequently, and the mixture was stirred at room temperature for 16 h. The reaction mixture was washed with 10% citric acid solution (2 × 100 mL) and saturated $NaHCO_3$ solution (2 × 100 mL), dried over $MgSO_4$, filtered and concentrated under reduced pressure. The residue was purified by column chromatography (SiO_2, PE/EtOAc 1:1→EtOAc) to give Boc-Aib-Ala-Tyr(Bn)-OBn (**108**) (5.90 g, 9.54 mmol, 95%) as a white foam. **R_f** (EtOAc) 0.64; **mp** 52–55 °C; **^{1}H NMR** (500 MHz, $CDCl_3$) δ_H ppm 7.43–7.26 (m, 10H, ArH), 6.96 (d, *J* = 8.3 Hz, 2H, ArH), 6.82 (d, *J* = 8.5 Hz, 2H, ArH), 6.73 (d, *J* = 7.3 Hz, 1H, NH), 5.14 (d, *J* = 12.2 Hz, 1H, C*H*HPh), 5.08 (d, *J* = 12.2 Hz, 1H, CH*H*Ph), 5.01 (s, 2H, CH_2Ph), 4.89 (br. s, 1H, NH), 4.79 (q, *J* = 6.4 Hz, 1H, CHα-Tyr), 4.42 (quin, *J* = 7.1 Hz, 1H, CHα-Ala), 3.08 (dd, *J* = 14.0, 6.2 Hz, 1H, C*H*Hβ-Tyr), 3.03 (dd, *J* = 14.0, 6.3 Hz, 1H, CH*H*β-Tyr), 1.70 (d, *J* = 10.0 Hz, 1H, NH), 1.46 (s, 3H, CH_3-Aib), 1.42 (s, 9H, 3 × CH_3, Boc), 1.41 (s, 3H, CH_3-Aib), 1.32 (d, *J* = 7.0 Hz, 3H, CH_3β-Ala); **^{13}C NMR** (126 MHz, $CDCl_3$) δ_C ppm 174.4 (C=O), 172.0 (C=O), 171.2 (C=O), 158.0 (C), 154.9 (C=O, Boc), 137.1 (C), 135.3 (C), 130.5 (CH), 128.73 (CH), 128.68 (CH), 128.6 (CH), 128.5 (CH), 128.3 (C), 128.1 (CH), 127.6 (CH), 115.0 (CH), 80.6 (C, Boc), 70.1 (CH_2, Bn), 67.2 (CH_2, Bn), 56.8 (C, Aib), 53.7 (CH, α-Tyr), 49.1 (CH, α-Ala), 37.0 (CH_2, β-Tyr), 28.4 (CH_3, Boc), 25.5 (CH_3, Aib), 18.1 (CH_3, β-Ala); **ν_{max}** (neat) = 3306, 2977, 1650, 1509, 1241, 1159, 1077, 1015, 735, 696 cm^{-1}; **MS** (ESI$^+$) *m/z* 640 $[M+Na]^+$; **HRMS** (ESI$^+$) calcd. for $C_{35}H_{43}N_3NaO_7$ $[M+Na]^+$ 640.2993, found 640.2986; $[\alpha]_D^{26}$ –6.0 (*c* 0.20, $CHCl_3$).

Boc-Ala-Aib-Ala-Tyr(Bn)-OBn (109)

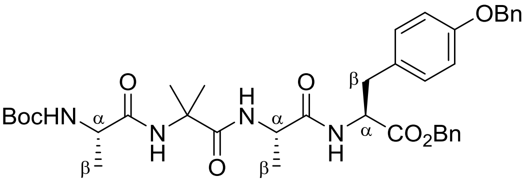

109

To a solution of Boc-Aib-Ala-Tyr(Bn)-OBn (**108**) (5.67 g, 9.18 mmol, 1.0 equiv) in CH_2Cl_2 (10 mL) was added TFA (10 mL) and the mixture was stirred at room temperature for 1 h (*Caution – gas evolution!*). The reaction mixture was concentrated under reduced pressure and the resulting residue repeatedly dissolved in CH_2Cl_2 (3 × 10 mL) and concentrated under reduced pressure to give the crude amine. The residue was dissolved in a mixture of CH_2Cl_2 (92 mL) and DMF (10 mL), Boc-Ala-OH (2.08 g, 11.0 mmol, 1.2 equiv), HATU (4.19 g, 11.0 mmol, 1.2 equiv) and DIPEA (6.39 mL, 36.7 mmol, 4.0 equiv) were added subsequently, and the mixture was stirred at room temperature for 16 h. The reaction mixture was washed with 10% citric acid solution (2 × 100 mL) and saturated $NaHCO_3$ solution (2 × 100 mL), dried over $MgSO_4$, filtered and concentrated under reduced pressure. The residue was purified by column chromatography (SiO_2, PE/EtOAc 1:1→EtOAc) to give Boc-Ala-Aib-Ala-Tyr(Bn)-OBn (**109**) (6.27 g, 9.10 mmol, 99%) as a white foam. **R_f** (EtOAc) 0.48; **mp** 51–54 °C; **^{1}H NMR** (500 MHz, $CDCl_3$) δ_H 7.54 (d, *J* = 7.5 Hz, 1H, NH), 7.42–7.34 (m, 5H, ArH), 7.33–7.27 (m, 5H, ArH), 7.11 (d, *J* = 8.3 Hz, 2H, ArH), 6.81 (d, *J* = 8.3 Hz, 3H, ArH, NH), 5.39 (br. s, 1H, NH), 5.14 (d, *J* = 12.4 Hz, 1H, C*H*HPh), 5.10 (d, *J* = 12.4 Hz, 1H, CH*H*Ph), 4.98 (s, 2H, CH_2Ph), 4.72 (dd, *J* = 14.2, 8.2 Hz, 1H, CHα-Tyr), 4.30 (quin, *J* = 7.2 Hz, 1H, CHα-Ala), 3.84 (td, *J* = 8.9, 5.4 Hz, 1H, CHα-Ala), 3.15 (dd, *J* = 14.0, 5.6 Hz, 1H, C*H*Hβ-Tyr), 3.07 (dd, *J* = 14.0, 8.8 Hz, 1H, CH*H*β-Tyr), 1.95 (br. s, 1 H, NH), 1.55 (s, 3H, CH_3-

Aib), 1.46 (s, 9H, 3 × CH_3, Boc), 1.43 (s, 3H, CH_3-Aib), 1.33 (d, *J* = 5.8 Hz, 3H, CH_3β-Ala), 1.31 (d, *J* = 6.7 Hz, 3H, CH_3β-Ala); **^{13}C NMR** (126 MHz, $CDCl_3$) δ_C ppm 174.7 (C=O), 173.9 (C=O), 172.9 (C=O), 172.5 (C=O), 157.8 (C), 156.8 (C=O, Boc), 137.2 (C), 135.5 (C), 130.5 (CH), 129.3 (C), 128.7 (CH), 128.6 (CH), 128.32 (CH), 128.31 (CH), 128.0 (CH), 127.5 (CH), 114.8 (CH), 81.2 (C, Boc), 70.0 (CH_2, Bn), 67.3 (CH_2, Bn), 56.8 (C, Aib), 54.1 (CH, α-Tyr), 52.7 (CH, α-Ala), 50.2 (CH, α-Ala), 36.7 (CH_2, β-Tyr), 28.3 (CH_3, Boc), 27.2 (CH_3, Aib), 23.8 (CH_3, Aib), 17.4 (CH_3, β-Ala), 16.9 (CH_3, β-Ala); **ν_{max}** (neat) = 3306, 2978, 1650, 1510, 1242, 1161, 838, 736, 696 cm^{-1}; **MS** (ESI^+) *m/z* 711 $[M+Na]^+$; **HRMS** (ESI^+) calcd. for $C_{38}H_{48}N_4NaO_8$ $[M+Na]^+$ 711.3364, found 711.3366; $[\alpha]_D^{26}$ +6.0 (*c* 1.00, $CHCl_3$).

Cbz-Leu-Ala-Aib-Ala-Tyr(Bn)-OBn (110)

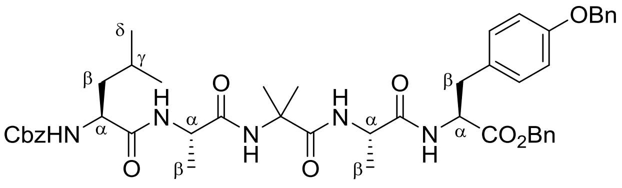

110

To a solution of Boc-Ala-Aib-Ala-Tyr(Bn)-OBn (**109**) (689 mg, 1.00 mmol, 1.0 equiv) in CH_2Cl_2 (5.0 mL) was added TFA (5.0 mL) and the mixture was stirred at room temperature for 1 h (*Caution – gas evolution!*). The reaction mixture was concentrated under reduced pressure and the resulting residue repeatedly dissolved in CH_2Cl_2 (3 × 10 mL) and concentrated under reduced pressure to give the crude amine. The residue was dissolved in a mixture of CH_2Cl_2 (8.0 mL) and DMF (2.0 mL), Cbz-Leu-OH (531 mg, 2.00 mmol, 2.0 equiv), HATU (760 mg, 2.00 mmol, 2.0 equiv) and DIPEA (1.05 mL, 6.00 mmol, 6.0 equiv) were added subsequently, and the mixture was stirred at room temperature for 16 h. The reaction mixture was diluted with CH_2Cl_2 (20 mL) washed with 10% citric acid solution (2 × 20 ml) and saturated $NaHCO_3$ solution (2 × 20 ml), dried over $MgSO_4$, filtered and concentrated under reduced pressure. The residue was purified by column chromatography (SiO_2, PE/EtOAc 1:1→EtOAc) to give Cbz-Leu-Ala-Aib-Ala-Tyr(Bn)-OBn (**110**) (727 mg, 0.87 mmol, 87%) as a white foam. **R_f** (EtOAc) 0.38; **mp** 65–66 °C; **^{1}H NMR** (500 MHz, $CDCl_3$) δ_H ppm 7.44–7.26 (m, 16H, ArH, NH), 7.07 (d, *J* = 8.4 Hz, 2H, ArH), 6.96 (d, *J* = 7.4 Hz, 1H, NH), 6.89 (s, 1H, NH), 6.80 (d, *J* = 8.4 Hz, 2H, ArH), 6.69 (d, *J* = 5.1 Hz, 1H, NH), 5.38 (d, *J* = 4.2 Hz, 1H, NH), 5.15–5.05 (m, 4H, 2 × CH_2Ph), 4.98 (s, 2H, CH_2Ph), 4.76 (q, *J* = 7.4 Hz, 1H, CHα-Tyr), 4.40 (quin, *J* = 7.2 Hz, 1H, CHα-Ala), 4.11–4.03 (m, 2H, CHα-Ala, CHα-Leu), 3.13 (dd, *J* = 13.9, 6.2 Hz, 1H, C*H*Hβ-Tyr), 3.03 (dd, *J* = 13.9, 8.0 Hz, 1H, CH*H*β-Tyr), 1.75–1.61 (m, 2H, C*H*Hβ-Leu, CHγ-Leu), 1.51 (s, 3H, CH_3-Aib), 1.50–1.48 (m, 1H, CH*H*β-Leu), 1.47 (s, 3H, CH_3-Aib), 1.32 (d, *J* = 7.2 Hz, 3H, CH_3β-Ala), 1.26 (d, *J* = 7.2 Hz, 3H, CH_3β-Ala), 0.95 (d, *J* = 6.4 Hz, 3H, CH_3δ-Leu), 0.93 (d, *J* = 6.4 Hz, 3H, CH_3δ-Leu); **^{13}C NMR** (126 MHz, $CDCl_3$) δ_C ppm 173.9 (C=O), 173.4 (C=O), 172.7 (C=O), 172.4 (C=O), 171.7 (C=O), 157.7 (C), 156.9 (C=O, Cbz), 137.2 (C), 136.1 (C), 135.7 (C), 130.6 (CH), 129.3 (C), 128.8 (CH), 128.70 (CH), 128.65 (CH), 128.6 (CH), 128.31 (CH), 128.27 (CH), 128.2 (CH), 128.1 (CH), 127.6 (CH), 114.8 (CH), 70.1 (CH_2, Bn), 67.7 (CH_2, Bn), 67.0 (CH_2, Bn), 57.2 (C, Aib), 54.8 (CH, α-Leu), 54.0 (CH, α-Tyr), 50.9 (CH, α-Ala), 49.7 (CH, α-Ala), 40.7 (CH_2, β-Leu), 37.0 (CH_2, β-Tyr), 26.2 (CH_3, Aib), 25.0 (CH, γ-Leu), 24.7 (CH_3, Aib), 22.9 (CH_3, δ-Leu), 21.9 (CH_3, δ-Leu), 17.5 (CH_3, β-Ala), 16.9 (CH_3, β-Ala); **ν_{max}** (neat) = 3303, 2978, 1651, 1510, 1242, 1161, 839, 736, 696 cm^{-1}; **MS** (ESI^+) *m/z* 858 $[M+Na]^+$; **HRMS** (ESI^+) calcd. for $C_{47}H_{57}N_5NaO_9$ $[M+Na]^+$ 858.4048, found 858.4053; $[\alpha]_D^{27}$ –7.3 (*c* 0.10, $CHCl_3$).

H-Leu-Ala-Aib-Ala-Tyr-OH (111)

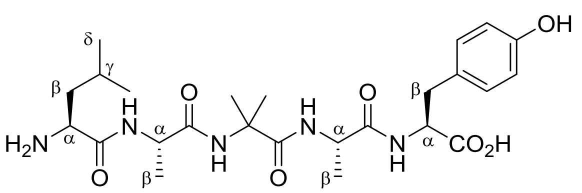

111

To a solution of pentapeptide **110** (445 mg, 0.53 mmol, 1.0 equiv) in anhydrous MeOH (6.0 mL) was added 10 wt% Pd/C (45 mg, 10 wt%) and the reaction flask was evacuated, filled with nitrogen, evacuated, and placed under an atmosphere of hydrogen (balloon). The mixture was stirred at room temperature for 16 h, placed under nitrogen and filtered through a plug of Celite, which was washed with MeOH (3×). The filtrate was concentrated *in vacuo* to give **111** as an off-white solid (276 mg, 0.53 mmol) in quantitative yield. **mp** 198–202 °C; **^{1}H NMR** (500 MHz, CD_3OD) δ_H ppm 7.05 (d, *J* = 8.2 Hz, 2H, ArH), 6.69 (d, *J* = 8.2 Hz, 2H, ArH), 4.51 (t, *J* = 6.7 Hz, 1H, CHα-Tyr), 4.33 (q, *J* = 6.9 Hz, 1H, CHα-Ala), 4.26 (q, *J* = 6.9 Hz, 1H, CHα-Ala), 3.97–3.89 (m, 1H, CHα-Leu), 3.10 (dd, *J* = 13.8, 5.1 Hz, 1H, C*H*Hβ-Tyr), 2.94 (dd, *J* = 13.8, 8.1 Hz, 1H, CH*H*β-Tyr), 1.81–1.69 (m, 2H, C*H*Hβ-Leu, CHγ-Leu), 1.67–1.59 (m, 1H, CH*H*β-Leu), 1.45 (s, 3H, CH_3-Aib), 1.41 (s, 3H, CH_3-Aib), 1.37 (d, *J* = 7.0 Hz, 3H, CH_3β-Ala), 1.29 (d, *J* = 7.0 Hz, 3H, CH_3β-Ala), 1.00 (d, *J* = 5.9 Hz, 1H, CH_3δ-Leu), 0.98 (d, *J* = 5.9 Hz, 1H, CH_3δ-Leu); **^{13}C NMR** (126 MHz, CD_3OD) δ_C ppm 176.5 (C=O), 176.4 (C=O), 174.7 (C=O), 174.0 (C=O), 170.7 (C=O), 157.2 (C), 131.4 (CH), 129.1 (C), 116.2 (CH), 57.8 (C, Aib), 55.8 (CH, α-Tyr), 52.8 (CH, α-Leu), 51.3 (CH, α-Ala), 50.6 (CH, α-Ala), 41.6 (CH_2, β-Leu), 37.6 (CH_2, β-Tyr), 26.4 (CH_3, Aib), 25.3 (CH, γ-Leu), 24.5 (CH_3, Aib), 23.0 (CH_3, δ-Leu), 22.2 (CH_3, δ-Leu), 18.1 (CH_3, β-Ala), 17.3 (CH_3, β-Ala); **ν_{max}** (neat) = 3278, 2958, 1651, 1514, 1216, 1173 cm^{-1}; **MS** (ESI^+) *m/z* 522 $[M+H]^+$, 544 $[M+Na]^+$; **HRMS** (ESI^+) calcd. for $C_{25}H_{40}N_5O_7$ $[M+H]^+$ 522.2922, found 522.2923; $[\alpha]_D^{27}$ +4.5 (*c* 0.20, MeOH).

Cyclo(Leu-Ala-Aib-Ala-Tyr) (112)

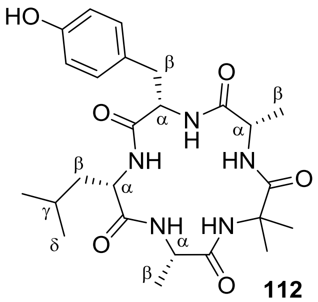

112

To a solution of H-Leu-Ala-Aib-Ala-Tyr-OH (**111**) (52 mg, 0.10 mmol, 1.0 equiv) in anhydrous DMF (100 mL, 0.001 M) under an atmosphere of nitrogen was added DEPBT (60 mg, 0.20 mmol, 2.0 equiv) and DIPEA (35 µL, 0.20 mmol, 2.0 equiv) and the reaction mixture was stirred for 24 h at room temperature. The DMF was removed under reduced pressure at 60 °C over 30 min, and the residue was dried under reduced pressure. The residue was purified twice by column chromatography (SiO_2, DCM/MeOH 9:1→4:1) to give the cyclic pentapeptide (**112**) as an off-white solid (1st run: 15 mg, 30 µmol, 30%; 2nd run: 14 mg, 28 µmol, 28%). **R_f** (CH_2Cl_2/MeOH 4:1) 0.62; **mp** 262–264 °C (decomposition); **^{1}H NMR** (400 MHz, CD_3OD) δ_H ppm 7.09 (d, *J* = 8.4 Hz, 2H, ArH), 6.71 (d, *J* = 8.4 Hz, 2H, ArH), 4.54 (t, *J* = 8.0 Hz, 1H, CHα-Tyr), 4.42 (q, *J* = 7.0 Hz, 1H, CHα-Ala), 4.18 (q, *J* = 7.4 Hz, 1H, CHα-Ala), 3.84 (dd, *J* = 10.7, 5.5 Hz, 1H, CHα-Leu), 3.04 (dd, *J* = 13.6, 8.3 Hz, 1H, C*H*Hβ-Tyr), 3.00 (dd, *J* = 13.6, 7.8 Hz, 1H, CH*H*β-Tyr), 1.96 (ddd, *J* = 13.8, 10.7, 4.7 Hz, 1H, C*H*Hβ-Leu), 1.62 (s, 3H, CH_3-Aib), 1.50–1.46 (m, 2H, CH*H*β-Leu, CHγ-Leu), 1.36–1.30 (m, 9H, CH_3-Aib, 2 × CH_3β-Ala), 0.88 (d, *J* = 6.6 Hz, 3H, CH_3δ-Leu), 0.77 (d, *J* = 6.6 Hz, 3H, CH_3δ-Leu); **^{13}C NMR** (126 MHz, CD_3OD) δ_C ppm 176.9 (C=O), 175.7 (C=O), 175.1 (C=O), 174.7 (C=O), 173.3 (C=O), 157.3 (C), 131.3 (CH), 128.9 (C), 116.2 (CH), 57.9 (CH, α-Leu), 57.8 (C, Aib), 57.0 (CH, α-Tyr), 52.2 (CH, α-Ala), 49.6 (CH, α-Ala), 39.6 (CH_2, β-Leu), 36.7 (CH_2, β-Tyr), 26.1 (CH_3, Aib), 25.8 (CH, γ-Leu), 23.31 (CH_3, Aib), 23.25 (CH_3, δ-Leu), 21.4 (CH_3, δ-Leu), 18.9 (CH_3, β-Ala), 17.5 (CH_3, β-Ala); **ν_{max}** (neat) = 3288, 2934, 1654, 1515, 1458, 1060, 826 cm^{-1}; **MS** (ESI^+) *m/z* 526 $[M+Na]^+$; **HRMS** (ESI^+) calcd. for $C_{25}H_{37}N_5NaO_6$ $[M+Na]^+$ 526.2636, found 526.2637; $[\alpha]_D^{27}$ –76.7 (*c* 0.27, MeOH).

*Preparation of cyclic pentapeptides **92**, **98**, **102**, **107** and **112** (see Scheme 4 in paper)*

Linear pentapeptide	Cyclic peptide	1st run	2nd run	Average yield
H-Leu-Ala-Sar-Ala-Tyr-OH (**91**)	**92**	39%	33%	**36%**
H-Leu-Ala-$NHCH_2CH_2$-Ala-Tyr-OH (**97**)	**98**	28%	n.d.	**28%**
H-Leu-Ala-$NHCH_2C(Me)_2$-Ala-Tyr-OH (**101**)	**102**	33%	31%	**32%**
H-Leu-Ala-βAla-Ala-Tyr-OH (**106**)	**107**	37%	41%	**39%**
H-Leu-Ala-Aib-Ala-Tyr-OH (**111**)	**112**	30%	28%	**29%**

*2.13 Preparation of cyclic hexapeptide **14***

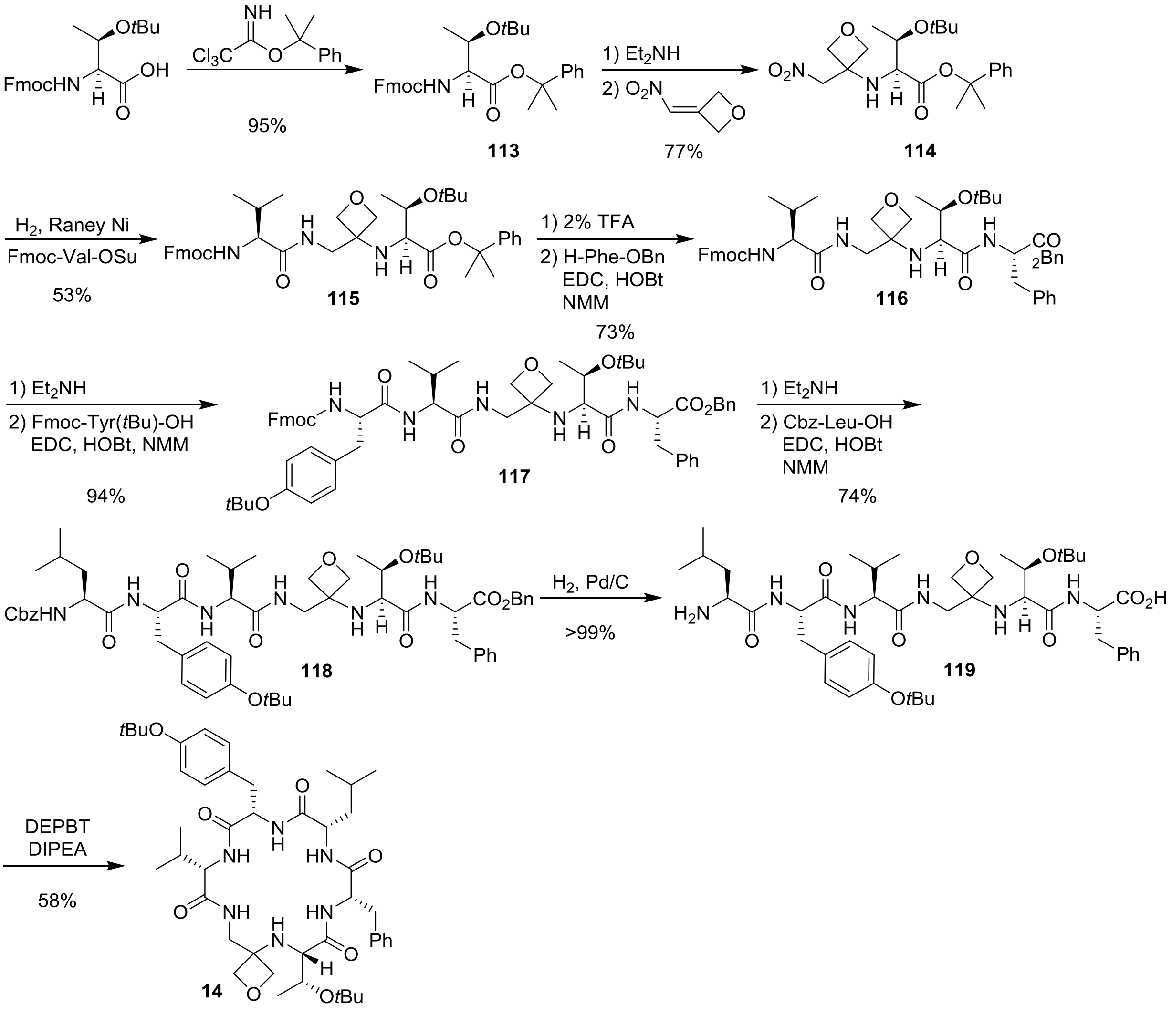

Fmoc-Thr(*t*Bu)-OCumyl (113)

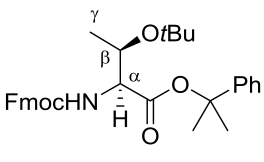

113

To sodium hydride (60% dispersion in mineral oil, 84 mg, 2.50 mmol, 0.5 equiv) in Et_2O (5.0 mL) at 0 °C was added freshly distilled 2-phenyl-2-propanol (1.50 g, 11.0 mmol, 2.2 equiv) in Et_2O (5.5 mL) and stirred at room temperature for 1 h. The mixture was cooled to 0 °C, trichloroacetonitrile (1.44 mL, 10.0 mmol, 2.0 equiv) was added, the reaction mixture stirred at room temperature for 3 h and then concentrated *in vacuo*. Petroleum ether (2.25 mL) and MeOH (100 μL, 0.5 equiv) were added and stirred at room temperature for 10 min. The

mixture was filtered through a plug of Celite eluting with PE. The eluent was concentrated under reduced pressure to give the crude imidate. To the crude imidate in CH_2Cl_2 (10 mL) was added Fmoc-Thr(*t*Bu)-OH (1.99 g, 5.00 mmol, 1.0 equiv) and the reaction mixture stirred at room temperature for 16 h. The mixture was filtered through a plug of Celite, the crude product was concentrated *in vacuo* and purified by column chromatography (PE/EtOAc 4:1) to yield **113** as a waxy solid (2.45 g, 4.75 mmol, 95%). $\mathbf{R_f}$ (PE/EtOAc 4:1) 0.28; **^{1}H NMR** (500 MHz, $CDCl_3$) δ_H 7.77 (d, *J* = 7.5 Hz, 2H, ArH), 7.63 (d, *J* = 7.1 Hz, 2H, ArH), 7.45–7.38 (m, 4H, ArH), 7.36–7.27 (m, 4H, ArH), 7.28–7.22 (m, 1H, ArH), 5.56 (d, *J* = 9.2 Hz, 1H, NH), 4.45–4.36 (m, 2H, CH_2-Fmoc), 4.29–4.22 (m, 3H, CH-Fmoc, CHα-Thr, CHβ-Thr), 1.82 (d, *J* = 4.4 Hz, 6H, 2 × CH_3, cumyl), 1.24–1.17 (m, 12H, CH_3γ-Thr, 3 × CH_3, *t*Bu); **^{13}C NMR** (126 MHz, $CDCl_3$) δ_C 169.9 (C=O), 156.7 (C=O, Fmoc), 145.5 (C), 144.2 (C), 144.0 (C), 141.4 (C), 128.4 (CH), 127.8 (CH), 127.29 (CH), 127.20 (CH), 127.18 (CH), 125.3 (CH), 124.7 (CH), 120.1 (CH), 83.4 (C, cumyl), 74.2 (C, *t*Bu), 67.4 (CH, α-Thr), 67.2 (CH_2, Fmoc), 60.4 (CH, β-Thr), 47.4 (CH, Fmoc), 28.9 (CH_3, *t*Bu), 28.7 (CH_3, cumyl), 28.4 (CH_3, cumyl), 20.6 (CH_3, γ-Thr). *N.B.* One aromatic C and one aromatic CH signal not observed. $\mathbf{\nu_{max}}$ (neat) = 2975, 1720, 1497, 1196, 1031, 738 cm^{-1}; **MS** (ESI^+) *m/z* 516 $[M+H]^+$, 538 $[M+Na]^+$; **HRMS** (ESI^+) calcd. for $C_{32}H_{37}NNaO_5$ $[M+Na]^+$ 538.2564, found 538.2569; $[\alpha]_D^{27}$ +20.0 (*c* 0.10, $CHCl_3$).

NO_2-GOx-Thr(*t*Bu)-OCumyl (114)

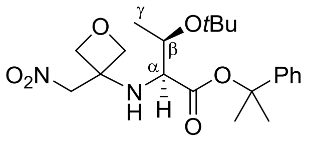

114

To a solution of Fmoc-Thr(*t*Bu)-OCumyl (**113**) (2.22 g, 4.30 mmol, 1.0 equiv) in CH_2Cl_2 (4.3 mL) was added diethylamine (4.3 mL) and the mixture was stirred at room temperature for 1 h. The reaction mixture was concentrated under reduced pressure and the resulting residue repeatedly dissolved in CH_2Cl_2 (3 × 50 mL) and concentrated under reduced pressure to give the crude amine. In a second reaction vessel, oxetane-3-one (503 µL, 8.60 mmol, 2.0 equiv), nitromethane (646 µL, 12.1 mmol, 2.8 equiv) and triethylamine (234 µL, 1.72 mmol, 0.4 equiv) were combined at 0 °C and stirred for 1 h at room temperature. The mixture was dissolved in anhydrous CH_2Cl_2 (34 mL), cooled to –78 °C, and triethylamine (2.34 mL, 17.2 mmol, 4.0 equiv) was added followed by dropwise addition of methanesulfonyl chloride (660 µL, 8.60 mmol, 2.0 equiv). The reaction mixture was stirred at –78 °C for 1.5 h and a solution of the crude amine in anhydrous CH_2Cl_2 (10 mL) was added slowly *via* syringe. The reaction mixture was allowed to warm to room temperature and stirred for 16 h. A saturated solution of NH_4Cl (100 mL) was added and stirred for 10 min. The layers were separated and the aqueous one extracted with CH_2Cl_2 (2 × 30 mL) and EtOAc (2 × 30 mL). The combined organic phases were washed saturated aqueous $NaHCO_3$ solution (50 mL), brine (50 mL), dried over Na_2SO_4, filtered and concentrated under reduced pressure. The residue was purified by column chromatography (SiO_2, PE/EtOAc 4:1) to yield **114** (1.35 g, 3.31 mmol, 77%) as a highly viscous yellow oil. $\mathbf{R_f}$ (PE/EtOAc 4:1) 0.20; **^{1}H NMR** (500 MHz, $CDCl_3$) δ_H 7.47 (d, *J* = 7.7 Hz, 1H, ArH), 7.36 (t, *J* = 7.7 Hz, 1H, ArH), 7.31–7.25 (m, 3H, ArH), 4.77 (s, 2H, CH_2GOx), 4.60–4.55 (m, 2H, OCH_2-Ox), 4.51 (d, *J* = 7.2 Hz, 1H, OC*H*H-Ox), 4.32 (d, *J* = 7.2 Hz, 1H, OCH*H*-Ox), 3.89 (quint, *J* = 6.2 Hz, 1H, CHβ-Thr), 3.36 (dd, *J* = 9.8, 4.6 Hz, 1H, CHα-Thr), 2.50 (d, *J* = 9.9 Hz, 1H, NH), 1.86 (s, 3H, CH_3, cumyl), 1.80 (s, 3H, CH_3, cumyl), 1.22 (s, 9H, 3 × CH_3, *t*Bu), 1.13 (d, *J* = 6.2 Hz, 3H, CH_3γ-Thr); **^{13}C NMR** (126 MHz, $CDCl_3$) δ_C 172.6 (C=O), 145.0 (C), 128.2 (CH), 127.4 (CH), 124.7 (CH), 83.0 (C, cumyl), 79.0 (OCH_2), 78.9 (OCH_2), 78.6 (CH_2, GOx), 74.0 (C, *t*Bu), 68.9 (CH, α-Thr), 61.5 (CH, β-Thr), 59.5 (C, Ox), 28.8 (CH_3, cumyl), 28.5 (CH_3, *t*Bu), 27.6 (CH_3, cumyl), 19.4 (CH_3, γ-Thr); $\mathbf{\nu_{max}}$ (neat) = 2975, 2934, 1724, 1554, 1271, 1194, 980, 763 cm^{-1}; **MS** (ESI^+) *m/z* 431 $[M+Na]^+$; **HRMS** (ESI^+) calcd. for $C_{21}H_{32}N_2NaO_6$ $[M+Na]^+$ 431.2153, found 431.2144; $[\alpha]_D^{27}$ +33.0 (*c* 0.10, $CHCl_3$).

Fmoc-Val-GOx-Thr(*t*Bu)-OCumyl (115)

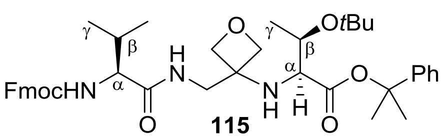

To a solution of NO_2-GOx-Thr(*t*Bu)-OCumyl (**114**) (1.24 g, 3.00 mmol, 1.0 equiv) in THF (136 mL) was added Fmoc-Val-OSu (1.96 g, 4.5 mmol, 1.5 equiv) and Raney Ni (slurry in H_2O, 5.0 mL). The solution was placed under an atmosphere of nitrogen, evacuated and filled with hydrogen (balloon). The reaction mixture was stirred vigorously for 4.0 h at room temperature. Then, the mixture was filtered through a plug of Celite eluting with EtOAc, concentrated *in vacuo*, the filtrate was diluted with EtOAc (50 mL), washed with saturated Na_2CO_3 (3 × 500 mL), dried over Na_2SO_4 and concentrated under reduced pressure. Fmoc-Val-GOx-Thr(*t*Bu)-OCumyl (**115**) was afforded after purification by column chromatography (SiO_2, CH_2Cl_2/EtOAc 4:1) as a white solid (1.11 g, 1.59 mmol, 53%); **mp** 74–76 °C; **R$_f$** (CH_2Cl_2/EtOAc 4:1) 0.34; **^{1}H NMR** (500 MHz, $CDCl_3$) δ_H 7.76 (d, *J* = 7.5 Hz, 2H, ArH), 7.59 (d, *J* = 7.4 Hz, 2H, ArH), 7.40 (m, 4H, ArH), 7.31 (m, 4H, ArH), 7.23 (m, 1H, ArH), 6.54–6.43 (t, *J* = 4.8 Hz, 1H, NH), 5.47 (d, *J* = 8.7 Hz, 1H, NH), 4.42 (dd, *J* = 10.3, 7.4 Hz, 1H, C*H*H-Fmoc), 4.36–4.28 (m, 3H, OCH_2-Ox, CH*H*-Fmoc), 4.20 (m, 3H, OCH_2-Ox, CH-Fmoc), 4.00–3.95 (dd, *J* = 7.9, 6.4 Hz, 1H, CHα-Val), 3.94–3.88 (m, 1H, CHα-Thr), 3.80 (dd, *J* = 13.8, 5.8 Hz, 1H, C*H*HGOx), 3.32 (dd, *J* = 13.8, 4.1 Hz, 1H, CH*H*GOx), 3.19 (d, *J* = 4.0 Hz, 1H, CHβ-Thr), 2.00 (m, 1H, CHβ-Val), 1.84 (s, 3H, CH_3, cumyl), 1.74 (s, 3H, CH_3, cumyl), 1.22–1.16 (m, 12H, CH_3γ-Thr, 3 × CH_3, *t*Bu), 0.90 (d, *J* = 6.6 Hz, 3H, CH_3γ-Val), 0.86 (d, *J* = 6.6 Hz, 3H, CH_3γ-Val); **^{13}C NMR** (126 MHz, $CDCl_3$) δ_C 173.9 (C=O), 171.8 (C=O), 156.4 (C=O, Fmoc), 145.0 (C), 144.1 (C), 144.0 (C), 141.4 (C), 128.4 (CH), 127.8 (CH), 127.6 (CH), 127.2 (CH), 125.3 (CH), 125.2 (CH), 124.8 (CH), 120.13 (CH), 120.11 (CH), 83.2 (C, cumyl), 80.5 (OCH_2), 79.4 (OCH_2), 74.2 (C, *t*Bu), 68.9 (CH, α-Thr), 67.1 (CH_2, Fmoc), 61.9 (CH, β-Thr), 60.4 (CH, α-Val), 59.2 (C, Ox), 47.3 (CH, Fmoc), 44.1 (CH_2, GOx), 31.5 (CH, β-Val), 29.4 (CH_3, cumyl), 28.8 (CH_3, *t*Bu), 27.2 (CH_3, cumyl), 20.4 (CH_3, γ-Thr), 19.3 (CH_3, γ-Val), 17.9 (CH_3, γ-Val). *N.B.* One aromatic C and two aromatic CH signals not observed; **ν_{max}** (neat) = 2971, 2873, 1721, 1658, 1466, 1237, 759 cm^{-1}; **MS** (ESI$^+$) *m/z* 700 [M+H]$^+$, 722 [M+Na]$^+$; **HRMS** (ESI$^+$) calcd. for $C_{41}H_{53}N_3NaO_7$ [M+Na]$^+$ 722.3776, found 722.3779; $[\alpha]_D^{27}$ +25.0 (*c* 0.10, $CHCl_3$).

Fmoc-Val-GOx-Thr(*t*Bu)-Phe-OBn (116)

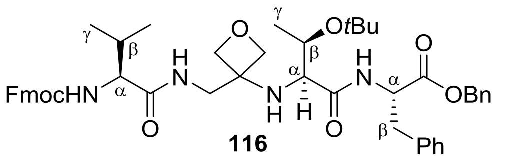

Compound **116** was prepared following a modified procedure from Beadle *et al.*[8] Fmoc-Val-GOx-Thr(*t*Bu)-OCumyl (**115**) (2.40 g, 3.40 mmol, 1.0 equiv) was dissolved in 2% TFA/CH_2Cl_2 and stirred at room temperature for 85 min. The mixture was concentrated under reduced pressure, the resulting residue was repeatedly re-dissolved in CH_2Cl_2 (50 mL) and the solvent was removed under reduced pressure. To the crude acid in CH_2Cl_2 (34 mL) was added H-Phe-OBn·HCl (1.10 g, 3.70 mmol, 1.1 equiv), NMM (1.51 mL, 13.7 mmol, 5.0 equiv), HOBt·H_2O (0.51 g, 3.70 mmol, 1.1 equiv) and EDC·HCl (0.72 g, 3.70 mmol, 1.1 equiv). The reaction mixture was allowed to stir for 18 h at room temperature under an atmosphere of nitrogen. The mixture was diluted with EtOAc (250 mL) and washed with brine (3 × 150 mL), dried (Na_2SO_4) and concentrated *in vacuo* to afford a yellow oil which was purified by flash column chromatography (CH_2Cl_2/MeOH 49:1). Fmoc-Val-GOx-Thr(*t*Bu)-Phe-OBn (**116**) was obtained as a white solid in 73% yield (2.03 g, 2.48 mmol). **R$_f$** (CH_2Cl_2/MeOH 49:1) 0.31; **mp** 68–70 ºC; **^{1}H NMR** (500 MHz, $CDCl_3$) δ_H 7.81–7.74 (m, 3H, NH, 2 × ArH), 7.63–7.56 (m, 2H, ArH), 7.43–7.27 (m, 10H, ArH), 7.24–7.17 (m, 2H, ArH), 7.11–7.01 (m, 3H, NH, 2 × ArH,), 5.51 (d, *J* = 8.9 Hz, 1H, NH), 5.22–5.09 (m, 2H, CH_2Ph), 4.91 (dt, *J* = 8.1, 5.9 Hz, 1H, CHα-Phe), 4.47–4.38 (m, 2H, OC*H*H-Ox, C*H*H-Fmoc), 4.36–4.20 (m, 5H, CH*H*-Fmoc, CH-Fmoc, OCH*H*-Ox, OCH_2-Ox), 4.08–4.02 (m, 1H, CHα-Val), 3.92 (dd, *J* = 13.9, 7.3 Hz, 1H C*H*HGOx), 3.57–

3.50 (m, 1H, CHα-Thr), 3.21–3.07 (m, 2H, CH*H*GOx, C*H*Hβ-Phe), 3.05–2.94 (m, 2H, CHβ-Thr, CH*H*β-Phe), 2.41 (br. s, 1H, NH), 2.11 (dq, *J* = 13.2, 6.4 Hz, 1H, CHβ-Val), 1.08 (s, 9H, 3 × CH_3, *t*Bu), 1.00–0.96 (m, 6H, CH_3γ-Thr, CH_3γ-Val), 0.93 (d, *J* = 6.6 Hz, 3H, CH_3γ-Val); **^{13}C NMR** (126 MHz, $CDCl_3$) δ_C 173.3 (C=O), 172.3 (C=O), 172.1 (C=O), 156.5 (C=O, Fmoc), 144.0 (C), 141.4 (C), 136.2 (C), 135.1 (C), 129.3 (CH), 128.8 (CH), 128.73 (CH), 128.70 (CH), 128.6 (CH), 127.9 (CH), 127.24 (CH), 127.23 (CH), 125.3 (CH), 125.2 (CH), 120.2 (CH), 120.1 (CH), 80.1 (OCH_2), 79.0 (OCH_2), 74.8 (C, *t*Bu), 69.8 (CH, α-Thr), 67.6 (CH_2, Bn), 67.2 (CH_2, Fmoc), 61.8 (CH, β-Thr), 60.6 (CH, α-Val), 60.3 (C, Ox), 53.3 (CH, α-Phe), 47.3 (CH, Fmoc), 44.1 (CH_2, GOx), 38.2 (CH_2, β-Phe), 31.2 (CH, β-Val), 28.4 (CH_3, *t*Bu), 19.5 (CH_3, γ-Val), 18.7 (CH_3, γ-Thr), 18.1 (CH_3, γ-Val). *N.B.* Two aromatic C and two aromatic CH signals not observed; **ν_{max}** (neat) = 2968, 1722, 1659, 1514, 1449, 1187, 1076, 738 cm^{-1}; **MS** (ESI^+) *m/z* 819 $[M+H]^+$, 841 $[M+Na]^+$; **HRMS** (ESI^+) calcd. for $C_{48}H_{58}N_4NaO_8$ $[M+Na]^+$ 841.4147, found 841.4134; $[\alpha]_D^{27}$ +21.5 (*c* 0.10, $CHCl_3$).

Fmoc-Tyr(*t*Bu)-Val-GOx-Thr(*t*Bu)-Phe-OBn (117)

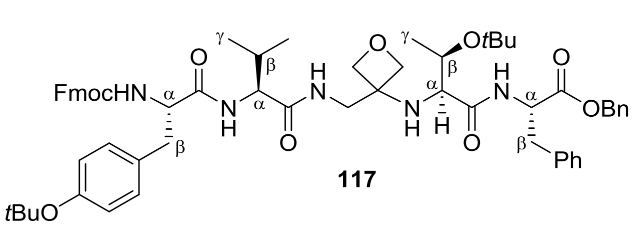

To a solution of tetrapeptide Fmoc-Val-GOx-Thr(*t*Bu)-Phe-OBn (**116**) (1.40 g, 1.71 mmol, 1.0 equiv) in CH_2Cl_2 (2.0 mL) was added diethylamine (2.0 mL) and the mixture was stirred at room temperature for 1 h. The reaction mixture was concentrated under reduced pressure and the resulting residue repeatedly dissolved in CH_2Cl_2 (3 × 20 mL) and concentrated under reduced pressure to give the crude amine. The residue was dissolved in CH_2Cl_2 (20 mL), Fmoc-Tyr(*t*Bu)-OH (0.86 g, 1.81 mmol, 1.1 equiv), EDC·HCl (0.35 g, 1.81 mmol, 1.1 equiv), HOBt·H_2O (0.24 g, 1.81 mmol, 1.1 equiv) and NMM (0.79 mL, 7.24 mmol, 4.0 equiv) were added subsequently, and the mixture was stirred at room temperature for 24 h. The reaction mixture was diluted with EtOAc (50 mL) and washed with brine (3 × 50 mL), dried over Na_2SO_4, filtered and concentrated under reduced pressure. The residue was purified by column chromatography (SiO_2, CH_2Cl_2/MeOH 49:1) to give pentapeptide Fmoc-Tyr(*t*Bu)-Val-GOx-Thr(*t*Bu)-Phe-OBn (**117**) (1.65 g, 1.59 mmol, 94%) as a white solid. **R_f** (CH_2Cl_2/MeOH 49:1) 0.23; **mp** 89–91 ºC; **^{1}H NMR** (400 MHz, $CDCl_3$) δ_H 7.80 (d, *J* = 8.1 Hz, 1H, NH), 7.76 (d, *J* = 7.5 Hz, 2H, ArH), 7.56–7.49 (m, 2H, ArH), 7.44–7.20 (m, 12H, ArH), 7.13–6.96 (m, 5H, 4 × ArH, NH), 6.90 (d, *J* = 8.2 Hz, 2H, ArH), 6.55 (d, *J* = 7.8 Hz, 1H, NH), 5.29 (d, *J* = 6.5 Hz, 1H, NH), 5.22–5.08 (m, 2H, CH_2Ph), 4.97–4.86 (m, 1H, CHα-Phe), 4.49–4.18 (m, 9H, CH-Fmoc, CH_2-Fmoc, 2 × OCH_2-Ox, CHα-Tyr, CHα-Val), 3.90–3.79 (m, 1H, C*H*HGOx), 3.67–3.58 (m, 1H, CHα-Thr), 3.22–2.98 (m, 6H, CH*H*GOx, CHβ-Thr, CH_2β-Tyr, CH_2β-Phe), 2.47 (s, 1H, NH), 2.15–2.02 (m, 1H, CHβ-Val), 1.31 (s, 9H, 3 × CH_3, *t*Bu), 1.13 (s, 9H, 3 × CH_3, *t*Bu), 1.02 (d, *J* = 6.2 Hz, 3H, CH_3γ-Thr), 0.89 (d, *J* = 6.7 Hz, 3H, CH_3γ-Val), 0.81 (d, *J* = 6.7 Hz, 3H, CH_3γ-Val); **^{13}C NMR** (126 MHz, $CDCl_3$) δ_C 173.3 (C=O), 172.0 (C=O), 171.5 (C=O), 171.1 (C=O), 156.1 (C=O, Fmoc), 154.6 (C), 143.9 (C), 143.8 (C), 141.4 (C), 136.1 (C), 135.1 (C), 129.3 (CH), 128.78 (CH), 128.76 (CH), 128.7 (CH), 128.6 (CH), 127.9 (CH), 127.3 (CH), 127.2 (CH), 125.20 (CH), 125.15 (CH), 124.5 (CH), 123.0 (CH), 120.1 (CH), 79.9 (OCH_2), 79.0 (OCH_2), 78.6 (C, *t*Bu), 74.9 (C, *t*Bu), 69.8 (CH, α-Thr), 67.5 (CH_2, Bn), 67.2 (CH_2, Fmoc), 61.6 (CH, β-Thr), 60.2 (C, Ox), 58.9 (CH, α-Val), 56.3 (CH, α-Tyr), 53.4 (CH, α-Phe), 47.2 (CH, Fmoc), 44.3 (CH_2, GOx), 38.2 (CH_2, β-Tyr), 37.5 (CH_2, β-Phe), 30.9 (CH, β-Val), 29.0 (CH_3, *t*Bu), 28.5 (CH_3, *t*Bu), 19.4 (CH_3, γ-Val), 18.7 (CH_3, γ-Thr), 18.1 (CH_3, γ-Val). *N.B.* Two aromatic C and three aromatic CH signals not observed; **ν_{max}** (neat) = 2971, 2927, 1733, 1644, 1505, 1232, 1160, 757 cm^{-1}; **MS** (ESI^+) *m/z* 1038 $[M+H]^+$, 1060 $[M+Na]^+$; **HRMS** (ESI^+) calcd. for $C_{61}H_{75}N_5NaO_{10}$ $[M+Na]^+$ 1060.5406, found 1060.5381; $[\alpha]_D^{27}$ +19.5 (*c* 0.10, $CHCl_3$).

Cbz-Leu-Tyr(*t*Bu)-Val-GOx-Thr(*t*Bu)-Phe-OBn (118)

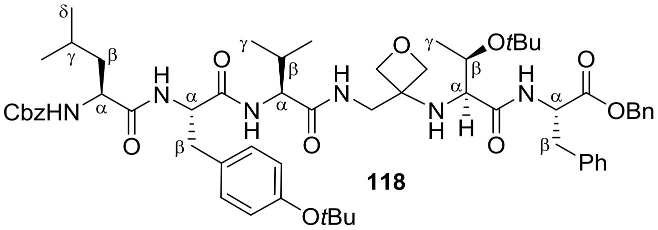

118

To a solution of pentapeptide Fmoc-Tyr(*t*Bu)-Val-GOx-Thr(*t*Bu)-Phe-OBn (**117**) (1.80 g, 1.73 mmol, 1.0 equiv) in CH_2Cl_2 (2.0 mL) was added diethylamine (2.0 mL) and the mixture was stirred at room temperature for 1 h. The reaction mixture was concentrated under reduced pressure and the resulting residue repeatedly dissolved in CH_2Cl_2 (3 × 20 mL) and concentrated under reduced pressure to give the crude amine. The residue was dissolved in CH_2Cl_2 (20 mL), Cbz-Leu-OH (0.50 g, 1.90 mmol, 1.1 equiv), EDC·HCl (0.36 g, 1.90 mmol, 1.1 equiv), HOBt·H_2O (0.26 g, 1.90 mmol, 1.1 equiv) and NMM (0.84 mL, 7.60 mmol, 4.0 equiv) were added subsequently, and the mixture was stirred at room temperature for 24 h. The reaction mixture was diluted with EtOAc (50 mL) and washed with brine (3 × 50 mL), dried over Na_2SO_4, filtered and concentrated under reduced pressure. The residue was purified by column chromatography (SiO_2, CH_2Cl_2/MeOH 49:1) to give hexapeptide Cbz-Leu-Tyr(*t*Bu)-Val-GOx-Thr(*t*Bu)-Phe-OBn (**118**) (1.37 g, 1.29 mmol, 74%) as a white solid. $\mathbf{R_f}$ (CH_2Cl_2/MeOH 49:1) 0.16; **mp** 146–148 °C; **^{1}H NMR** (500 MHz, $CDCl_3$) δ_H 7.80 (d, 1H, *J* = 8.1 Hz, NH), 7.39–7.25 (m, 10H, ArH), 7.25–7.21 (m, 3H, ArH), 7.14–7.04 (m, 5H, 4 × ArH, NH), 6.88 (d, *J* = 8.3 Hz, 2H, ArH), 6.75 (d, *J* = 7.6 Hz, 1H, NH), 6.68 (d, *J* = 6.5 Hz, 1H, NH), 5.16 (d, *J* = 12.2 Hz, 1H, C*H*HPh), 5.11–5.04 (m, 3H, CH_2Ph, NH), 4.98 (d, *J* = 12.2 Hz, 1H, CH*H*Ph), 4.89 (dd, *J* = 13.8, 7.5 Hz, 1H, CHα-Tyr), 4.60–4.52 (m, 1H, CHα-Phe), 4.42 (d, *J* = 6.4 Hz, 1H, OC*H*H-Ox), 4.34 (d, *J* = 6.4 Hz, 1H, OCH*H*-Ox), 4.32–4.25 (m, 3H, OCH_2-Ox, CHα-Val), 4.08–4.02 (m, 1H, CHα-Leu), 3.81 (dd, *J* = 13.9, 7.0 Hz, 1H, C*H*HGOx), 3.68 (app. quint, *J* = 5.8 Hz, 1H, CHα-Thr), 3.22–3.00 (m, 6H, CH*H*GOx, CHβ-Thr, CH_2β-Phe, CH_2β-Tyr), 2.63 (s, 1H, NH), 2.27–2.15 (m, 1H, CHβ-Val), 1.63–1.48 (m, 2H, C*H*Hβ-Leu, CHγ-Leu), 1.44–1.36 (m, 1H, CH*H*β-Leu), 1.30 (s, 9H, 3 × CH_3, *t*Bu), 1.12 (s, 9H, 3 × CH_3, *t*Bu), 1.02 (d, *J* = 6.2 Hz, 3H, CH_3γ-Thr), 0.93–0.80 (m, 12H, 2 × CH_3 γ-Val, 2 × CH_3δ-Leu); **^{13}C NMR** (126 MHz, $CDCl_3$) δ_C 173.4 (C=O), 172.7 (C=O), 171.8 (C=O), 171.6 (C=O), 171.1 (C=O), 156.4 (C=O, Cbz), 154.6 (C), 136.1 (C), 136.0 (C), 135.2 (C), 131.3 (C), 129.8 (CH), 129.3 (CH), 128.8 (CH), 128.7 (CH), 128.6 (CH), 128.5 (CH), 128.3 (CH), 127.3 (CH), 124.5 (CH), 79.8 (OCH_2), 79.1 (OCH_2), 78.5 (C, *t*Bu), 74.8 (C, *t*Bu), 70.0 (CH, α-Thr), 67.5 (CH_2, Bn), 67.4 (CH_2, Bn), 61.4 (CH, β-Thr), 60.2 (C, Ox), 59.1 (CH, α-Val), 55.4 (CH, α-Phe), 54.1 (CH, α-Leu), 53.4 (CH, α-Tyr), 44.3 (CH_2, GOx), 41.0 (CH_2, β-Leu), 38.2 (CH_2, β-Phe), 36.6 (CH_2, β-Tyr), 30.3 (CH, β-Val), 29.0 (CH_3, *t*Bu), 28.5 (CH_3, *t*Bu), 24.8 (CH, γ-Leu), 23.0 (CH_3, δ-Leu), 21.9 (CH_3, δ-Leu), 19.5 (CH_3, γ-Val), 18.4 (CH_3, γ-Thr), 17.8 (CH_3, γ-Val). *N.B.* Two aromatic CH signals not observed; $\mathbf{\nu_{max}}$ (neat) = 2969, 2932, 1739, 1694, 1532, 1505, 1232, 1161, 696 cm^{-1}; **MS** (ESI^+) *m/z* 1063 $[M+H]^+$, 1085 $[M+Na]^+$; **HRMS** (ESI^+) calcd. for $C_{60}H_{82}N_6NaO_{11}$ $[M+Na]^+$ 1085.5934, found 1085.5936; $[\boldsymbol{\alpha}]_D^{27}$ +13.5 (*c* 0.10, $CHCl_3$).

H-Leu-Tyr(*t*Bu)-Val-GOx-Thr(*t*Bu)-Phe-OH (119)

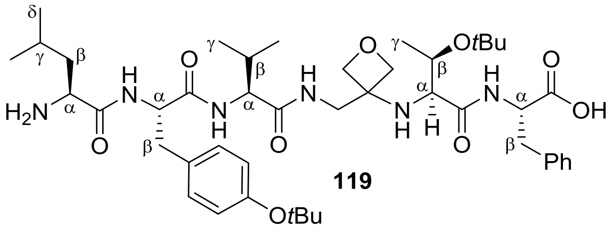

119

To a solution of **118** (1.33 g, 1.25 mmol, 1.0 equiv) in MeOH (15 mL) was added 10 wt% Pd/C (133 mg, 10 wt%) and the reaction flask was evacuated, filled with nitrogen, evacuated, and placed under an atmosphere of hydrogen (balloon). The reaction mixture was stirred at room temperature for 16 h, placed under nitrogen and filtered through a plug of Celite, which was washed with MeOH (3×). The filtrate was concentrated *in vacuo* to give **119** as a white solid (1.04 g, 1.25 mmol, quant. yield). **mp** 143–145 °C; **^{1}H NMR**

(500 MHz, CD_3OD) δ_H 7.28–7.16 (m, 7H, ArH), 6.91 (d, *J* = 8.4 Hz, 2H, ArH), 4.69 (dd, *J* = 9.8, 5.2 Hz, 1H, CHα-Tyr or CHα-Phe), 4.65 (dd, *J* = 8.3, 4.9 Hz, 1H, CHα-Tyr or CHα-Phe), 4.39 (d, *J* = 6.5 Hz, 1H, OC*H*H-Ox), 4.35 (d, *J* = 6.6 Hz, 1H, OC*H*H-Ox), 4.32 (d, *J* = 6.5 Hz, 1H, OCH*H*-Ox), 4.28 (d, *J* = 6.6 Hz, 1H, OCH*H*-Ox), 4.16 (d, *J* = 7.6 Hz, 1H, CHα-Val), 3.87–3.80 (m, 2H, C*H*HGOx, CHα-Leu), 3.68–3.60 (m, 1H, CHα-Thr), 3.24 (dd, *J* = 13.8, 4.7 Hz, 1H, C*H*Hβ-Tyr or C*H*Hβ-Phe), 3.14 (dd, *J* = 14.1, 5.1 Hz, 1H, C*H*Hβ-Tyr or C*H*Hβ-Phe), 3.06–2.99 (m, 3H, CHβ-Thr, CH*H*GOx, CH*H*β-Tyr or CH*H*β-Phe), 2.96 (dd, *J* = 14.1, 9.9 Hz, 1H, CH*H*β-Tyr or CH*H*β-Phe), 2.07–1.96 (m, 1H, CHβ-Val), 1.69–1.56 (m, 3H, CH_2β-Leu, CHγ-Leu), 1.32 (s, 9H, 3 × CH_3, *t*Bu), 1.18 (s, 9H, 3 × CH_3, *t*Bu), 1.06 (d, *J* = 6.1 Hz, 3H, CH_3γ-Thr), 0.99–0.87 (m, 12H, 2 × CH_3δ-Leu, 2 × CH_3γ-Val); **^{13}C NMR** (126 MHz, CD_3OD) δ_C 176.6 (C=O), 175.4 (C=O), 174.1 (C=O), 173.5 (C=O), 170.8 (C=O), 155.4 (C), 139.0 (C), 133.5 (C), 130.9 (CH), 130.6 (CH), 129.5 (CH), 127.8 (CH), 125.2 (CH), 80.7 (OCH_2), 80.5 (OCH_2), 79.5 (C, *t*Bu), 75.7 (C, *t*Bu), 70.5 (CH, α-Thr), 64.4 (CH, β-Thr), 62.0 (C, Ox), 61.5 (CH, α-Val), 57.2 (CH, α-Tyr or CH, α-Phe), 55.9 (CH, α-Tyr or CH, α-Phe), 52.9 (CH, α-Leu), 45.1 (CH_2, GOx), 41.7 (CH_2, β-Leu), 39.3 (CH_2, β-Tyr or CH_2, β-Phe), 37.9 (CH_2, β-Tyr or CH_2, β-Phe), 32.3 (CH, β-Val), 29.2 (CH_3, *t*Bu), 29.1 (CH_3, *t*Bu), 25.2 (CH, γ-Leu), 23.3 (CH_3, δ-Leu), 21.9 (CH_3, δ-Leu), 21.0 (CH_3, γ-Thr), 19.8 (CH_3, γ-Val), 19.2 (γ-CH_3, Val); **ν_{max}** (neat) = 3064, 2968, 2873, 1643, 1505, 1160, 698 cm^{-1}; **MS** (ESI^+) *m/z* 839 $[M+H]^+$, 861 $[M+Na]^+$; **HRMS** (ESI^+) calcd. for $C_{45}H_{71}N_6O_9$ $[M+H]^+$ 839.5277, found 839.5273; $[\alpha]_D^{27}$ –9.6 (*c* 0.16, MeOH).

Cyclo(Leu-Tyr(*t*Bu)-Val-GOx-Thr(*t*Bu)-Phe) (14)

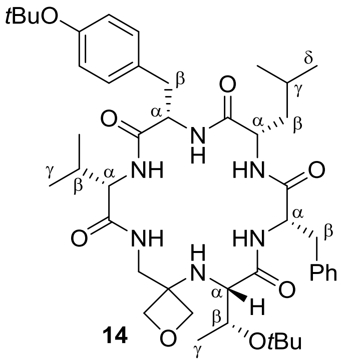

To a solution of H-Leu-Tyr(*t*Bu)-Val-GOx-Thr(*t*Bu)-Phe-OH (**119**) (416 mg, 0.50 mmol, 1.0 equiv) in anhydrous DMF (100 mL, 0.005 M) under an atmosphere of nitrogen was added DEPBT (297 mg, 0.99 mmol, 2.0 equiv) and DIPEA (172 µL, 0.99 mmol, 2.0 equiv) and the reaction mixture was stirred for 48 h at room temperature. The solvent was removed under reduced pressure at 60 °C over 30 min, and the residue was dried *in vacuo*. The residue was analysed by LCMS and purified twice by column chromatography (SiO_2, CH_2Cl_2/MeOH 49:1→19:1) to give the cyclic hexapeptide (**14**) as a white solid (235 mg, 0.29 mmol, 58%). **R_f** (CH_2Cl_2/MeOH 49:1) 0.22; **mp** 127–129 °C; **^{1}H NMR** (500 MHz, CD_3OD) δ_H 7.36–7.31 (m, 2H, ArH), 7.30–7.23 (m, 3H, ArH), 7.17 (d, *J* = 8.3 Hz, 2H, ArH), 6.94 (d, *J* = 8.4 Hz, 2H, ArH), 4.63 (dd, *J* = 9.6, 6.2 Hz, 1H, CHα-Phe), 4.50–4.45 (m, 3H, OCH_2-Ox, OC*H*H-Ox), 4.41–4.35 (m, 2H, OCH*H*-Ox, CHα-Val), 4.14 (dd, *J* = 11.4, 3.6 Hz, 1H, CHα-Tyr), 4.03 (d, *J* = 14.9 Hz, 1H, C*H*HGOx), 3.79–3.71 (m, 1H, CHα-Leu), 3.59–3.45 (m, 2H, CHα-Thr, C*H*Hβ-Tyr), 3.37 (dd, *J* = 13.9, 3.9 Hz, 1H, CH*H*β-Tyr), 3.29–3.18 (m, 2H, C*H*Hβ-Phe, CH*H*GOx), 3.07 (d, *J* = 4.3 Hz, 1H, CHβ-Thr), 2.75 (dd, *J* = 13.8, 9.8 Hz, 1H, CH*H*β-Phe), 2.13–2.03 (m, 1H, CHβ-Val), 1.58–1.50 (m, 1H, C*H*Hβ-Leu), 1.36–1.28 (m, 10H, CH*H*β-Leu, 3 × CH_3, *t*Bu), 1.20–1.15 (m, 1H, CHγ-Leu), 1.10–1.03 (m, 18H, 2 × CH_3γ-Val, CH_3γ-Thr, 3 × CH_3, *t*Bu), 0.81 (d, *J* = 6.5 Hz, 3H, CH_3δ-Leu), 0.77 (d, *J* = 6.5 Hz, 3H, CH_3δ-Leu); **^{13}C NMR** (126 MHz, CD_3OD) δ_C 176.4 (C=O), 174.9 (C=O), 174.5 (C=O), 174.4 (C=O), 173.0 (C=O), 155.3 (C), 137.7 (C), 134.7 (C), 130.6 (CH), 130.2 (CH), 129.9 (CH), 128.3 (CH), 125.3 (CH), 80.3 (OCH_2), 80.2 (OCH_2), 79.4 (C, *t*Bu), 75.3 (C, *t*Bu), 69.2 (CH, α-Thr), 64.8 (CH, β-Thr), 62.4 (CH, α-Val), 62.1 (C, Ox), 59.3 (CH, α-Tyr), 55.7 (CH, α-Leu), 54.7 (CH, α-Phe), 45.6 (CH_2, GOx), 40.2 (CH_2, β-Leu), 39.5 (CH_2, β-Phe), 35.3 (CH_2, β-Tyr), 33.2 (CH, β-Val), 29.2 (CH_3, *t*Bu), 28.9 (CH_3, *t*Bu), 25.2 (CH, γ-Leu), 23.2 (CH_3, δ-Leu), 22.1 (CH_3, δ-Leu), 22.0 (CH_3, γ-Val), 19.9 (CH_3, γ-Val), 19.5 (CH_3, γ-Thr); **ν_{max}** (neat) = 2964, 2930, 1641, 1504, 1389, 1161 cm^{-1}; **MS** (ESI^+) *m/z* 843 $[M+Na]^+$; **HRMS** (ESI^+) calcd. for $C_{45}H_{68}N_6NaO_8$ $[M+Na]^+$ 843.4991, found 843.4995; $[\alpha]_D^{27}$ –74.3 (*c* 0.13, MeOH).

*2.14 Preparation of cyclic peptides **18-21** via SPPS*

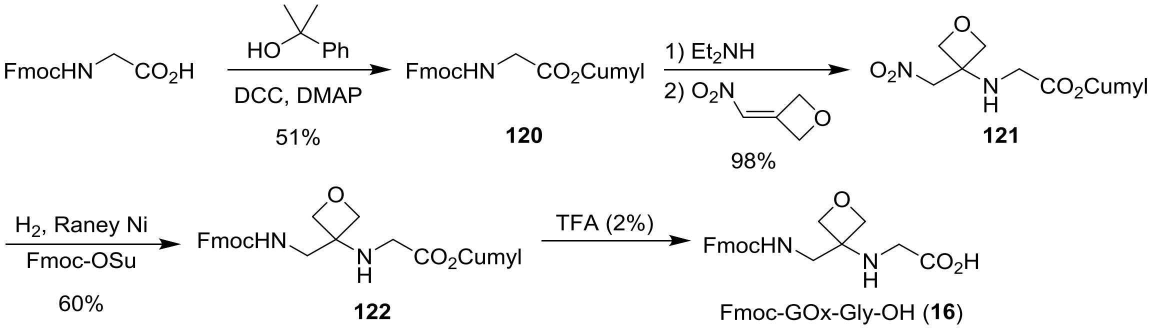

Fmoc-Gly-OCumyl (120)

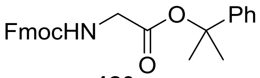

120

To a solution of Fmoc-Gly-OH (2.00 g, 6.72 mmol, 1.0 equiv) in anhydrous CH_2Cl_2 (30 mL) were added 2-phenyl-2-propanol (3.36 g, 24.7 mmol, 3.7 equiv), DCC (1.70 g, 8.24 mmol, 1.2 equiv) and DMAP (167 mg, 1.37 mmol, 0.2 equiv) and the mixture was stirred for 24 h at room temperature. The solvent was removed *in vacuo*, the residue was diluted with diethyl ether (100 mL) and filtered through a plug of Celite eluting with diethyl ether. The filtrate was washed with saturated $NaHCO_3$ solution (100 mL), dried over $MgSO_4$, filtered, and concentrated under reduced pressure. The residue was purified by column chromatography (SiO_2, PE/EtOAc 9:1→4:1) to give Fmoc-Gly-OCumyl (**120**) (1.43 g, 3.44 mmol, 51%) as a white solid. **R_f** (PE/EtOAc 2:1) 0.46; **mp** 109–111 °C; **^{1}H NMR** (500 MHz, $CDCl_3$) δ_H ppm 7.78 (d, J = 7.5 Hz, 2H, ArH), 7.60 (d, J = 7.5 Hz, 2H, ArH), 7.43–7.26 (m, 9H, ArH), 5.31 (s, 1H, NH), 4.40 (d, J = 7.2 Hz, 2H, CH_2-Fmoc), 4.23 (t, J = 7.2 Hz, 1H, CH-Fmoc), 4.01 (d, J = 5.3 Hz, 2H, CH_2Gly), 1.84 (s, 6H, 2 × CH_3, cumyl); **^{13}C NMR** (126 MHz, $CDCl_3$) δ_C ppm 168.7 (C=O), 156.3 (C=O, Fmoc), 145.1 (C), 143.9 (C), 141.3 (C), 128.5 (CH), 127.8 (CH), 127.4 (CH), 127.1 (CH), 125.2 (CH), 124.4 (CH), 120.1 (CH), 83.4 (C, cumyl), 67.2 (CH_2, Fmoc), 47.2 (CH, Fmoc), 43.5 (CH_2, Gly), 28.6 (CH_3, cumyl); **ν_{max}** (neat) = 3308, 2938, 1738, 1687, 1546, 1214, 1053, 760, 697 cm^{-1}; **MS** (ESI$^+$) *m/z* 438 [M+Na]$^+$, 454 [M+K]$^+$; **HRMS** (ESI$^+$) calcd. for $C_{26}H_{25}NNaO_4$ [M+Na]$^+$ 438.1676, found 438.1674.

O_2N-GOx-Gly-OCumyl (121)

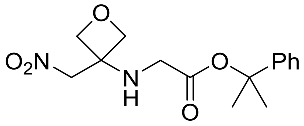

121

To a solution of Fmoc-Gly-OCumyl (**120**) (1.29 g, 3.10 mmol, 1.0 equiv) in CH_2Cl_2 (4.0 mL) was added diethylamine (4.0 mL) and the mixture was stirred at room temperature for 2 h. The reaction mixture was concentrated under reduced pressure and the resulting residue repeatedly dissolved in CH_2Cl_2 (3 × 15 mL) and concentrated under reduced pressure to give the crude amine. In a second reaction vessel, oxetane-3-one (398 µL, 6.21 mmol, 2.0 equiv), nitromethane (470 µL, 8.68 mmol, 2.8 equiv) and trimethylamine (173 µL, 1.24 mmol, 0.4 equiv) were combined at 0 °C and stirred for 1 h at room temperature. The mixture was dissolved in anhydrous CH_2Cl_2 (20 mL), cooled to –78 °C, and trimethylamine (1.73 mL, 12.4 mmol, 4.0 equiv) was added followed by dropwise addition of a solution of methanesulfonyl chloride (481 µL, 6.21 mmol, 2.0 equiv) in anhydrous CH_2Cl_2 (6.0 mL). The reaction mixture was stirred at –78 °C for 1.5 h and a solution of the crude amine in anhydrous CH_2Cl_2 (20 mL) was added slowly *via* syringe. The reaction mixture was allowed to warm to room temperature and stirred for 16 h. A saturated solution of NH_4Cl (20 mL) was added and stirred for 10 min. The layers were separated and the aqueous one extracted with CH_2Cl_2 (2 × 30 mL) and EtOAc (2 × 30 mL). The combined organic phases were concentrated under reduced pressure and the residue was purified by column chromatography (SiO_2, PE/EtOAc 4:1→2:1→1:1) to give **121** (940 mg,

3.05 mmol, 98%) as an off-white solid. **R$_f$** (PE/EtOAc 2:1) 0.15; **mp** 71–72 °C; **^{1}H NMR** (400 MHz, $CDCl_3$) δ_H ppm 7.39–7.27 (m, 5H, ArH), 4.77 (s, 2H, NO_2CH_2), 4.57 (d, *J* = 7.2 Hz, 2H, OCH_2-Ox), 4.53 (d, *J* = 7.2 Hz, 2H, OCH_2-Ox), 3.53 (s, 2H, CH_2Gly), 2.29 (s, 1H, NH), 1.80 (s, 6H, 2 x CH_3, cumyl); **^{13}C NMR** (101 MHz, $CDCl_3$) δ_C ppm 170.6 (C=O), 145.1 (C), 128.5 (CH), 127.5 (CH), 124.4 (CH), 83.3 (C, cumyl), 78.9 (NO_2CH_2), 78.3 (2 × OCH_2), 59.6 (C, Ox), 45.6 (CH_2, Gly), 28.6 (CH_3, cumyl); **ν_{max}** (neat) = 3293, 2979, 1736, 1545, 1364, 1215, 1140, 1101, 978, 762, 695 cm^{-1}; **MS** (ESI$^+$) *m/z* 331 $[M+Na]^+$; **HRMS** (ESI$^+$) calcd. for $C_{15}H_{20}N_2NaO_5$ $[M+Na]^+$ 331.1264, found 331.1268.

Fmoc-GOx-Gly-OCumyl (122)

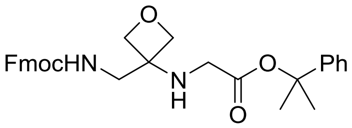

122

To a solution of NO_2-GOx-Gly-OCumyl (**121**) (928 mg, 3.00 mmol, 1.0 equiv) in THF (30 mL) was added Fmoc *N*-hydroxysuccinimide ester (2.02 g, 6.00 mmol, 2.0 equiv), $NaHCO_3$ (1.01 g, 12.0 mmol, 4.0 equiv) and Raney Ni (slurry in H_2O, 3.0 mL). The reaction mixture was placed under an atmosphere of nitrogen, evacuated and filled with hydrogen (balloon). The mixture was stirred vigorously for 4 h at room temperature. Then, the mixture was filtered through a plug of Celite eluting with EtOAc, the filtrate was washed with saturated Na_2CO_3 (3 × 50 mL) and concentrated under reduced pressure. Fmoc-GOx-Gly-OCumyl (**122**) was afforded after purification by column chromatography (SiO_2, PE/EtOAc 2:1→1:1→EtOAc) as a white sticky foam (897 mg, 1.79 mmol, 60%). **R$_f$** (PE/EtOAc 1:1) 0.21; **^{1}H NMR** (400 MHz, $CDCl_3$) δ_H ppm 7.72 (d, *J* = 7.4 Hz, 2H, ArH), 7.54 (d, *J* = 7.4 Hz, 2H, ArH), 7.38–7.21 (m, 9H, ArH), 5.26 (s, 1H, NH), 4.48–4.24 (m, 6H, CH_2-Fmoc, 2 x OCH_2-Ox), 4.16 (t, *J* = 6.2 Hz, 1H, CH-Fmoc), 3.50 (d, *J* = 5.1 Hz, 2H, CH_2GOx), 3.41 (s, 2H, CH_2Gly), 1.92 (br. s, 1H, NH), 1.76 (s, 6H, 2 × CH_3, cumyl); **^{13}C NMR** (101 MHz, $CDCl_3$) δ_C ppm 171.4 (C=O), 157.0 (C=O, Fmoc), 145.1 (C), 144.0 (C), 141.4 (C), 128.5 (CH), 127.8 (CH), 127.5 (CH), 127.2 (CH), 125.2 (CH), 124.4 (CH), 120.1 (CH), 83.2 (C, cumyl), 79.2 (2 × OCH_2), 66.9 (CH_2, Fmoc), 59.7 (C, Ox), 47.3 (CH, Fmoc), 45.5 (CH_2, GOx or Gly), 45.4 (CH_2, GOx or Gly), 28.6 (CH_3, cumyl); **ν_{max}** (neat) = 3309, 2941, 1716, 1535, 1448, 1214, 1134, 974, 758, 739, 698 cm^{-1}; **MS** (ESI$^+$) *m/z* 501 $[M+H]^+$, 523 $[M+Na]^+$.; **HRMS** (ESI$^+$) calcd. for $C_{30}H_{32}N_2NaO_5$ $[M+Na]^+$ 523.2203, found 523.2197.

*Solid-phase peptide synthesis of cyclo(Leu-GOx-Gly-Trp(Boc)) (**18**)*

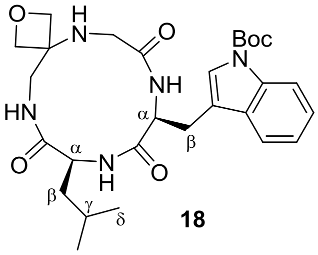

18

Fmoc-GOx-Gly-Cumyl (**122**) (200 mg, 0.40 mmol, 4.0 equiv) was stirred at room temperature in 2% TFA in CH_2Cl_2 (8.0 mL) for 2–3 h until complete deprotection of the cumyl ester was observed by TLC. The solvent was removed under reduced pressure and the resulting residue was repeatedly dissolved in CH_2Cl_2 (3 × 10 mL) and concentrated under reduced pressure. The crude Fmoc-GOx-Gly-OH (**16**) was used for coupling without further purification.

H-Trp(Boc)-2-chlorotrityl resin (**15**) (145 mg, 0.10 mmol, 1.0 equiv) was placed in a 10 mL reaction vessel and the resin was pre-swollen in DMF (2.0 mL) for 30 min. Fmoc-GOx-Gly-OH (**16**) was dissolved in DMF (4.0 mL). HATU (72 mg, 0.19 mmol, 1.9 equiv) and DIPEA (70 µL, 0.40 mmol, 4.0 equiv) were added to 2.0 mL solution of **16** and the coupling solution was added to the resin. The coupling reaction was allowed to proceed for 2 h at room temperature under slight agitation. The resin was filtered, washed with DMF (1 × 2.0 mL) and the coupling step was repeated before the Fmoc group was removed with 20% piperidine in DMF (2.0 mL) for 20 min at room temperature. After washing the resin with DMF (5 × 2.0 mL), Fmoc-Leu-OH (177 mg, 0.50 mmol, 5.0 equiv) was coupled with HATU

(186 mg, 0.49 mmol, 4.9 equiv), DIPEA (174 µL, 1.00 mmol, 10 equiv) in DMF (2.0 mL) for 1 h at room temperature. In case of a positive TNBS test, the coupling step was repeated. The resin was washed with DMF (5 × 2.0 mL) before the Fmoc-group was removed as described before. The tetrapeptide was then cleaved from the resin with TFE in CH_2Cl_2 (1:4, 1.0 mL) for 1 h at room temperature. This was repeated twice and the combined cleavage solutions were evaporated to dryness under reduced pressure. Success of the synthesis was confirmed by mass spectrometry and NMR. The crude yield of the solid phase synthesis was approximately 70–80%.

The crude peptide was dissolved in DMF (76 mL, ~1 mM) and DEPBT (45 mg, 0.15 mmol, 2.0 equiv) and DIPEA (26 µL, 0.15 mmol, 2.0 equiv) were added. The reaction mixture was stirred at room temperature for 64 h before, the solvent was removed under reduced pressure and the residue purified twice by column chromatography (5–12% MeOH in CH_2Cl_2). Cyclic tetrapeptide **18** was obtained as a sticky white/colourless solid in 39% yield (21.1 mg, 39 µmol) over the complete reaction sequence.

R_f (CH_2Cl_2/MeOH 9:1) 0.55; **^{1}H NMR** (500 MHz, DMSO-*d6*) δ_H 8.25 (d, *J* = 10.3 Hz, 1H, NH), 8.03 (d, *J* = 8.1 Hz, 1H, ArH), 7.95 (d, *J* = 9.1 Hz, 1H, NH), 7.63 (d, *J* = 7.7 Hz, 1H, ArH), 7.48 (s, 1H, ArH), 7.47–7.43 (m, 1H, NH), 7.34 (t, *J* = 7.7 Hz, 1H, ArH), 7.27 (t, *J* = 7.5 Hz, 1H, ArH), 4.61 (q, *J* = 8.8 Hz, 1H, CHα-Trp), 4.40 (d, *J* = 6.3 Hz, 1H, OC*H*H-Ox), 4.17 (d, *J* = 6.9 Hz, 1H, OC*H*H-Ox), 4.15 (d, *J* = 6.3 Hz, 1H, OCH*H*-Ox), 4.01 (td, *J* = 9.8, 5.2 Hz, 1H, CHα-Leu), 3.89 (d, *J* = 6.9 Hz, 1H, OCH*H*-Ox), 3.80 (dd, *J* = 13.4, 7.8 Hz, 1H, C*H*HGOx), 3.44–3.35 (m, 1H, C*H*HGly), 3.24 (d, *J* = 15.2 Hz, 1H, C*H*HGly), 3.18 (dd, *J* = 15.0, 5.6 Hz, 1H, C*H*Hβ-Trp), 3.07–3.03 (m, 1H, CH*H*β-Trp), 3.01 (d, *J* = 11.3 Hz, 1H, CH*H*GOx), 1.62 (s, 9H, 3 × CH_3, Boc), 1.61–1.56 (m, 2H, C*H*Hβ-Leu, CHγ-Leu), 1.52–1.44 (m, 1H, CH*H*β-Leu), 0.91 (d, *J* = 6.2 Hz, 3H, CH_3δ-Leu), 0.78 (d, *J* = 6.2 Hz, 3H, CH_3δ-Leu). *N.B.* One NH not observed; **^{13}C NMR** (126 MHz, DMSO-*d6*) δ_C 173.13 (C=O), 173.09 (C=O), 171.0 (C=O), 148.9 (C=O, Boc), 134.7 (C), 130.0 (C), 124.5 (CH), 123.4 (CH), 122.6 (CH), 119.1 (CH), 116.1 (C), 114.8 (CH), 83.7 (C, Boc), 78.1 (OCH_2), 76.3 (OCH_2), 60.3 (C, Ox), 55.2 (CH, α-Trp), 54.0 (CH, α-Leu), 47.2 (CH_2, Gly), 44.3 (CH_2, GOx), 39.6 (CH_2, β-Leu), 27.7 (CH_3, Boc), 25.8 (CH_2, β-Trp), 24.6 (CH, γ-Leu), 22.9 (CH_3, δ-Leu), 21.1 (CH_3, δ-Leu); **ν_{max}** (neat) = 3266, 2925, 1672, 1532, 1225, 704 cm^{-1}; **MS** (ESI^+) *m/z* 564 $[M+Na]^+$; **HRMS** (ESI^+) calcd. for $C_{28}H_{39}N_5NaO_6^+$ $[M+Na]^+$ 564.2793, found 564.2791; $[\boldsymbol{\alpha}]_D^{27}$ +10.0 (*c* 0.05, $CHCl_3$).

*Solid-phase peptide synthesis of cyclo(Ala-Trp-GOx-Gly-Leu) (**19**)*

Cyclic peptide **19** was synthesised as described above starting from H-Leu-2-chlorotrityl resin (67.5 mg, 0.05 mmol). Tryptophan was incorporated without side chain protecting group. The crude cyclic peptide was purified by preparative HPLC (solvent A: 0.1% TFA in water; solvent B: 0.1% TFA in MeCN; gradient: 0–3 min, 5% B; 3–28 min, 3–40% B; 28–32 min, 40–100% B; retention time: 28.9 min). Cyclic pentapeptide **19** was obtained after freeze-drying as TFA salt (2.4 mg, 6.5 µmol, 13%).

HRMS (ESI^+) calcd. for $C_{26}H_{37}N_6O_5^+$ $[M+H]^+$ 513.2820, found 513.2816.

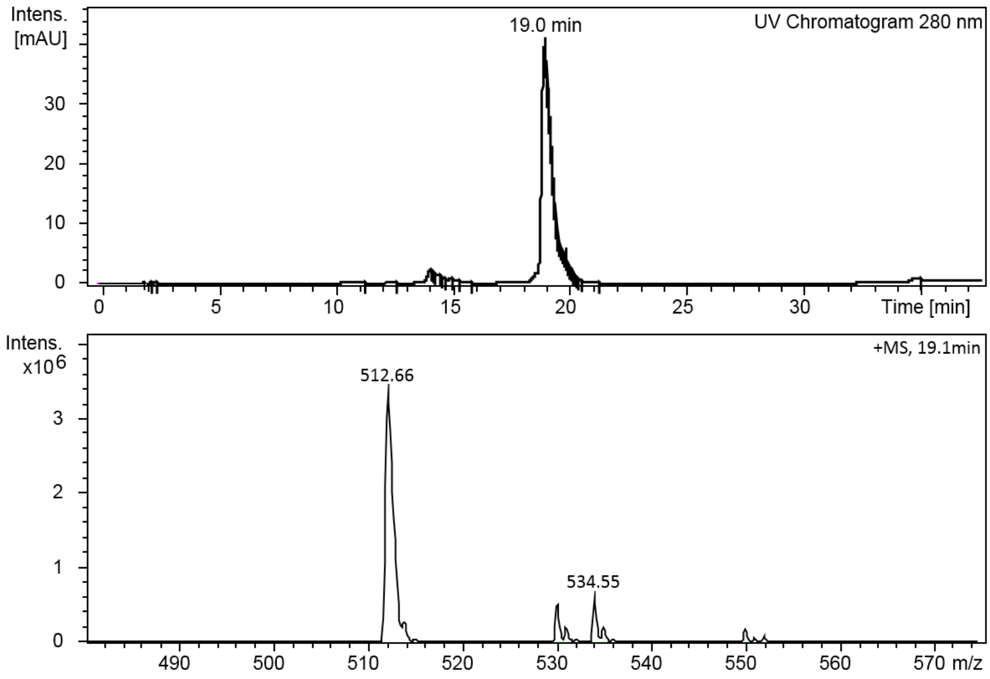

*Solid-phase peptide synthesis of cyclo(Met-Ala-Trp-GOx-Gly-Leu) (**20**)*

Cyclic peptide **20** was synthesised as described above starting from H-Leu-2-chlorotrityl resin (67.5 mg, 0.05 mmol). Tryptophan was incorporated without side chain protecting group. The crude cyclic peptide was purified by preparative HPLC (solvent A: 0.1% TFA in water; solvent B: 0.1% TFA in MeCN; gradient: 0–3 min, 5% B; 3–28 min, 3–50% B; 28–32 min, 50–100% B; retention time: 25.3 min). Cyclic hexapeptide **20** was obtained after freeze-drying as TFA salt (2.5 mg, 6.5 µmol, 7%).

HRMS (ESI$^+$) calcd. for $C_{31}H_{46}N_7O_6S^+$ $[M+H]^+$ 644.3225, found 644.3220.

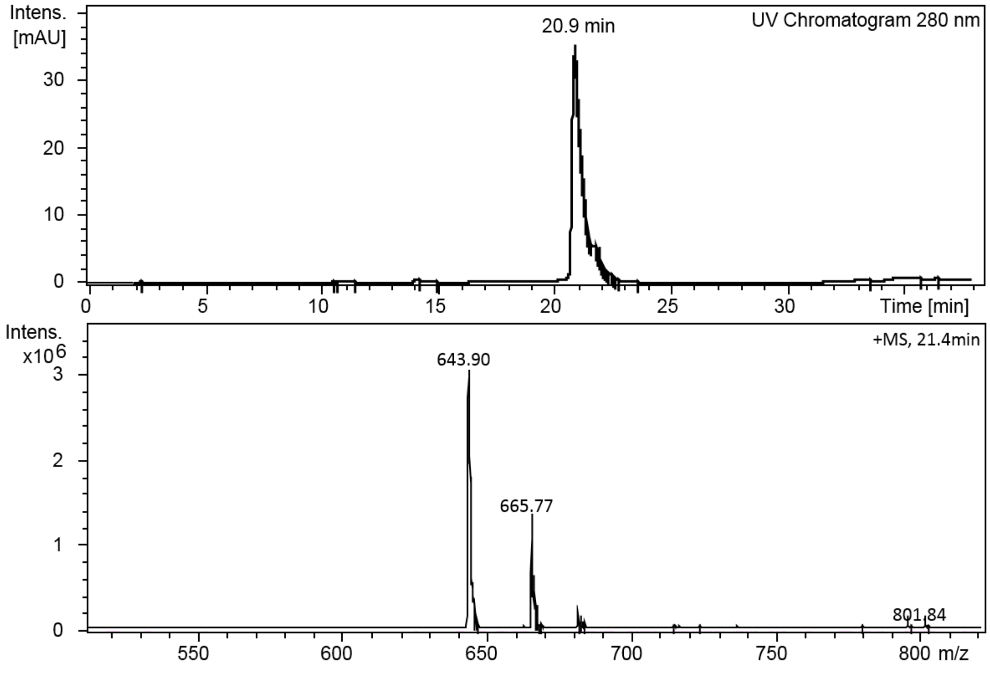

*Solid-phase peptide synthesis of cyclo(Ser-Met-Ala-Trp-GOx-Gly-Leu) (**21**)*

Cyclic peptide **21** was synthesised as described above starting from H-Leu-2-chlorotrityl resin (67.5 mg, 0.05 mmol). Tryptophan was incorporated without side chain protecting group. The crude cyclic peptide was purified by preparative HPLC (solvent A: 0.1% TFA in water; solvent B: 0.1% TFA in MeCN; gradient: 0–3 min, 5% B; 3–28 min, 3–50% B; 28–32 min, 50–100% B; retention time: 26.8 min). Cyclic heptapeptide **21** was obtained after freeze-drying as TFA salt (6.2 mg, 6.5 µmol, 13%).

HRMS (ESI^+) calcd. for $C_{34}H_{50}N_8NaO_8S^+$ $[M+Na]^+$ 753.3365, found 753.3358.

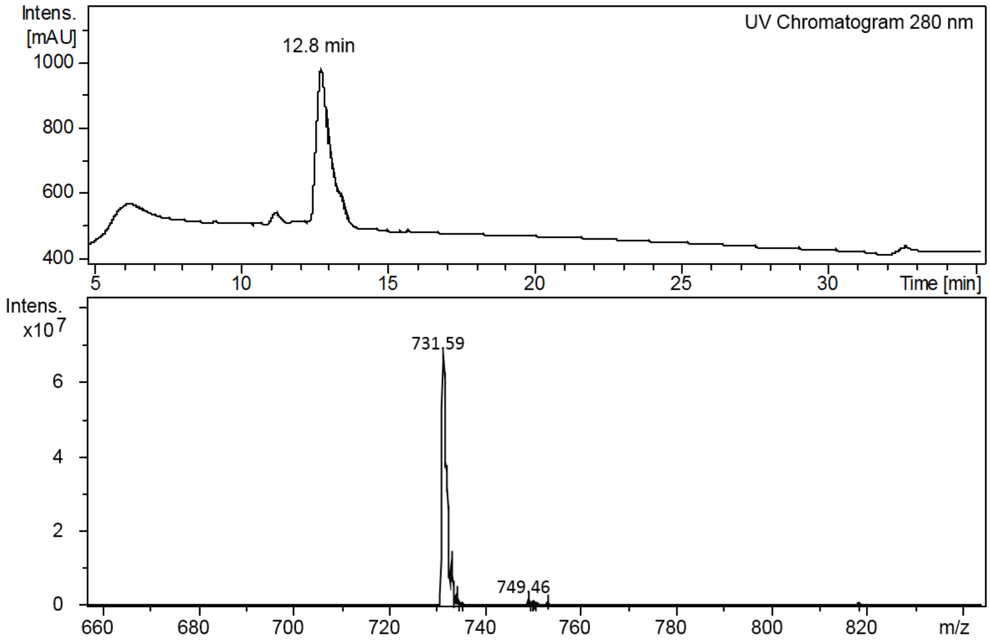

2.15 Preparation of cyclic disulfide-bridged pentapeptide **22**

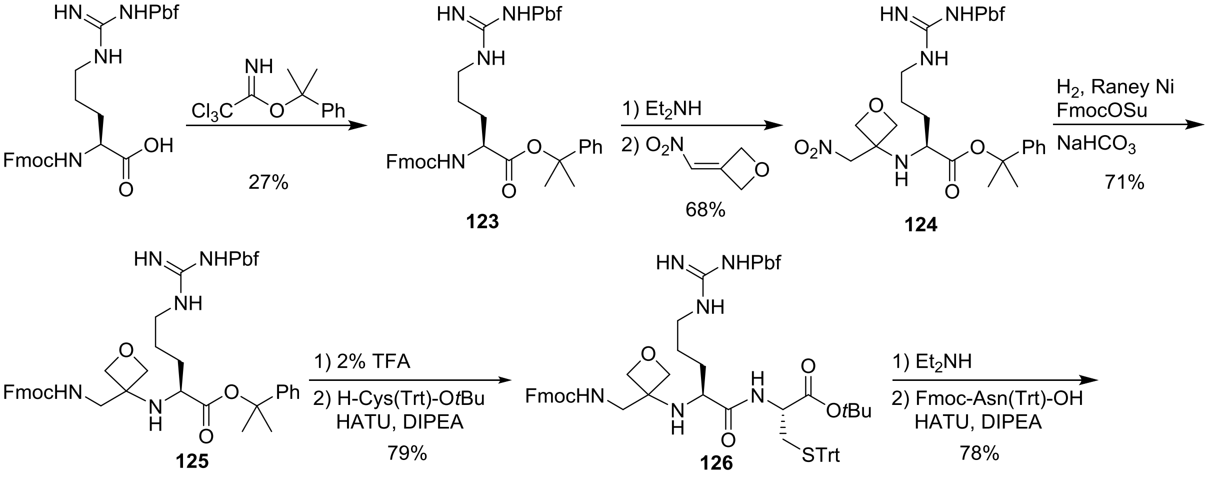

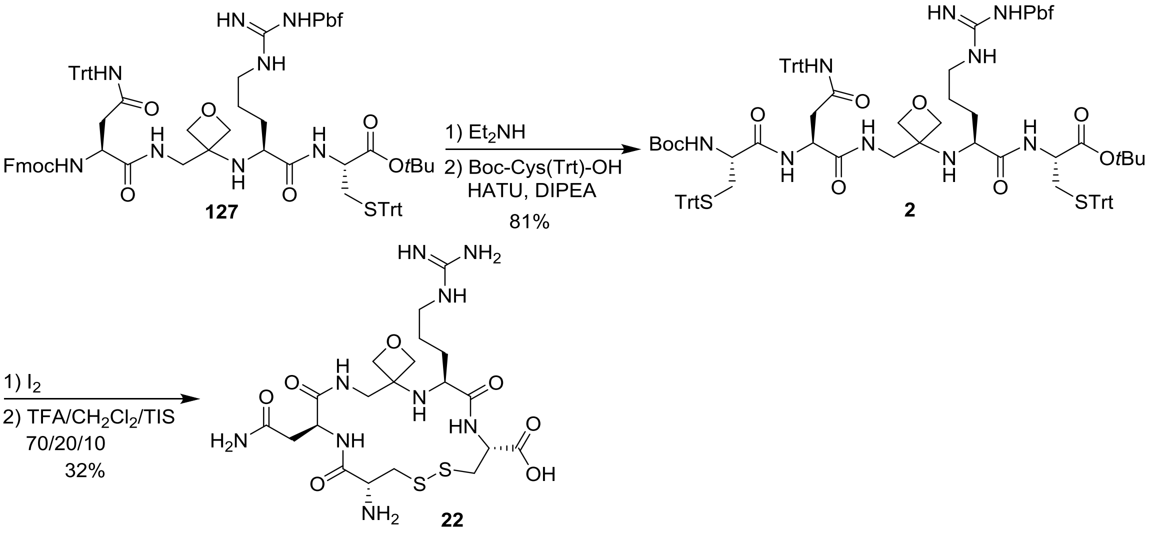

Fmoc-Arg(Pbf)-OCumyl (123)

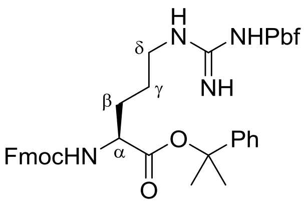

123

To sodium hydride (60% dispersion in mineral oil, 200 mg, 5.00 mmol, 0.5 equiv) in anhydrous diethyl ether (20 mL) was added freshly distilled 2-phenyl-2-propanol (3.00 g, 22.0 mmol, 2.2 equiv) at 0 °C and the mixture was stirred for 1 h at room temperature. The reaction mixture was cooled to 0 °C, 2,2,2-trichloroacetonitrile (2.00 mL, 20.0 mmol, 2.0 equiv) were added slowly and stirring was continued for 3 h at ambient temperature. The solvent was removed under reduced pressure and the residue re-dissolved in petroleum ether (5.0 mL), anhydrous MeOH (202 μL, 5.00 mmol, 0.5 equiv) was added and the solution was stirred for 10 min at room temperature. The mixture was filtered through a plug of Celite eluting with PE and the filtrate was concentrated under reduced pressure to give the crude imidate. To a suspension of Fmoc-Arg(Pbf)-OH (6.49 g, 10.0 mmol, 1.0 equiv) in CH_2Cl_2 (60 mL) was added a solution of the imidate in CH_2Cl_2 (15 mL) and the mixture was stirred for 16 h at room temperature. The reaction mixture was filtered through a plug of Celite eluting with CH_2Cl_2, the solvent was removed *in vacuo*, and the residue was purified by column chromatography (SiO_2, PE/EtOAc 1:1→EtOAc) to give Fmoc-Arg(Pbf)-OCumyl (**123**) (2.10 g, 2.74 mmol, 27%) as a white solid. **R_f** (EtOAc) 0.60; **mp** 100–102 °C; **^{1}H NMR** (400 MHz, $CDCl_3$) δ_H ppm 7.74 (d, J = 7.5 Hz, 2H, ArH), 7.54 (d, J = 7.5 Hz, 2H, ArH), 7.38 (t, J = 7.5 Hz, 2H, ArH), 7.35–7.26 (m, 7H, ArH), 5.92 (br. s, 3H, 3 × NH), 5.48 (d, J = 7.6 Hz, 1H, NH), 4.36 (d, J = 6.7 Hz, 2H, CH_2-Fmoc), 4.28 (d, J = 6.7 Hz, 1H, CHα-Arg), 4.16 (t, J = 7.0 Hz, 1H, CH-Fmoc), 3.34–3.23 (m, 1H, C*H*Hδ-Arg), 3.23–3.12 (m, 1H, CH*H*δ-Arg), 2.91 (s, 2H, CH_2, Pbf), 2.58 (s, 3H, CH_3, Pbf), 2.51 (s, 3H, CH_3, Pbf), 2.07 (s, 3H, CH_3, Pbf), 1.96–1.85 (m, 1H, C*H*Hβ-Arg), 1.79 (s, 3H, CH_3, cumyl), 1.76 (s, 3H, CH_3, cumyl), 1.69–1.55 (m, 3H, CH_2γ-Arg, CH*H*β-Arg), 1.43 (s, 6H, 2 × CH_3, Pbf); **^{13}C NMR** (101 MHz, $CDCl_3$) δ_C ppm 170.9 (C=O), 158.9 (C), 156.6 (C=O, Fmoc), 156.3 (C=NH), 144.9 (C), 143.8 (C), 143.7 (C), 141.4 (C), 138.5 (C), 133.1 (C), 132.4 (C), 128.5 (CH), 127.9 (CH), 127.5 (CH), 127.2 (CH), 125.2 (CH), 124.7 (C), 124.4 (CH), 120.1 (CH), 117.6 (C), 86.5 (C, Pbf), 83.7 (C, cumyl), 67.3 (CH_2, Fmoc), 53.8 (CH, α-Arg), 47.2 (CH, Fmoc), 43.3 (CH_2, Pbf), 40.9 (CH_2, δ-Arg), 30.4 (CH_2, β-Arg), 28.7 (CH_3, 2 × Pbf), 28.6 (CH_3, cumyl), 28.4 (CH_3, cumyl), 25.2 (CH_2, γ-Arg), 19.4 (CH_3, Pbf), 18.1 (CH_3, Pbf), 12.6 (CH_3, Pbf); **ν_{max}** (neat) = 3326, 2978, 1717, 1545, 1449, 1243, 1088, 758, 740 cm^{-1}; **MS** (ESI^+) *m/z* 767 $[M+Na]^+$, 789 $[M+Na]^+$; **HRMS** (ESI^+) calcd. for $C_{43}H_{50}N_4NaO_7S$ $[M+Na]^+$ 789.3292, found 789.3296; $[\boldsymbol{\alpha}]_D^{28}$ +1.66 (*c* 0.79, $CHCl_3$).

NO_2-GOx-Arg(Pbf)-OCumyl (124)

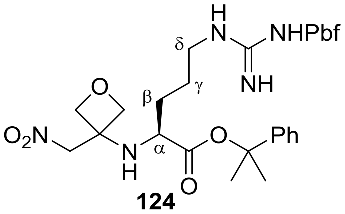

124

To a solution of Fmoc-Arg(Pbf)-OCumyl (**123**) (2.40 g, 3.13 mmol, 1.0 equiv) in CH_2Cl_2 (3.5 mL) was added diethylamine (3.5 mL) and the mixture was stirred at room temperature for 1 h. The reaction mixture was concentrated under reduced pressure and the resulting residue repeatedly dissolved in CH_2Cl_2 (3 × 10 mL) and concentrated under reduced pressure to give the crude amine. In a second reaction vessel, oxetane-3-one (410 μL, 6.26 mmol, 2.0 equiv), nitromethane (475 μL, 8.76 mmol, 2.8 equiv) and triethylamine (174 μL, 1.25 mmol, 0.4 equiv) were combined at 0 °C and stirred for 1 h at room temperature. The mixture was dissolved in anhydrous CH_2Cl_2 (24 mL), cooled to –78 °C, and triethylamine (1.74 mL, 12.5 mmol, 4.0 equiv) was added followed by dropwise addition of a solution of methanesulfonyl chloride (485 μL, 6.26 mmol, 2.0 equiv) in anhydrous CH_2Cl_2 (6.0 mL). The reaction mixture was stirred at –78 °C for 1.5 h and a solution of the crude amine in anhydrous CH_2Cl_2 (12 mL) was added slowly *via* syringe. The reaction mixture was allowed to warm to room temperature and stirred for 16 h. A saturated solution of NH_4Cl (30 mL) was added and stirred for 10 min. The layers were separated and the aqueous layer was extracted with CH_2Cl_2 (2 × 20 mL) and EtOAc (2 × 20 mL). The combined organic phases were washed with saturated aqueous $NaHCO_3$ solution (30 mL), brine (30 mL), dried over $MgSO_4$, filtered, and concentrated under reduced pressure. The residue was purified by column chromatography (SiO_2, PE/EtOAc 1:1→EtOAc) to give **124** (1.41 g, 2.14 mmol, 68%) as an orange foam. $\mathbf{R_f}$ (EtOAc) 0.48; **mp** 80–83 °C; **^{1}H NMR** (400 MHz, $CDCl_3$) δ_H ppm 7.35–7.30 (m, 4H, ArH), 7.28–7.23 (m, 1H, ArH), 5.97 (br. s, 2H, NH), 5.73 (br. s, 1H, NH), 4.77 (d, *J* = 12.8 Hz, 1H, C*H*HGOx), 4.68 (d, *J* = 12.8 Hz, 1H, CH*H*GOx), 4.54 (d, *J* = 7.3 Hz, 1H, OC*H*H-Ox), 4.49 (d, *J* = 7.2 Hz, 1H, OC*H*H-Ox), 4.40 (d, *J* = 7.3 Hz, 1H, OCH*H*-Ox), 4.37 (d, *J* = 7.2 Hz, 1H, OCH*H*-Ox), 3.43 (br. m, 1H, CHα-Arg), 3.22–3.08 (m, 2H, CH_2δ-Arg), 2.95 (s, 2H, CH_2, Pbf), 2.58 (s, 3H, CH_3, Pbf), 2.51 (s, 3H, CH_3, Pbf), 2.39–2.30 (br. s, 1H, NH), 2.09 (s, 3H, CH_3, Pbf), 1.78 (s, 3H, CH_3, cumyl), 1.77 (s, 3H, CH_3, cumyl), 1.76–1.70 (m, 1H, C*H*Hβ-Arg), 1.67–1.54 (m, 3H, CH_2γ-Arg, CH*H*β-Arg), 1.45 (s, 6H, 2 × CH_3, Pbf); **^{13}C NMR** (101 MHz, $CDCl_3$) δ_C ppm 173.9 (C=O), 158.9 (C), 156.1 (C=NH), 144.7 (C), 138.5 (C), 133.1 (C), 132.4 (C), 128.6 (CH), 127.7 (CH), 124.8 (C), 124.5 (CH), 117.6 (C), 86.5 (C, Pbf), 83.4 (C, cumyl), 78.9 (CH_2GOx or OCH_2), 78.8 (CH_2GOx or OCH_2), 78.7 (OCH_2), 59.8 (C, Ox), 56.0 (CH, α-Arg), 43.4 (CH_2, Pbf), 41.1 (CH_2, δ-Arg), 31.5 (CH_2, β-Arg), 28.7 (CH_3, 2 × Pbf), 28.5 (CH_3, cumyl), 28.0 (CH_3, cumyl), 25.5 (CH_2, γ-Arg), 19.4 (CH_3, Pbf), 18.1 (CH_3, Pbf), 12.6 (CH_3, Pbf); $\mathbf{\nu_{max}}$ (neat) = 3323, 2944, 1727, 1549, 1245, 1100, 970 cm^{-1}; **MS** (ESI^+) *m/z* 660 $[M+H]^+$, 682 $[M+Na]^+$; **HRMS** (ESI^+) calcd. for $C_{32}H_{45}N_5NaO_8S$ $[M+Na]^+$ 682.2881, found 682.2883; $[\boldsymbol{\alpha}]_D^{27}$ +1.1 (*c* 1.0, $CHCl_3$).

Fmoc-GOx-Arg(Pbf)-OCumyl (125)

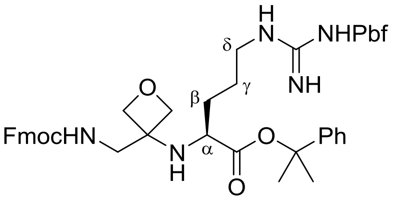

125

To a solution of NO_2-GOx-Arg(Pbf)-OCumyl (**124**) (1.41 g, 2.14 mmol, 1.0 equiv) in THF (22 mL) was added Fmoc *N*-hydroxysuccinimide ester (1.44 g, 4.28 mmol, 2.0 equiv), $NaHCO_3$ (719 mg, 8.56 mmol, 4.0 equiv) and Raney Ni (slurry in H_2O, 2.2 mL). The reaction mixture was placed under an atmosphere of nitrogen, evacuated and filled with hydrogen (balloon). The reaction mixture was stirred vigorously for 2.5 h at room temperature, filtered through a plug of Celite eluting with EtOAc, and the filtrate was concentrated under reduced pressure. Fmoc-GOx-Arg(Pbf)-OCumyl (**125**) was afforded after purification by column chromatography (SiO_2, PE/EtOAc 1:1→EtOAc) as a white foam (1.29 g, 1.51 mmol, 71%). $\mathbf{R_f}$ (EtOAc) 0.30; **mp** 72–75 °C; **^{1}H NMR** (600 MHz, $CDCl_3$ @ 323 K) δ_H ppm 7.75 (d, *J* = 7.6 Hz, 2H, ArH), 7.56

(t, J = 6.8 Hz, 2H, ArH), 7.38 (t, J = 7.4 Hz, 2H, ArH), 7.34 (d, J = 7.6 Hz, 2H, ArH), 7.33–7.26 (m, 4H, ArH), 7.23 (d, J = 7.2 Hz, 1H, ArH), 6.09 (br. s, 2H, NH), 6.03 (br. s, 1H, NH), 5.37 (br. s, 1H, NH), 4.43–4.32 (m, 3H, CH_2-Fmoc, OC*H*H-Ox), 4.31–4.25 (m, 3H, OCH_2-Ox, OCH*H*-Ox), 4.17 (t, J = 7.6 Hz, 1H, CH-Fmoc), 3.64 (br. m, 1H, C*H*HGOx), 3.33 (m, 1H, CHα-Arg), 3.30 (dd, J = 14.0, 4.9 Hz, 1H, CH*H*GOx), 3.23–3.13 (m, 2H, CH_2δ-Arg), 2.92 (s, 2H, CH_2, Pbf), 2.60 (s, 3H, CH_3, Pbf), 2.53 (s, 3H, CH_3, Pbf), 2.11 (br. s, 1H, NH), 2.09 (s, 3H, CH_3, Pbf), 1.79 (s, 3H, CH_3, cumyl), 1.78–1.73 (m, 1H, C*H*Hβ-Arg) 1.77 (s, 3H, CH_3, cumyl), 1.65–1.59 (m, 2H, CH_2γ-Arg), 1.58–1.50 (m, 1H, CH*H*β-Arg), 1.44 (s, 6H, 2 × CH_3, Pbf); **^{13}C NMR** (151 MHz, $CDCl_3$ @ 323 K) δ_C ppm 174.7 (C=O), 158.9 (C), 157.2 (C=O, Fmoc), 156.3 (C=NH), 144.9 (C), 144.0 (C), 141.5 (C), 138.5 (C), 133.5 (C), 132.5 (C), 128.5 (CH), 127.9 (CH), 127.6 (CH), 127.2 (CH), 125.2 (CH), 124.7 (C), 124.6 (CH), 120.1 (CH), 117.6 (C), 86.5 (C, Pbf), 83.2 (C, cumyl), 79.6 (OCH_2), 79.1 (OCH_2), 67.2 (CH_2, Fmoc), 60.2 (C, Ox), 56.2 (CH, α-Arg), 47.5 (CH, Fmoc), 46.1 (CH_2, GOx), 43.5 (CH_2, Pbf), 41.2 (CH_2, δ-Arg), 31.7 (CH_2, β-Arg), 28.7 (CH_3, 2 × Pbf), 28.6 (CH_3, cumyl), 28.1 (CH_3, cumyl), 25.9 (CH_2, γ-Arg), 19.3 (CH_3, Pbf), 18.0 (CH_3, Pbf), 12.5 (CH_3, Pbf); **ν_{max}** (neat) = 3334, 2934, 1717, 1548, 1244, 1100, 970 cm^{-1}; **MS** (ESI^+) *m/z* 852 $[M+H]^+$, 874 $[M+Na]^+$; **HRMS** (ESI^+) calcd. for $C_{47}H_{57}N_5NaO_8S$ $[M+Na]^+$ 874.3820, found 874.3827; $[\boldsymbol{\alpha}]_D^{28}$ +8.6 (*c* 1.30, $CHCl_3$).

Fmoc-GOx-Arg(Pbf)-Cys(Trt)-O*t*Bu (126)

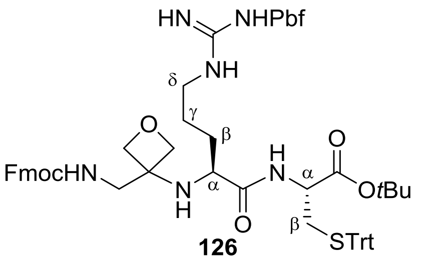

126

Fmoc-GOx-Arg(Pbf)-OCumyl (**125**) (256 mg, 0.30 mmol, 1.0 equiv) was dissolved in 2% TFA/CH_2Cl_2 (0.05 M) and stirred at room temperature for 2 h following a procedure from Beadle *et al.*[8] The reaction mixture was concentrated under reduced pressure, and the resulting residue was repeatedly re-suspended in CH_2Cl_2 (3 × 15 mL) and the solvent removed under reduced pressure. Meanwhile, diethylamine (2.0 mL) was added to a solution of Fmoc-Cys(Trt)-O*t*Bu (**128**) (298 mg, 0.45 mmol, 1.5 equiv) in CH_2Cl_2 (2.0 mL) and the reaction mixture was stirred at room temperature for 1 h. The mixture was concentrated under reduced pressure and the resulting residue repeatedly dissolved in CH_2Cl_2 (3 × 15 mL) and concentrated under reduced pressure to give the crude amine. The crude Fmoc-GOx-Arg(Pbf)-OH was dissolved in DMF (5.0 mL) and HATU (125 mg, 0.33 mmol, 1.1 equiv), diisopropylethyl-amine (204 µL, 1.20 mmol, 4.0 equiv) and the crude amine in DMF (2.0 mL) were added successively. The reaction mixture was stirred at room temperature for 48 h and the solvent removed under reduced pressure. The residue was purified by flash column chromatography (SiO_2, PE/EtOAc 1:1→5% MeOH in CH_2Cl_2) to give tripeptide **126** (268 mg, 0.24 mmol, 79%) as a white foam; **R_f** (5% MeOH in CH_2Cl_2) 0.51; **mp** 175–178 °C; **^{1}H NMR** (500 MHz, $CDCl_3$ @ 323 K) δ_H ppm 7.75 (d, J = 7.6 Hz, 2H, ArH), 7.58 (d, J = 7.5 Hz, 2H, ArH), 7.40–7.34 (m, 9H, ArH, NH), 7.30–7.22 (m, 8H, ArH), 7.19 (t, J = 7.2 Hz, 3H, ArH), 6.02 (br. s, 2H, NH), 5.88 (br. s, 1H, NH), 4.53–4.34 (m, 6H, C*H*H-Fmoc, 2 × OCH_2-Ox, CHα-Cys), 4.32-4.25 (m, 1H, CH*H*-Fmoc), 4.15 (t, J = 6.6 Hz, 1H, CH-Fmoc), 3.75 (dd, J = 14.4, 7.4 Hz, 1H, C*H*HGOx), 3.35 (dd, J = 14.4, 4.2 Hz, 1H, CH*H*GOx), 3.33–3.27 (m, 1H, CHα-Arg), 3.27–3.21 (m, 1H, C*H*Hδ-Arg), 3.18–3.10 (m, 1H, CH*H*δ-Arg), 2.92 (s, 2H, CH_2, Pbf), 2.68 (dd, J = 12.3, 7.3 Hz, 1H, C*H*Hβ-Cys), 2.58 (s, 3H, CH_3, Pbf), 2.52 (s, 3H, CH_3, Pbf), 2.48 (dd, J = 12.3, 4.2 Hz, 1H, CH*H*β-Cys), 2.11 (br. s, 1H, NH), 2.08 (s, 3H, CH_3, Pbf), 1.75–1.52 (m, 4H, CH_2β-Arg, CH_2γ-Arg), 1.44 (s, 6H, 2 × CH_3, Pbf), 1.39 (s, 9H, 3 × CH_3, *t*Bu); **^{13}C NMR** (151 MHz, $CDCl_3$ @ 323 K) δ_C ppm 174.8 (C=O), 170.0 (C=O), 158.9 (C), 157.6 (C=O, Fmoc), 156.1 (C=NH), 144.3 (C), 144.1 (C), 144.0 (C), 141.4 (C), 138.6 (C), 133.1 (C), 132.5 (C), 129.6 (CH), 128.2 (CH), 127.9 (CH), 127.2 (CH), 127.1 (CH), 125.4 (CH), 125.3 (CH), 124.9 (CH), 124.7 (C), 120.1 (CH), 117.6 (C), 86.5 (C, Pbf), 83.2 (C, *t*Bu), 79.1 (OCH_2), 78.8 (OCH_2), 67.0 (CH_2, Fmoc), 66.8 (C, Trt), 60.7 (C, Ox), 56.1 (CH, α-Arg), 51.6 (CH, α-Cys), 47.2

(CH, Fmoc), 45.8 (CH_2, GOx), 43.4 (CH_2, Pbf), 40.8 (CH_2, δ-Arg), 34.1 (CH_2, β-Cys), 32.2 (CH_2, β-Arg), 28.7 (CH_3, 2 × Pbf), 28.0 (CH_3, *t*Bu), 25.3 (CH_2, γ-Arg), 19.4 (CH_3, Pbf), 18.1 (CH_3, Pbf), 12.6 (CH_3, Pbf); **ν_{max}** (neat) = 3326, 2924, 1722, 1546, 1240, 1151, 1104 cm^{-1}; **MS** (ESI^+) *m/z* 1135 $[M+H]^+$, 1157 $[M+Na]^+$; **HRMS** (ESI^+) calcd. for $C_{64}H_{75}N_6O_9S_2$ $[M+H]^+$ 1135.5031, found 1135.5022; $[\alpha]_D^{28}$ +18.2 (*c* 0.00033, $CHCl_3$).

Fmoc-Asn(Trt)-GOx-Arg(Pbf)-Cys(Trt)-O*t*Bu (127)

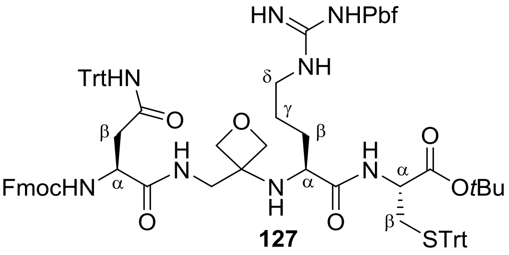

127

Diethylamine (2.0 mL) was added to a solution of Fmoc-GOx-Arg(Pbf)-Cys(Trt)-O*t*Bu (**126**) (262 mg, 0.23 mmol, 1.0 equiv) in CH_2Cl_2 (2.0 mL) and the reaction mixture was stirred at room temperature for 1 h. The mixture was concentrated *in vacuo* and the resulting residue repeatedly dissolved in CH_2Cl_2 (3 × 15 mL) and concentrated under reduced pressure to give the crude amine. HATU (132 mg, 0.35 mmol, 1.5 equiv), diisopropylethylamine (181 µL, 1.10 mmol, 3.0 equiv) and Fmoc-Asn(Trt)-OH (208 mg, 0.35 mmol, 1.5 equiv) were added to the crude amine in CH_2Cl_2 (5.0 mL). The reaction mixture was stirred at room temperature for 16 h and the solvent was removed *in vacuo*. The residue was purified by flash column chromatography (SiO_2, CH_2Cl_2→5% MeOH in CH_2Cl_2) to give tetrapeptide **127** (262 mg, 0.18 mmol, 78%) as a white foam; **R_f** (5% MeOH in CH_2Cl_2) 0.40; **mp** 179–183 °C; **^{1}H NMR** (500 MHz, $CDCl_3$ @ 323 K) δ_H ppm 7.74 (d, *J* = 7.7 Hz, 1H, ArH), 7.72 (d, *J* = 7.7 Hz, 1H, ArH), 7.53 (d, *J* = 7.4 Hz, 2H, ArH), 7.39–7.33 (m, 8H, ArH), 7.28–7.17 (m, 20H, ArH), 7.14 (d, *J* = 7.1 Hz, 6H, ArH), 7.05 (br. s, 1H, NH), 6.99 (br. s, 1H, NH), 6.18 (br. s, 1H, NH), 5.65 (br. s, 2H, NH), 4.51–4.41 (m, 2H, CHα-Asn, CH_2-Fmoc), 4.40–4.28 (m, 4H, CHα-Cys, OCH*H*-Ox, CH_2-Fmoc), 4.28–4.21 (m, 2H, OC*H*H-Ox), 4.20–4.12 (m, 2H, OCH*H*-Ox, CH-Fmoc), 3.61–3.50 (m, 2H, CH_2β-Asn), 3.15–3.10 (m, 1H, C*H*HGOx), 3.12-3.06 (m, 1H, CHα-Arg), 2.91 (s, 2H, CH_2, Pbf), 2.93–2.83 (m, 2H, CH_2δ-Arg), 2.72-2.63 (m, 2H, C*H*Hβ-Cys, CH*H*GOx), 2.55 (s, 3H, CH_3, Pbf), 2.48 (s, 3H, CH_3, Pbf), 2.46 (dd, *J* = 12.3, 4.4 Hz, 1H, CH*H*β-Cys), 2.06 (s, 3H, CH_3, Pbf), 1.51–1.33 (m, 4H, CH_2β-Arg, CH_2γ-Arg), 1.43 (s, 6H, 2 × CH_3, Pbf), 1.39 (s, 9H, 3 × CH_3, *t*Bu). *N.B.* Three NH signals cannot be detected; **^{13}C NMR** (126 MHz, $CDCl_3$ @ 323 K) δ_C ppm 174.9 (C=O), 171.6 (C=O), 170.7 (C=O), 169.6 (C=O), 158.7 (C), 156.5 (C=O, Fmoc), 156.0 (C=NH), 144.4 (C), 144.0 (C), 143.7 (C), 143.7 (C), 141.5 (C), 138.5 (C), 133.5 (C), 132.4 (C), 129.6 (CH), 128.7 (CH), 128.3 (CH), 128.2 (CH), 128.0 (CH), 127.5 (CH), 127.3 (CH), 127.0 (CH), 125.1 (CH), 124.6 (C),124.1 (CH), 120.23 (CH),120.21 (CH), 117.5 (CH), 86.4 (C, Pbf), 83.0 (C, *t*Bu), 79.5 (OCH_2), 78.9 (OCH_2), 71.1 (C, Trt), 67.3 (C, Trt), 66.9 (CH_2, Fmoc), 59.9 (C, Ox), 56.0 (CH, α-Arg), 52.0 (CH, α-Asn or α-Cys), 51.9 (CH, α-Asn or α-Cys), 47.3 (CH, Fmoc), 44.5 (CH_2, β-Asn), 43.4 (CH_2, Pbf), 40.7 (CH_2, δ-Arg), 38.5 (CH_2, GOx), 33.9 (CH_2, β-Cys), 31.9 (CH_2, β-Arg), 28.7 (CH_3, 2 × Pbf), 28.0 (CH_3, *t*Bu), 25.1 (CH_2, γ-Arg), 19.5 (CH_3, Pbf), 18.1 (CH_3, Pbf), 12.6 (CH_3, Pbf); **ν_{max}** (neat) = 3326, 2926, 1621, 1248, 1151, 1104, 839 cm^{-1}; **MS** (ESI^+) *m/z* 1492 $[M+H]^+$, 1514 $[M+Na]^+$; **HRMS** (ESI^+) calcd. for $C_{87}H_{94}N_8Na_2O_{11}S_2$ $[M+2Na]^{2+}$ 768.3134, found 768.3131; $[\alpha]_D^{28}$ +9.9 (*c* 0.00025, $CHCl_3$).

Fmoc-Cys(Trt)-Asn(Trt)-GOx-Arg(Pbf)-Cys(Trt)-O*t*Bu (2)

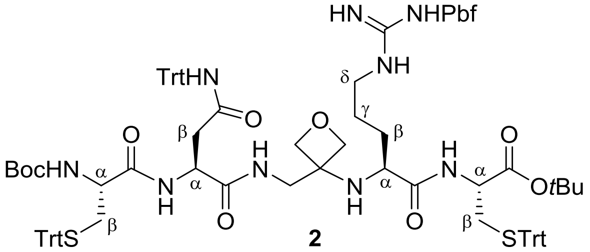

2

Diethylamine (1.0 mL) was added to a solution of Fmoc-GOx-Arg(Pbf)-Cys(Trt)-O*t*Bu (**127**) (200 mg, 0.14 mmol, 1.0 equiv) in CH_2Cl_2 (1.0 mL) and the reaction mixture was stirred at room temperature for 1 h. The reaction mixture was concentrated under reduced pressure and the resulting residue repeatedly dissolved in CH_2Cl_2 (3 × 15 mL) and concentrated under reduced pressure to give the crude amine. HATU (76 mg, 0.20 mmol, 1.4 equiv), diisopropylethylamine (105 µL, 0.60 mmol, 3.0 equiv) and Boc-Cys(Trt)-OH (94 mg, 0.20 mmol, 1.4 equiv) were added to the crude amine in CH_2Cl_2 (5.0 mL). The reaction mixture was stirred at room temperature for 16 h and the solvent removed under reduced pressure. The residue was purified by flash column chromatography (SiO_2, CH_2Cl_2 → 5% MeOH in CH_2Cl_2) to give pentapeptide **2** (191 mg, 0.11 mmol, 81%) as a white foam; **R_f** (5% MeOH in CH_2Cl_2) 0.37; **mp** 183–185 °C; **^{1}H NMR** (500 MHz, $CDCl_3$ @ 323 K) δ_H ppm 8.06 (s, 1H, NH), 7.56 (s, 1H, NH), 7.37 (d, *J* = 7.7 Hz, 17H, ArH), 7.30–7.11 (s, 28H, ArH), 6.85 (s, 1H, NH), 5.92 (s, 3H, NH), 4.60 (s, 1H, NH), 4.54 (s, 1H, CHα-Cys), 4.50 (d, *J* = 6.8 Hz, 1H, OC*H*H-Ox), 4.40 (d, *J* = 6.8 Hz, 1H, OCH*H*-Ox), 4.38–4.33 (m, 1H, CHα-Cys), 4.30 (d, *J* = 5.8 Hz, 1H, OC*H*H-Ox), 4.26 (d, *J* = 6.9 Hz, 1H, OCH*H*-Ox), 3.90 (dd, *J* = 11.9, 6.4 Hz, 1H, C*H*HGOx), 3.55–3.47 (m, 1H, CHα-Asn), 3.40–3.31 (m, 2H, CHα-Arg, CH*H*GOx), 3.14–3.02 (m, 3H, CH_2γ-Arg, C*H*Hβ-Cys), 2.93 (s, 2H, CH_2, Pbf), 2.66–2.60 (m, 3H, CH*H*β-Cys, CH_2β-Asn), 2.59 (s, 3H, CH_3, Pbf), 2.53 (s, 3H, CH_3, Pbf), 2.52–2.44 (m, 2H, 2 × CH*H*β-Cys), 2.08 (s, 3H, CH_3, Pbf), 1.44 (s, 6H, 2 × CH_3, Pbf), 1.40 (s, 9H, 3 × CH_3, *t*Bu), 1.25 (s, 9H, 3 × CH_3, *t*Bu). *N.B.* Two NH signals not observed; **^{13}C NMR** (126 MHz, $CDCl_3$ @ 323 K) δ_C ppm 174.4 (C=O), 173.4 (C=O), 171.4 (C=O), 171.2 (C=O), 170.1 (C=O), 169.3 (C=O), 158.50 (C), 156.0 (C=NH), 144.4 (C), 144.1 (C), 143.9 (C), 138.4 (C), 135.9 (C), 133.5 (C), 132.3 (C), 129.5 (CH), 129.4 (CH), 128.7 (CH), 128.3 (CH), 128.0 (CH), 127.3 (CH), 127.2 (CH), 126.8 (CH), 124.8 (CH), 124.4 (C), 117.3 (C), 86.2 (C, Pbf), 82.5 (C, *t*Bu), 81.3 (C, *t*Bu), 79.6 (OCH_2), 78.4 (OCH_2), 70.8 (C, Trt), 66.7 (C, Trt), 64.6 (C, Trt), 60.5 (C, Ox), 55.9 (CH, α-Arg), 54.5 (CH, α-Asn), 51.7 (CH, α-Cys), 50.6 (CH, α-Cys), 45.9 (CH_2, GOx), 43.3 (CH_2, Pbf), 40.6 (CH_2, δ-Arg), 36.8 (CH_2, β-Cys), 34.2 (CH_2, β-Cys), 32.9 (CH_2, β-Asn), 31.5 (CH_2, β-Arg), 28.6 (CH_3, 2 × Pbf), 28.2 (CH_3, *t*Bu), 27.9 (CH_3, *t*Bu), 25.0 (CH_2, γ-Arg), 19.3 (CH_3, Pbf), 17.9 (CH_3, Pbf), 12.5 (CH_3, Pbf); **ν_{max}** (neat) = 3317, 2971, 1669, 1445, 1250, 1153, 671 cm^{-1}; **MS** (ESI^+) *m/z* 1715 $[M+H]^+$, 1737 $[M+Na]^+$; **HRMS** (ESI^+) calcd. for $C_{99}H_{111}N_9Na_2O_{12}S_3$ $[M+2Na]^{2+}$ 879.8649, found 879.8653; $[\alpha]_D^{28}$ +10.9 (*c* 0.0004, $CHCl_3$).

Cyclo(H-Cys-Asn-GOx-Arg-Cys-OH) (22)

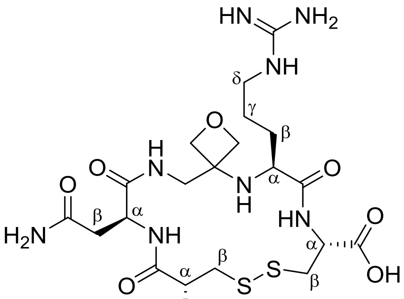

22

The fully protected pentapeptide **2** (66.5 mg, 0.04 mmol, 1.0 equiv) was dissolved in MeOH (2.0 mL) and slowly added to a solution of iodine (30 mg, 0.12 mmol, 3.0 equiv) in MeOH (2.0 mL). The mixture was stirred for 1 h at room temperature, cooled to 0 °C and saturated aqueous $Na_2S_2O_2$ was added until a nearly colourless solution was obtained. The mixture was concentrated *in vacuo* to a volume of ca. 0.5 mL, EtOAc (10 mL) was added, the solution was washed with 0.1 M aqueous $Na_2S_2O_2$ solution (5.0 mL), dried over Na_2SO_4 and filtered. The crude product was treated with 70% TFA/20% CH_2Cl_2/10% TIS under anhydrous conditions for 2.5 h at room temperature. The cleavage cocktail was removed under a steam of nitrogen and the crude peptide precipitated in cold diethyl ether.

After centrifugation, the peptide was dissolved in water and further purified by HPLC (0–3 min 3%, 3–10 min 25%, 10–15 min 100%, R_t = 7.32 min) to give the cyclic peptide **22** as a white solid (7.4 mg, 32% yield over two steps). **mp** 161–165 °C (decomposition); **^{1}H NMR** (500 MHz, D_2O @ 323 K) δ_H ppm 4.70 (m, 1H, CHα-Asn) 4.56–4.51 (m, 2H, OC*H*H-Ox, CHα-Cys), 4.45–4.36 (m, 3H, OCH_2-Ox, OCH*H*-Ox), 4.14 (t, *J* = 4.9 Hz, 1H, CHα-Cys), 3.98 (d, *J* = 14.6 Hz, 1H, C*H*HGOx), 3.64 (dd, *J* = 14.7, 5.5 Hz, 1H, C*H*Hβ-Cys), 3.45 (t, *J* = 6.2 Hz, 1H, CHα-Arg), 3.35 (dd, *J* = 14.7, 4.5 Hz, 1H, CH*H*β-Cys), 3.21 (d, *J* = 14.6 Hz, 2H, C*H*Hβ-Cys, CH*H*GOx), 3.12 (t, *J* = 6.6 Hz, 2H, CH_2δ-Arg), 2.81 (dd, *J* = 14.6, 10.6 Hz, 1H, CH*H*β-Cys), 2.78–2.68 (m, 2H, CH_2β-Asn), 1.70–1.61 (m, 2H, CH_2β-Arg), 1.59–1.49 (m, 2H, CH_2γ-Arg). *N.B.* CHα-Asn under water peak; **^{13}C NMR** (126 MHz, D_2O @ 323 K) δ_C ppm 176.4 (C=O), 176.1 (C=O), 174.4 (C=O), 172.7 (C=O), 170.2 (C=O), 156.7 (C=NH), 80.2 (OCH_2), 78.7 (OCH_2), 60.4 (C, Ox), 56.1 (CH, α-Arg), 54.3 (CH, α-Cys), 52.6 (CH, α-Cys), 50.8 (CH, α-Asn), 44.0 (CH_2 β-Cys), 43.6 (CH_2, GOx), 42.7 (CH_2 β-Cys), 40.7 (CH_2, δ-Arg), 36.2 (CH_2, β-Asn), 31.3 (CH_2, β-Arg), 24.2 (CH_2, γ-Arg); **ν_{max}** (neat) = 2943, 1660, 1409, 1285, 1170, 697, 466 cm^{-1}; **MS** (ESI^+) *m/z* 578 $[M+H]^+$, 600 $[M+Na]^+$; **HRMS** (ESI^+) calcd. for $C_{20}H_{36}N_9O_7S_2$ $[M+H]^+$ 578.2174, found 578.2178; $[\alpha]_D^{28}$ –81.3 (*c* 0.0004, DMF).

*2.16 Preparation of cyclic disulfide-bridged tripeptide **23***

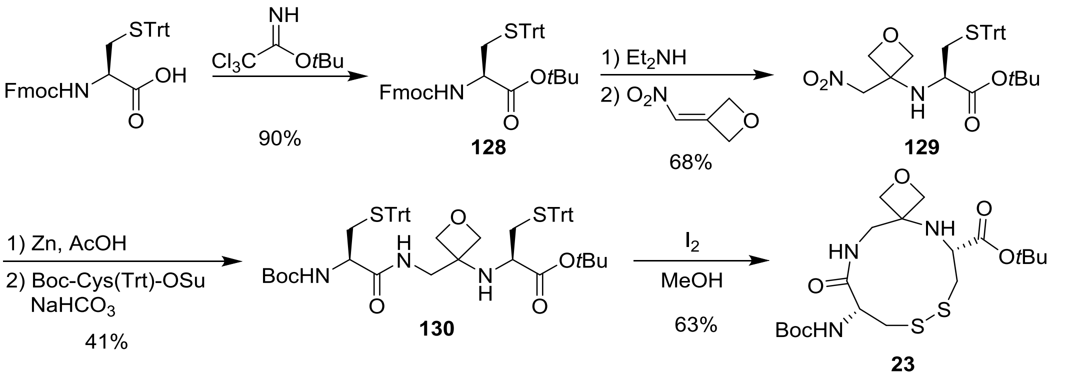

Fmoc-Cys(Trt)-O*t*Bu (128)

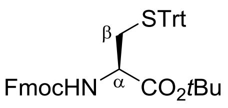

128

To a suspension of Fmoc-Cys(Trt)-OH (11.7 g, 20.0 mmol, 1.0 equiv) in anhydrous CH_2Cl_2 (160 mL) was added *tert*-butyl 2,2,2-trichloroacetimidate (8.74 g, 40.0 mmol, 2.0 equiv) and the mixture was stirred at ambient temperature for 3 d. The mixture was filtered through a pad of Celite and the solids were washed with EtOAc. The filtrate was concentrated *in vacuo* and the residue was purified by column chromatography (SiO_2, PE/EtOAc 5:1) to give **128** (11.6 g, 18.1 mmol, 90%) as a white solid. **R_f** (PE/EtOAc 5:1) 0.20; **mp** 71–72 °C; **^{1}H NMR** (400 MHz, $CDCl_3$) δ_H ppm 7.71 (d, *J* = 7.4 Hz, 2H, ArH), 7.56 (d, *J* = 7.3 Hz, 2H, ArH), 7.37–7.31 (m, 8H, ArH), 7.26–7.18 (m, 8H, ArH), 7.17–7.11 (m, 3H, ArH), 5.26 (d, *J* = 8.2 Hz, 1H, NH), 4.29 (d, *J* = 7.2 Hz, 2H, CH_2-Fmoc), 4.26–4.21 (m, 1H, CHα-Cys), 4.21 (t, *J* = 7.2 Hz, 1H, CH-Fmoc), 2.57 (dd, *J* = 12.1, 5.8 Hz, 1H, C*H*Hβ-Cys), 2.49 (dd, *J* = 12.1, 4.4 Hz, 1H, CH*H*β-Cys), 1.38 (s, 9H, 3 × CH_3, *t*Bu); **^{13}C NMR** (101 MHz, $CDCl_3$) δ_C ppm 169.7 (C=O), 155.7 (C=O, Fmoc), 144.5 (C), 144.1 (C), 144.0 (C), 141.4 (C), 137.0 (C), 129.7 (CH), 128.1 (CH), 127.8 (CH), 127.2 (CH), 127.0 (CH), 125.3 (CH), 120.1 (CH), 82.8 (C, *t*Bu), 67.24 (CH_2, Fmoc), 67.19 (CH_2, CS), 53.5 (CH, α-Cys), 47.3 (CH, Fmoc), 34.6 (CH_2, β-Cys), 28.1 (CH_3, *t*Bu); **ν_{max}** (neat) = 1710, 1492, 1446, 1244, 1150, 1035, 739, 698 cm^{-1}; **MS** (ESI^+) *m/z* 664 $[M+Na]^+$; **HRMS** (ESI^+) calcd. for $C_{41}H_{39}NNaO_4S$ $[M+Na]^+$ 664.2492, found 664.2490; $[\alpha]_D^{28}$ +13.6 (*c* 1.0, $CHCl_3$). Lit. $[\alpha]_D^{20}$ +10.0 (*c* 1.0, $CHCl_3$).[9]

NO_2-GOx-Cys(Trt)-O*t*Bu (129)

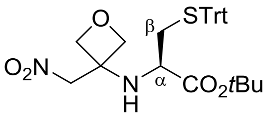

129

To a solution of Fmoc-Cys(Trt)-O*t*Bu (**128**) (3.85 g, 6.00 mmol, 1.0 equiv) in CH_2Cl_2 (6.0 mL) was added diethylamine (6.0 mL) and the mixture was stirred at room temperature for 1 h. The reaction mixture was concentrated under reduced pressure and the resulting residue repeatedly dissolved in CH_2Cl_2 (3 × 20 mL) and concentrated under reduced pressure to give the crude amine. In a second reaction vessel, oxetane-3-one (770 μL, 12.0 mmol, 2.0 equiv), nitromethane (910 μL, 16.8 mmol, 2.8 equiv) and triethylamine (335 μL, 2.40 mmol, 0.4 equiv) were combined at 0 °C and stirred for 1 h at room temperature. The mixture was dissolved in anhydrous CH_2Cl_2 (40 mL), cooled to –78 °C, and triethylamine (3.35 mL, 24.0 mmol, 4.0 equiv) was added followed by dropwise addition of a solution of methanesulfonyl chloride (930 μL, 12.0 mmol, 2.0 equiv) in anhydrous CH_2Cl_2 (12 mL). The reaction mixture was stirred at –78 °C for 1.5 h and a solution of the crude amine in anhydrous CH_2Cl_2 (20 mL) was added slowly *via* syringe. The reaction mixture was allowed to warm to room temperature and stirred for 16 h. A saturated solution of NH_4Cl (50 mL) was added and stirred for 10 min. The layers were separated and the aqueous one extracted with CH_2Cl_2 (2 × 40 mL) and EtOAc (2 × 40 mL). The combined organic phases were washed with saturated aqueous $NaHCO_3$ solution (30 mL), brine (30 mL), dried over $MgSO_4$, filtered, and concentrated under reduced pressure. The residue was purified by column chromatography (SiO_2, PE/EtOAc 4:1→2:2→1:1) to give **129** (2.18 g, 4.08 mmol, 68%) as an orange foam. **R_f** (PE/EtOAc 1:1) 0.55; **^{1}H NMR** (400 MHz, $CDCl_3$) δ_H ppm 7.42 (d, *J* = 7.6 Hz, 6H, ArH), 7.30 (t, *J* = 7.5 Hz, 6H, ArH), 7.22 (t, *J* = 7.2 Hz, 3H, ArH), 4.69 (s, 2H, CH_2GOx), 4.55 (d, *J* = 7.1 Hz, 1H, OC*H*H-Ox), 4.44 (d, *J* = 7.1 Hz, 1H, OC*H*H-Ox), 4.41 (d, *J* = 7.1 Hz, 1H, OCH*H*-Ox), 4.38 (d, *J* = 7.1 Hz, 1H, OCH*H*-Ox), 3.03 (q, *J* = 7.4 Hz, CHα-Cys), 2.42 (app. d, *J* = 6.6 Hz, 3H, CH_2β-Cys, NH), 1.39 (s, 9H, 3 × CH_3, *t*Bu); **^{13}C NMR** (101 MHz, $CDCl_3$) δ_C ppm 172.7 (C=O), 144.7 (C), 129.7 (CH), 128.1 (CH), 126.9 (CH), 82.6 (C, *t*Bu), 79.0 (CH_2, GOx), 78.7 (OCH_2), 78.4 (OCH_2), 67.1 (CS), 59.2 (C, Ox), 56.5 (CH, α-Cys), 36.3 (CH_2, β-Cys), 28.0 (CH_3, *t*Bu); **ν_{max}** (neat) = 1723, 1554, 1488, 1444, 1369, 1251, 1148, 981, 840, 741, 698 cm^{-1}; **MS** (ESI^+) *m/z* 557 $[M+Na]^+$; **HRMS** (ESI^+) calcd. for $C_{30}H_{34}N_2NaO_5S$ $[M+Na]^+$ 557.2081, found 557.2085; $[\boldsymbol{\alpha}]_D^{28}$ +10.6 (*c* 2.17, $CHCl_3$).

Boc-Cys(Trt)-GOx-Cys(Trt)-O*t*Bu (130)

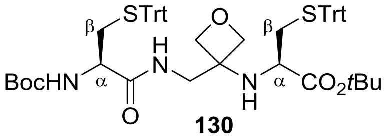

130

To a solution of **129** (534 mg, 1.00 mmol, 1.0 equiv) in THF (20 mL) was added zinc powder (196 mg, 3.00 mmol, 3.0 equiv) and acetic acid (458 μL, 8.00 mmol, 8.0 equiv) and the reaction mixture was vigorously stirred with a glass-coated magnetic stir bar at room temperature for 1 h. Additional zinc powder (196 mg, 3.00 mmol, 3.0 equiv) and acetic acid (458 μL, 8.00 mmol, 8.0 equiv) were added and the mixture was stirred at ambient temperature for 1 h (repeat 3×). The mixture was cooled to 0 °C and saturated aqueous $NaHCO_3$ solution (20 mL) was added followed by Boc-Cys-OSu (841 mg, 1.50 mmol, 1.5 equiv) and the solution was stirred for 16 h at room temperature. Brine (20 mL) was added and the mixture as extracted with EtOAc (3 × 15 mL). The combined organic layers were washed with saturated aqueous $NaHCO_3$ solution (30 mL) and brine (30 mL), dried over $MgSO_4$, filtered and concentrated *in vacuo*. Purification by column chromatography (SiO_2, PE/EtOAc 2:1→1:1) gave tripeptide **130** (391 mg, 0.41 mmol, 41%) as a white foam. **R_f** (PE/EtOAc 1:1) 0.36; **mp** 83–84 °C; **^{1}H NMR** (600 MHz, $CDCl_3$ @ 323 K) δ_H ppm 7.42 (d, *J* = 7.7 Hz, 6H, ArH), 7.39 (d, *J* = 7.7 Hz, 6H, ArH), 7.30–7.25 (m, 12H, ArH), 7.21 (t, *J* = 7.2 Hz, 6H, ArH), 6.67 (t, *J* = 5.1 Hz, 1H, NH), 4.82 (br. s, 1H, NH), 4.31 (d, *J* = 6.4 Hz, 1H, OC*H*H-Ox), 4.23 (d, *J* = 6.7 Hz, 1H, OC*H*H-Ox), 4.18 (d, *J* = 6.7 Hz, 1H, OCH*H*-Ox), 4.15 (d, *J* = 6.4 Hz, 1H, OCH*H*-Ox), 3.90 (dd, *J* = 12.8, 7.5 Hz, 1H, CHα-Cys), 3.59 (dd, *J* = 14.0, 6.4 Hz, 1H, C*H*HGOx), 3.37 (dd, *J* = 14.0, 4.4 Hz, 1H, CH*H*GOx), 2.70 (dd, *J* = 8.9, 4.6 Hz, 1H, CHα-Cys), 2.59 (dd, *J* = 12.4, 6.7 Hz, 1H, C*H*Hβ-Cys),

2.50 (dd, J = 12.6, 8.7 Hz, 1H, C*H*Hβ-Cys), 2.47 (dd, J = 12.4, 5.1 Hz, 1H, CH*H*β-Cys), 2.36 (dd, J = 12.6, 4.6 Hz, 1H, CH*H*β-Cys), 1.96 (br. s, 1H, NH), 1.44 (s, 9H, 3 × CH_3, *t*Bu), 1.36 (s, 9H, 3 × CH_3, *t*Bu); **^{13}C NMR** (151 MHz, $CDCl_3$ @ 323 K) δ_C ppm 173.6 (C=O), 171.0 (C=O), 155.1 (C=O, Boc), 144.8 (C), 144.7 (C), 129.79 (CH), 129.78 (CH), 128.17 (CH), 128.15 (CH), 127.03 (CH), 126.98 (CH), 82.2 (C, *t*Bu), 80.3 (OCH_2), 80.2 (C, Boc), 79.8 (OCH_2), 67.6 (CS), 67.2 (CS), 59.5 (C, Ox), 56.4 (CH, α-Cys), 54.2 (CH, α-Cys), 42.8 (CH_2, GOx), 36.6 (CH_2, β-Cys), 34.5 (CH_2, β-Cys), 28.5 (CH_3, *t*Bu), 28.0 (CH_3, Boc); **ν_{max}** (neat) = 1718, 1673, 1486, 1366, 1249, 975, 742, 698 cm^{-1}; **MS** (ESI$^+$) *m/z* 950 $[M+H]^+$, 974 $[M+Na]^+$; **HRMS** (ESI$^+$) calcd. for $C_{57}H_{63}N_3NaO_6S_2$ $[M+Na]^+$ 972.4050, found 972.4048; $[\boldsymbol{\alpha}]_D^{28}$ +45.3 (*c* 1.0, $CHCl_3$).

Cyclo(Boc-Cys-GOx-Cys-O*t*Bu) (23)

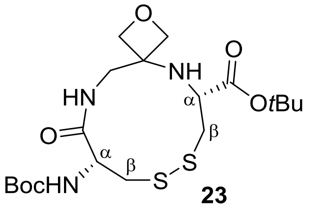

To a solution of iodine (305 mg, 1.20 mmol, 3.0 equiv) in anhydrous MeOH (40 mL) was slowly added a solution of **130** (380 mg, 0.40 mmol, 1.0 equiv) in anhydrous MeOH (40 mL). The mixture was stirred for 1 h at room temperature, cooled to 0 °C and a saturated aqueous solution of $Na_2S_2O_2$ was added until a nearly colourless solution was obtained. The mixture was concentrated *in vacuo* to a volume of ca. 5 mL, EtOAc (25 mL) was added, the solution was washed with 0.1 M aqueous $Na_2S_2O_2$ solution (10 mL), dried over Na_2SO_4 and filtered. The solvent was removed *in vacuo* and the residue was purified by column chromatography (SiO_2, PE/EtOAc 1:1→ EtOAc) to afford cyclic tripeptide **23** (1st run: 119 mg, 0.26 mmol, 64%; 2nd run (0.22 mmol scale): 62 mg, 0.13 mmol, 61%) as a white foam. **R_f** (EtOAc) 0.55; **mp** 98–100 °C; **^{1}H NMR** (600 MHz, $CDCl_3$ @ 323 K) δ_H ppm 6.58–6.54 (m, 1H, NH), 5.43 (d, J = 4.2 Hz, 1H, NH), 4.59 (d, J = 6.5 Hz, 1H, OC*H*H-Ox), 4.48 (d, J = 6.5 Hz, 1H, OC*H*H-Ox), 4.43 (d, J = 6.5 Hz, 2H, 2 × OCH*H*-Ox), 4.27 (t, J = 7.2 Hz, 1H, CHα-Cys), 4.04 (dd, J = 14.0, 6.8 Hz, 1H, C*H*HGOx), 3.69 (dd, J = 14.0, 4.5 Hz, 1H, CH*H*GOx), 3.63 (t, J = 5.8 Hz, 1H, CHα-Cys), 3.39 (d, J = 13.6 Hz, 1H, C*H*Hβ-Cys), 3.20 (br. m, 1H, CH*H*β-Cys), 2.94 (dd, J = 14.0, 5.1 Hz, 1H, C*H*Hβ-Cys), 2.82 (dd, J = 14.0, 5.6 Hz, 1H, CH*H*β-Cys), 2.45 (br. s, 1H, NH), 1.48 (s, 9H, 3 × CH_3, *t*Bu), 1.45 (s, 9H, 3 × CH_3, *t*Bu); **^{13}C NMR** (151 MHz, $CDCl_3$ @ 323 K) δ_C ppm 172.7 (C=O), 171.5 (C=O), 155.1 (C=O, Boc), 82.8 (C, *t*Bu), 82.3 (OCH_2), 80.9 (C, Boc), 79.8 (OCH_2), 60.0 (C, Ox), 57.1 (CH, α-Cys), 55.7 (CH, α-Cys), 46.4 (CH_2, GOx), 44.9 (CH_2, β-Cys), 28.5 (CH_3, *t*Bu), 28.2 (CH_3, *t*Bu). *N.B.* One carbon signal for CH_2, β-Cys not visible; **ν_{max}** (neat) = 3306, 2931, 1714, 1657, 1490, 1366, 1247, 1149, 971, 843, 751 cm^{-1}; **MS** (ESI$^+$) *m/z* 464 $[M+H]^+$, 486 $[M+Na]^+$; **HRMS** (ESI$^+$) calcd. for $C_{19}H_{33}N_3NaO_6S_2$ $[M+Na]^+$ 486.1703, found 486.1705; $[\boldsymbol{\alpha}]_D^{28}$ +81.4 (*c* 1.04, $CHCl_3$).

*2.17 Preparation of cyclic disulfide-bridged tetrapeptide **24***

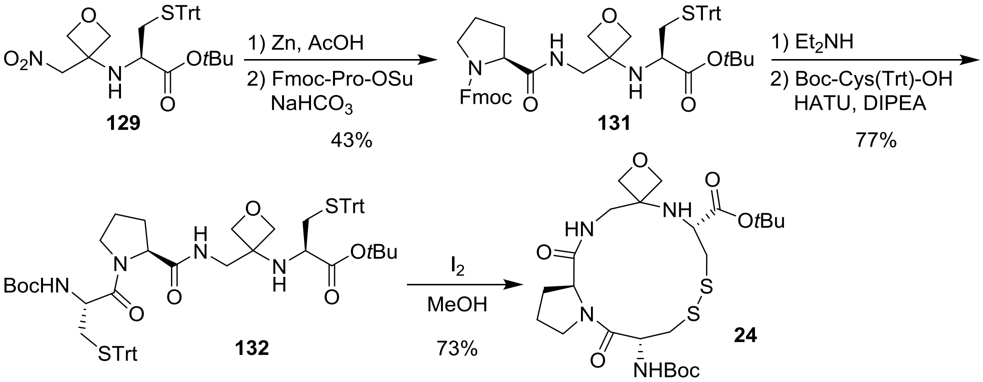

Fmoc-Pro-GOx-Cys(Trt)-O*t*Bu (131)

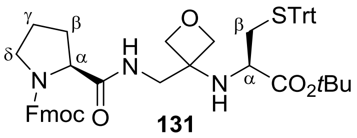

131

To a solution of **129** (532 mg, 1.00 mmol, 1.0 equiv) in THF (20 mL) was added zinc powder (196 mg, 3.00 mmol, 3.0 equiv) and acetic acid (458 µL, 8.00 mmol, 8.0 equiv) and the reaction mixture was vigorously stirred with a glass-coated magnetic stir bar at room temperature for 1 h. Additional zinc powder (196 mg, 3.00 mmol, 3.0 equiv) and acetic acid (458 µL, 8.00 mmol, 8.0 equiv) were added and the mixture was stirred at ambient temperature for 1 h (repeat 3×). The mixture was cooled to 0 °C and saturated aqueous $NaHCO_3$ solution (20 mL) was added followed by Fmoc-Pro-OSu (652 mg, 1.50 mmol, 1.5 equiv) and the solution was stirred for 16 h at room temperature. Brine (20 mL) was added and the mixture as extracted with EtOAc (3 × 15 mL). The combined organic layers were washed with saturated aqueous $NaHCO_3$ solution (30 mL) and brine (30 mL), dried over $MgSO_4$, filtered and concentrated *in vacuo*. Purification by column chromatography (SiO_2, PE/EtOAc 1:1→EtOAc) gave tripeptide **131** (352 mg, 0.43 mmol, 43%) as a white foam. $\mathbf{R_f}$ (EtOAc) 0.54; **mp** 83–86 °C; **^{1}H NMR** (600 MHz, toluene-*d8* @ 353 K) 7.55 (d, *J* = 7.4 Hz, 2H, ArH), 7.52–7.46 (m, 6H, ArH, NH), 7.09–7.05 (m, 8H), 6.99–6.94 (m, 8H), 6.73 (br. s, 1H, NH), 4.40 (dd, *J* = 10.6, 6.7 Hz, 1H, C*H*H-Fmoc), 4.35 (dd, *J* = 10.6, 6.9 Hz, 1H, CH*H*-Fmoc), 4.29 (d, *J* = 6.3 Hz, 1H, OC*H*H-Ox), 4.17–4.12 (m, 3H, OCH_2-Ox, OCH*H*-Ox), 4.10 (dd, *J* = 8.2, 2.8 Hz, 1H, CHα-Pro), 4.06 (t, *J* = 6.7 Hz, 1H, CH-Fmoc), 3.56 (dd, *J* = 13.8, 5.7 Hz, 1H, C*H*HGOx), 3.42 (dd, *J* = 13.8, 5.6 Hz, 1H, CH*H*GOx), 3.39–3.30 (m, 1H, C*H*Hδ-Pro), 3.24–3.16 (m, 1H, CH*H*δ-Pro), 3.03 (t, *J* = 6.6 Hz, 1H, CHα-Cys), 2.50 (d, *J* = 6.6 Hz, 2H, CH_2β-Cys), 1.73–1.67 (m, 1H, C*H*Hγ-Pro), 1.64–1.56 (m, 1H, C*H*Hβ-Pro), 1.38–1.35 (m, 1H, CH*H*γ-Pro), 1.34–1.27 (m, 1H, CH*H*β-Pro), 1.22 (s, 9H, 3 × CH_3, *t*Bu); **^{13}C NMR** (151 MHz, toluene-*d8* @ 353 K) δ_C ppm 173.8 (C=O), 172.3 (C=O), 145.6 (C), 145.0 (C), 144.9 (C), 142.09 (C), 142.06 (C), 130.3 (CH), 128.3 (CH), 127.5 (CH), 127.1 (CH), 125.6 (CH), 125.5 (CH), 120.3 (CH), 81.7 (C, *t*Bu), 79.6 (2 × OCH_2), 67.9 (CS), 67.8 (CH_2-Fmoc), 61.5 (CH, α-Pro), 60.5 (C, Ox), 56.9 (CH, α-Cys), 48.3 (CH-Fmoc), 47.4 (CH_2, δ-Pro), 44.6 (CH_2, GOx), 37.4 (CH_2, β-Cys), 28.0 (*t*Bu), 24.6 (CH_2, γ-Pro). *N.B.* Fmoc carbonyl carbon signal and CH, β-Pro not visible; $\mathbf{\nu_{max}}$ (neat) = 3310, 2978, 1699, 1444, 1413, 1350, 1147, 1178, 978, 740, 700 cm^{-1}; **MS** (ESI$^+$) *m/z* 824 $[M+H]^+$, 846 $[M+Na]^+$; **HRMS** (ESI$^+$) calcd. for $C_{50}H_{53}N_3NaO_6S$ $[M+Na]^+$ 846.3547, found 846.3552; $[\boldsymbol{\alpha}]_D^{29}$ +31.3 (*c* 0.14, $CHCl_3$).

Boc-Cys(Trt)-Pro-GOx-Cys(Trt)-O*t*Bu (132)

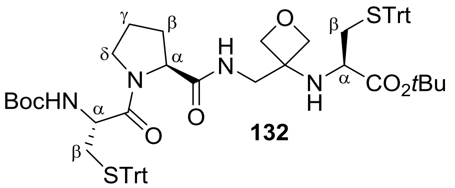

132

To a solution of tripeptide **131** (320 mg, 0.39 mmol, 1.0 equiv) in CH_2Cl_2 (2.0 mL) was added diethylamine (0.4 mL) and the mixture was stirred at room temperature for 1 h. The reaction mixture was concentrated under reduced pressure and the resulting residue repeatedly dissolved in CH_2Cl_2 (3 × 10 mL) and concentrated under reduced pressure to give the crude amine. The residue was dissolved in CH_2Cl_2 (8.0 mL), Boc-Cys(Trt)-OH (216 mg, 0.47 mmol, 1.2 equiv), HATU (177 mg, 0.47 mmol, 1.2 equiv) and DIPEA (204 µL, 1.17 mmol, 3.0 equiv) were added subsequently, and the mixture was stirred at room temperature for 16 h. The reaction mixture was diluted with CH_2Cl_2 (20 mL) and washed with 10% citric acid solution (20 mL) and saturated $NaHCO_3$ solution (20 mL), dried over $MgSO_4$, filtered and concentrated under reduced pressure. The residue was purified by flash column chromatography (SiO_2, PE/EtOAc 1:1→EtOAc) to give tetrapeptide **132** (313 mg, 0.30 mmol, 77%) as a white foam. $\mathbf{R_f}$ (EtOAc) 0.56; **mp** 92–95 °C; NMR data reported for the major rotamer: **^{1}H NMR** (600 MHz, $CDCl_3$) δ_H ppm 7.43–7.36 (m, 12H, ArH), 7.31–7.27 (m, 12H, ArH), 7.21 (t, *J* = 7.2 Hz, 6H, ArH, NH), 6.82 (t, *J* = 5.2 Hz, 1H, NH), 4.98 (d, *J* = 8.8 Hz, 1H, NH), 4.29–4.20 (m, 3H, OC*H*H-Ox, CHα-Cys, CHα-Pro), 4.12 (d, *J* = 6.7 Hz, 1H, OC*H*H-Ox), 4.08 (d,

J = 6.7 Hz, 1H, OCH*H*-Ox), 4.03 (d, J = 6.5 Hz, 1H, OCH*H*-Ox), 3.48 (dd, J = 13.8, 6.4 Hz, 1H, C*H*HGOx), 3.35 (dd, J = 16.5, 7.8 Hz, 1H, C*H*Hδ-Pro), 3.29 (dd, J = 13.8, 4.3 Hz, 1H, CH*H*GOx), 3.07–3.02 (m, 1H CH*H*δ-Pro), 2.79 (dd, J = 8.8, 4.6 Hz, 1H, CH, α-Cys), 2.60 (dd, J = 12.8, 5.4 Hz, 1H, C*H*Hβ-Cys), 2.43 (dd, J = 12.8, 8.5 Hz, 2H, CH_2β-Cys), 2.27 (dd, J = 12.8, 4.6 Hz, 1H, CH*H*β-Cys), 2.09–2.03 (m, 1H, C*H*Hβ-Pro), 2.02 (br. s, 1H, NH), 1.90–1.82 (m, 1H, CH*H*β-Pro), 1.82–1.75 (m, 1H, C*H*Hγ-Pro), 1.72–1.64 (m, 1H, CH*H*γ-Pro), 1.41 (s, 9H, 3 × CH_3, *t*Bu), 1.35 (s, 9H, 3 × CH_3, *t*Bu); **^{13}C NMR** (126 MHz, $CDCl_3$) δ_C ppm 173.5 (C=O), 171.7 (C=O), 170.4 (C=O), 155.1 (C=O, Boc), 144.6 (C), 129.80 (CH), 129.79 (CH), 128.2 (CH), 128.2 (CH), 127.0 (CH), 127.0 (CH), 82.0 (C, *t*Bu), 80.0 (OCH_2), 79.6 (OCH_2), 79.4 (C, Boc), 67.5 (CS), 67.4 (CS), 60.6 (CH, α-Cys), 59.3 (C, Ox), 56.2 (CH, α-Cys), 51.4 (CH, α-Pro), 47.3 (CH_2, δ-Pro), 42.8 (CH_2, GOx), 36.5 (CH_2, β-Cys), 34.5 (CH_2, β-Cys), 28.5 (CH_3, *t*Bu), 28.4 (CH_2, β-Pro) 28.0 (CH_3, *t*Bu), 24.9 (CH_2, γ-Pro); **ν_{max}** (neat) = 3339, 2979, 1711, 1651, 1488, 1443, 1366, 1149, 741, 698 cm^{-1}; **MS** (ESI$^+$) *m/z* 1047 [M+H]$^+$, 1069 [M+Na]$^+$; **HRMS** (ESI$^+$) calcd. for $C_{62}H_{70}N_4NaO_7S_2$ [M+Na]$^+$ 1069.4578, found 1069.4583; $[\alpha]_D^{29}$ +25.1 (*c* 0.88, $CHCl_3$).

Cyclo(Boc-Cys-Pro-GOx-Cys-O*t*Bu) (24)

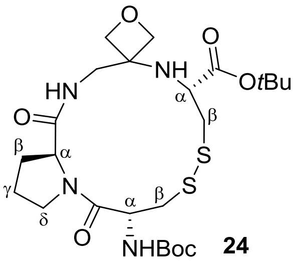

24

To a solution of iodine (76 mg, 0.30 mmol, 3.0 equiv) in anhydrous MeOH (10 mL) was slowly added a solution of Boc-Cys(Trt)-Pro-GOx-Cys(Trt)-O*t*Bu (**132**) (105 mg, 0.10 mmol, 1.0 equiv) in anhydrous MeOH (10 mL). The mixture was stirred for 1 h at room temperature, cooled to 0 °C and a saturated aqueous solution of $Na_2S_2O_2$ was added until a nearly colourless solution was obtained. The mixture was concentrated under reduced pressure to a volume of ca. 2 mL, EtOAc (25 mL) was added, the solution was washed with 0.1 M aqueous $Na_2S_2O_2$ solution (10 mL), dried over Na_2SO_4 and filtered. The solvent was removed *in vacuo* and the residue was purified by column chromatography (SiO_2, EtOAc) to afford cyclic tetrapeptide **24** (41 mg, 73 µmol, 73%) as a white foam. **R_f** (EtOAc) 0.31; **mp** 96–98 °C; **^{1}H NMR** (400 MHz, $CDCl_3$) δ_H ppm 7.33 (d, J = 6.3 Hz, 1H, NH), 5.21 (br. s, 1H, NH), 4.67 (d, J = 7.6 Hz, 1H, CH, α-Cys), 4.55 (t, J = 8.6 Hz, 1H, CHα-Pro), 4.47 (d, J = 6.4 Hz, 1H, OC*H*H-Ox), 4.42 (d, J = 6.6 Hz, 1H, OC*H*H-Ox), 4.36 (d, J = 6.6 Hz, 1H, OCH*H*-Ox), 4.21 (d, J = 6.4 Hz, 1H, OCH*H*-Ox), 4.05 (dd, J = 14.2, 7.0 Hz, 1H, C*H*HGOx), 3.63 (dd, J = 16.0, 7.2 Hz, 2H, CH_2δ-Pro), 3.47 (d, J = 14.2 Hz, 1H, CH*H*GOx), 3.32 (dd, J = 7.4, 5.1 Hz, 1H, CH, α-Cys), 3.25 (dd, J = 11.9, 3.3 Hz, 1H, C*H*Hβ-Cys), 3.15 (d, J = 11.4 Hz, 1H, CH*H*β-Cys), 3.09 (dd, J = 8.9, 3.8 Hz, 1H, C*H*Hβ-Cys), 2.83 (dd, J = 13.1, 8.3 Hz, 1H, CH*H*β-Cys), 2.50 (dd, J = 12.1, 6.4 Hz, 1H, C*H*Hβ-Pro), 2.43 (br. s, 1H, NH), 2.21–2.07 (m, 1H, C*H*Hγ-Pro), 2.05–1.95 (dd, J = 6.6, 3.6 Hz, 1H, CH*H*γ-Pro), 1.83 (ddd, J = 11.7, 7.4, 4.4 Hz, 1H, CH*H*β-Pro), 1.43 (s, 9H, 3 × CH_3, *t*Bu), 1.42 (s, 9H, 3 × CH_3, *t*Bu); **^{13}C NMR** (101 MHz, $CDCl_3$) δ_C ppm 173.3 (C=O), 171.0 (C=O), 170.3 C=O), 154.7 (C=O, Boc), 82.6 (C, *t*Bu), 81.8 (OCH_2), 81.0 (OCH_2), 80.8 (C, Boc), 59.9 (CH, α-Cys), 58.4 (C, Ox), 55.4 (CH, α-Cys), 51.4 (CH, α-Pro), 47.8 (CH_2, δ-Pro), 42.8 (CH_2, GOx), 41.1 (CH_2, β-Cys), 40.0 (CH_2, β-Cys), 28.4 (CH_3, *t*Bu), 28.1 (CH_3, *t*Bu), 26.8 (CH_2, β-Pro), 25.1 (CH_2, γ-Pro); **ν_{max}** (neat) = 3297, 2976, 1708, 1668, 1634, 1514, 1451, 1366, 1247, 1147, 971, 731 cm^{-1}; **MS** (ESI$^+$) *m/z* 561 [M+H]$^+$, 583 [M+Na]$^+$; **HRMS** (ESI$^+$) calcd. for $C_{24}H_{40}N_4NaO_7S_2$ [M+Na]$^+$ 583.2231, found 583.223; $[\alpha]_D^{29}$ +43.2 (*c* 0.76, $CHCl_3$).

2.18 Preparation of cyclic disulfide-bridged tetrapeptide ***136***

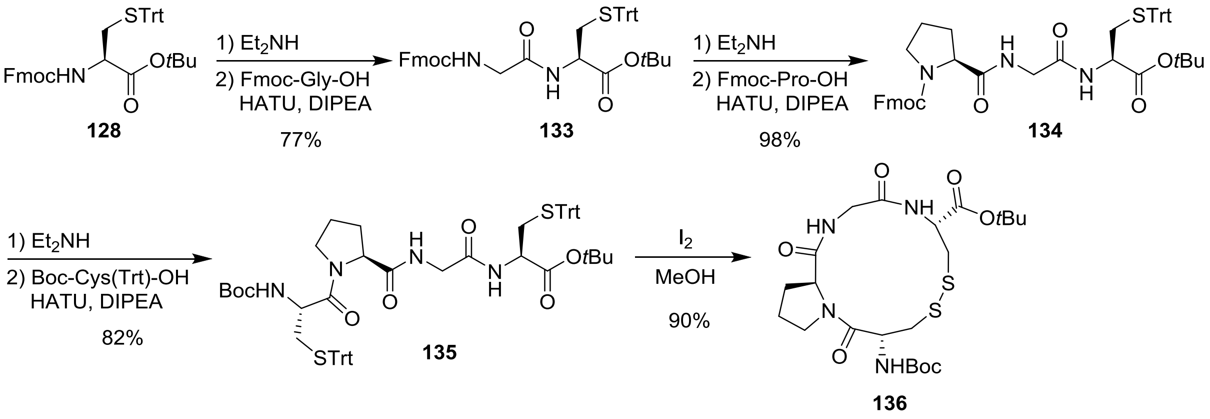

Fmoc-Gly-Cys(Trt)-O*t*Bu (133)

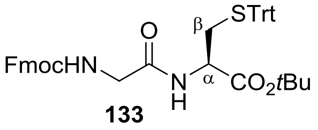

To a solution of Fmoc-Cys(Trt)-O*t*Bu (**128**) (3.15 g, 4.90 mmol, 1.0 equiv) in CH_2Cl_2 (5.0 mL) was added diethylamine (5.0 mL) and the mixture was stirred at room temperature for 1 h. The reaction mixture was concentrated under reduced pressure and the resulting residue repeatedly dissolved in CH_2Cl_2 (3 × 15 mL) and concentrated under reduced pressure to give the crude amine. The residue was dissolved in CH_2Cl_2 (50 mL), Fmoc-Gly-OH (1.75 g, 5.89 mmol, 1.2 equiv), HATU (2.24 g, 5.89 mmol, 1.2 equiv) and DIPEA (2.56 mL, 14.7 mmol, 3.0 equiv) were added subsequently, and the mixture was stirred at room temperature for 16 h. The reaction mixture was diluted with CH_2Cl_2 (30 mL) and washed with 10% citric acid solution (50 mL) and saturated $NaHCO_3$ solution (50 mL), dried over $MgSO_4$, filtered and concentrated under reduced pressure. The residue was purified by flash column chromatography (SiO_2, PE/EtOAc 2:1→1:1) to give dipeptide **133** (2.63 g, 3.80 mmol, 77%) as a white foam. $\mathbf{R_f}$ (PE/EtOAc 1:1) 0.38; **mp** 85–86 °C; **^{1}H NMR** (400 MHz, $CDCl_3$) δ_H ppm 7.77 (d, *J* = 7.5 Hz, 2H, ArH), 7.59 (d, *J* = 7.0 Hz, 2H, ArH), 7.43–7.36 (m, 8H, ArH), 7.32–7.25 (m, 8H, ArH), 7.20 (t, *J* = 7.2 Hz, 3H, ArH), 6.35 (br. s, 1H, NH), 5.42 (br. s, 1H, NH), 4.51 (dd, *J* = 12.2, 5.3 Hz, CHα-Cys), 4.43 (dd, *J* = 9.4, 6.5 Hz, C*H*H-Fmoc), 4.39 (dd, *J* = 9.4, 6.2 Hz, CH*H*-Fmoc), 4.23 (t, *J* = 7.0 Hz, 1H, CH-Fmoc), 3.87 (d, *J* = 3.3 Hz, 1H, CH_2Gly), 2.69 (dd, *J* = 12.2, 5.6 Hz, 1H, C*H*Hβ-Cys), 2.54 (dd, *J* = 12.2, 4.5 Hz, 1H, CH*H*β-Cys), 1.44 (s, 9H, 3 × CH_3, *t*Bu); **^{13}C NMR** (101 MHz, $CDCl_3$) δ_C ppm 169.2 (C=O), 168.3 (C=O), 156.51 (C=O, Fmoc), 144.4 (C), 144.0 (C), 143.9 (C), 141.4 (C), 129.6 (CH), 128.1 (CH), 127.9 (CH), 127.2 (CH), 127.0 (CH), 125.2 (CH), 120.1 (CH), 83.1 (C, *t*Bu), 67.4 (CH_2, Fmoc), 66.9 (CS), 51.8 (CH, α-Cys), 47.2 (CH, Fmoc), 44.4 (CH_2Gly), 34.1 (CH_2, β-Cys), 28.1 (CH_3, *t*Bu); $\mathbf{\nu_{max}}$ (neat) = 3300, 2976, 1726, 1668, 1508, 1445, 1245, 1149, 739, 698 cm^{-1}; **MS** (ESI^+) *m/z* 721 $[M+Na]^+$; **HRMS** (ESI^+) calcd. for $C_{43}H_{42}N_2NaO_5S$ $[M+Na]^+$ 721.2707, found 721.2704; $[\boldsymbol{\alpha}]_D^{29}$ +21.3 (*c* 1.31, $CHCl_3$).

Fmoc-Pro-Gly-Cys(Trt)-O*t*Bu (134)

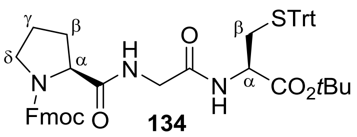

To a solution of Fmoc-Gly-Cys(Trt)-O*t*Bu (**133**) (2.55 g, 3.65 mmol, 1.0 equiv) in CH_2Cl_2 (4.0 mL) was added diethylamine (4.0 mL) and the mixture was stirred at room temperature for 1 h. The reaction mixture was concentrated under reduced pressure and the resulting residue repeatedly dissolved in CH_2Cl_2 (3 × 15 mL) and concentrated under reduced pressure to give the crude amine. The residue was dissolved in CH_2Cl_2 (40 mL), Fmoc-Pro-OH (1.48 g, 4.38 mmol, 1.2 equiv), HATU (1.67 g, 4.38 mmol, 1.2 equiv) and DIPEA (1.91 mL, 11.0 mmol, 3.0 equiv) were added subsequently, and the mixture was stirred at room temperature for 16 h. The reaction mixture was

diluted with CH_2Cl_2 (30 mL) and washed with 10% citric acid solution (50 mL) and saturated $NaHCO_3$ solution (50 mL), dried over $MgSO_4$, filtered and concentrated under reduced pressure. The residue was purified by flash column chromatography (SiO_2, PE/EtOAc 1:1→EtOAc) to give tripeptide **134** (2.84 g, 3.57 mmol, 98%) as a white foam. **R_f** (EtOAc) 0.54; **mp** 97–98 °C; **^{1}H NMR** (600 MHz, toluene-*d8* @ 353 K) 7.55 (d, *J* = 7.5 Hz, 2H, ArH), 7.53–7.43 (m, 7H, ArH), 7.19 (t, *J* = 7.4 Hz, 2H, ArH), 7.15 (t, *J* = 7.4 Hz, 2H, ArH), 7.10–7.06 (m, 6H, ArH), 7.00–6.95 (m, 4H, ArH), 6.52 (br. s, 1H, NH), 4.55 (dd, *J* = 10.8, 5.6 Hz, 1H, CHα-Cys), 4.46 (dd, *J* = 10.4, 6.5 Hz, 1H, C*H*H-Fmoc), 4.42 (dd, *J* = 10.4, 6.5 Hz, 1H, CH*H*-Fmoc), 4.04 (t, *J* = 6.5 Hz, 1H, CH-Fmoc), 4.03–3.98 (m, 1H, CHα-Pro), 3.80 (dd, *J* = 16.4, 5.0 Hz, 1H, C*H*H-Gly), 3.51 (dd, *J* = 16.4, 4.5 Hz, 1H, CH*H*Gly), 3.31–3.25 (m, 1H, C*H*Hδ-Pro), 3.20–3.12 (m, 1H, CH*H*δ-Pro), 2.71 (dd, *J* = 12.2, 5.9 Hz, 1H, C*H*Hβ-Cys), 2.66 (dd, *J* = 12.2, 5.0 Hz, 1H, CH*H*β-Cys), 2.08–2.02 (m, 1H, C*H*Hβ-Pro), 1.77–1.68 (m, 1H, C*H*Hγ-Pro), 1.52 (dt, *J* = 12.3, 8.6 Hz, 1H, CH*H*β-Pro), 1.37–1.31 (m, 1H, CH*H*γ-Pro), 1.28 (s, 9H, 3 × CH_3, *t*Bu), 0.98 (s, 1H, NH); **^{13}C NMR** (151 MHz, toluene-*d8* @ 353 K) δ_C ppm 172.1 (C=O), 169.7 (C=O), 168.6 (C=O), 145.4 (C), 144.9 (C), 142.1 (C), 130.2 (CH), 128.4 (CH), 128.0 (CH), 127.5 (CH), 127.4 (CH), 127.1 (CH), 125.6 (CH), 125.5 (CH), 120.3 (CH), 82.0 (C, *t*Bu), 67.8 (CH_2-Fmoc), 67.5 (CS), 61.1 (CH, α-Pro), 52.7 (CH, α-Cys), 48.3 (CH-Fmoc), 47.3 (CH_2, δ-Pro), 43.6 (CH_2, Gly), 35.0 (CH_2, β-Cys), 28.1 (CH_3, *t*Bu), 24.5 (CH_2, γ-Pro). *N.B.* Fmoc carbonyl carbon signal and CH, β-Pro not observed; **ν_{max}** (neat) = 3307, 2978, 1674, 1515, 1419, 1367, 1152, 1122, 740, 700 cm^{-1}; **MS** (ESI^+) *m/z* 818 $[M+Na]^+$; **HRMS** (ESI^+) calcd. for $C_{48}H_{49}N_3NaO_6S$ $[M+Na]^+$ 818.3234, found 818.3238; $[\alpha]_D^{29}$ +3.80 (*c* 1.18, $CHCl_3$).

Boc-Cys(Trt)-Pro-Gly-Cys(Trt)-O*t*Bu (135)

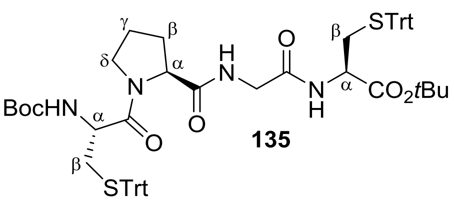

135

To a solution of Fmoc-Pro-Gly-Cys(Trt)-O*t*Bu (**134**) (2.65 g, 3.33 mmol, 1.0 equiv) in CH_2Cl_2 (4.0 mL) was added diethylamine (4.0 mL) and the mixture was stirred at room temperature for 1 h. The reaction mixture was concentrated under reduced pressure and the resulting residue repeatedly dissolved in CH_2Cl_2 (3 × 15 mL) and concentrated under reduced pressure to give the crude amine. The residue was dissolved in CH_2Cl_2 (35 mL), Boc-Cys(Trt)-OH (1.85 g, 4.00 mmol, 1.2 equiv), HATU (1.52 g, 4.00 mmol, 1.2 equiv) and DIPEA (1.74 mL, 10.0 mmol, 3.0 equiv) were added subsequently, and the mixture was stirred at room temperature for 16 h. The reaction mixture was diluted with CH_2Cl_2 (30 mL) and washed with 10% citric acid solution (50 mL) and saturated $NaHCO_3$ solution (50 mL), dried over $MgSO_4$, filtered and concentrated under reduced pressure. The residue was purified by flash column chromatography (SiO_2, PE/EtOAc 1:1→EtOAc) to give tetrapeptide **135** (2.79 g, 2.74 mmol, 82%) as a white foam. **R_f** (EtOAc) 0.57; **mp** 101–105 °C; NMR data reported for the major rotamer: **^{1}H NMR** (600 MHz, $CDCl_3$ @ 313 K) δ_H ppm 7.43 (d, *J* = 7.8 Hz, 6H, ArH), 7.36 (d, *J* = 7.7 Hz, 6H, ArH), 7.31–7.26 (m, 11H, ArH), 7.22 (t, *J* = 7.3 Hz, 6H, ArH), 7.17 (t, *J* = 6.0 Hz, 1H, ArH), 6.67 (d, *J* = 7.2 Hz, 1H, NH), 5.41 (d, *J* = 6.2 Hz, 1H, NH), 4.55 (dd, *J* = 8.0, 4.3 Hz, 1H, CHα-Pro), 4.48 (dd, *J* = 11.4, 6.8 Hz, 1H, CHα-Cys), 4.32 (dd, *J* = 11.7, 6.1 Hz, 1H, CHα-Cys), 3.67 (dd, *J* = 16.7, 6.7 Hz, 1H, C*H*HGly), 3.50–3.39 (m, 2H, CH*H*Gly, C*H*Hδ-Pro), 3.12 (dd, *J* = 12.3, 8.6 Hz, 1H, CH*H*δ-Pro), 2.75 (dd, *J* = 13.3, 5.8 Hz, 1H, C*H*Hβ-Cys), 2.73 (dd, *J* = 12.9, 6.7 Hz, 1H, C*H*Hβ-Cys), 2.57 (dd, *J* = 12.4, 5.0 Hz, 1H, CH*H*β-Cys), 2.44 (dd, *J* = 12.1, 6.3 Hz, 1H, CH*H*β-Cys), 2.23–2.15 (m, 1H, C*H*Hβ-Pro), 2.07–1.99 (m, 1H, CH*H*β-Pro), 1.95 (dt, *J* = 14.6, 6.9 Hz, 1H, C*H*Hγ-Pro), 1.89–1.81 (m, 1H, CH*H*γ-Pro), 1.69 (br. s, 1H, NH), 1.44 (s, 9H, 3 × CH_3, *t*Bu), 1.43 (s, 9H, 3 × CH_3, *t*Bu); **^{13}C NMR** (151 MHz, $CDCl_3$ @ 313 K) δ_C ppm 171.2 (C=O), 170.8 (C=O), 169.3 (C=O), 168.4 (C=O), 155.2 (C=O, Boc), 144.7 (C), 144.3 (C), 129.8 (CH), 129.6 (CH), 128.3 (CH), 128.1 (CH), 127.2 (CH), 126.9 (CH), 82.4 (C, *t*Bu), 80.2 (C, Boc), 67.3 (CS), 67.1 (CS), 61.0 (CH, α-Pro), 52.4 (CH, α-Cys), 51.6 (CH, α-Cys), 47.6 (CH_2, δ-Pro), 42.8 (CH_2, Gly), 34.7

(CH_2, β-Cys), 34.1 (CH_2, β-Cys), 28.5 (CH_3, *t*Bu), 28.5 (CH_2, β-Pro), 28.1 (CH_3, *t*Bu), 25.0 (CH_2, γ-Pro); **ν_{max}** (neat) = 3312, 2979, 1637, 1489, 1443, 1247, 1152, 741, 698 cm^{-1}; **MS** (ESI$^+$) *m/z* 1041 [M+Na]$^+$; **HRMS** (ESI$^+$) calcd. for $C_{60}H_{66}N_4NaO_7S_2$ [M+Na]$^+$ 1041.4265, found 1041.4263; $[\alpha]_D^{28}$ +0.1 (*c* 1.17, $CHCl_3$).

Cyclo(Boc-Cys-Pro-Gly-Cys-O*t*Bu) (136)

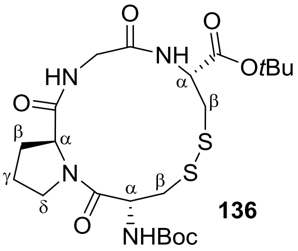

136

To a solution of iodine (152 mg, 0.60 mmol, 3.0 equiv) in anhydrous MeOH (20 mL) was added a solution of tetrapeptide **135** (204 mg, 0.20 mmol, 1.0 equiv) in anhydrous MeOH (10 mL) over a period of 15 min. The mixture was stirred for 1 h at room temperature, cooled to 0 °C and a saturated aqueous solution of $Na_2S_2O_2$ was added until a nearly colourless solution was obtained. The mixture was concentrated *in vacuo* to a volume of ca. 4 mL, EtOAc (25 mL) was added, the solution was washed with 0.1 M aqueous $Na_2S_2O_2$ solution (10 mL), dried over Na_2SO_4 and filtered. The solvent was removed *in vacuo* and the residue was purified by column chromatography (SiO_2, EtOAc→CH_2Cl_2/MeOH 9:1) to afford tetrapeptide **136** (1st run: 78 mg, 0.15 mmol, 73%; 2nd run: 96 mg, 0.18 mmol, 90%) as a white solid. **R_f** (CH_2Cl_2/MeOH 9:1) 0.31; **mp** 226–228 °C (decomposition); **^{1}H NMR** (400 MHz, DMSO-*d6*) δ_H ppm 8.62 (br. s, 0.5H, NH), 7.47 (d, *J* = 6.5 Hz, 1H, NH), 7.42 (d, *J* = 8.2 Hz, 0.5H, NH), 4.41 (t, *J* = 7.7 Hz, 1H, CHα-Cys), 4.25 (dd, *J* = 7.9, 3.7 Hz, 1H, CHα-Pro), 4.11 (dd, *J* = 9.3, 6.6 Hz, 1H, CHα-Cys), 3.77 (dd, *J* = 17.2, 4.1 Hz, 1H, C*H*HGly), 3.70 (dd, *J* = 9.5, 5.5 Hz, 1H, C*H*Hδ-Pro), 3.54 (dd, *J* = 17.2, 3.8 Hz, 1H, CH*H*Gly), 3.44 (dd, *J* = 15.0, 6.4 Hz, 1H, CH*H*δ-Pro), 3.34–3.25 (m, 2H, 2 × C*H*Hβ-Cys), 3.03 (d, *J* = 12.5 Hz, 1H, CH*H*β-Cys), 2.87 (d, *J* = 11.9 Hz, 1H, CH*H*β-Cys), 2.16–1.96 (m, 2H, C*H*Hβ-Pro, C*H*Hγ-Pro), 1.94–1.78 (m, 2H, CH*H*β-Pro, CH*H*γ-Pro), 1.40 (s, 9H, 3 × CH_3, *t*Bu), 1.39 (s, 9H, 3 × CH_3, *t*Bu). *N.B.* One NH signal is nor observed; **^{13}C NMR** (101 MHz, DMSO-*d6*) δ_C ppm 171.9 (C=O), 169.1 (C=O), 168.8 (C=O), 168.7 (C=O), 154.8 (C=O, Boc), 81.3 (C, *t*Bu), 78.9 (C, Boc), 60.9 (CH, α-Pro), 55.1 (CH, α-Cys), 51.9 (CH, α-Cys), 46.8 (CH_2, δ-Pro), 42.2 (CH_2, Gly), 37.3 (CH_2, β-Cys), 34.6 (CH_2, β-Cys), 28.5 (CH_2, β-Pro), 28.1 (CH_3, *t*Bu), 27.5 (CH_3, *t*Bu), 24.7 (CH_2, γ-Pro); **ν_{max}** (neat) = 3307, 2978, 1758, 1706, 1673, 1536, 1509, 1430, 1304, 1226, 1157, 1044, 1022, 710 cm^{-1}; **MS** (ESI$^+$) *m/z* 555 [M+Na]$^+$; **HRMS** (ESI$^+$) calcd. for $C_{22}H_{36}N_4NaO_7S_2$ [M+Na]$^+$ 555.1918, found 555.1919; $[\alpha]_D^{29}$ –39.2 (*c* 0.15, $CHCl_3$).

*2.19 Preparation of pentapeptides **137** and **138***

H-Leu-Ala-Gly-Ala-Tyr-OMe (137)

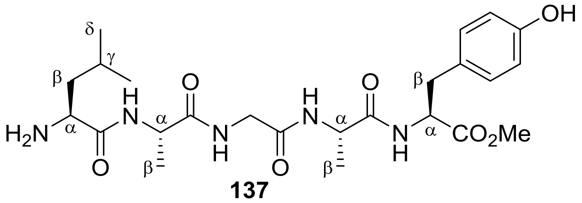

137

Cbz-Leu-Ala-Gly-Ala-OH was synthesised as described above on solid-phase starting from Fmoc-Ala-2-chlorotrityl resin (193 mg, 0.10 mmol, 1.0 equiv). After cleavage from the resin the tetrapeptide (35 mg, 75 μmol, 1.0 equiv) was dissolved in DMF (5.0 mL), H-Tyr-OMe (29 mg, 0.15 mmol, 2.0 equiv), HATU (29 mg, 75 μmol, 1.0 equiv) and diisopropylethylamine (52 μL, 0.30 mmol, 4.0 equiv) were added subsequently, and the mixture was stirred at room temperature for 16 h. The reaction mixture was diluted with CH_2Cl_2 (20 mL) and washed with 10% citric acid solution (20 mL) and saturated $NaHCO_3$ solution (20 ml), dried over $MgSO_4$, filtered and concentrated under reduced pressure. The residue was purified by column chromatography (SiO_2, CH_2Cl_2/MeOH 95:5→9:1) to give Cbz-Leu-Ala-Gly-Ala-Tyr-OMe (34 mg, 75 μmol) as a white solid. The pentapeptide

was dissolved in anhydrous MeOH (2.05 mL), 10 wt% Pd/C (7.5 mg, 20 wt%) was added and the reaction flask was evacuated, filled with nitrogen, evacuated, and placed under an atmosphere of hydrogen (balloon). The reaction mixture was stirred at room temperature for 6 h, placed under nitrogen and filtered through a plug of Celite, which was washed with MeOH. The filtrate was concentrated *in vacuo* and the residue re-dissolved in water. After freeze-drying **137** was obtained as a white solid (22 mg, 43 μmol) in 43% yield over all steps. **mp** 106–107 °C; **^{1}H NMR** (700 MHz, DMSO-*d6*) δ_H ppm 8.22 (d, *J* = 7.8 Hz, 1H, NH), 8.17 (t, *J* = 6.0 Hz, 1H, NH), 8.14 (br. s, 1H, OH), 7.87 (d, *J* = 7.9 Hz, 1H, NH), 6.96 (d, *J* = 8.7 Hz, 2H, ArH), 6.63 (d, *J* = 8.7 Hz, 2H, ArH), 4.31 (dt, *J* = 5.9, 5.5 Hz, 1H, CHα-Tyr), 4.28 (quint, *J* = 7.3 Hz, 1H, CHα-Ala), 4.23 (dd, *J* = 14.9, 7.2 Hz, 1H, CHα-Leu), 3.66 (d, *J* = 6.1 Hz, 2H, CH_2Gly), 3.54 (s, 3H, OCH_3), 3.16 (dd, *J* = 9.5, 5.1 Hz, 1H, CHα-Ala), 2.86 (dd, *J* = 13.7, 6.2 Hz, 1H, C*H*Hβ-Tyr), 2.79 (dd, *J* = 13.7, 8.7 Hz, 1H, CH*H*β-Tyr), 1.70 (nonet, *J* = 6.7 Hz, 1H, CHγ-Leu), 1.38 (ddd, *J* = 13.8, 9.0, 5.0 Hz, 1H, C*H*Hβ-Leu), 1.22–1.19 (m, 1H, CH*H*β-Leu), 1.14 (d, *J* = 7.3 Hz, 3H, CH_3β-Ala), 1.14 (d, *J* = 7.3 Hz, 3H, CH_3β-Ala), 0.85 (d, *J* = 6.7 Hz, 3H, CH_3δ-Leu), 0.81 (d, *J* = 6.7 Hz, 3H, CH_3δ-Leu). *N.B.* Three protic NH signals not observed; **^{13}C NMR** (126 MHz, DMSO-*d6*) δ_C ppm 175.3 (C=O), 172.6 (C=O), 172.2 (C=O), 171.9 (C=O), 168.2 (C=O), 156.0 (C), 130.0 (CH), 127.0 (C), 115.1 (CH), 54.0 (CH, α-Tyr), 52.9 (CH, α-Ala), 51.8 (OCH_3), 48.1 (CH, α-Leu), 47.7 (CH, α-Ala), 43.8 (CH_2, β-Leu), 41.9 (CH_2, Gly), 35.9 (CH_2, β-Tyr), 24.0 (CH, γ-Leu), 23.3 (CH_3, δ-Leu), 21.7 (CH_3, δ-Leu), 18.3 (CH_3, β-Ala), 18.2 (CH_3, β-Ala); **ν_{max}** (neat) = 3291, 2957, 1738, 1644, 1515, 1447, 1368, 1221, 1172, 829 cm^{-1}; **MS** (ESI$^+$) *m/z* 508 [M+H]$^+$, 530 [M+Na]$^+$; **HRMS** (ESI$^+$) calcd. for $C_{24}H_{37}N_5NaO_7$ [M+Na]$^+$ 530.2585, found 530.2589; $[\alpha]_D^{24}$ +31.3 (*c* 0.02, MeOH).

H-Leu-Ala-GOx-Ala-Tyr-OMe (138)

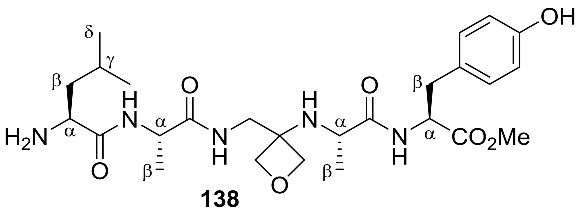

138

To a solution of Cbz-Leu-Ala-GOx-Ala-Tyr(Bn)-OMe (752 mg, 0.99 mmol, 1.0 equiv) in anhydrous MeOH (10 mL) was added 10 wt% Pd/C (75 mg, 10 wt%) and the reaction flask was evacuated, filled with nitrogen, evacuated, and placed under an atmosphere of H_2 (balloon). The reaction mixture was stirred at room temperature for 16 h, placed under N_2 and filtered through a plug of Celite, which was washed with MeOH. The filtrate was concentrated *in vacuo* to give **138** as a white solid (502 mg, 0.94 mmol, 95%). **mp** 116–118 °C; **^{1}H NMR** (700 MHz, DMSO-*d6*) δ_H ppm 8.63 (d, *J* = 8.0 Hz, 1H, NH), 8.30 (d, *J* = 8.4 Hz, 1H, NH), 8.00 (t, *J* = 6.2 Hz, 1H, NH), 6.97 (d, *J* = 8.7 Hz, 2H, ArH), 6.66 (d, *J* = 8.7 Hz, 2H, ArH), 4.43–4.39 (m, 1H, CHα-Tyr), 4.37 (quint, *J* = 7.2 Hz, 1H, CHα-Ala), 4.28 (d, *J* = 6.5 Hz, 1H, OC*H*H-Ox), 4.18 (d, *J* = 6.5 Hz, 1H, OC*H*H-Ox), 4.11 (d, *J* = 6.5 Hz, 1H, OCH*H*-Ox), 3.96 (d, *J* = 6.5 Hz, 1H, OCH*H*-Ox), 3.66 (dd, *J* = 8.1, 6.9 Hz, CHα-Leu), 3.59 (s, 3H, OCH_3), 3.39 (dd, *J* = 13.6, 6.6 Hz, 1H, C*H*HGOx), 3.31 (dd, *J* = 13.6, 5.6 Hz, 1H, CH*H*GOx), 3.29 (q, *J* = 7.2 Hz, 1H, CHα-Ala), 2.95 (dd, *J* = 13.8, 5.3 Hz, 1H, C*H*Hβ-Tyr), 2.85 (dd, *J* = 13.8, 9.5 Hz, 1H, CH*H*β-Tyr), 1.68 (nonet, *J* = 6.7 Hz, 1H, CHγ-Leu), 1.53 (ddd, *J* = 14.2, 7.8, 6.5 Hz, 1H, C*H*Hβ-Leu), 1.46–1.42 (m, 1H, CH*H*β-Leu), 1.24 (d, *J* = 7.2 Hz, 3H, CH_3β-Ala), 1.06 (d, *J* = 7.2 Hz, 3H, CH_3β-Ala), 0.89 (d, *J* = 6.7 Hz, 3H, CH_3δ-Leu), 0.87 (d, *J* = 6.7 Hz, 3H, CH_3δ-Leu). *N.B.* Four protic NH/OH signals not observed; **^{13}C NMR** (126 MHz, DMSO-*d6*) δ_C ppm 175.6 (C=O), 172.5 (C=O), 172.0 (C=O), 170.0 (C=O), 156.0 (C), 130.0 (CH), 127.1 (C), 115.0 (CH), 78.0 (OCH_2), 77.7 (OCH_2), 59.6 (C, Ox), 53.2 (CH, α-Tyr), 51.8 (OCH_3), 51.6 (CH, α-Ala), 51.2 (CH, α-Leu), 48.4 (CH, α-Ala), 42.7 (CH_2, GOx), 41.1 (CH_2, β-Leu), 35.7 (CH_2, β-Tyr), 23.6 (CH_3, γ-Leu), 22.8 (CH_3, δ-Leu), 22.0 (CH_3, δ-Leu), 20.5 (CH_3, β-Ala), 18.5 (CH_3, β-Ala); **ν_{max}** (neat) = 3271, 2956, 2874, 1739, 1649, 1514, 1441, 1368, 1223, 1174, 967, 830 cm^{-1}; **MS** (ESI$^+$) *m/z* 536 [M+H]$^+$, 558 [M+Na]$^+$; **HRMS** (ESI$^+$) calcd. for $C_{26}H_{42}N_5O_7$ [M+H]$^+$ 536.3079, found 536.3071; $[\alpha]_D^{24}$ –5.1 (*c* 0.20, MeOH).

3. Kinetic measurement of the cyclization reaction

HPLC measurements were conducted on an Agilent 1260 Infinity analytical HPLC system on an Agilent Eclipse Plus C18 column (5.0 µm, 4.6 × 150 mm) with a flow rate of 1.0 mL/min (solvent A: 0.1% TFA in water; solvent B: 0.1% TFA in MeCN; gradient: 0–3 min, 3% B; 3–14 min, 3–20% B; 14–20 min, 20% B; 20–41 min, 20–50% B; 41–43 min, 50–100% B; 43–45 min, 100% B).

To separate 20 mL vials were added linear precursor **6** or **7** (10 µmol, 1.0 equiv) and anhydrous DMF (10 mL). At this time a 500 µL sample was withdrawn to determine the initial value by analytical HPLC before DEPBT (20 µmol, 2.0 equiv) and DIPEA (20 µmol, 2.0 equiv) were added to the solution. At designated time points, 500 µL of reaction mixture was taken and diluted with 200 µL distilled water. 10 µL of these samples were directly injected into the analytical HPLC. Further samples were taken after 0.5, 1, 2, 3, 4, 5, 6, 7, 8, 10, 26, 30, 34, 50 and 74 h and treated in the same manner. Signals for linear precursors **6** or **7** and cyclic peptides **8** or **9** as well as dimer **42** were integrated at 280 nm.

To check the accuracy of the integration at 280 nm, calibration curves for the linear and cyclic oxetane modified peptide **7** and **9** were measured by injecting 10 µL of stock solutions of known concentration. UV signals at 280 nm were integrated and the resulting areas plotted against the amount of injected compound in nmol. Linear fitting gave equations shown below for each compound. The obtained data show that conversions obtained from sole integration of the peaks at 280 nm are in accordance with yields obtained from the calibrations curves. The same was assumed for the parent system (compound **6** and **8**). Conversions were calculated from initial integral of linear precursor determined before addition of coupling reagent and led to 49% for cyclic peptide **8**, 11% for the dimer **42** and 83% for cyclic oxetane modified peptide **9**. Conversion obtained for the dimer was divided by two due to two Trp-residues in the structure. Retention times of linear precursors and formed products was confirmed by LC-MS (Bruker Amazon X) under the same HPLC conditions and injection of purified compounds.

*Calibration curve for WLGOxG (**7**)*

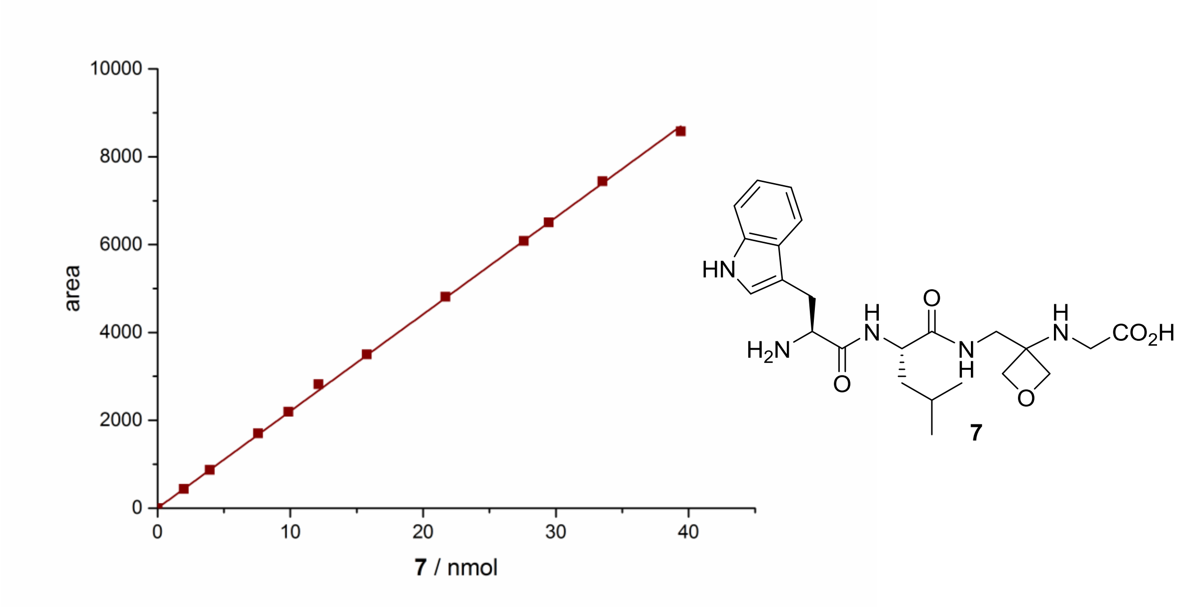

$y = 220.7x$

*Calibration curve for cyclic WLGOxG (**9**)*

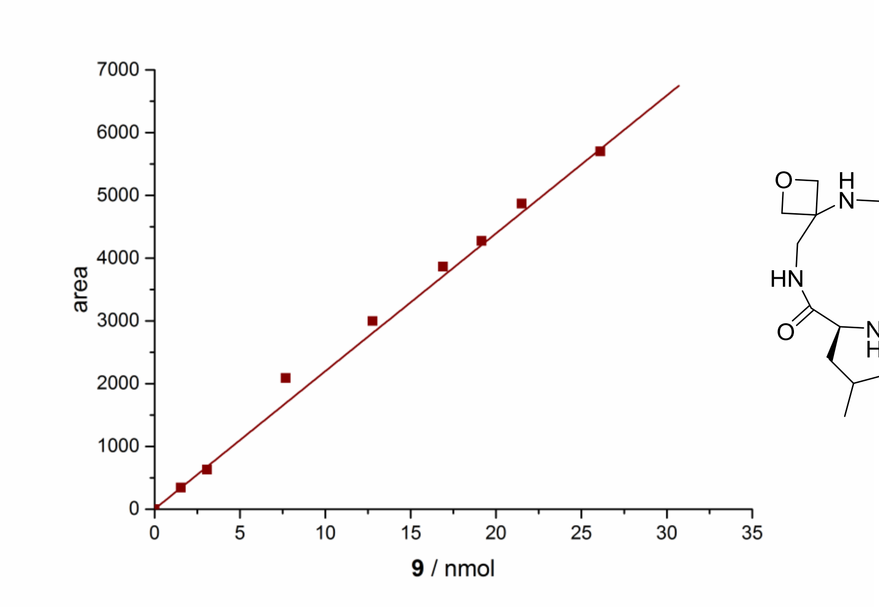

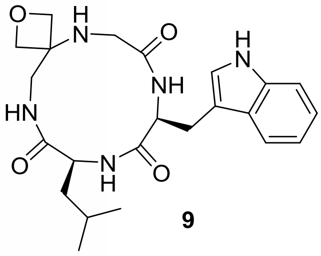

9

$y = 219.8x$

*Conversion of linear peptides **6** & **7** to cyclic peptides **8** & **9** and dimer **42** over 74 h*

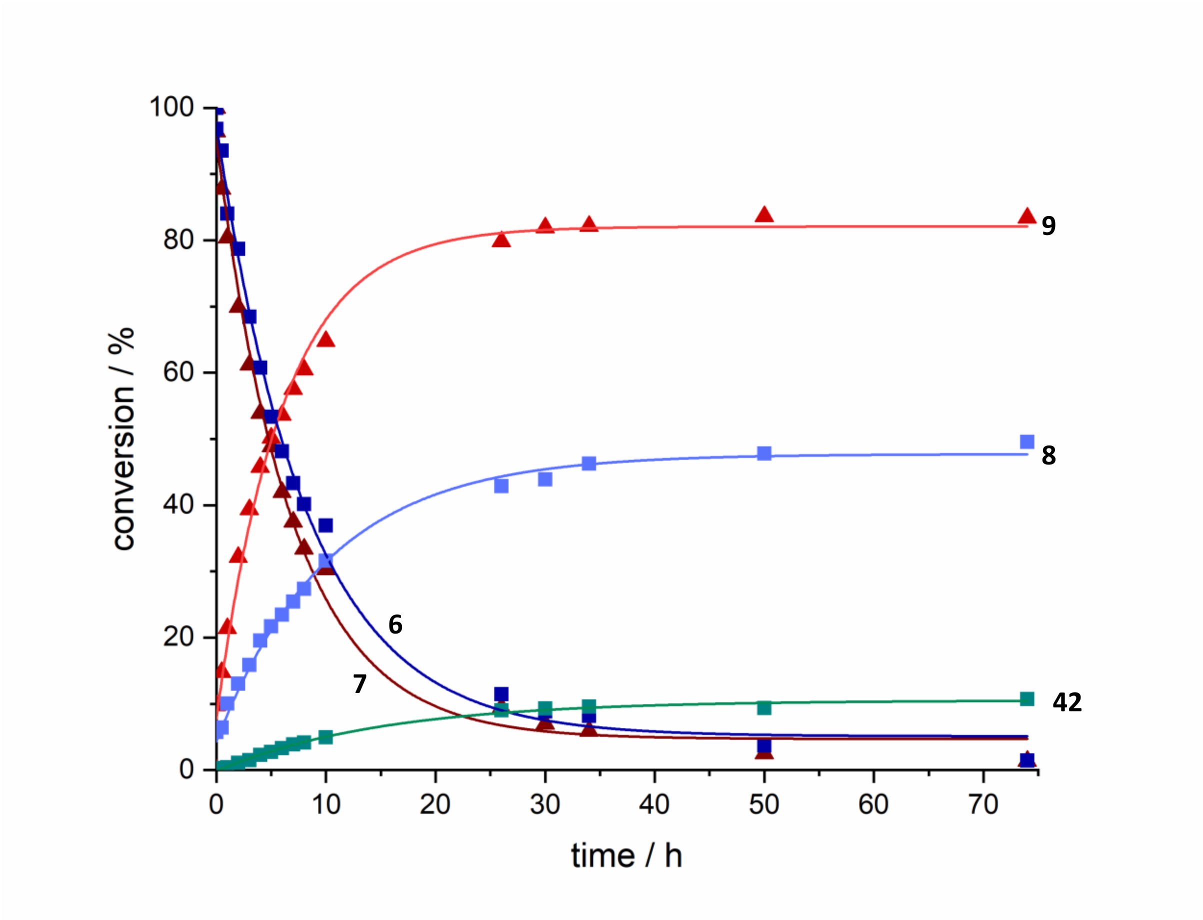

*UV and LC-MS traces (280 nm) of the cyclization toward oxetane modified peptide **9** over 74 h*

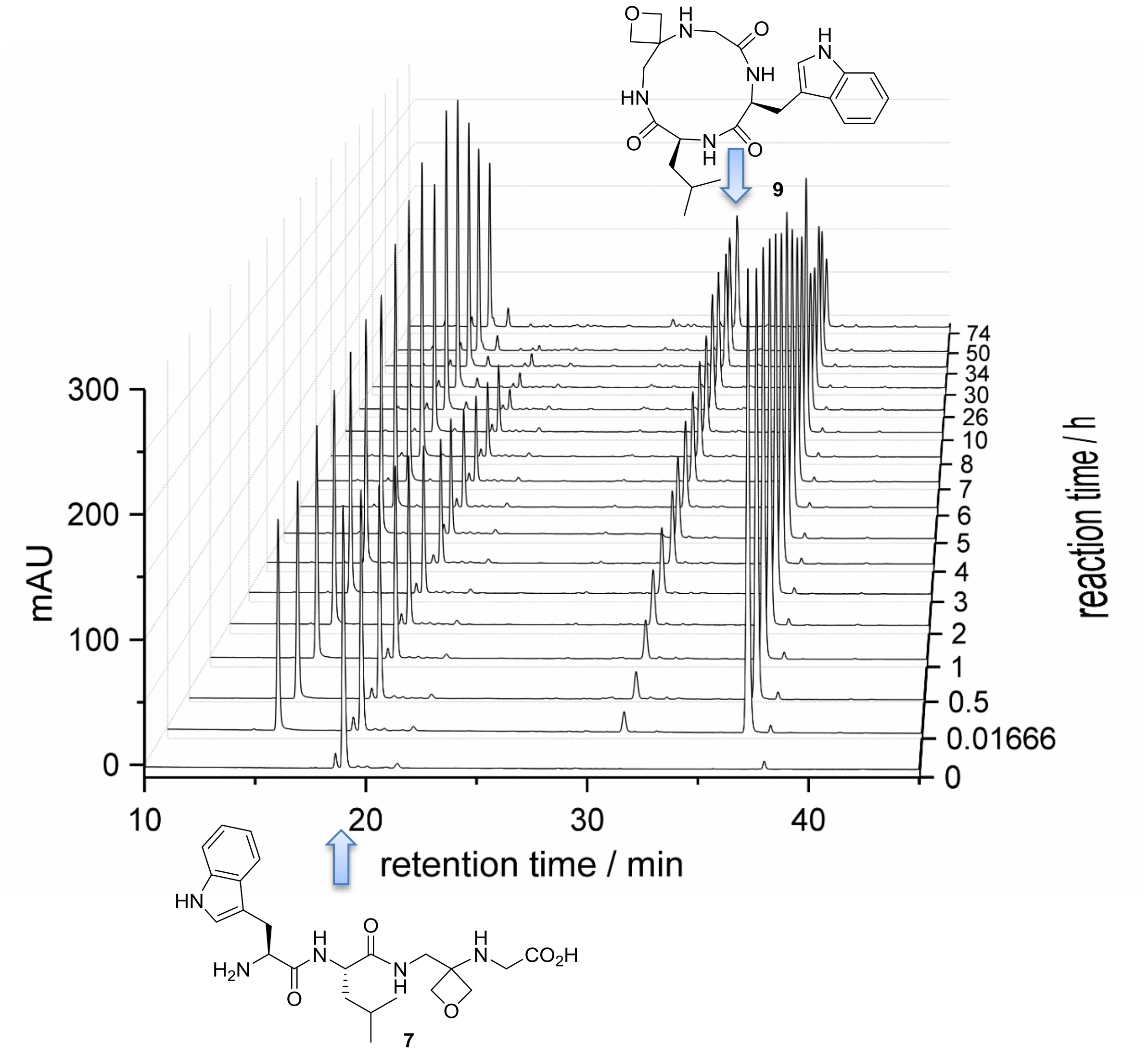

*LC-MS trace (280 nm) of the cyclization reaction toward cyclic peptide **9** after 8 h*

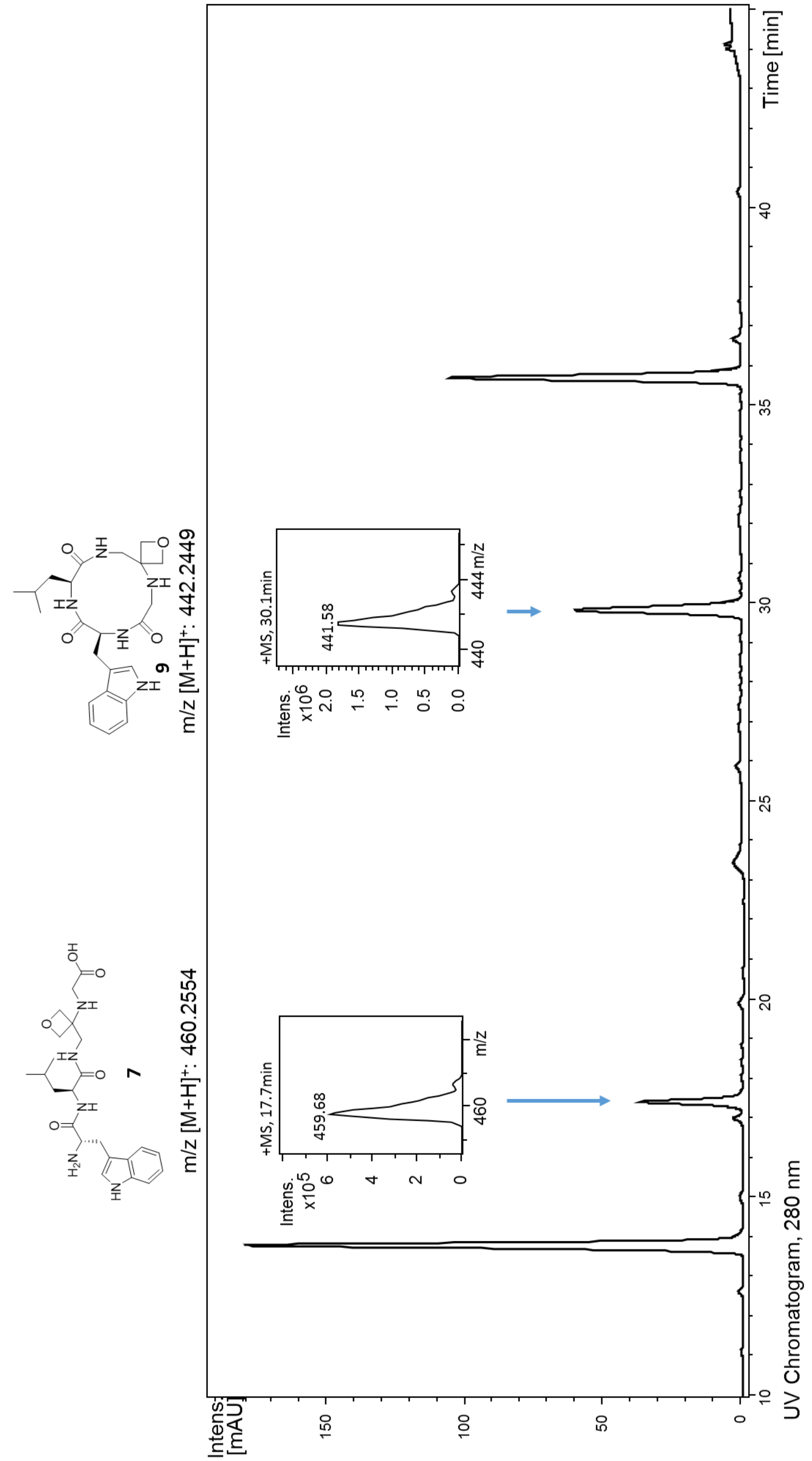

*UV and LC-MS traces (280 nm) of the cyclization reaction toward cyclic peptide **8** and cyclic dimer **42** over 74 h*

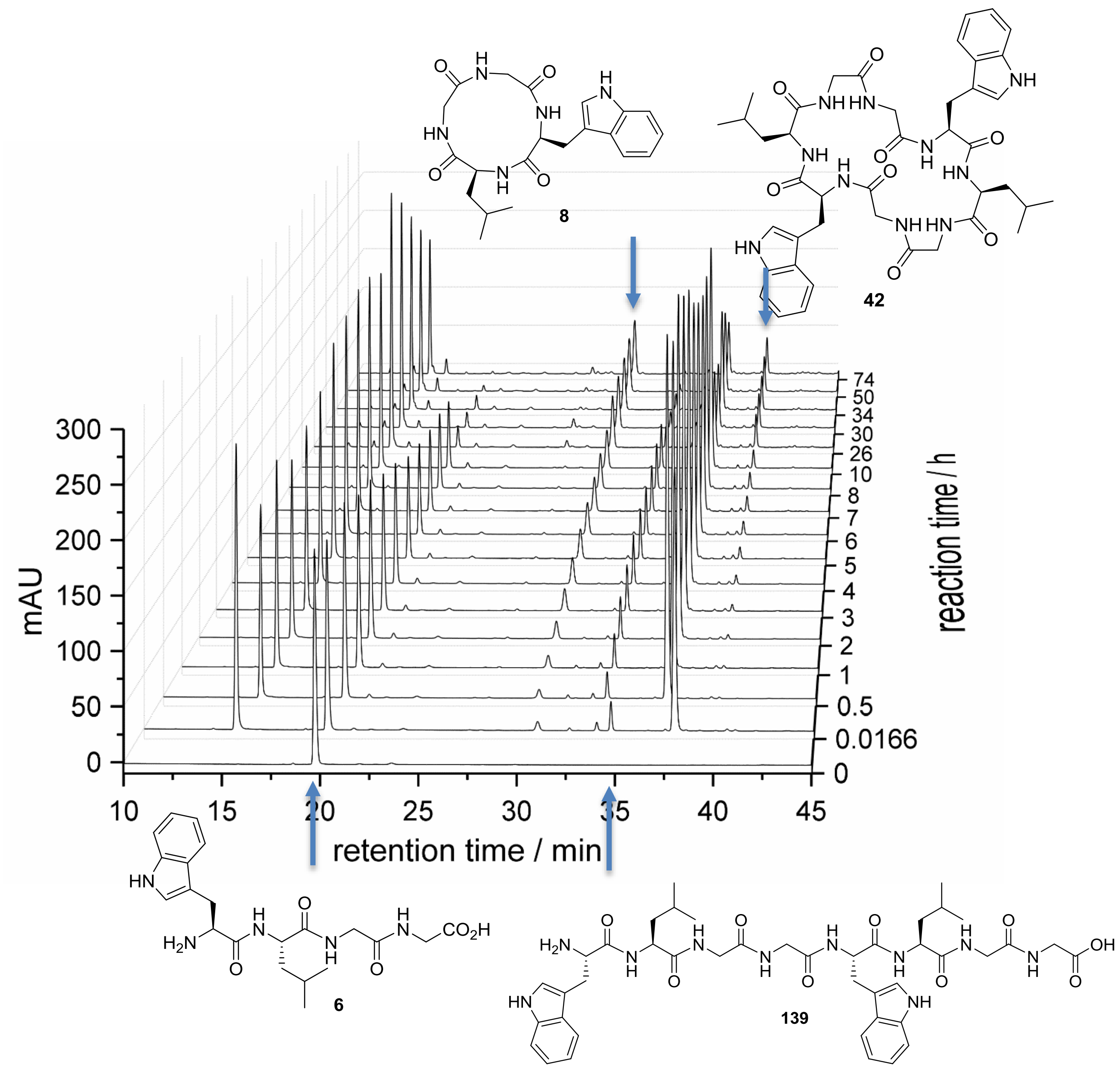

*LC-MS trace (280 nm) of the cyclization reaction toward cyclic peptide **8** and cyclic dimer **42** after 8 h*

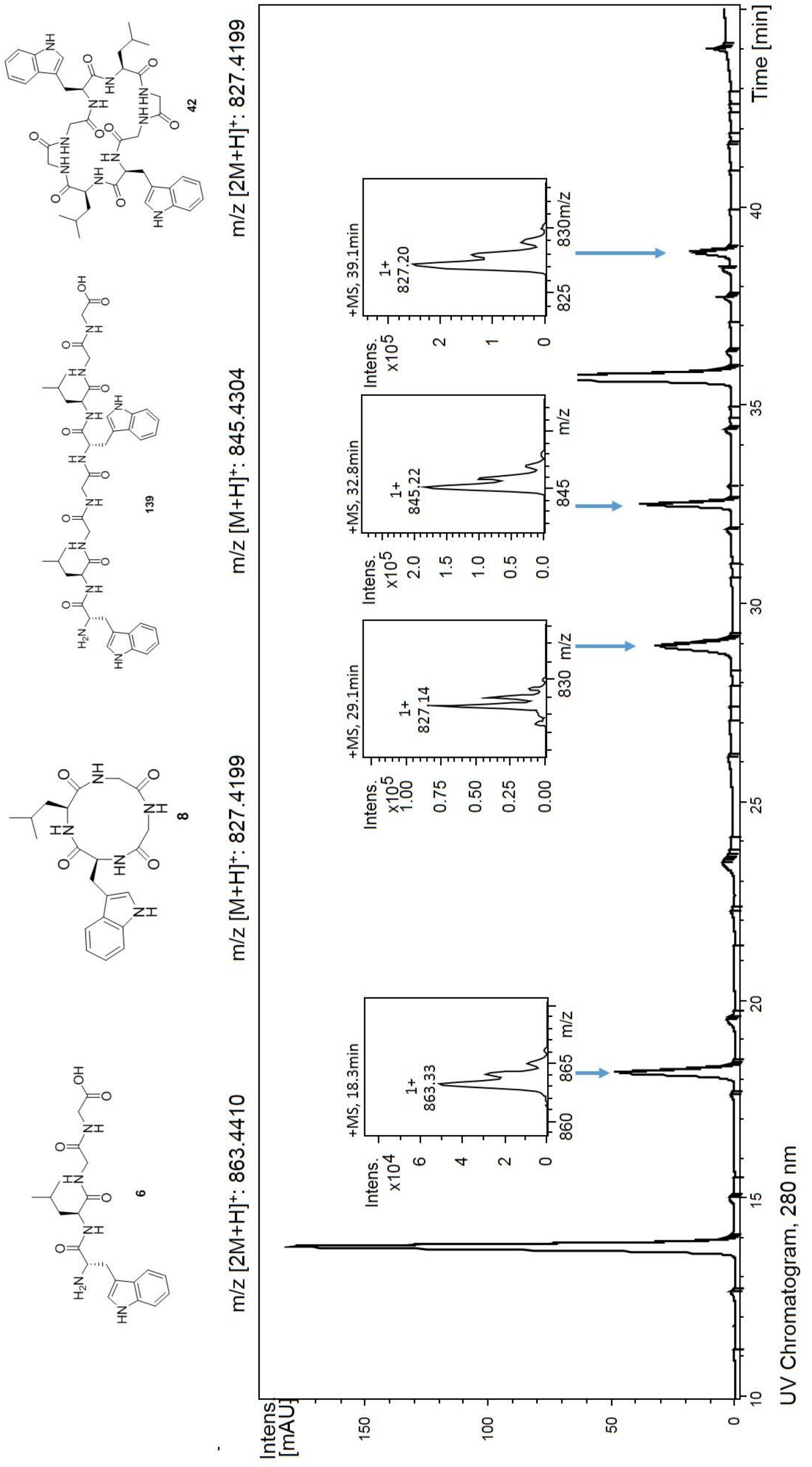

4. In vitro inhibition assay of aminopeptidase N with oxetane modified peptide 22 and parent peptide 26

Peptide **26** was synthesised following a procedure by Piras *et al.*[10] using HCTU as coupling reagent and NMM as base. Oxidation was performed by on-resin cyclization as described for the biotin labelled compound. Analytical data were in accordance with the literature.

For the determination of IC_{50} values of oxetane modified peptide **22** and parent peptide **26**, a protocol published by Piras *et al.*[10] was followed using L-leucine-*p*-nitroanilide as substrate and microsomal aminopeptidase from porcine kidney (*p*APN, Sigma Aldrich) (18 units/mg protein). IC_{50} values were calculated by following the formation of *p*-nitroaniline. Formation of *p*-nitroaniline was monitored by measurement of the UV absorption at 405 nm on a Hidex Sense plate reader. The assay was performed in a 96-well plate in PBS buffer (pH 7.2, 1.47 mM KH_2PO_4, 7.8 mM Na_2HPO_4, 137 mM NaCl, 2.7 mM KCl, 1.8 mM $CaCl_2$, 1.8 mM $MgCl_2$) at 37 °C with a total volume of 100 µL. Bestatin hydrochloride was used as positive control. Peptides were used in gradient concentrations between 2.5 µM and 3.5 mM, and bestatin in concentrations between 50 nM and 25 µM. The peptides were incubated with the enzyme (1.0 µg/mL) for 5 min. before a solution of L-leucine-*p*-nitroanilide was added with a final concentration of 250 µM. The plate was incubated at 37 °C for 1 hour before the *p*-nitroaniline was detected. IC_{50} was defined as the concentration that led to 50% of maximal pAPN catalytic activity. For the calculation of the IC_{50} values log of the concentration was plotted against the UV absorption in *GraphPad* Prism 5 using nonlinear regression (variable slope (four parameters) with interpolation) for analysis.

Figure S1. Inhibition of APN by oxetane modified peptide **22**. The shown data are the average of two independent experiments performed in duplicate. Error bars are displaying standard deviations.

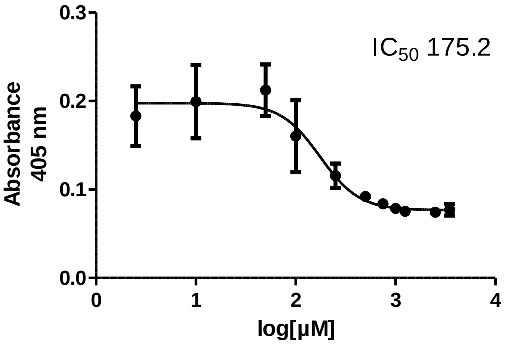

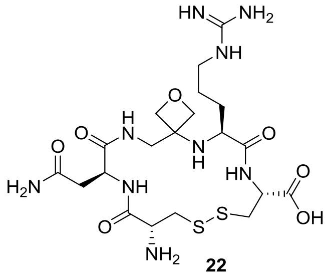

22

Figure S2. Inhibition of APN by native peptide **26**. The shown data are the average of two independent experiments performed in duplicate and triplicate, respectively. Error bars are displaying standard deviations.

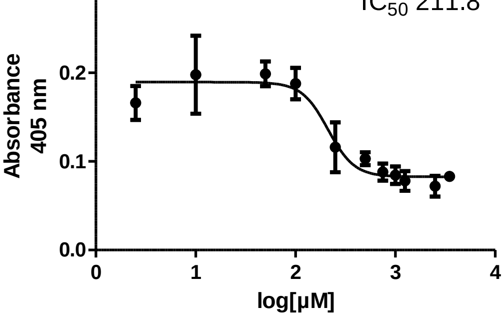

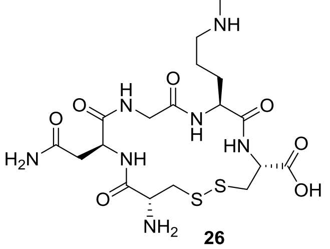

26

5. NMR analysis of pentapeptides 137 and 138

Figure S3. ^{1}H-^{1}H TOCSY spectrum collected with a mixing time of 70 ms (red) and ^{1}H-^{1}H NOESY spectrum collected with a mixing time of 250 ms (green) are shown for peptide **137**. Peak assignments are annotated on the spectrum.

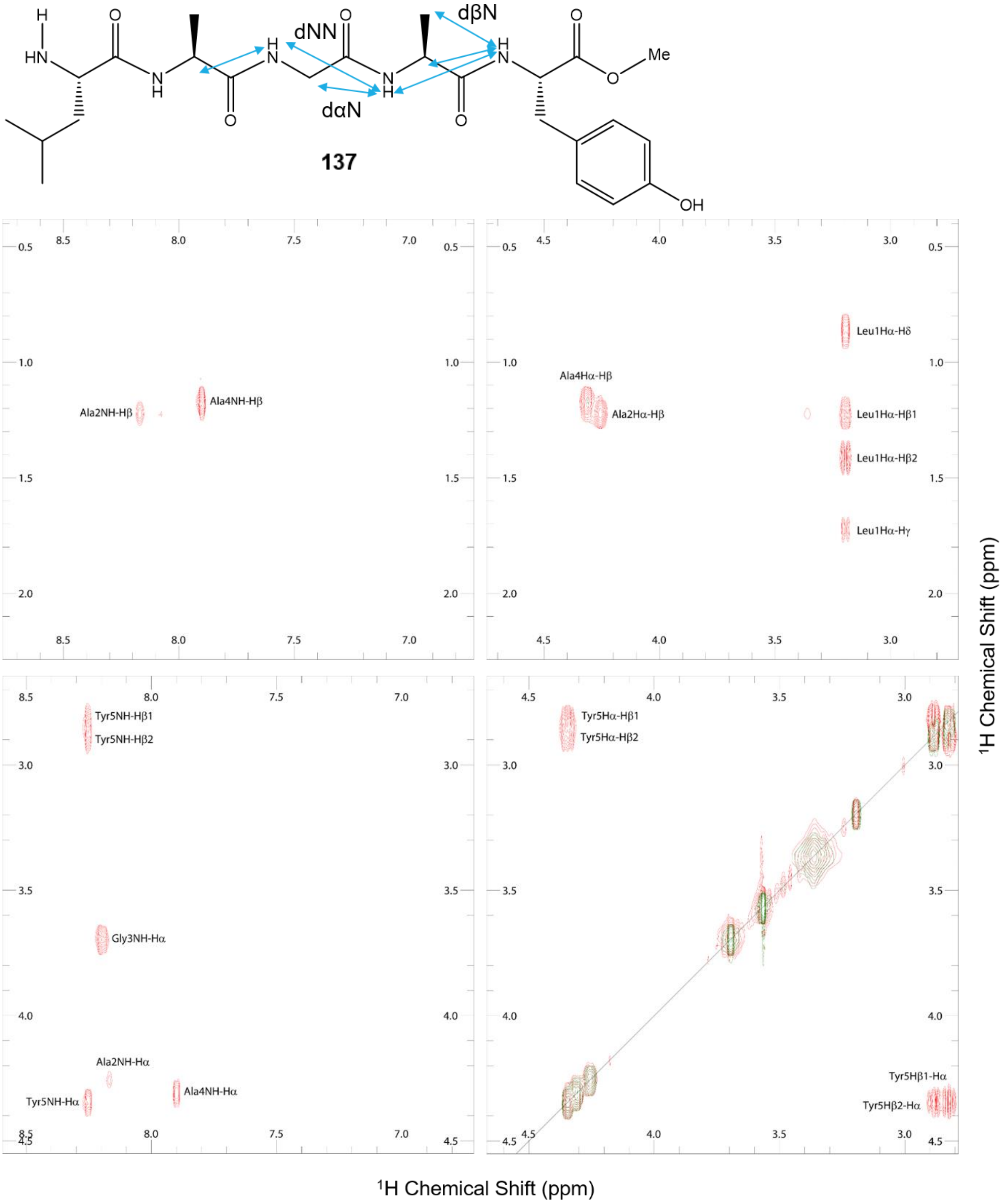

Figure S4. ^{1}H-^{1}H TOCSY spectrum collected with a mixing time of 70 ms (red) and ^{1}H-^{1}H NOESY spectrum collected with a mixing time of 250 ms (green) are shown for peptide **138**. Peak assignments are annotated on the spectrum.

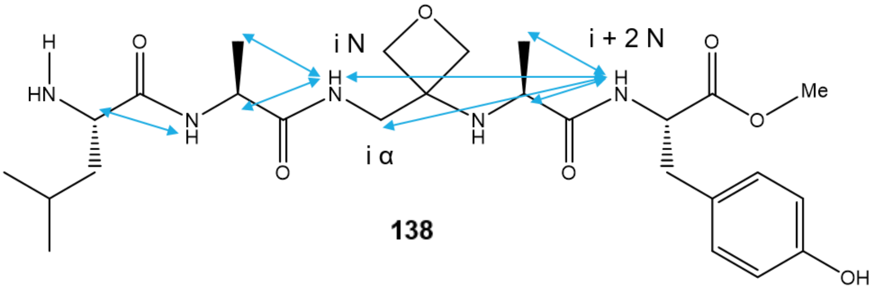

138

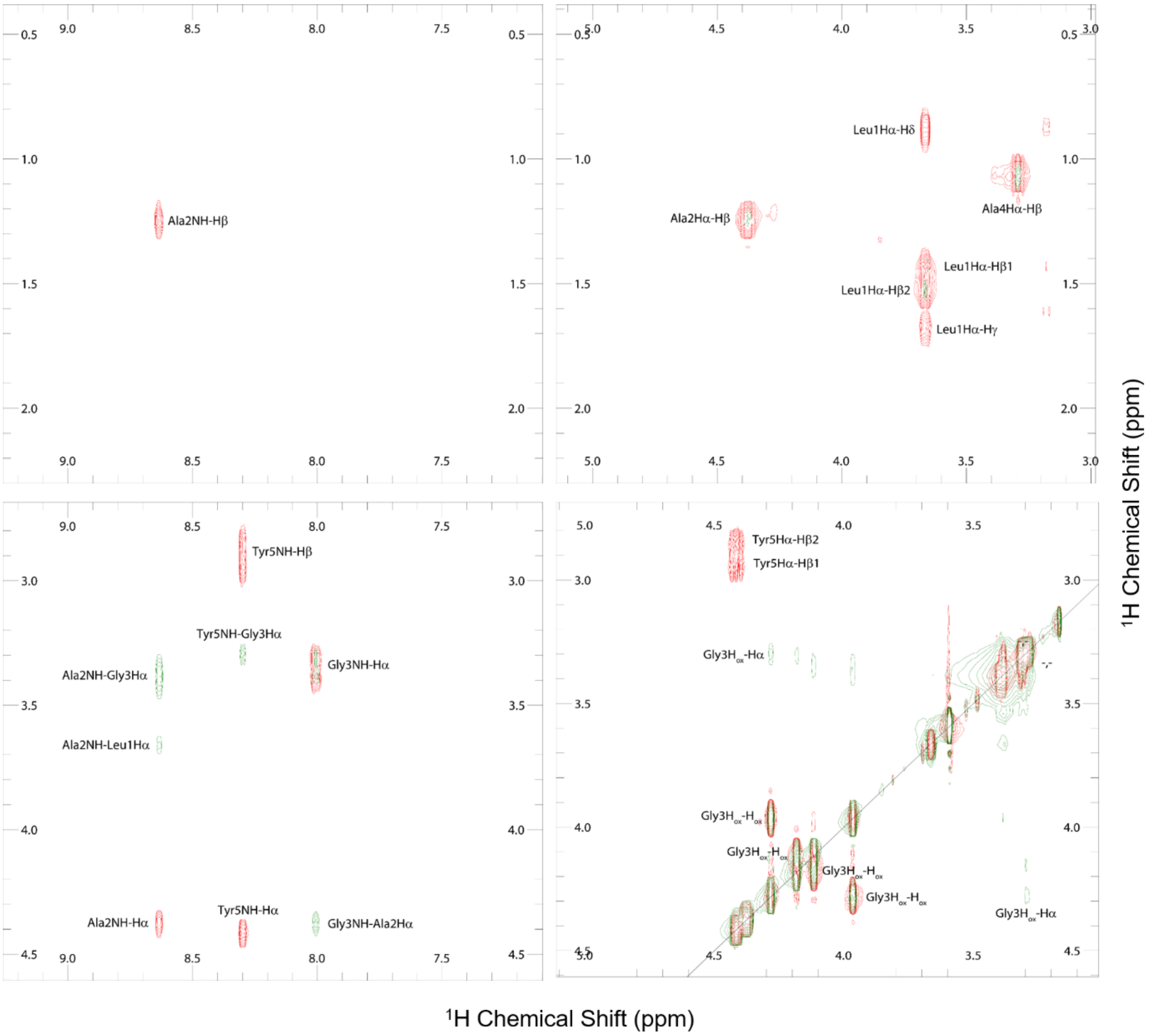

6. NMR analysis of cyclic pentapeptides 13 and 25

Figure S5. Overlay of 1D ^{1}H spectra of peptide **25** and **13** at concentrations ranging from 2–60 mM. No significant chemical shift changes were observed, indicating that the peptide does not have a propensity to self-associate within this concentration range.

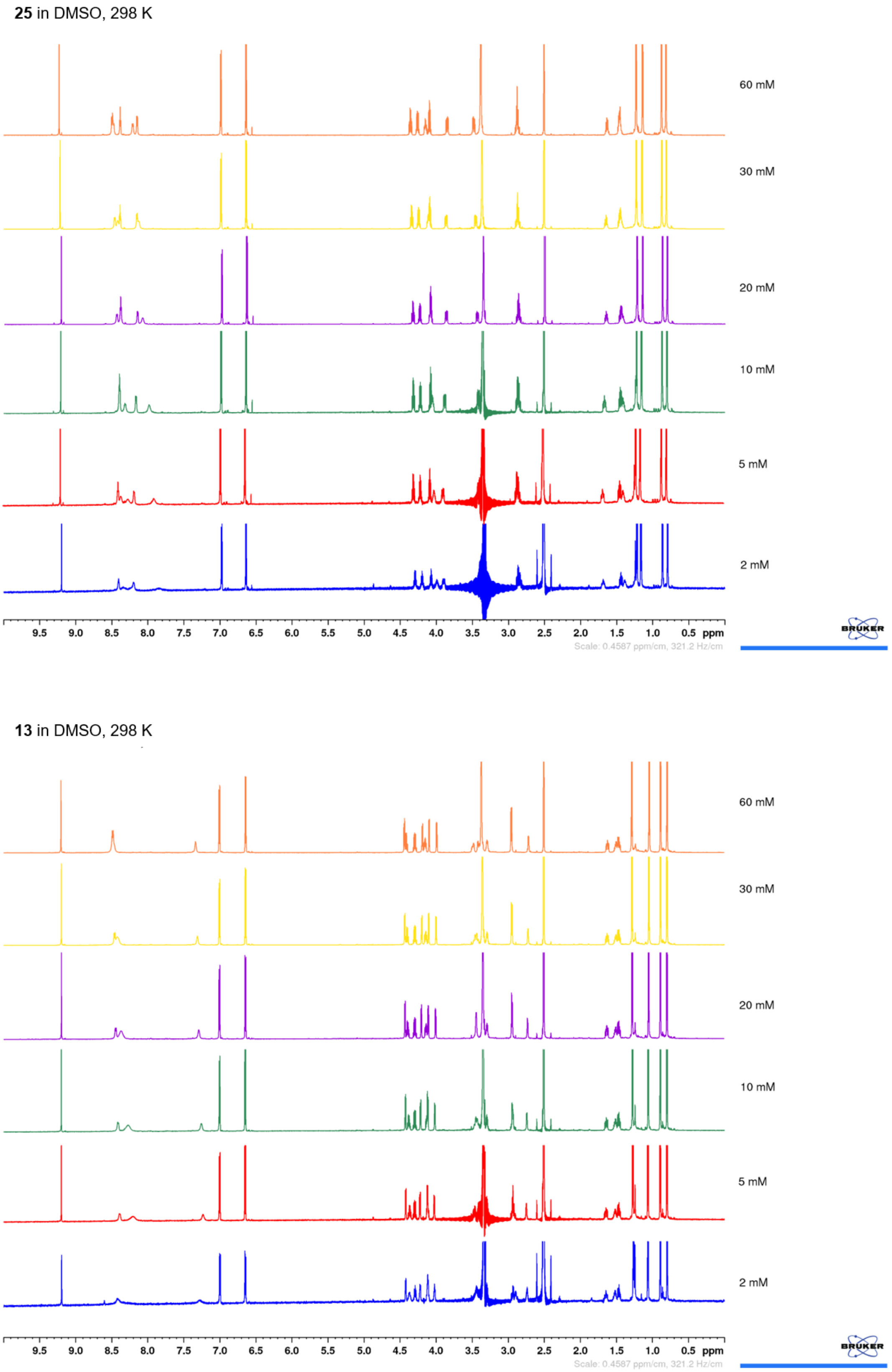

Figure S6. ¹H-¹H TOCSY spectrum collected with a mixing time of 70 ms (red) and ¹H-¹H NOESY spectrum collected with a mixing time of 250 ms (green) are shown for peptide **25**. Peak assignments are annotated on the spectrum.

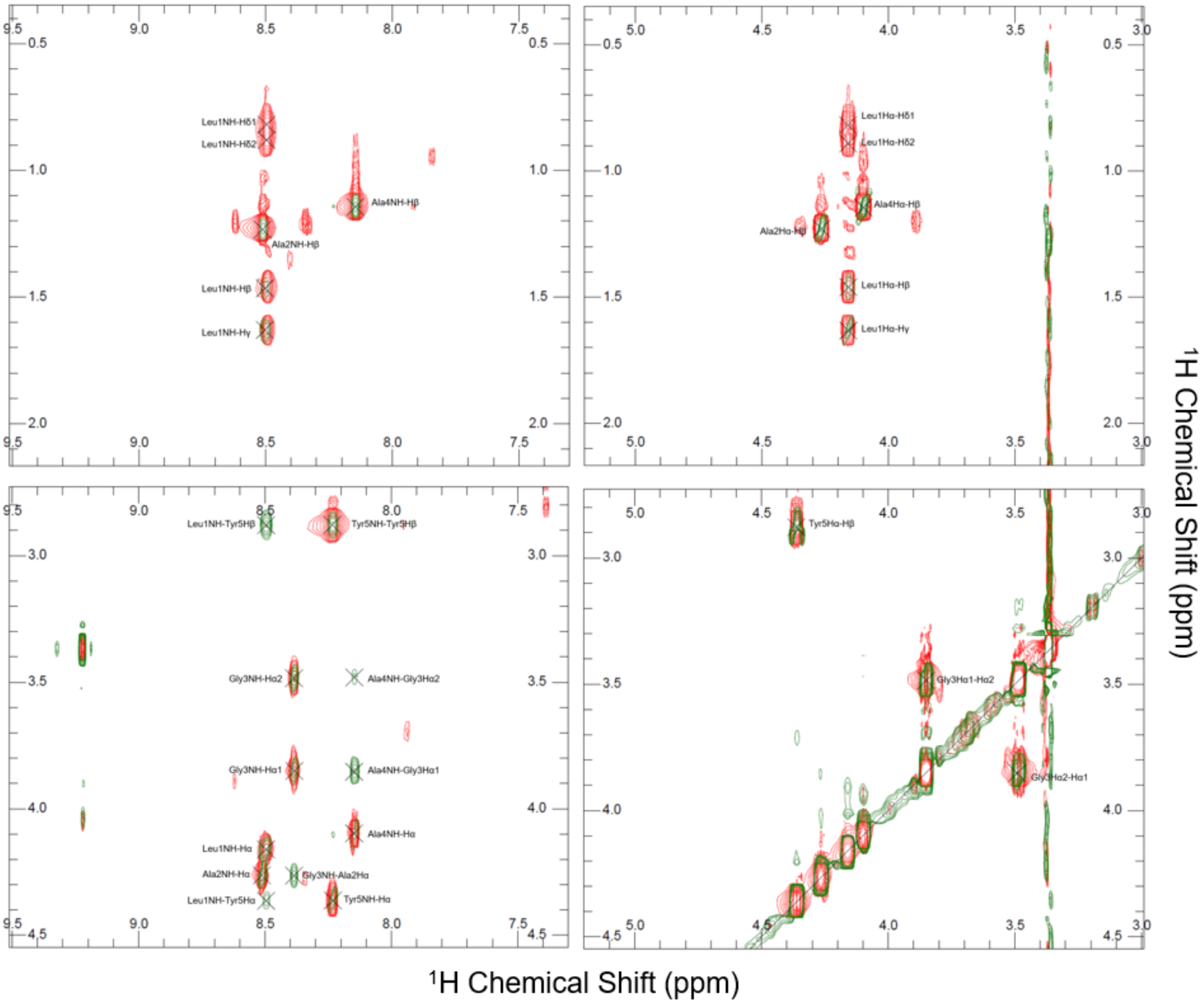

Figure S7. ^1H-^1H TOCSY spectrum collected with a mixing time of 70 ms (red) and ^1H-^1H NOESY spectrum collected with a mixing time of 250 ms (green) are shown for peptide **13**. Peak assignments are annotated on the spectrum.

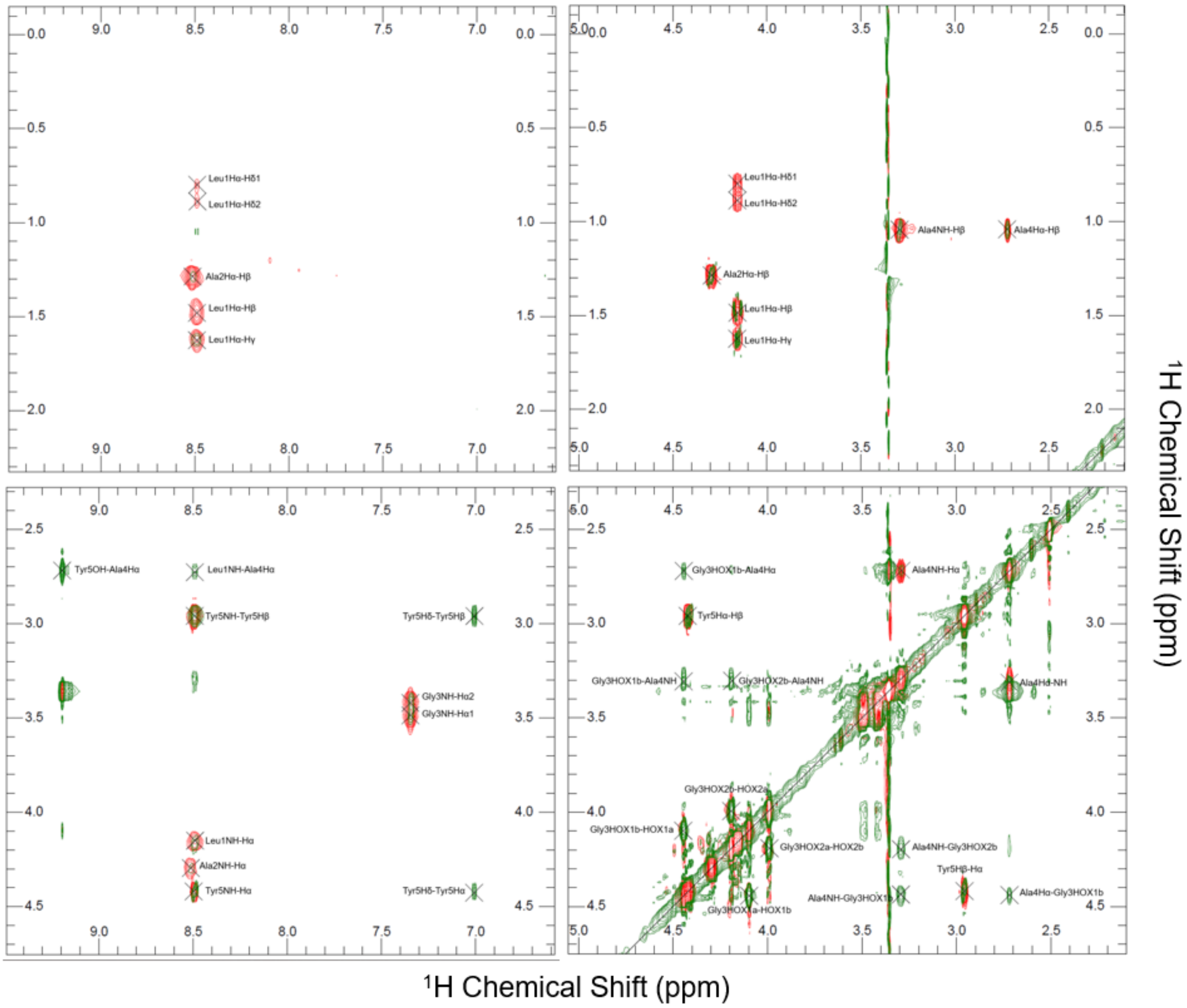

Figure S8. NOE-buildup curves for 4 inter-residue NOE peaks observed in **25** at mixing times ranging from 100–800 ms. All peak volumes were normalised to that of the Tyr aryl proton NOE peaks before plotting.

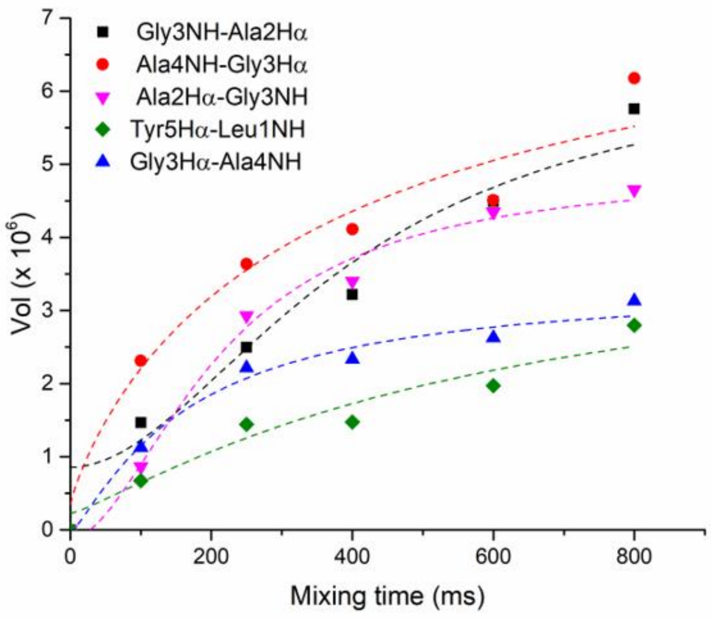

Table S1. Inter-residue NOE observed in cyclic pentapeptides measured in DMSO-d_6 at 25 °C.

Peptide	NOE	Intensity
**25**	3Gly NH-2Ala Hα	Weak
	3Gly Hα1-4Ala NH	Weak
	1Leu NH-5Tyr NH	Strong
	1Leu NH-5Tyr Hβ1	Medium
	1Leu NH-5Tyr Hβ2	Medium
	1Leu NH-5Tyr Hα	Weak
	3Gly Hα2-4Ala NH	Weak
	3Gly NH-5Tyr NH	Medium
**13**	3Gly NH-5Tyr NH	Medium
	4Ala Hα-1Leu NH	Weak
	4Ala Hα-5Tyr OH	Strong
	3Gly NH-4Ala Hα	Weak
	3Gly HOX-4Ala Hα	Weak
	3Gly HOX-4Ala NH	Weak

Table S2.

**Parameter**		**25**	**13**
Root mean squared deviations from experimental distance restraints (Å)	All inter-residue	0.04 ± 0.05	0.25 ± 0.75
	Strong	0.04	2.63
	Medium	0.12 ± 0.03	0.06
	Weak	0.00 ± 0.01	0.00 ± 0.01

7. Molecular dynamics simulations

To obtain insights into the effect of the oxetane ring on the structure of the cyclic peptides we carried out molecular dynamics (MD) computer simulations with NMR restraints of cLAGAY (**25**) and the OMCP cLAGOxAY (**13**) in DMSO.

Model Building

Starting coordinates of the peptides were generated using the Avogadro program version 1.1.0.[11,12] The peptides were built assuming a linear conformation (ϕ, ψ and ω backbone dihedral angles were set to 180°) and uncharged ends. The peptides were then cyclised and, for peptide **13**, the C=O oxetane substitution was made. This was followed by a steepest descents energy minimisation in Avogadro using the Universal Force Field,[12] which generated the starting structure of each cyclic peptide for subsequent MD simulation in Gromacs 5.1.4.[13] Input topology files for Gromacs were generated using the Gromacs pdb2gmx tool after first modifying the residue database to include oxetane-substituted residues. Following a short simulation in the *NVT* ensemble in vacuum in Gromacs (see below) each structure was solvated with a pre-equilibrated box 425 or 428 DMSO molecules, which was sufficient to ensure that the protein was not interacting with its periodic image.

Forcefield Parameters

MD simulations were carried out using the CHARMM27 forcefield for proteins[14] and DMSO[15] with modified parameters for the oxetane ring that were parameterised and are provided in detail in our previous work.[2]

Simulation Parameters

Energy minimization and MD simulations were carried out using the Gromacs simulation package version 5.1.4.[13] The initial structures were relaxed by performing 50000 steps of MD simulation in the *NVT* ensemble in vacuum at 300 K. The final structures were then solvated with DMSO and the system was subjected to 50000 steps of steepest descents energy minimization. This was followed by 100000 steps of simulation at 300 K in the *NVT* ensemble and 50000 steps of simulation at 300 K and 1 bar in the *NPT* ensemble to equilibrate the temperature and density of the system respectively. To overcome the issue of kinetic trapping in local minima and to enhance the sampling of conformational space, each peptide was then simulated for 100 ns at 500 K in the *NVT* ensemble. Cluster analysis (see below) was performed on the resultant trajectory to group peptide conformations according to their structural similarity. The central structure of the top five most populated clusters (which accounted for > 99.9% of the total population) was then used as the starting configuration for five independent 100 ns simulations of each peptide at 300 K and 1 bar in the *NPT* ensemble. Accordingly, each peptide was simulated for a total simulation time of 500 ns.

In all MD simulations, all bonds were constrained using the LINCS algorithm[16] and a simulation timestep of 2 fs was used. Periodic boundary conditions were applied in all directions. Lennard-Jones interactions were cutoff at 1.0 nm. Electrostatic interactions were handled using the particle mesh Ewald approach with a real-space cutoff of 1.0 nm. The temperature was controlled using velocity rescaling with a stochastic termwith a time constant of 0.1 ps[17] and the pressure was isotropically maintained at 1 bar using the Parrinello-Rahman barostat with time constant 2.0 ps and compressibility 4.5×10^{-5} bar^{-1}.[18,19] Atomic coordinates were saved every 20 ps for all analysis except the cluster analysis, which was performed on snapshots spaced 40 ps apart to avoid computer memory issues.

To improve the quality of our models of **25** and **13** in DMSO, selected NOE distances from the NMR experiments (see Table S1) were incorporated as distance restraints in the MD simulations. The Gromacs implementation follows that of Torda *et al.*, whereby *time-averaged* distance restraints are used.[20,21] Time-averaged distance restraints provide a better approximation of the physical nature of the NOE

(which may reflect an averaging of multiple conformations) as they enable an atom to satisfy seemingly incompatible distance restraints *on average* by moving between multiple positions.[20,21] If the time-averaged distance between two atoms exceeds the NOE upper bound a harmonic restoring force (the strength of which is controlled by the corresponding force constant for the restraint) will pull the atoms back towards each other. Based on the analysis of the NMR experiments described above, the distance ranges and force constants were 1.8–2.7 Å and 2000 kJ mol^{-1} nm^2 , for strong restraints, 1.8–3.3 Å and 1500 kJ mol^{-1} nm^2 for medium restraints and 1.8–5.0 Å and 1000 kJ mol^{-1} for weak restraints. The time constant for the distance restraints running average was 10 ps.

Assessment of convergence and sampling

Cluster analysis was used to identify distinct peptide conformations from the trajectories. We used the algorithm proposed by Daura *et al.* whereby the root mean square deviation (RMSD) of atom positions between all pairs of structures is determined.[22] For each structure the number of other structures for which the RMSD of the backbone atoms was ≤ 0.05 nm (neighbor conformations) was calculated. The structure with the highest number of neighbors was taken as the center of a cluster and together with its neighbors formed the first cluster. All these structures were eliminated from the pool of structures and the process repeated to find new clusters until the pool of structures was empty. In this way, each structure belonged to only one cluster. To assess whether the sampling was adequate in our simulations, we calculated the number of clusters as a function of the total simulation time (*i.e.* the independent trajectories were joined together). A total of 5 clusters are identified for the parent cyclic peptide **25**, and no new structures are found after the third independent simulation (after approx. 250 ns total simulation time, Figure S9a). On the other hand, only 4 clusters are identified for the OMCP **13**, and it also takes three independent simulations (and approx. 200 ns total simulation time) for the number of new structures to converge (see Figure S9b). The relative population of each cluster with increasing simulation time is shown in Figure S10. It can be seen that the cluster populations have converged with the last 180 ns of simulation essentially contributing no new structural information.

Figure S9. Number of clusters over time as the five independent 100 ns simulations are added to the trajectory for (a) the parent cyclic peptide **25** and (b) the OMCP peptide **13**.

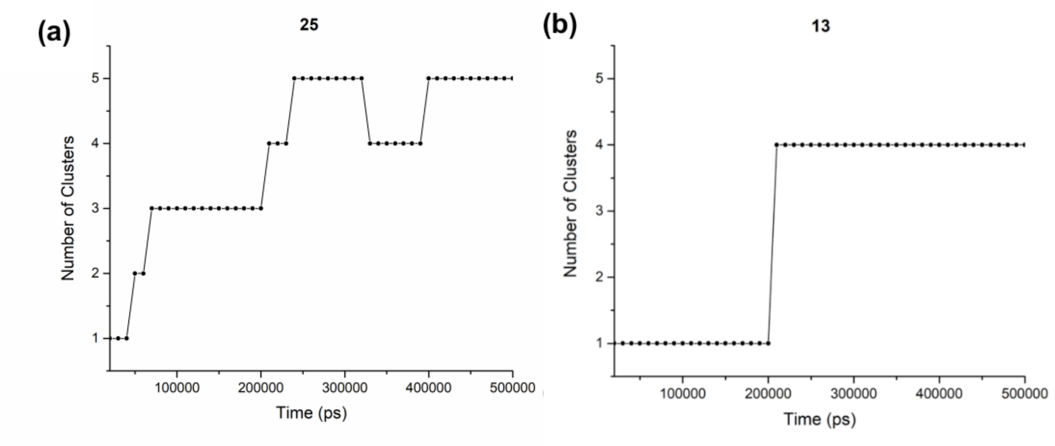

Figure S10. Population (% of total) of each cluster as the 5 independent 100 ns simulations are added to the trajectory for (a) the parent cyclic peptide **25** and (b) the OMCP **13**. Data are overlaid going from the lightest shade of blue 0–20 ns to the darkest shade of blue 0–500 ns.

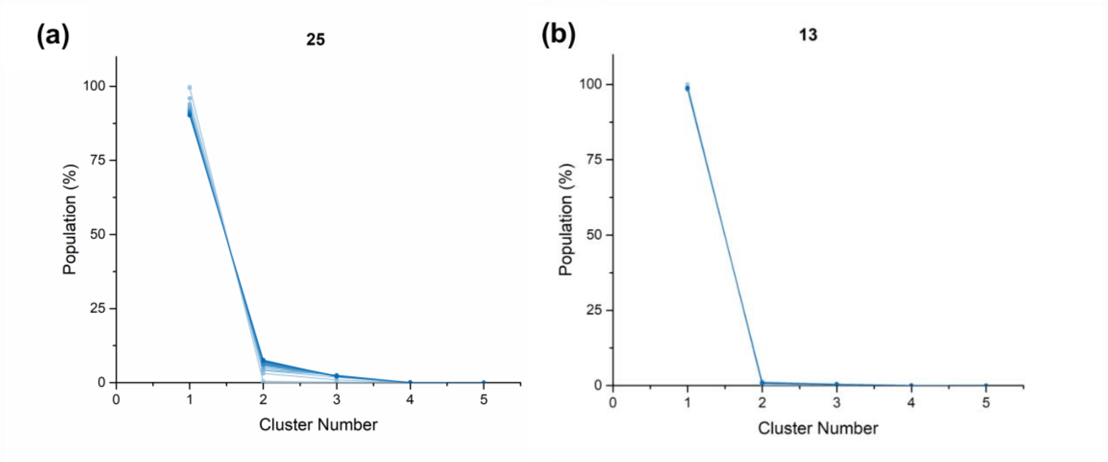

Simulation Results

Cluster Analysis

Representative structures of the three most populated clusters of **25** and **13** are shown in Figure S11. For both **13** and **25**, the three most populated clusters represent ≥ 99.9% of all conformations sampled. Ten representative structures from Cluster 1 (the most populated cluster) of both peptides were overlaid, showing the range of conformations explored by the side chains during the simulations. It is noted that the strong NOE restraint in **13** restricts the mobility of the Tyr side chain compared to **25**. Oxetane introduction facilitates an inversion of the amide bond between Ala4 and Tyr5, which allows the structure to be stabilised by an intra-peptide hydrogen bond across the macrocycle (see main paper and below).

Figure S11. Snapshots of the central structure of each of the three most populated clusters. LHS: **25**, with carbon atoms colored cyan; RHS: **13** with carbon atoms colored green.

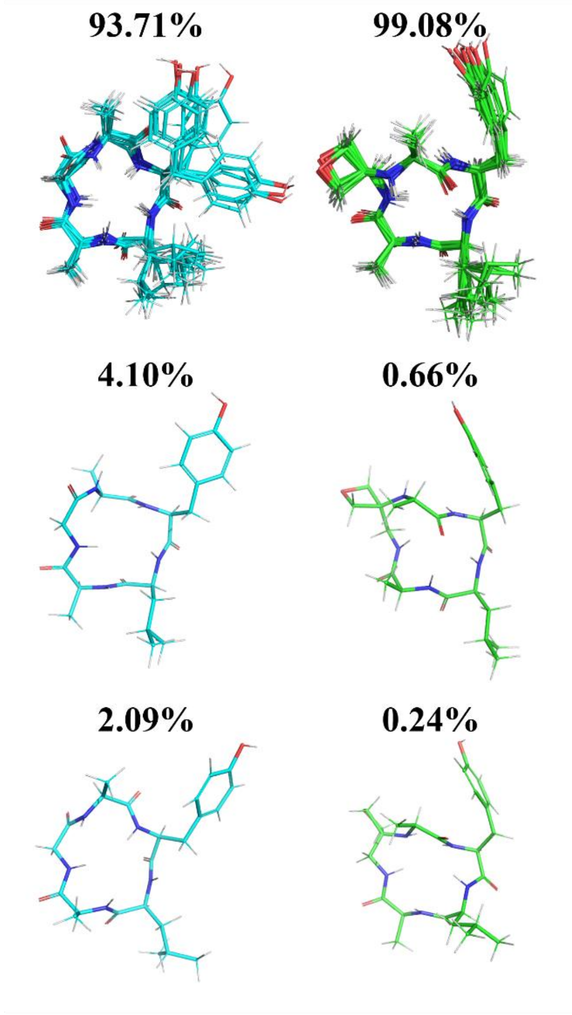

Hydrogen bonding

Analysis of the MD simulations was carried out on the entire 500 ns trajectory. Hydrogen bonds were identified using geometric criteria whereby a hydrogen bond is said to exist if the donor---acceptor distance ≤ 3.5 Å and the angle donor-hydrogen---acceptor ≤ 35°. On average both peptides form approximately five hydrogen bonds in total, with **25** forming five peptide-solvent hydrogen bonds and **13** forming four peptide-solvent hydrogen bonds and one intra-peptide hydrogen bond (Table S3). The percentage occupancy of all intra-peptide hydrogen bonds is given in Table S4. As mentioned above, intra-peptide hydrogen-bonding is negligible in the parent cyclic peptide **25**; however in the OMCP **13** there is on average approximately one intra-peptide hydrogen bond between the NH group of Leu1 and the O atom of Ala4, which may contribute to the rigidity of the macrocycle.

Table S3. Average number of peptide-solvent and intra-peptide hydrogen bonds formed by **25** and **13** in DMSO.

Peptide	Number of intra-peptide hydrogen bonds	Number of peptide-solvent hydrogen bonds	Total number of hydrogen bonds
**25**	0.002	5.034	5.036
**13**	0.966	4.029	4.995

Table S4. Occupancy of intra-peptide hydrogen bonds for **25** and **13** in DMSO.

Peptide	Donor Atom	Acceptor Atom	% Occupancy
**25**	Gly3N	Leu1O	0.02
**13**	Leu1N	Ala4O	86.30
	Gly3N	Ala4O	0.03

8. References

[1] P.-Y. Yang, M. Wang, L. Li, H. Wu, C. Y. He and S. Q. Yao, *Chem. Eur. J.*, 2012, **18**, 6528.

[2] N. H. Powell, G. J. Clarkson, R. Notman, P. Raubo, N. G. Martin and M. Shipman, *Chem. Commun.*, 2014, **50**, 8797.

[3] R. Pajewski, R. Ferdani, J. Pajewska, R. Li and G. W. Gokel, *J. Am. Chem. Soc.*, 2005, **127**, 1828.

[4] J. D. Beadle, PhD thesis, University of Warwick, 2018.

[5] S. Gangwar, G. M. Pauletti, T. J. Siahaan, V. J. Stella and R. T. Borchardt, *J. Org. Chem.*, 1997, **62**, 1356.

[6] Y. Tang, H. Xie, G. Tian and Y. Ye, *J. Peptide Res.*, 2002, **60**, 95.

[7] O. E. Jensen and S.-O. Lawesson, *Tetrahedron*, 1985, **41**, 5595.

[8] J. D. Beadle, A. Knuhtsen, A. Hoose, R. Raubo, A. G. Jamieson and M. Shipman, *Org. Lett.*, 2017, **19**, 3303.

[9] T. Haag, R. A. Hughes, G. Ritter and R. R. Schmidt, *Eur. J. Org. Chem.*, 2007, 6016.

[10] A. Graziadio, M. Zanda, S. Frau, I. N. Fleming, M. Musolino, S. Dall'Angelo, M. Baldassarre and M. Piras, *Bioconjugate Chem.*, 2016, **27**, 1332.

[11] *Avogadro: An open-source molecular builder and visualisation tool*, Version 1.1.0; http://avogadro.cc/.

[12] M. D. Hanwell, D. E. Curtis, D. C. Lonie, T. Vandermeersch, E. Zurek and G. R. Hutchison, *J. Cheminform.*, 2012, **4**, 17.

[13] A. K. Rappe, C. J. Casewit, K. S. Colwell, W. A. Goddard and W. M. Skiff, *J. Am. Chem. Soc.*, 1992, **114**, 10024.

[13] M. J. Abraham, T. Murtola, R. Schulz, S. Páll, J. C. Smith, B. Hess and E. Lindahl, *SoftwareX*, 2015, **1-2**, 19.

[14] A. D. MacKerell, B. R. Brooks, C. L. Brooks, L. Nilsson, B. Roux, Y. Won and M. Karplus, *Encyclopedia of Computational Chemistry*, John Wiley & Sons, Hoboken, 1988, vol. 1.

[15] M. L. Strader and S. E. Feller, *J. Phys. Chem. A*, 2002, **106**, 1074.

[16] B. Hess, C. Kutzner, D. van der Spoel and E. Lindahl, *J. Chem. Theory Comput.*, 2008, **4**, 435.

[17] G. Bussi, D. Donadio and M. Parrinello, *J. Chem. Phys.*, 2007, **126**, 014101.

[18] M. Parrinello and A. Rahman, *J. Appl. Phys.* 1981, **52**, 7182.

[19] S. Nose and M. L. Klein, *Mol. Phys.* 1983, **50**, 1055.

[20] A. E. Torda, R. M. Scheek and W. F. van Gunsteren, *Chem. Phys. Lett.*, 1989, **157**, 289.

[21] M. J. Abraham, D. van der Spoel, E. Lindahl, B. Hess and the GROMACS development team, Gromacs User Manual Version 5.1.4. www.gromacs.org, 2016.

[22] X. Daura, K. Gademann, B. Jaun, D. Seebach, W. F. van Gunsteren and A. E. Mark, *Angew. Chem. Int. Ed.* 1999, **38**, 236.

9. ^{1}H NMR and ^{13}C NMR spectra

Boc-Pro-Tyr(Bn)-OBn (27)

^{1}H NMR (600 MHz, DMSO-*d6* @ 373 K)

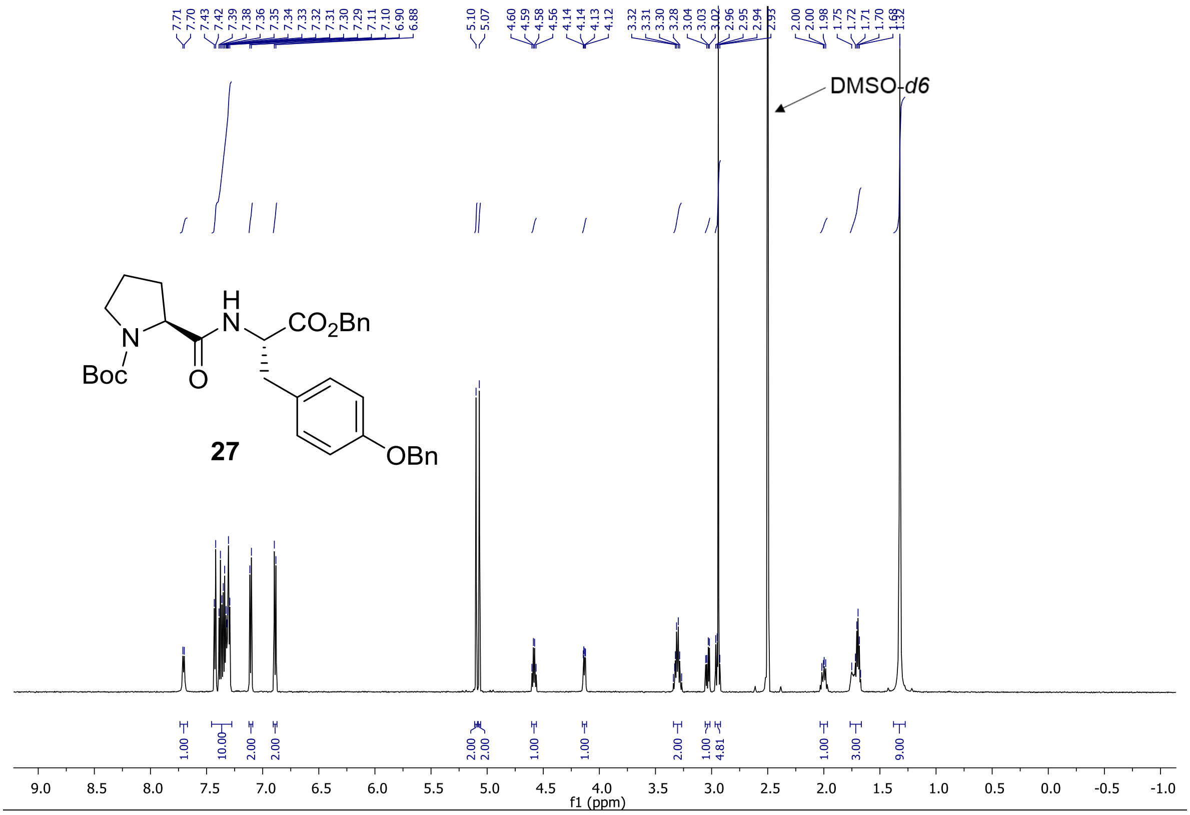

^{13}C NMR (151 MHz, DMSO-*d6*)

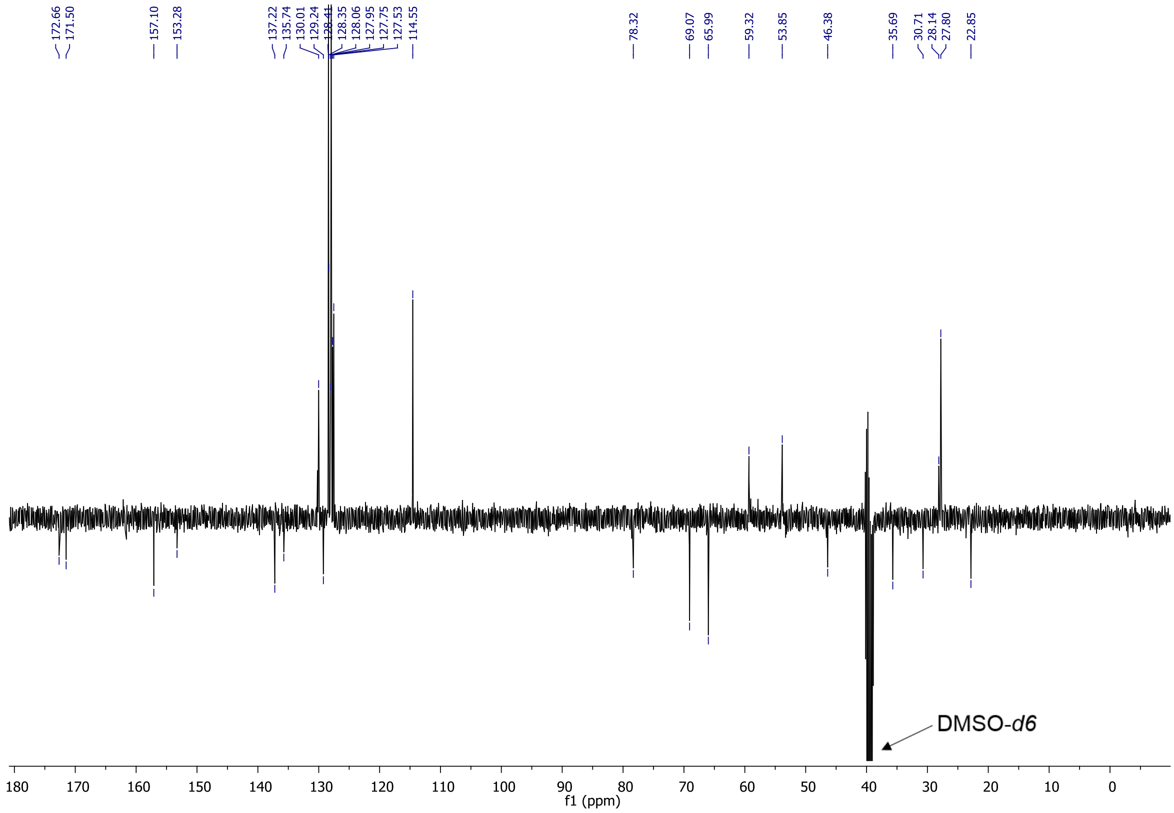

NO_2-GOx-Pro-Tyr(Bn)-OBn (28)

^{1}H NMR (500 MHz, $CDCl_3$)

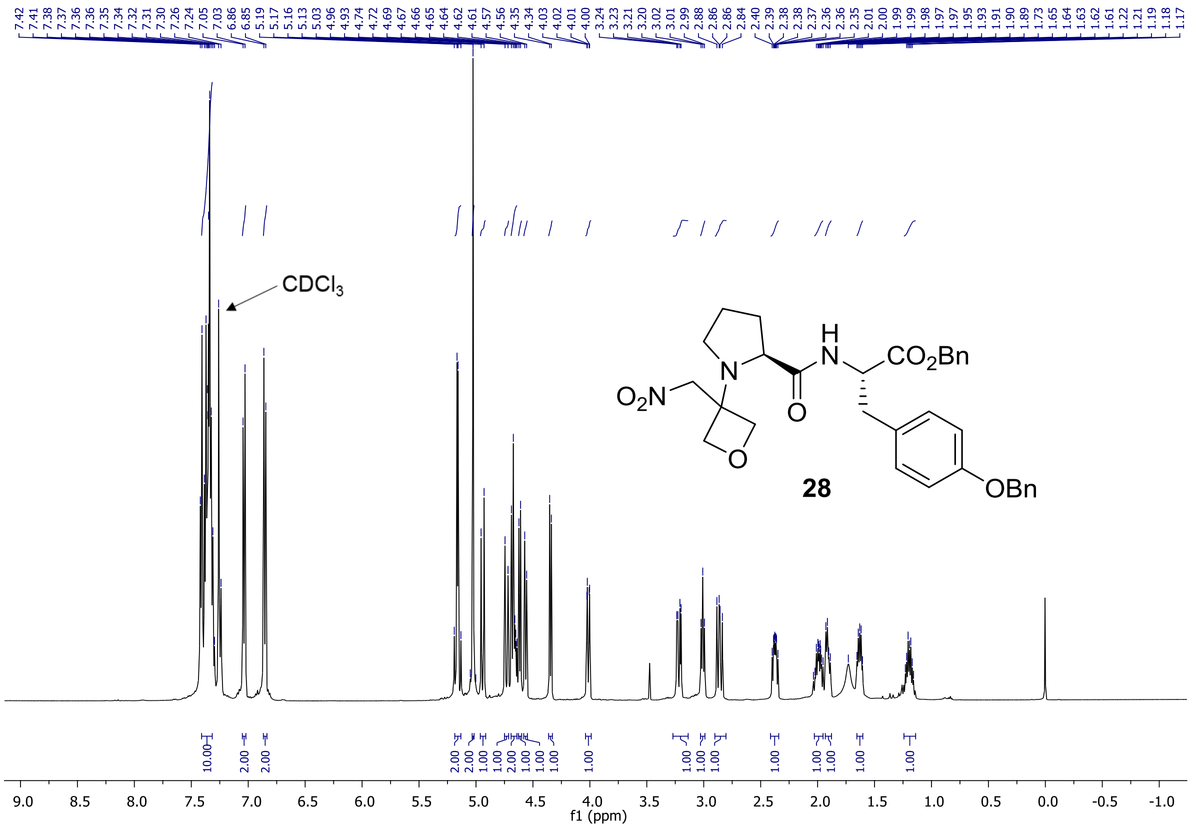

^{13}C NMR (126 MHz, $CDCl_3$)

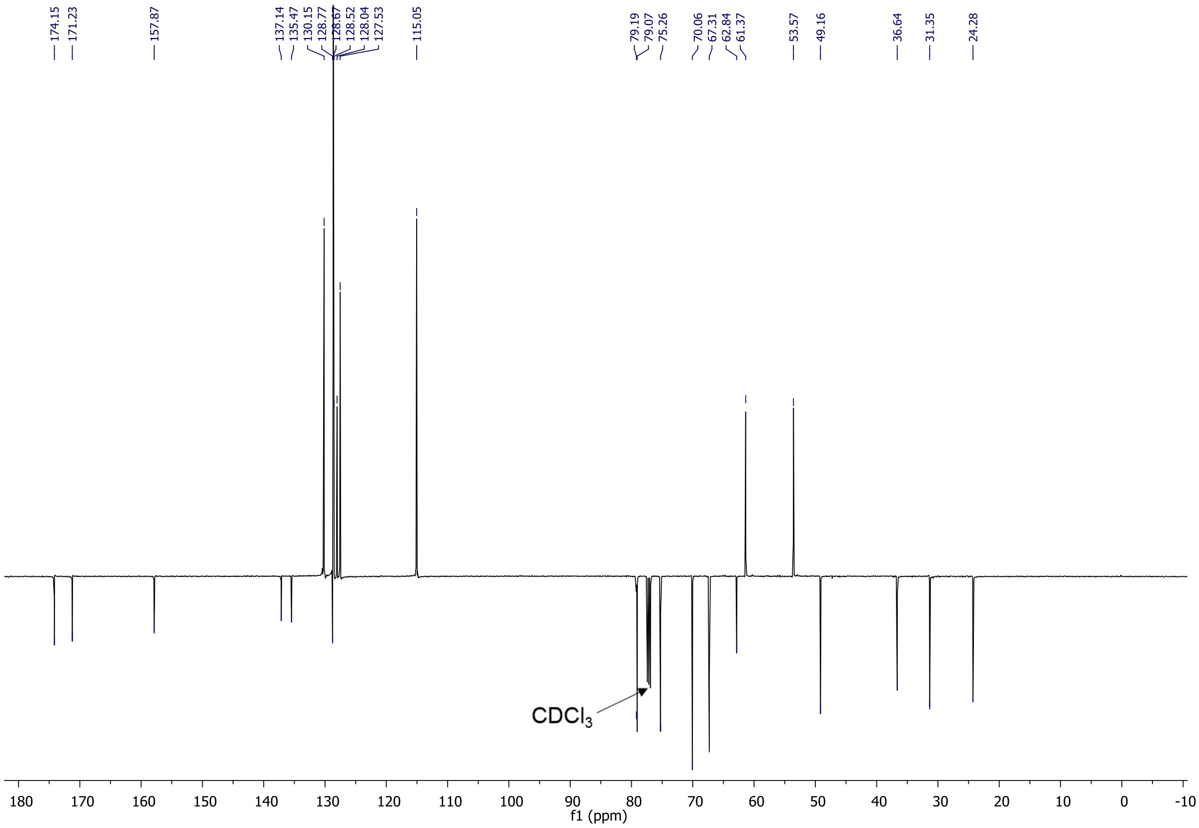

Boc-Ala-GOx-Pro-Tyr(Bn)-OBn (29)

^{1}H NMR (500 MHz, $CDCl_3$)

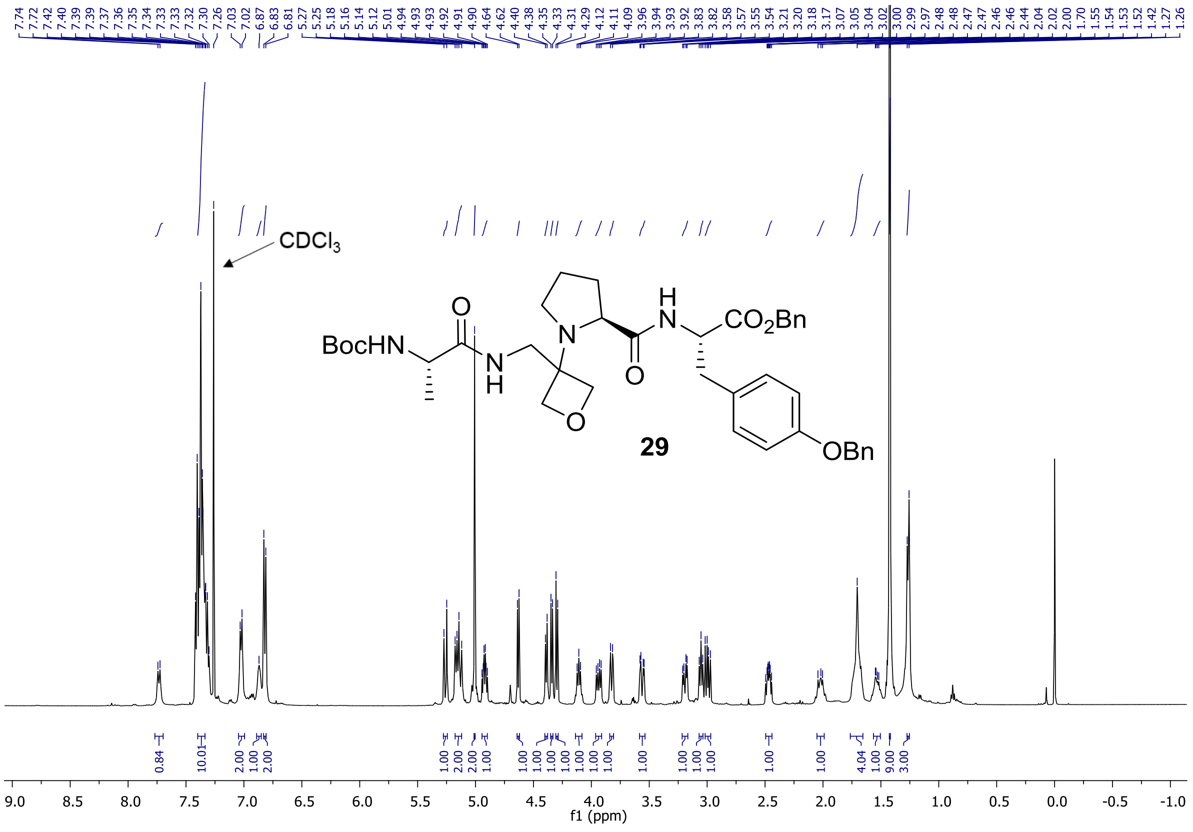

^{13}C NMR (126 MHz, $CDCl_3$)

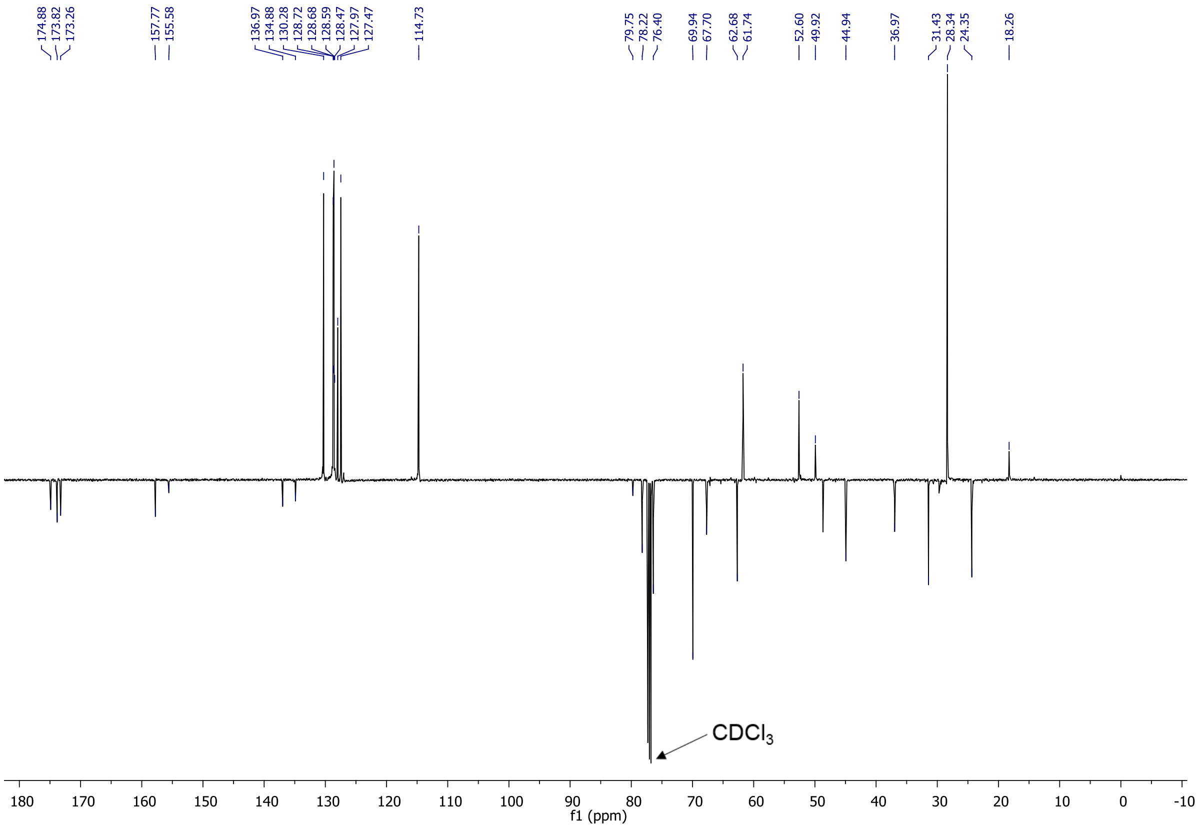

Cbz-Leu-Ala-GOx-Pro-Tyr(Bn)-OBn (30)

^{1}H NMR (500 MHz, $CDCl_3$)

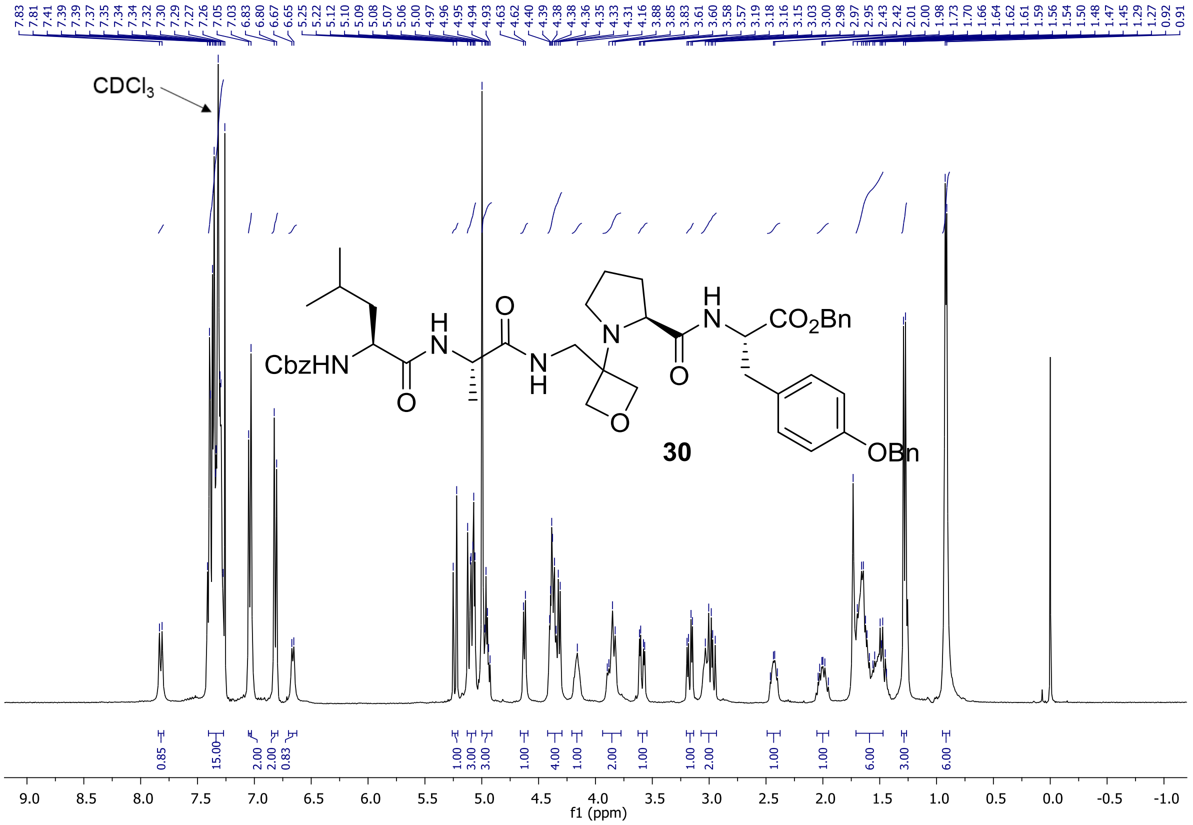

^{13}C NMR (126 MHz, $CDCl_3$)

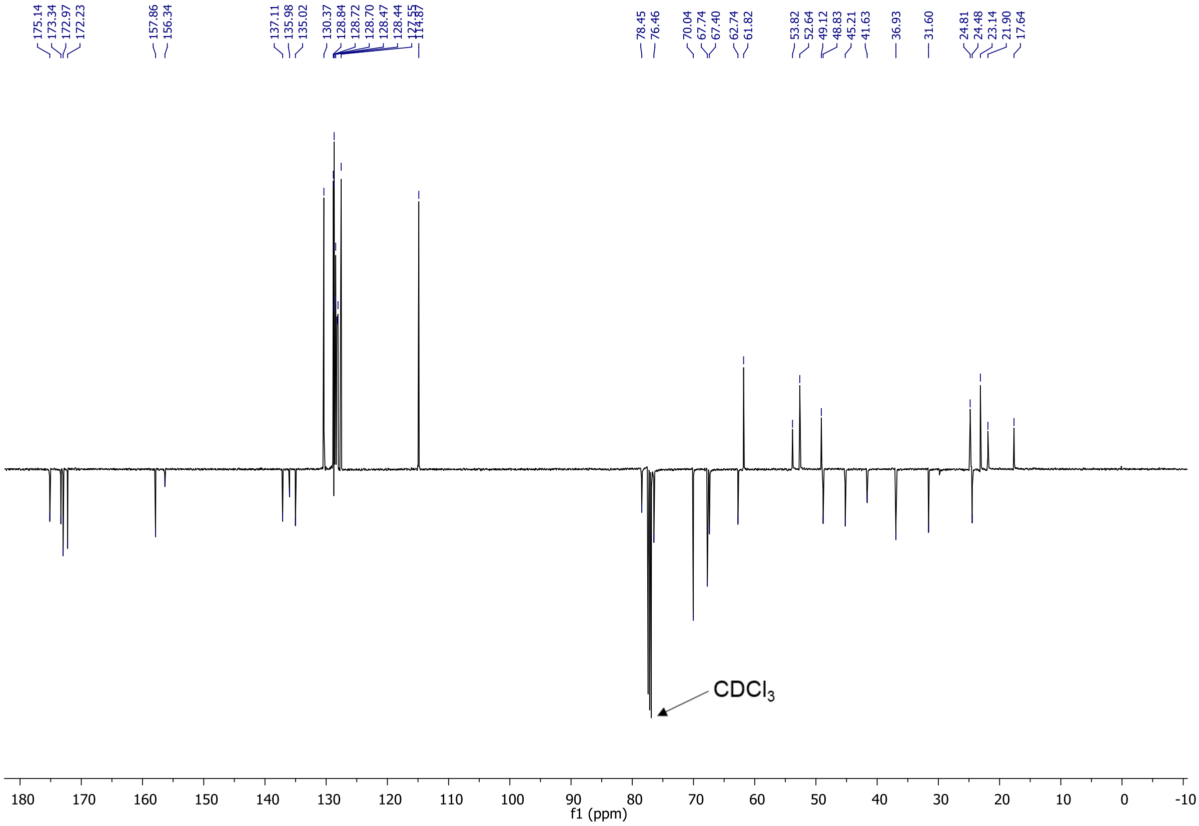

H-Leu-Ala-GOx-Pro-Tyr-OH (1)

^{1}H NMR (500 MHz, CD_3OD)

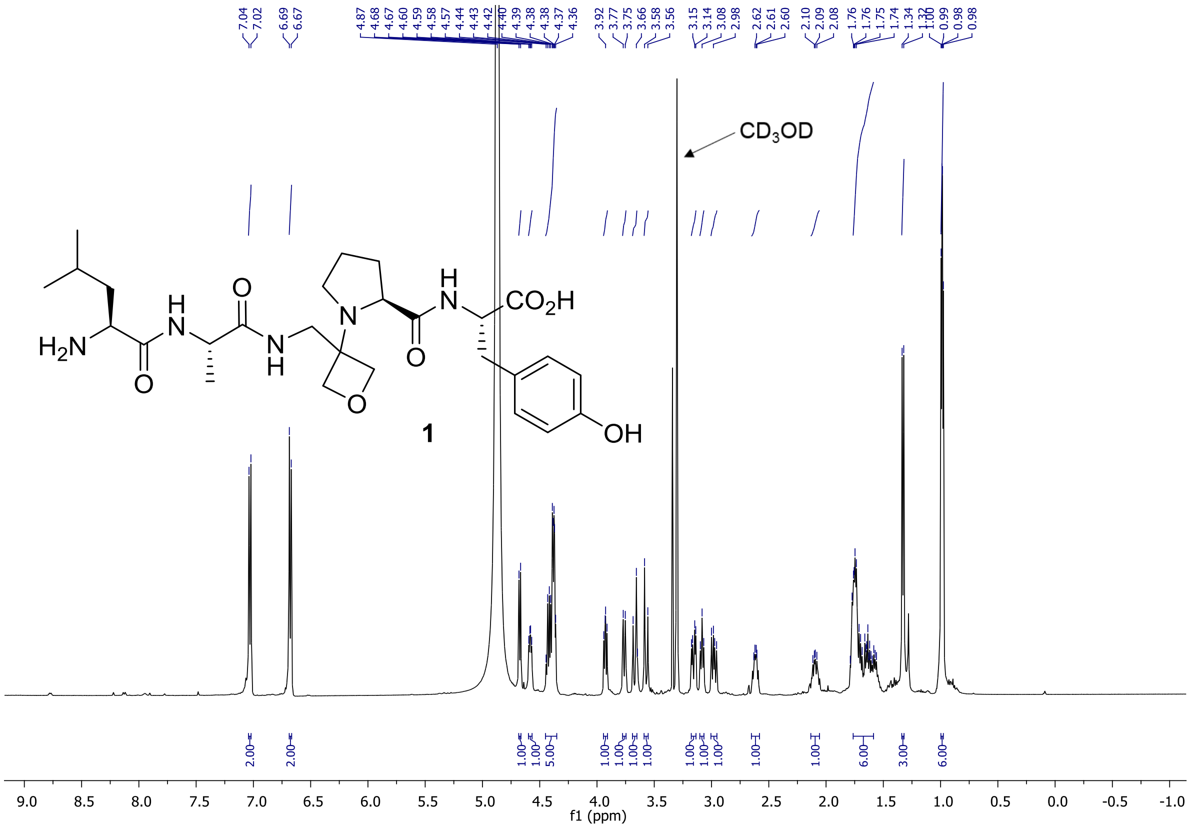

^{13}C NMR (126 MHz, CD_3OD)

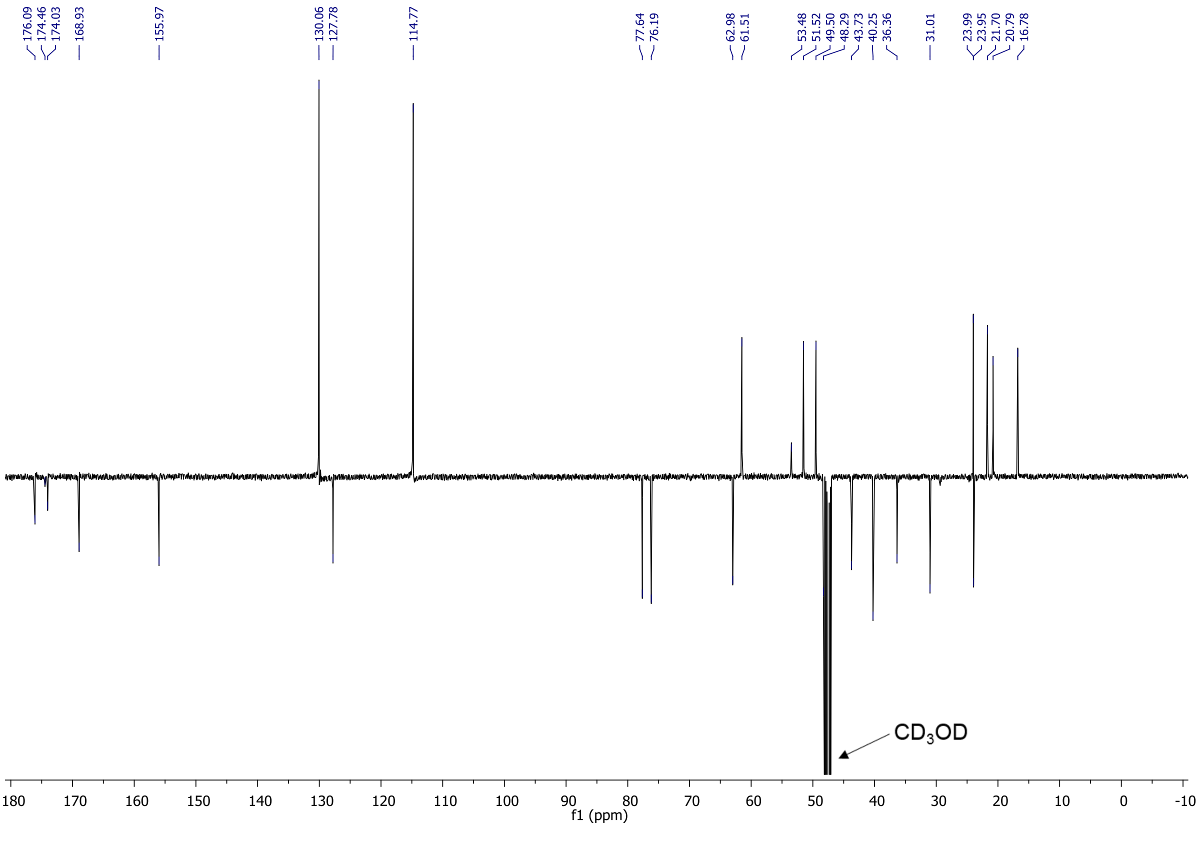

Cyclo(Leu-Ala-GOx-Pro-Tyr) (4)

^{1}H NMR (500 MHz, CD_3OD)

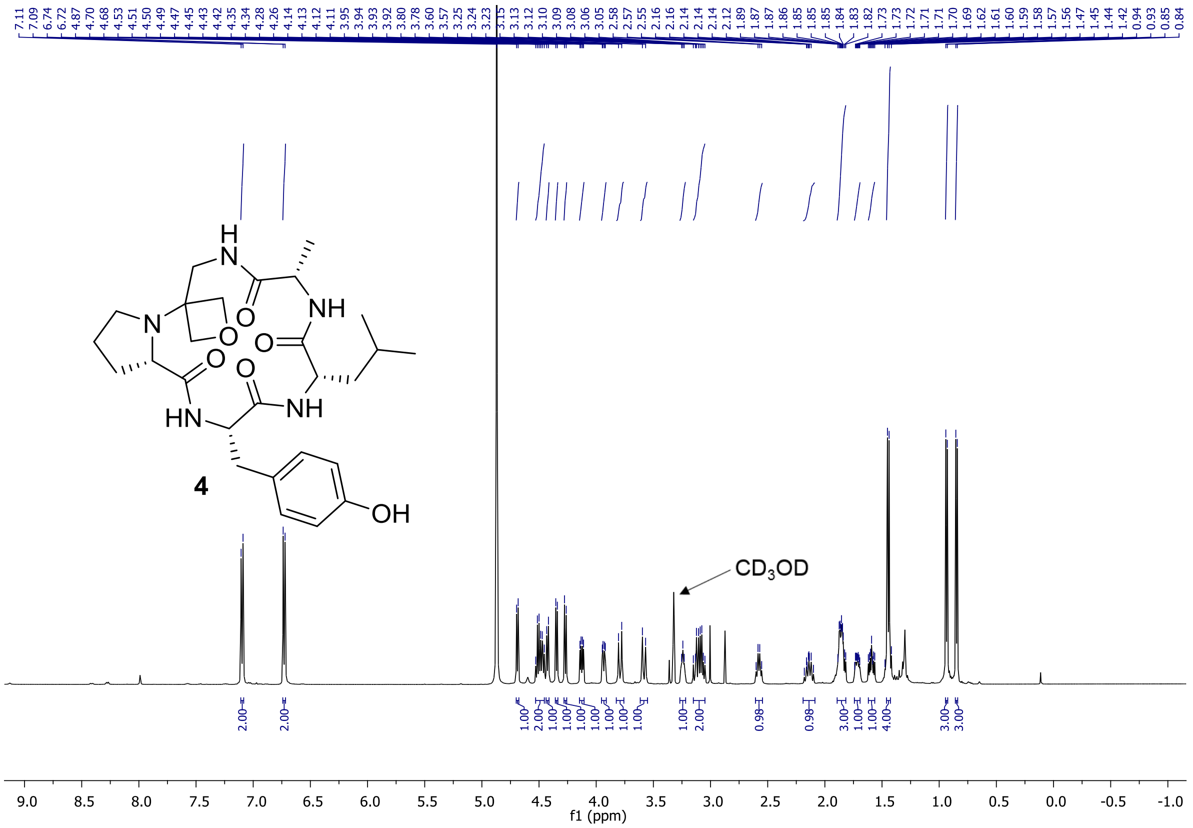

^{13}C NMR (126 MHz, CD_3OD)

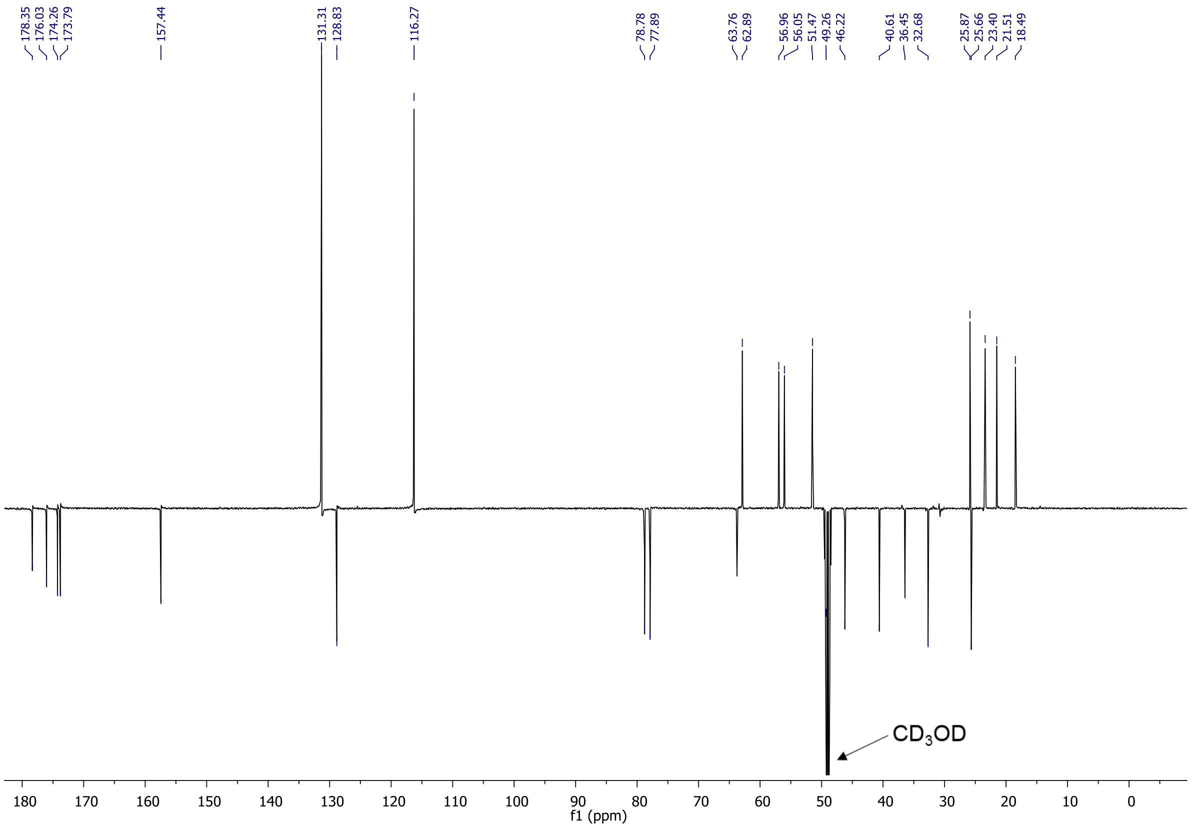

Boc-Gly-Pro-Tyr(Bn)-OBn (31)

^{1}H NMR (500 MHz, $CDCl_3$)

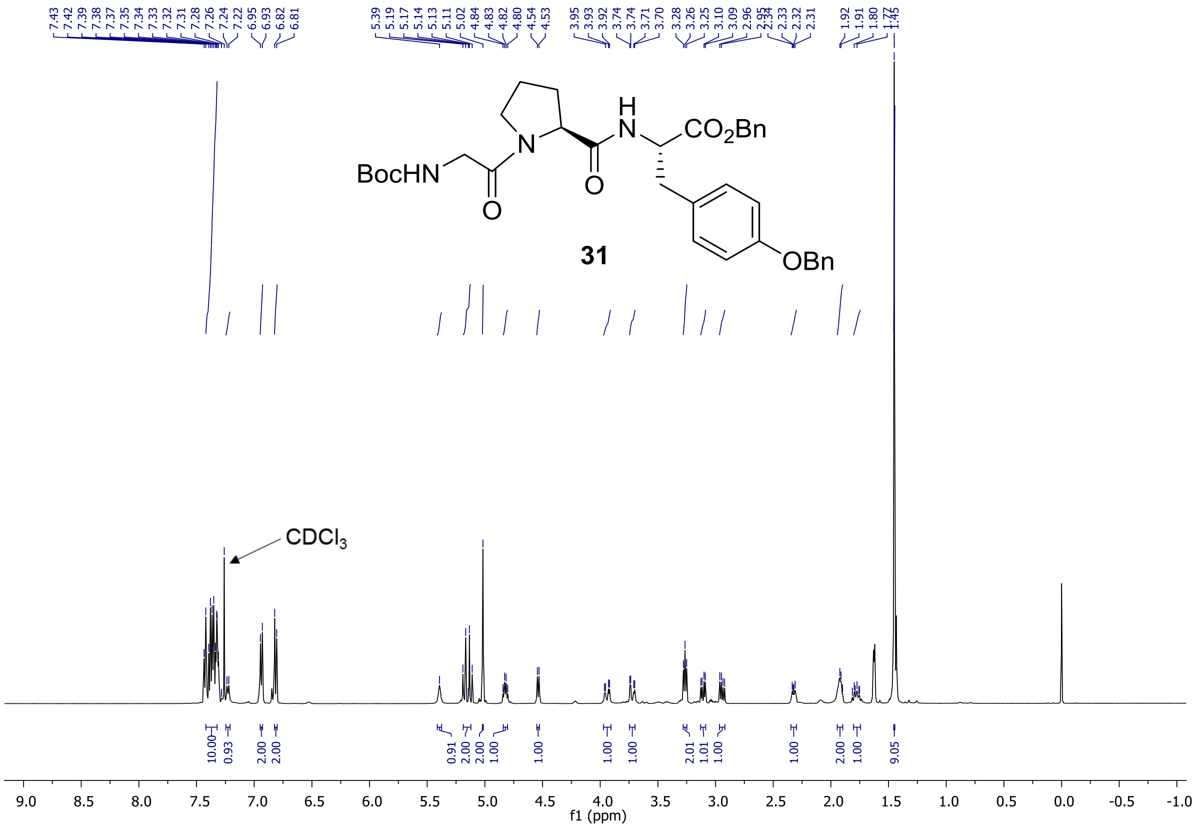

^{13}C NMR (126 MHz, $CDCl_3$)

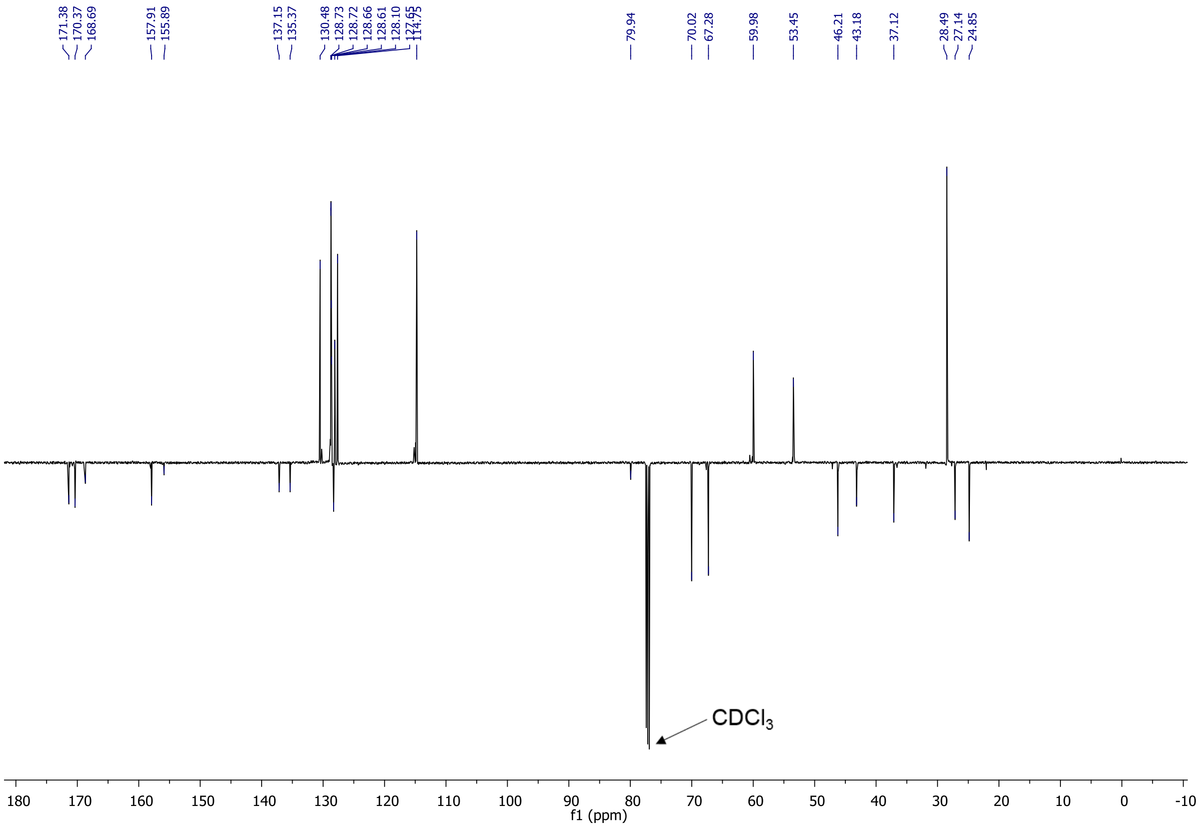

Boc-Ala-Gly-Pro-Tyr(Bn)-OBn (32)

^{1}H NMR (500 MHz, $CDCl_3$)

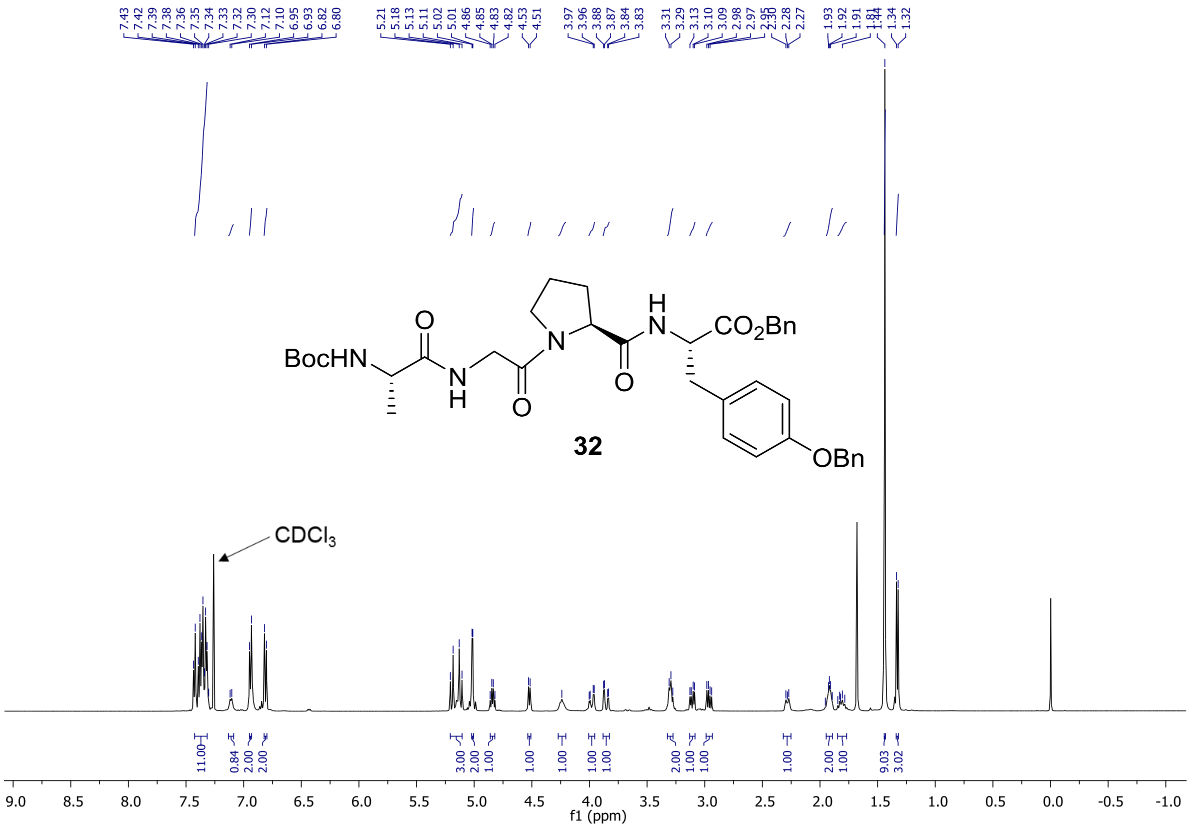

^{13}C NMR (126 MHz, $CDCl_3$)

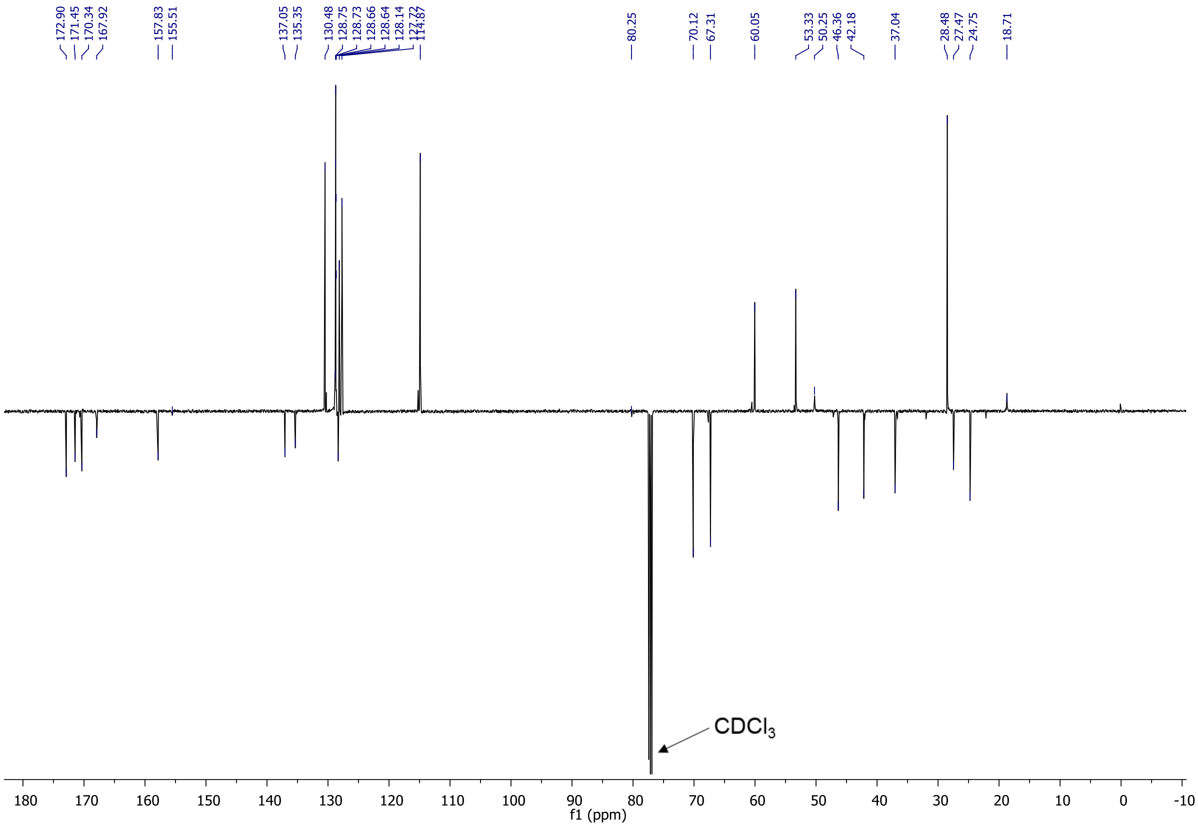

Cbz-Leu-Ala-Gly-Pro-Tyr(Bn)-OBn (33)

^{1}H NMR (600 MHz, DMSO-d_6 @ 373 K)

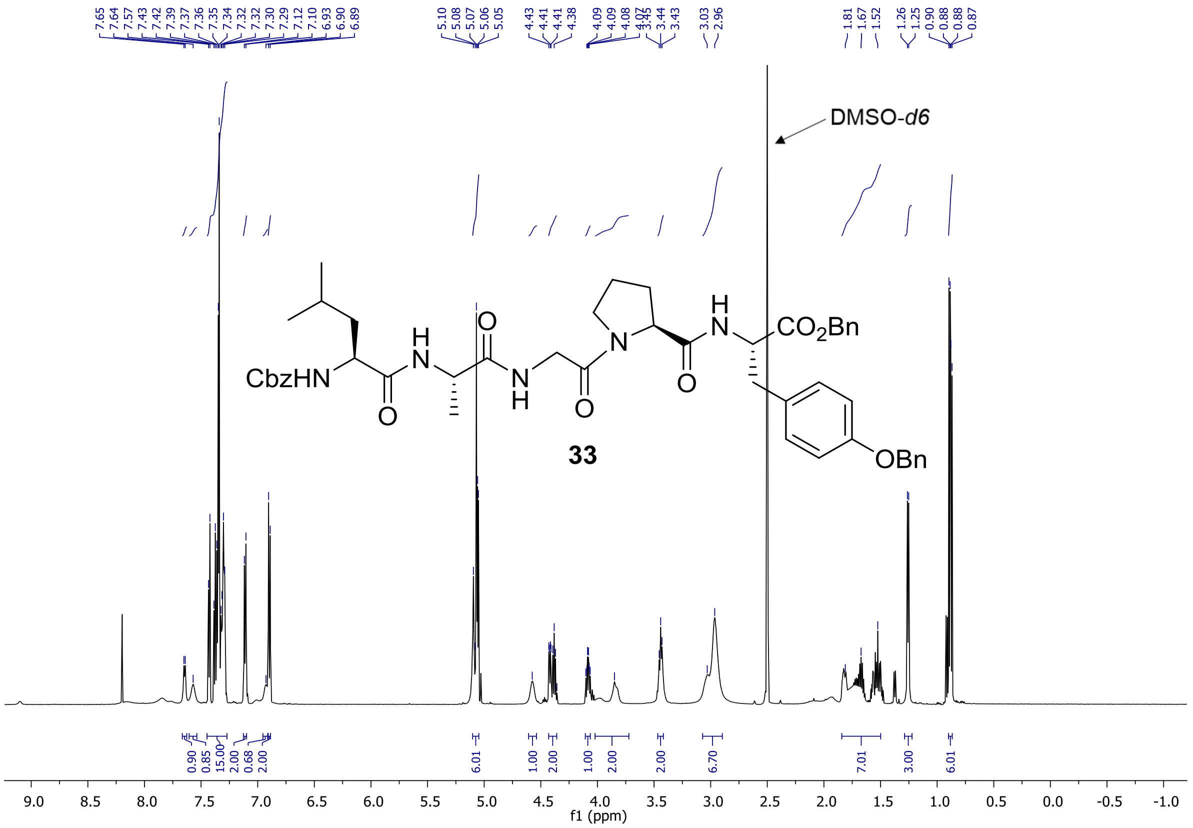

^{13}C NMR (151 MHz, DMSO-d_6 @ 373 K)

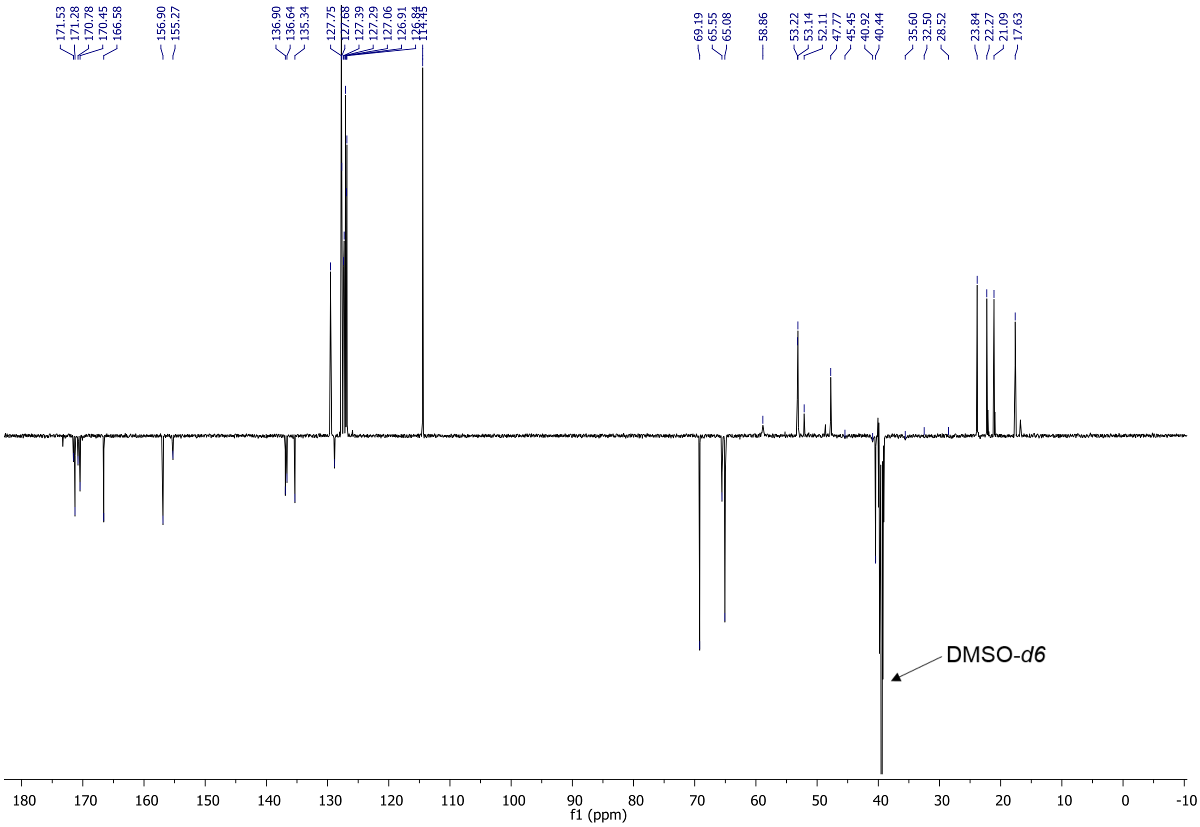

H-Leu-Ala-Gly-Pro-Tyr-OH (3)

^{1}H NMR (600 MHz, DMSO-d_6 @ 373 K)

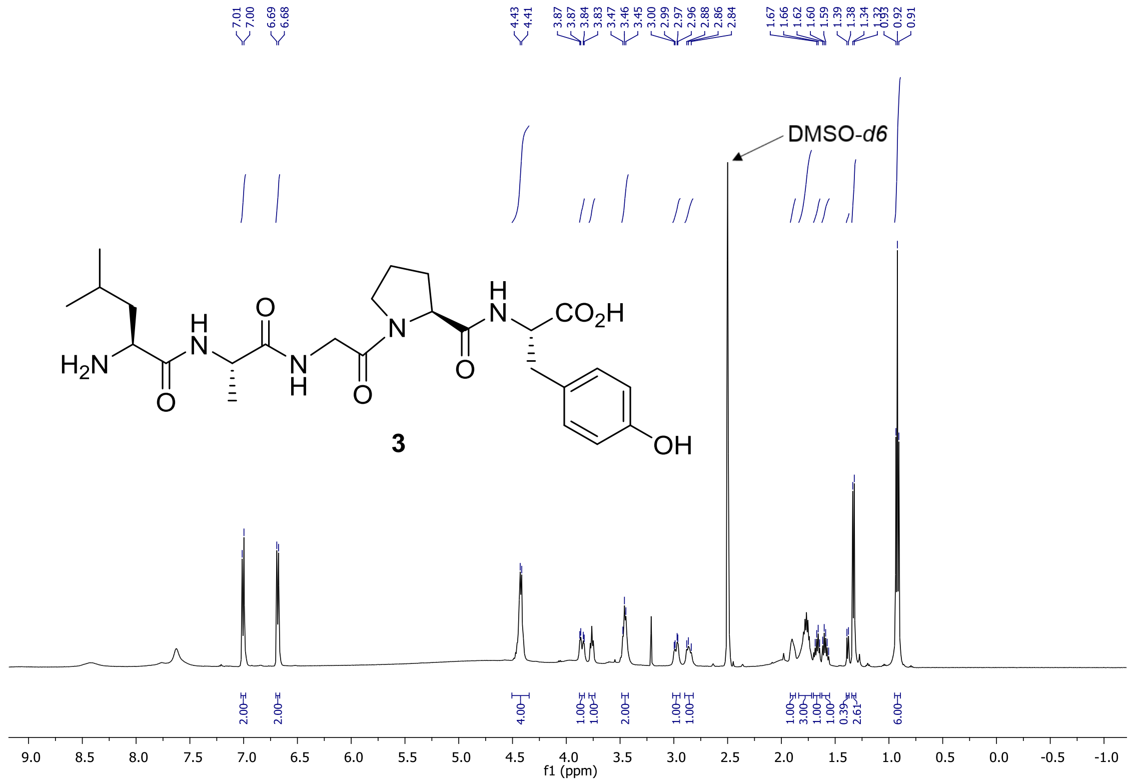

^{13}C NMR (151 MHz, DMSO-d_6 @ 373 K)

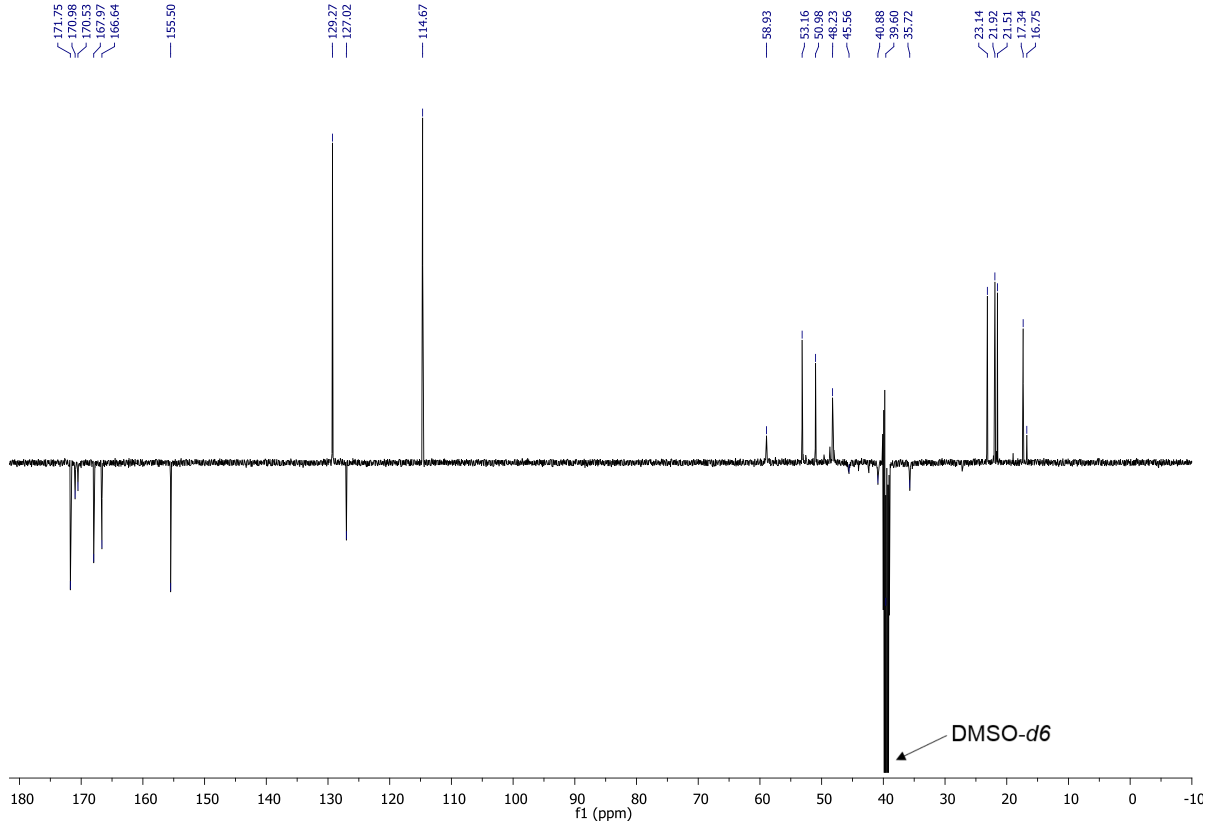

Cyclo(Leu-Ala-Gly-Pro-Tyr) (5)

^{1}H NMR (500 MHz, CD_3OD)

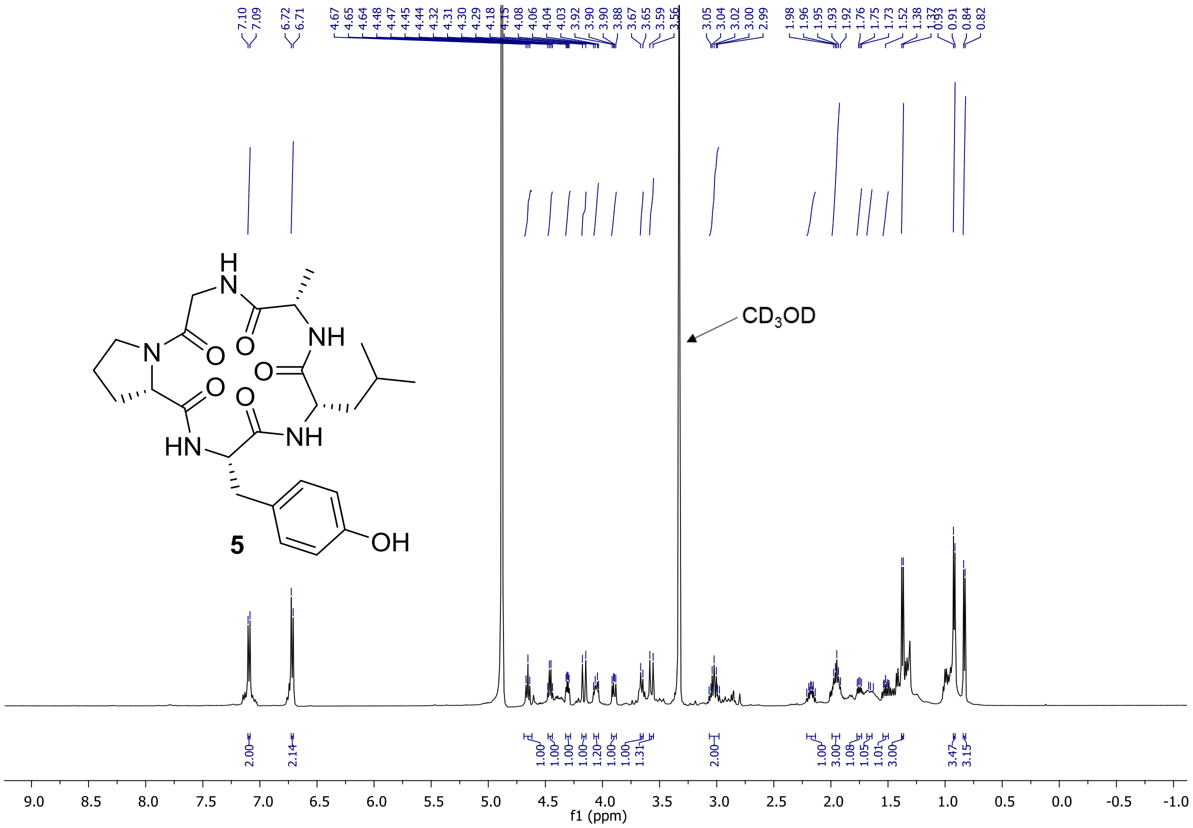

^{13}C NMR (126 MHz CD_3OD)

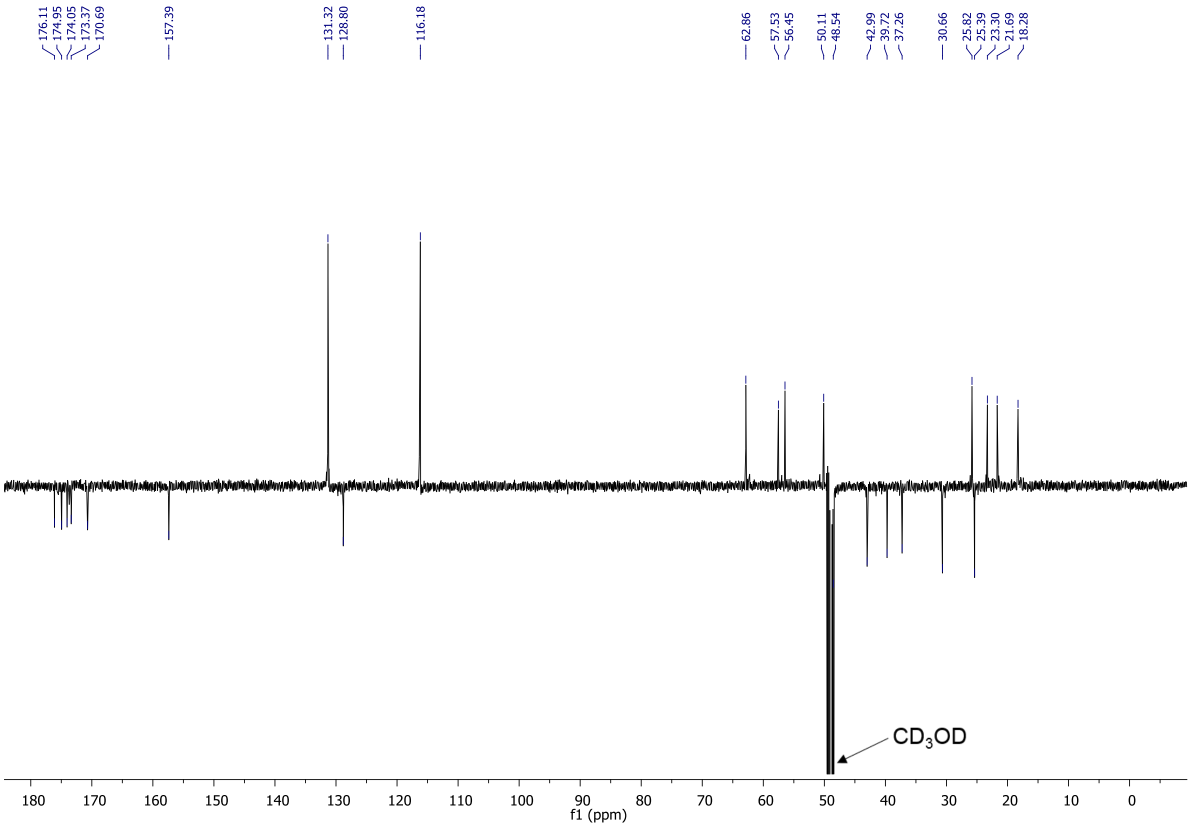

Boc-Leu-GOx-Gly-OBn (34)

^{1}H NMR (500 MHz, $CDCl_3$)

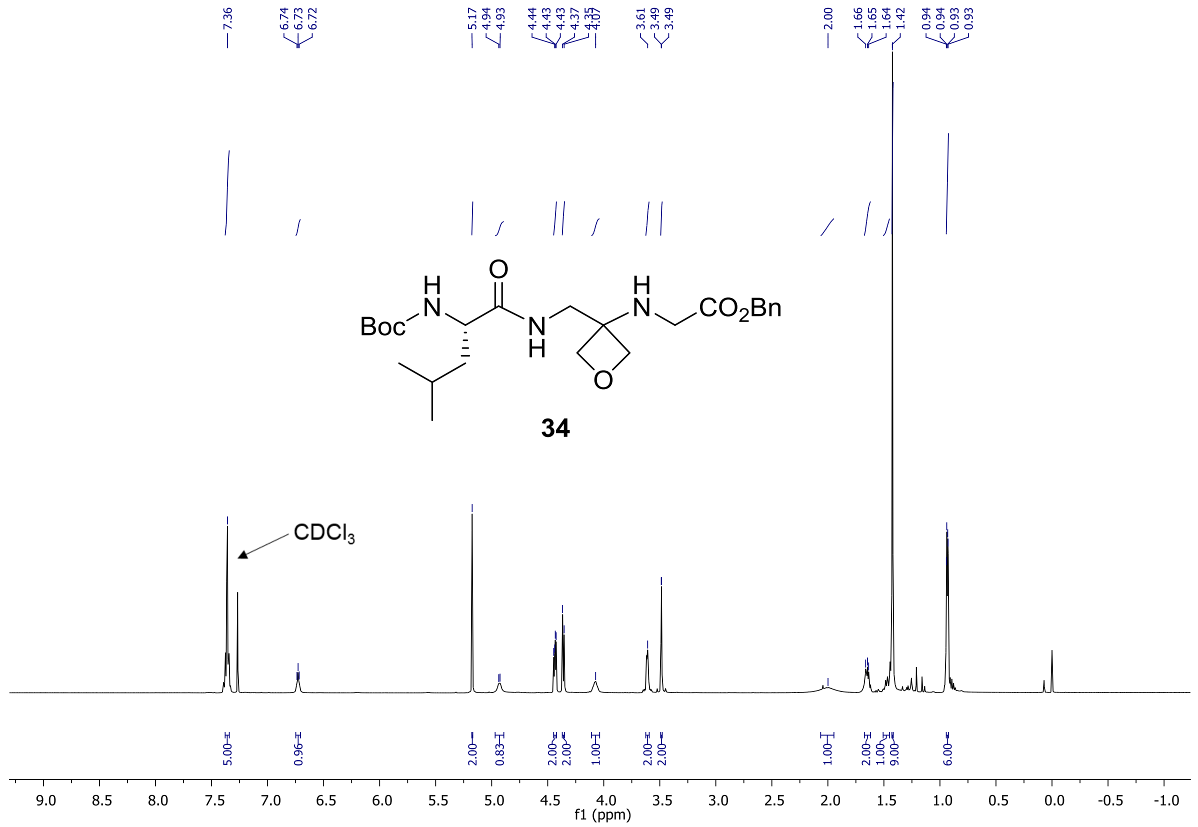

^{13}C NMR (101 MHz, $CDCl_3$)

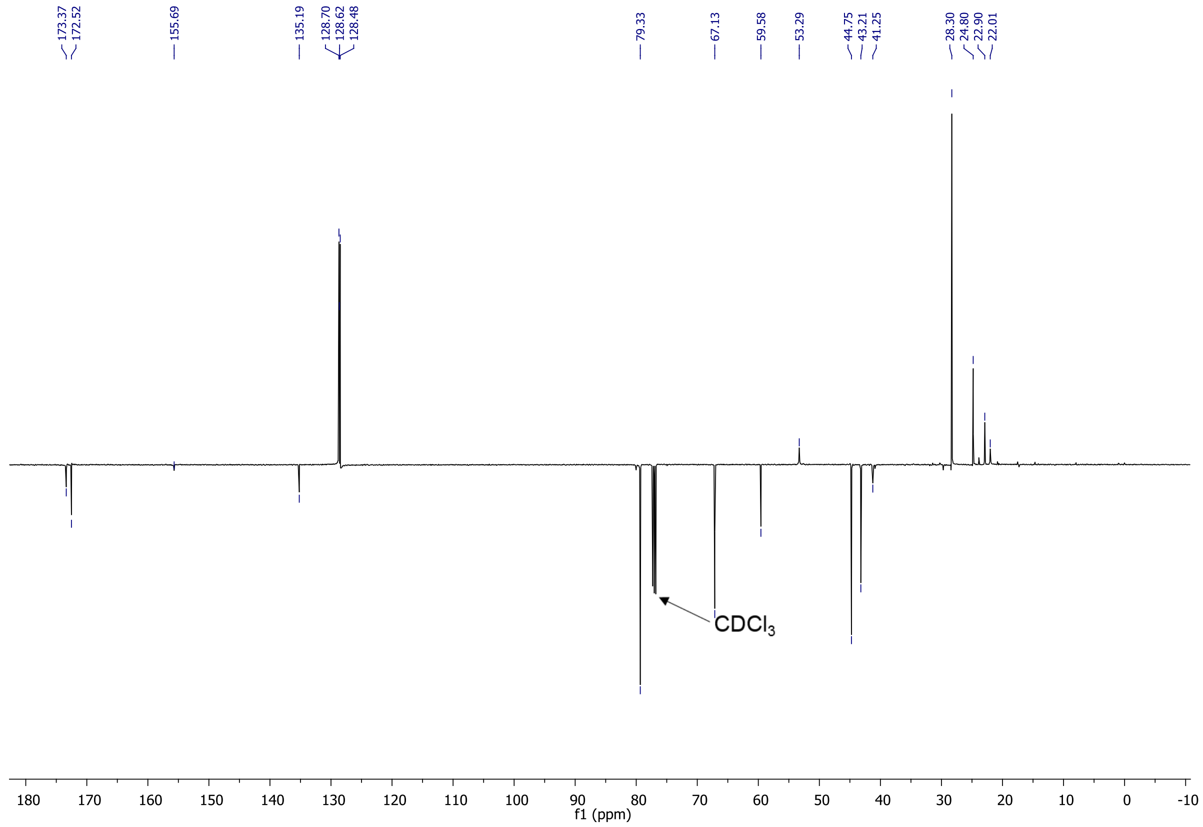

Cbz-Trp-Leu-GOx-Gly-OBn (35)

^{1}H NMR (500 MHz, $CDCl_3$)

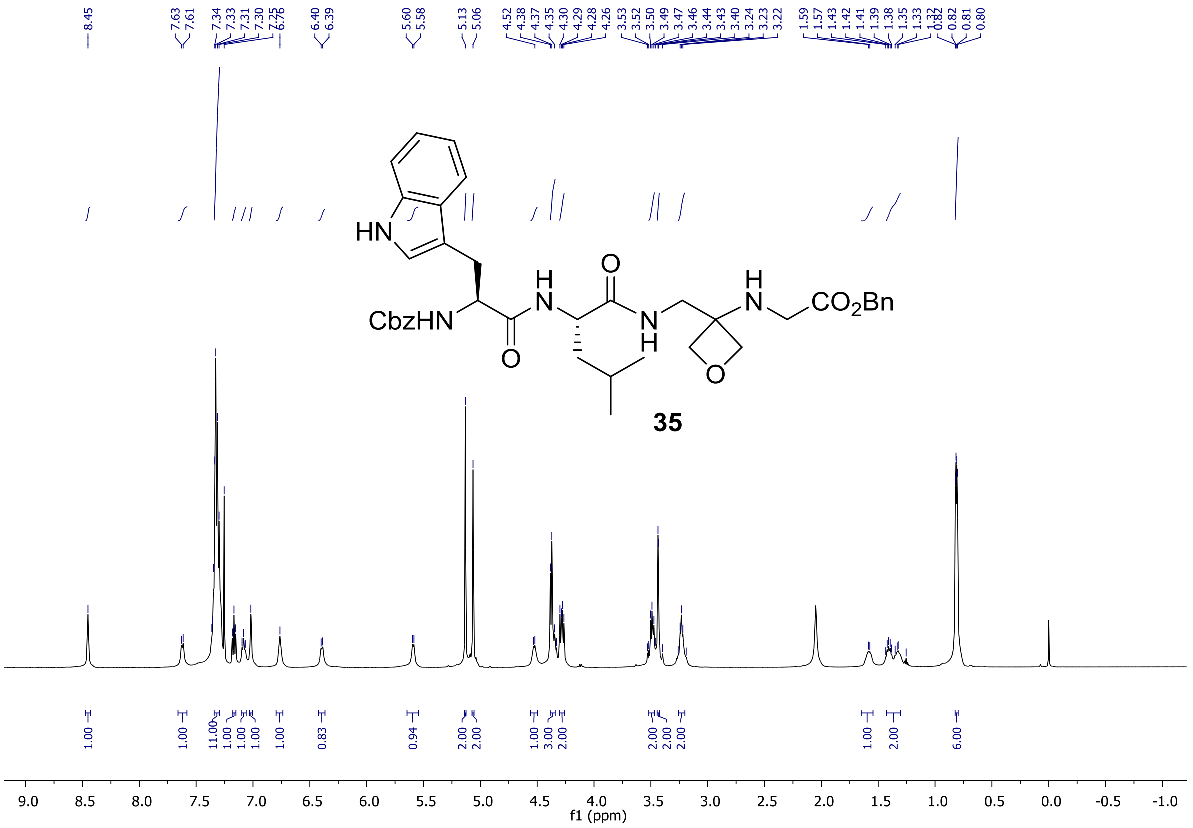

^{13}C NMR (126 MHz, $CDCl_3$)

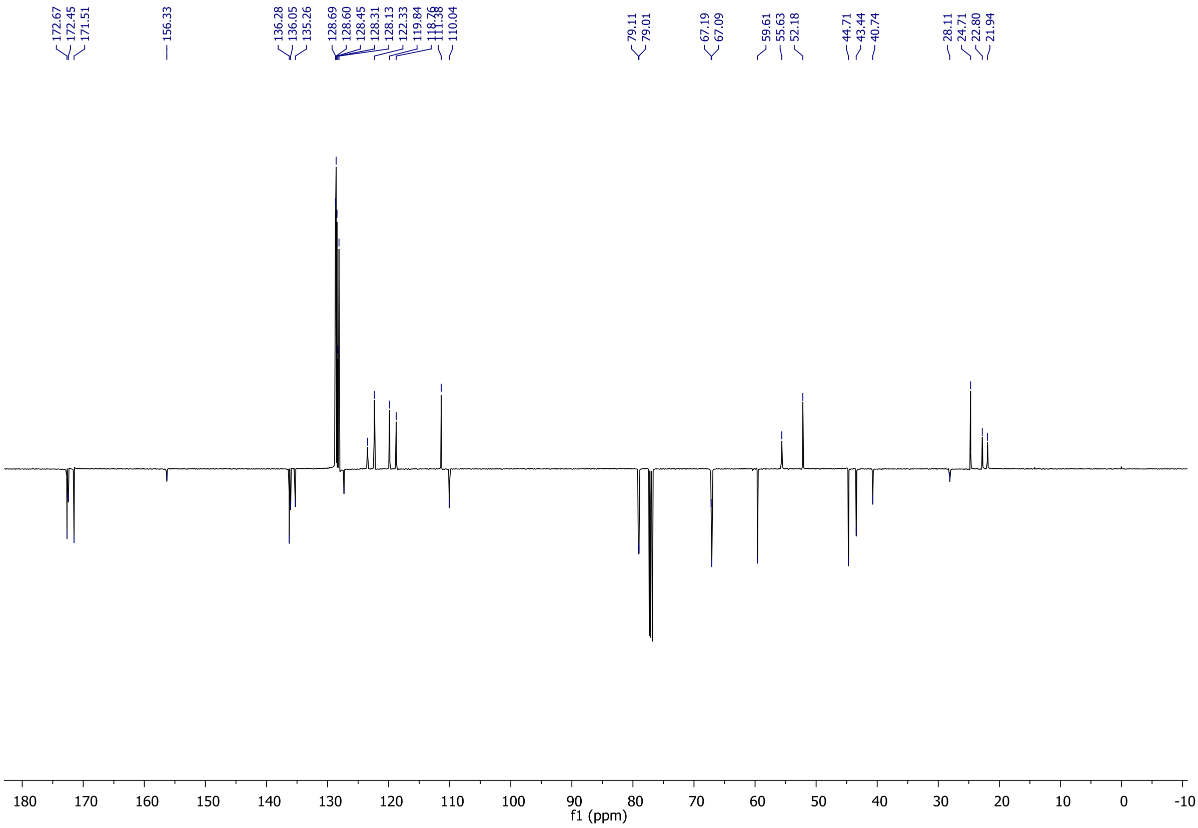

H-Trp-Leu-GOx-Gly-OH (7)

^{1}H NMR (500 MHz, D_2O)

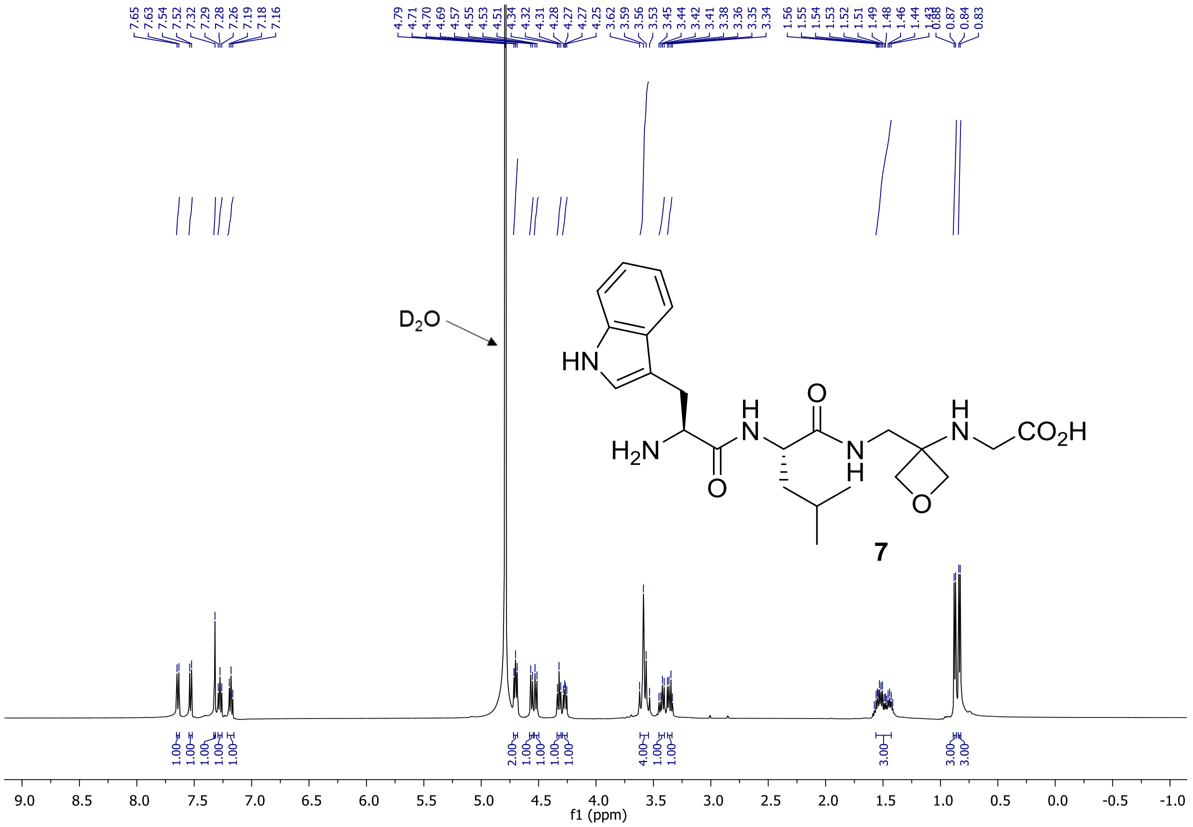

^{13}C NMR (126 MHz, D_2O)

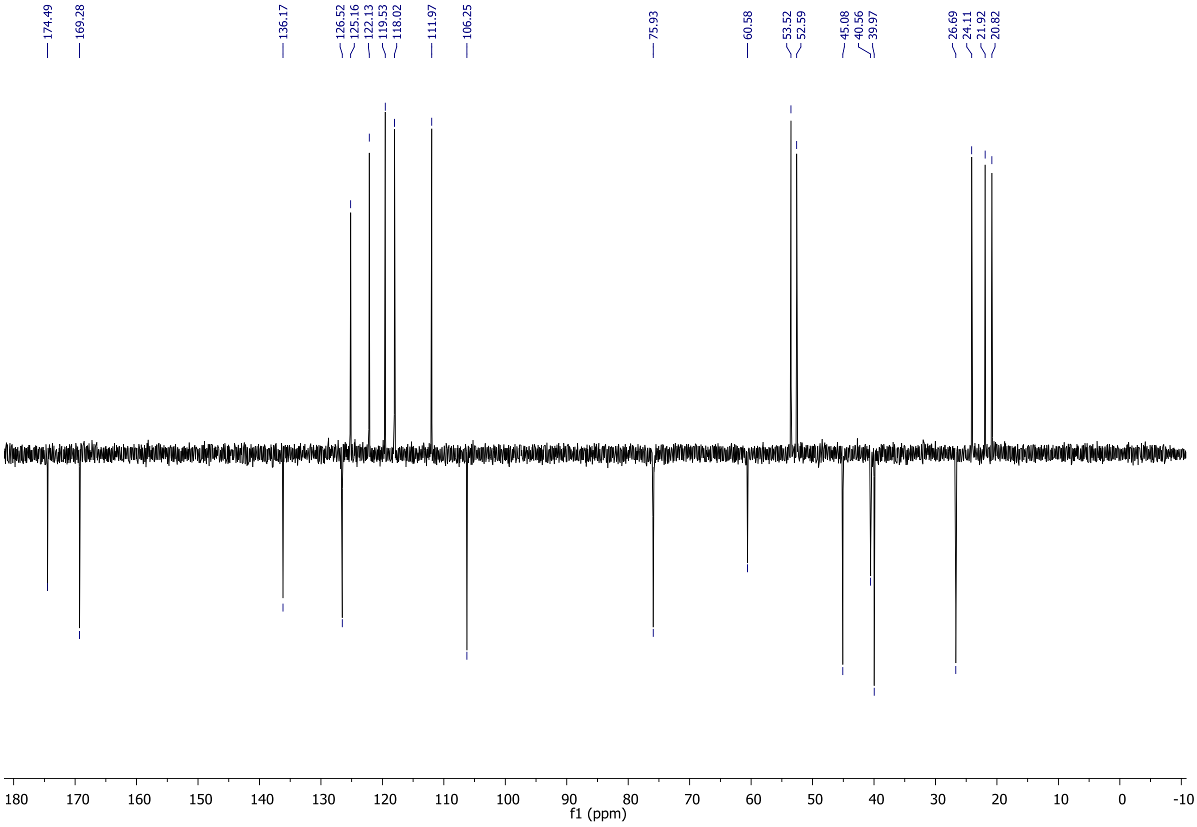

Cyclo(Trp-Leu-GOx-Gly) (9)

^{1}H NMR (500 MHz, DMSO-*d6*)

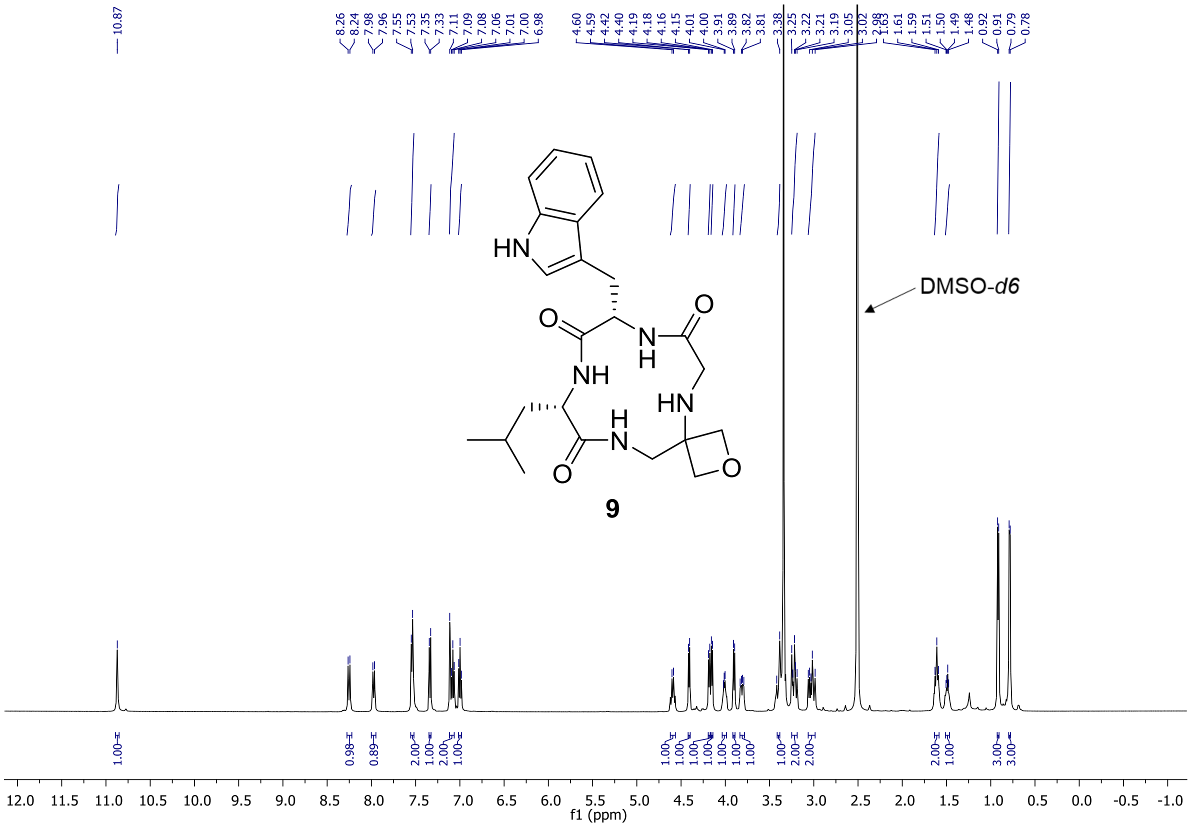

^{13}C NMR (126 MHz, DMSO-*d6*)

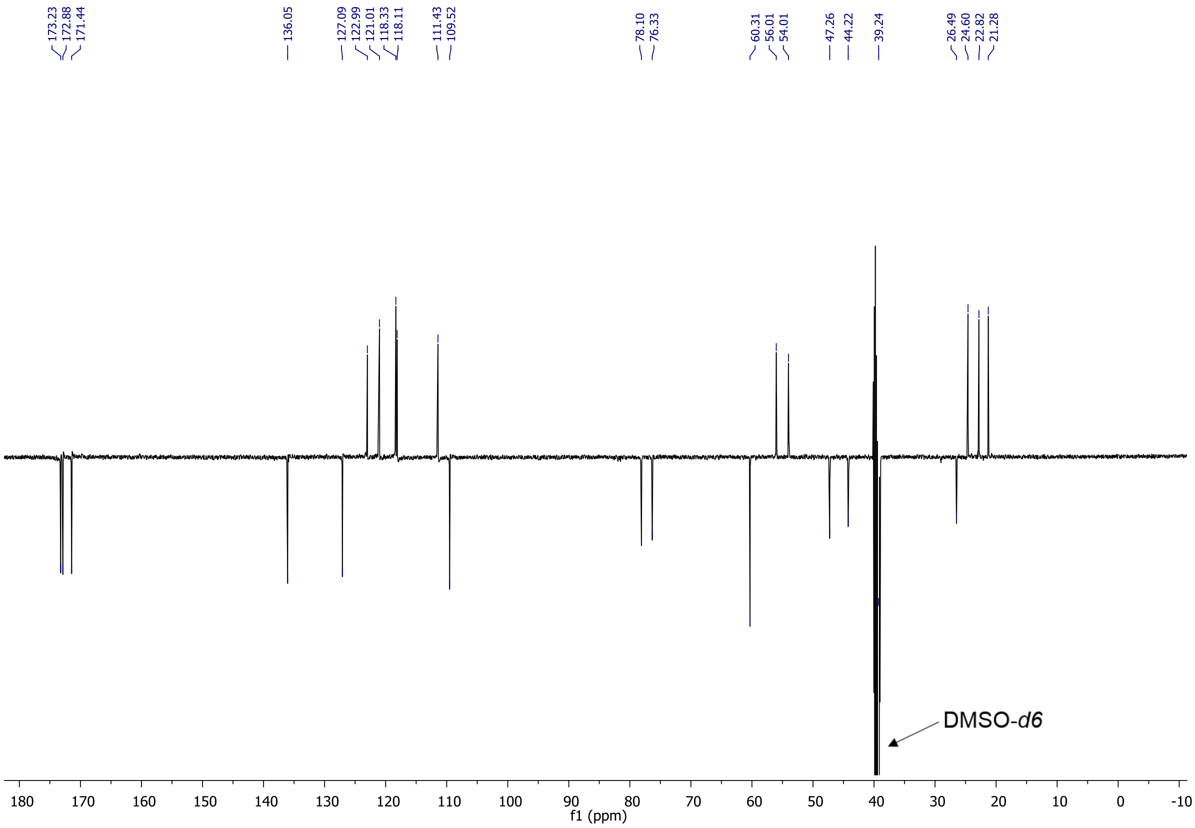

Cbz-D-Pro-Leu-GOx-Gly-OBn (36)

^{1}H NMR (600 MHz, DMSO-*d6* @ 373 K)

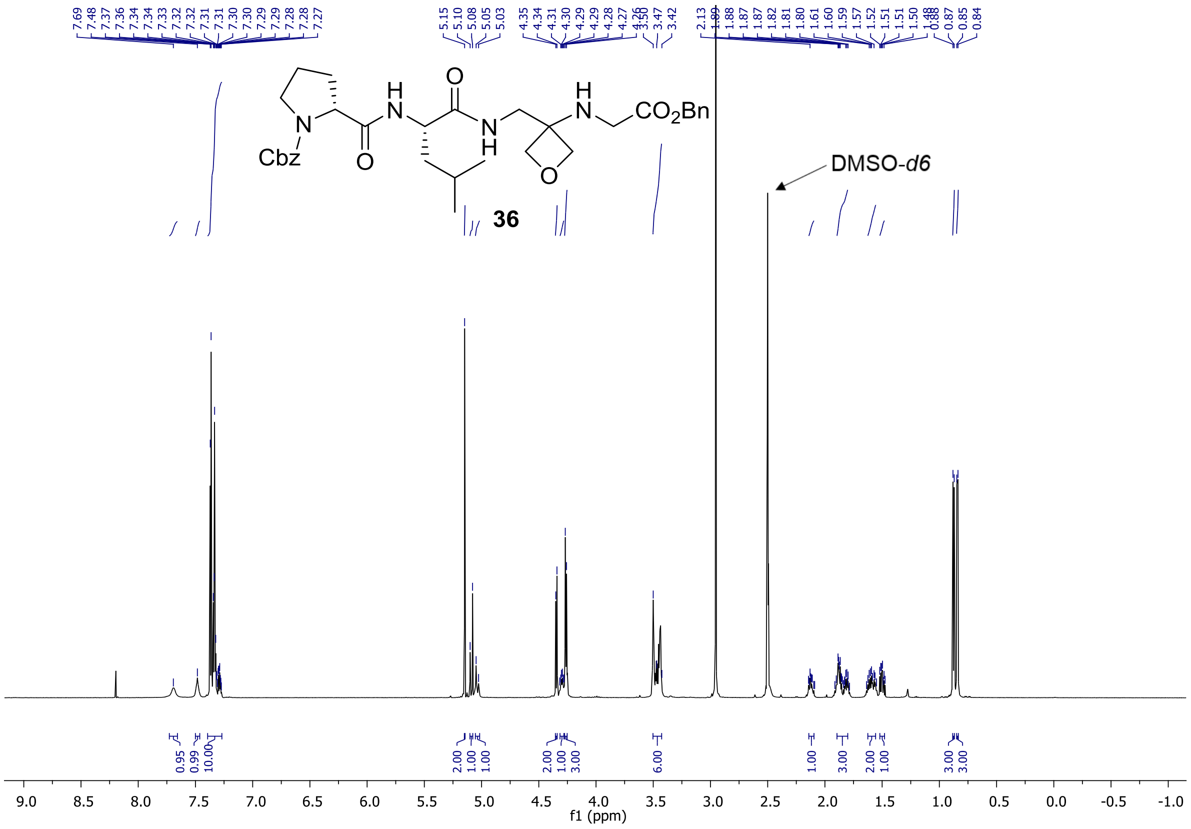

^{13}C NMR (151 MHz, DMSO-*d6* @ 373 K)

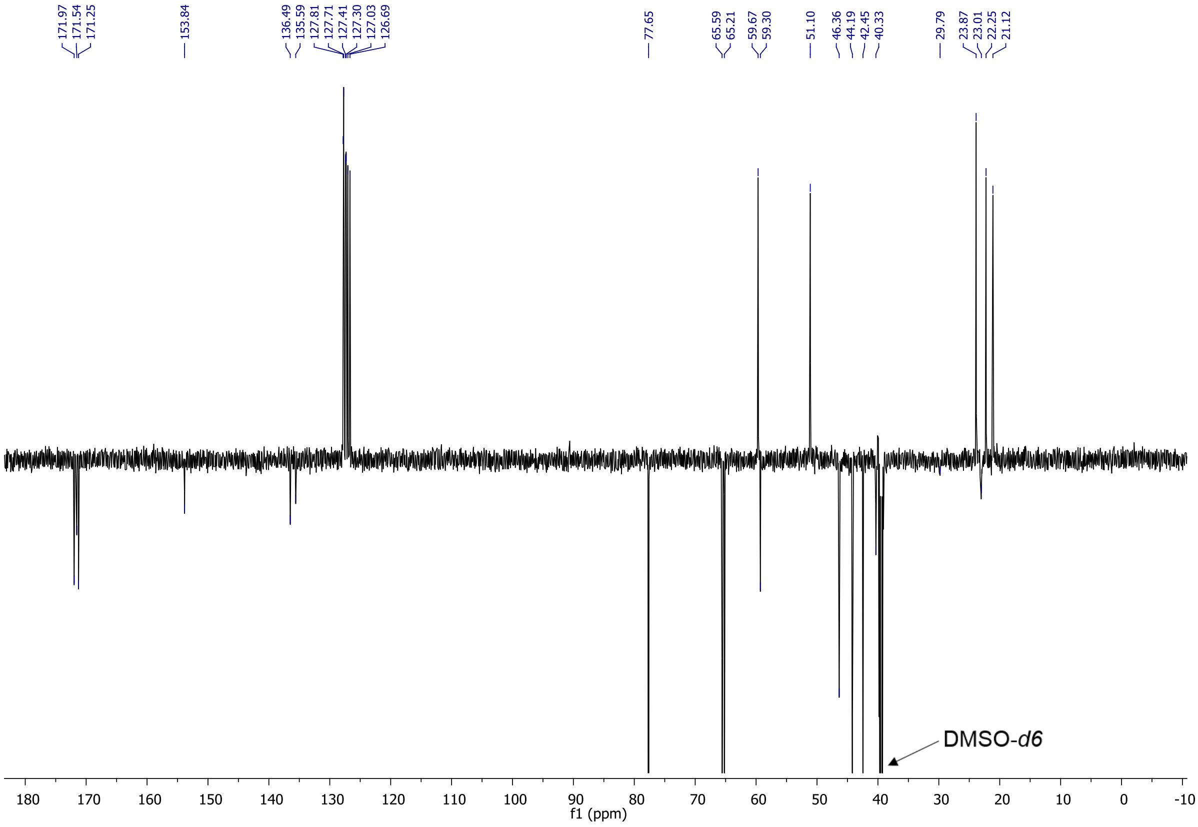

H-D-Pro-Leu-GOx-Gly-OH (37)

^{1}H NMR (500 MHz, D_2O)

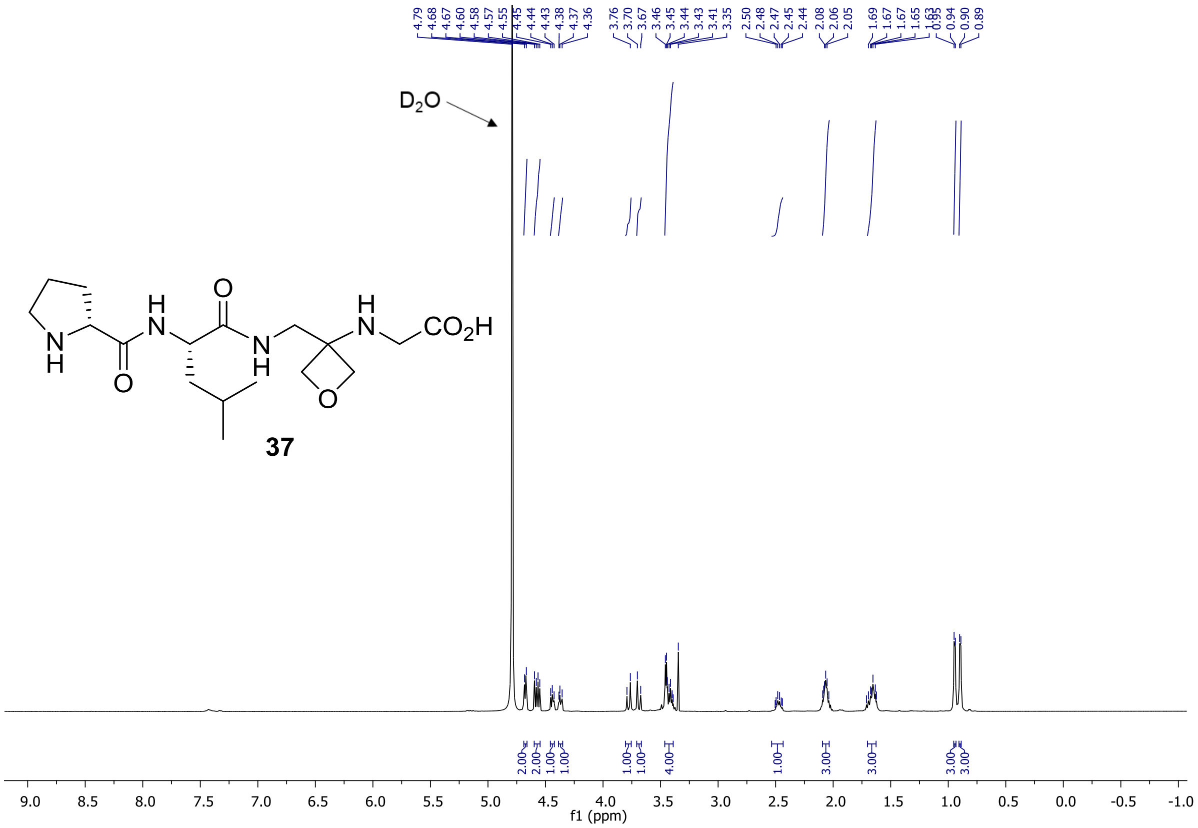

^{13}C NMR (126 MHz, D_2O)

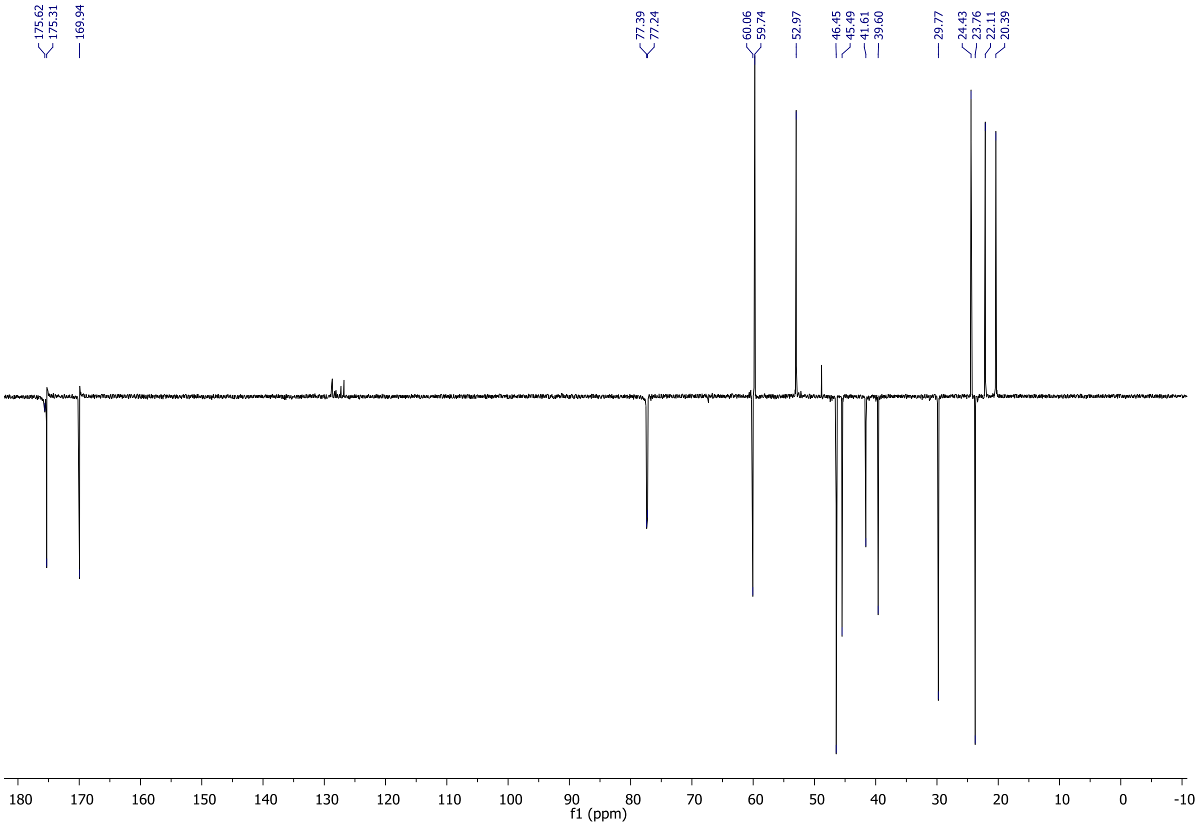

Cyclo(D-Pro-Leu-GOx-Gly) (10)

^{1}H NMR (500 MHz, CD_3OD)

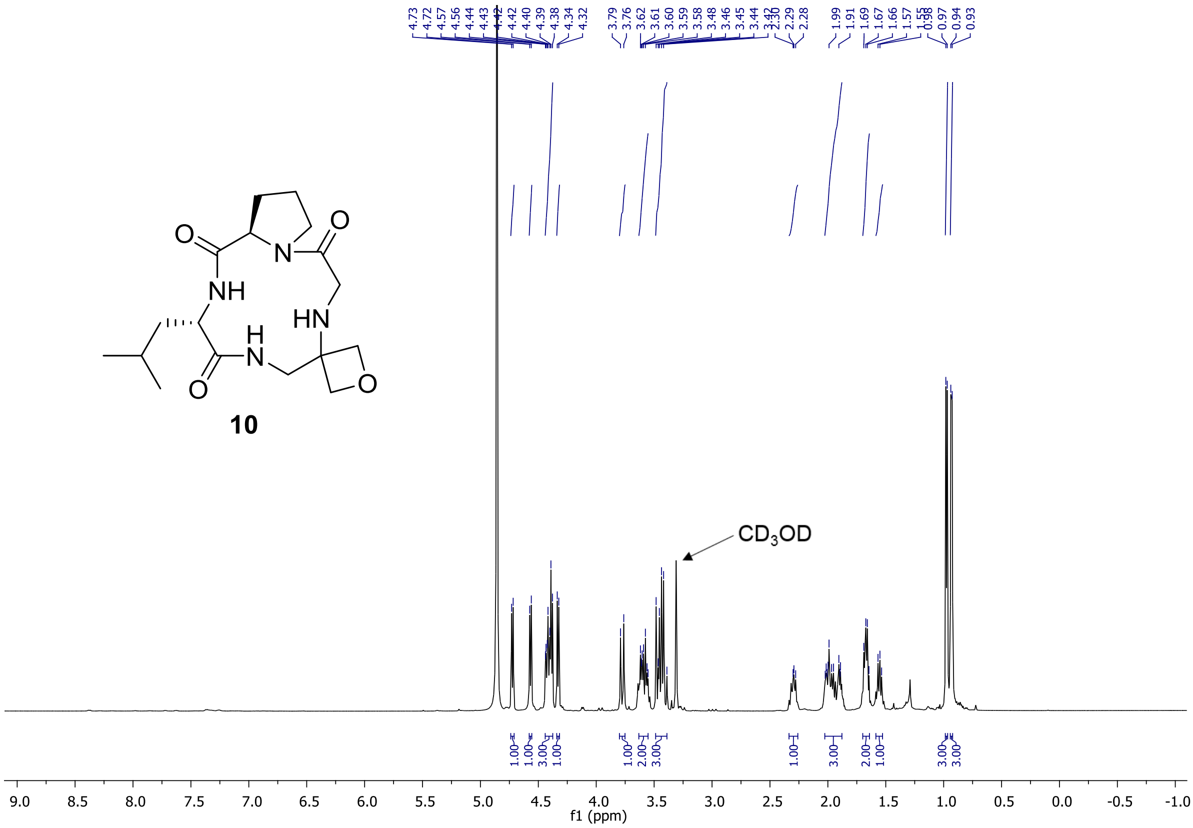

^{13}C NMR (126 MHz, CD_3OD)

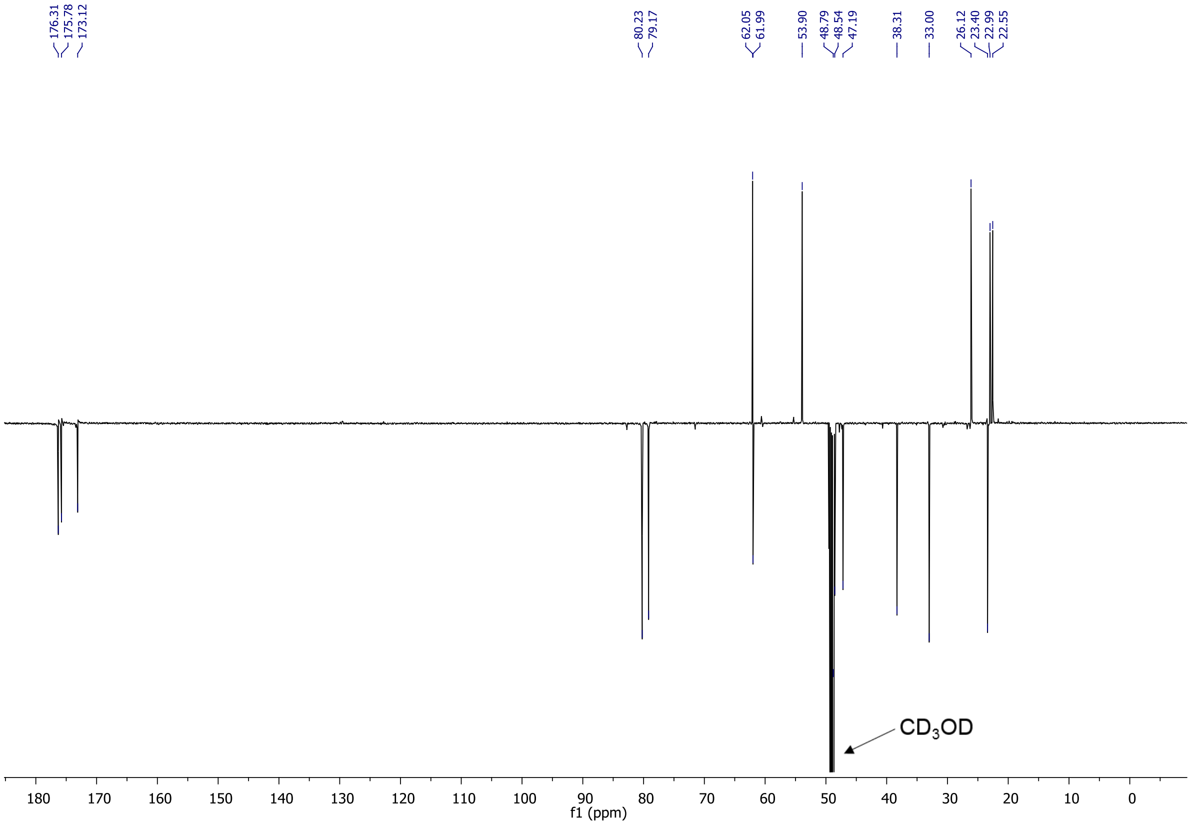

Cbz-Asp(*t*Bu)-Leu-GOx-Gly-OBn (38)

^{1}H NMR (500 MHz, $CDCl_3$)

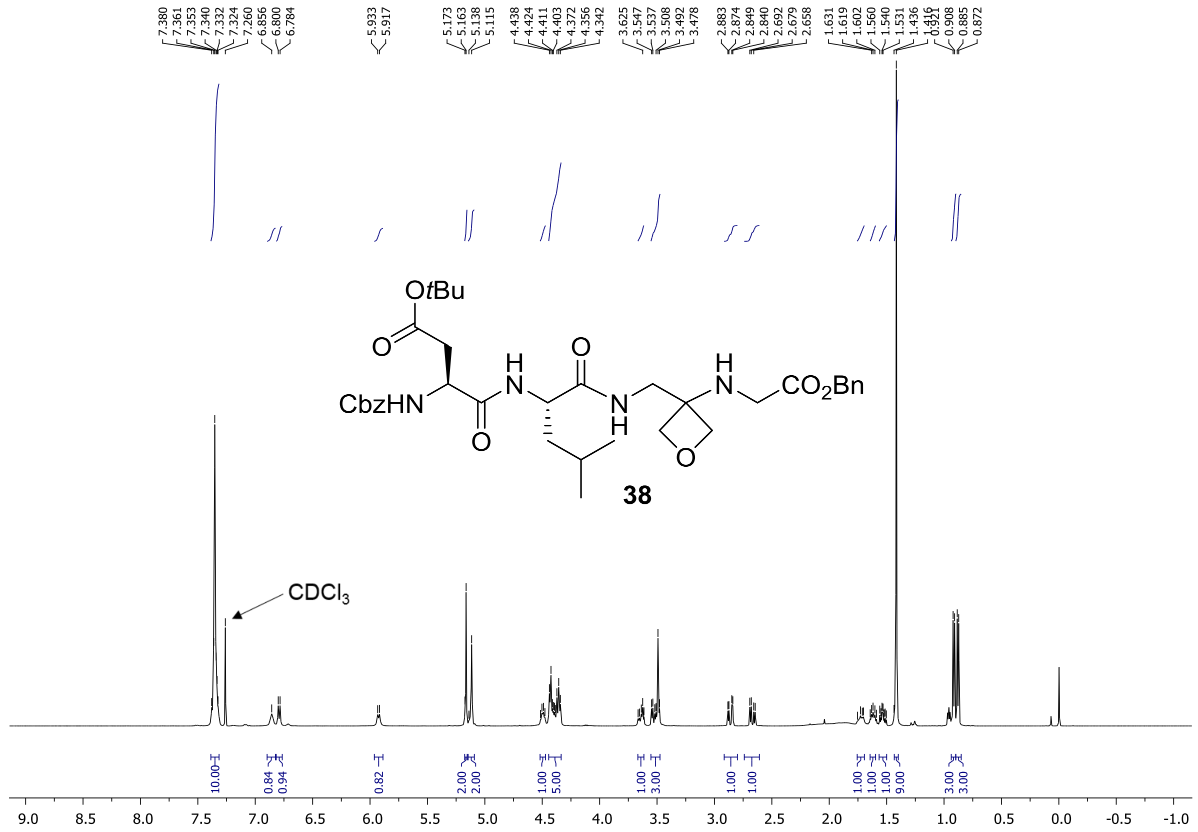

^{13}C NMR (126 MHz, $CDCl_3$)

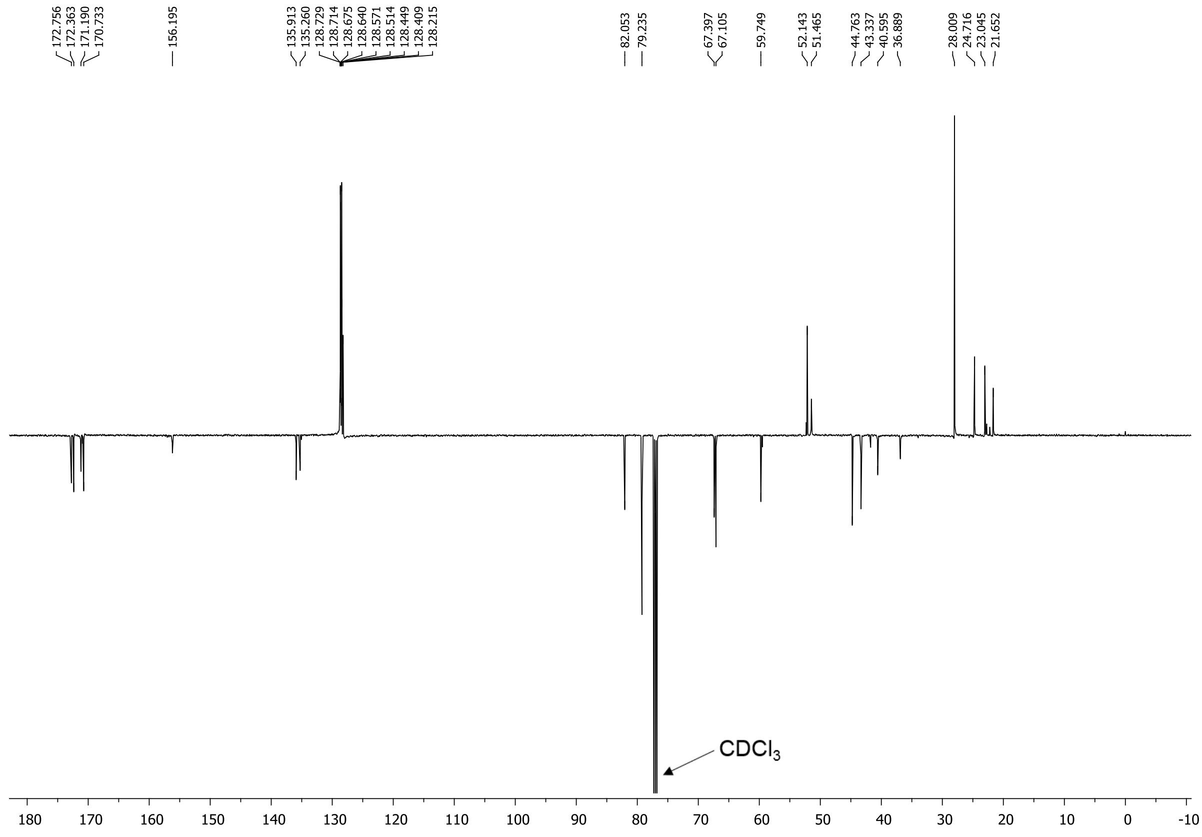

H-Asp(*t*Bu)-Leu-GOx-Gly-OBn (39)

^{1}H NMR (500 MHz, CD_3OD)

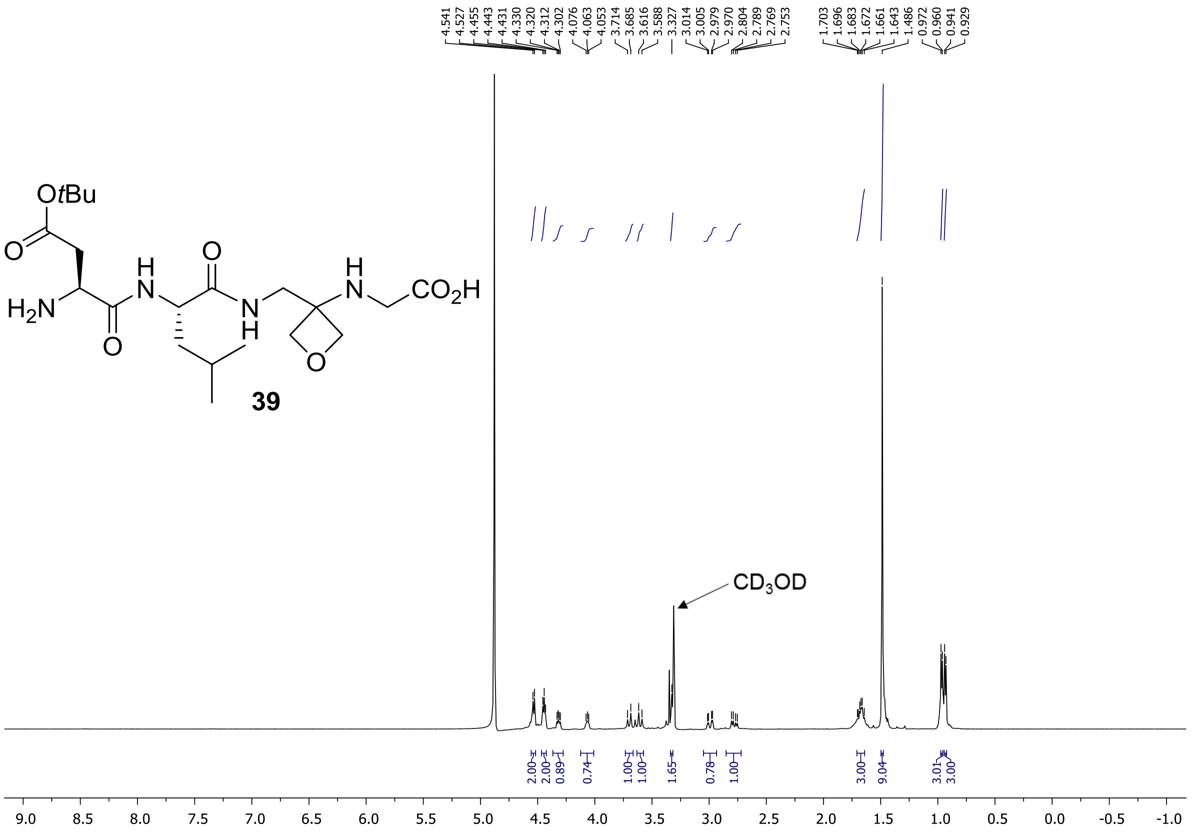

^{13}C NMR (126 MHz, CD_3OD)

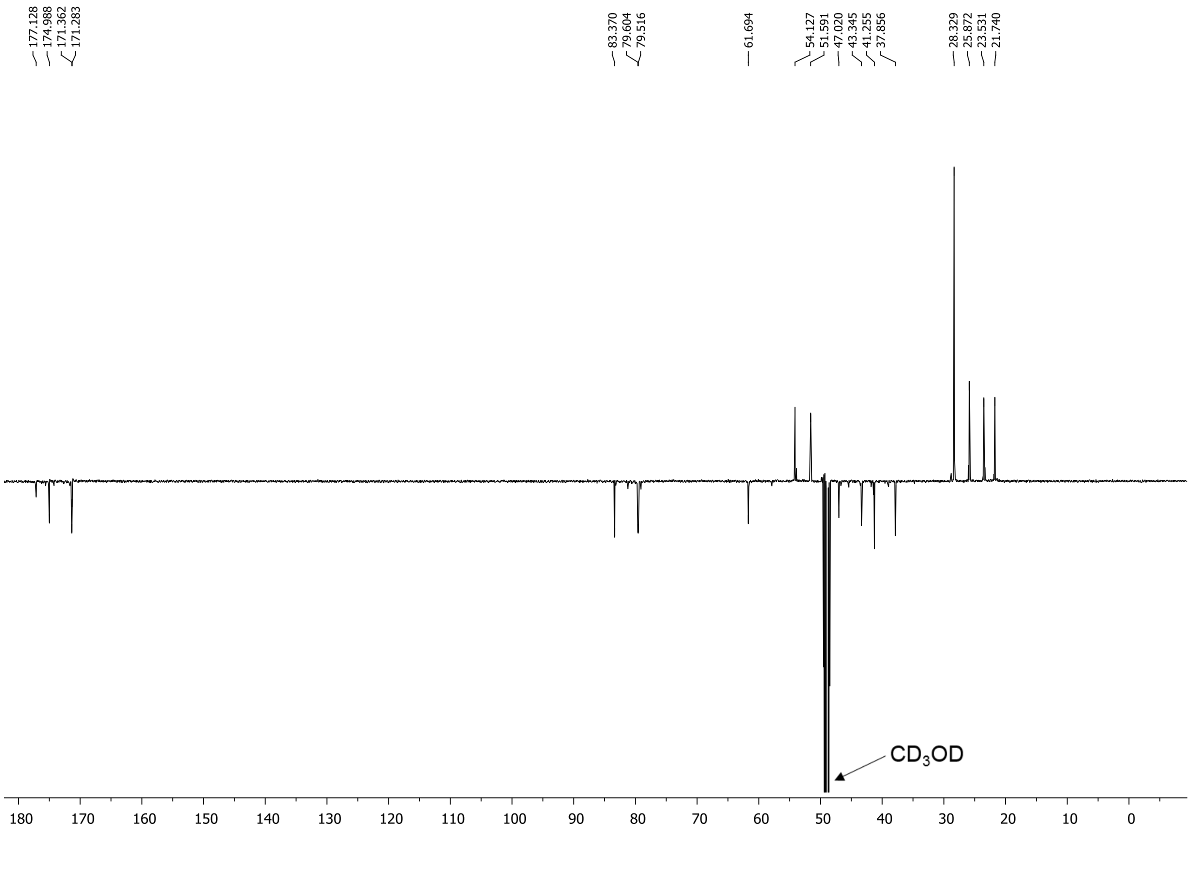

Cyclo(Asp(*t*Bu)-Leu-GOx-Gly) (11)

^{1}H NMR (500 MHz, DMSO-*d6*)

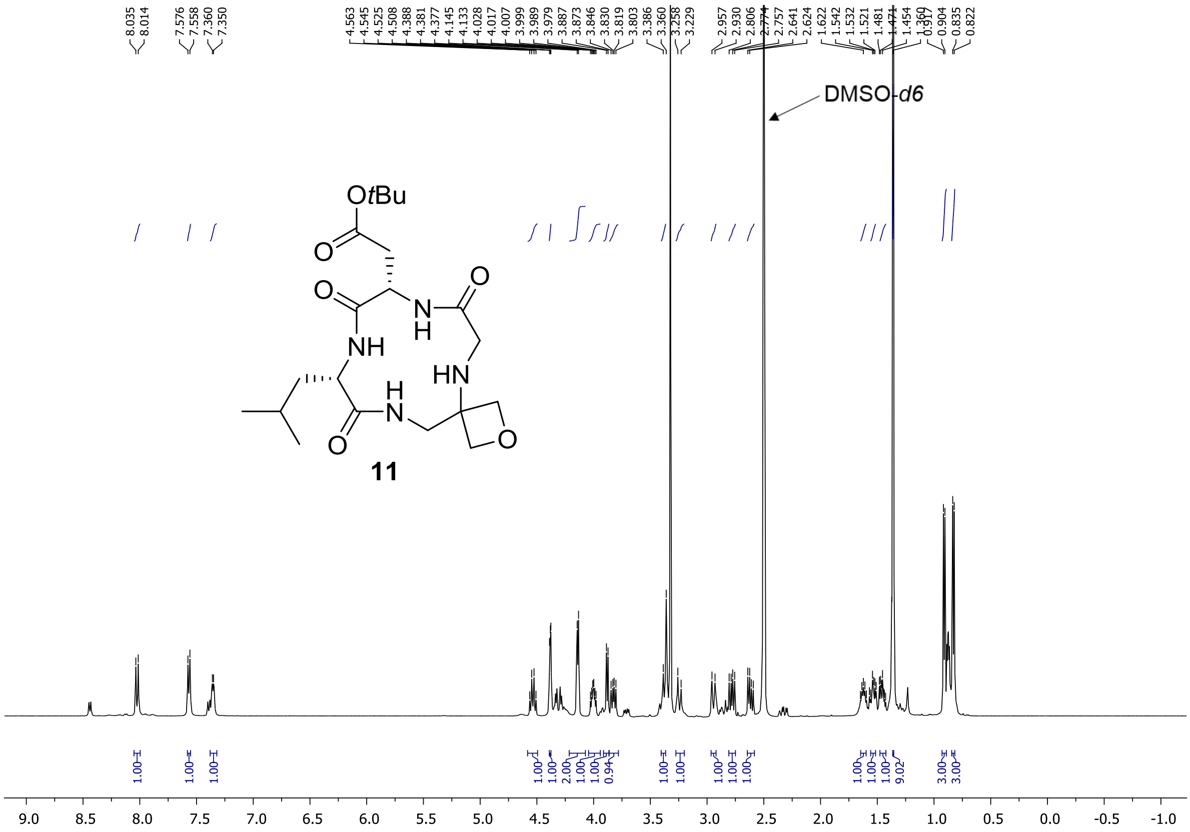

^{13}C NMR (126 MHz, DMSO-*d6*)

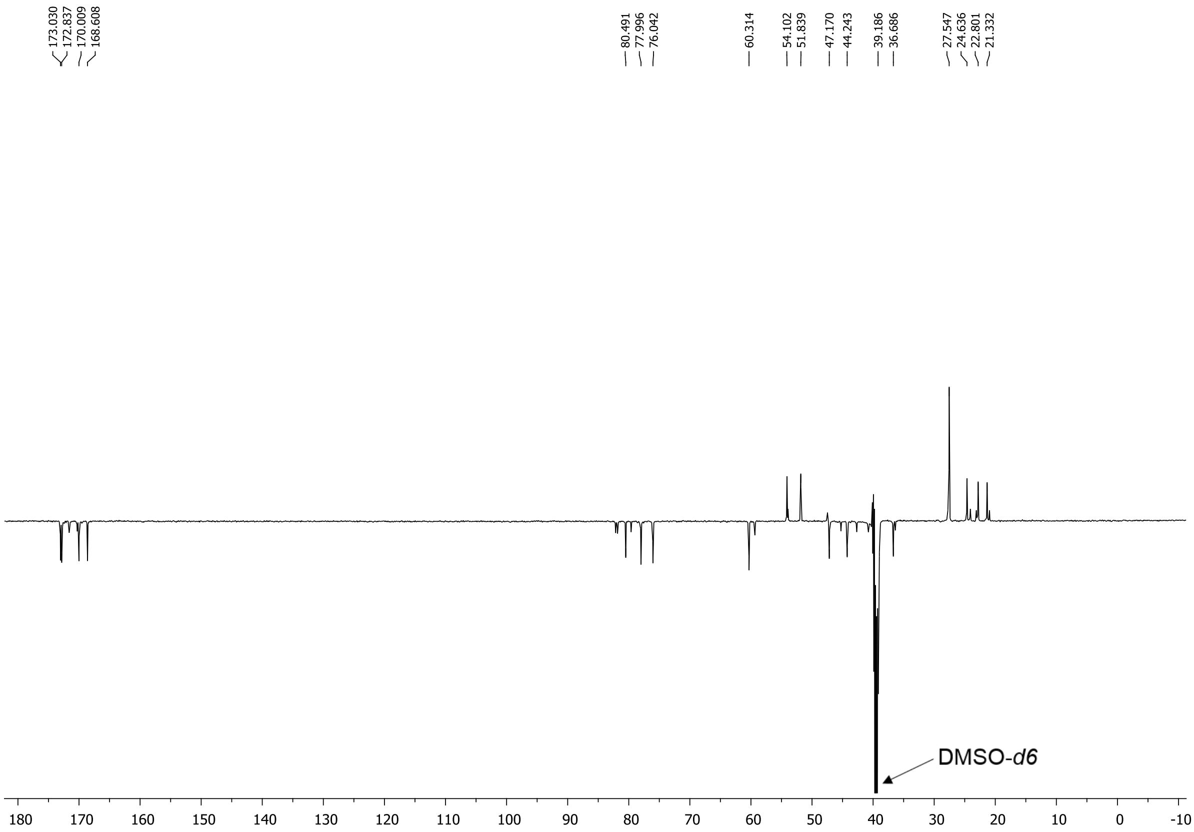

Boc-Leu-Gly-Gly-OBn (40)

^{1}H NMR (500 MHz, $CDCl_3$)

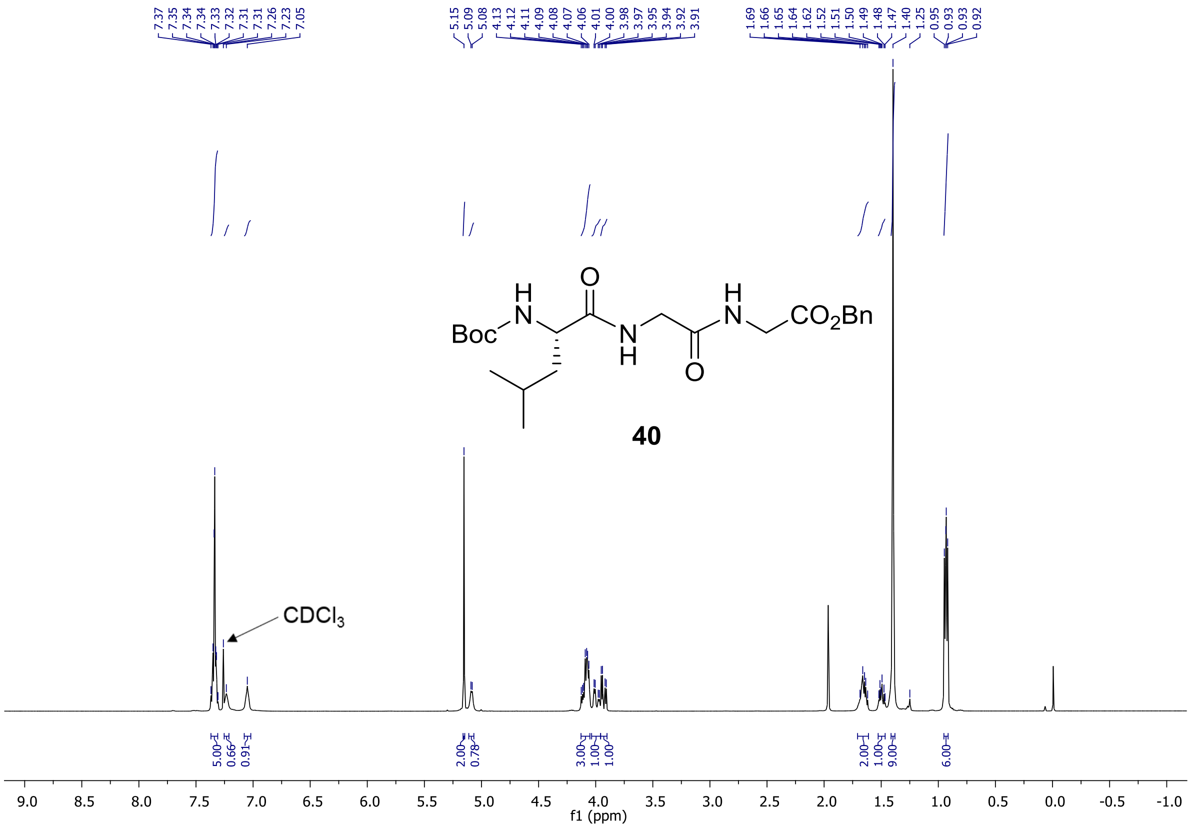

^{13}C NMR (126 MHz, $CDCl_3$)

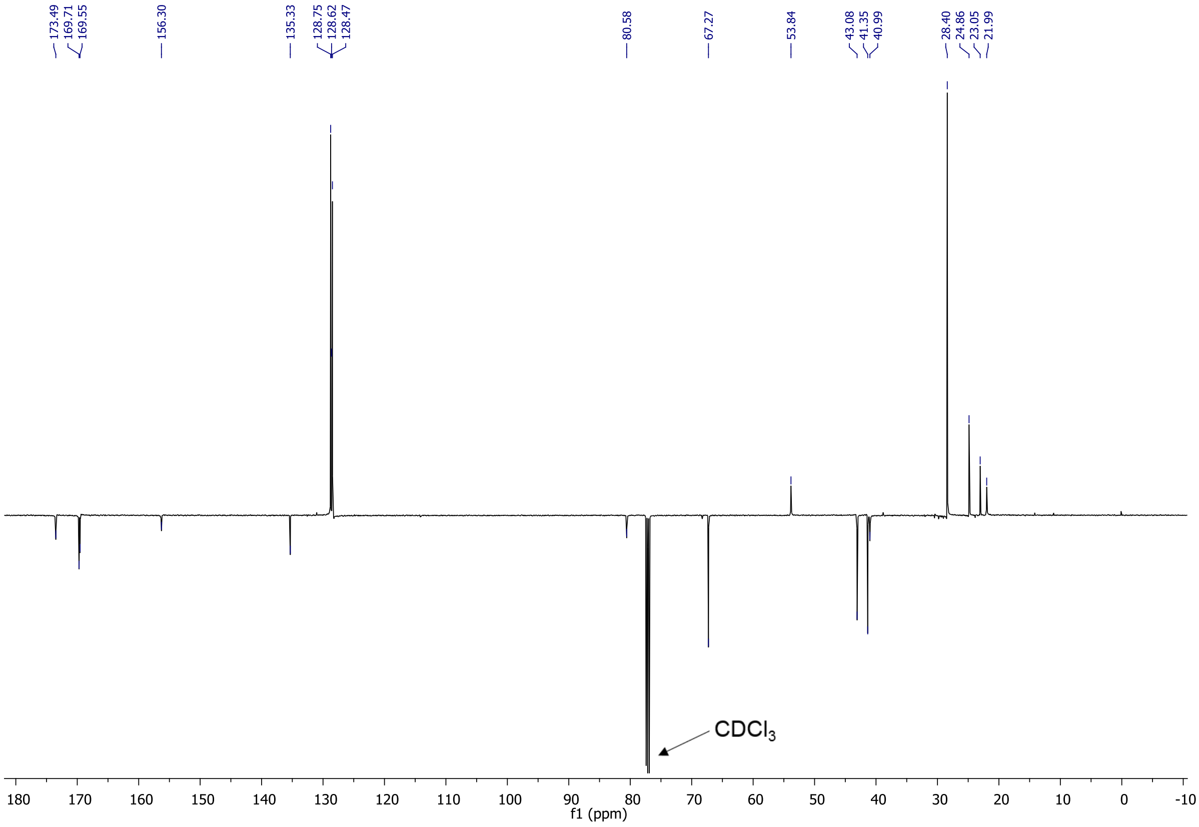

Cbz-Trp-Leu-Gly-Gly-OBn (41)

^1H NMR (500 MHz, CD_3CN)

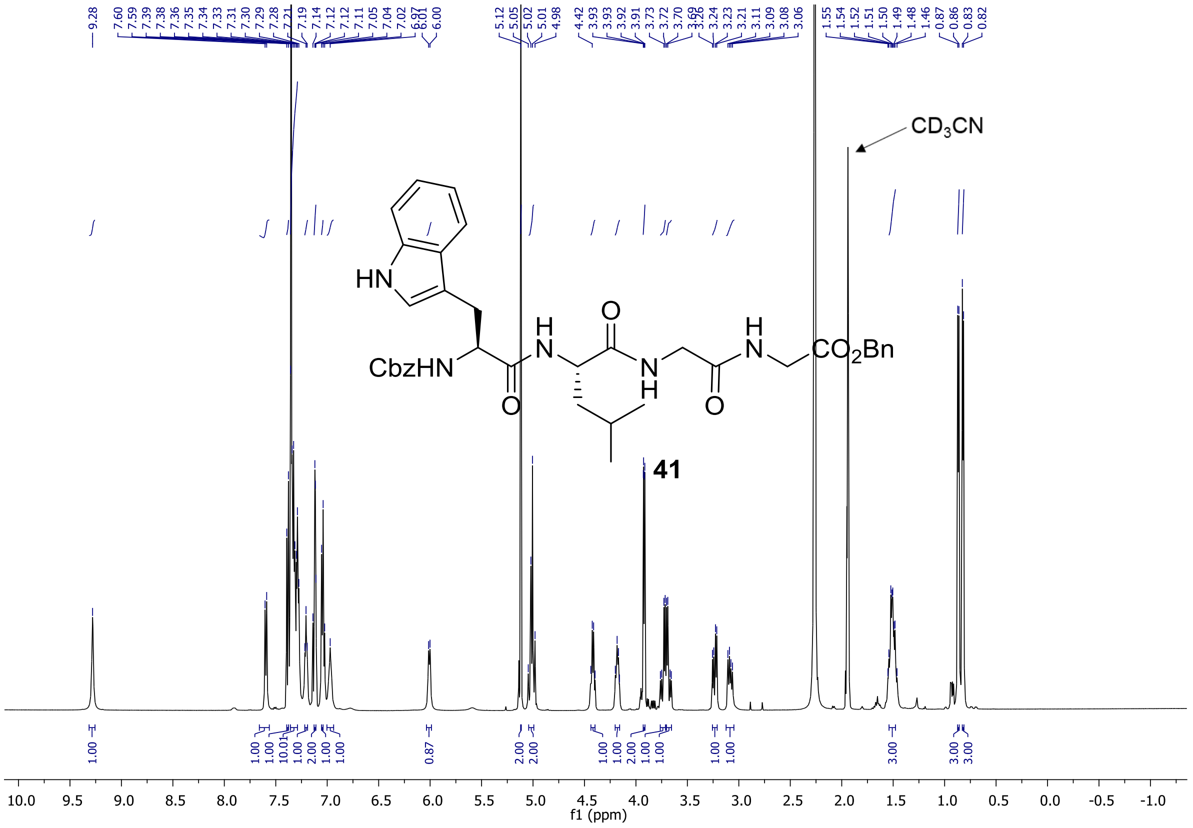

^{13}C NMR (126 MHz, CD_3CN)

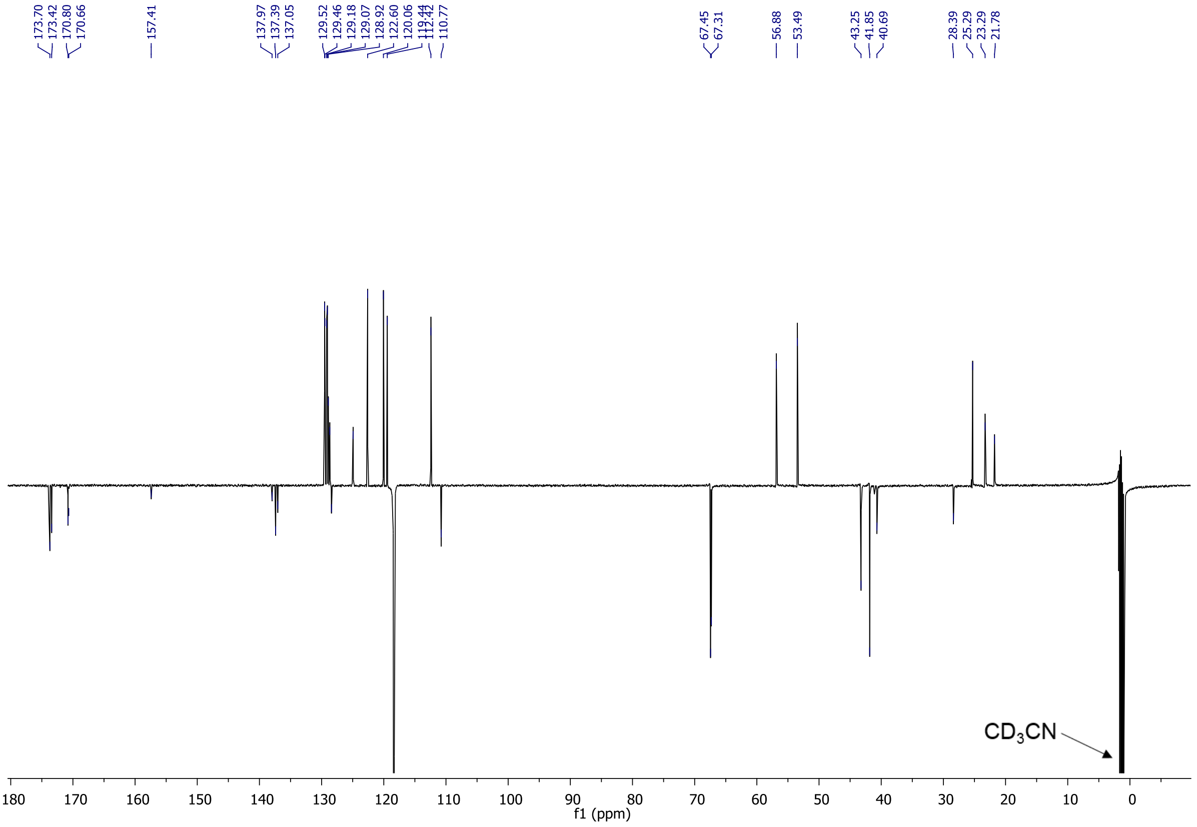

H-Trp-Leu-Gly-Gly-OH (6)

^{1}H NMR (500 MHz, D_2O)

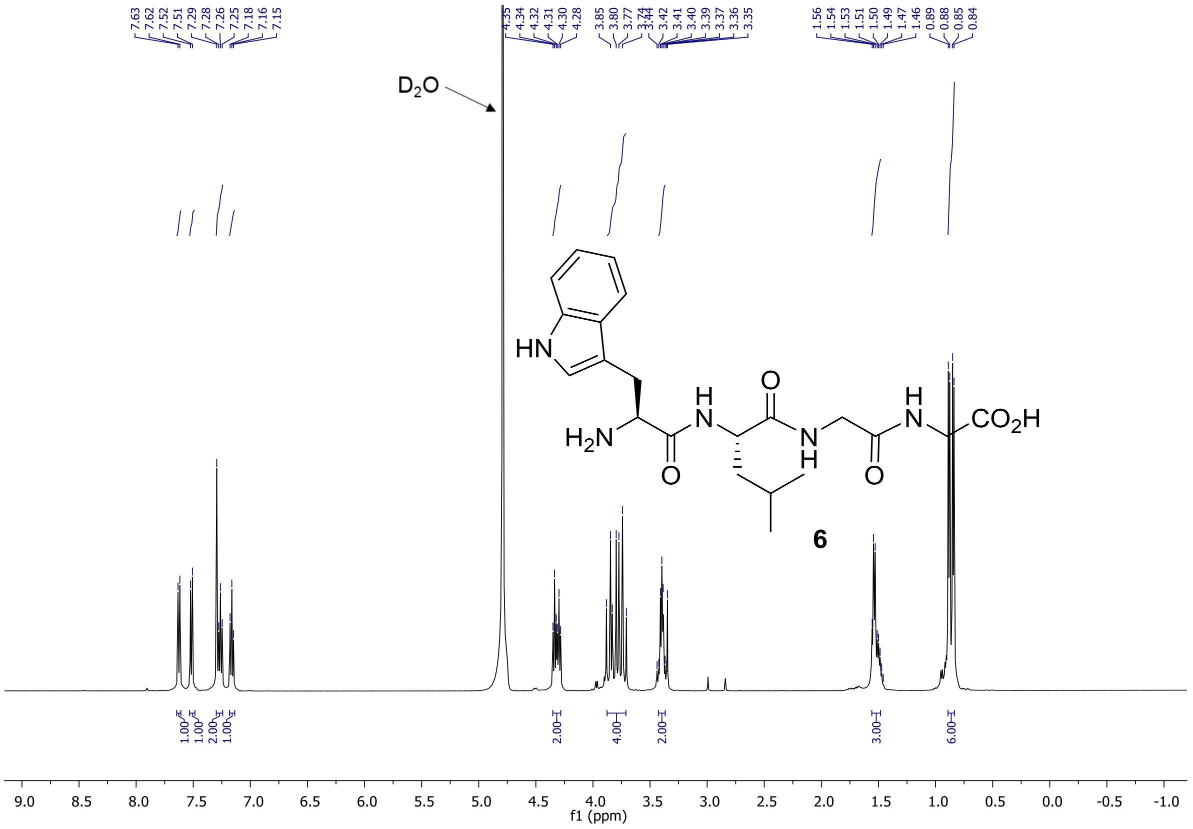

^{13}C NMR (126 MHz, D_2O)

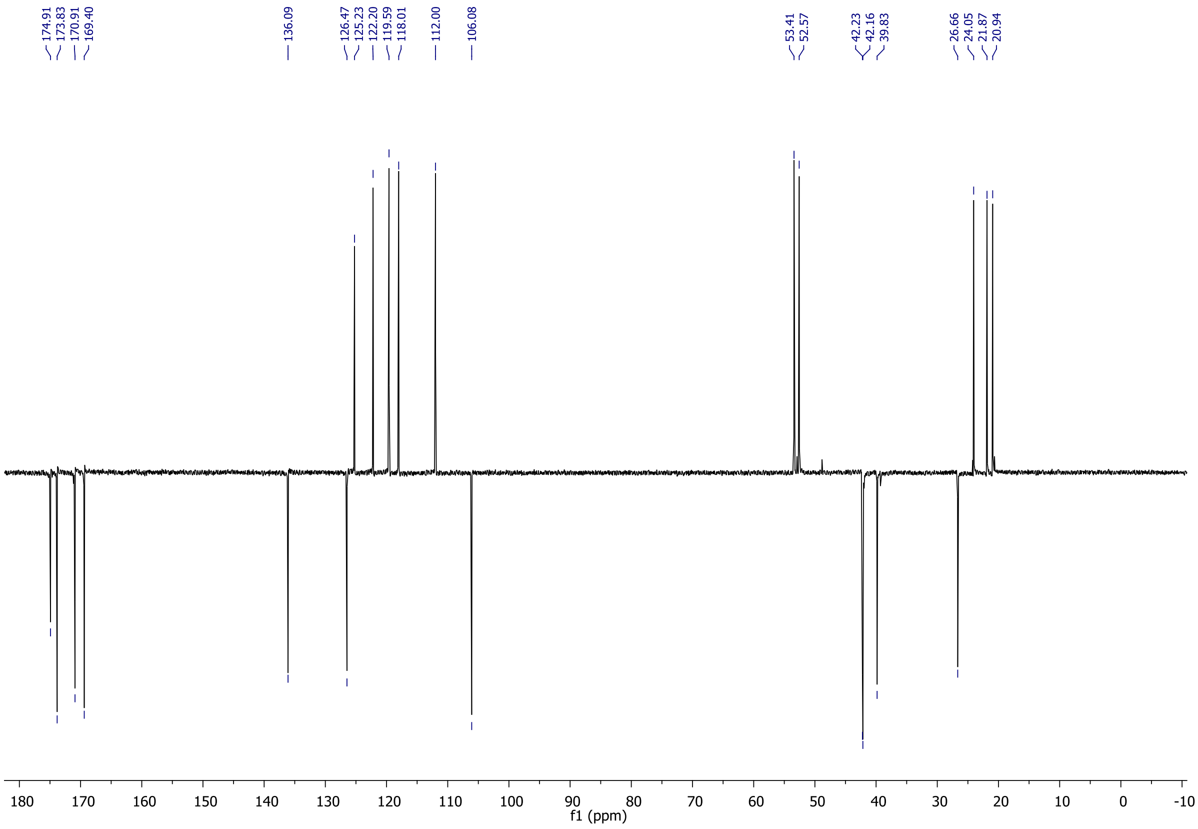

Cyclo(Trp-Leu-Gly-Gly) (8)

^{1}H NMR (500 MHz, DMSO-*d6*)

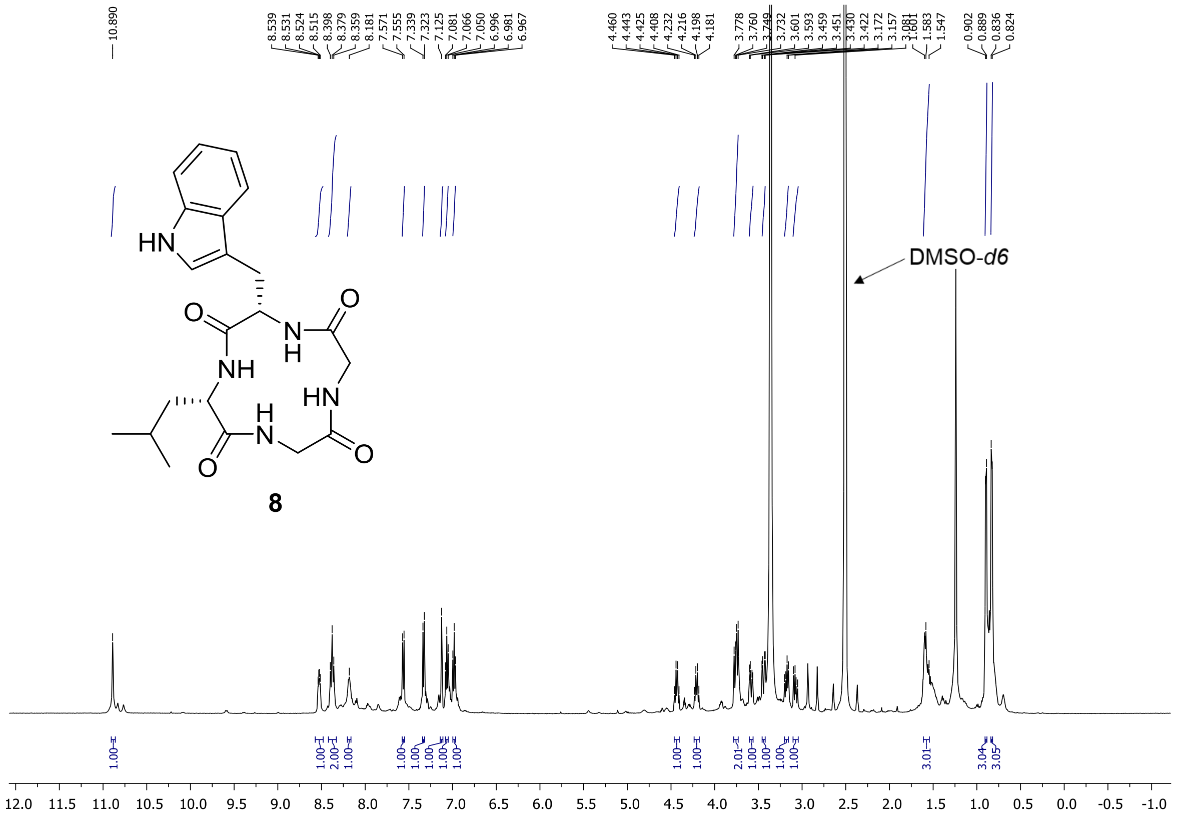

^{13}C NMR (126 MHz, DMSO-*d6*)

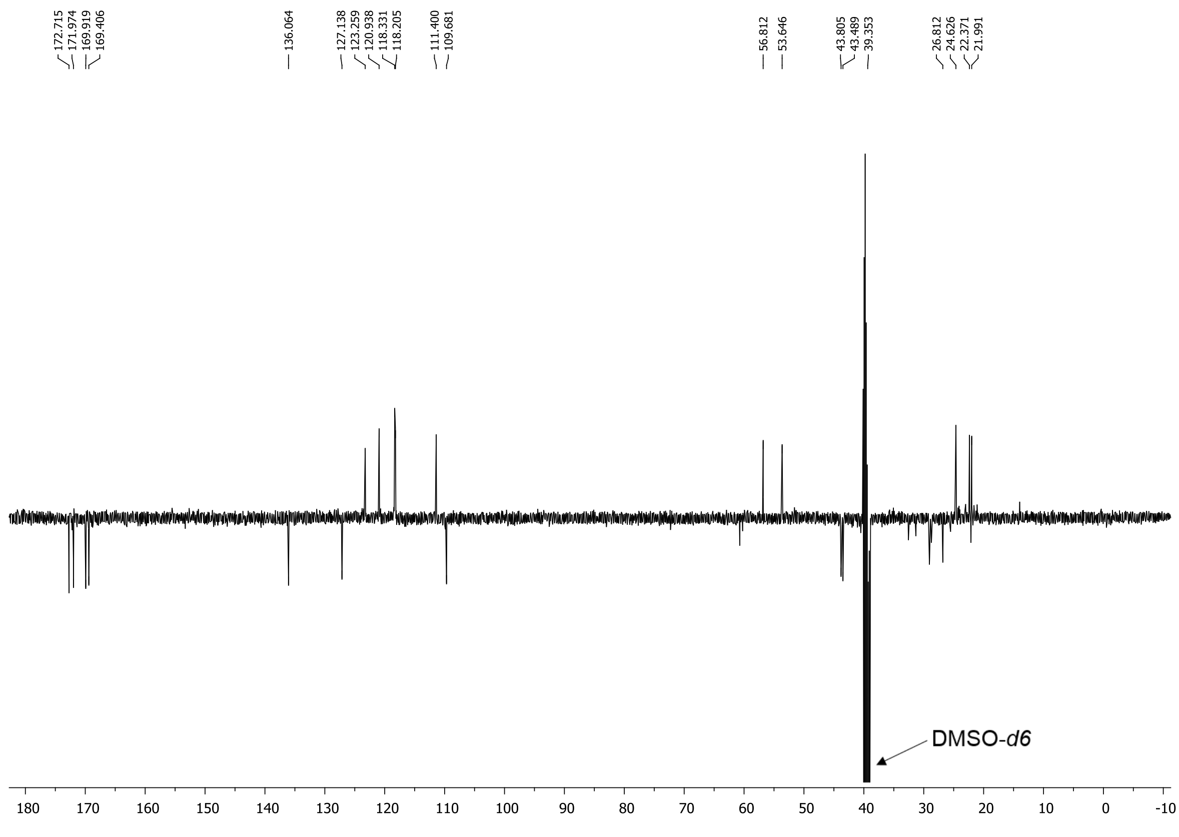

Cyclo(Trp-Leu-Gly-Gly-Trp-Leu-Gly-Gly) (42)

^{1}H NMR (500 MHz, CD_3OD)

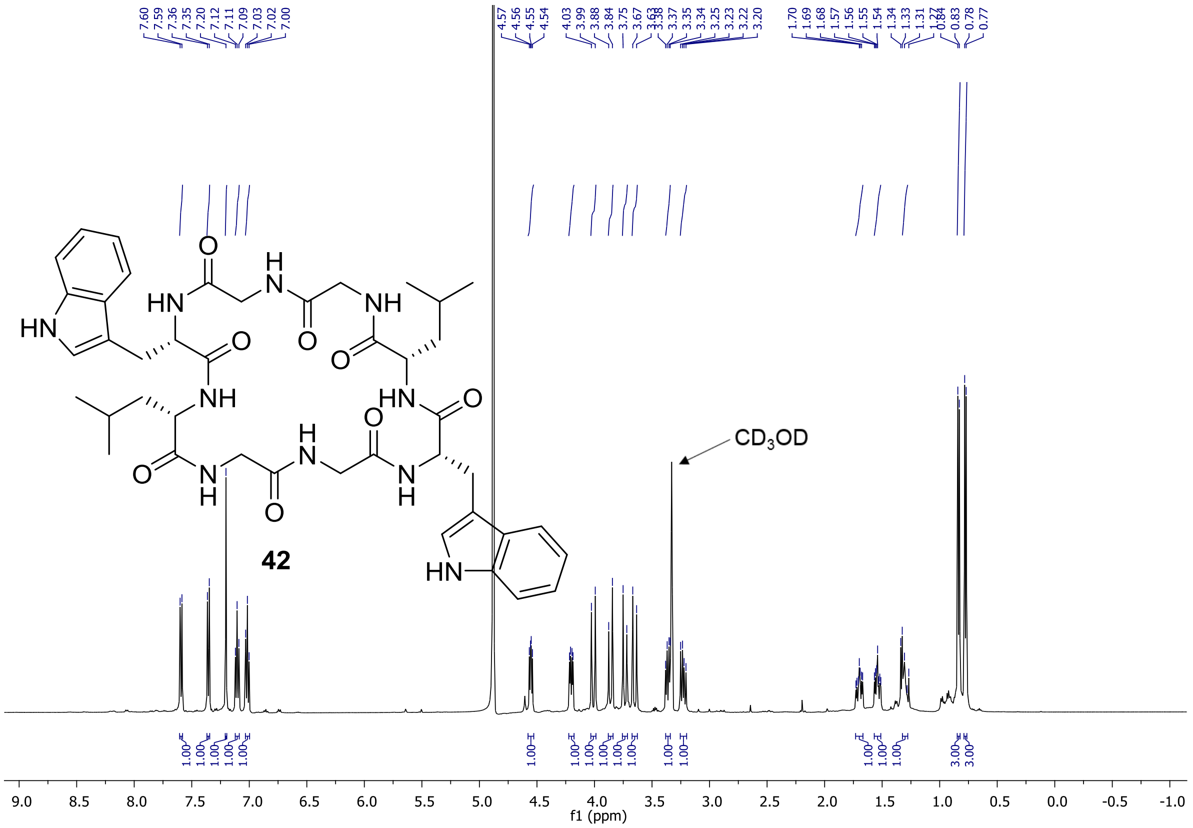

^{13}C NMR (126 MHz, CD_3OD)

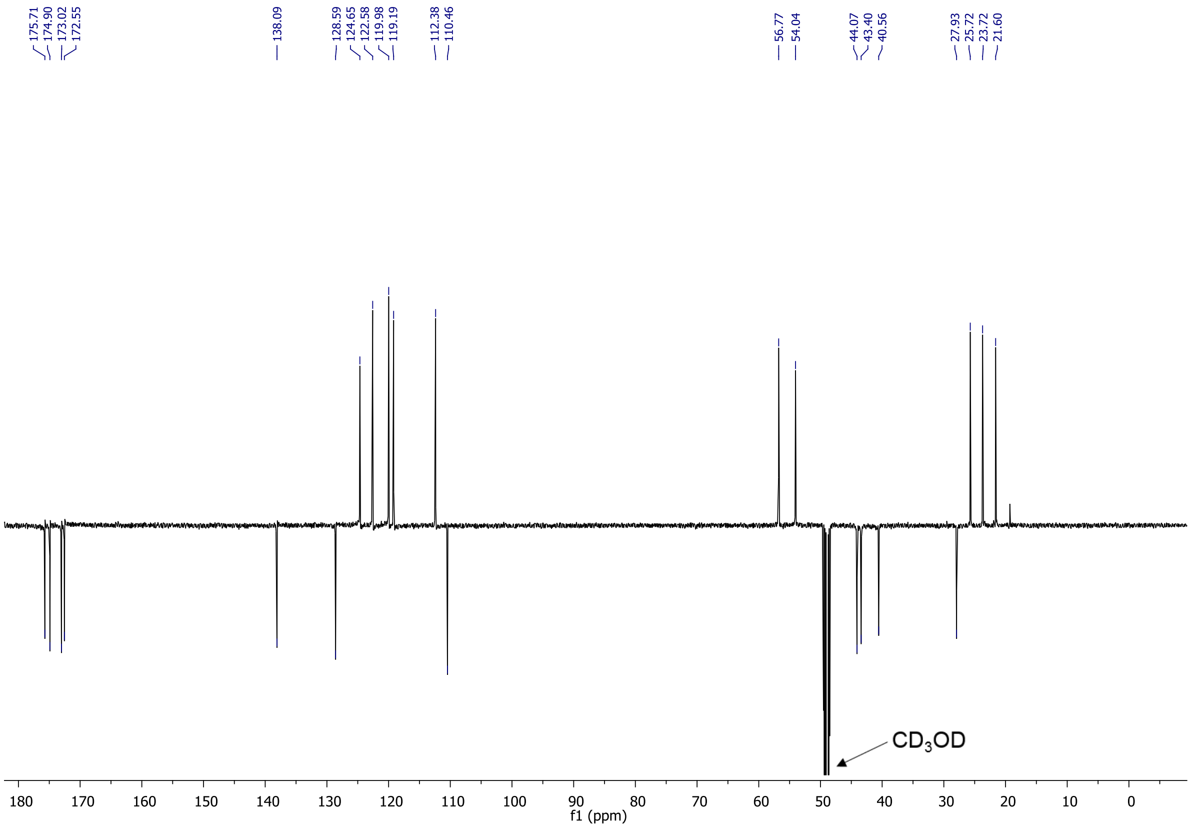

Cbz-D-Pro-Leu-Gly-Gly-OBn (43)

^{1}H NMR (600 MHz, DMSO-*d6* @ 373 K)

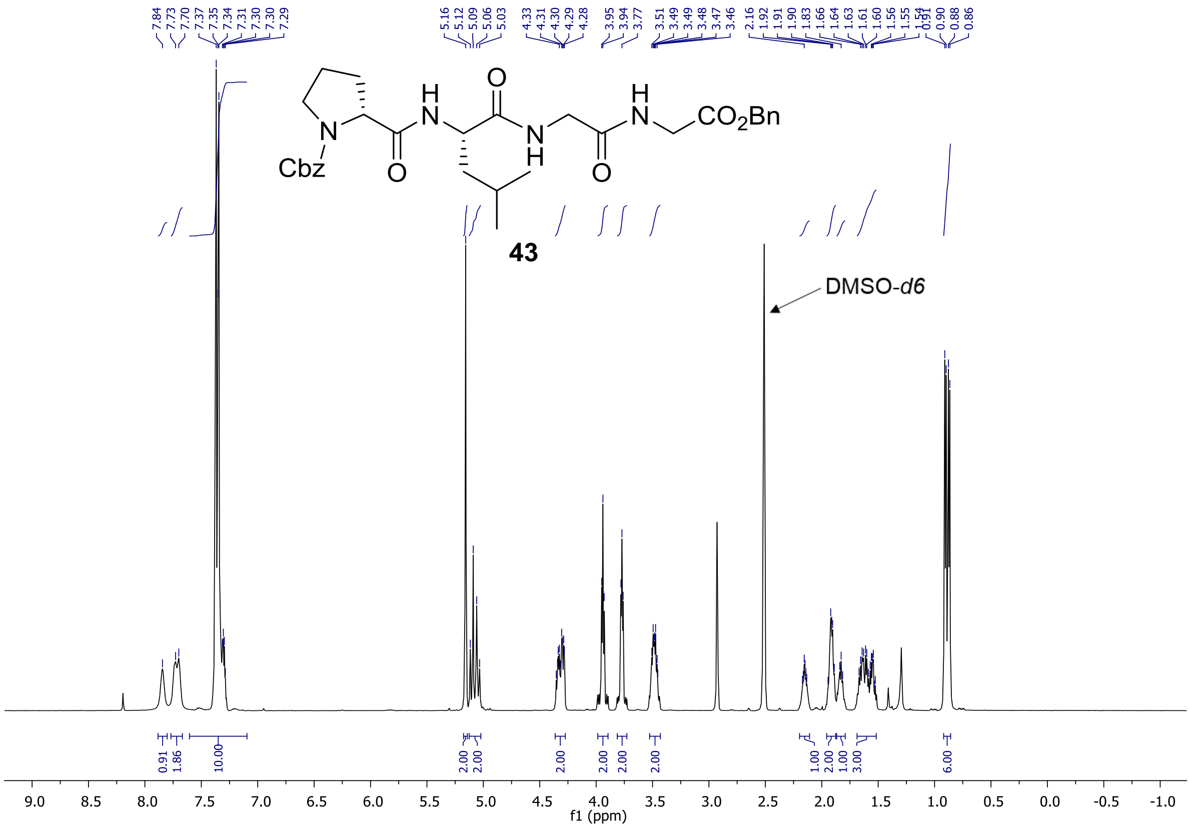

^{13}C NMR (151 MHz, DMSO-*d6* @ 373 K)

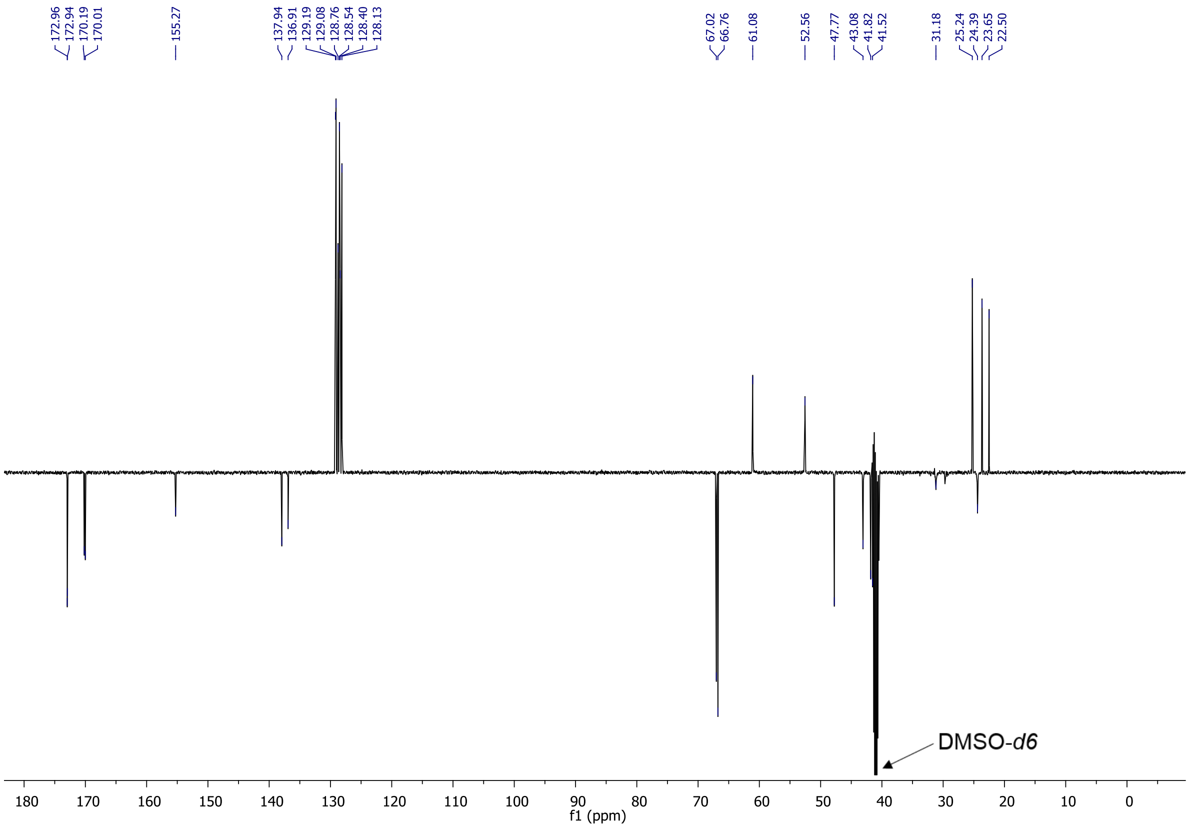

H-D-Pro-Leu-Gly-Gly-OH (44)

^{1}H NMR (500 MHz, D_2O)

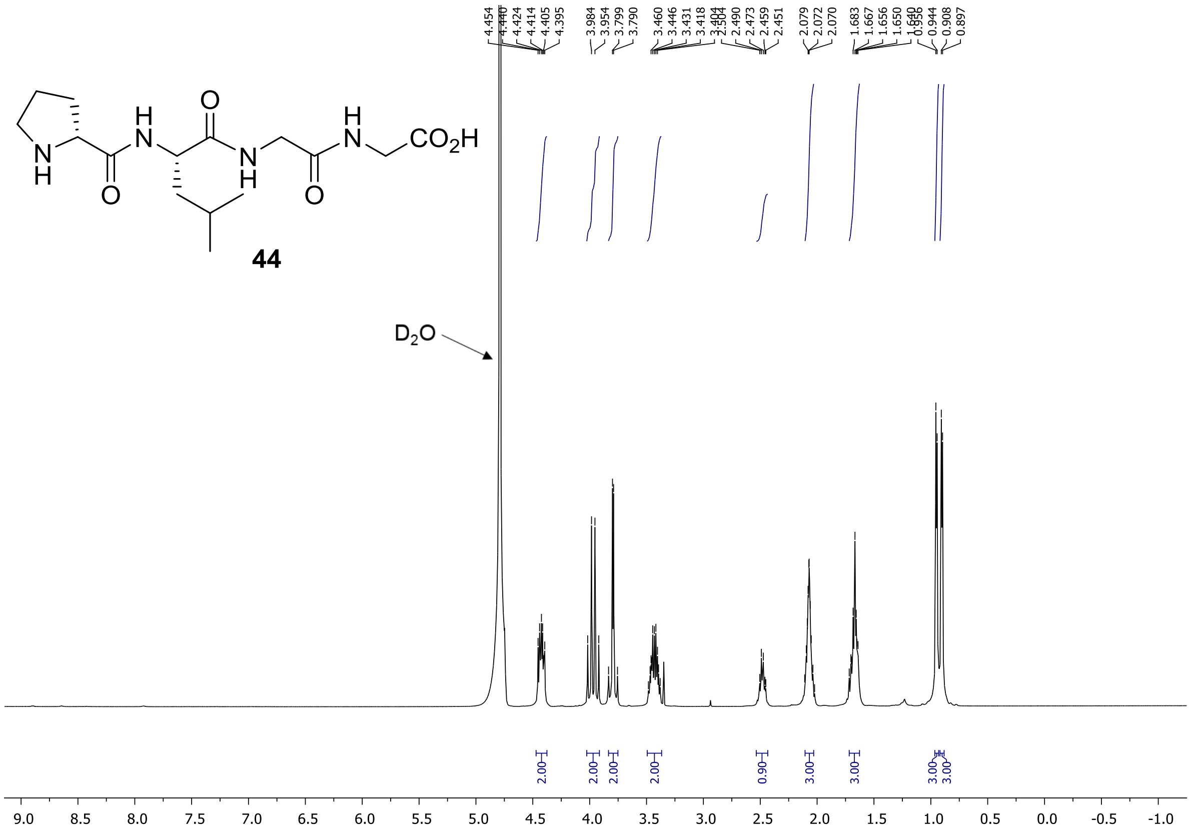

^{13}C NMR (126 MHz, D_2O)

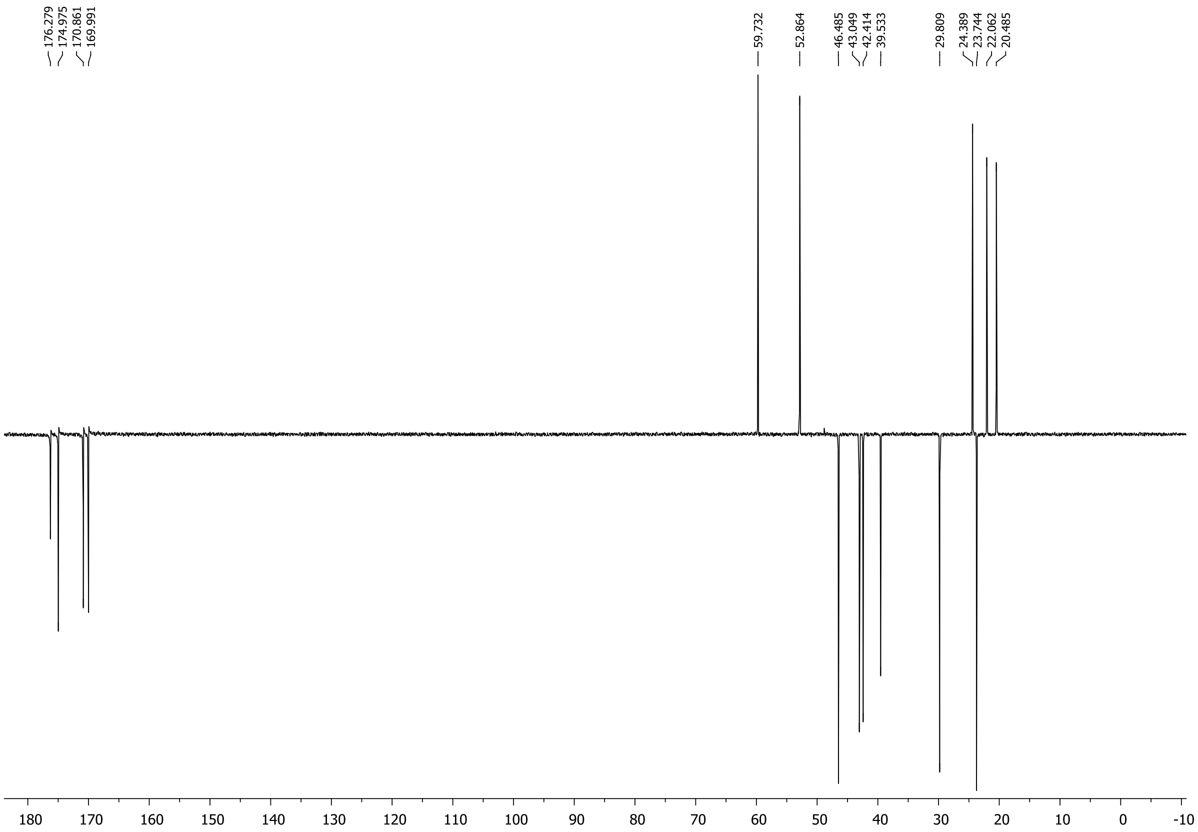

Cyclo(D-Pro-Leu-Gly-Gly-D-Pro-Leu-Gly-Gly) (45)

^{1}H NMR (500 MHz, DMSO-*d6*)

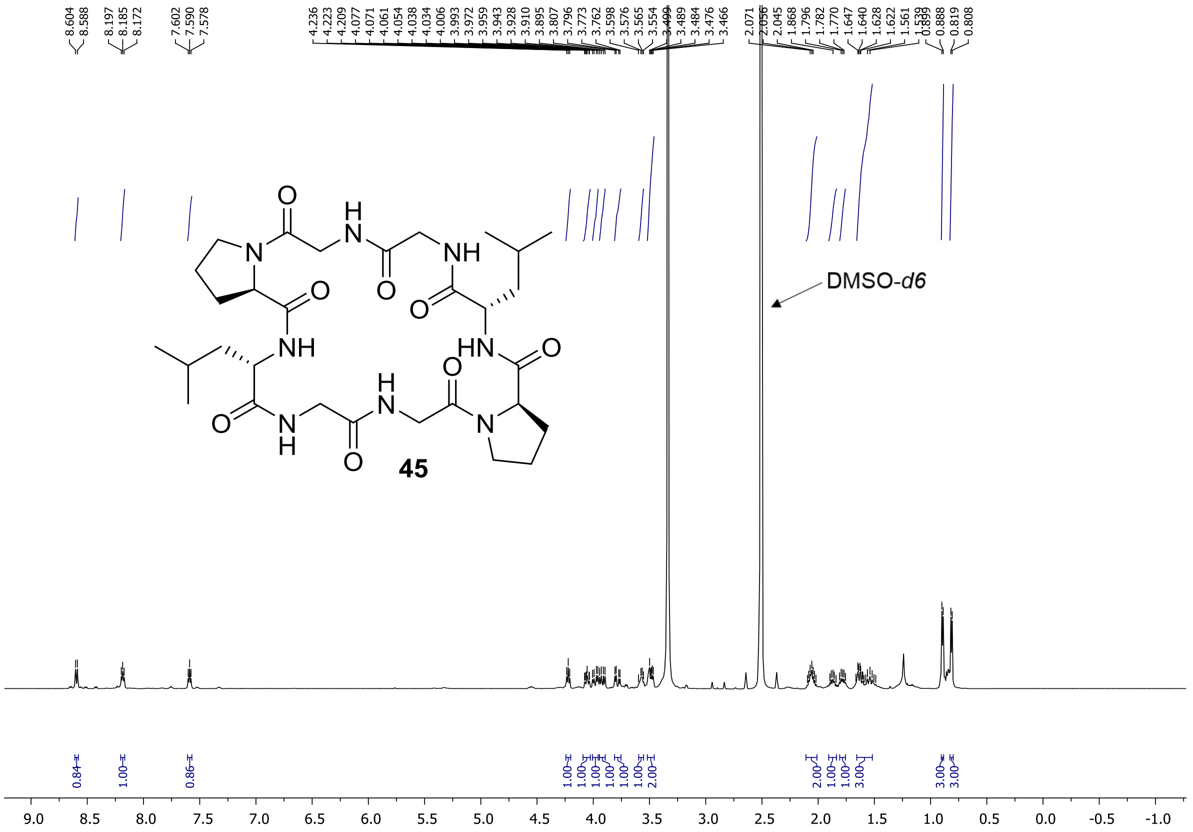

^{13}C NMR (126 MHz, DMSO-*d6*)

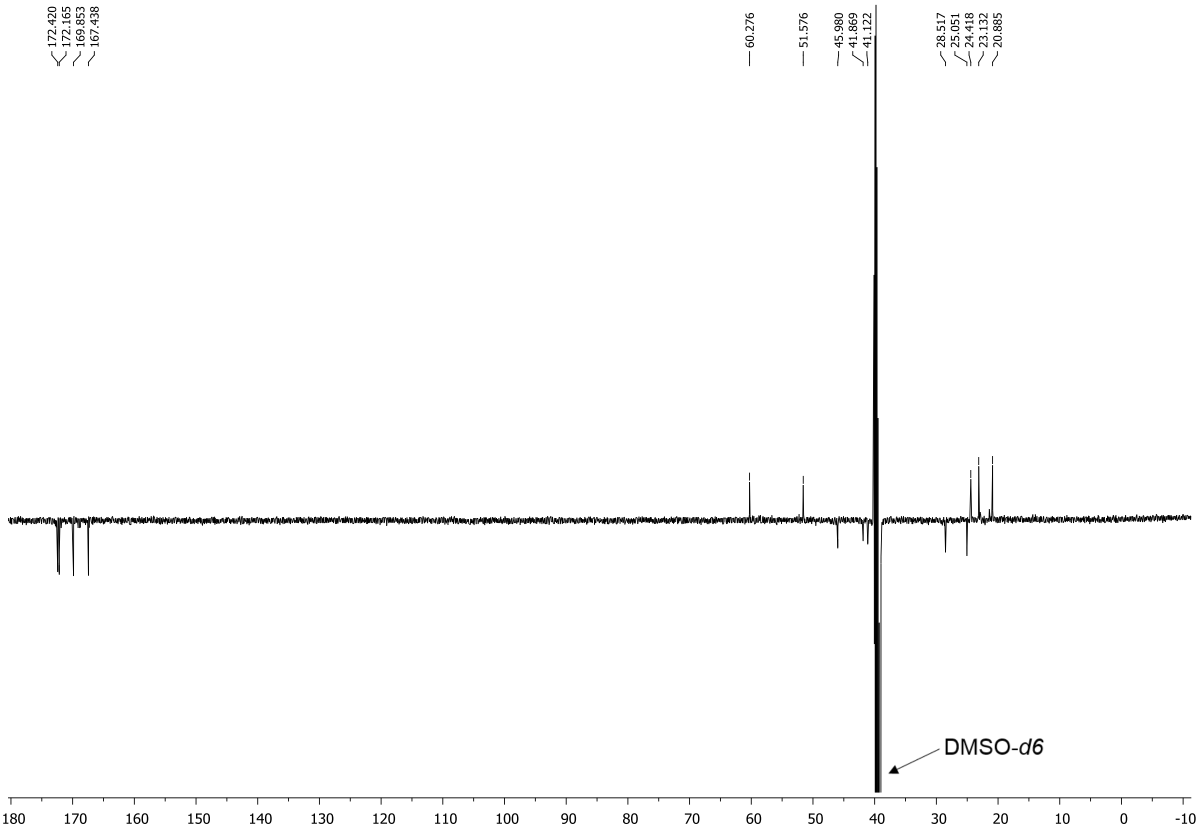

Cbz-Asp(*t*Bu)-Leu-Gly-Gly-OBn (46)

^{1}H NMR (500 MHz, $CDCl_3$)

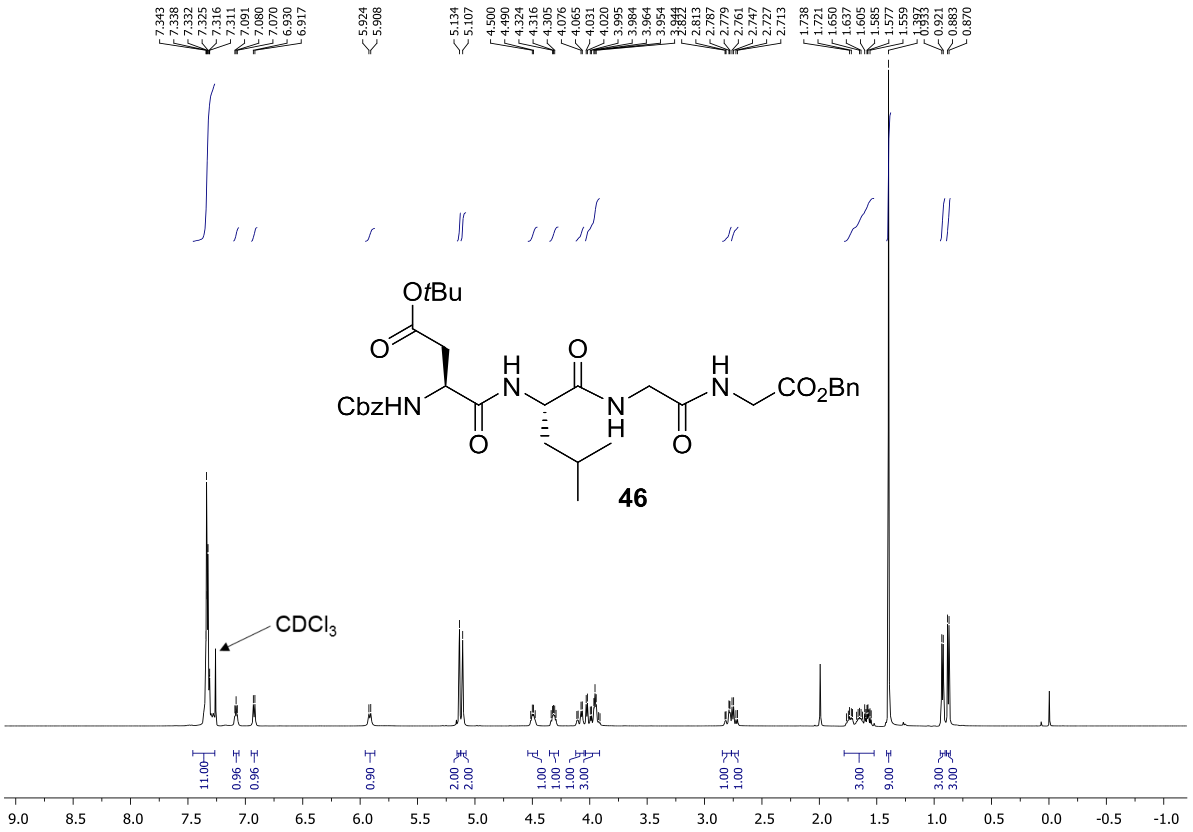

^{13}C NMR (126 MHz, $CDCl_3$)

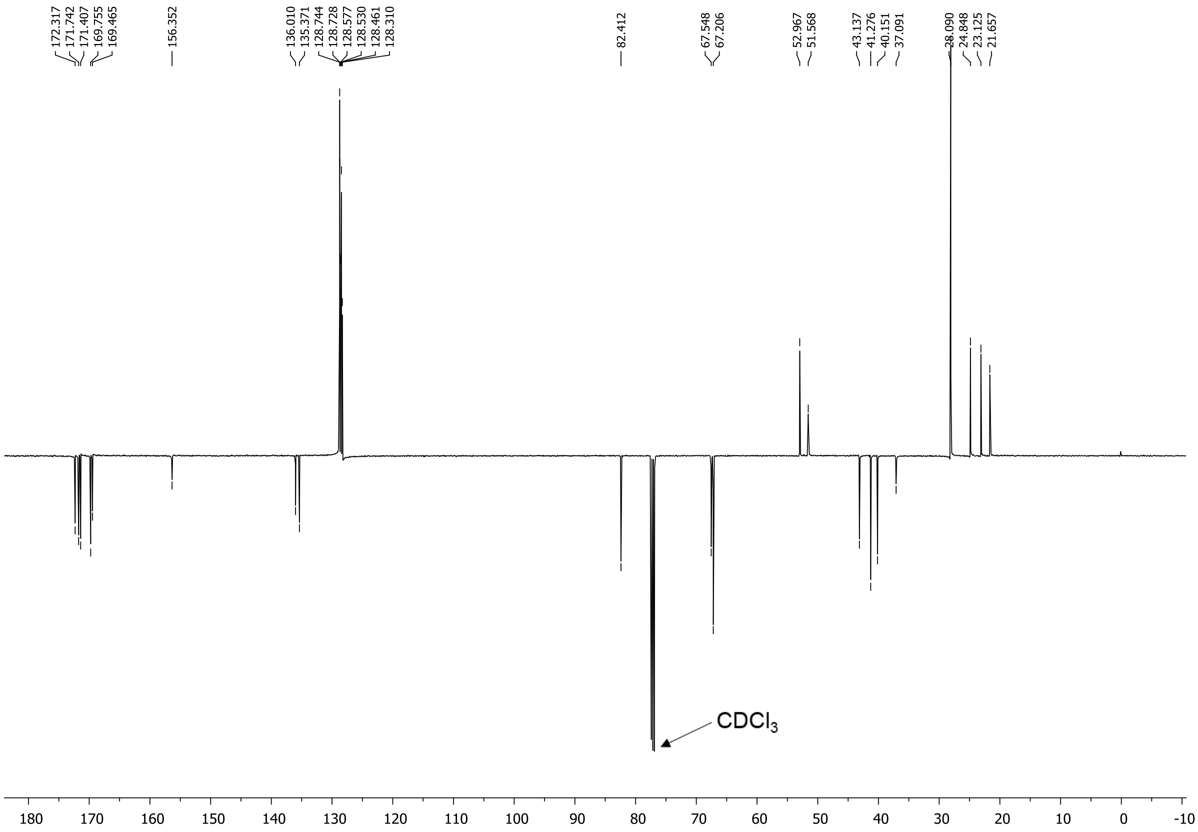

H-Asp(*t*Bu)-Leu-Gly-Gly-OBn (47)

^{1}H NMR (500 MHz, CD_3OD)

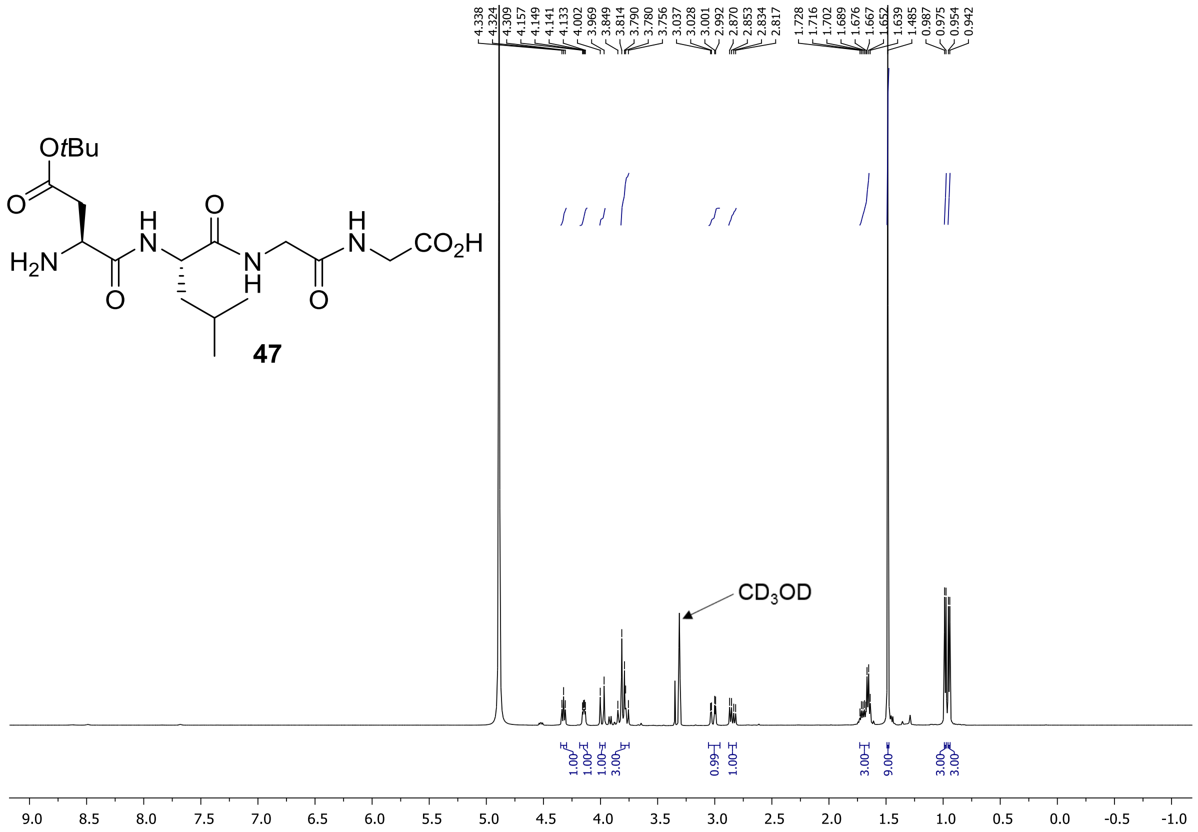

^{13}C NMR (126 MHz, CD_3OD)

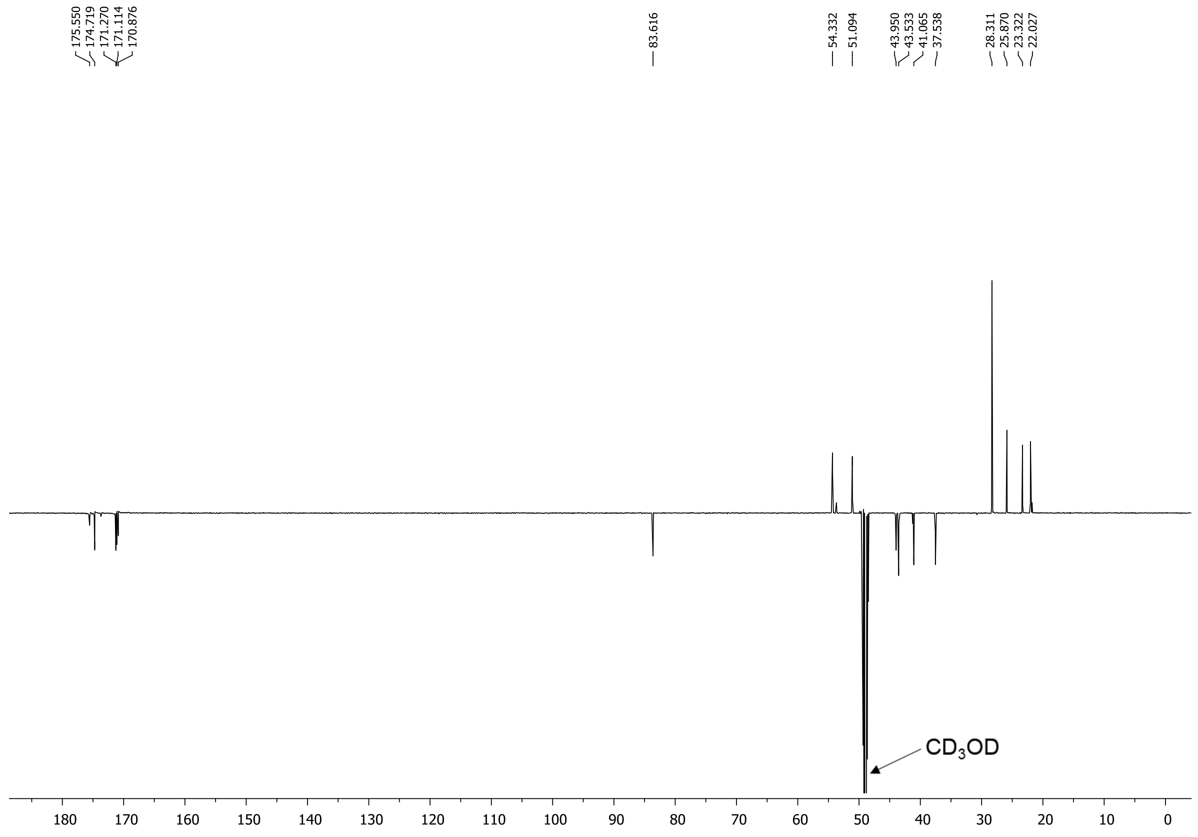

Cyclo(Asp(*t*Bu)-Leu-GOx-Gly) (48)

^{1}H NMR (500 MHz, CD_3OD)

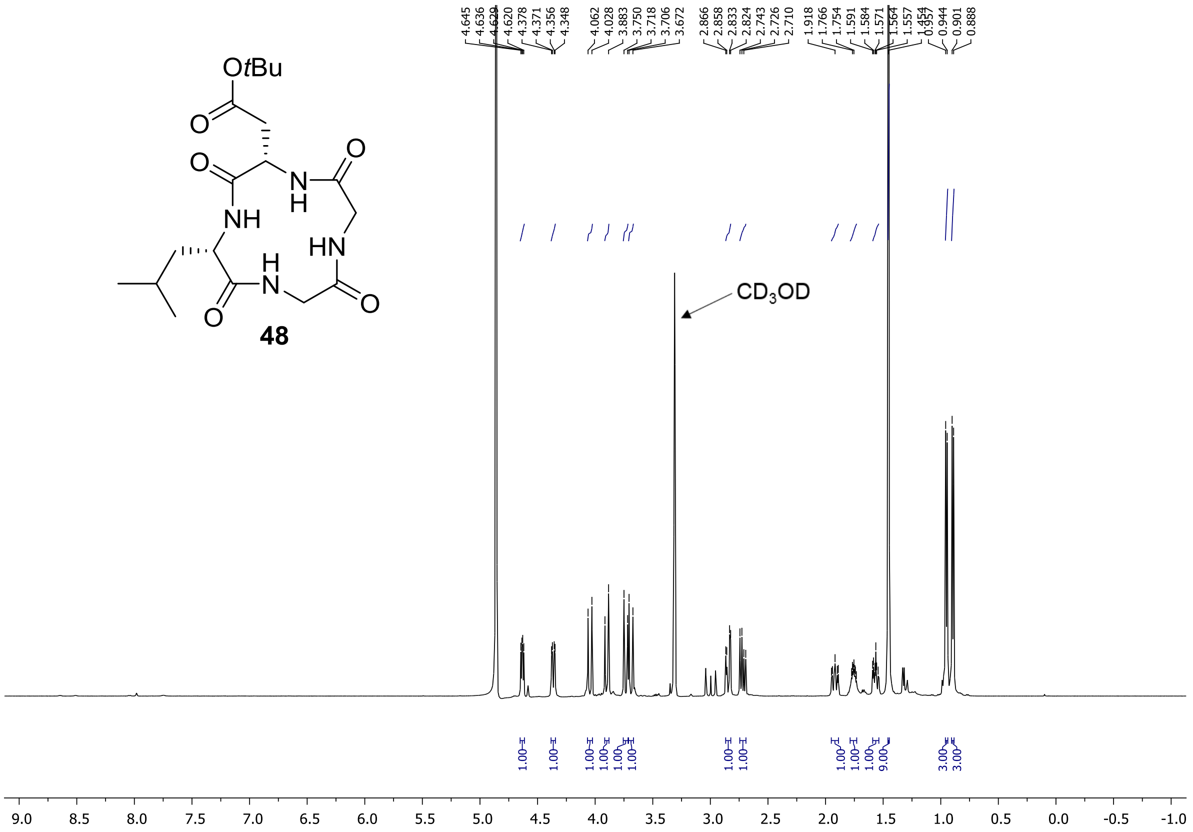

^{13}C NMR (126 MHz, CD_3OD)

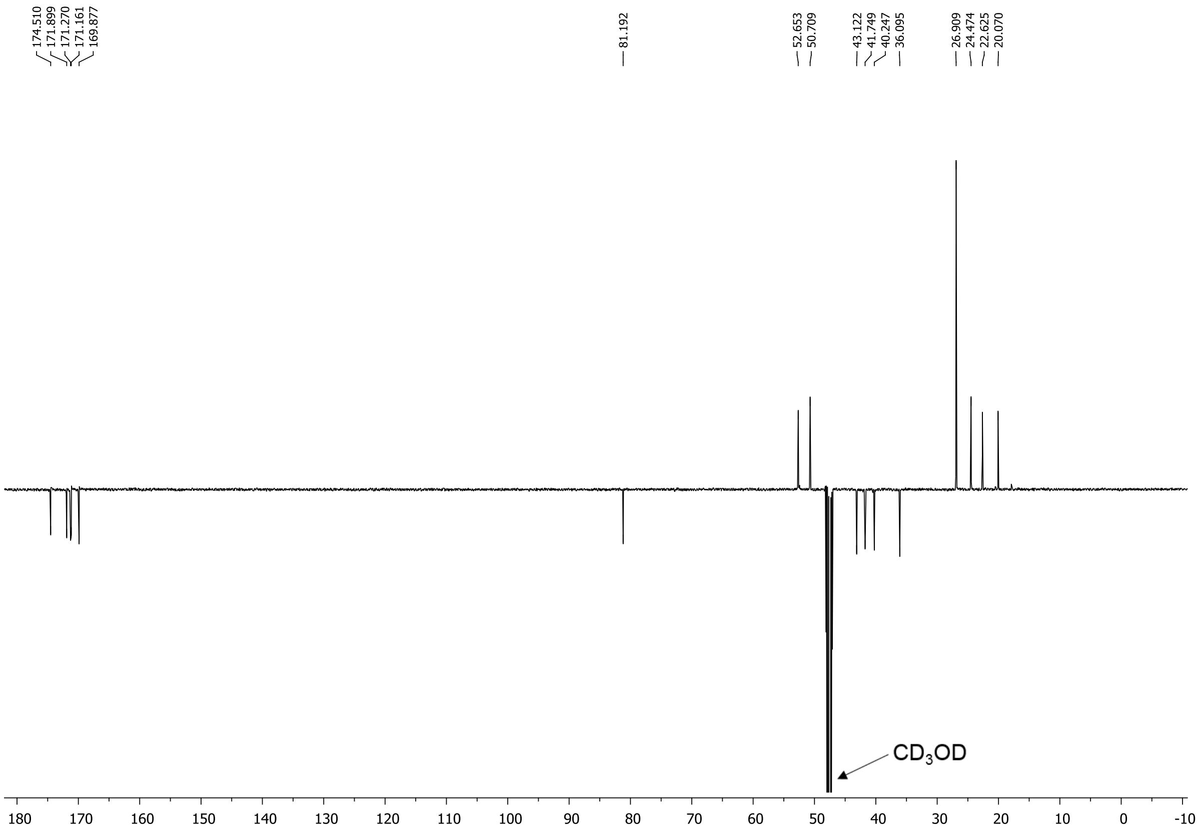

Boc-AOx-(*R*)-CH(Me)Ph (49)

^{1}H NMR (500 MHz, $CDCl_3$)

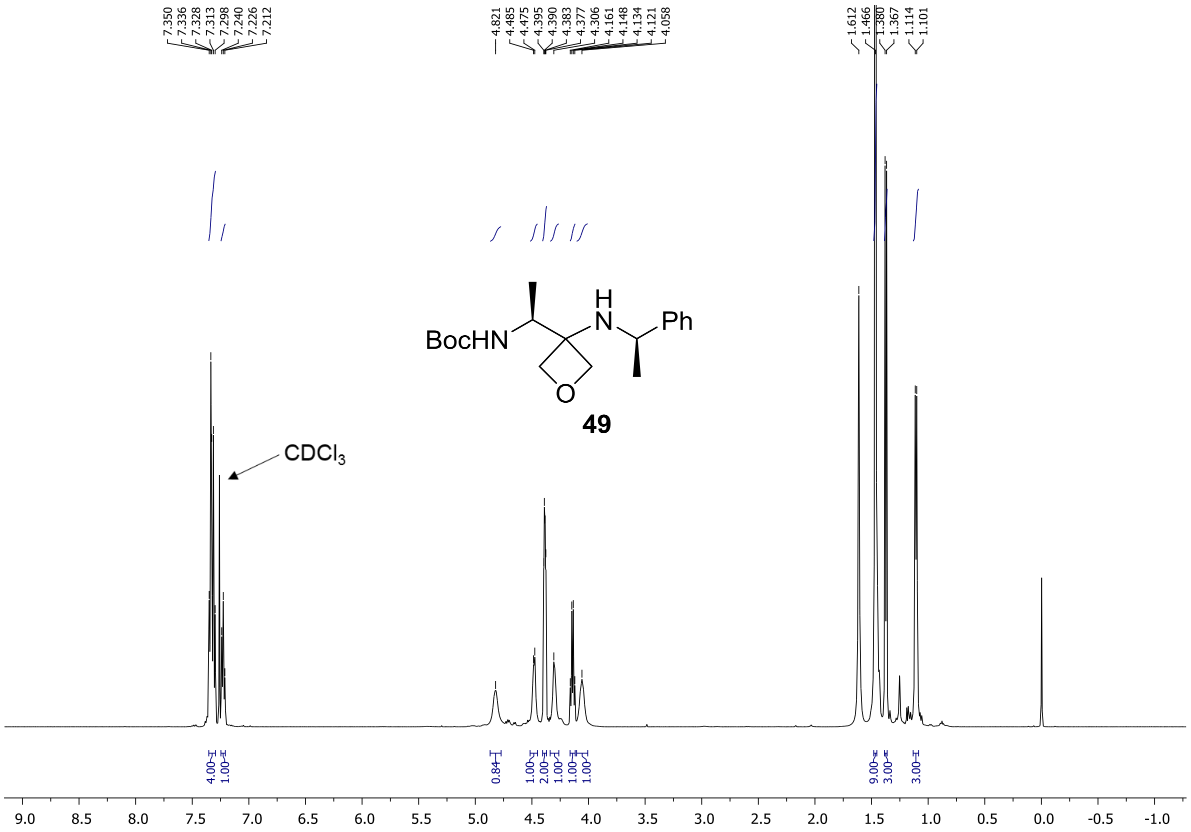

^{13}C NMR (126 MHz, $CDCl_3$)

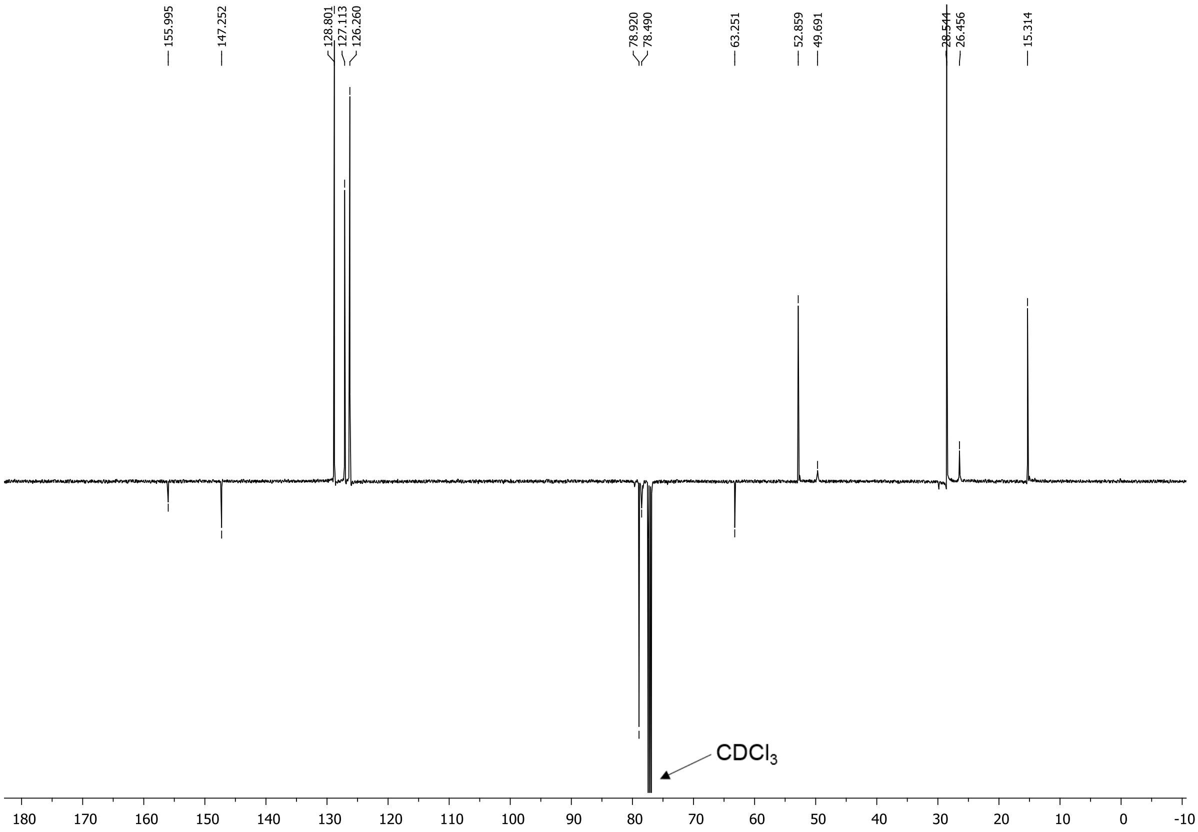

Boc-AOx-Gly-OBn (50)

^{1}H NMR (500 MHz, $CDCl_3$)

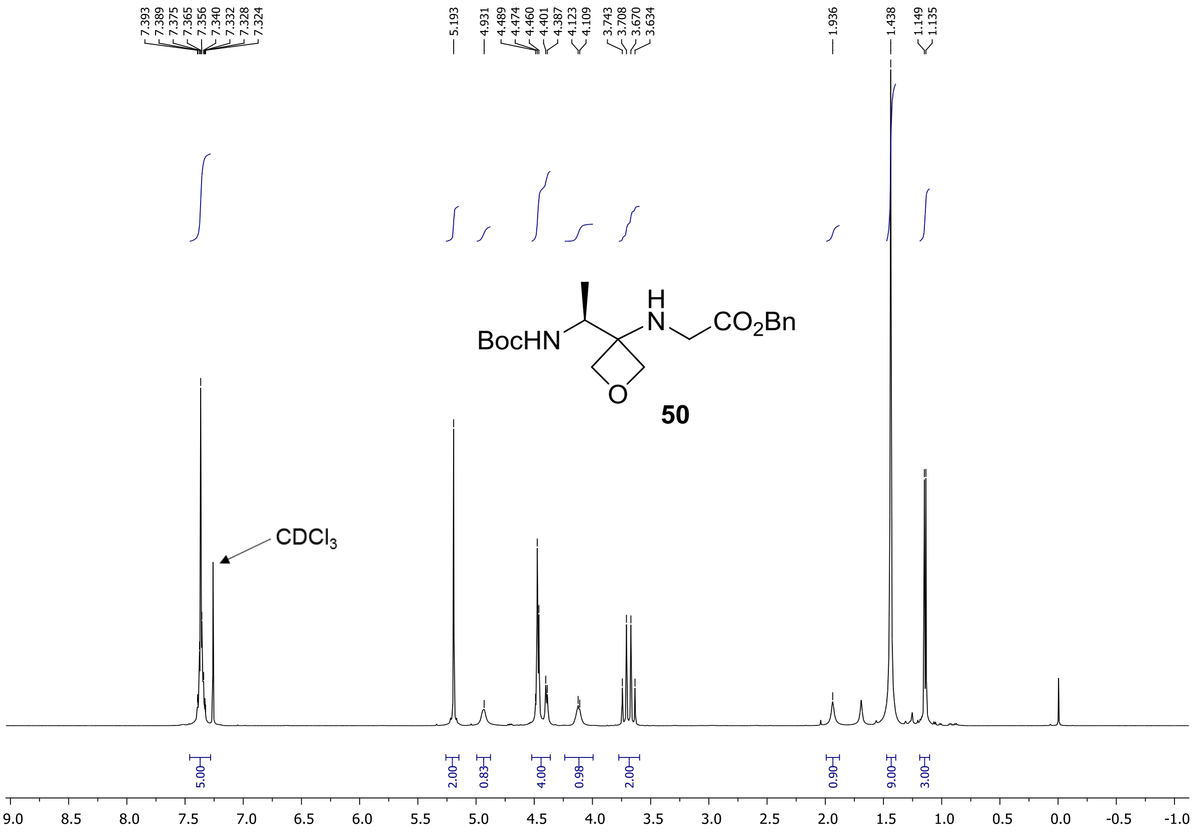

^{13}C NMR (126 MHz, $CDCl_3$)

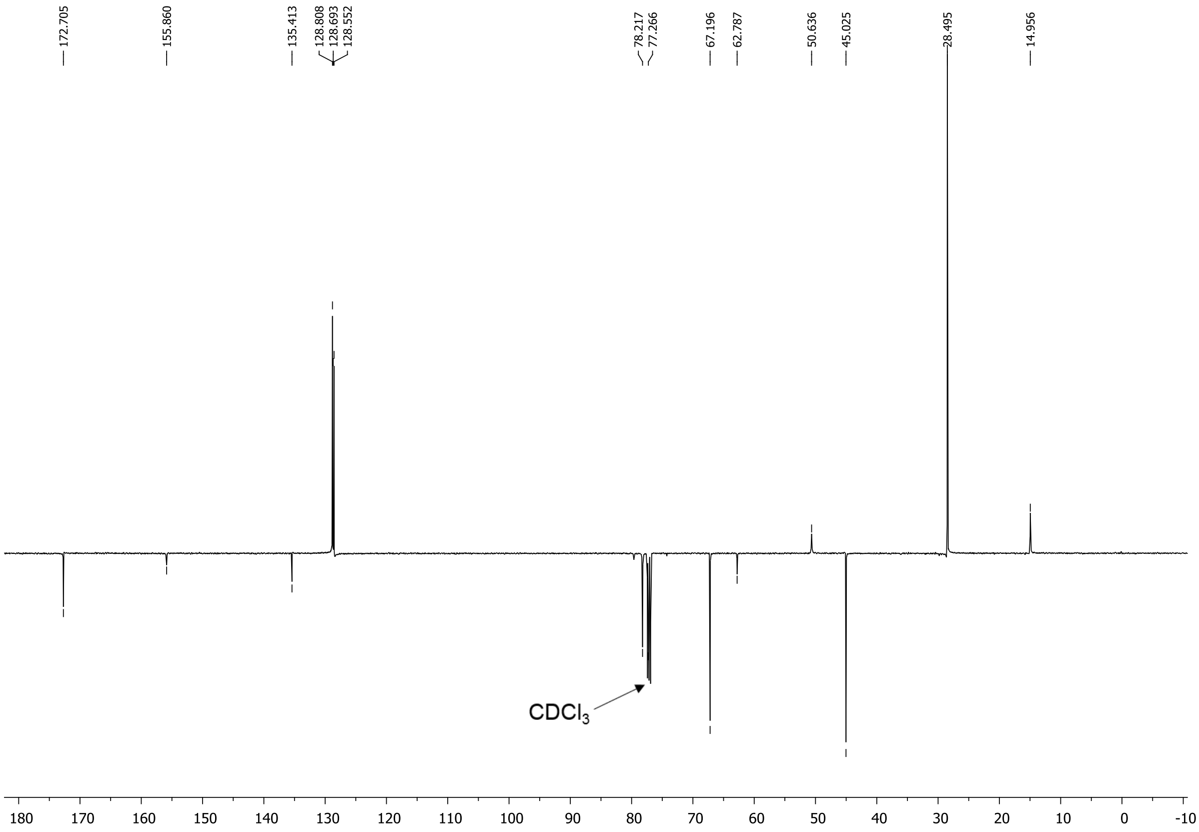

Boc-Leu-AOx-Gly-OBn (51)

^{1}H NMR (500 MHz, $CDCl_3$)

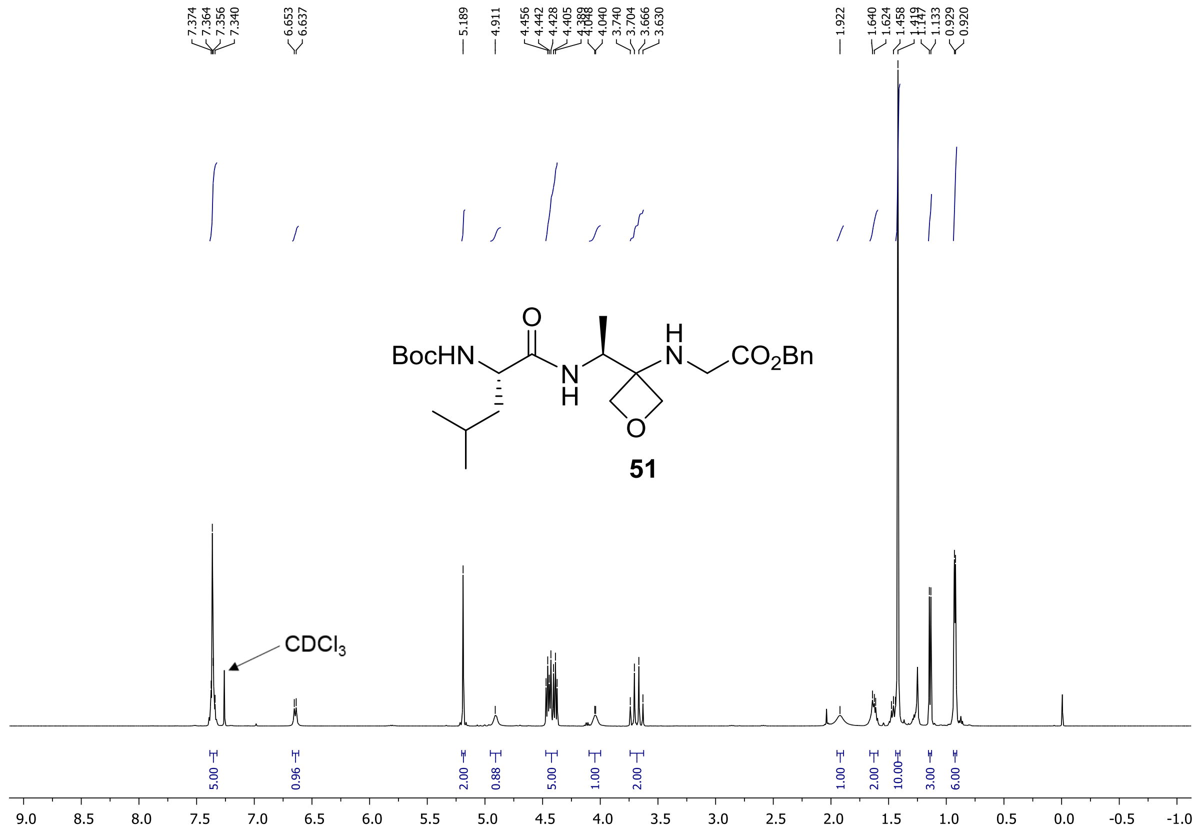

^{13}C NMR (126 MHz, $CDCl_3$)

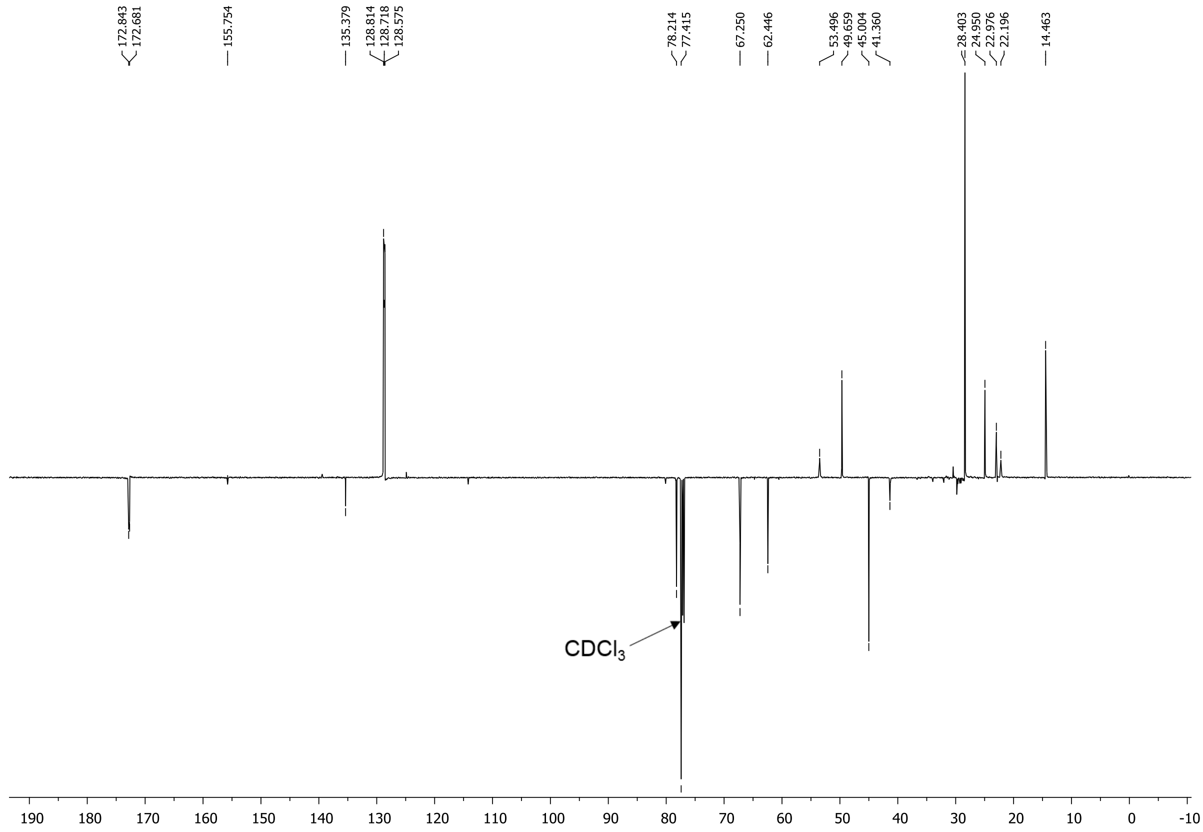

Cbz-Trp-Leu-AOx-Gly-OBn (52)

^{1}H NMR (500 MHz, $CDCl_3$)

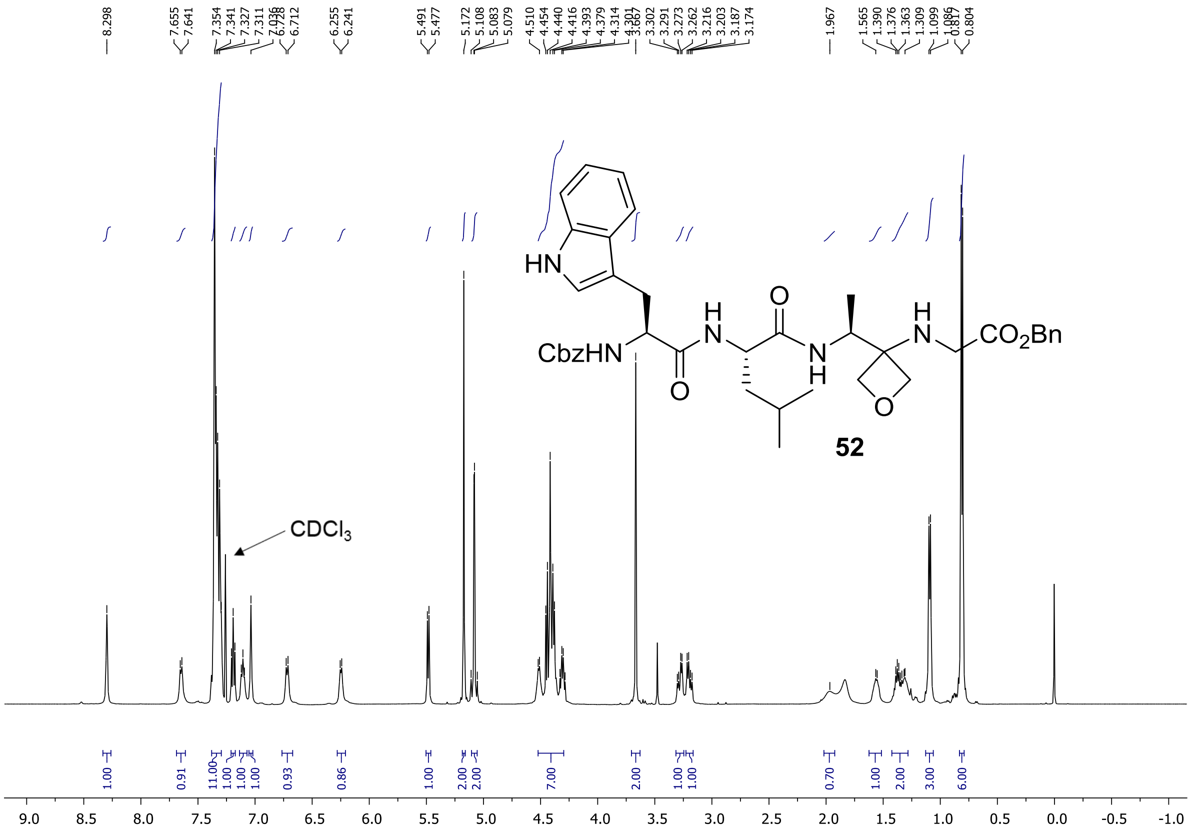

^{13}C NMR (126 MHz, $CDCl_3$)

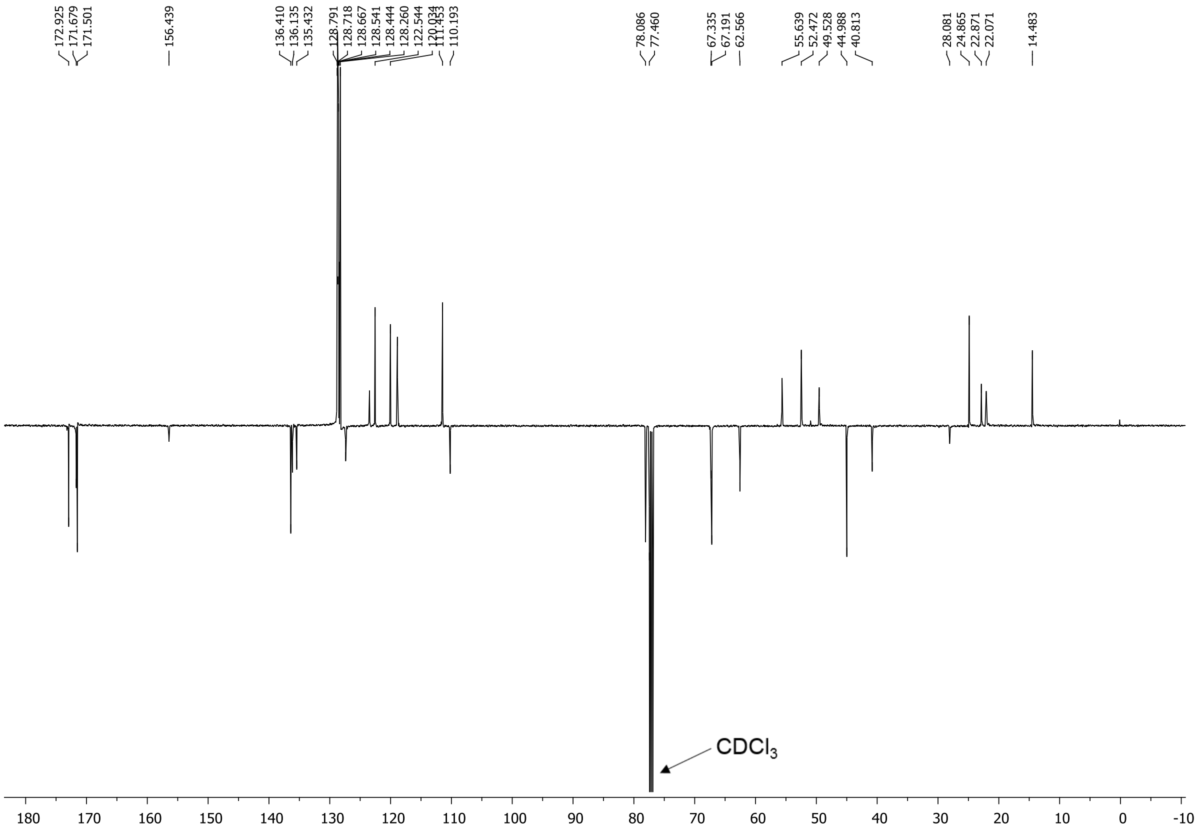

H-Trp-Leu-AOx-Gly-OH (53)

^{1}H NMR (500 MHz, CD_3OD)

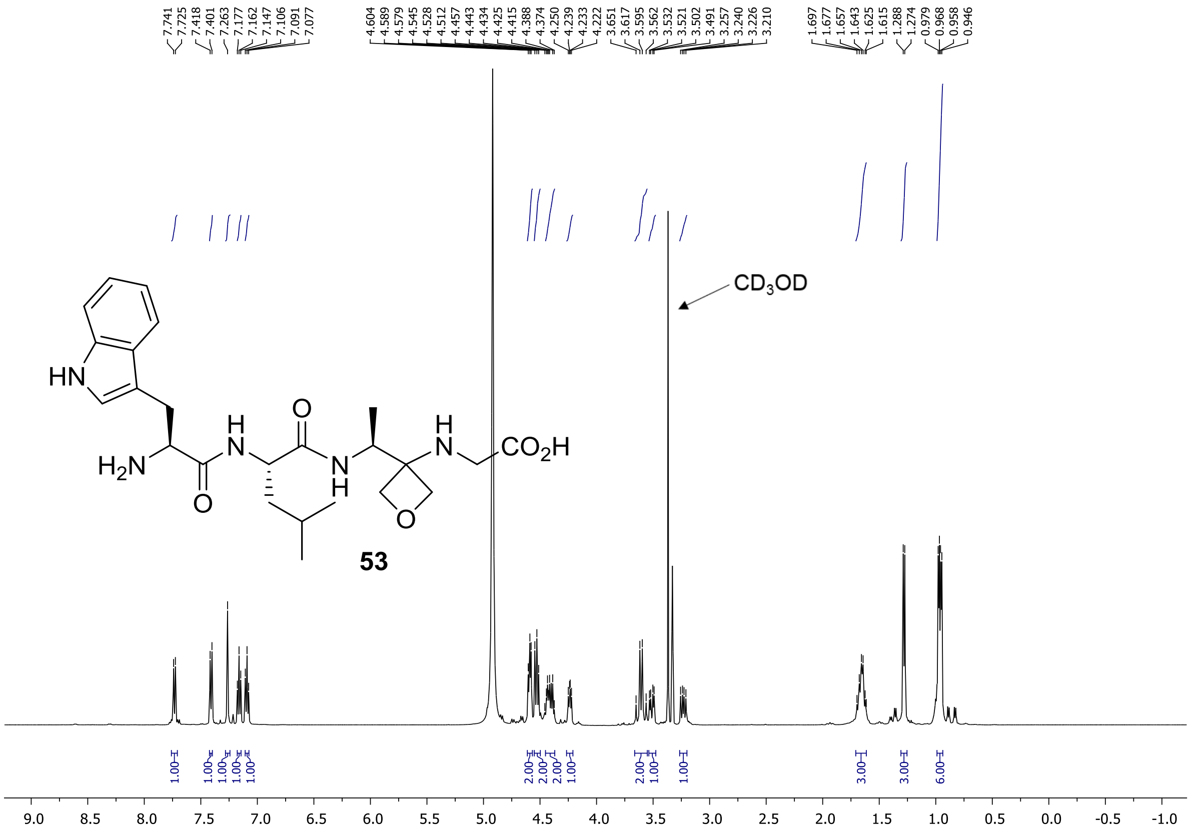

^{13}C NMR (126 MHz, CD_3OD)

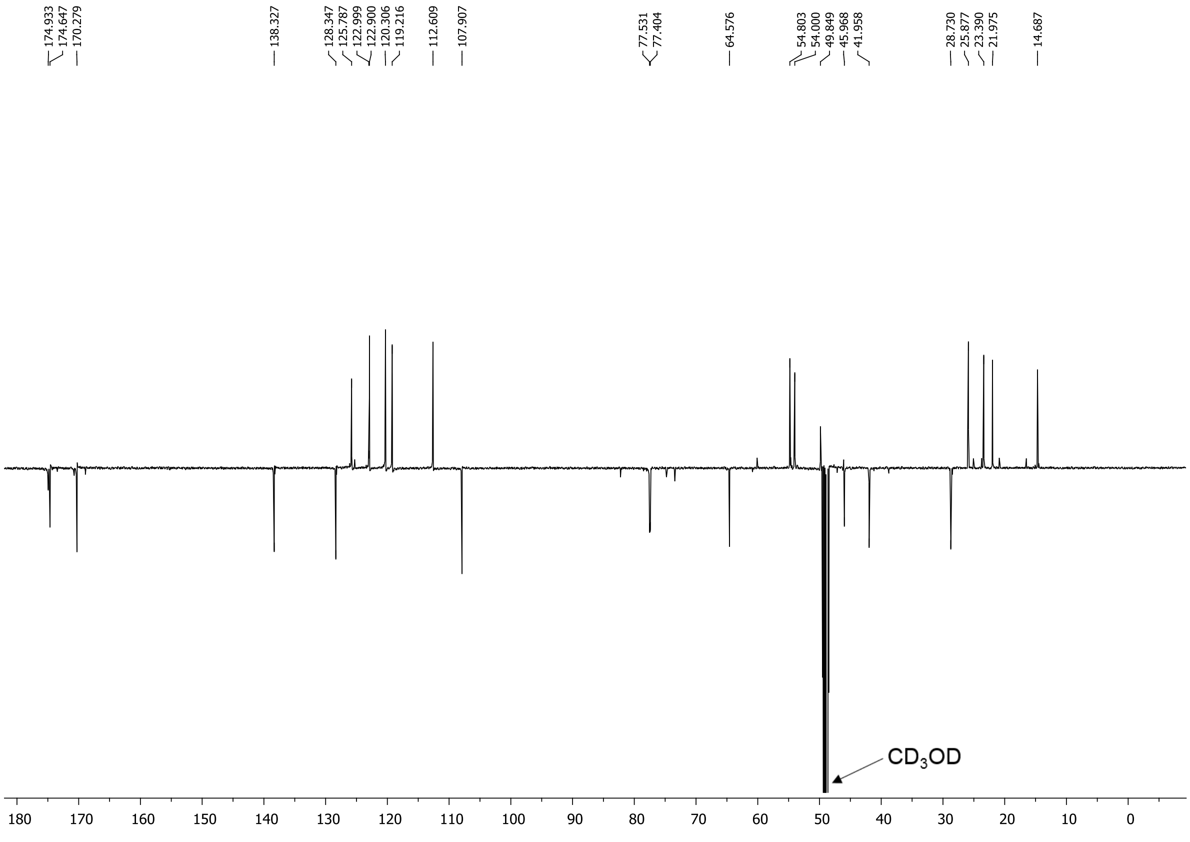

Cyclo(Trp-Leu-AOx-Gly) (12)

^{1}H NMR (500 MHz, CD_3OD)

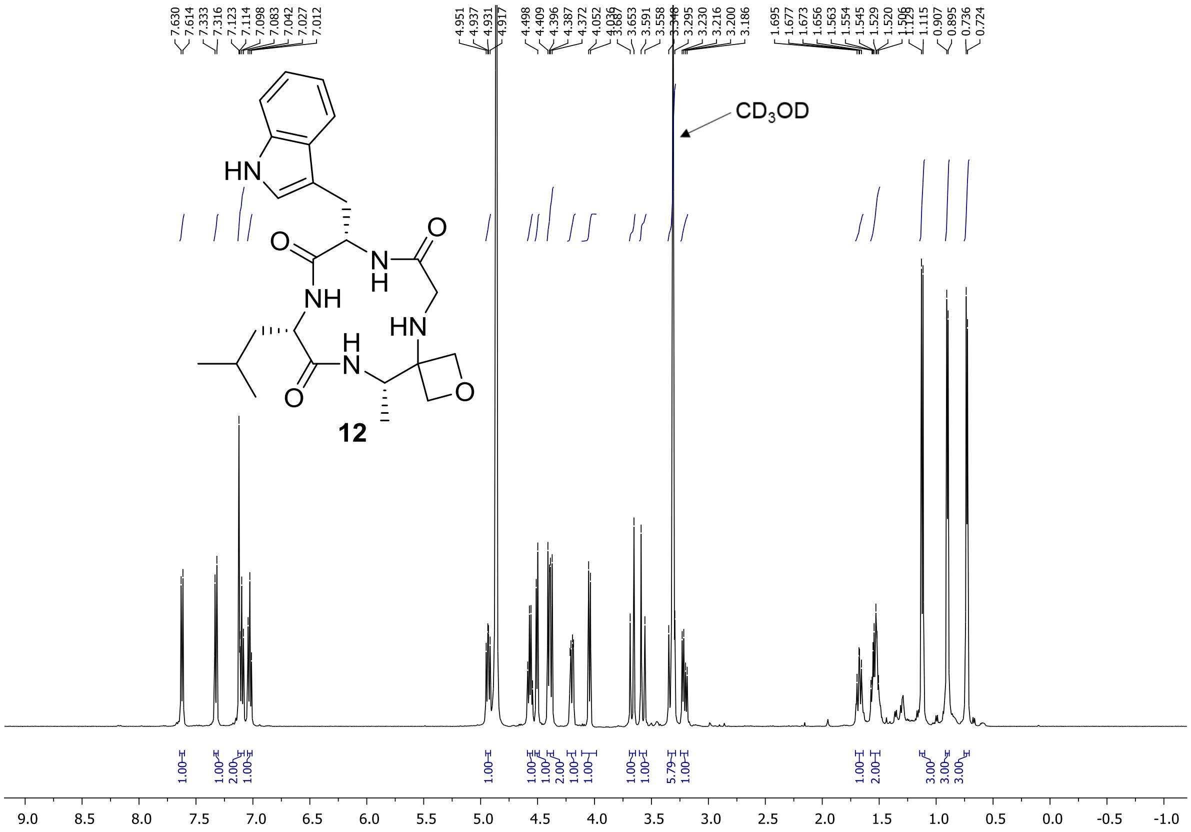

^{13}C NMR (126 MHz, CD_3OD)

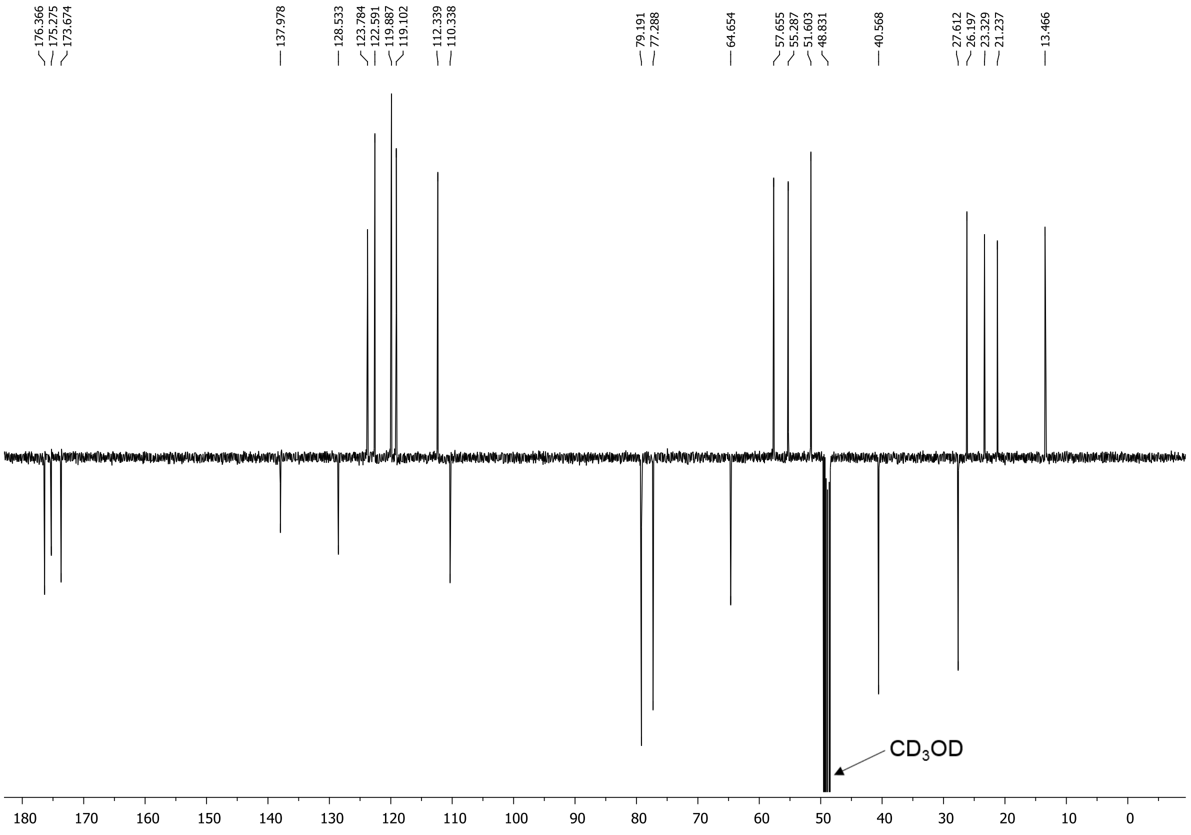

Boc-Leu-Ala-Gly-OBn (55)

^{1}H NMR (500 MHz, $CDCl_3$)

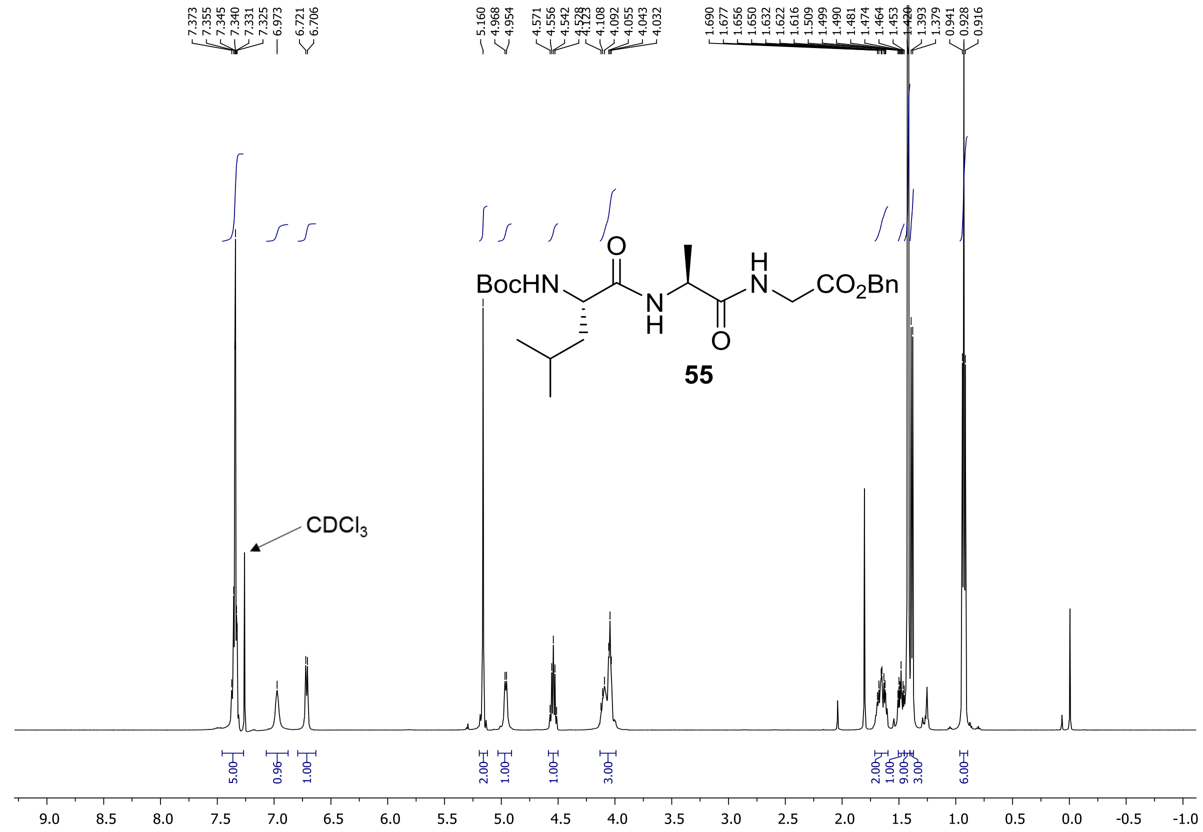

^{13}C NMR (126 MHz, $CDCl_3$)

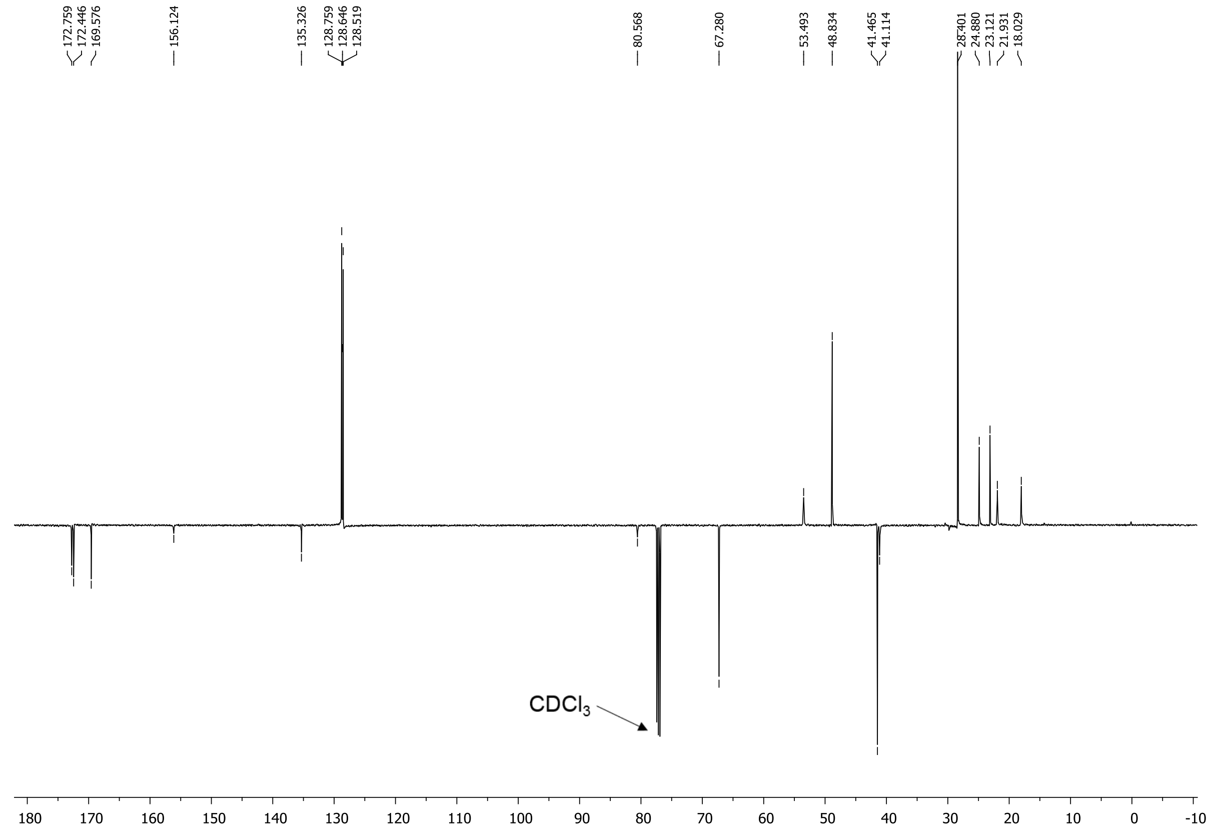

Cbz-Trp-Leu-Ala-Gly-OBn (56)

^{1}H NMR (500 MHz, CD_3OD)

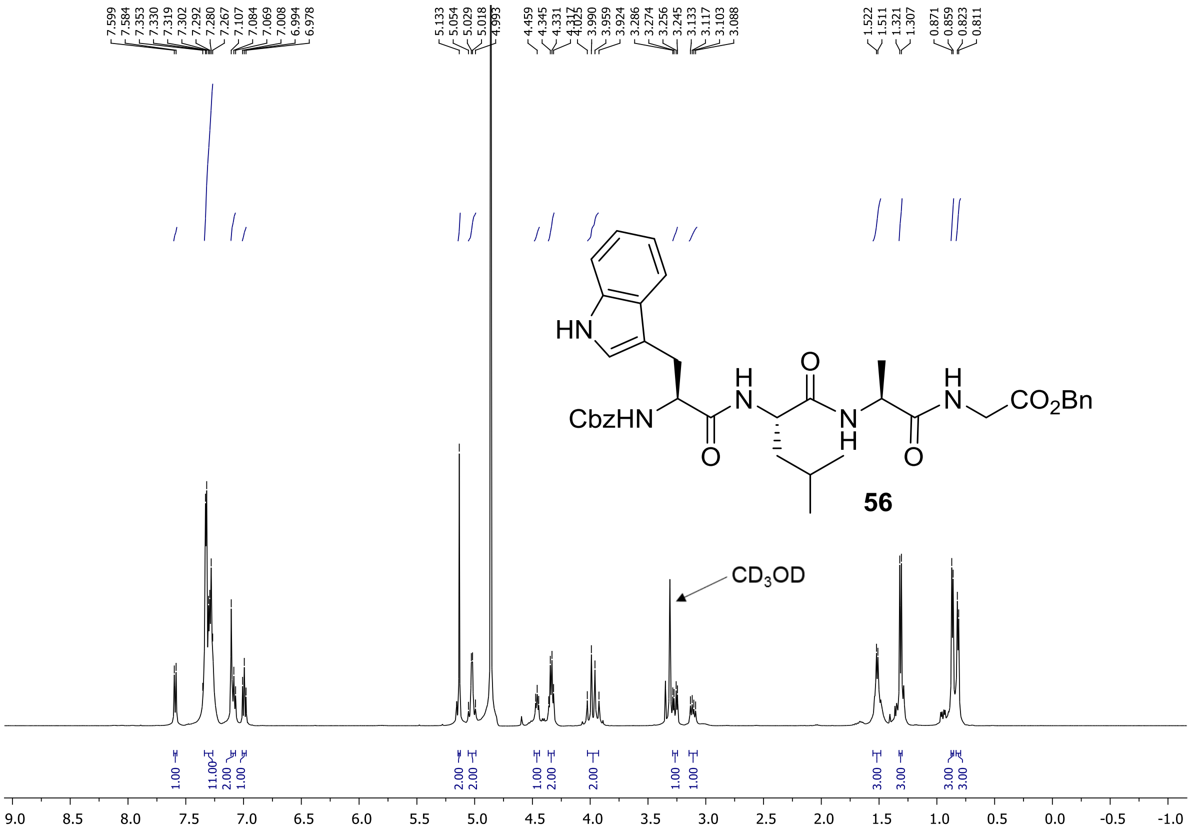

^{13}C NMR (126 MHz, CD_3OD)

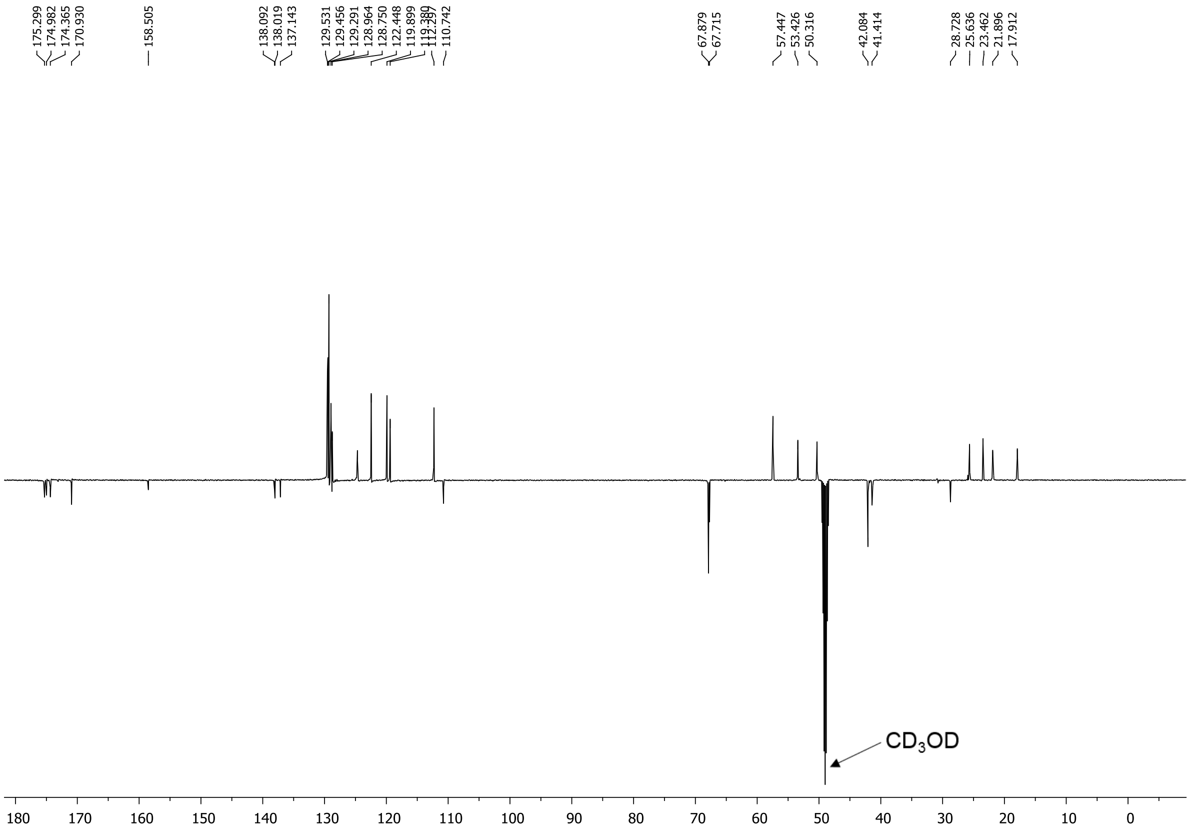

H-Trp-Leu-Ala-Gly-OH (57)

^{1}H NMR (500 MHz, D_2O)

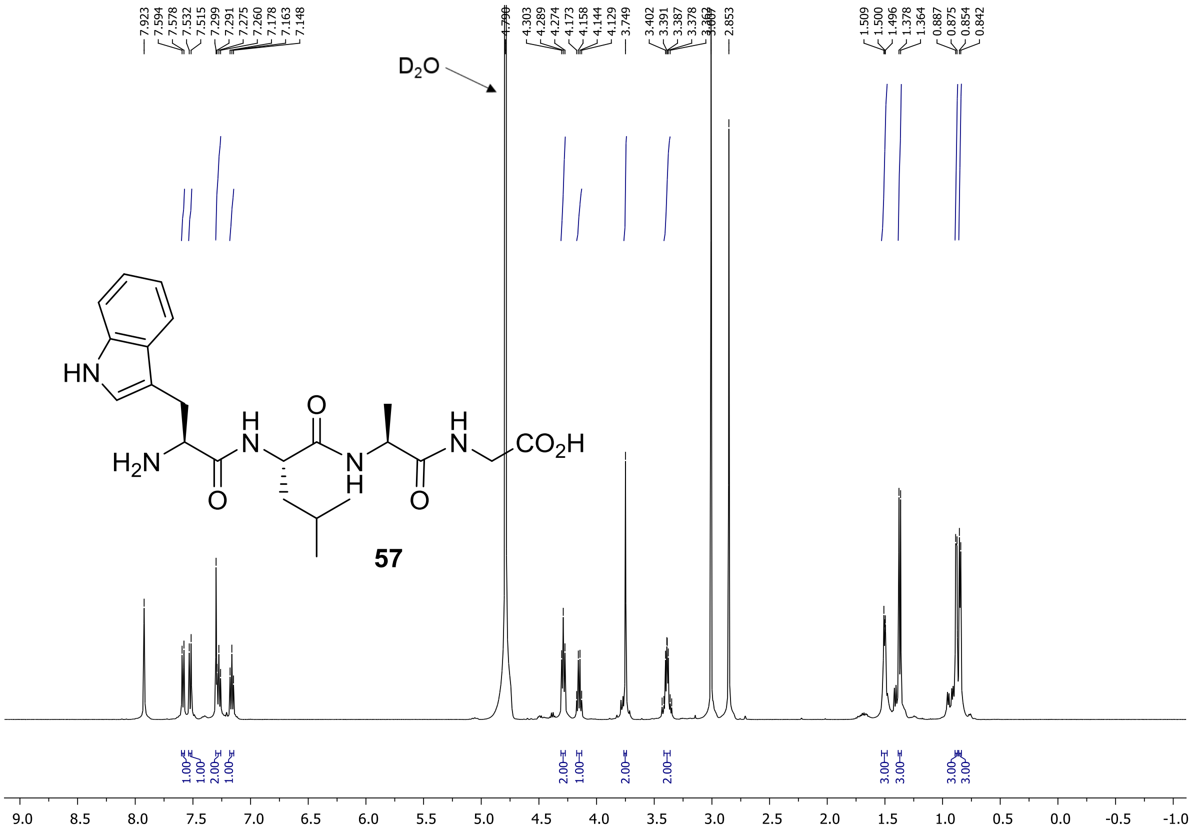

^{13}C NMR (126 MHz, D_2O)

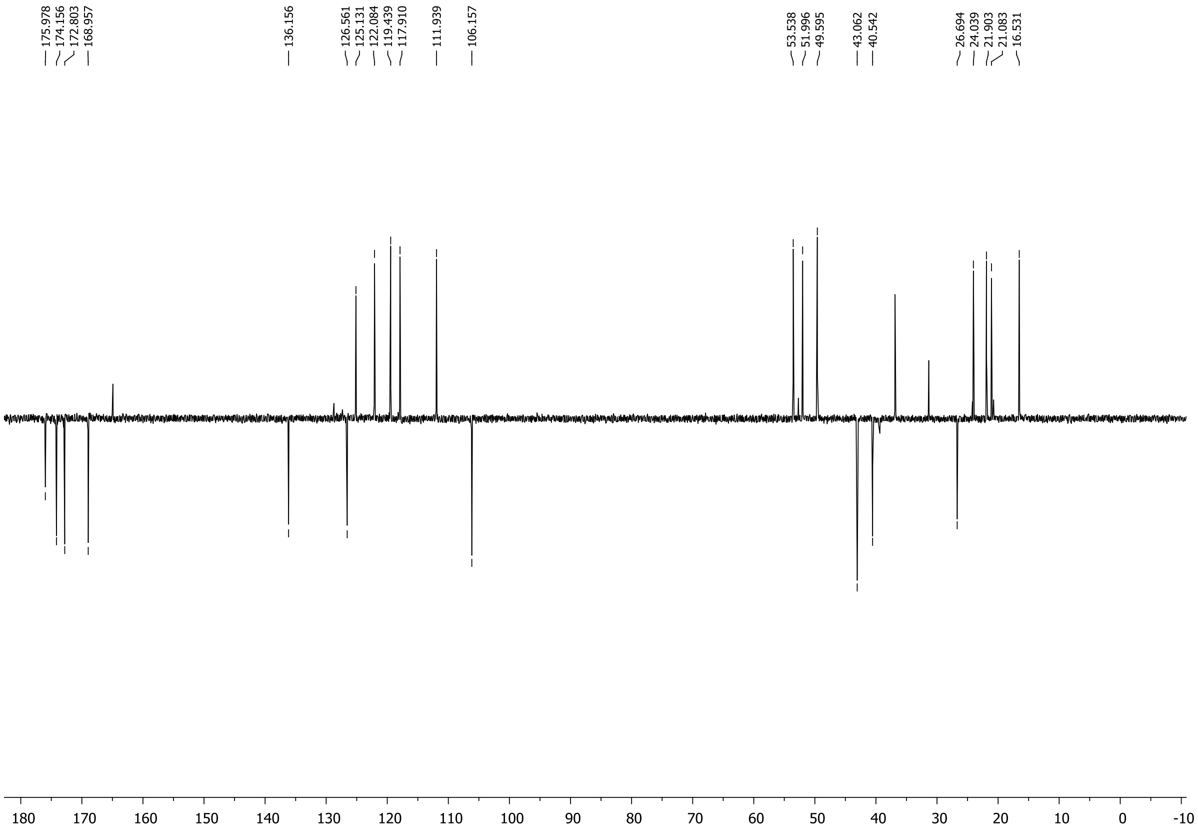

Cyclo(Trp-Leu-Ala-Gly) (58)

^{1}H NMR (500 MHz, DMSO-*d6*)

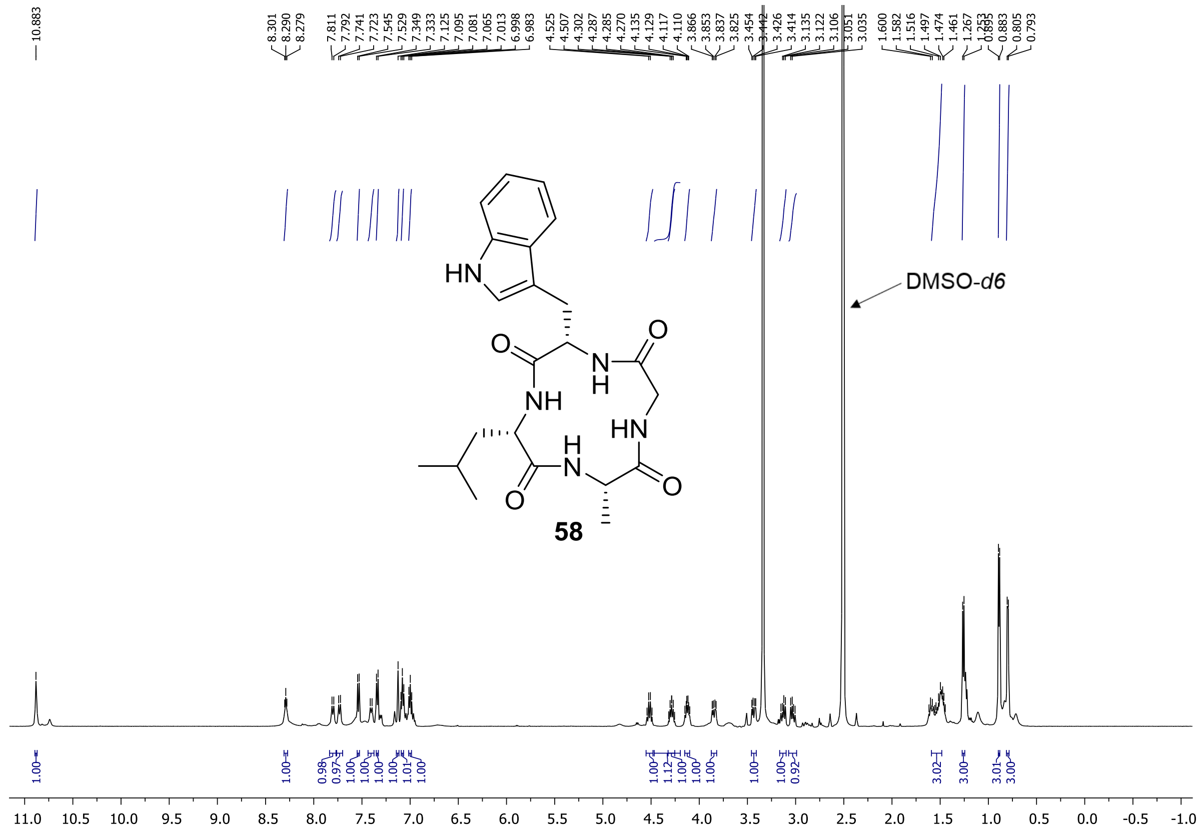

^{13}C NMR (500 MHz, DMSO-*d6*)

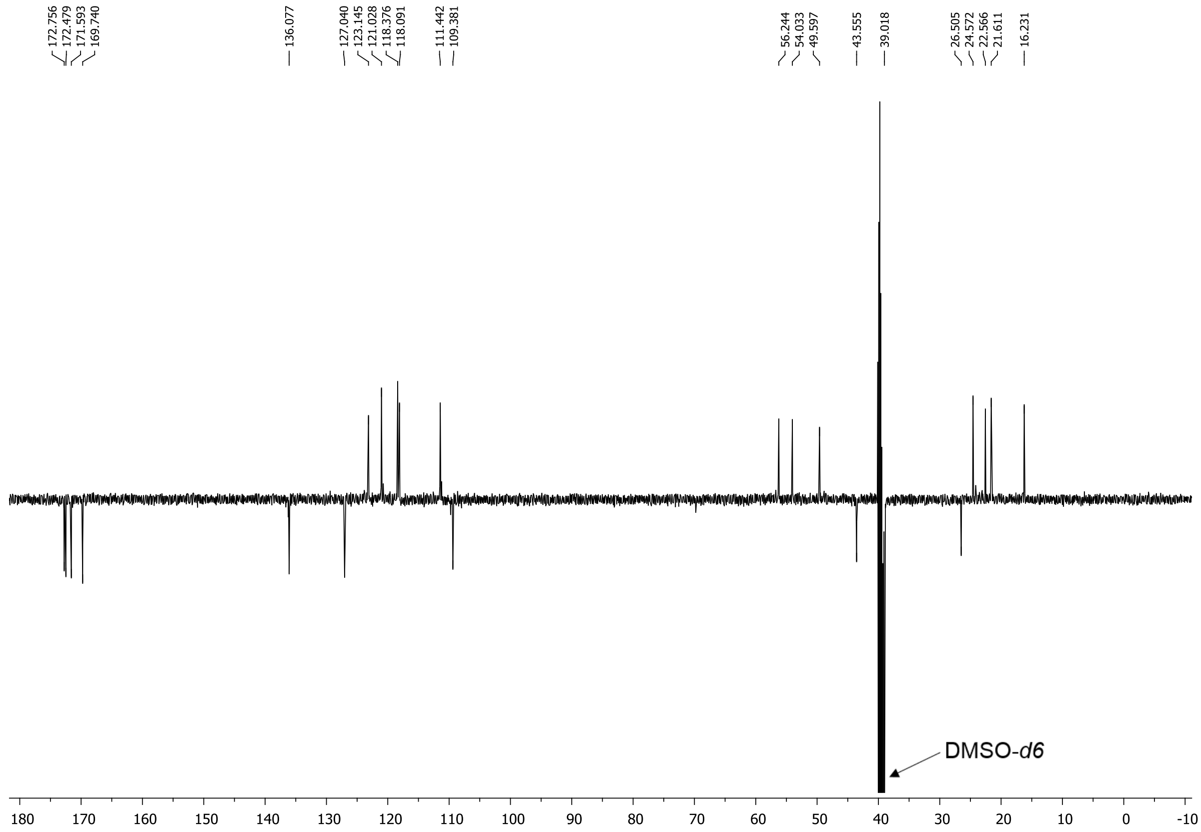

NO_2-GOx-Ala-OBn (59)

^{1}H NMR (400 MHz, $CDCl_3$)

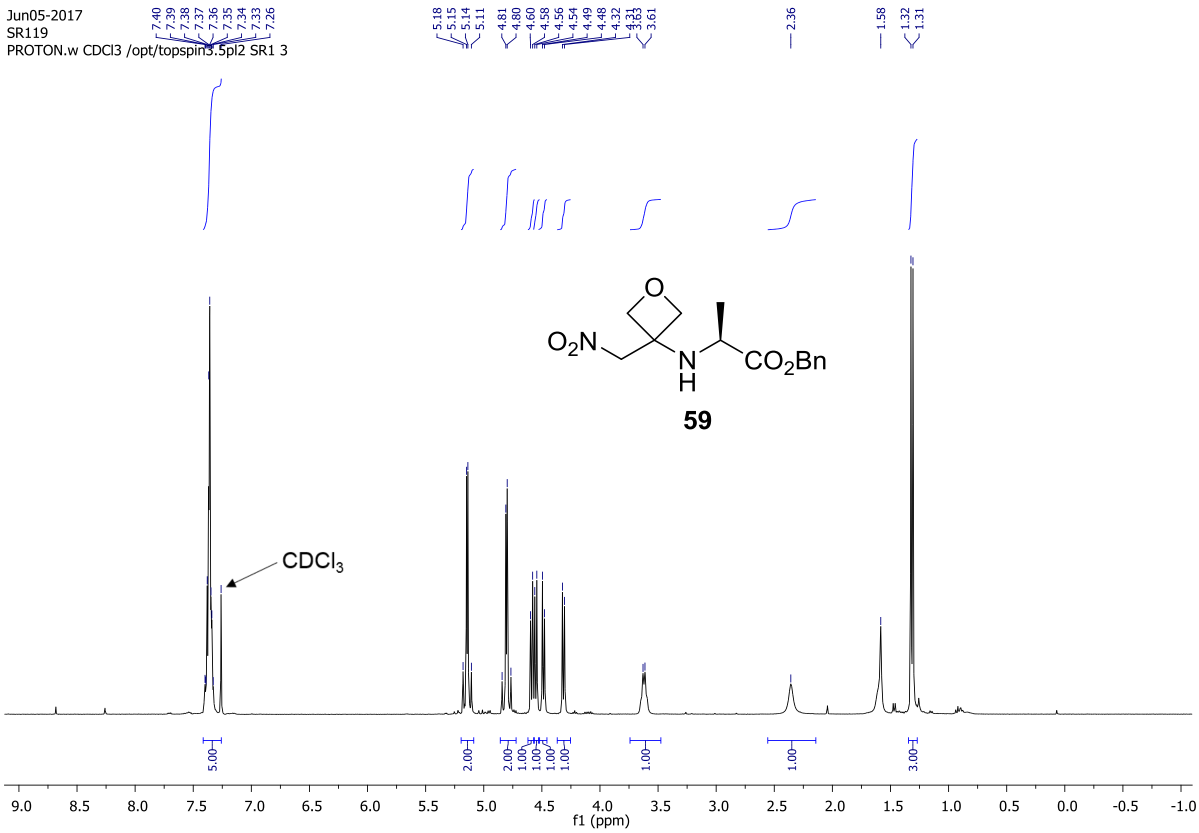

^{13}C NMR (101 MHz, $CDCl_3$)

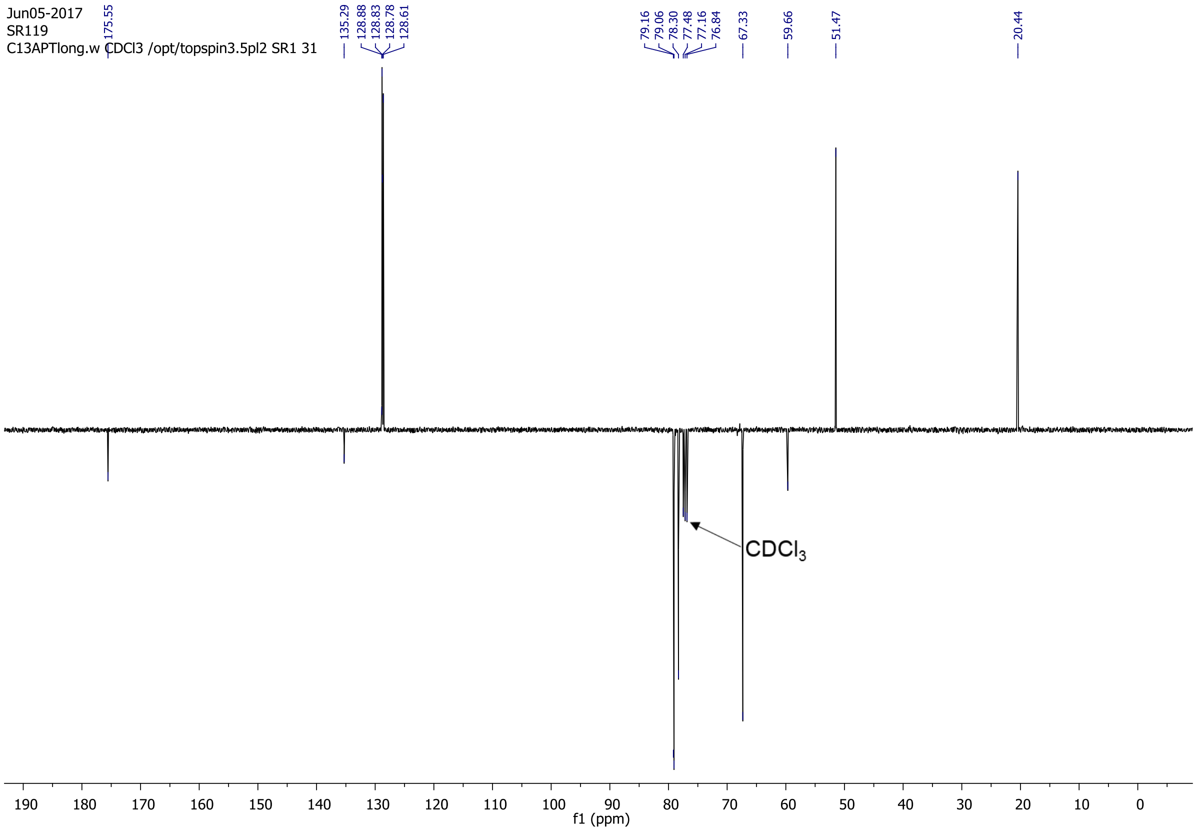

Boc-Ala-GOx-Ala-OBn (60)

^{1}H NMR (400 MHz, $CDCl_3$)

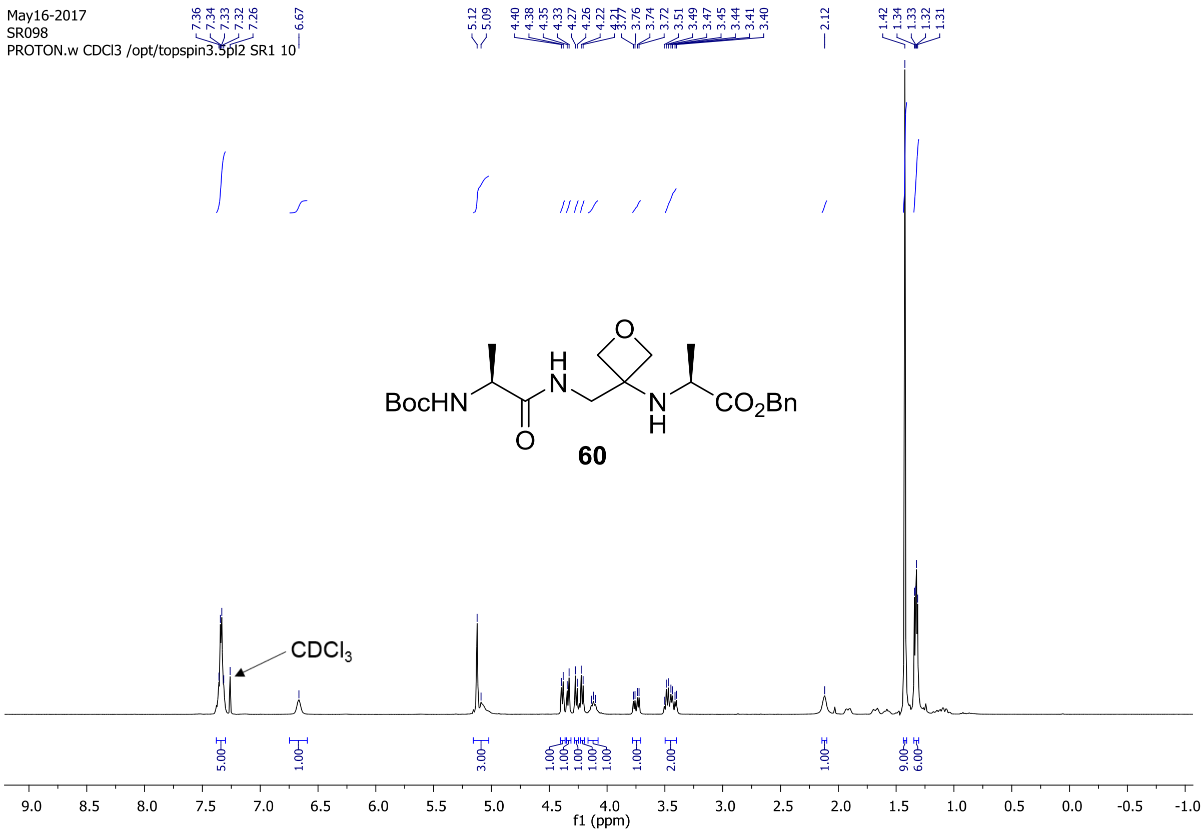

^{13}C NMR (101 MHz, $CDCl_3$)

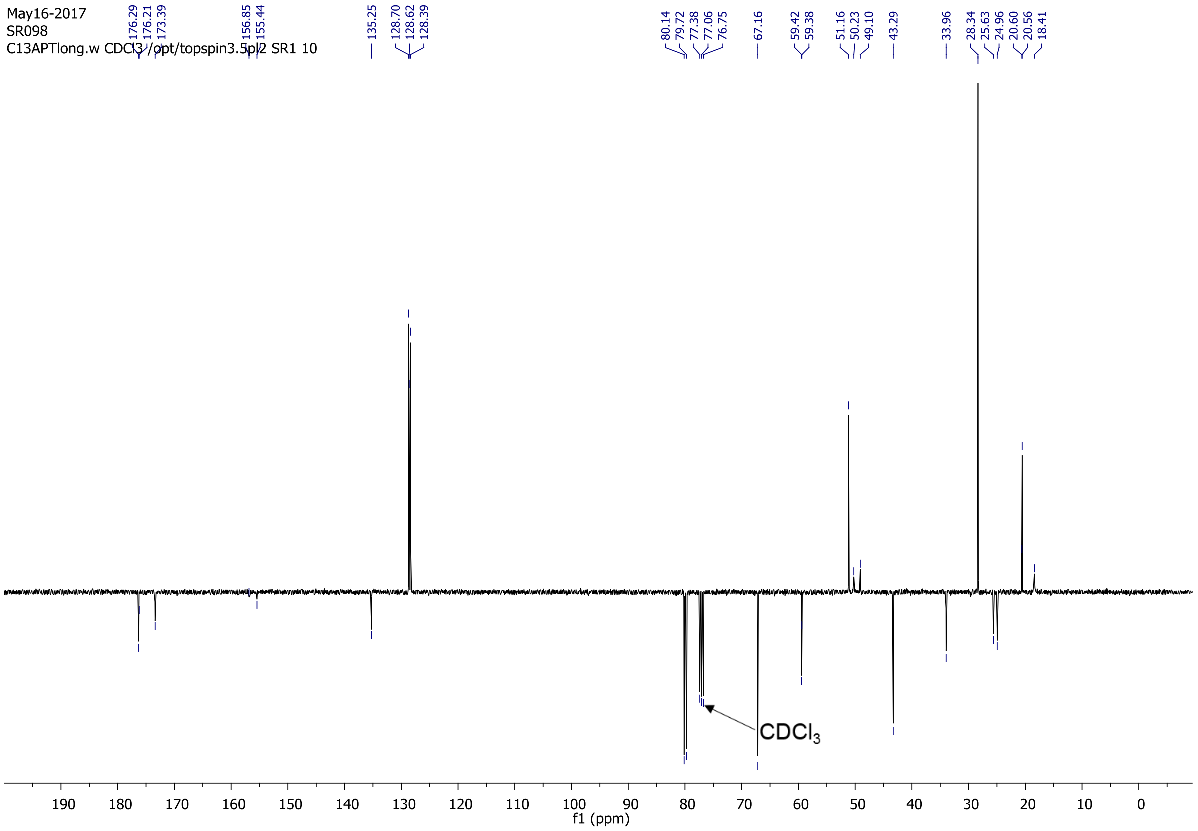

Boc-Leu-Ala-GOx-Ala-OBn (61)

^{1}H NMR (400 MHz, $CDCl_3$)

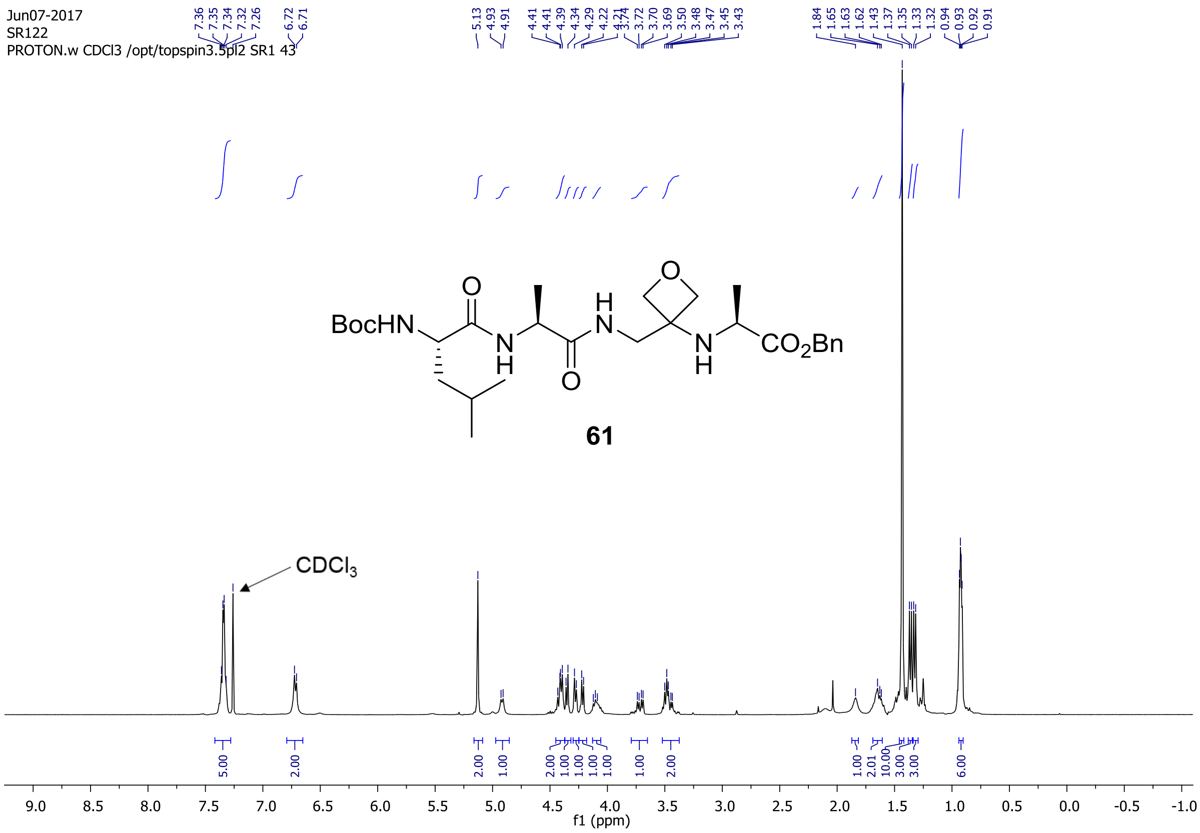

^{13}C NMR (101 MHz, $CDCl_3$)

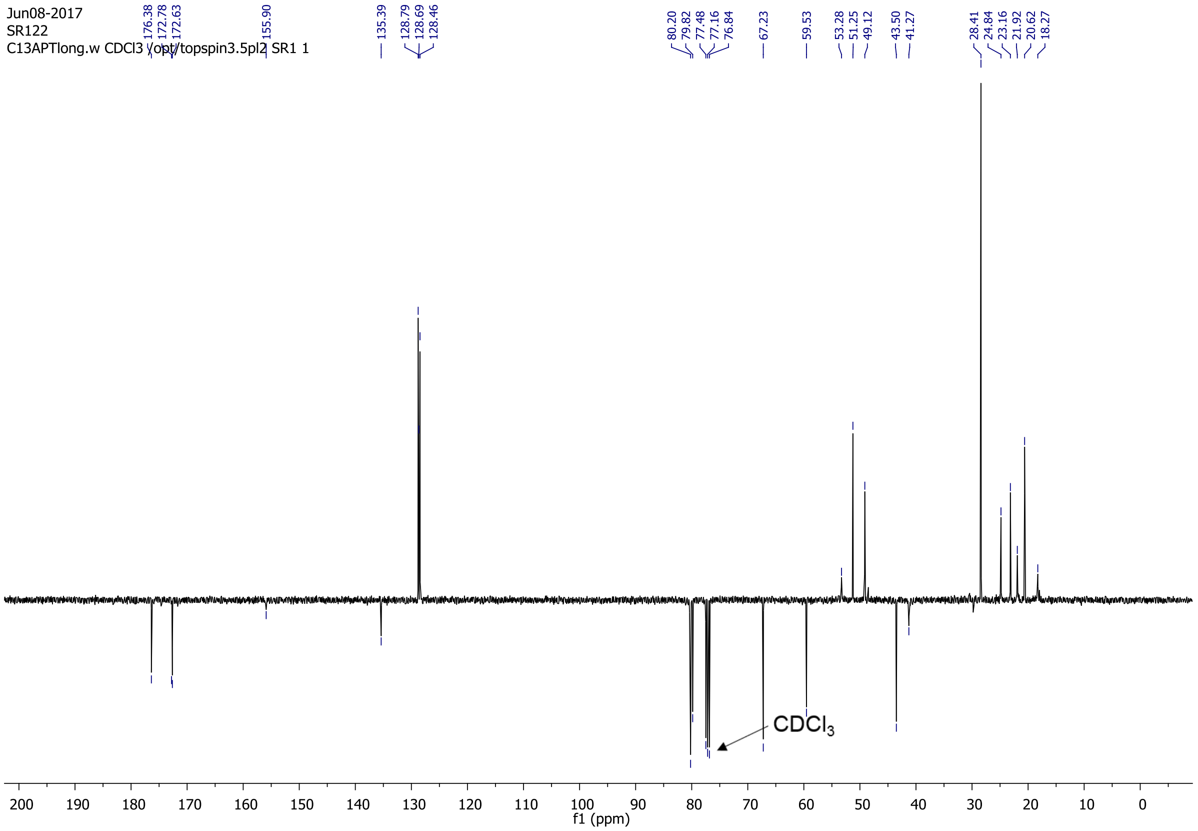

Cbz-Tyr(Bn)-Leu-Ala-GOx-Ala-OBn (62)

^{1}H NMR (400 MHz, $CDCl_3$)

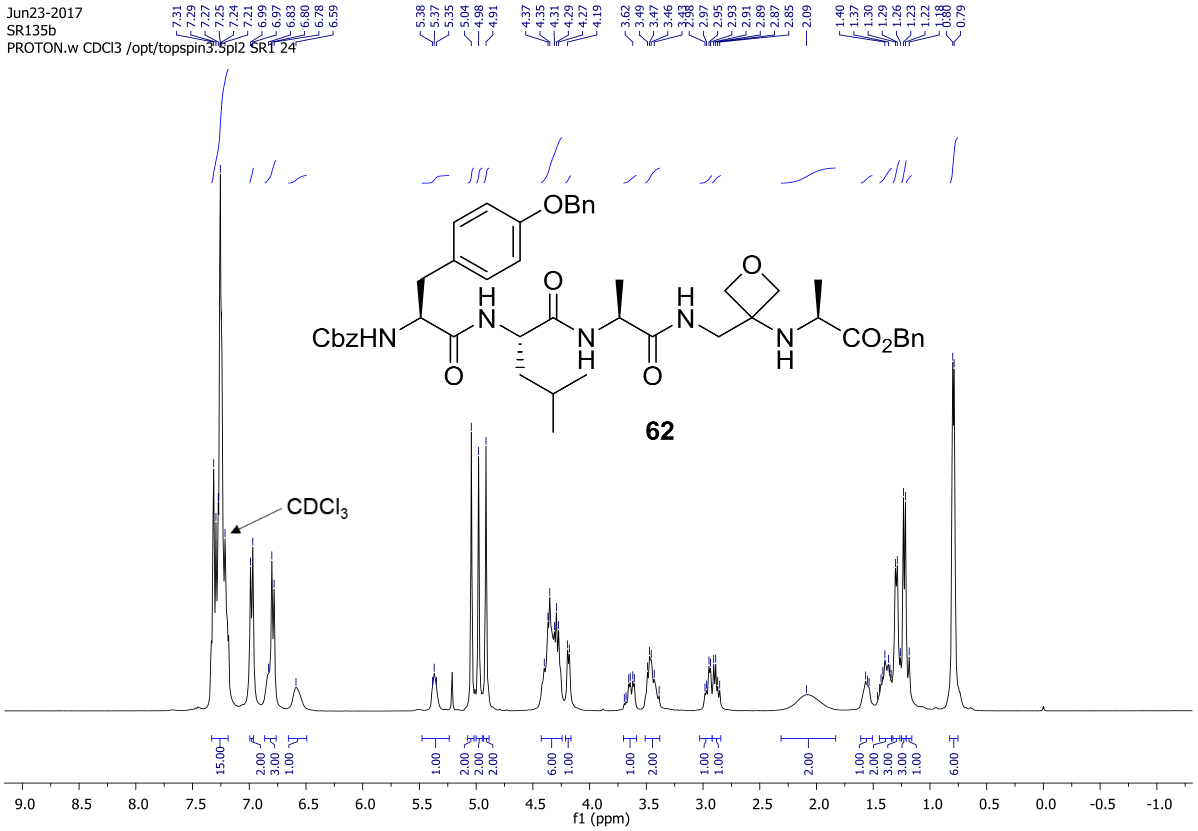

^{13}C NMR (126 MHz, $CDCl_3$)

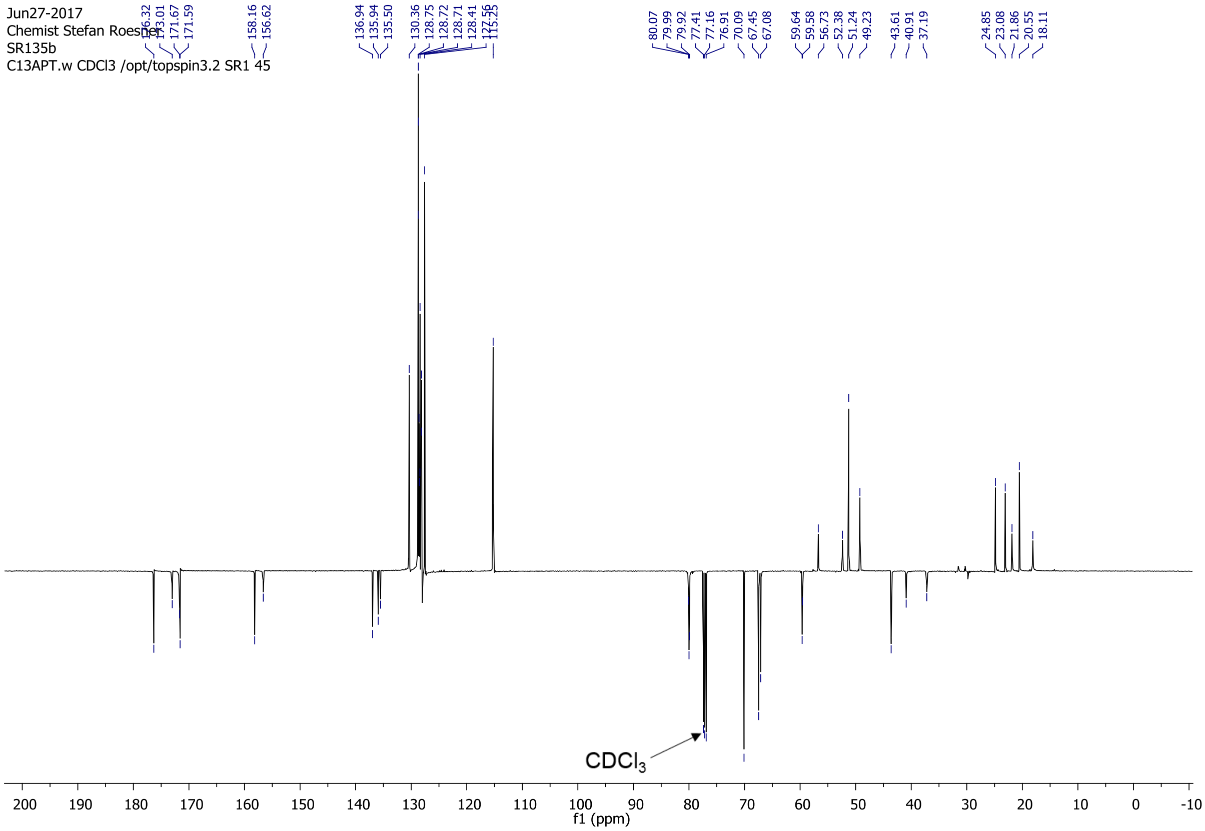

H-Tyr-Leu-Ala-GOx-Ala-OH (63)

^{1}H NMR (400 MHz, CD_3OD)

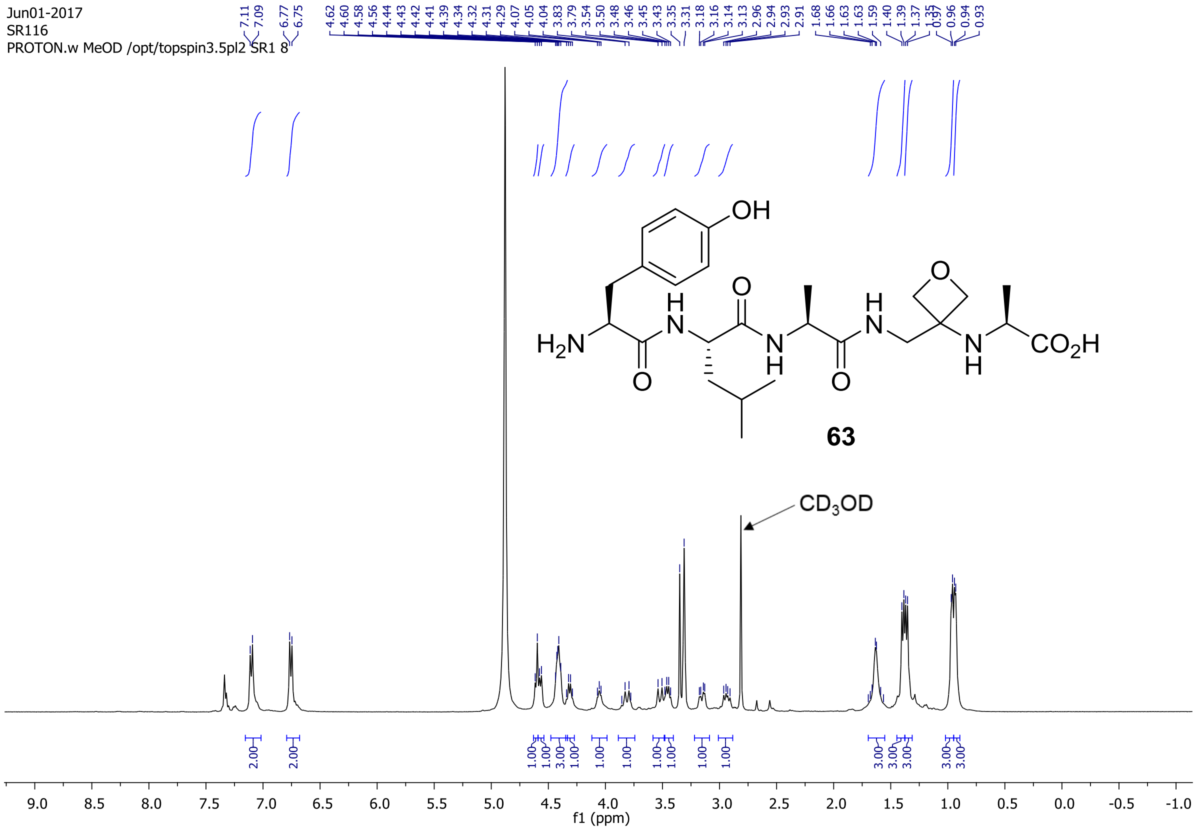

^{13}C NMR (101 MHz, CD_3OD)

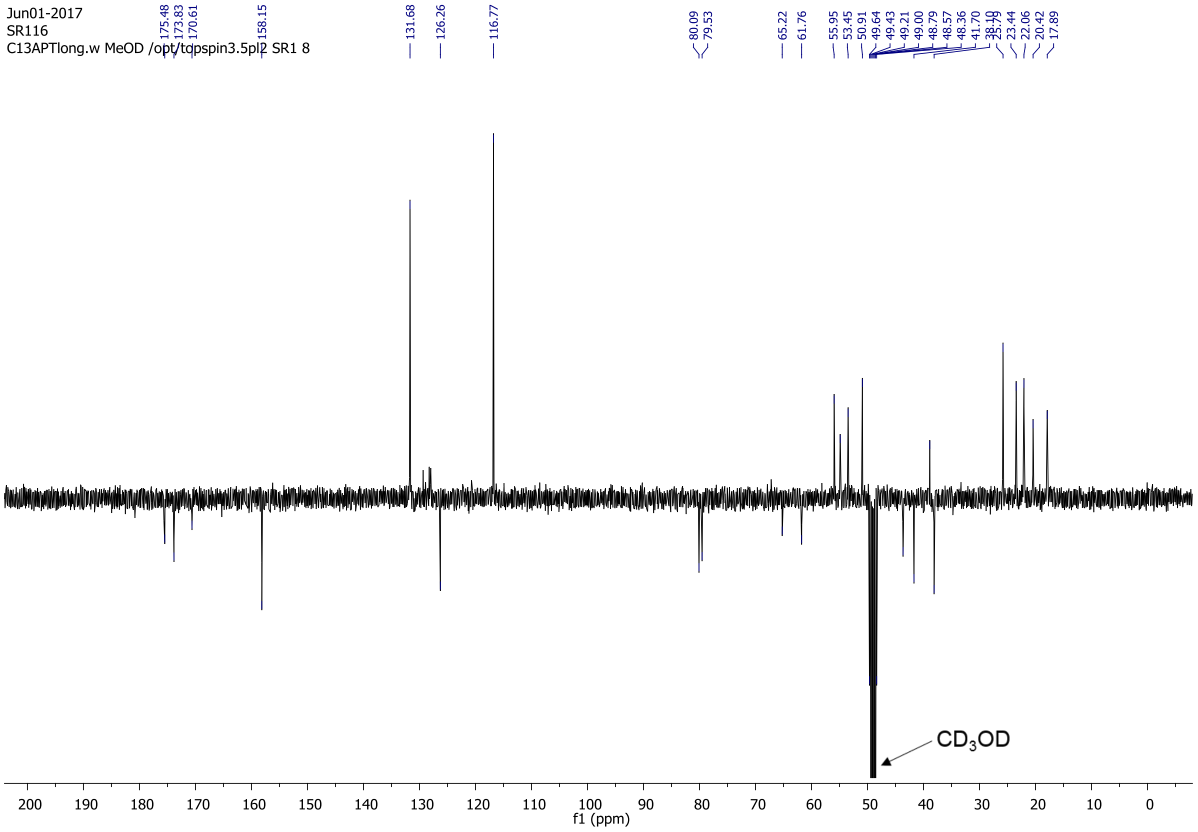

Boc-Tyr(Bn)-Leu-OBn (64)

^{1}H NMR (400 MHz, $CDCl_3$)

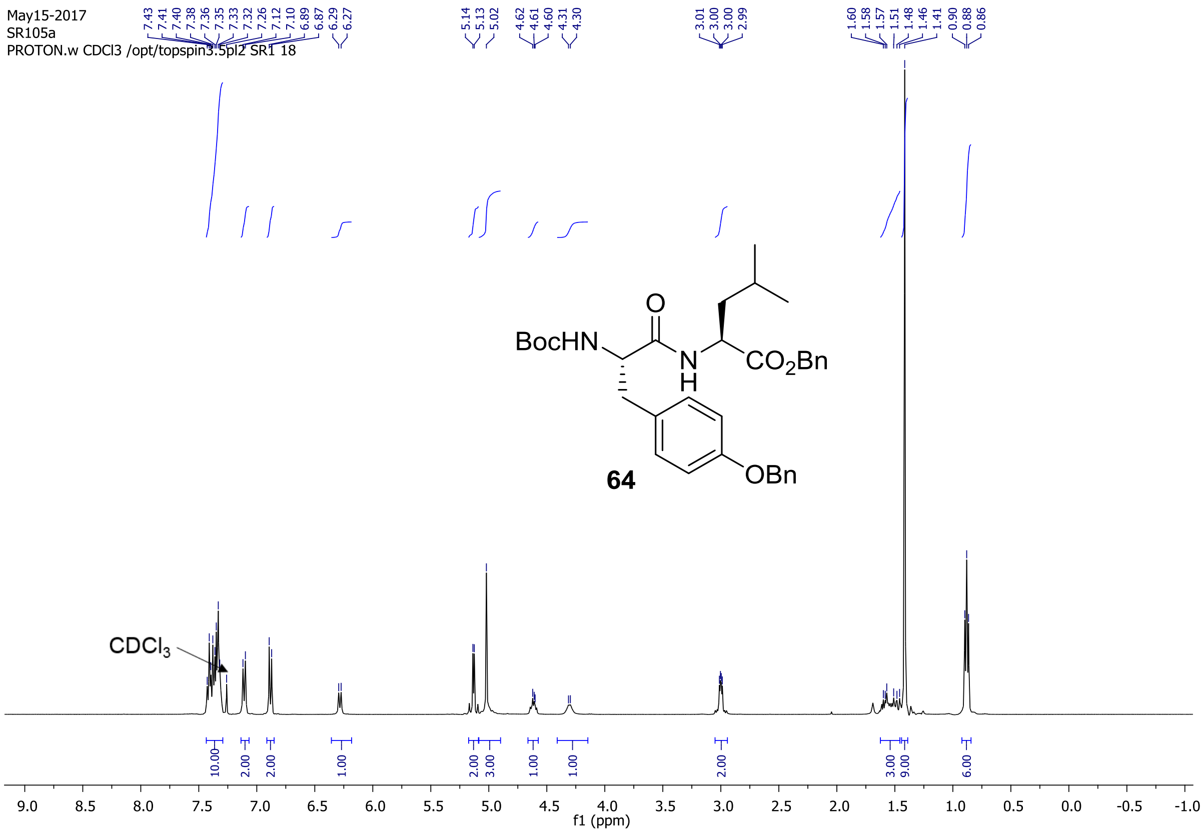

^{13}C NMR (101 MHz, $CDCl_3$)

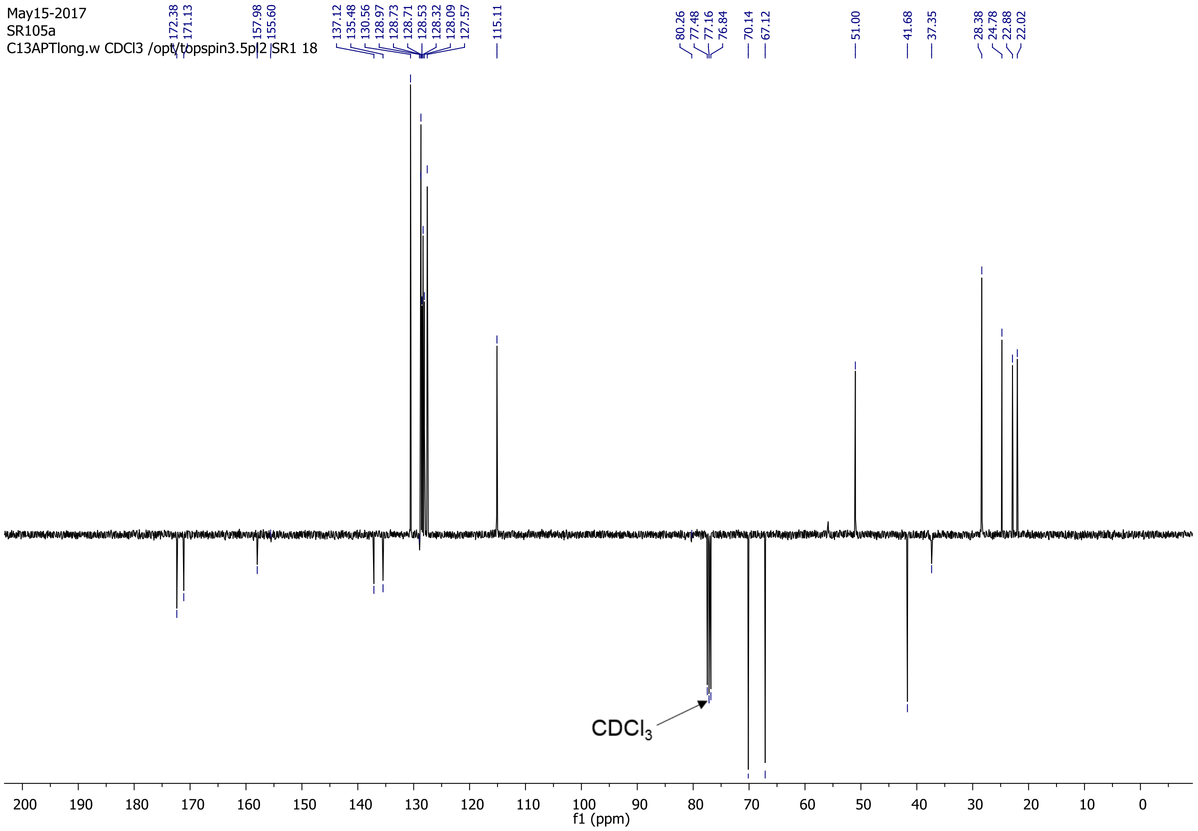

Boc-Ala-Tyr(Bn)-Leu-OBn (65)

^{1}H NMR (400 MHz, CDCl$_3$)

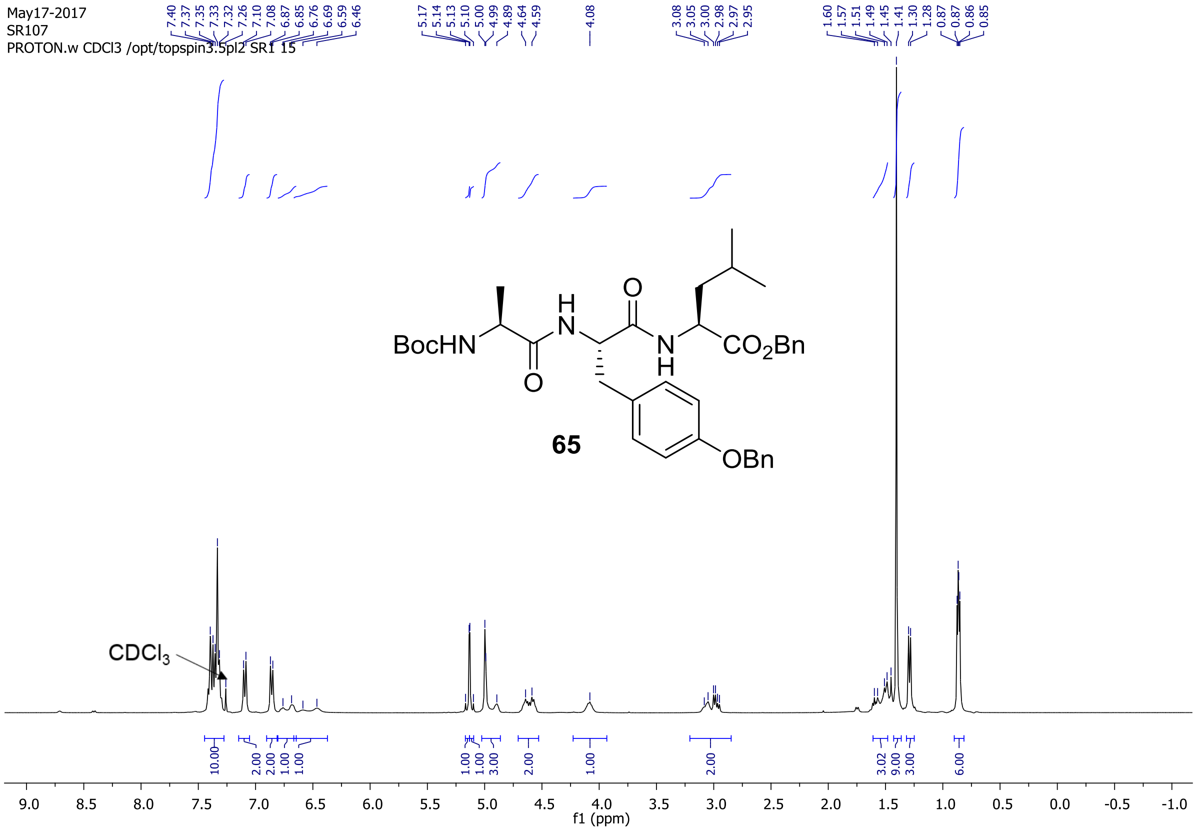

^{13}C NMR (101 MHz, CDCl$_3$)

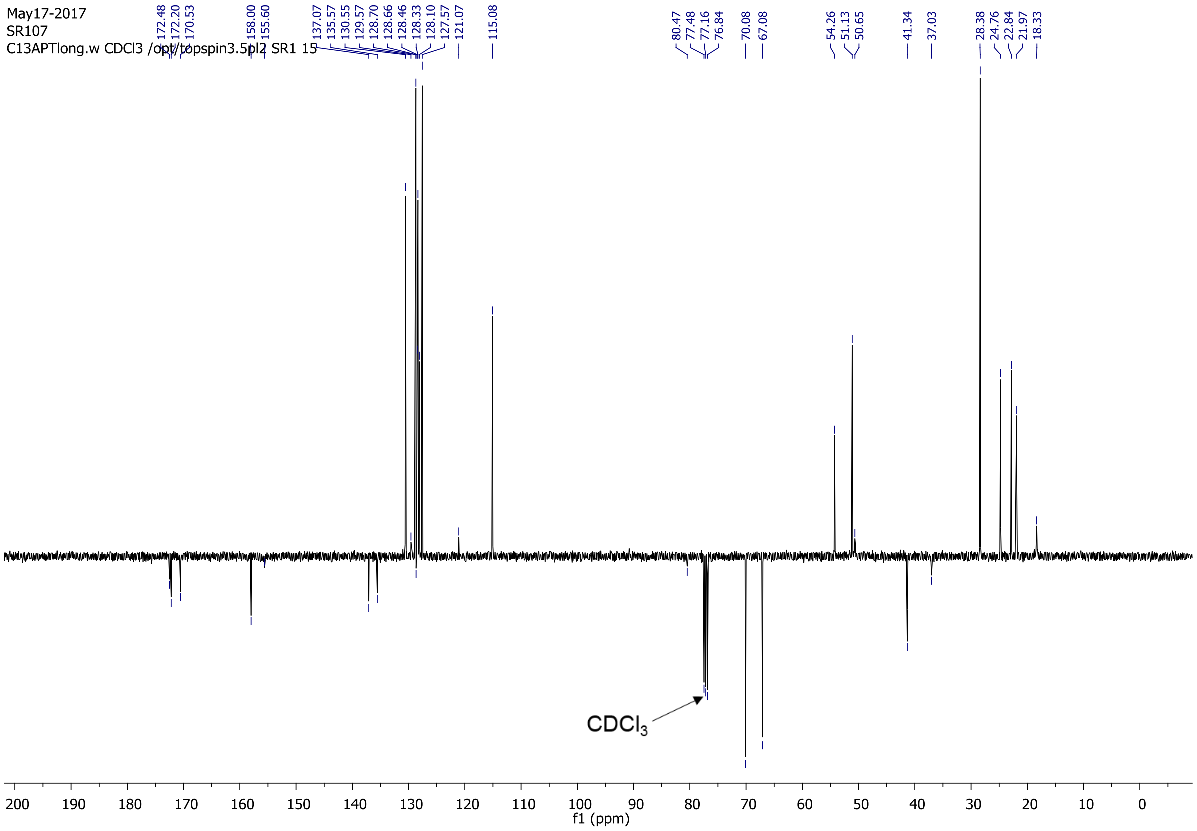

NO_2-GOx-Ala-Tyr(Bn)-Leu-OBn (66)

^{1}H NMR (400 MHz, $CDCl_3$)

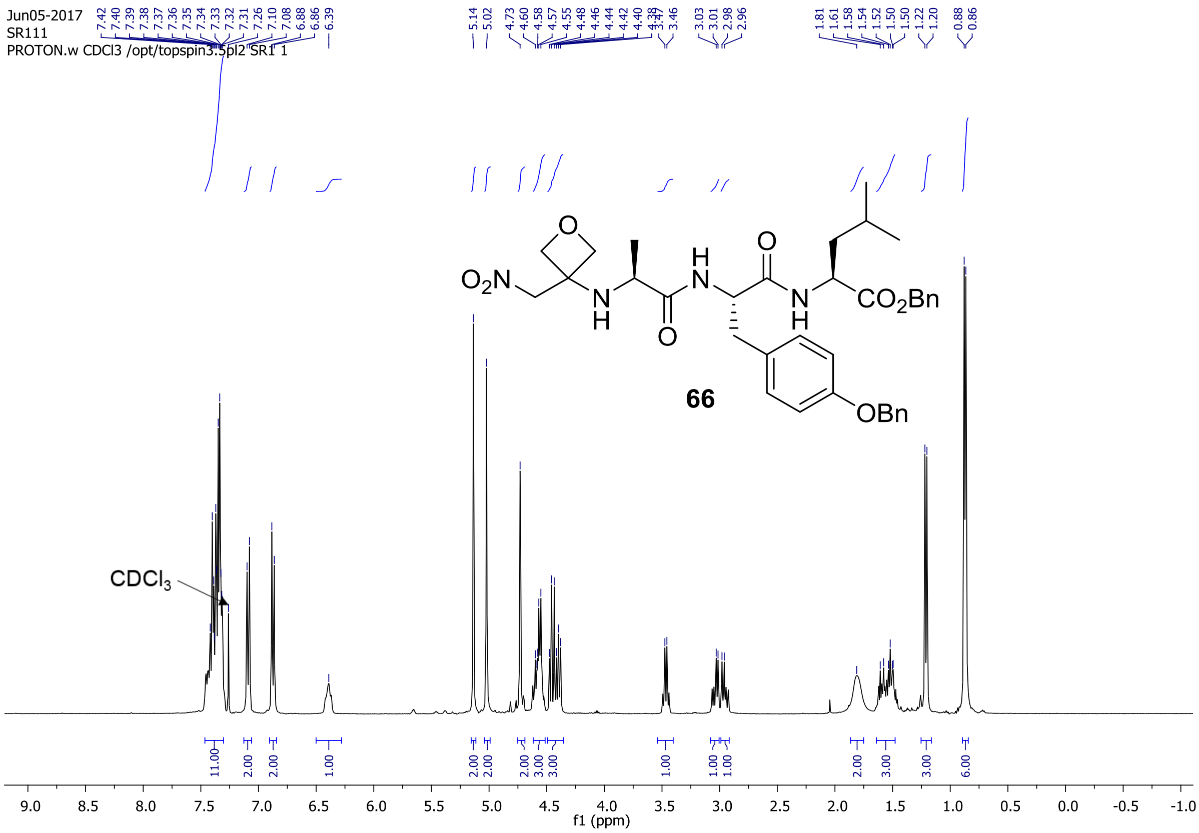

^{13}C NMR (101 MHz, $CDCl_3$)

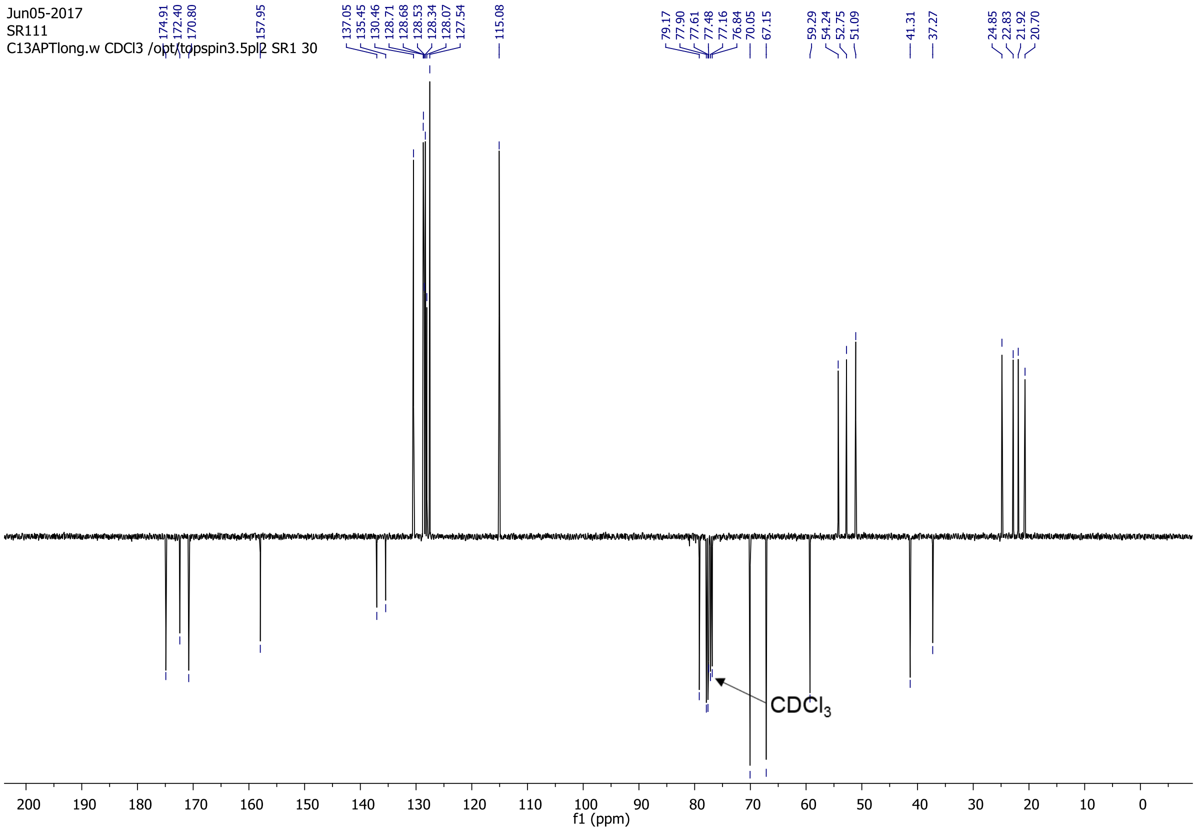

Cbz-Ala-GOx-Ala-Tyr(Bn)-Leu-OBn (67)

^{1}H NMR (400 MHz, $CDCl_3$)

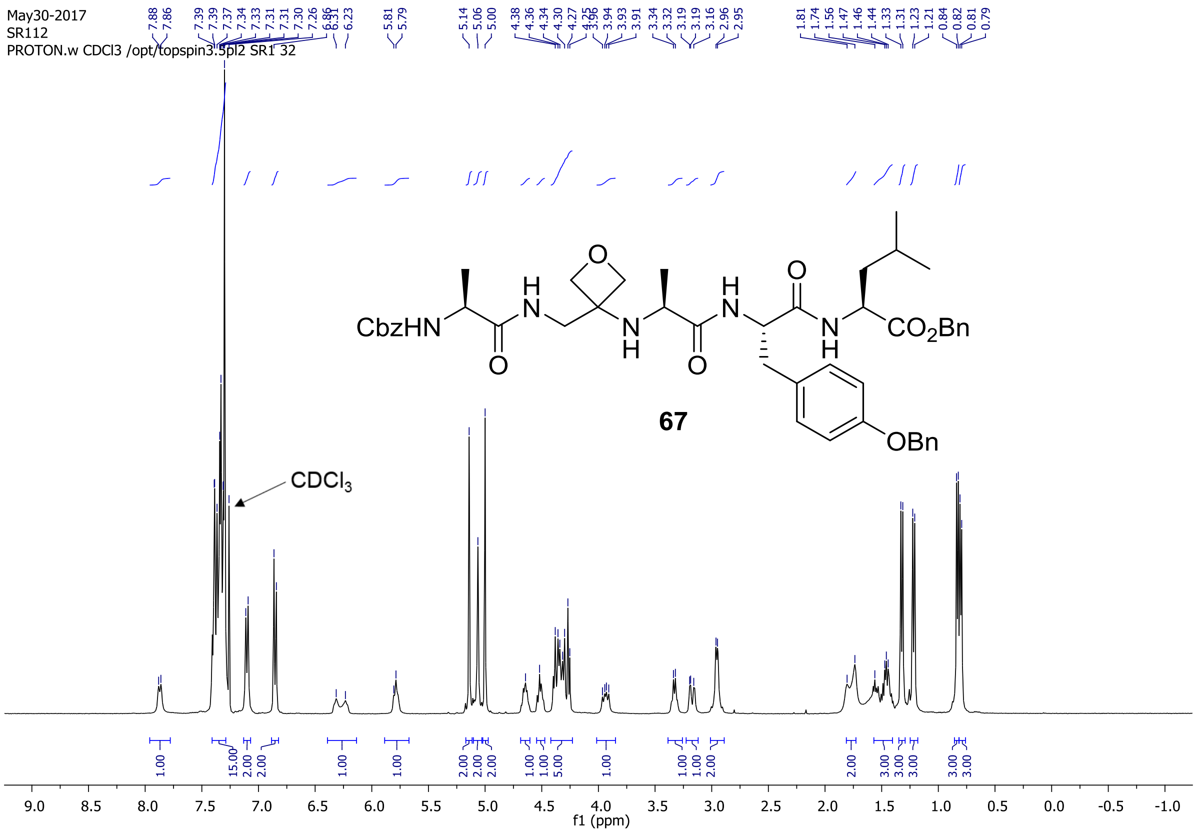

^{13}C NMR (126 MHz, $CDCl_3$)

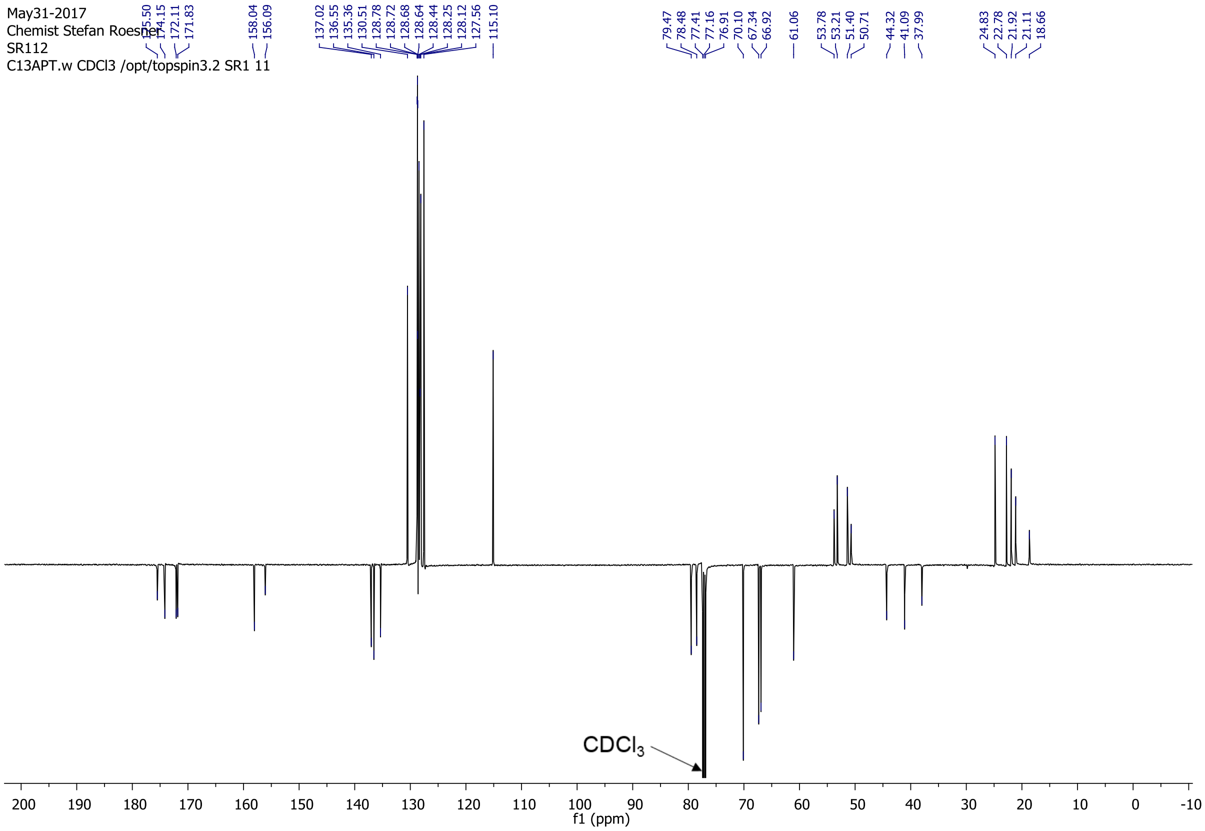

H-Ala-GOx-Ala-Tyr-Leu-OH (68)

^{1}H NMR (400 MHz, CD_3OD)

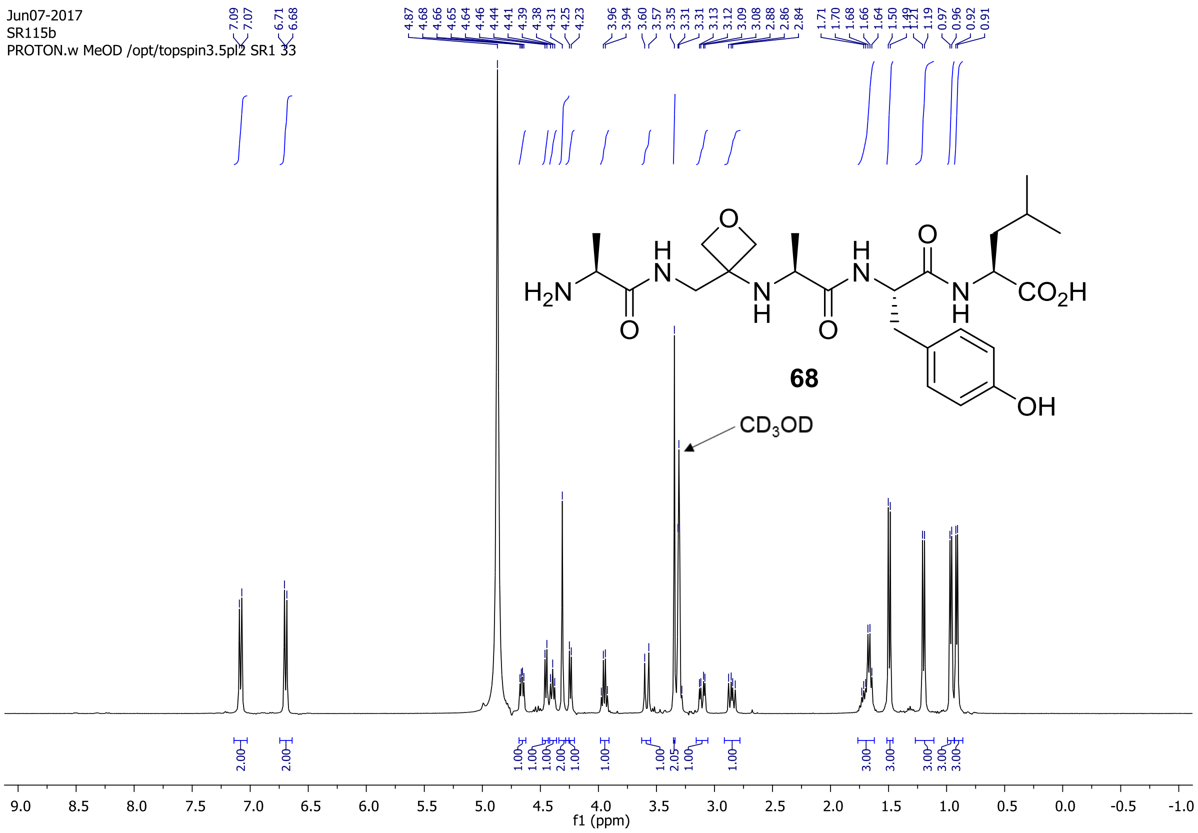

^{13}C NMR (126 MHz, CD_3OD)

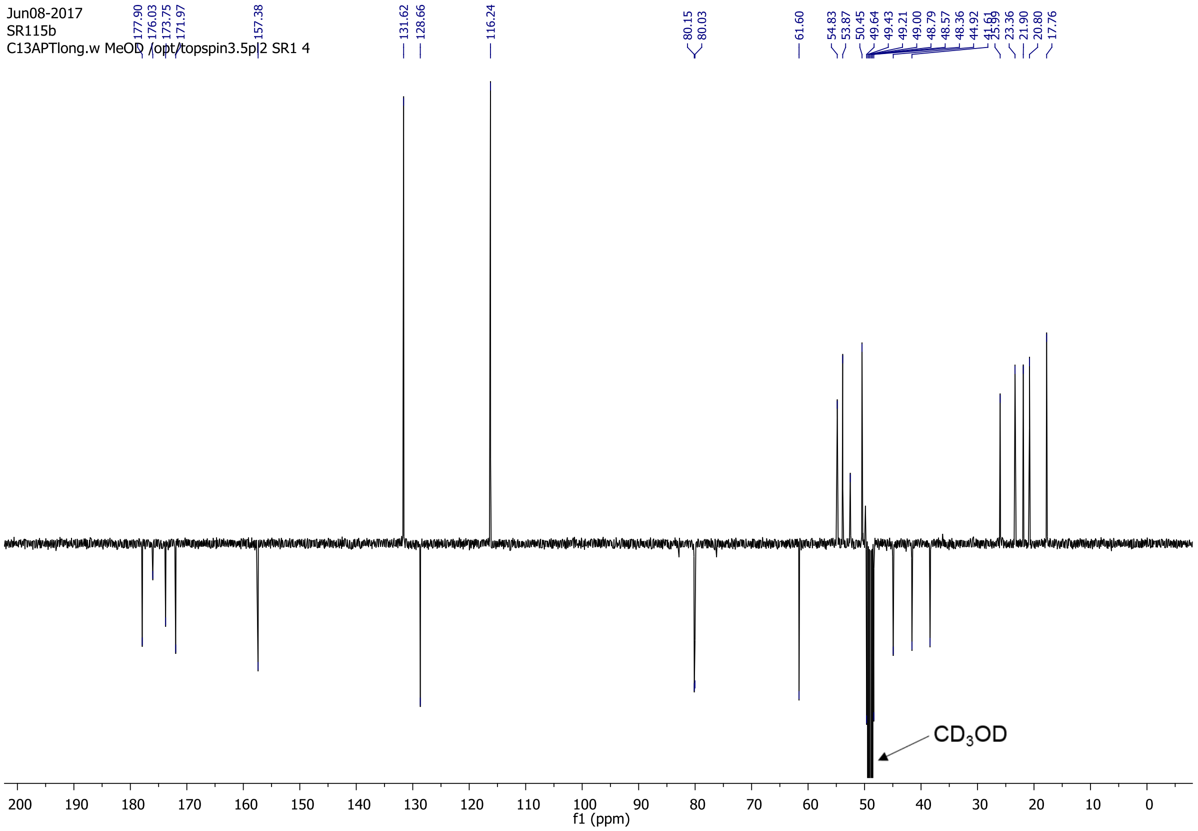

Boc-Ala-Tyr(Bn)-OBn (69)

^{1}H NMR (400 MHz, $CDCl_3$)

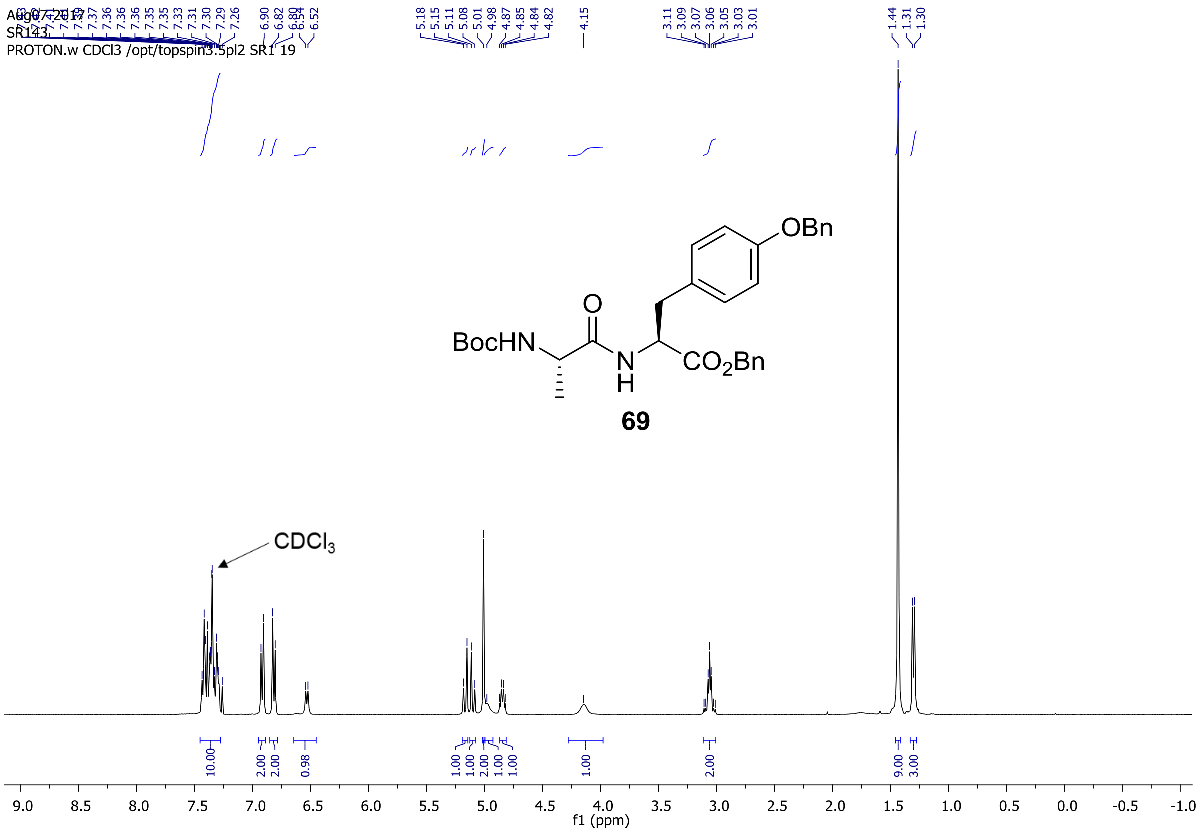

^{13}C NMR (101 MHz, $CDCl_3$)

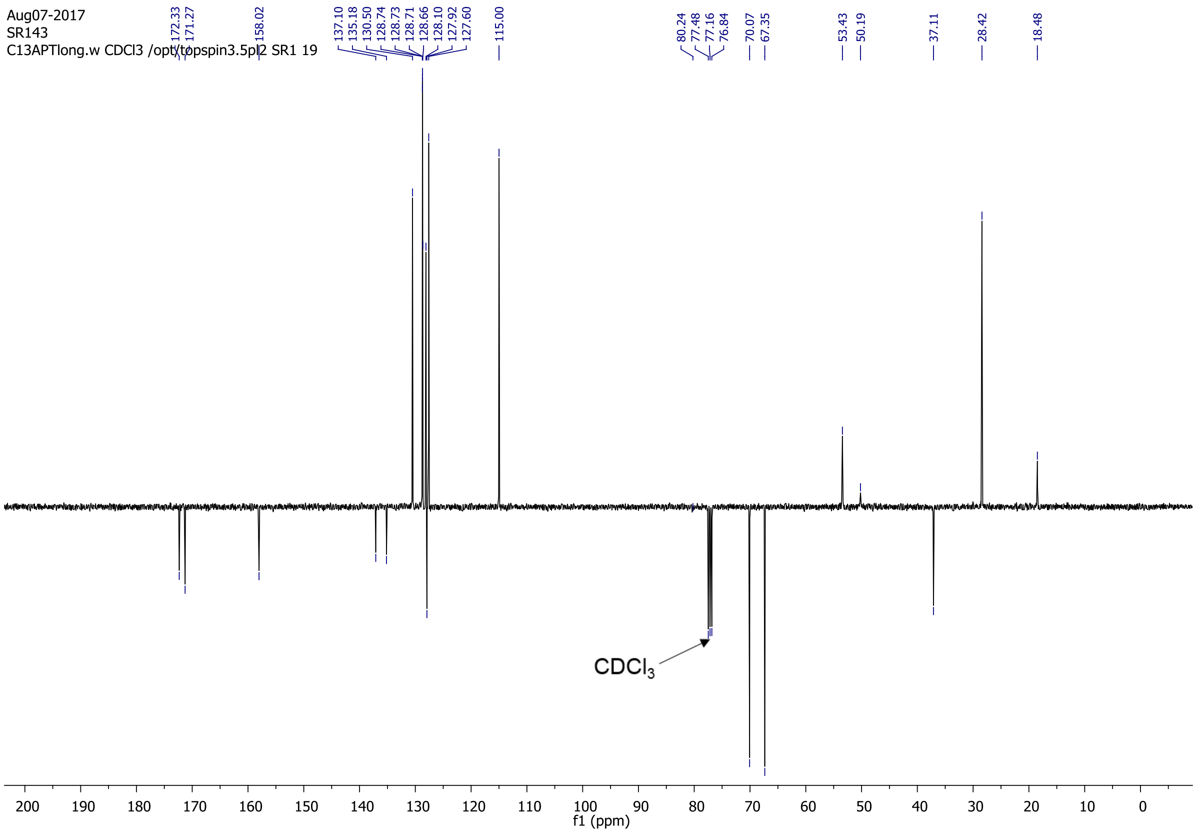

O_2N-GOx-Ala-Tyr(Bn)-OBn (70)

^{1}H NMR (400 MHz, $CDCl_3$)

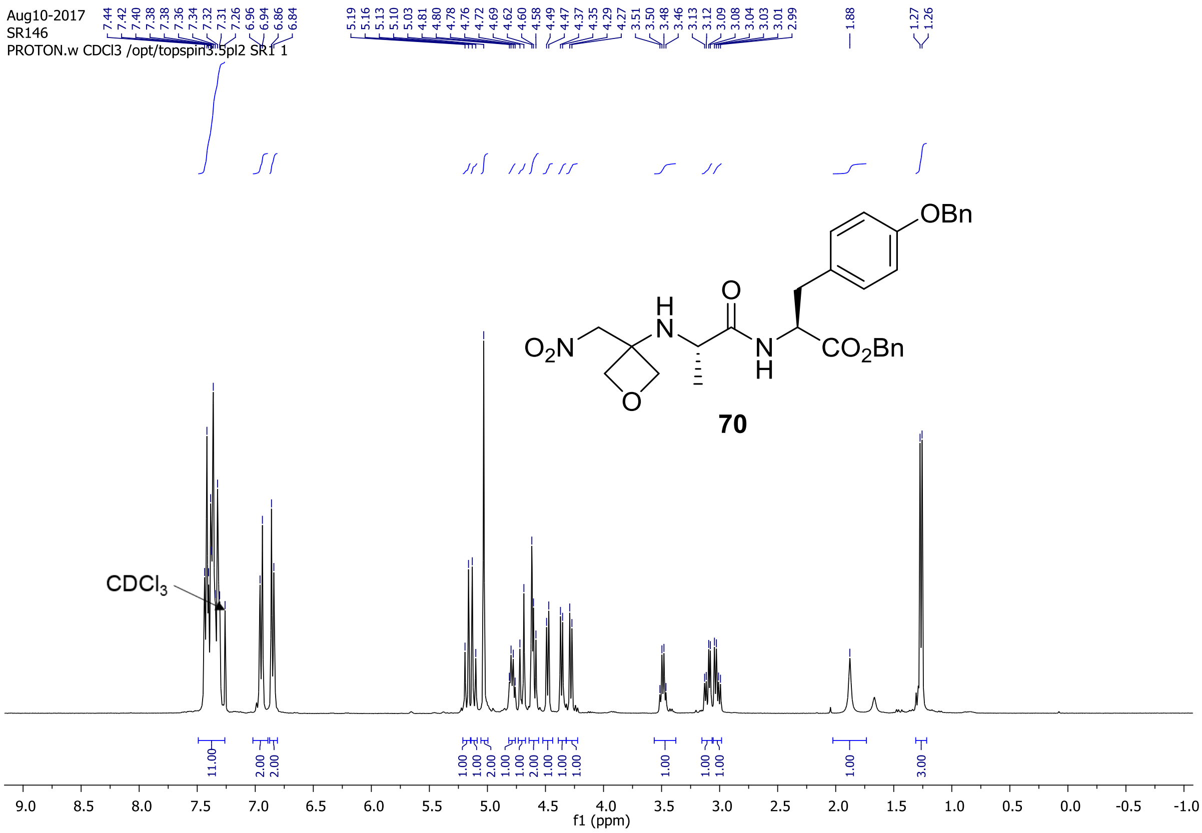

^{13}C NMR (101 MHz, $CDCl_3$)

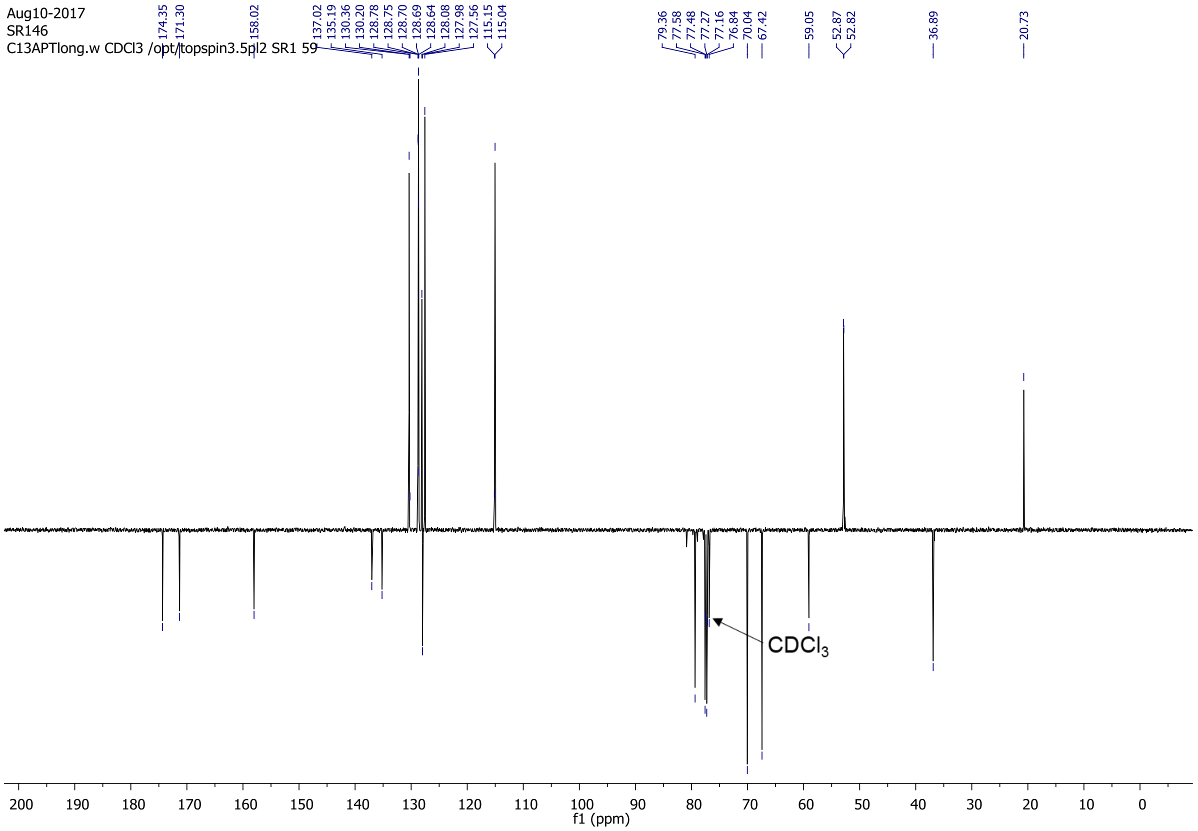

Boc-Ala-GOx-Ala-Tyr(Bn)-OBn (71)

^{1}H NMR (400 MHz, $CDCl_3$)

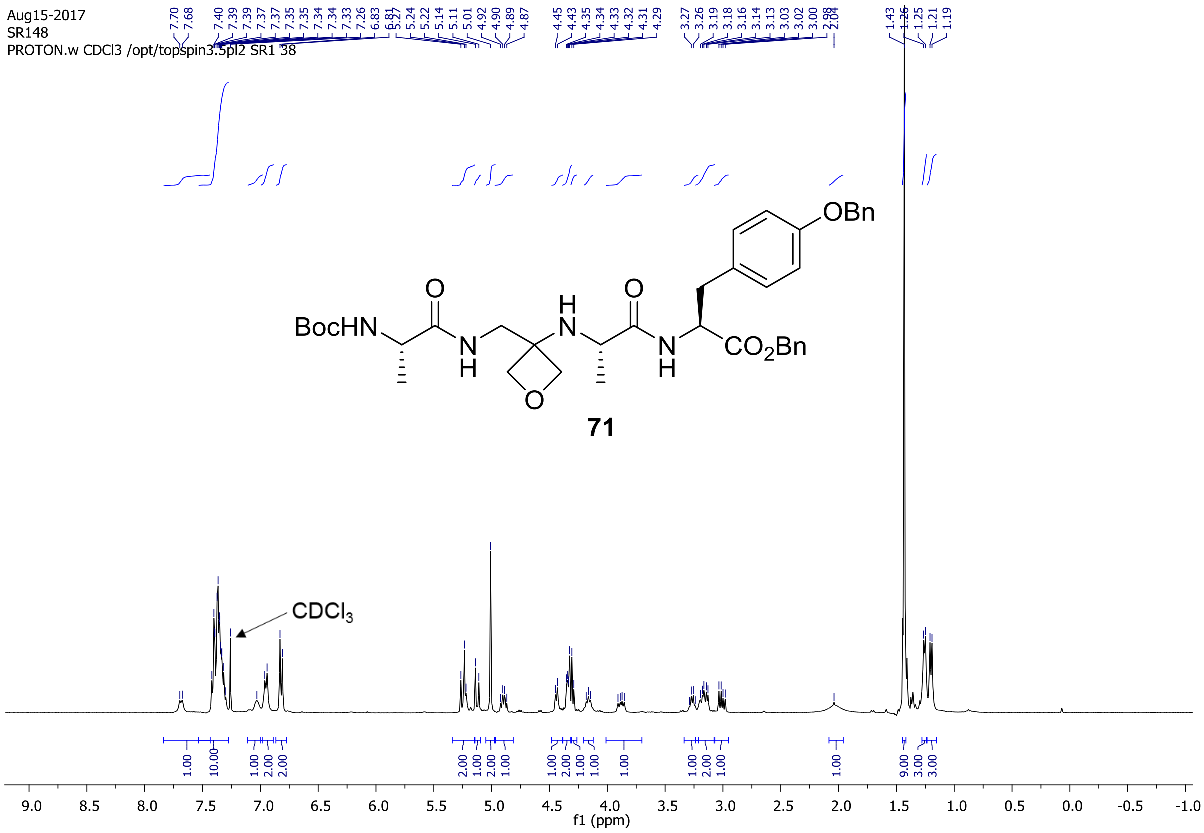

^{13}C NMR (101 MHz, $CDCl_3$)

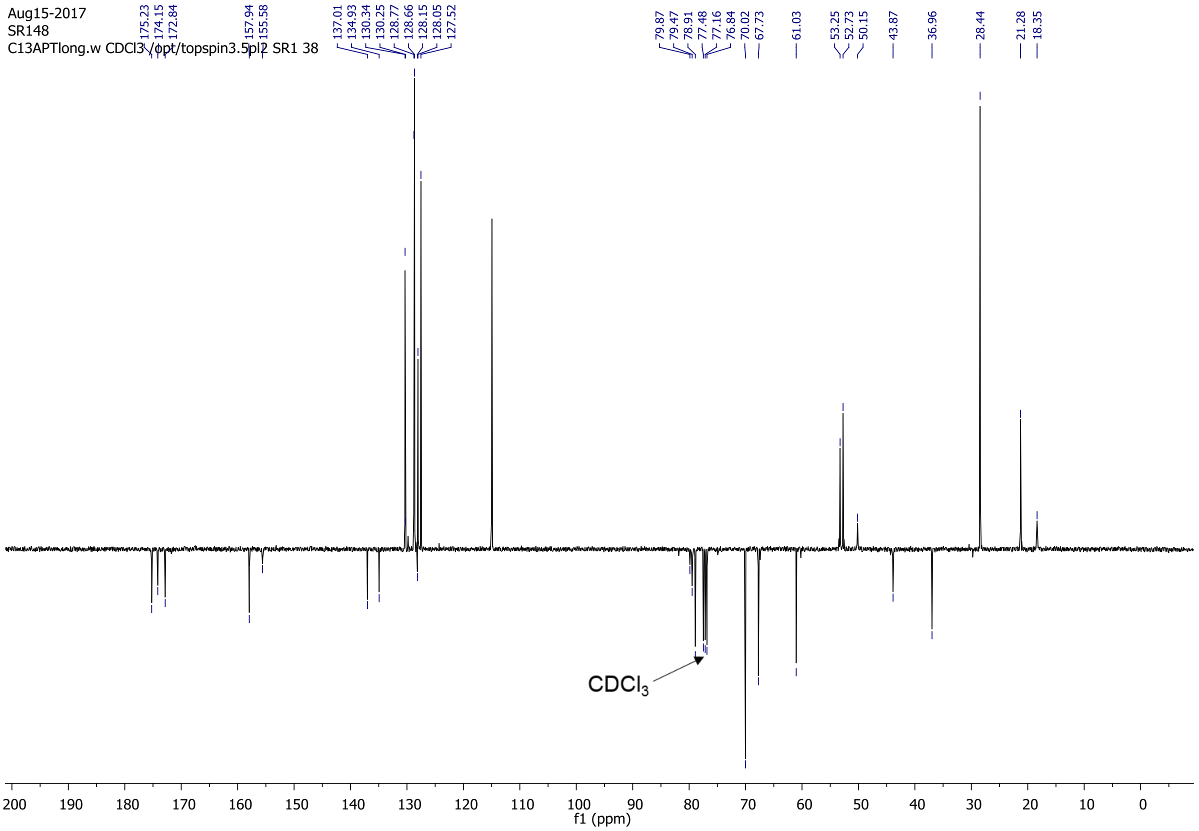

Cbz-Leu-Ala-GOx-Ala-Tyr(Bn)-OBn (72)

^{1}H NMR (400 MHz, CD_3OD)

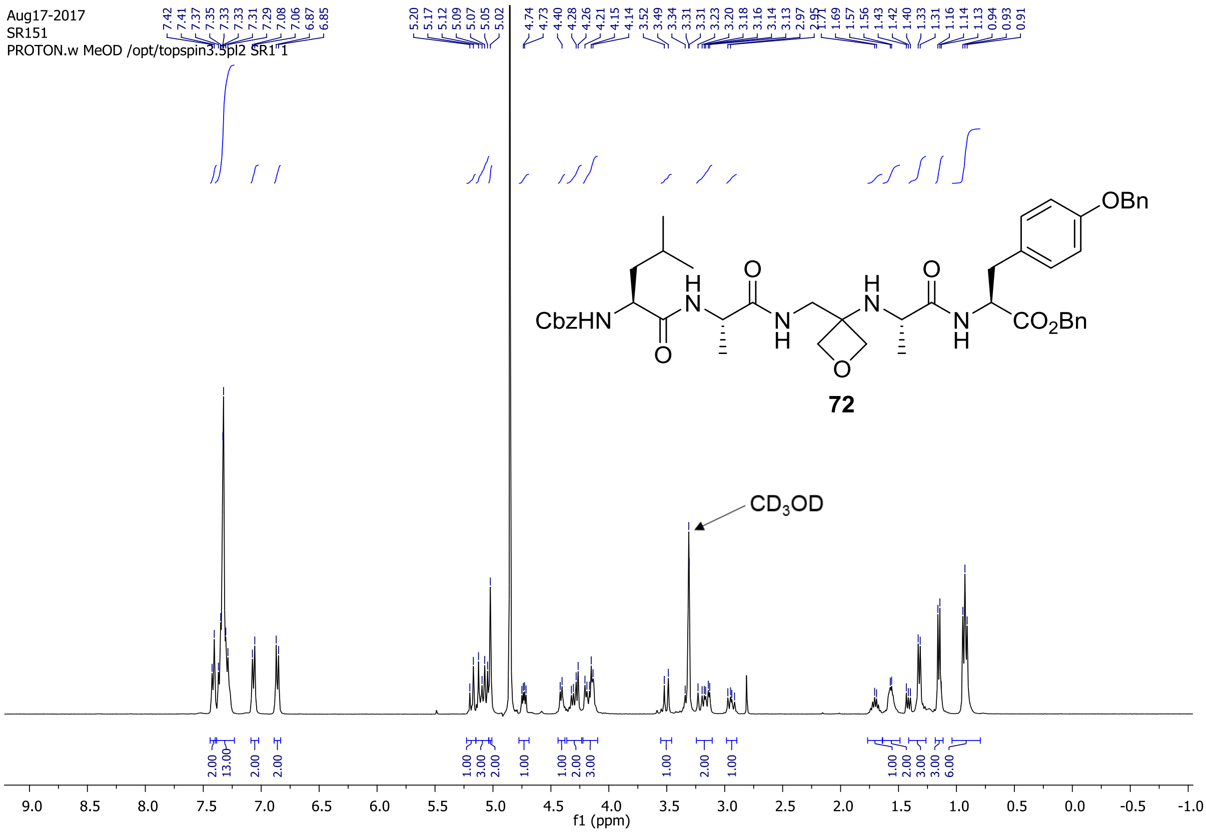

^{13}C NMR (101 MHz, CD_3OD)

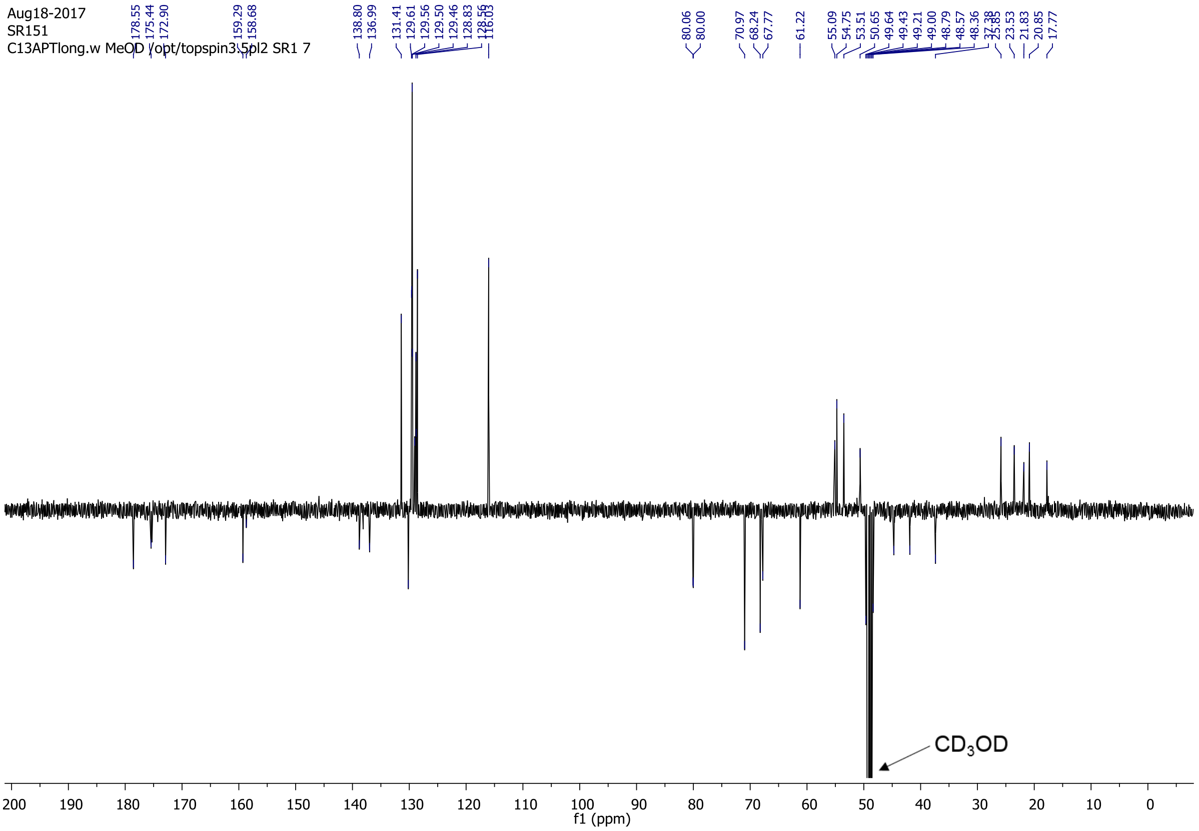

H-Leu-Ala-GOx-Ala-Tyr-OH (73)

^{1}H NMR (400 MHz, CD_3OD)

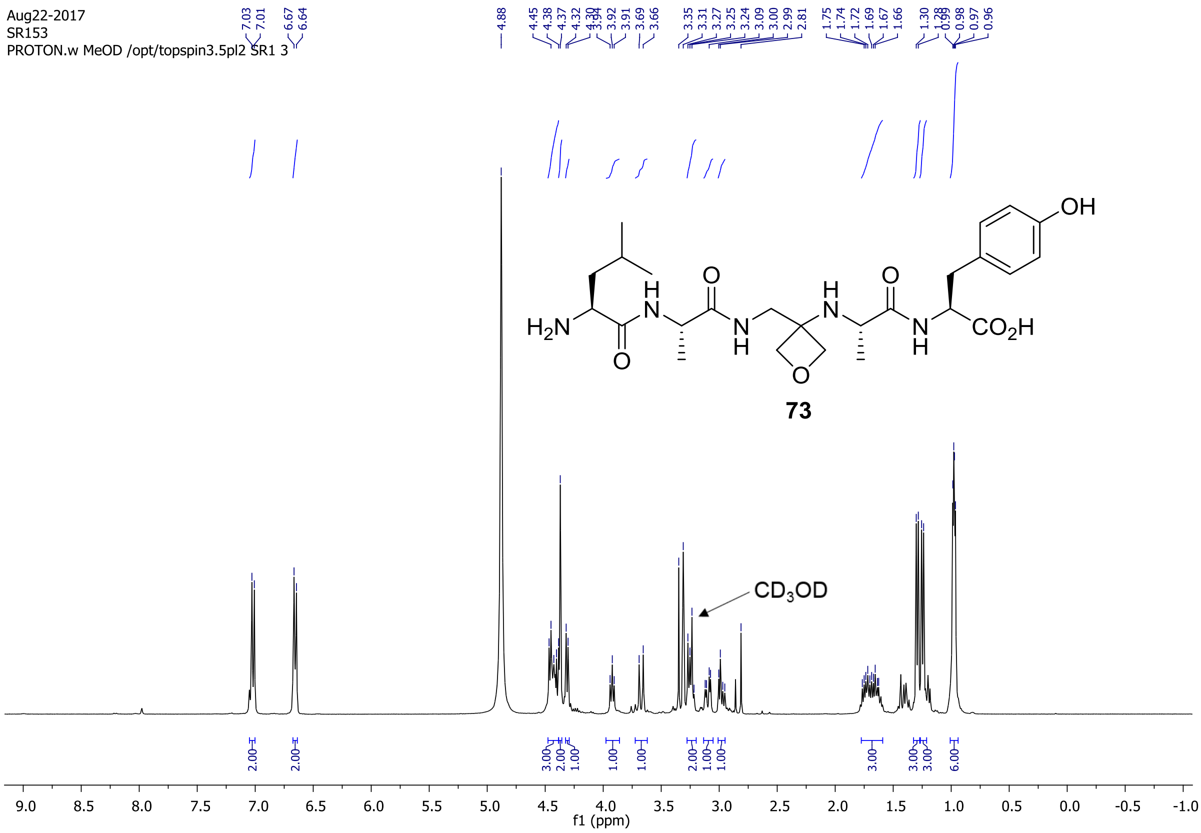

^{13}C NMR (101 MHz, CD_3OD)

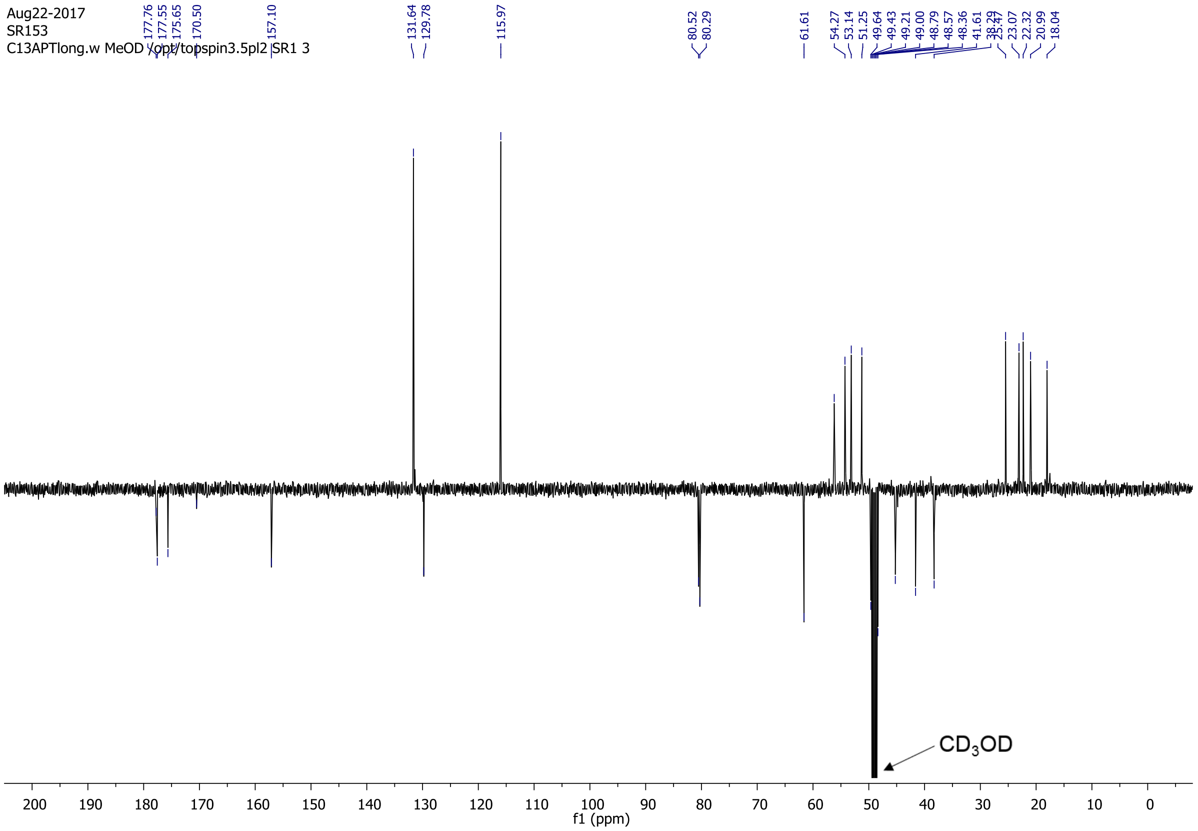

Fmoc-Ala-OCumyl (74)

^{1}H NMR (400 MHz, $CDCl_3$)

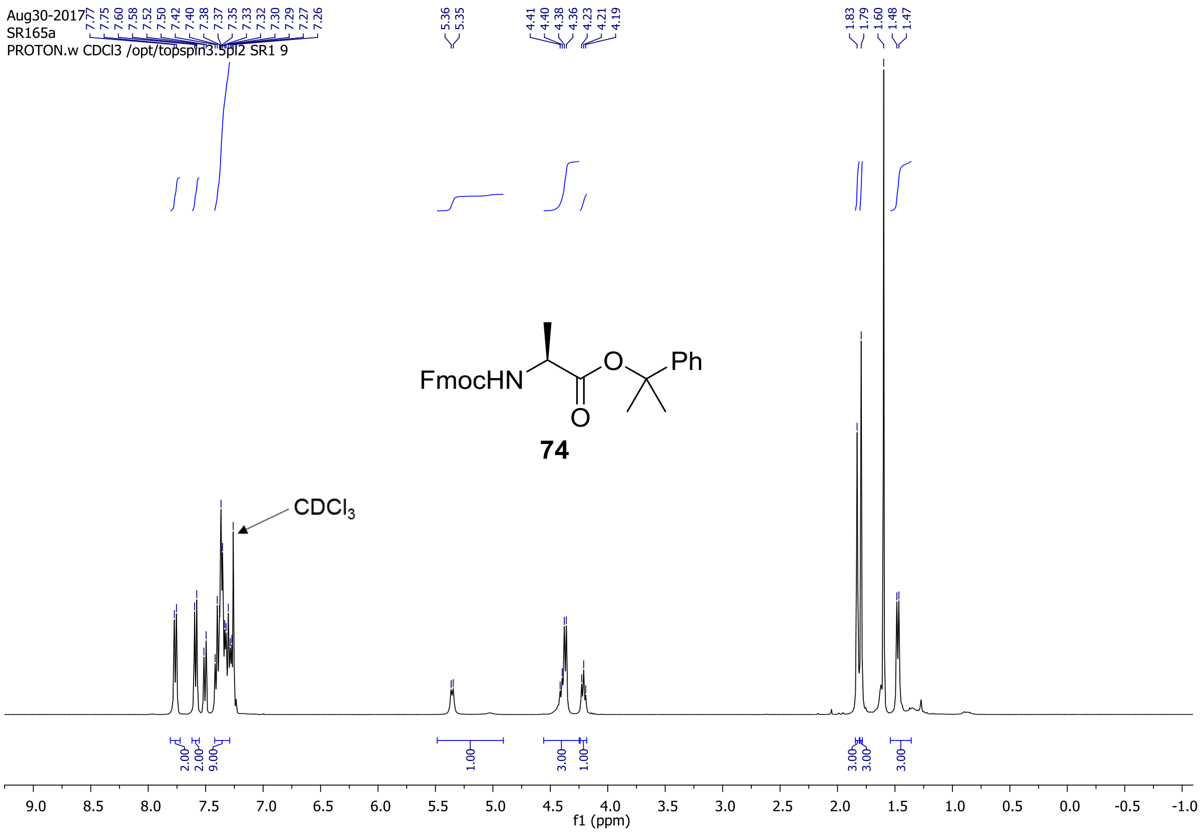

^{13}C NMR (101 MHz, $CDCl_3$)

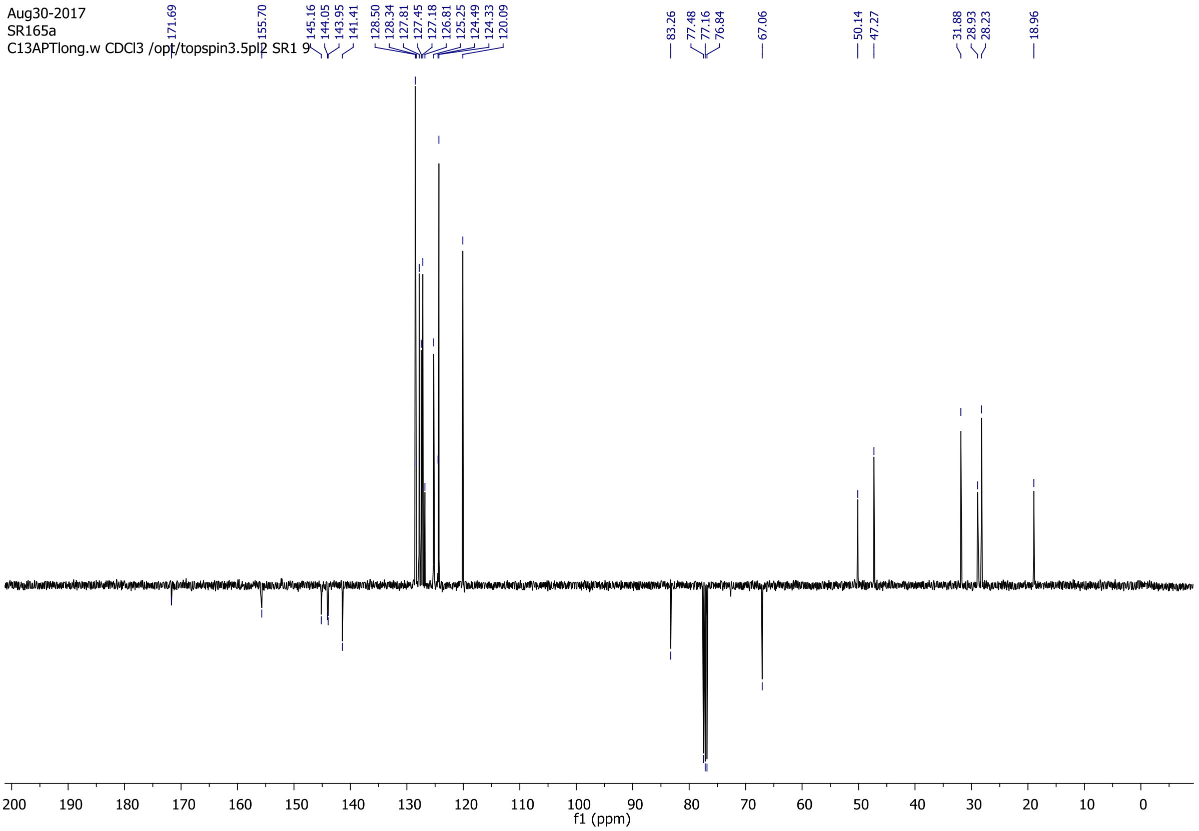

O_2N-GOx-Ala-OCumyl (75)

^1H NMR (400 MHz, $CDCl_3$)

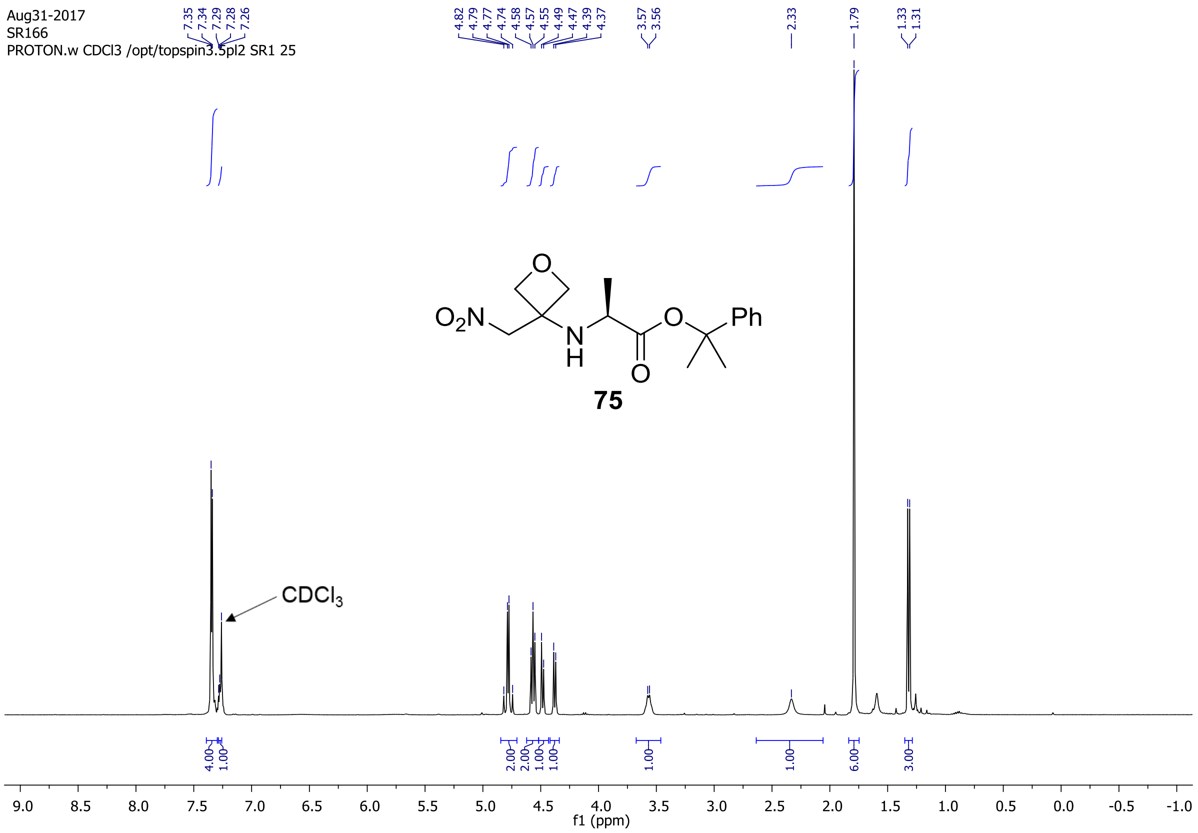

^{13}C NMR (101 MHz, $CDCl_3$)

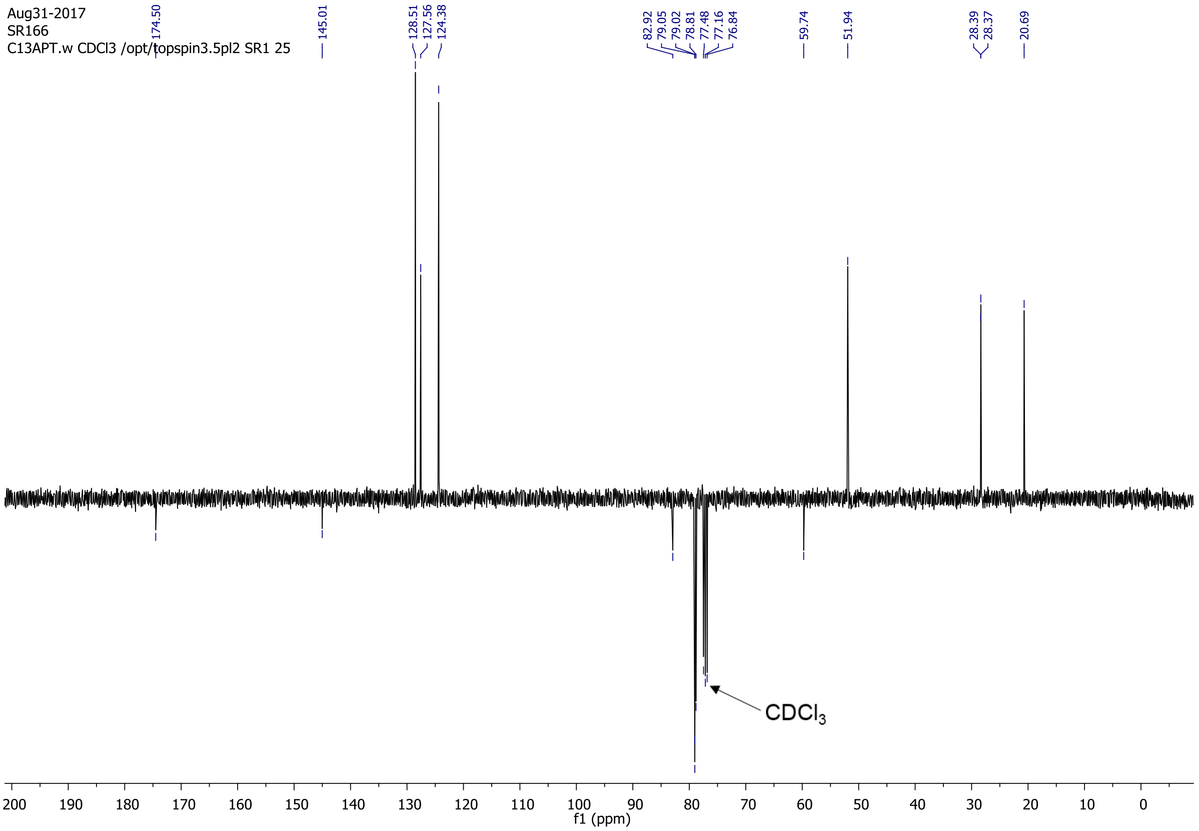

Cbz-GOx-Ala-OCumyl (76)

^{1}H NMR (400 MHz, $CDCl_3$)

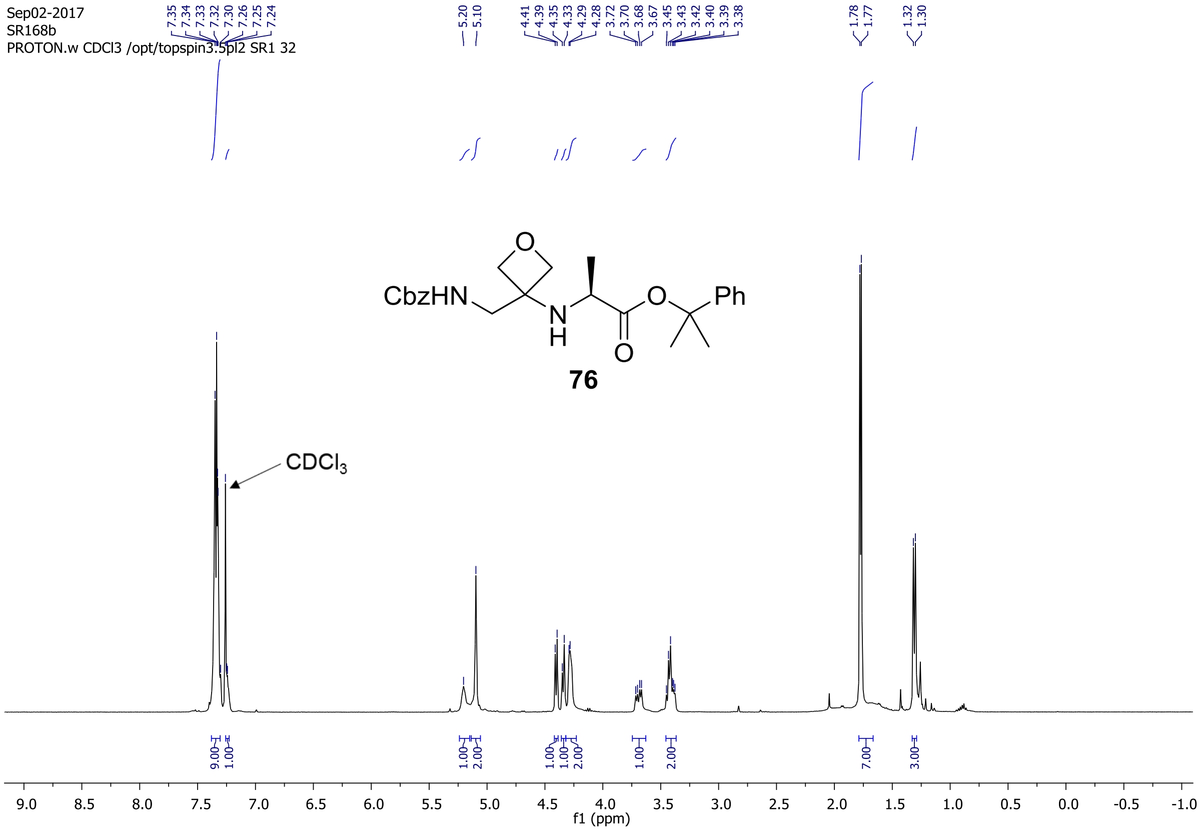

^{13}C NMR (101 MHz, $CDCl_3$)

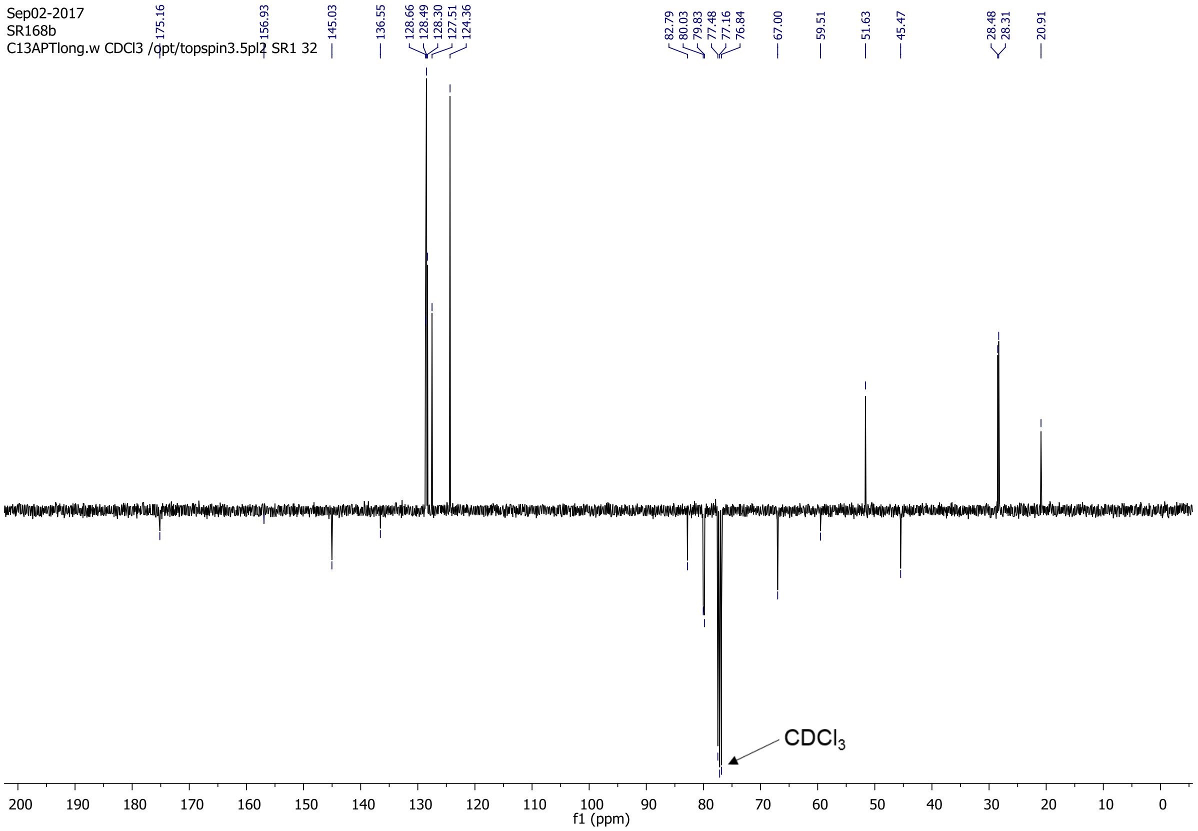

Boc-Leu-Ala-OBn (77)

^{1}H NMR (400 MHz, $CDCl_3$)

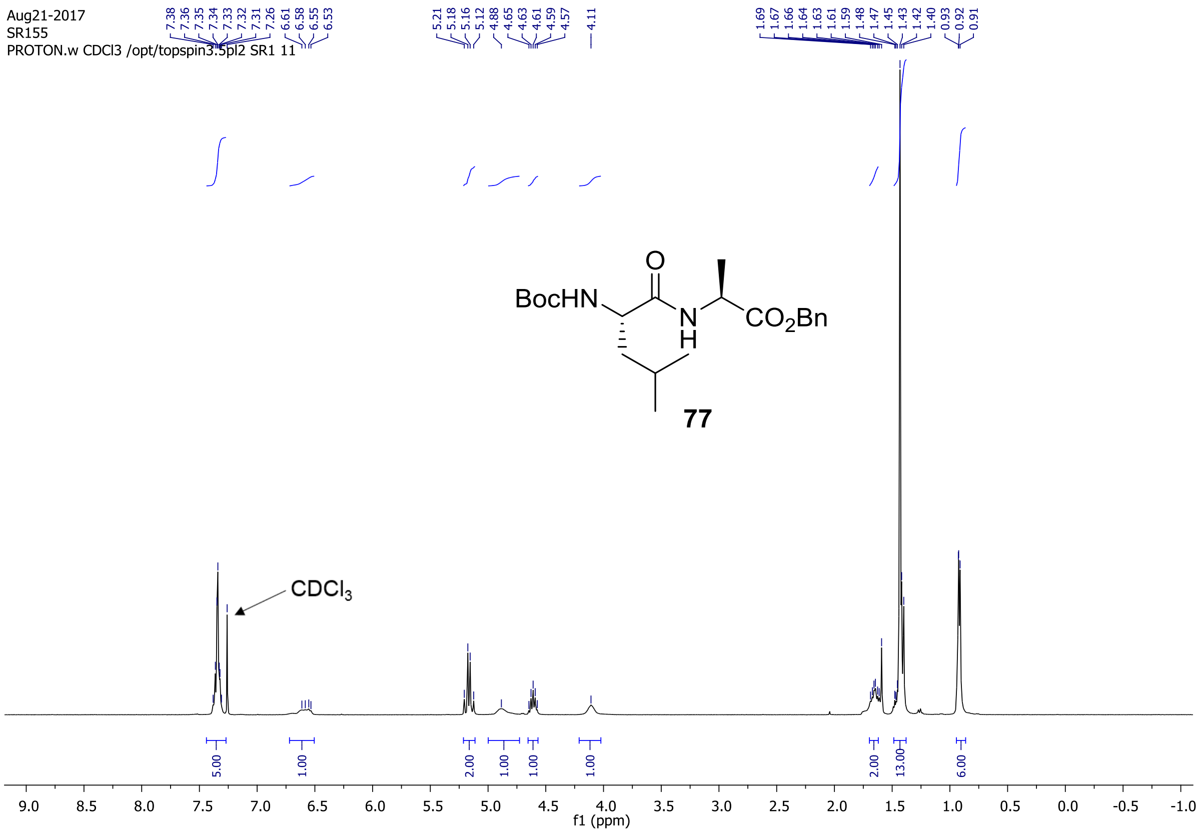

^{13}C NMR (101 MHz, $CDCl_3$)

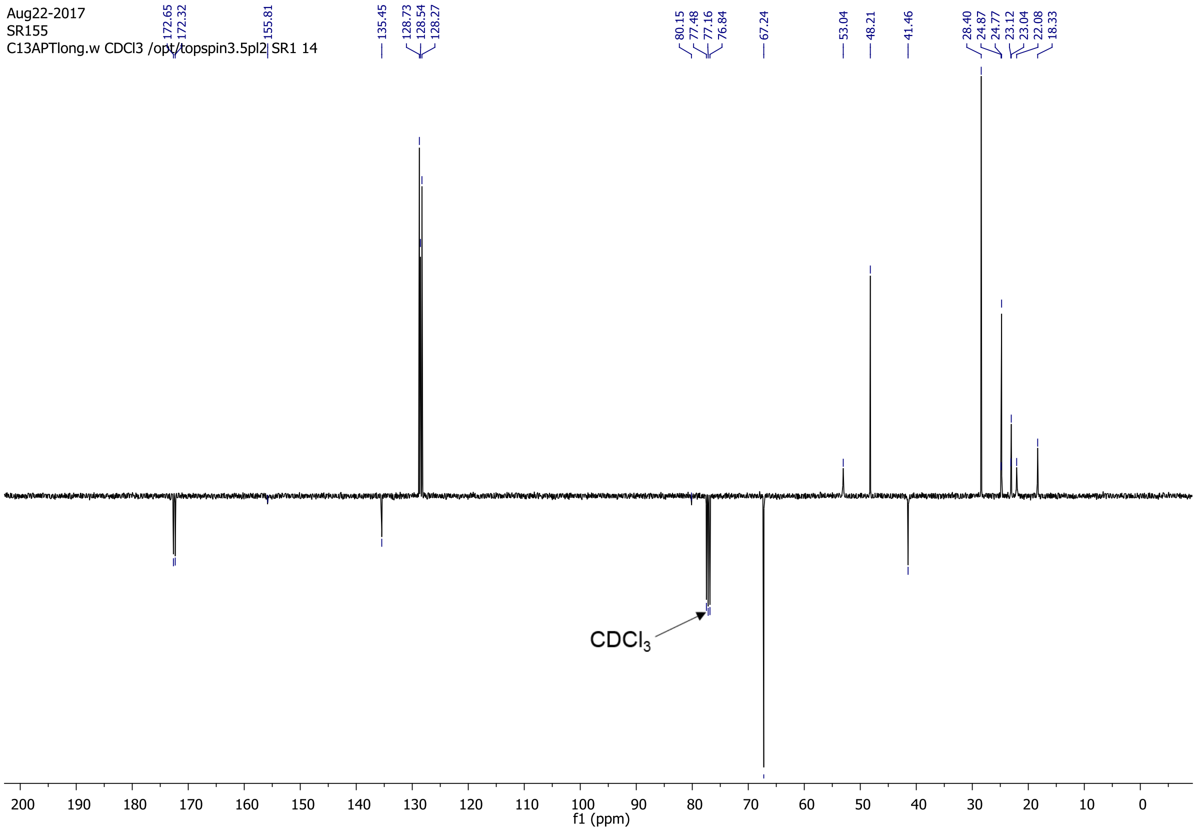

Boc-Tyr(Bn)-Leu-Ala-OBn (78)

^{1}H NMR (400 MHz, $CDCl_3$)

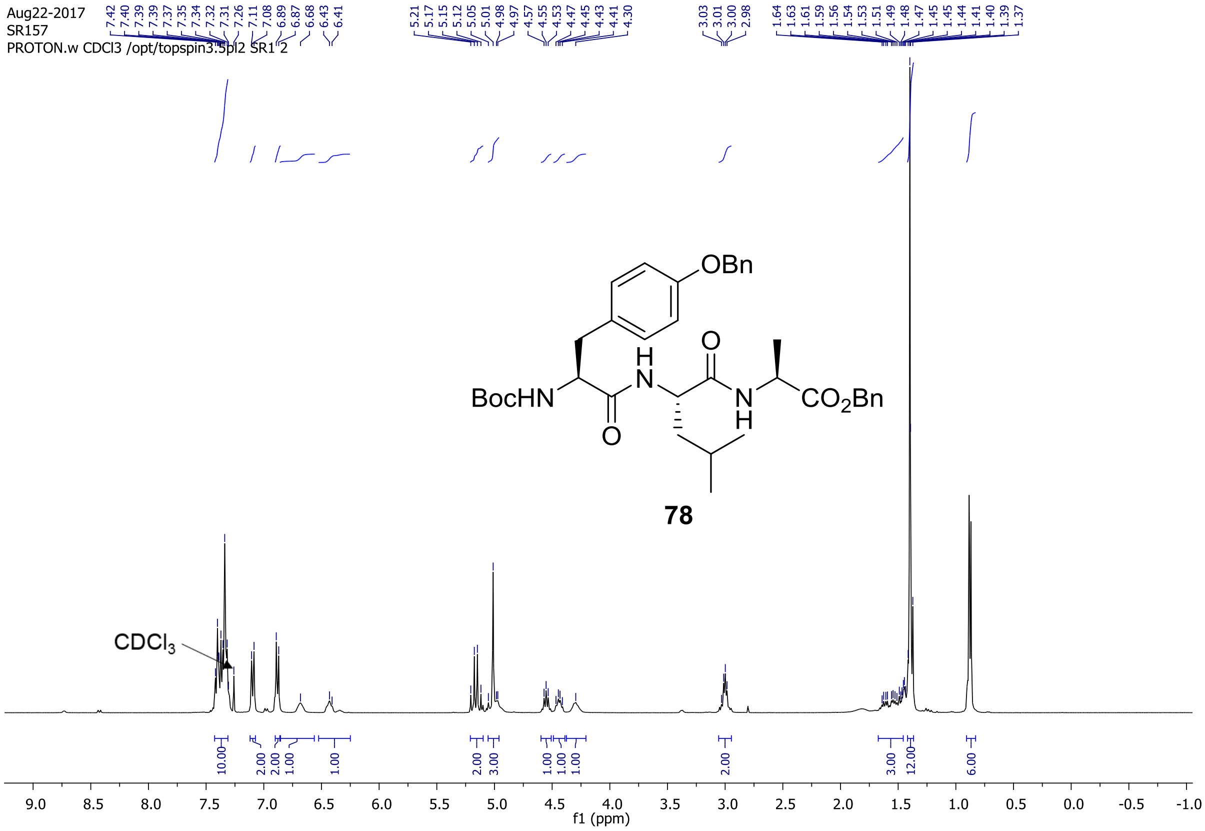

^{13}C NMR (101 MHz, $CDCl_3$)

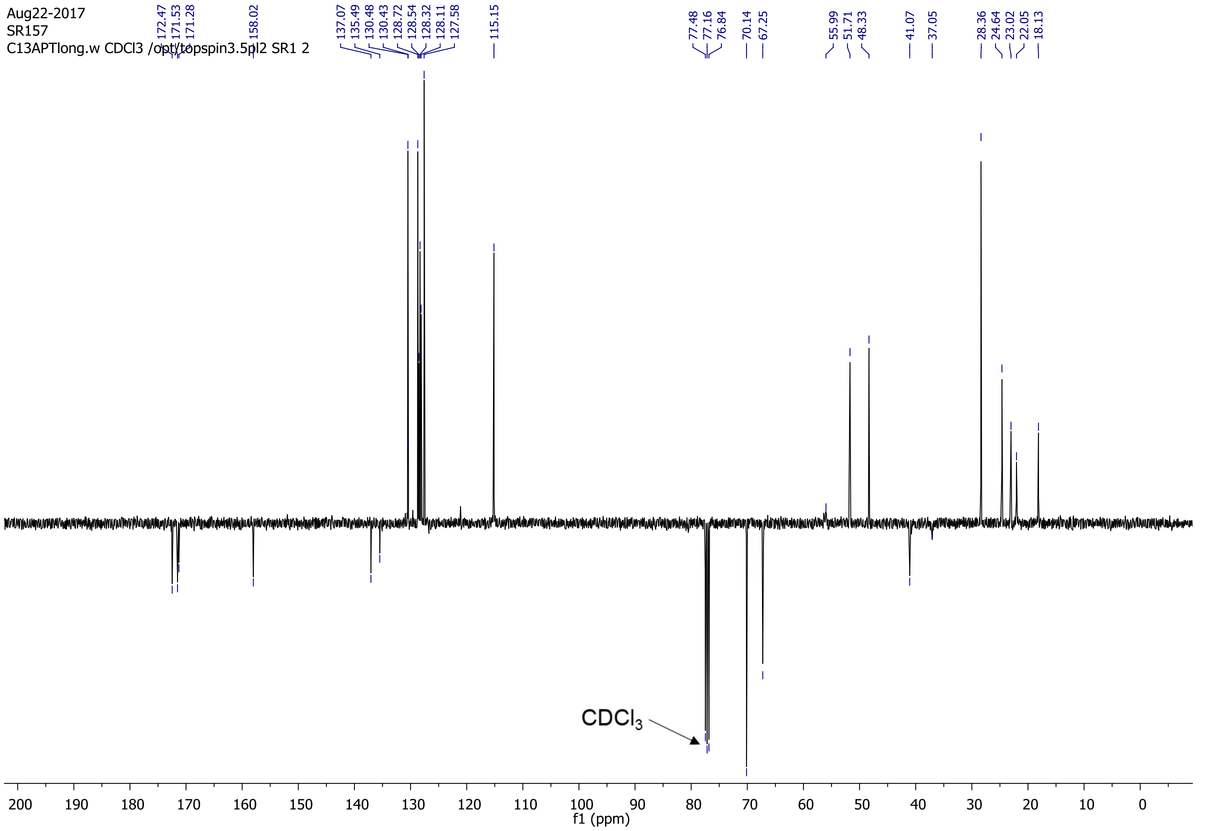

Cbz-GOx-Ala-Tyr(Bn)-Leu-Ala-OBn (79)

^{1}H NMR (400 MHz, CD_3OD)

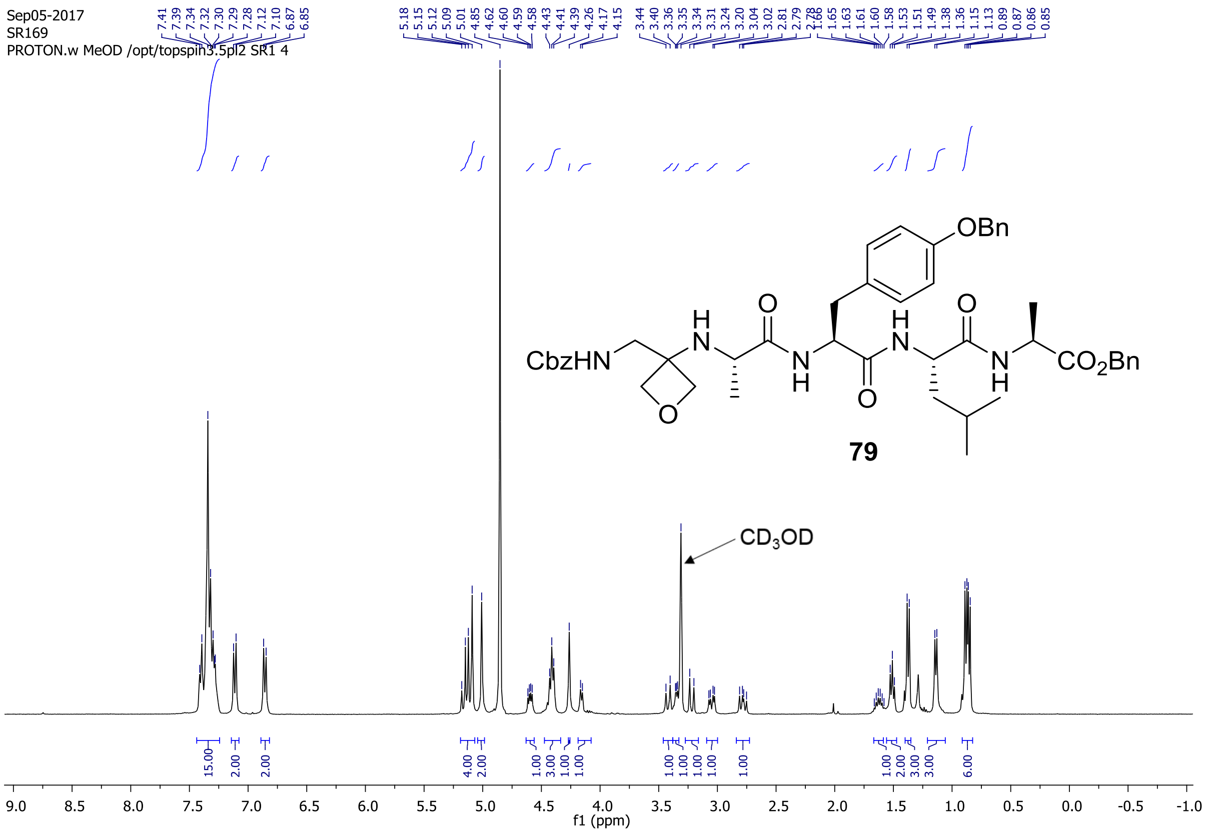

79

^{13}C NMR (101 MHz, CD_3OD)

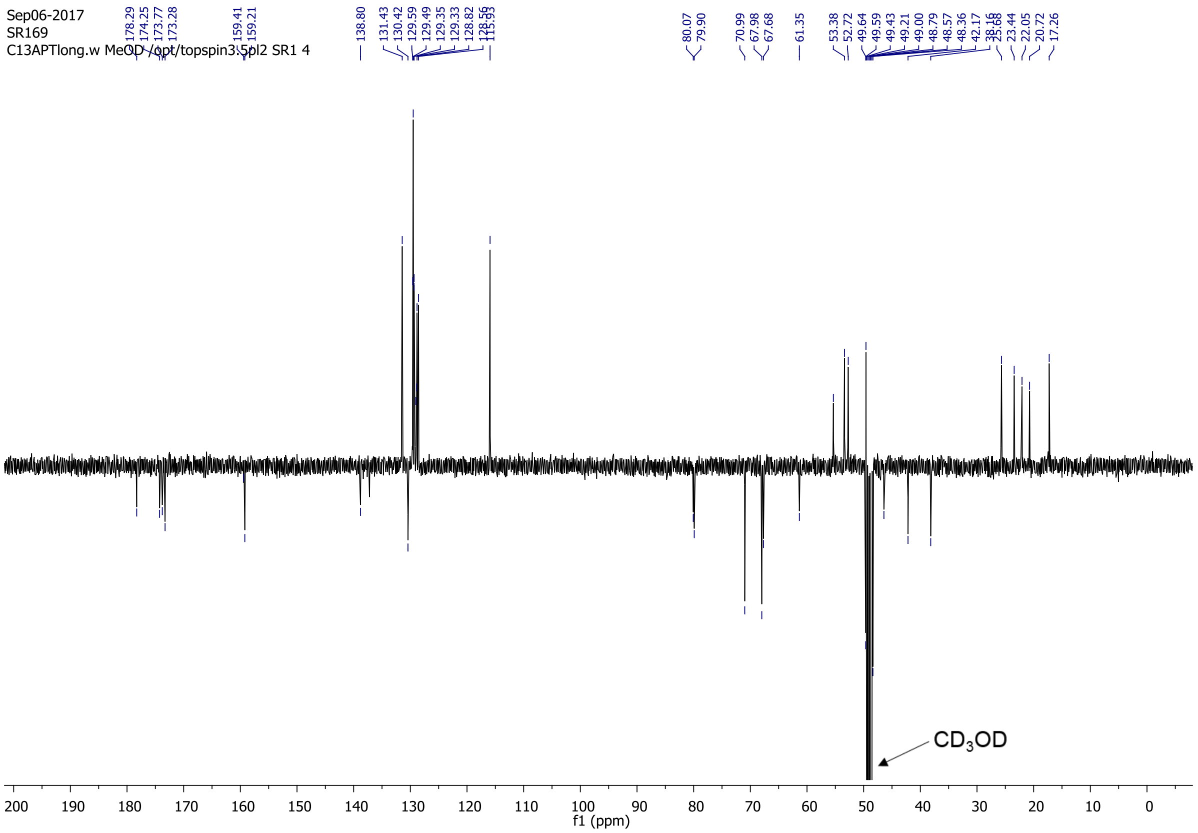

H-GOx-Ala-Tyr-Leu-Ala-OH (80)

^{1}H NMR (400 MHz, CD_3OD)

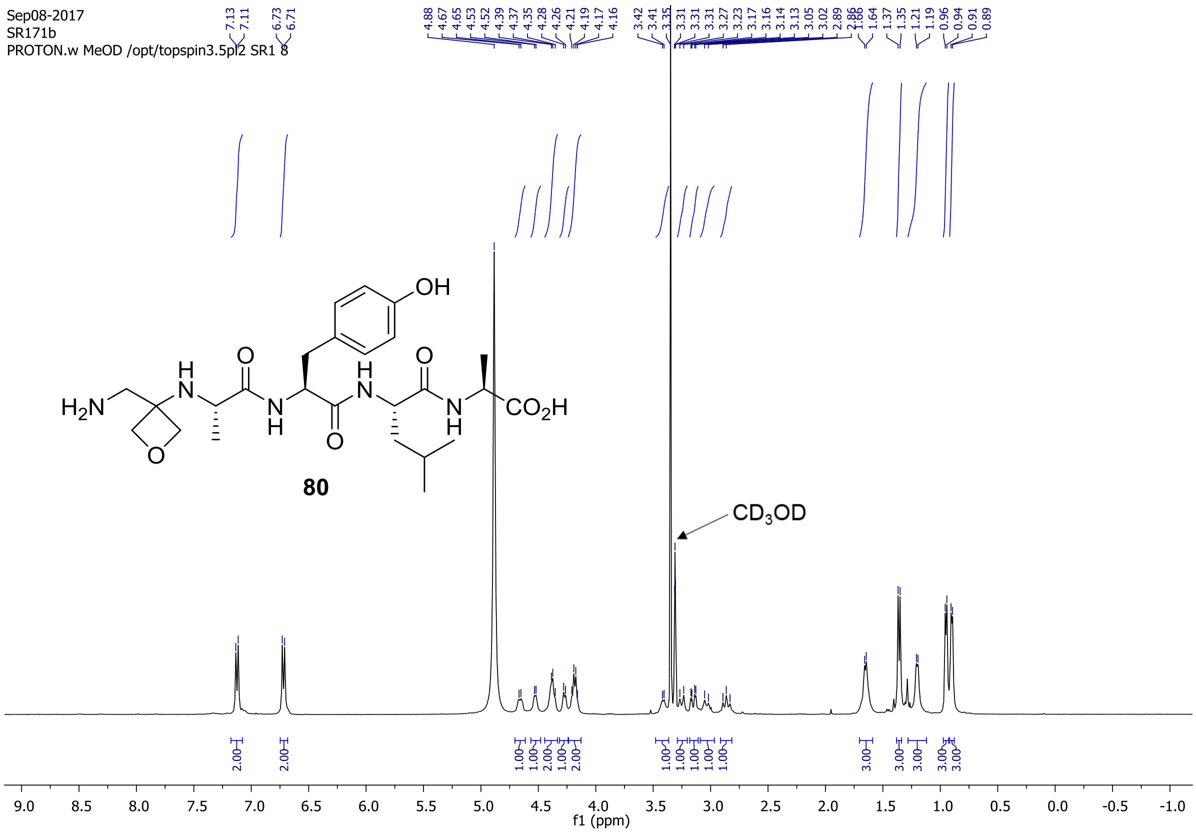

^{13}C NMR (101 MHz, CD_3OD)

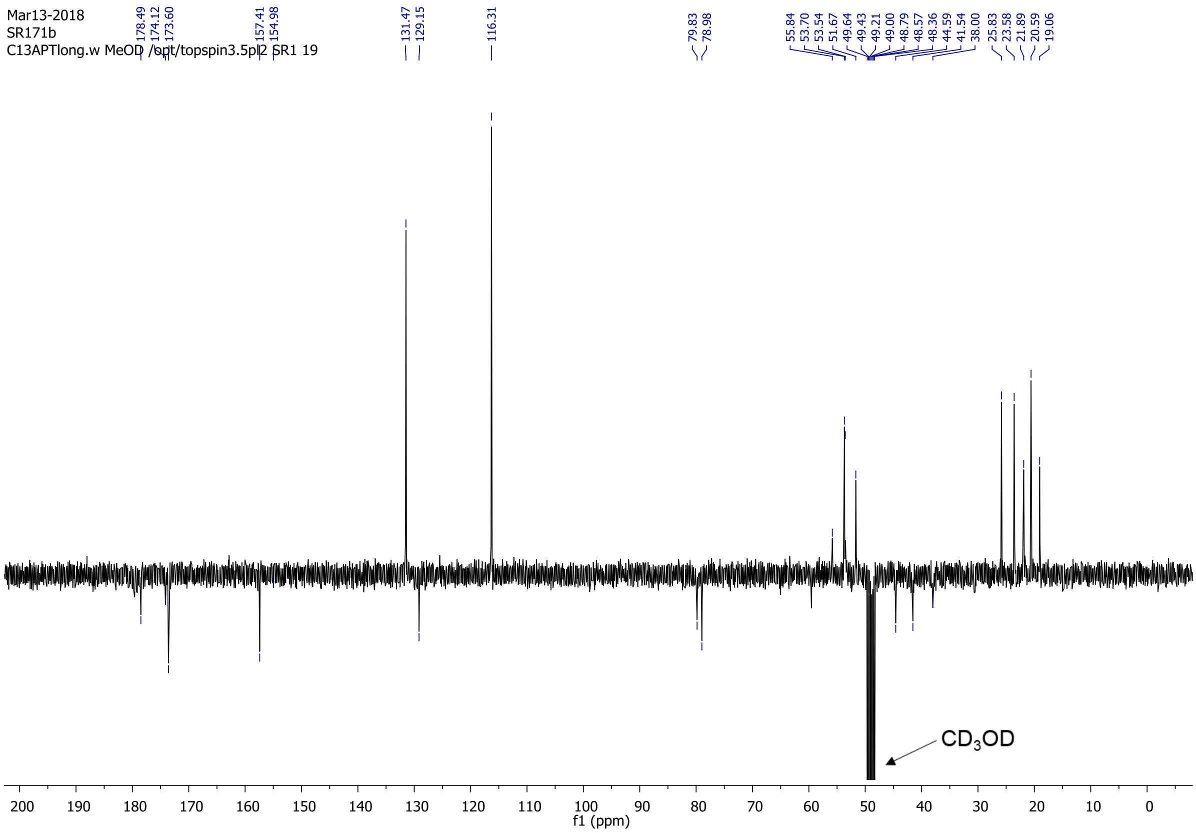

Cyclo(Ala-GOx-Ala-Tyr-Leu) (13)

^{1}H NMR (400 MHz, CD_3OD)

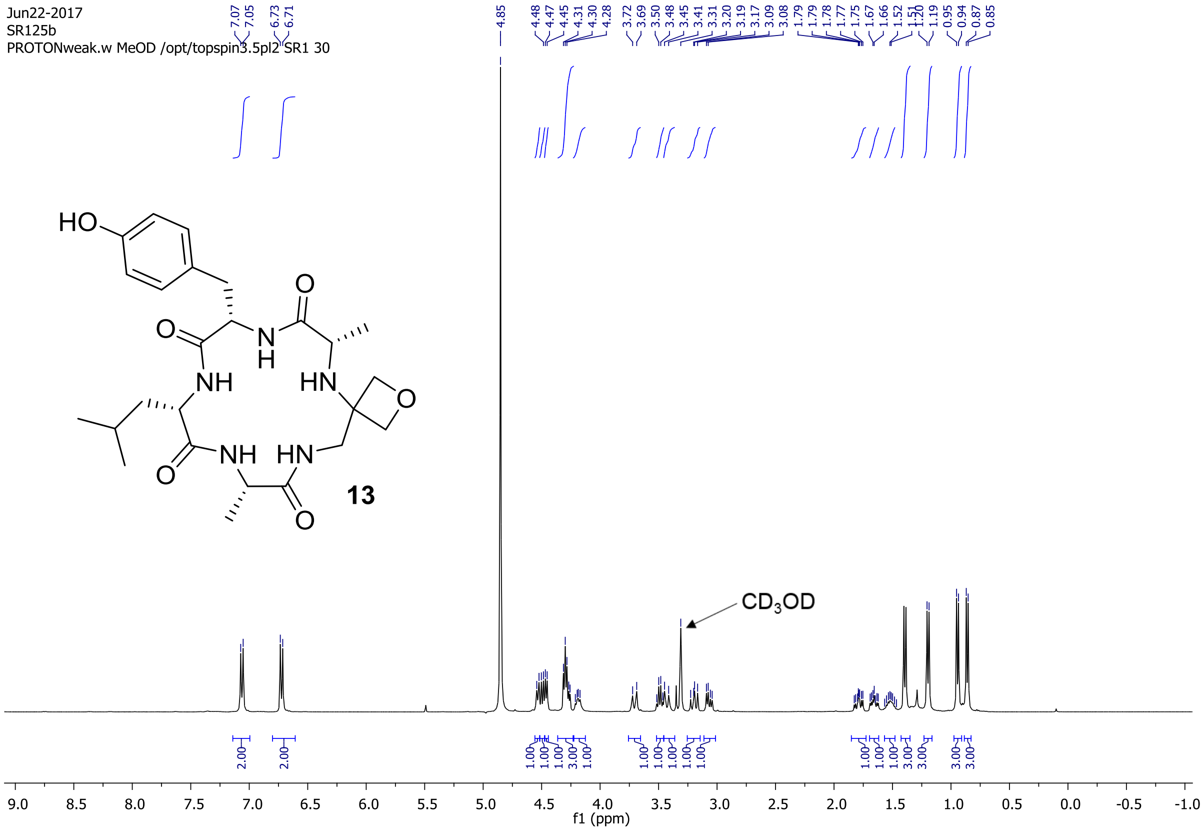

^{13}C NMR (126 MHz, CD_3OD)

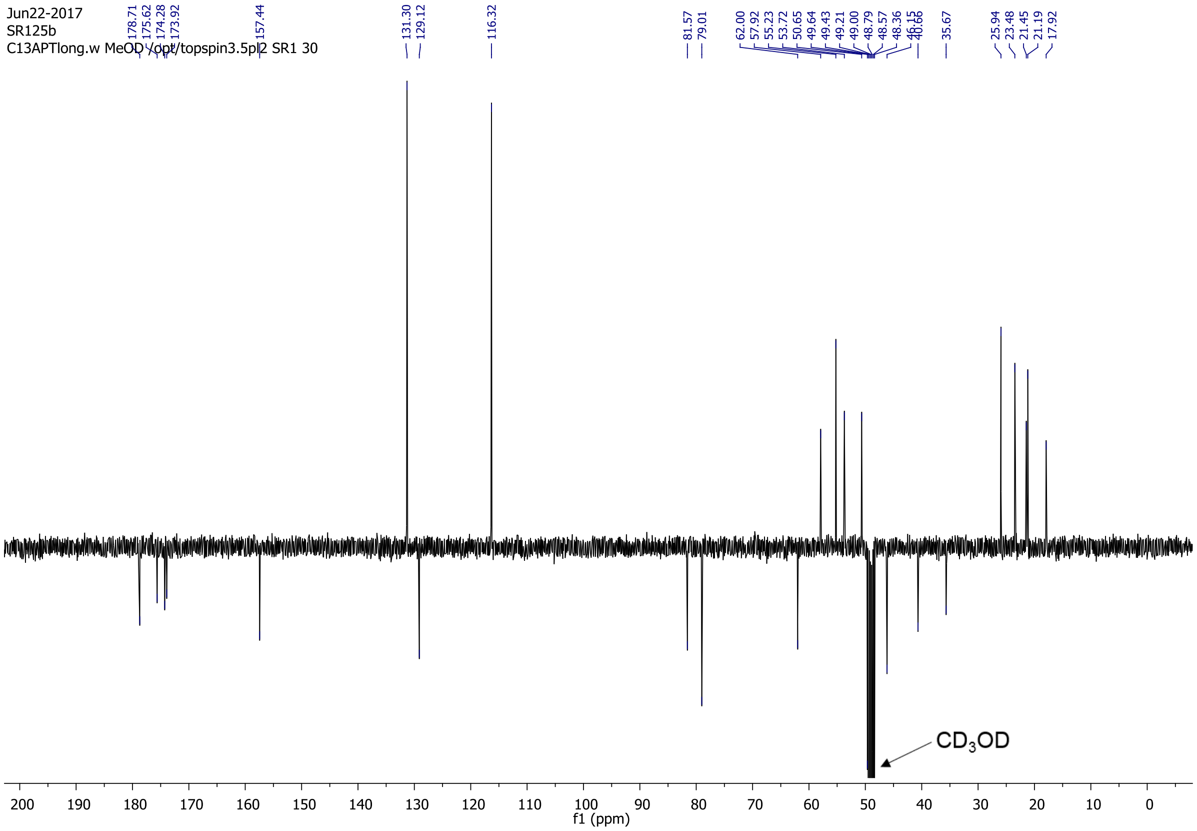

Boc-Gly-Ala-Tyr(Bn)-Leu-OBn (81)

^{1}H NMR (400 MHz, $CDCl_3$)

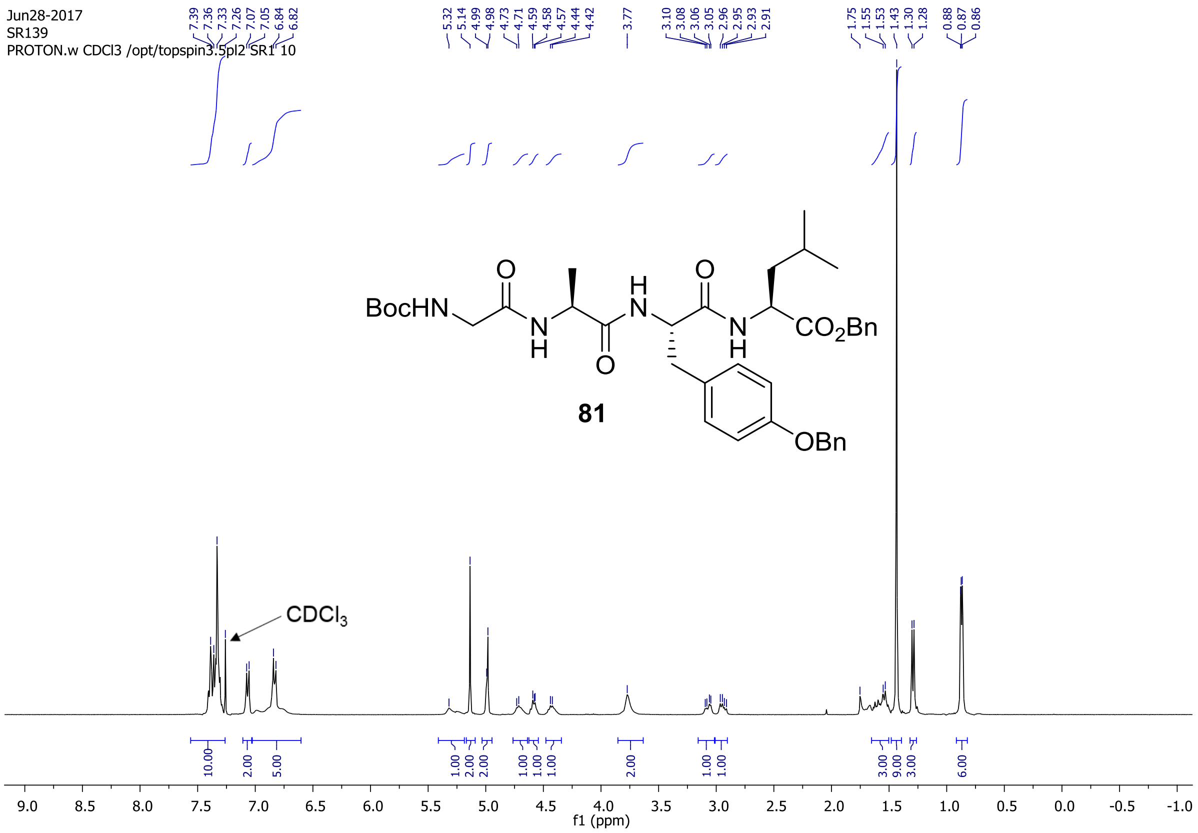

^{13}C NMR (101 MHz, $CDCl_3$)

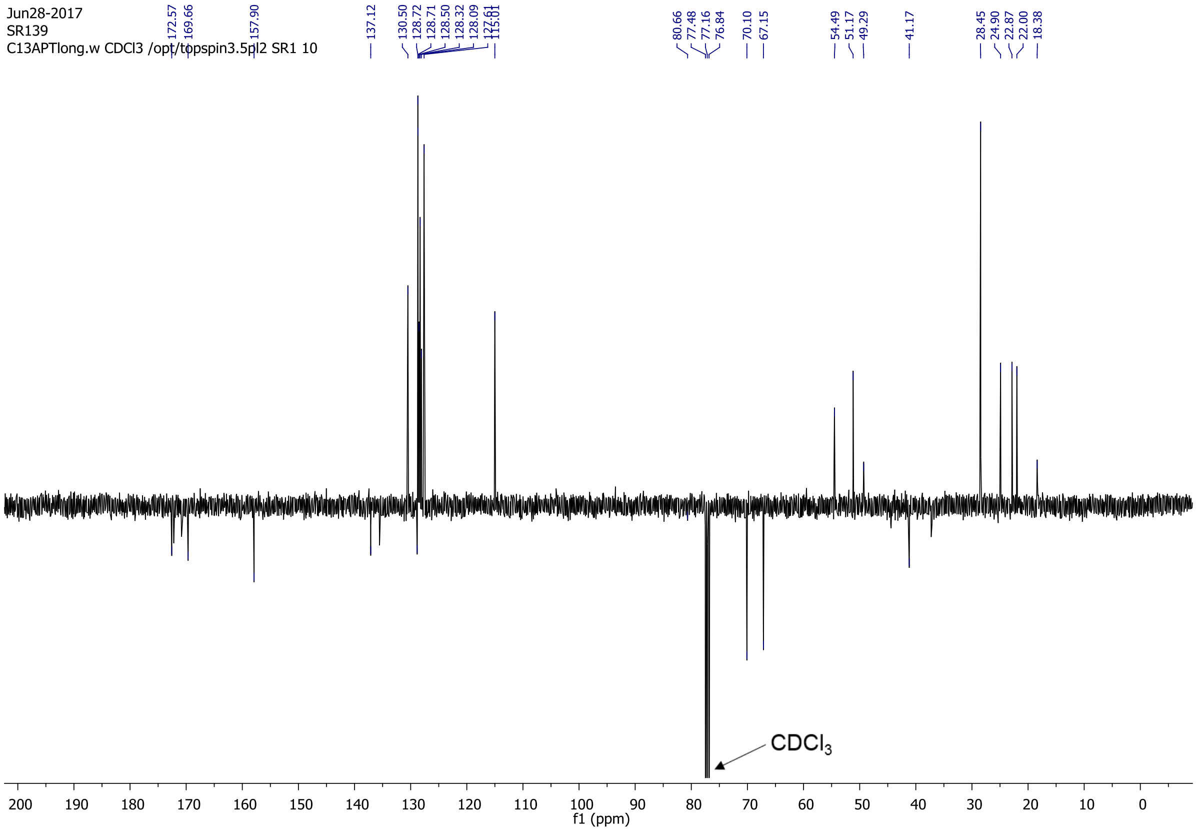

Cbz-Ala-Gly-Ala-Tyr(Bn)-Leu-OBn (82)

^{1}H NMR (400 MHz, DMSO-*d6*)

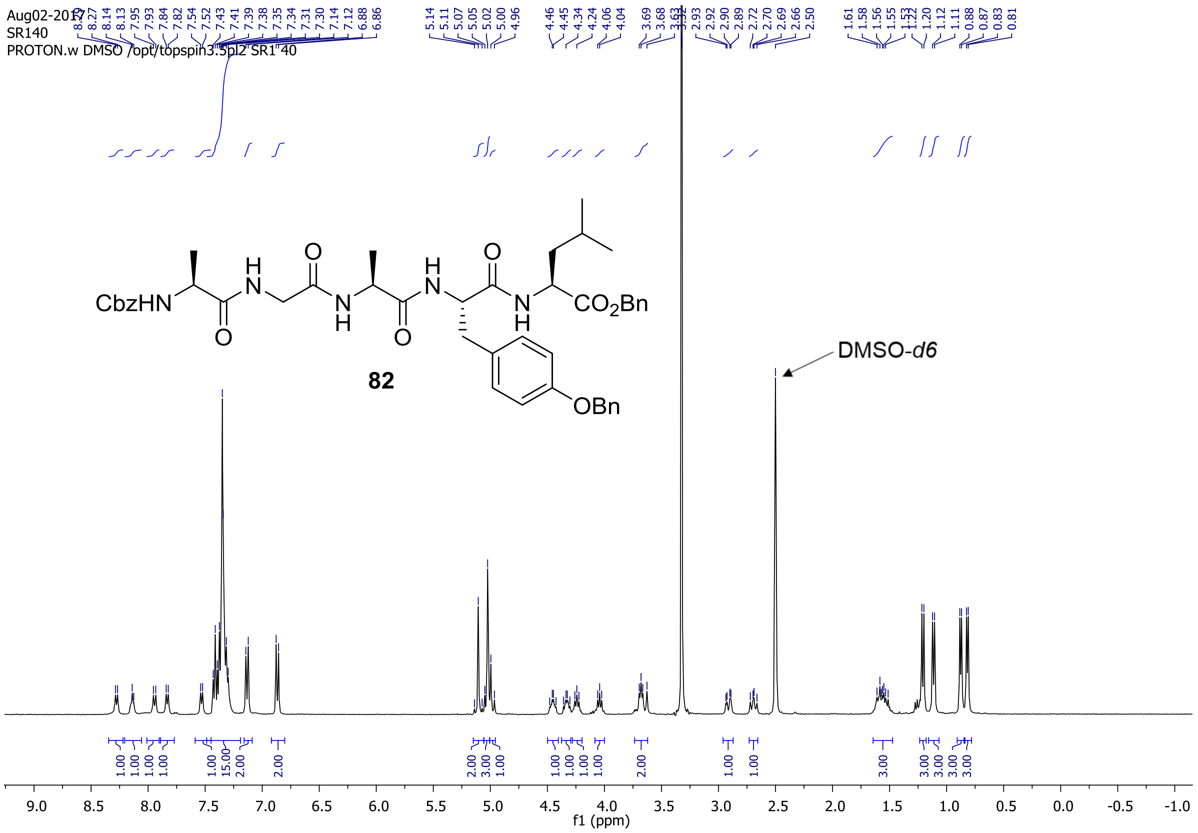

^{13}C NMR (101 MHz, DMSO-*d6*)

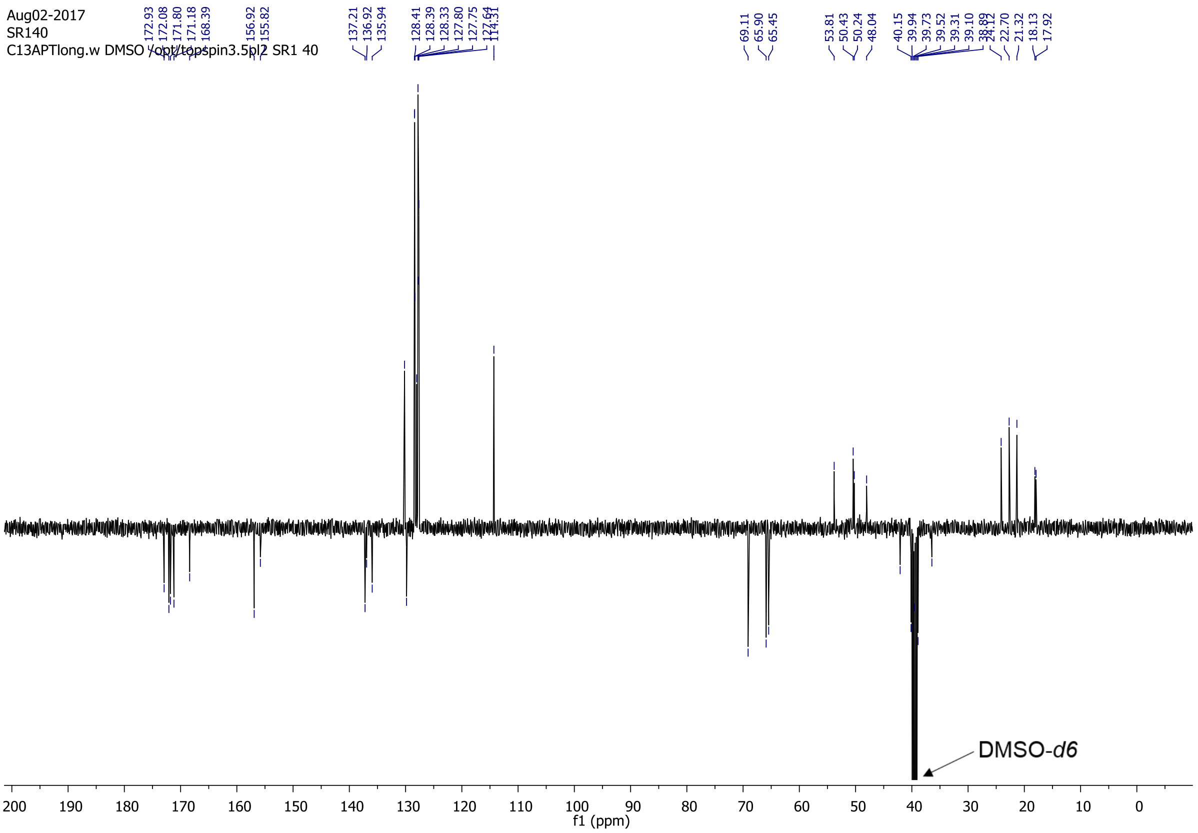

H-Ala-Gly-Ala-Tyr-Leu-OH (83)

^{1}H NMR (400 MHz, D_2O)

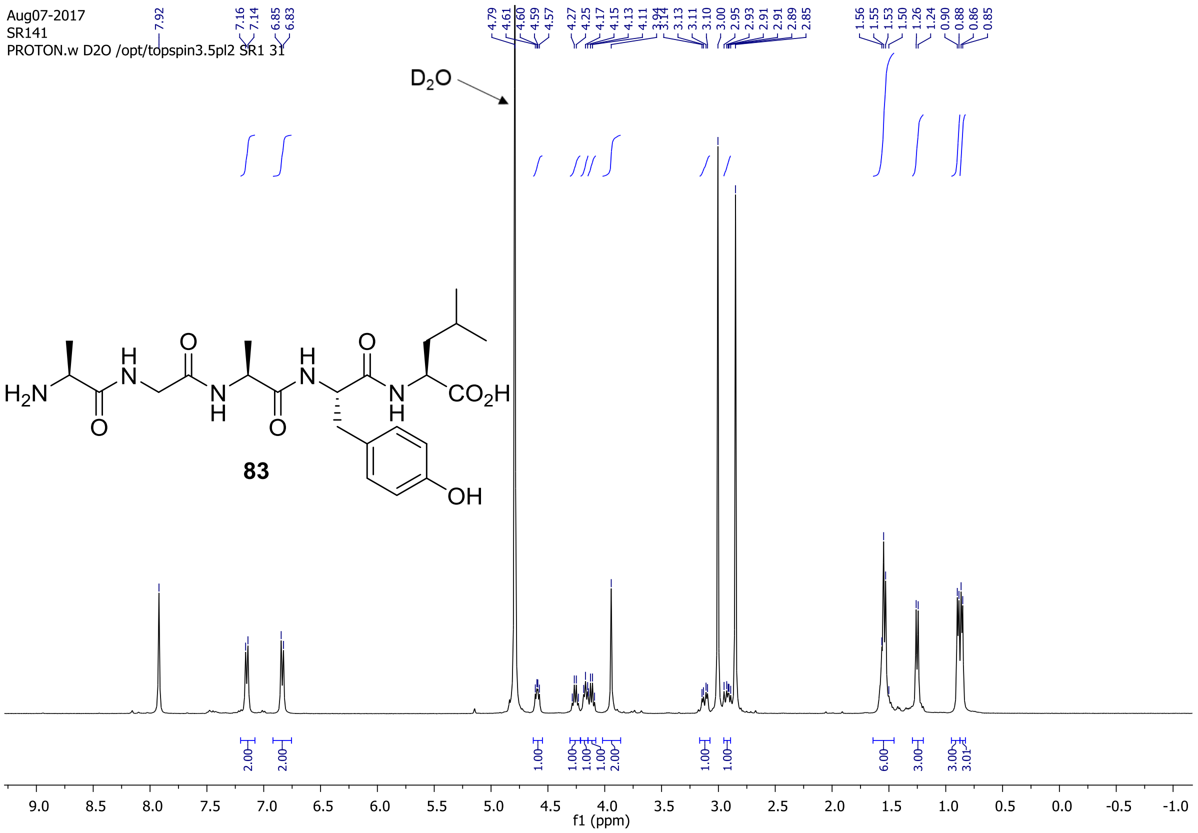

^{13}C NMR (101 MHz, D_2O)

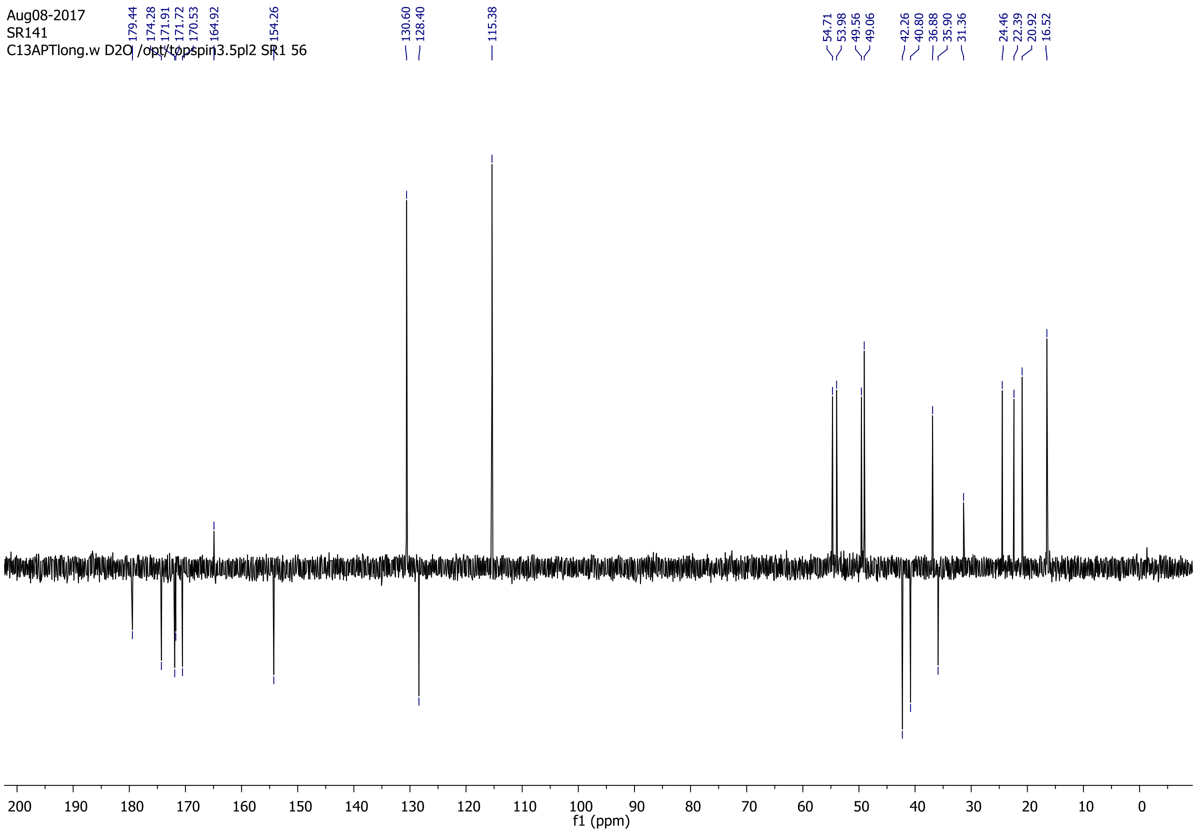

Boc-Gly-Ala-Tyr(Bn)-OBn (84)

^{1}H NMR (400 MHz, $CDCl_3$)

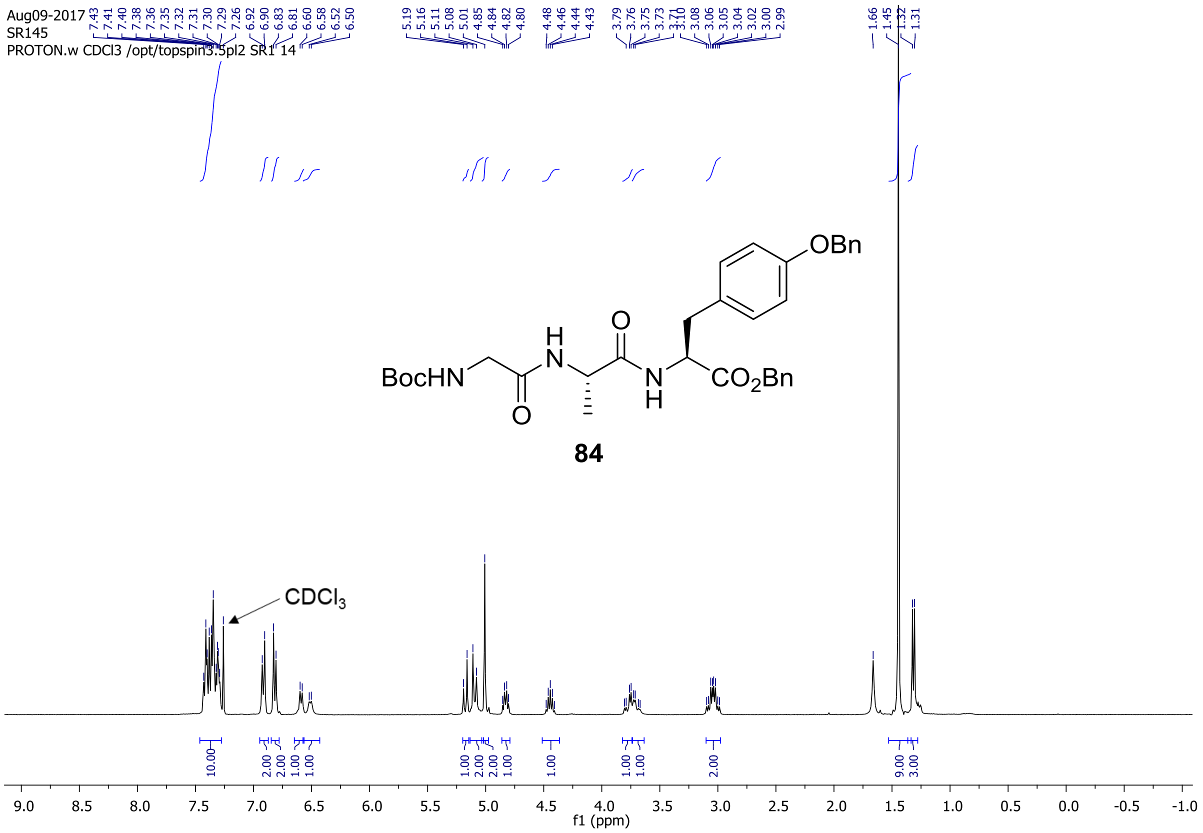

^{13}C NMR (101 MHz, $CDCl_3$)

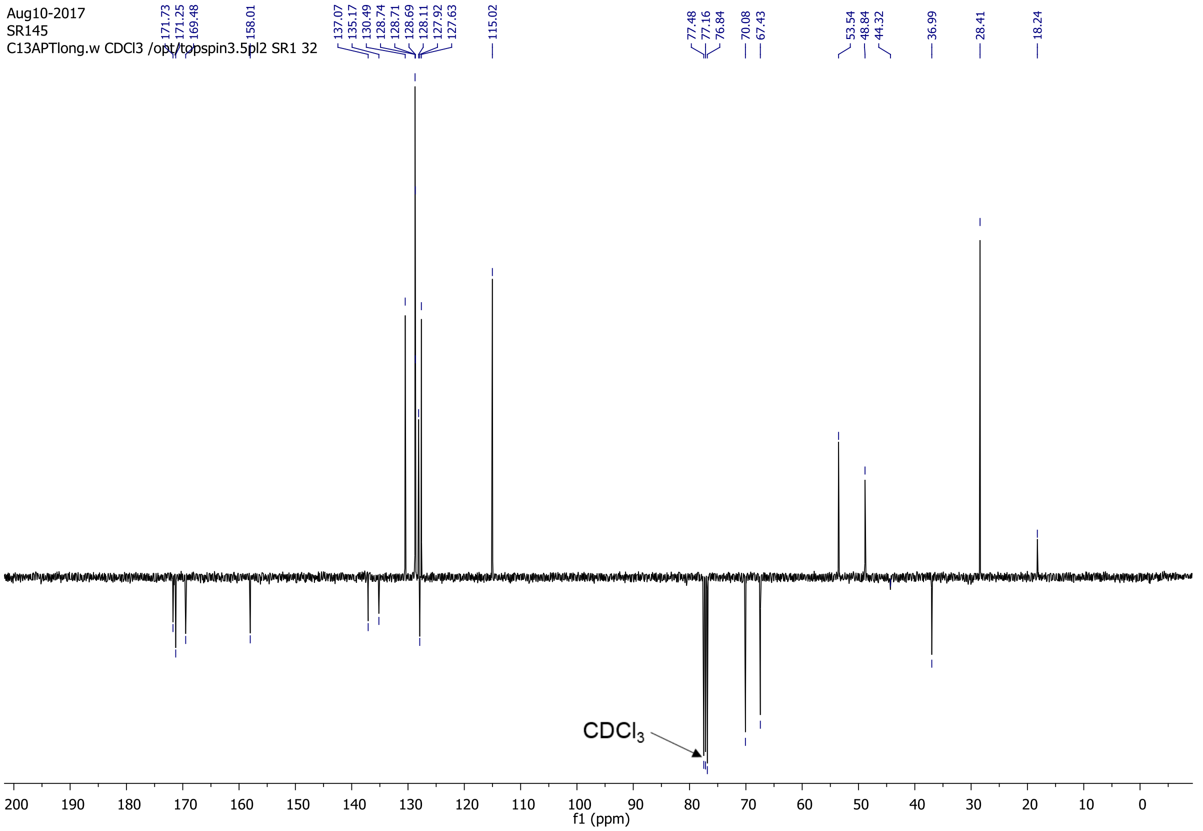

Boc-Ala-Gly-Ala-Tyr(Bn)-OBn (85)

^{1}H NMR (400 MHz, DMSO-*d6*)

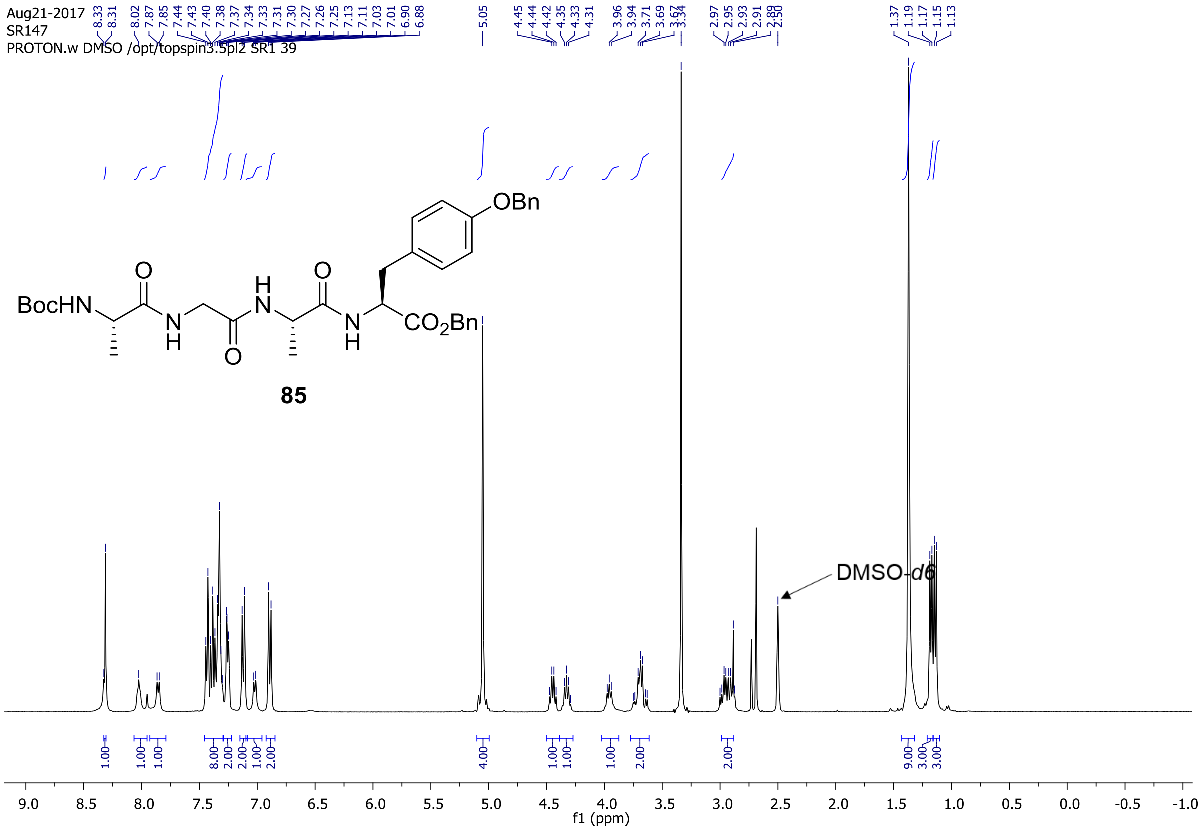

^{13}C NMR (101 MHz, DMSO-*d6*)

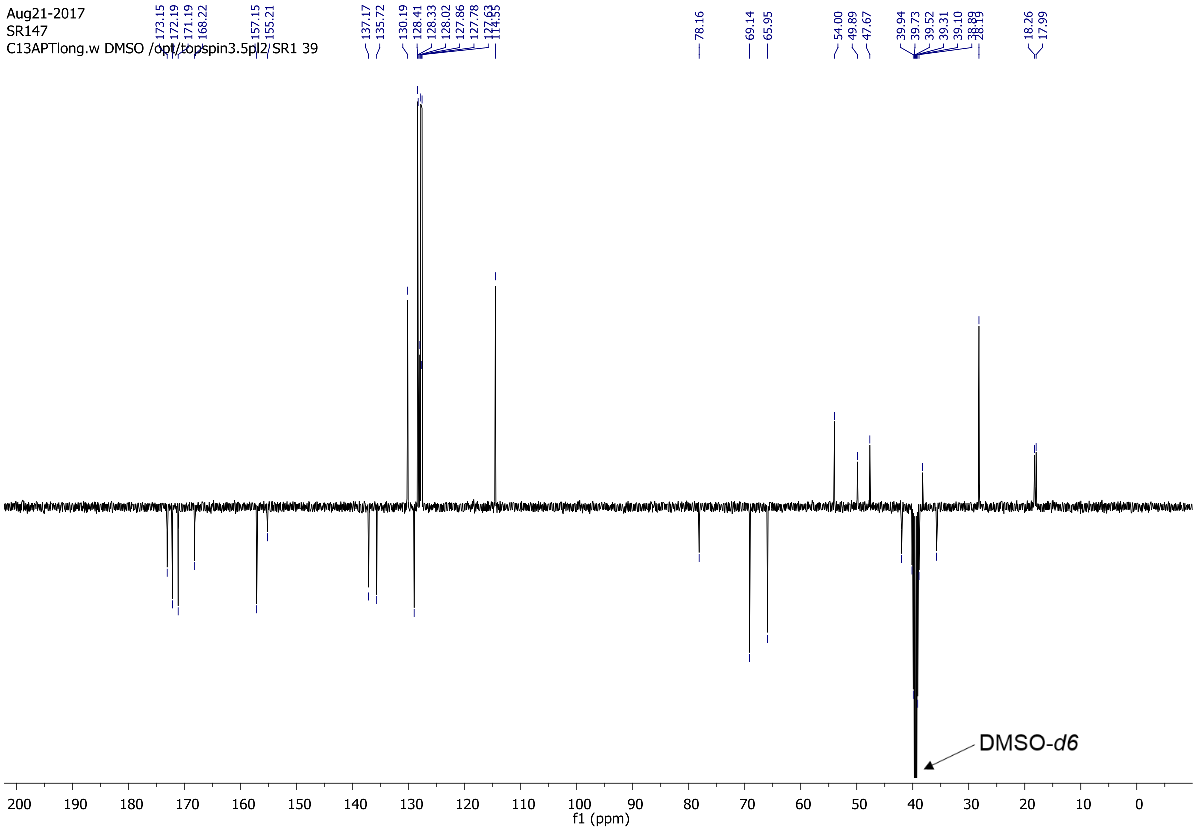

Cbz-Leu-Ala-Gly-Ala-Tyr(Bn)-OBn (86)

^{1}H NMR (400 MHz, DMSO-*d6*)

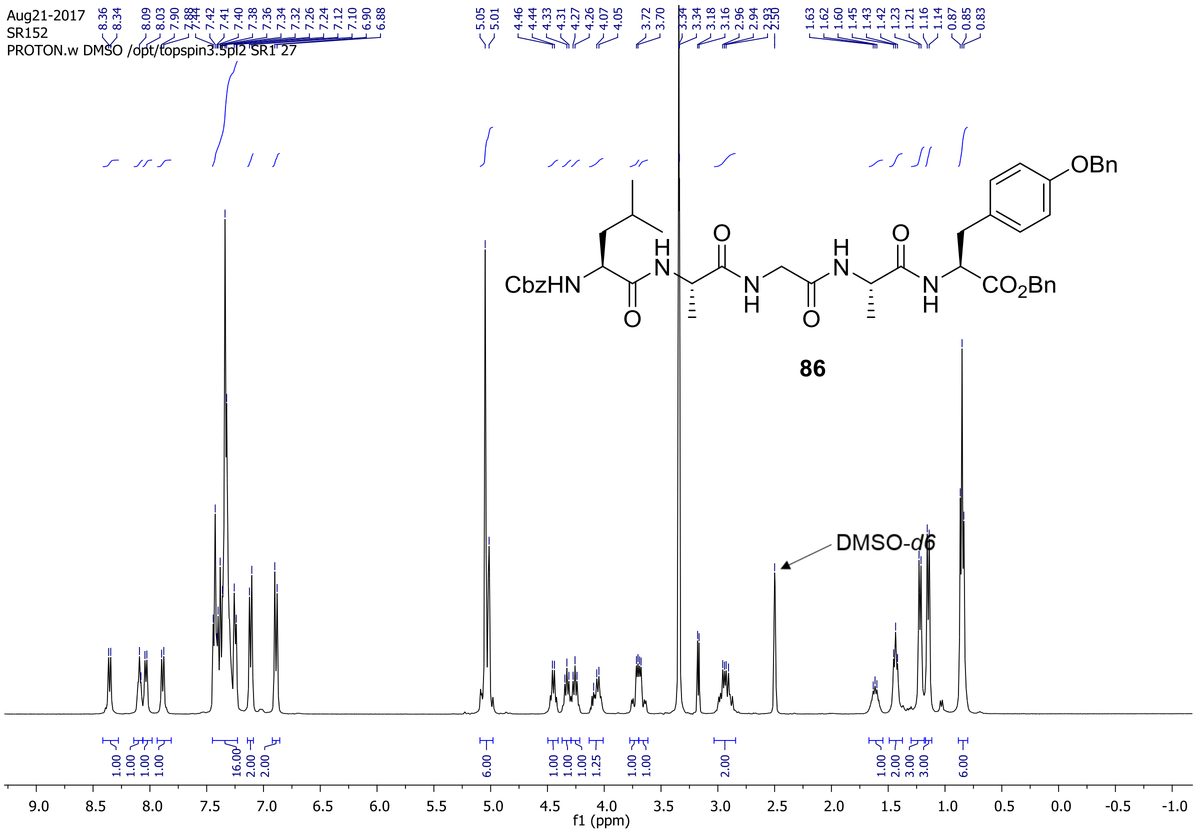

^{13}C NMR (101 MHz, DMSO-*d6*)

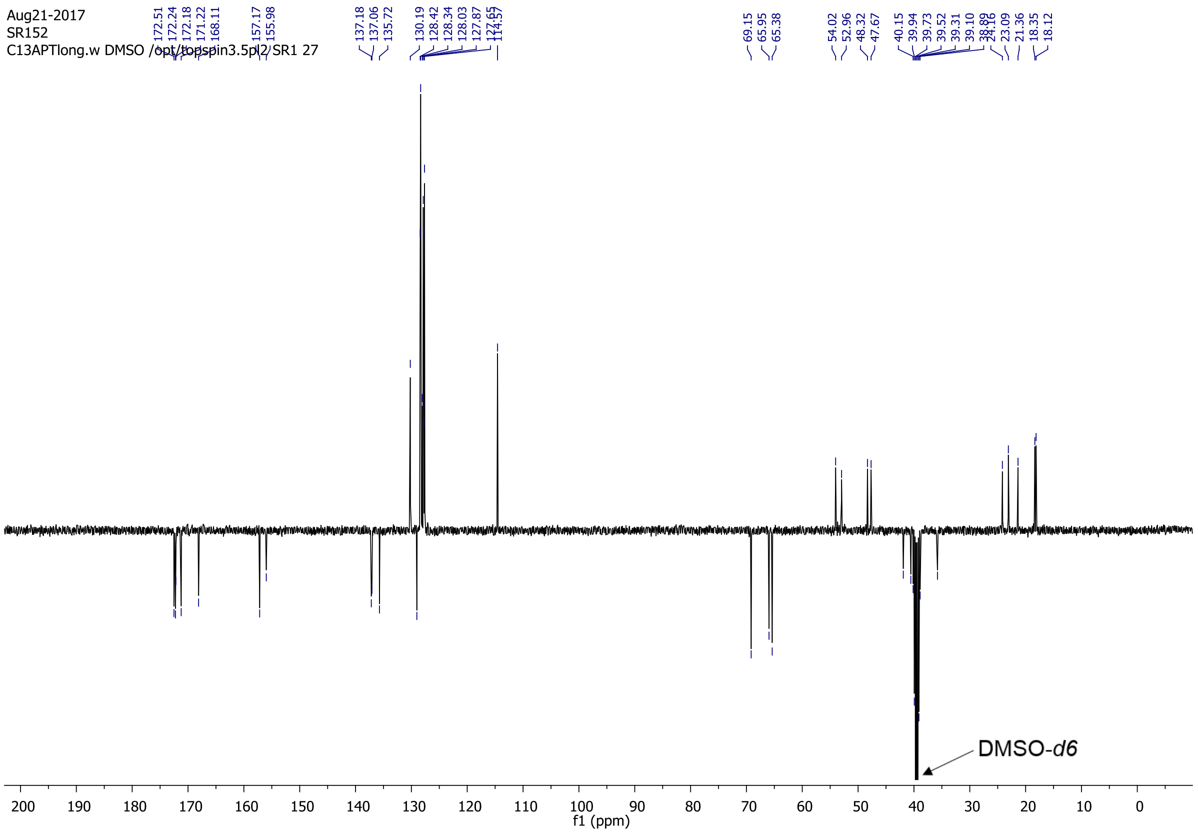

H-Leu-Ala-Gly-Ala-Tyr-OH (87)

^{1}H NMR (400 MHz, DMSO-*d6*)

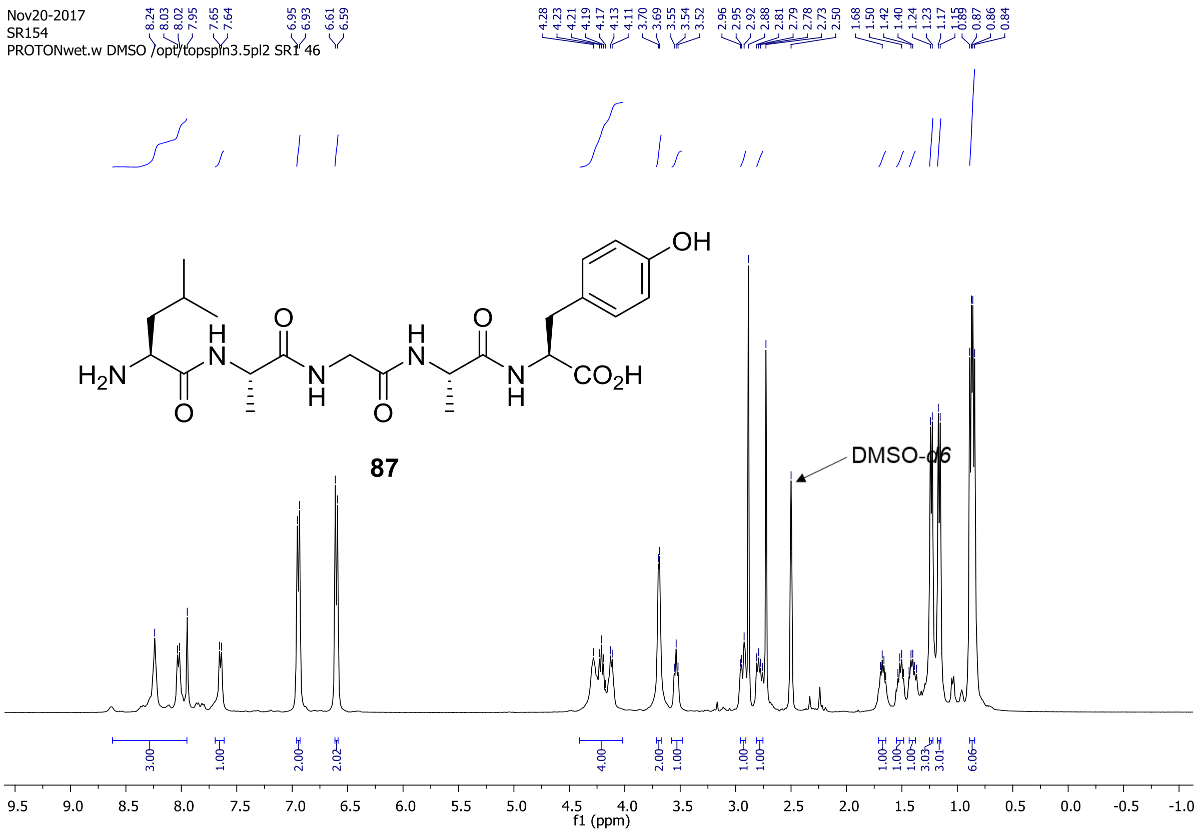

^{13}C NMR (101 MHz, DMSO-*d6*)

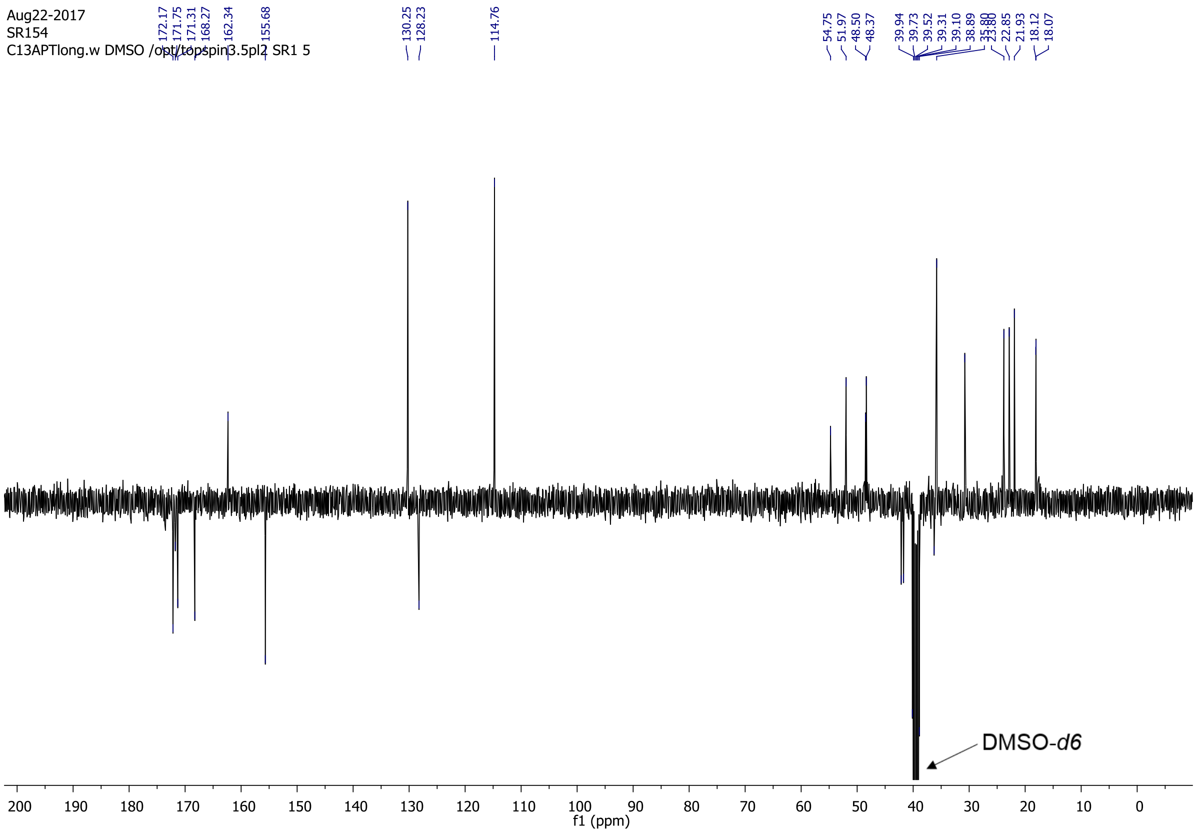

Cyclo(Ala-Gly-Ala-Tyr-Leu) (25)

^{1}H NMR (400 MHz, CD_3OD)

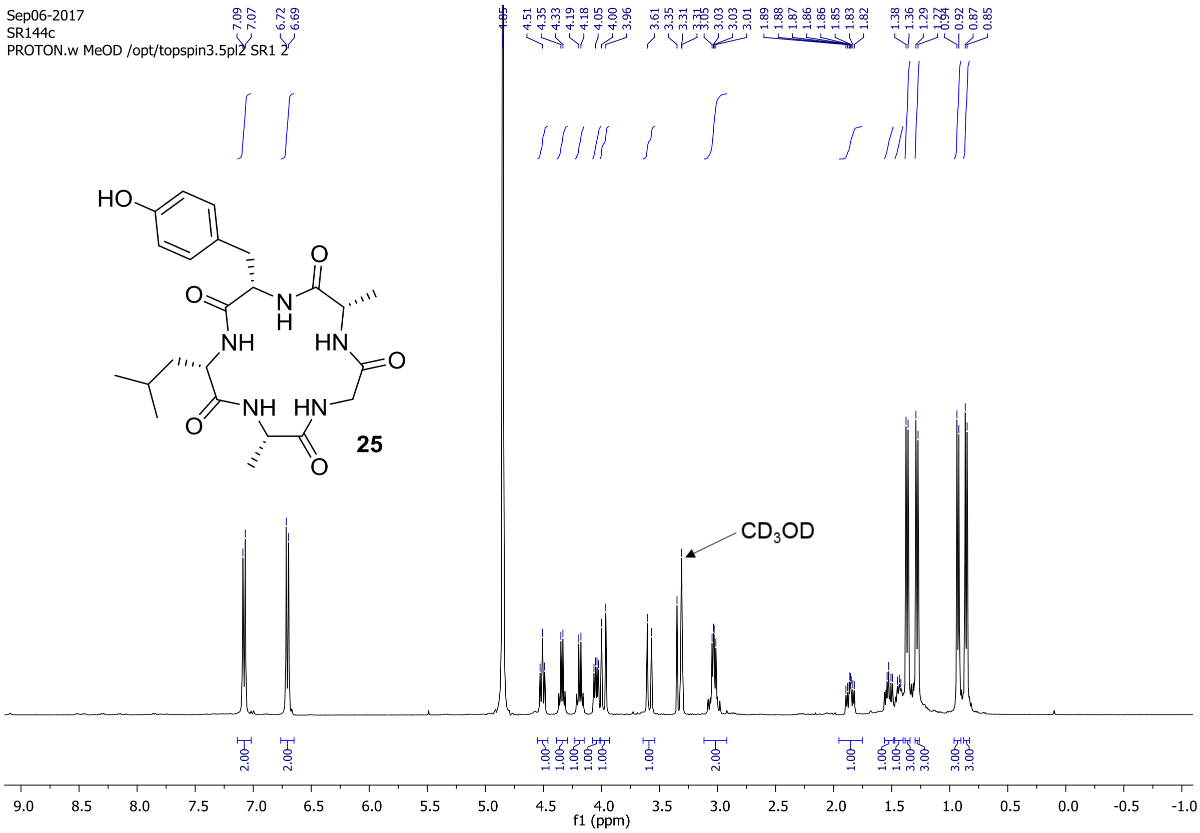

^{13}C NMR (101 MHz, CD_3OD)

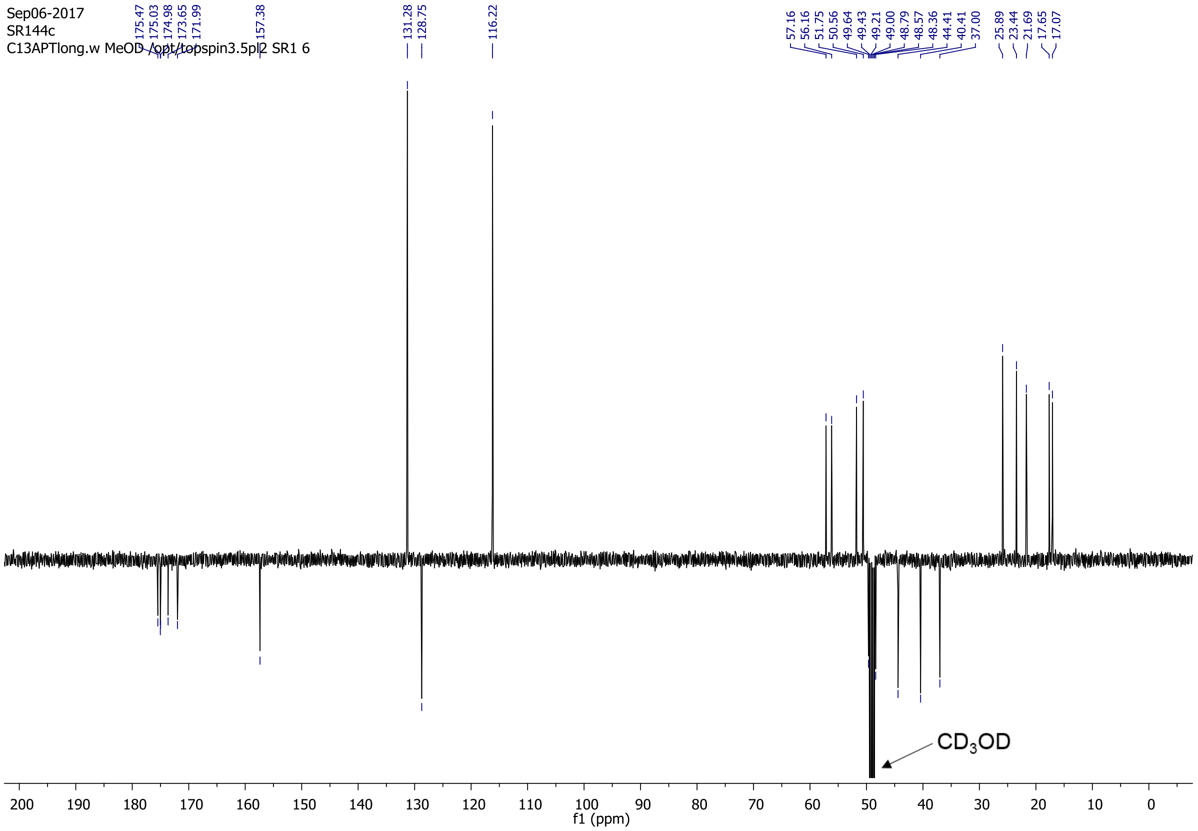

Boc-Sar-Ala-Tyr(Bn)-OBn (88)

^{1}H NMR (500 MHz, $CDCl_3$)

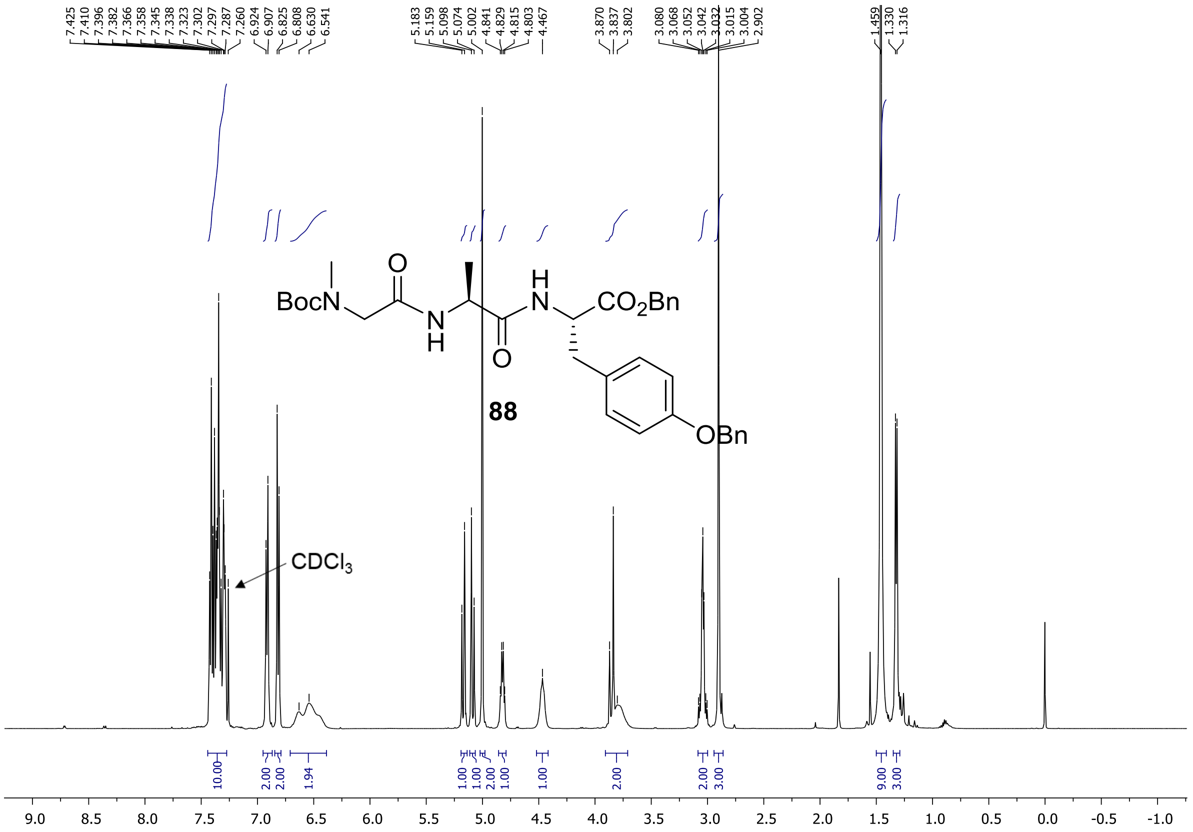

^{13}C NMR (126 MHz, $CDCl_3$)

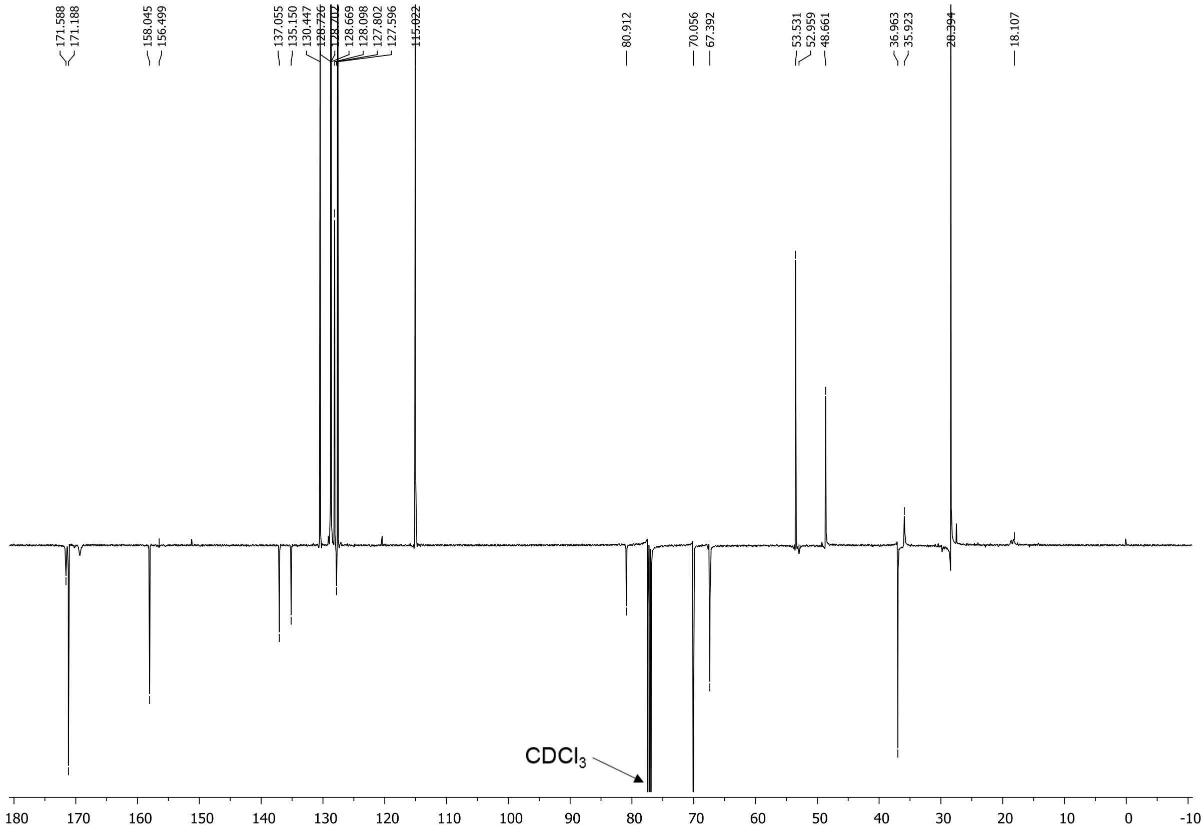

Boc-Ala-Sar-Ala-Tyr(Bn)-OBn (89)

^{1}H NMR (500 MHz, DMSO-*d6* @ 373 K)

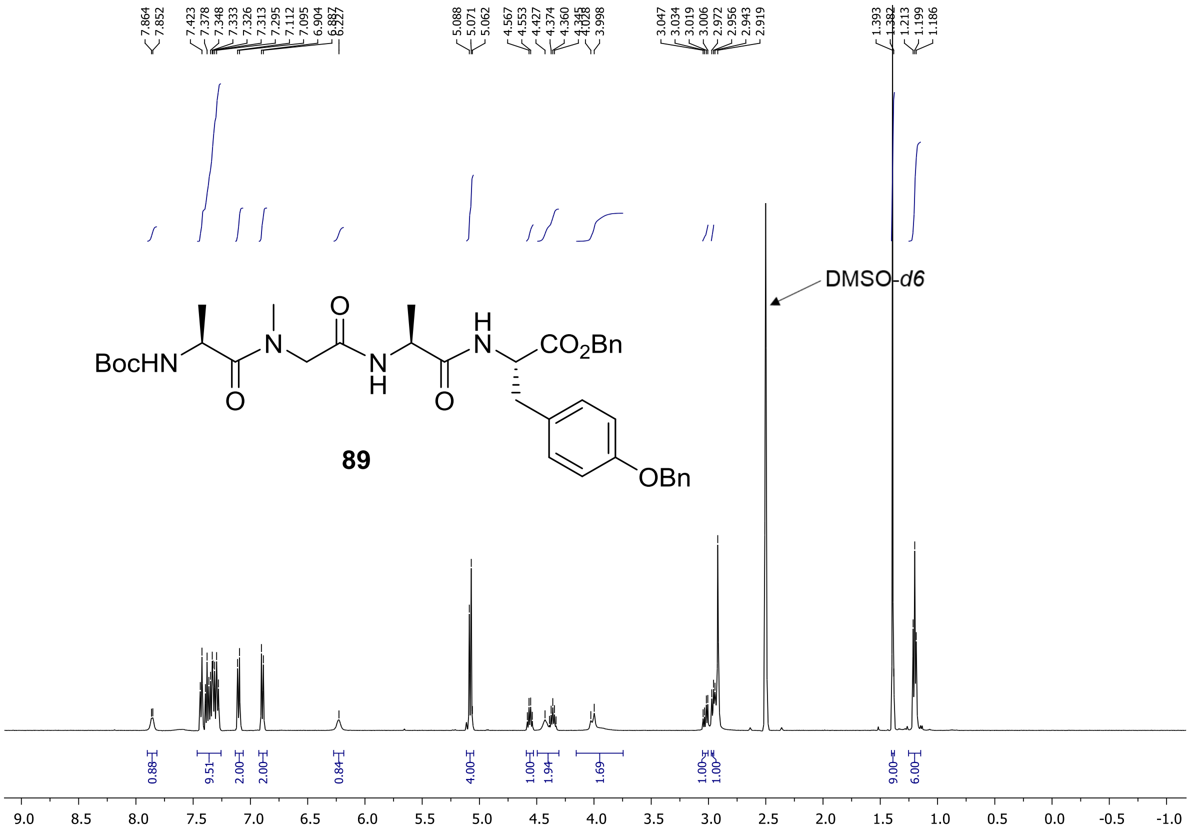

^{13}C NMR (126 MHz, DMSO-*d6* @ 373 K)

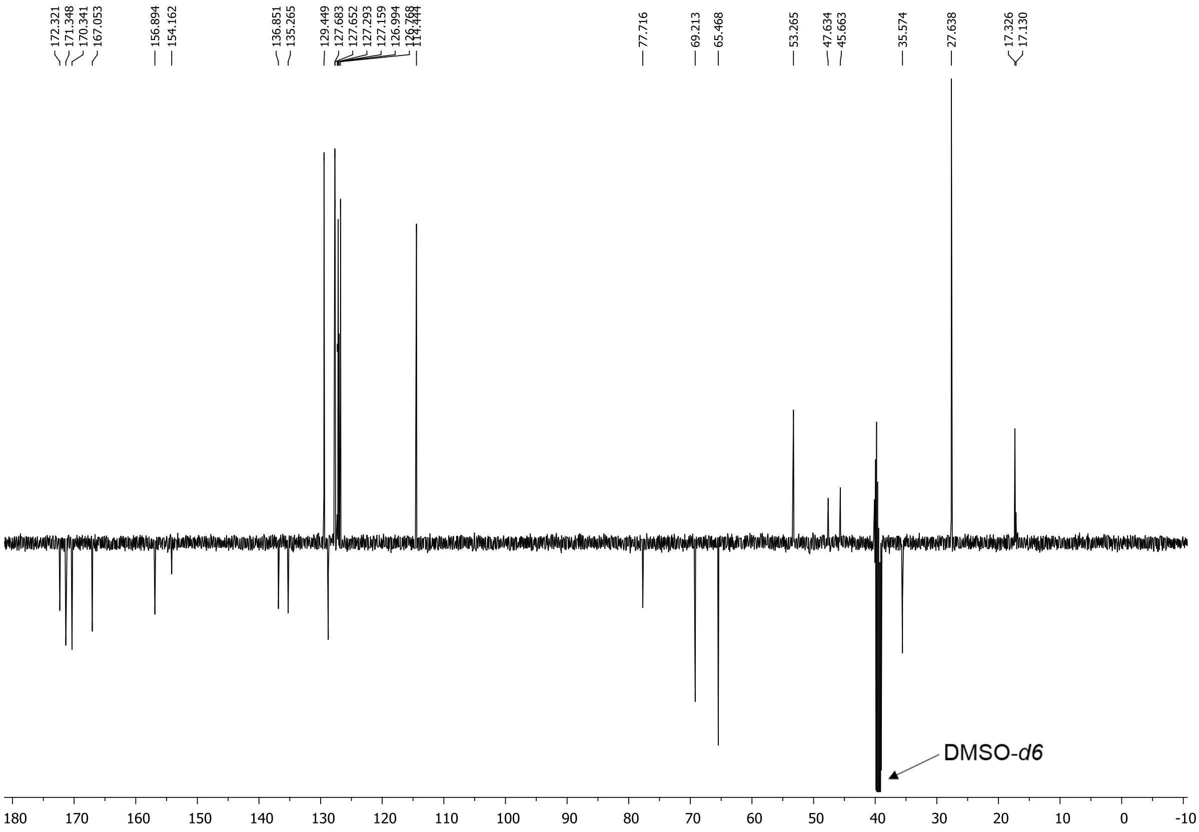

Cbz-Leu-Ala-Sar-Ala-Tyr(Bn)-OBn (90)

^{1}H NMR (500 MHz, DMSO-*d6* @ 373 K)

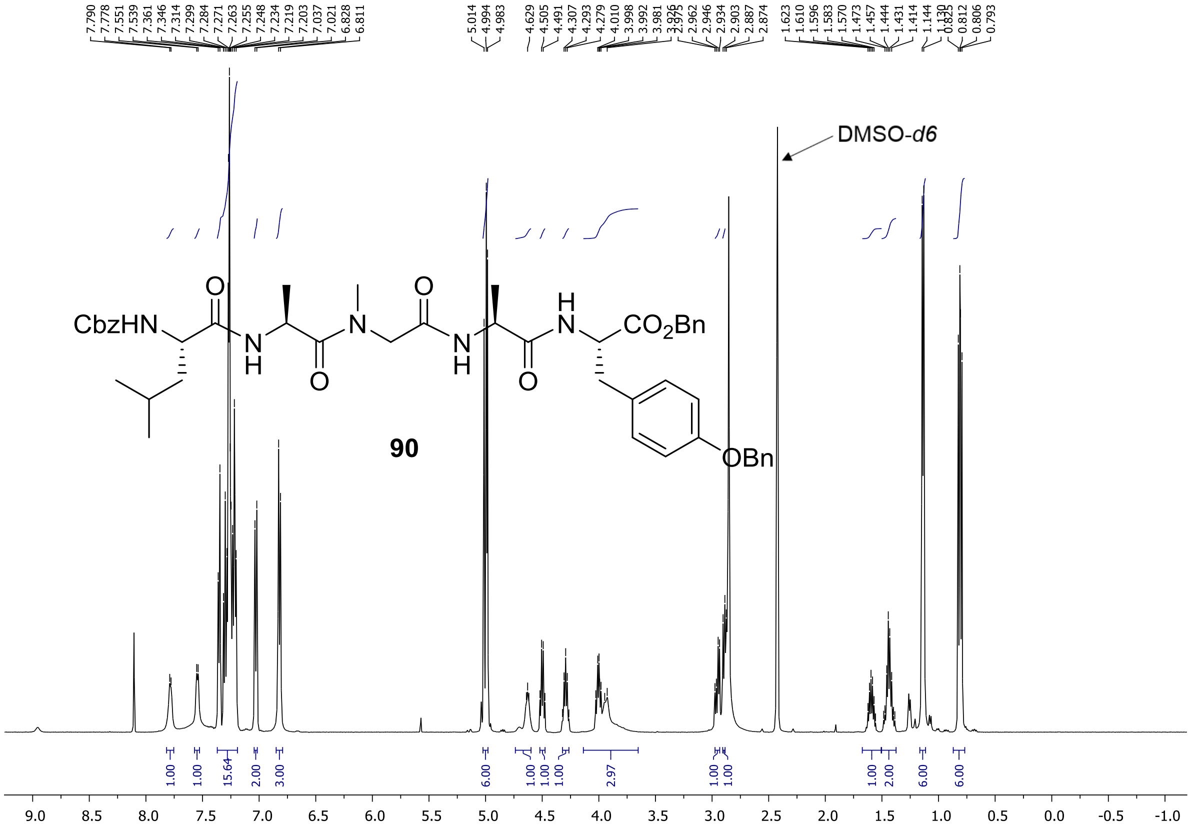

^{13}C NMR (126 MHz, DMSO-*d6* @ 373 K)

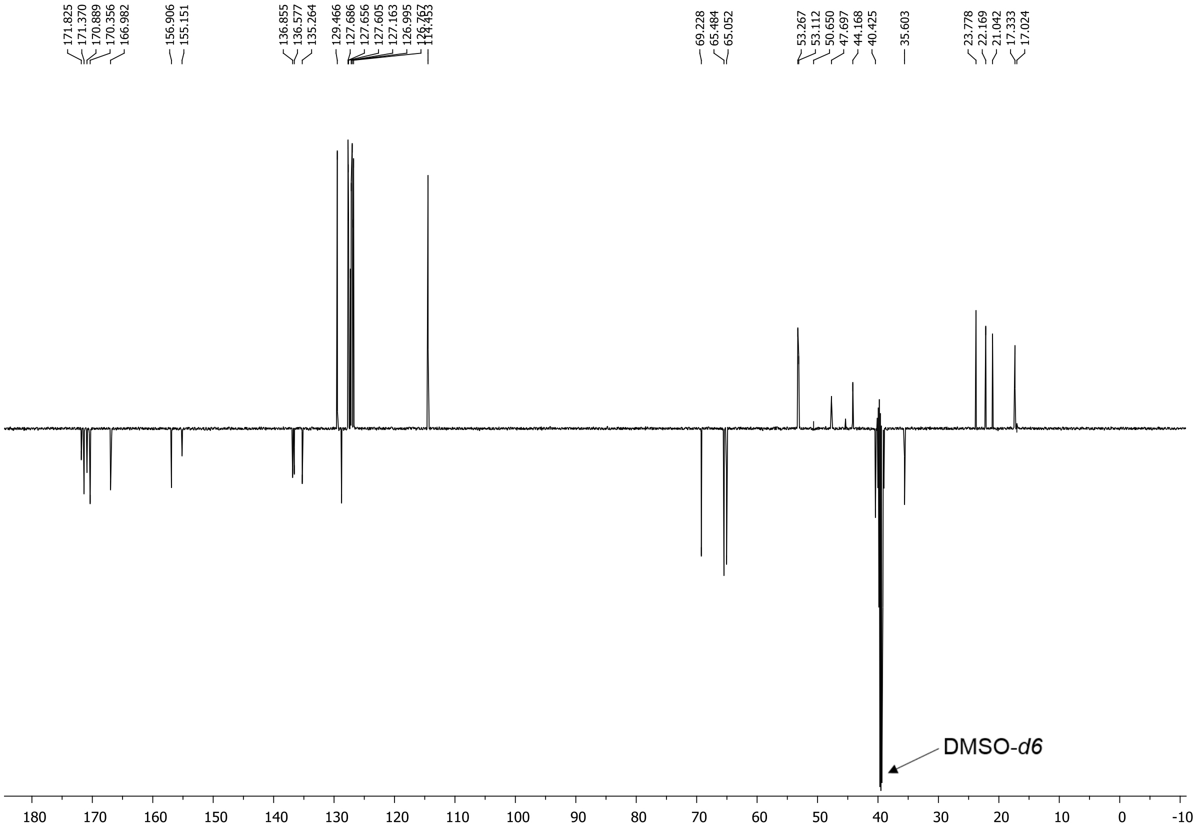

H-Leu-Ala-Sar-Ala-Tyr-OH (91)

^{1}H NMR (500 MHz, DMSO-*d6* @ 373 K)

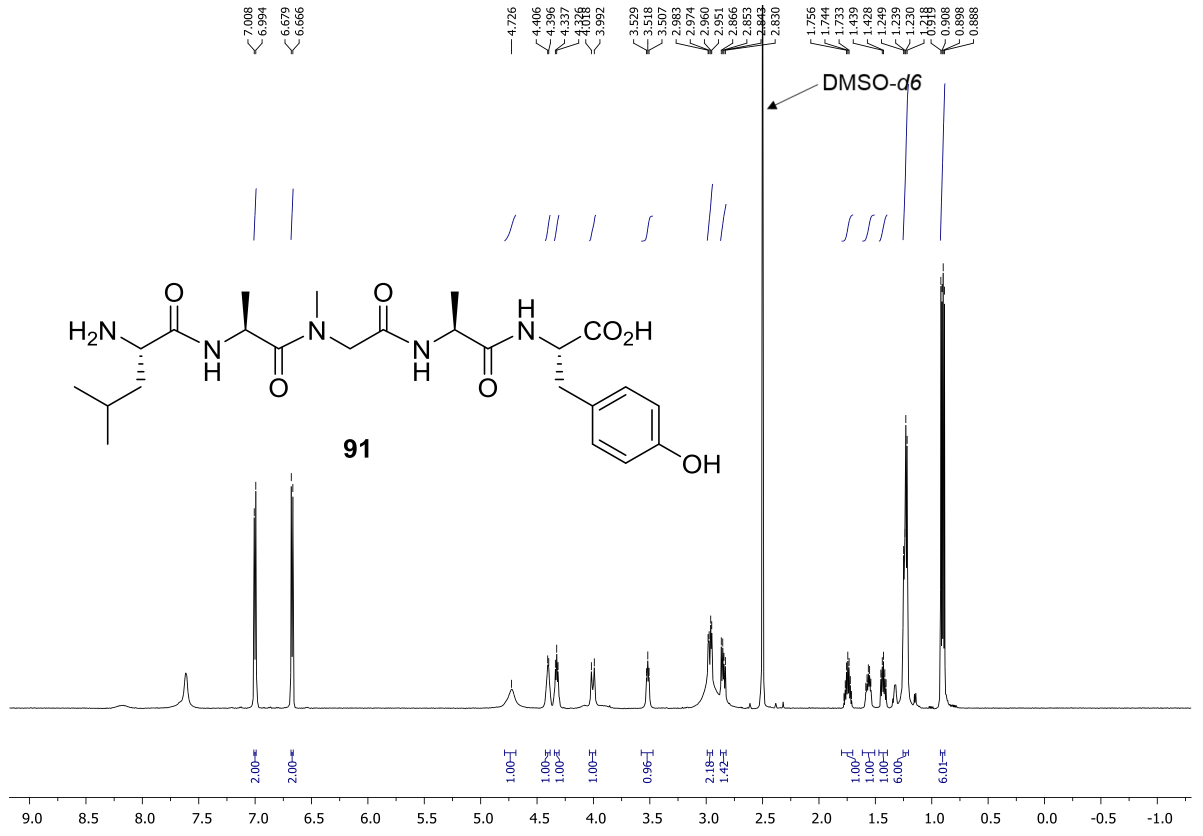

^{13}C NMR (126 MHz, DMSO-*d6* @ 373 K)

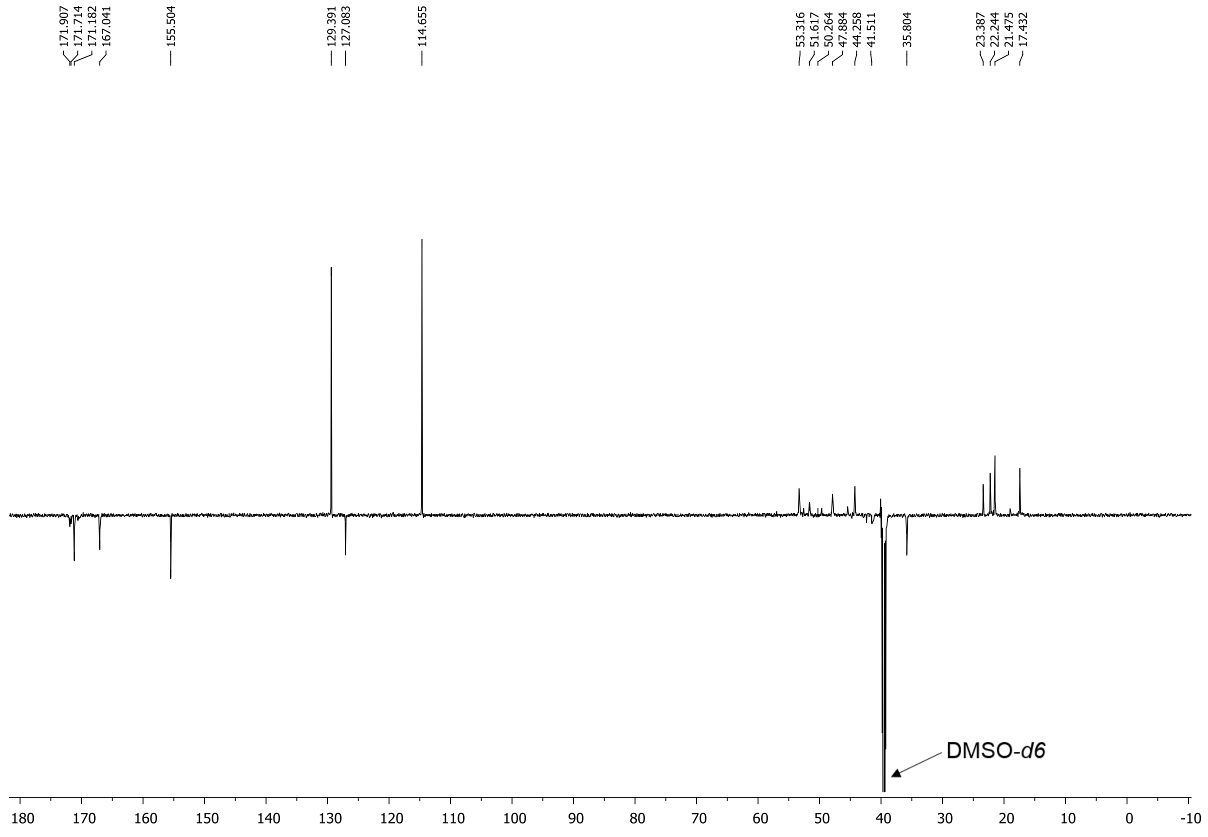

Cyclo(Leu-Ala-Sar-Ala-Tyr) (92)

^{1}H NMR (500 MHz, CD_3OD)

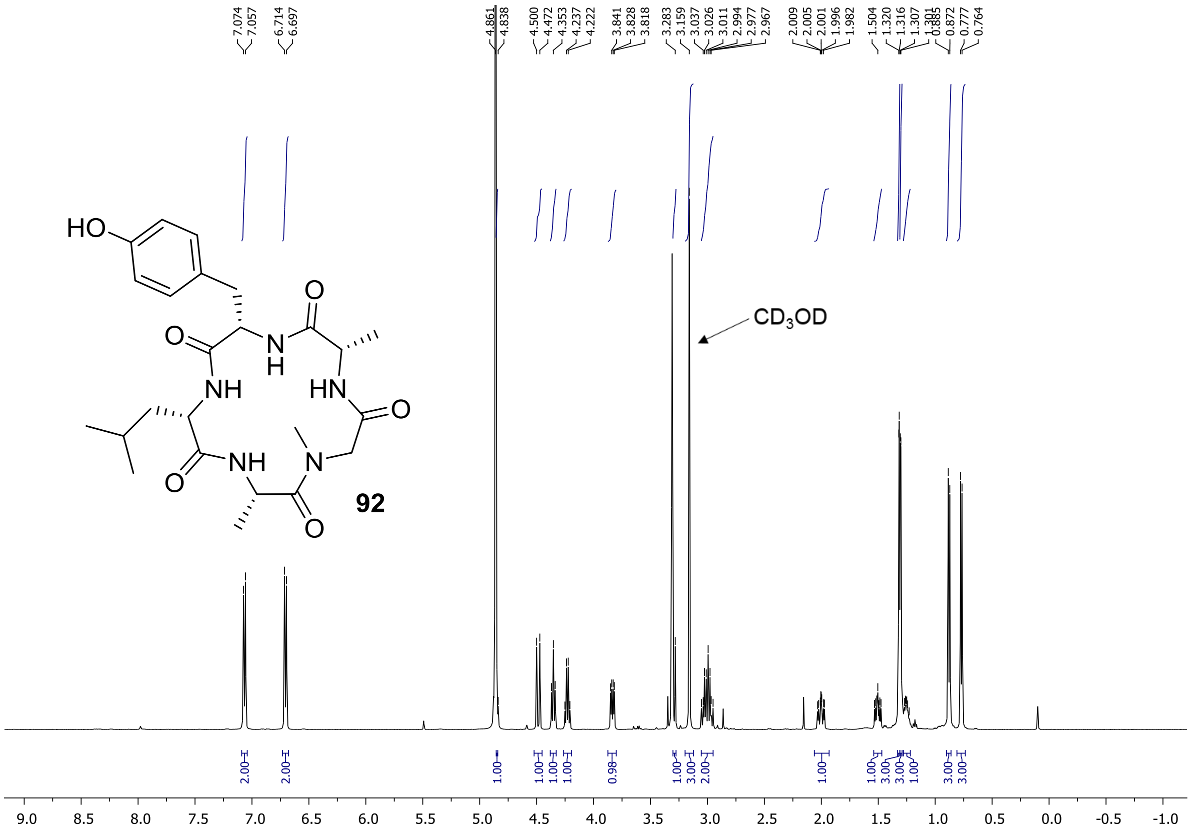

^{13}C NMR (500 MHz, CD_3OD)

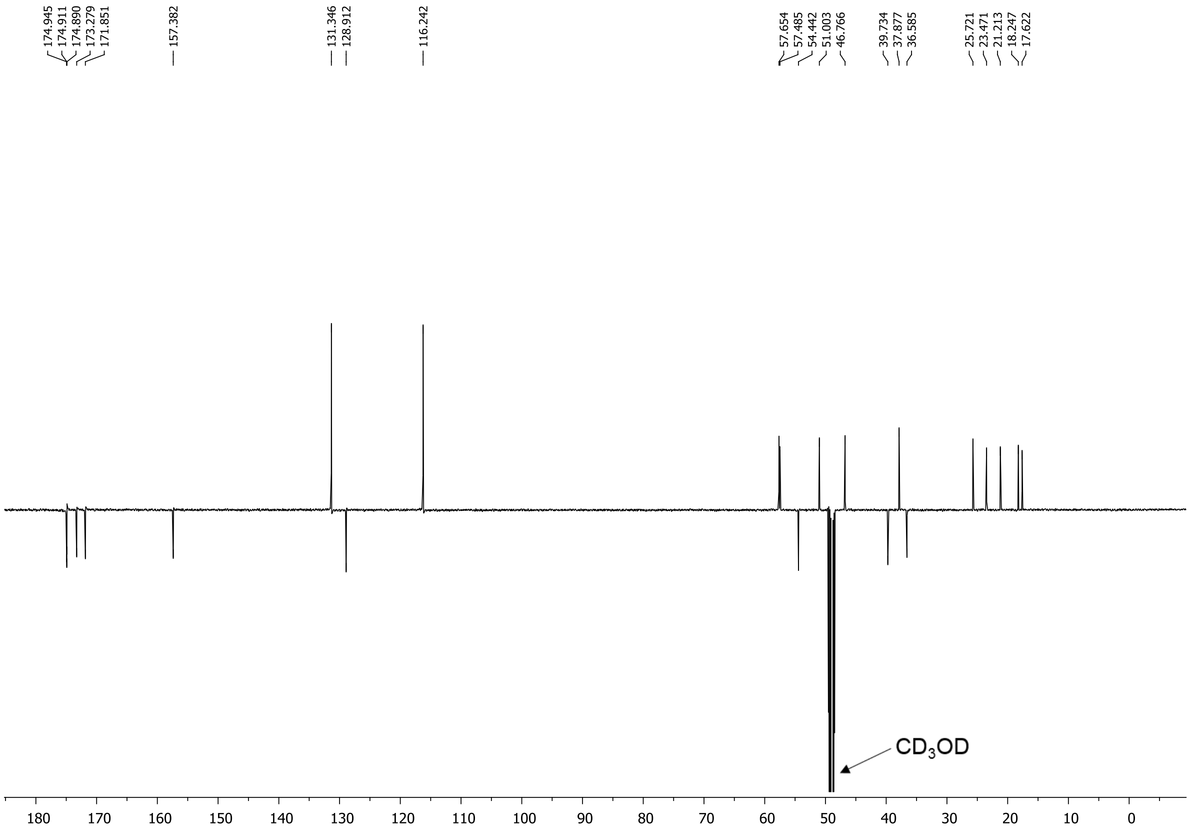

Boc-Gly-ψ[CSNH]Ala-Tyr(Bn)-OBn (93)

^{1}H NMR (400 MHz, $CDCl_3$)

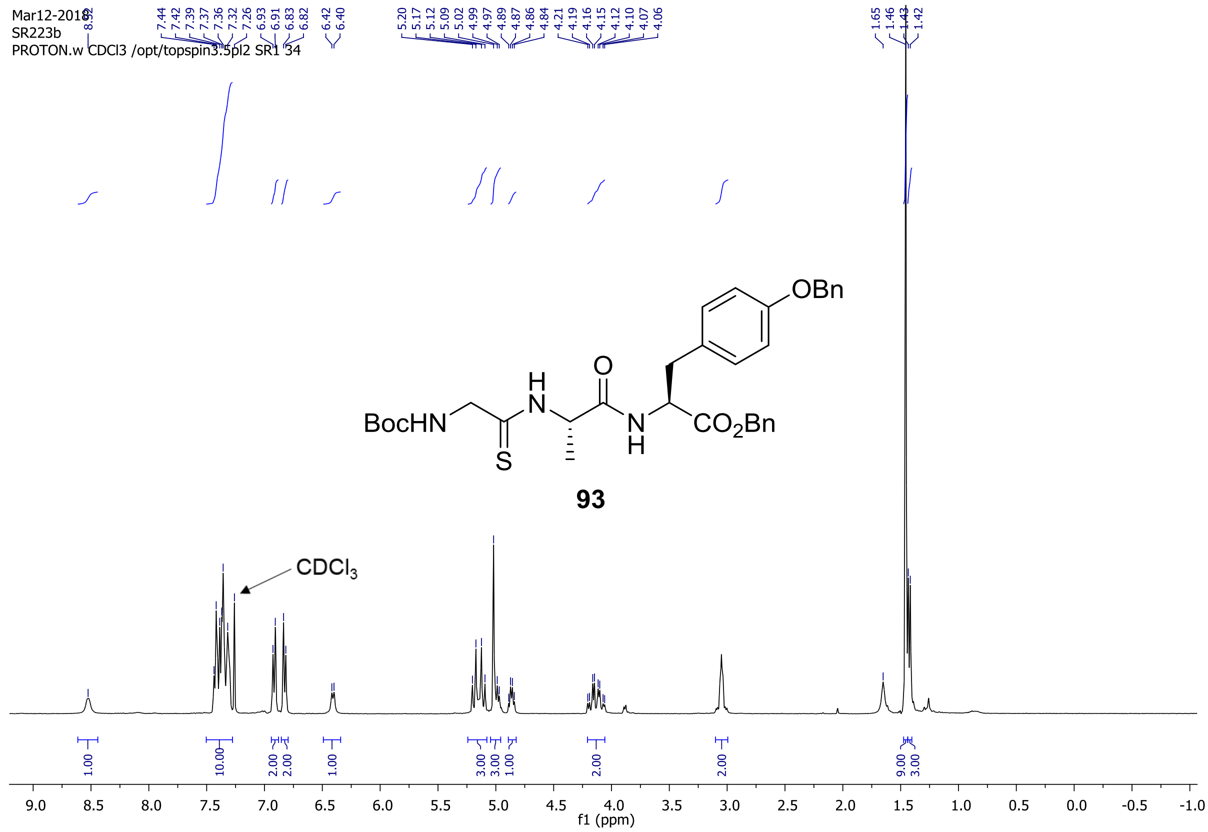

93

^{13}C NMR (101 MHz, $CDCl_3$)

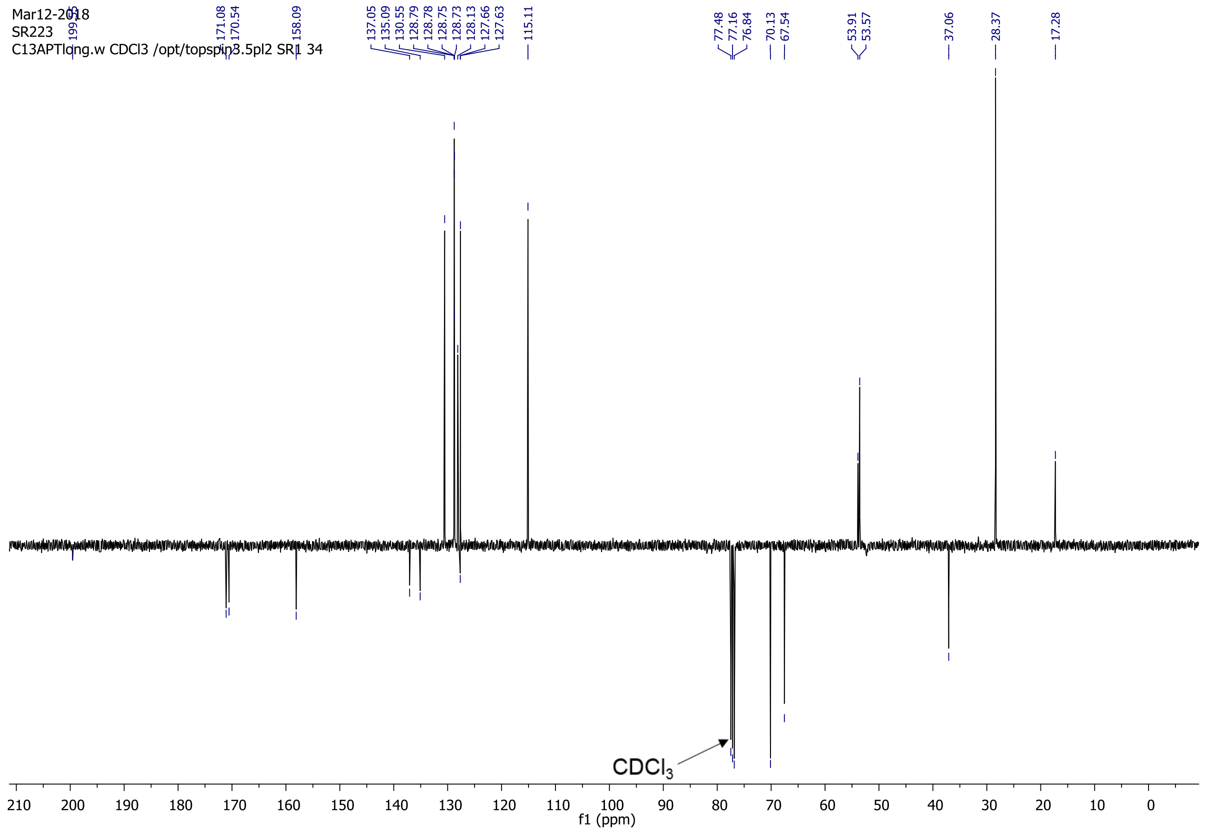

Boc-Ala-Gly-ψ[CSNH]Ala-Tyr(Bn)-OBn (94)

^{1}H NMR (400 MHz, $CDCl_3$)

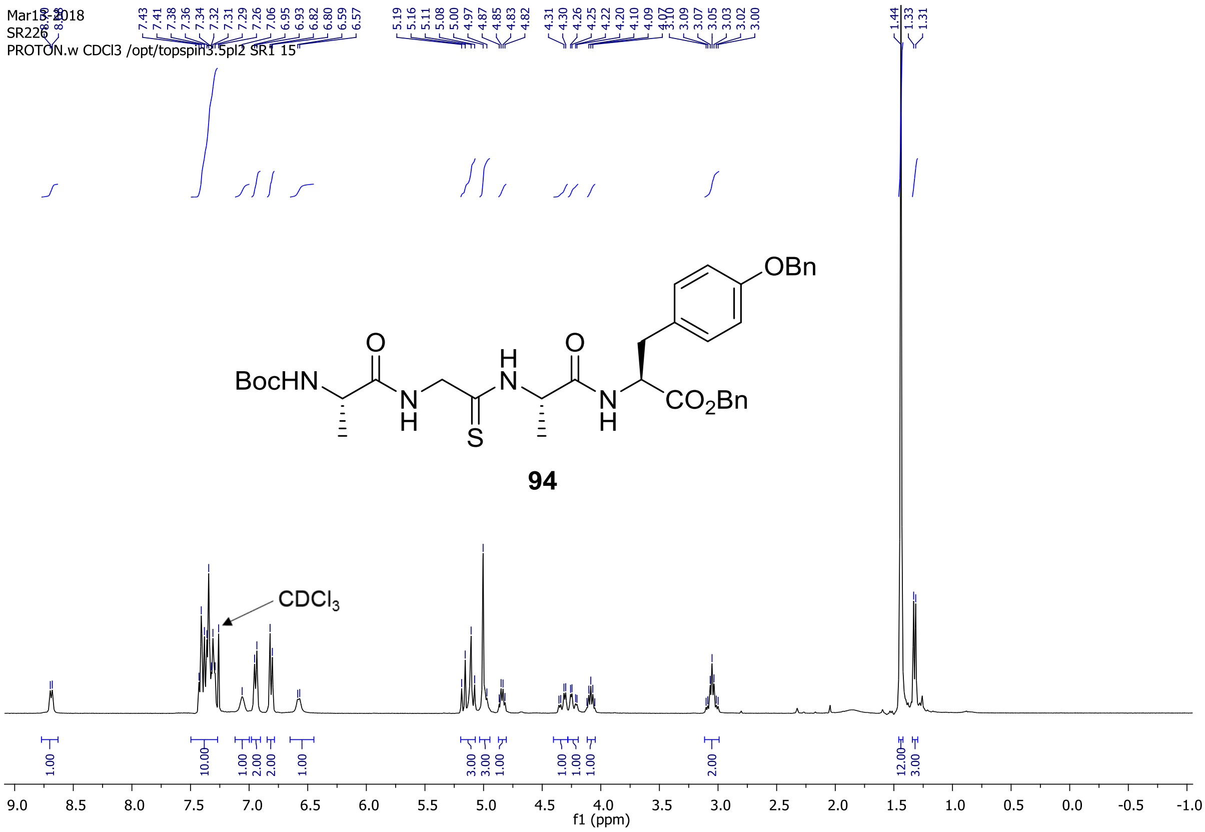

^{13}C NMR (101 MHz, $CDCl_3$)

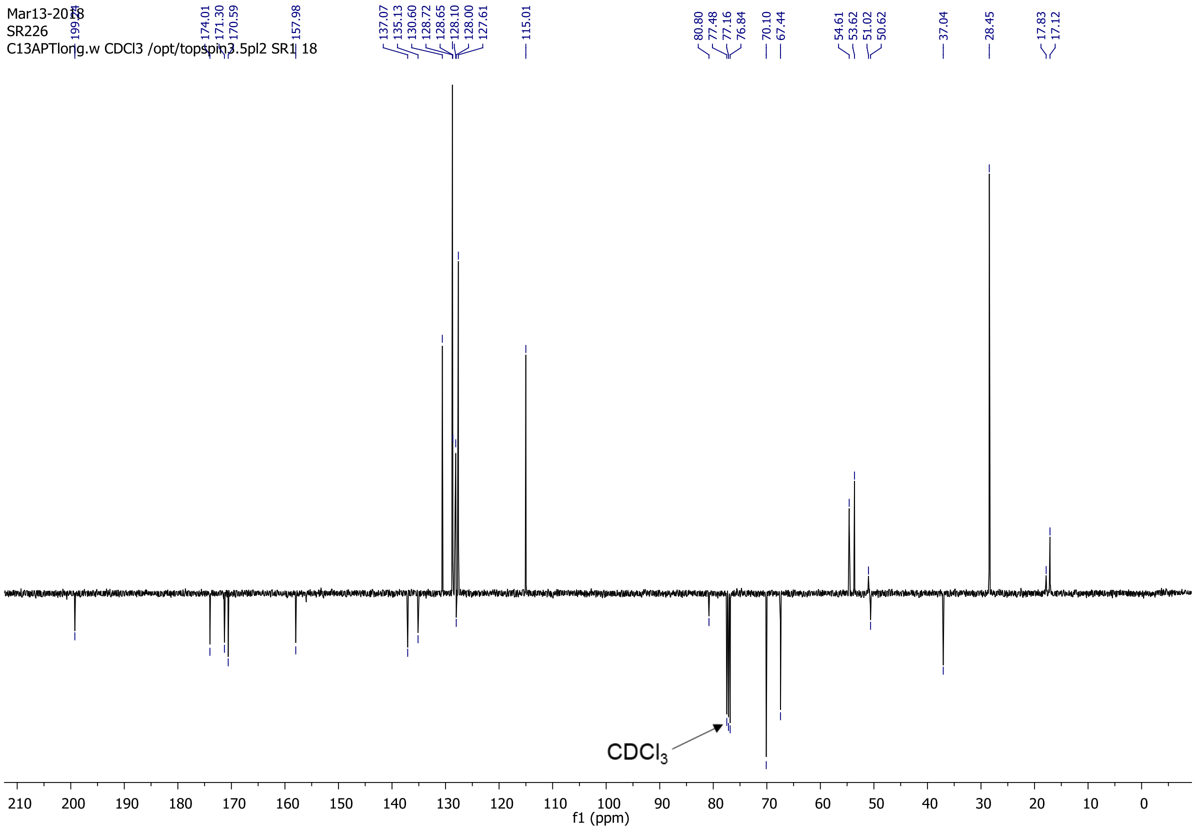

Cbz-Leu-Ala-Gly-ψ[CSNH]Ala-Tyr(Bn)-OBn (95)

^{1}H NMR (400 MHz, $CDCl_3$)

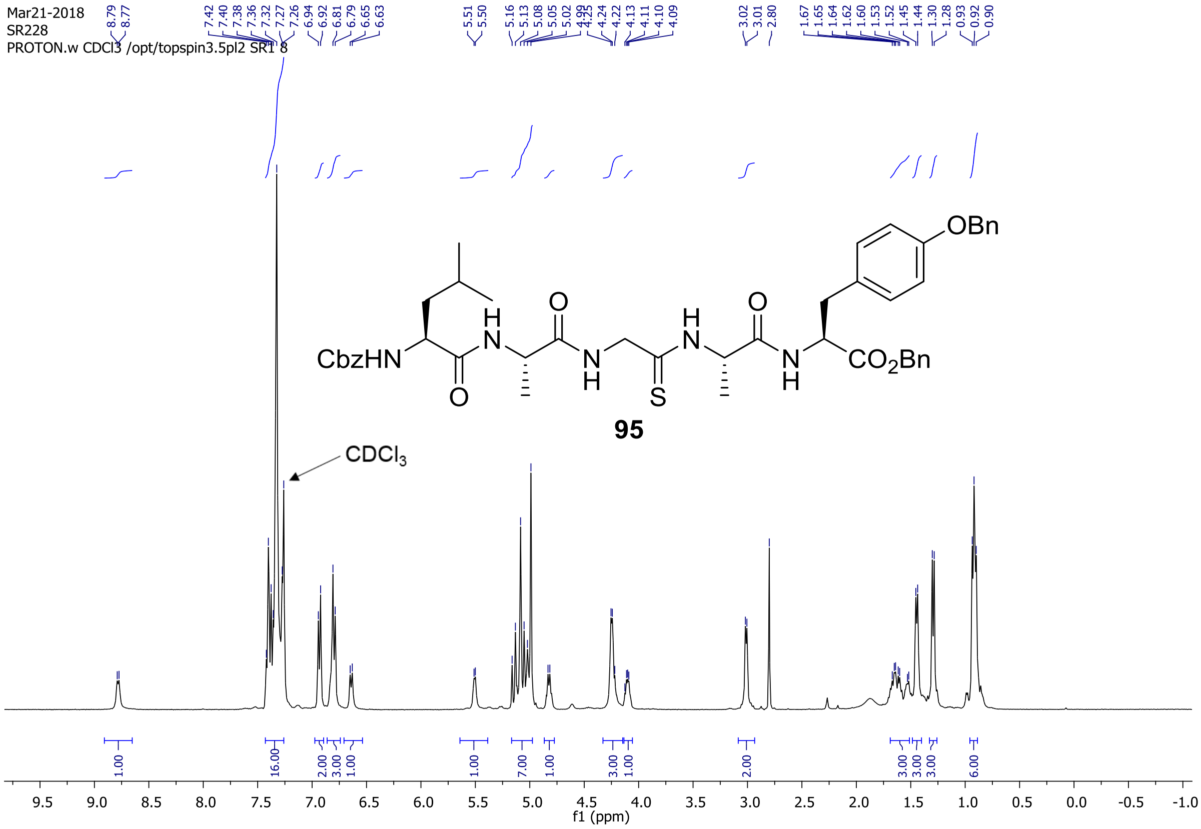

^{13}C NMR (101 MHz, $CDCl_3$)

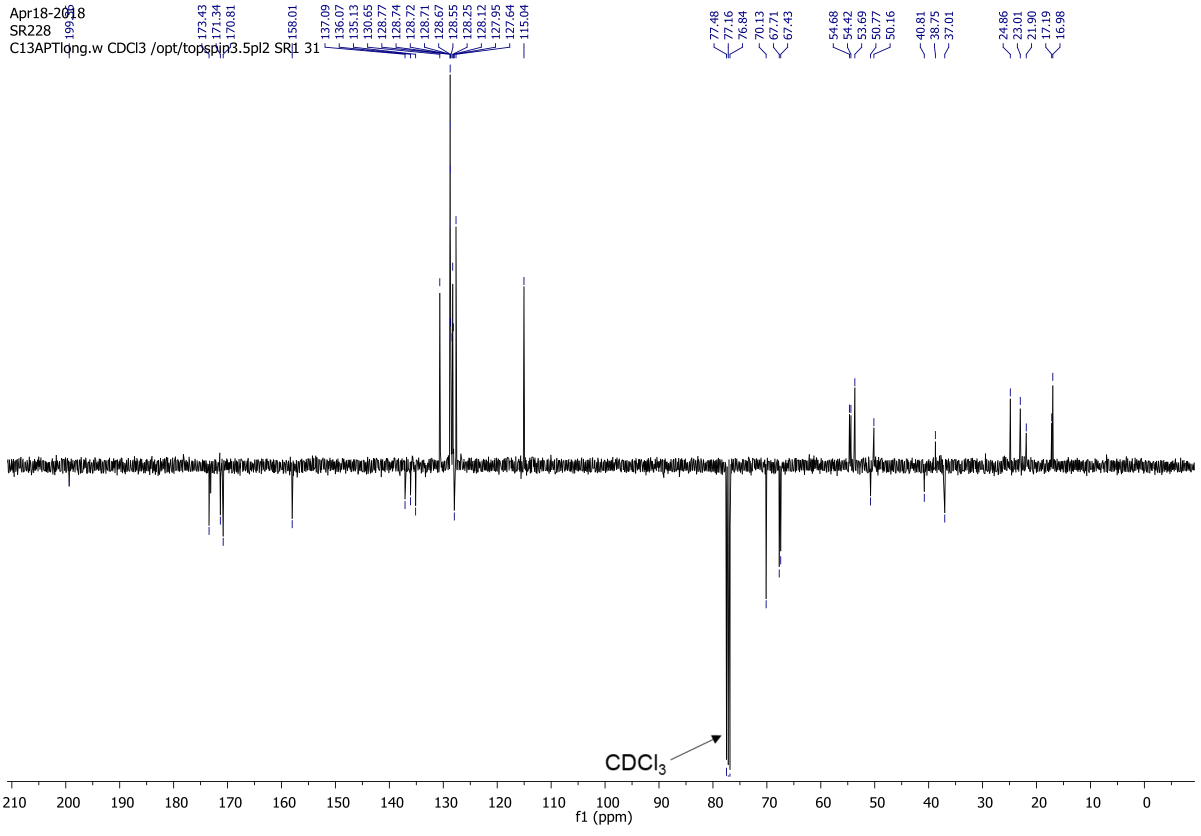

Cbz-Leu-Ala-NHCH$_2$CH$_2$-Ala-Tyr(Bn)-OBn (96)

^{1}H NMR (400 MHz, CD$_3$OD)

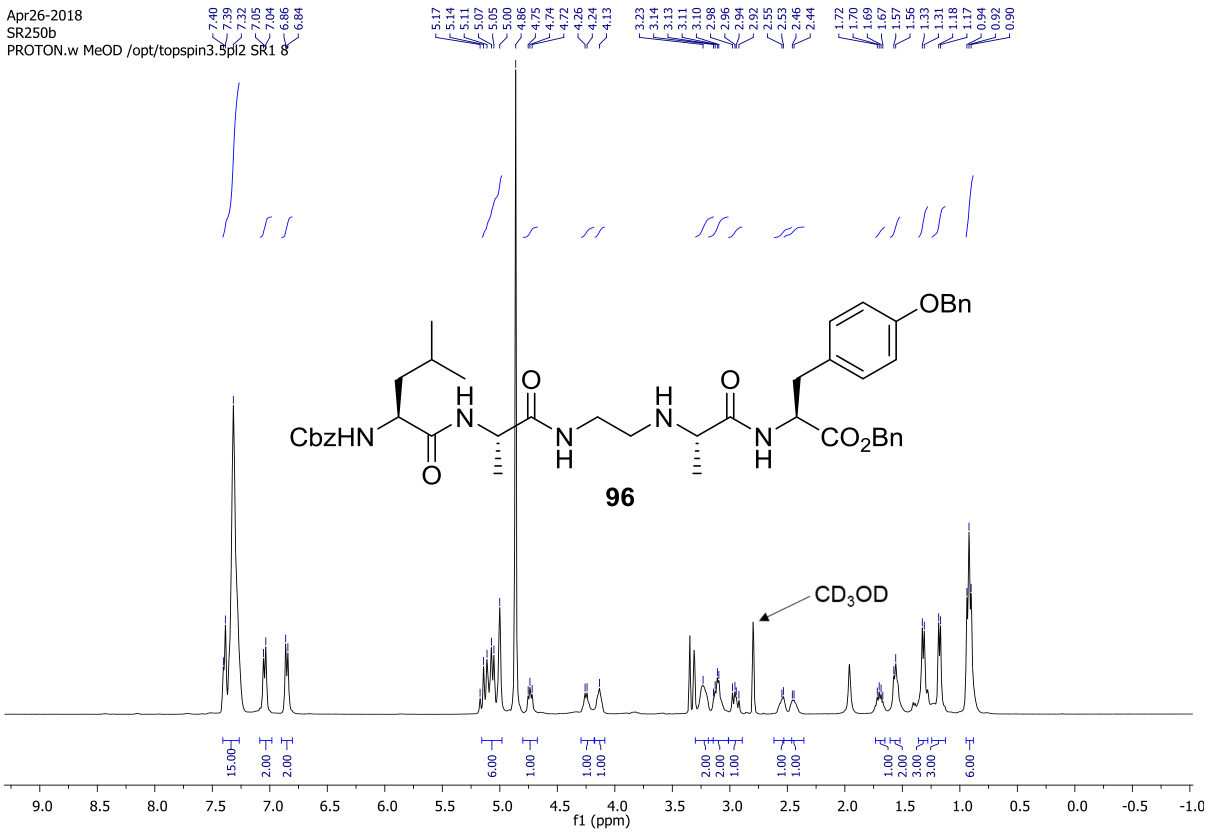

^{13}C NMR (101 MHz, CD$_3$OD)

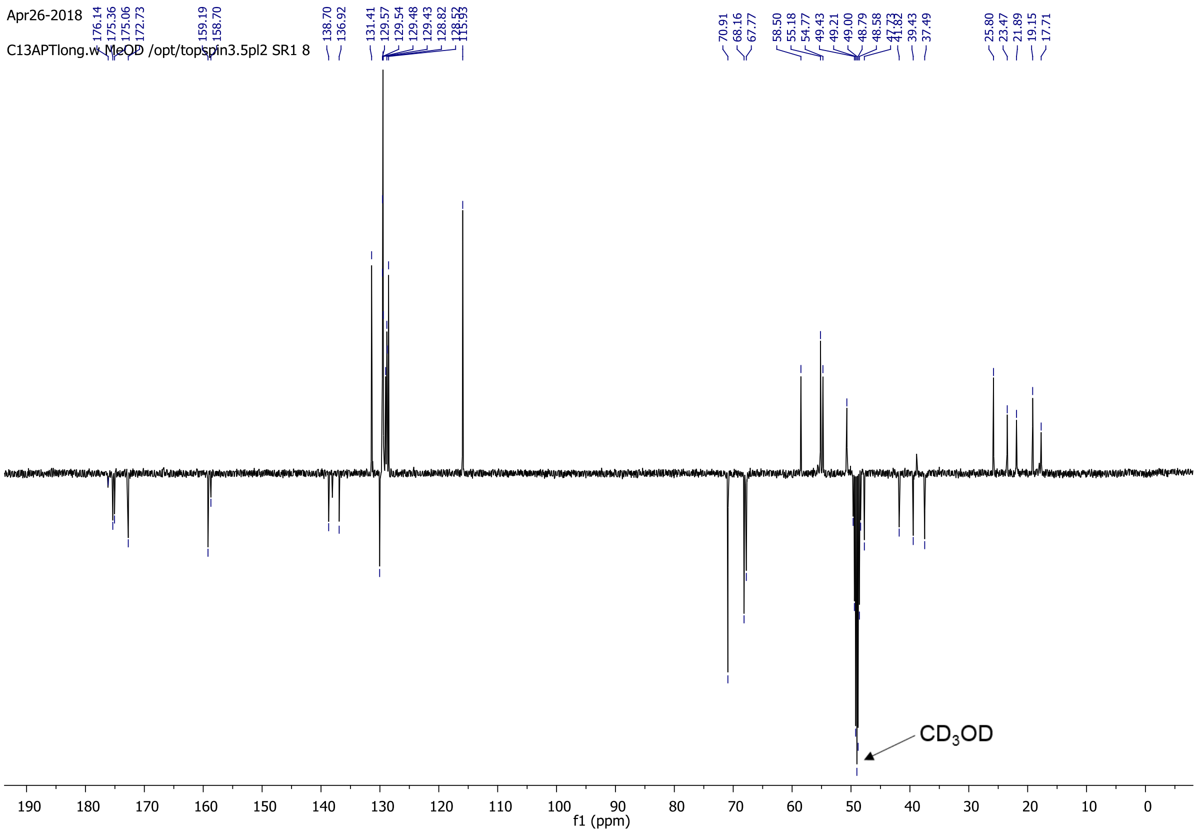

H-Leu-Ala-NHCH$_2$CH$_2$-Ala-Tyr-OH (97)

^{1}H NMR (400 MHz, CD_3OD)

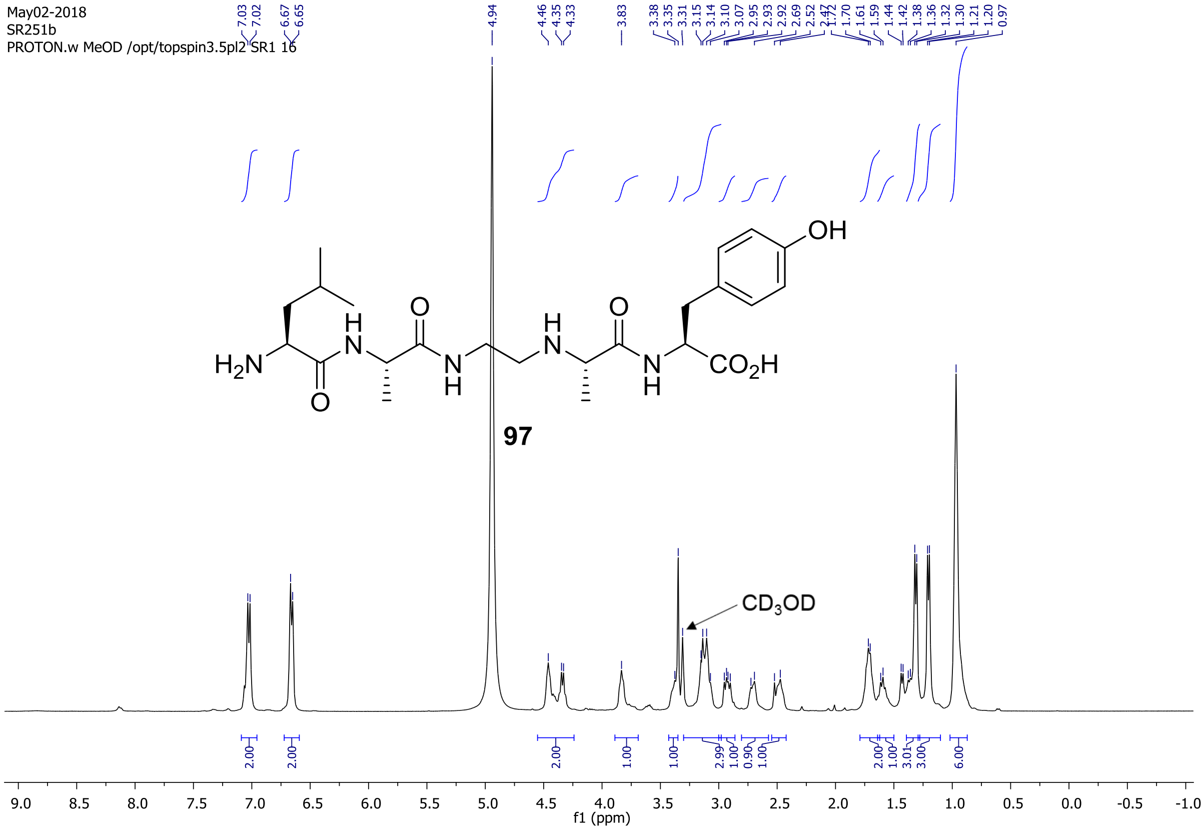

^{13}C NMR (101 MHz, CD_3OD)

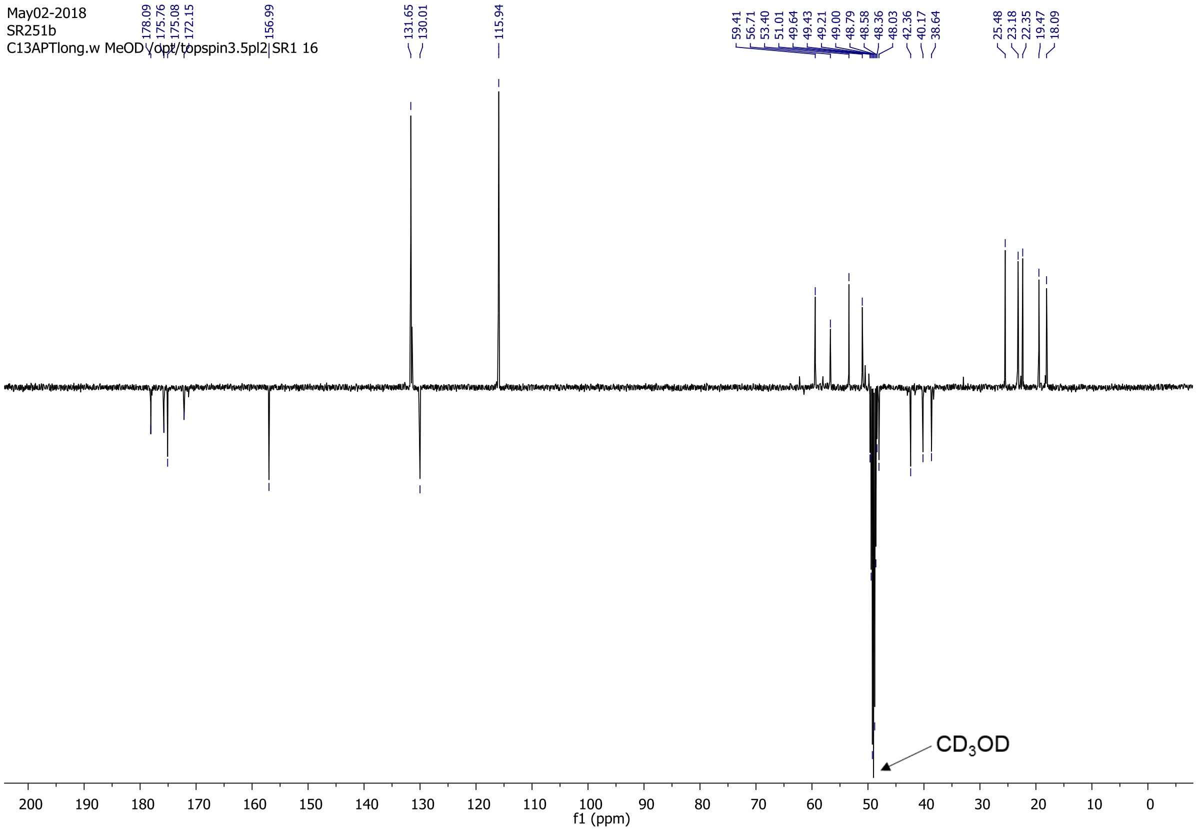

Cyclo(Leu-Ala-NHCH$_2$CH$_2$-Ala-Tyr) · TFA (98)

^{1}H NMR (500 MHz, CD$_3$OD)

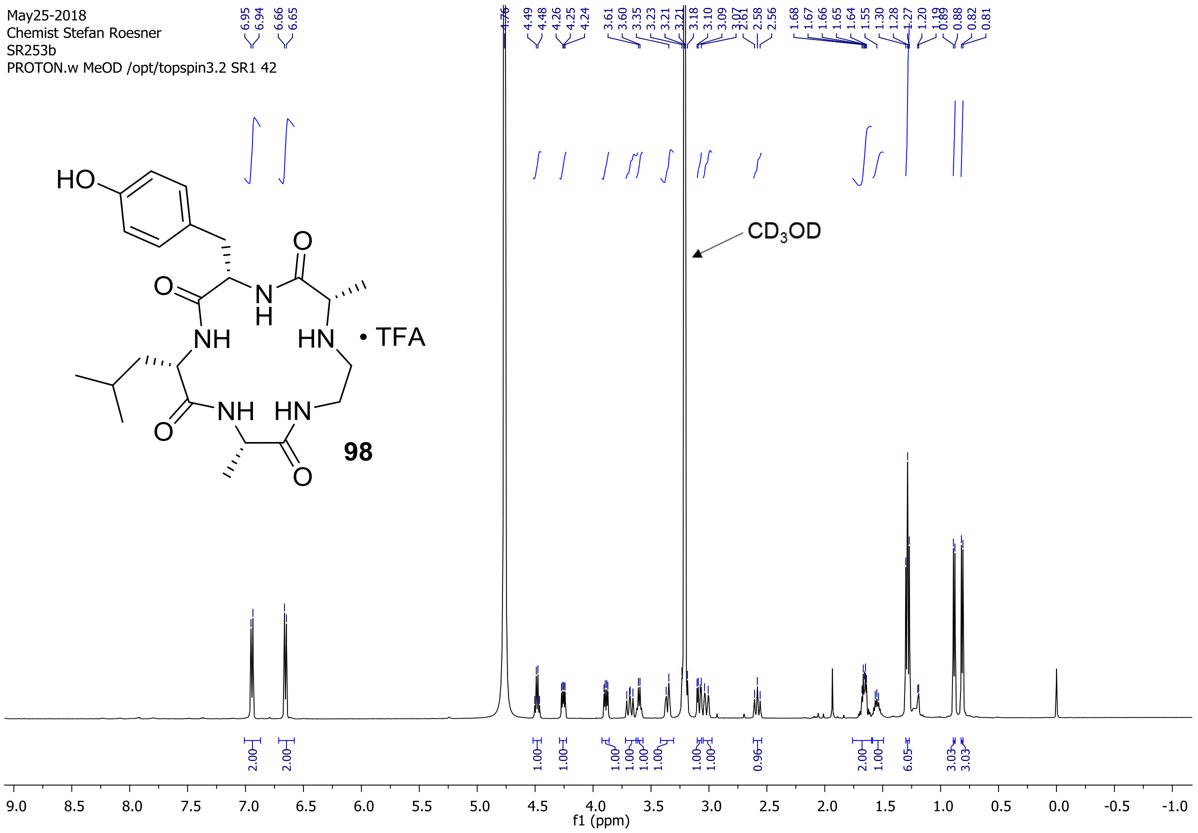

^{13}C NMR (126 MHz, CD$_3$OD)

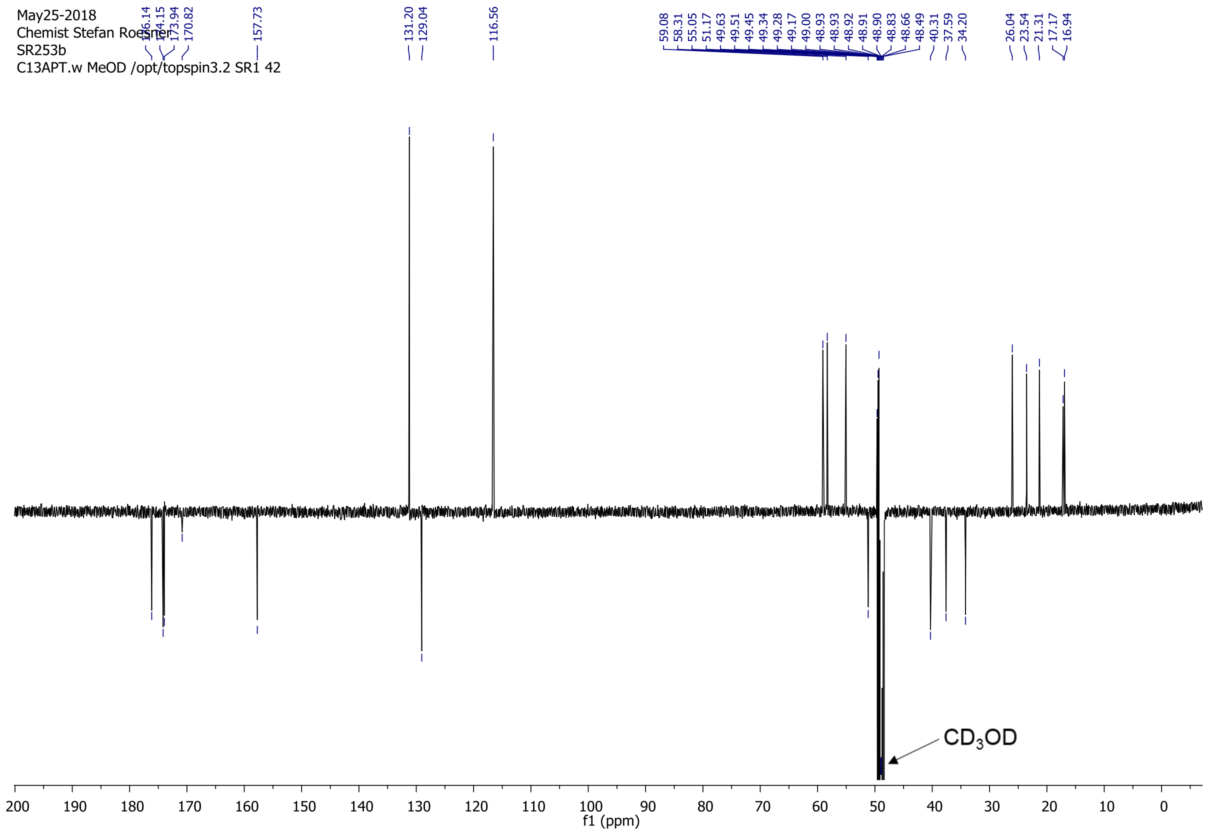

Boc-Ala-NHCH₂CH(Me)₂-Ala-Tyr(Bn)-OBn (99)

^{1}H NMR (500 MHz, CDCl$_3$)

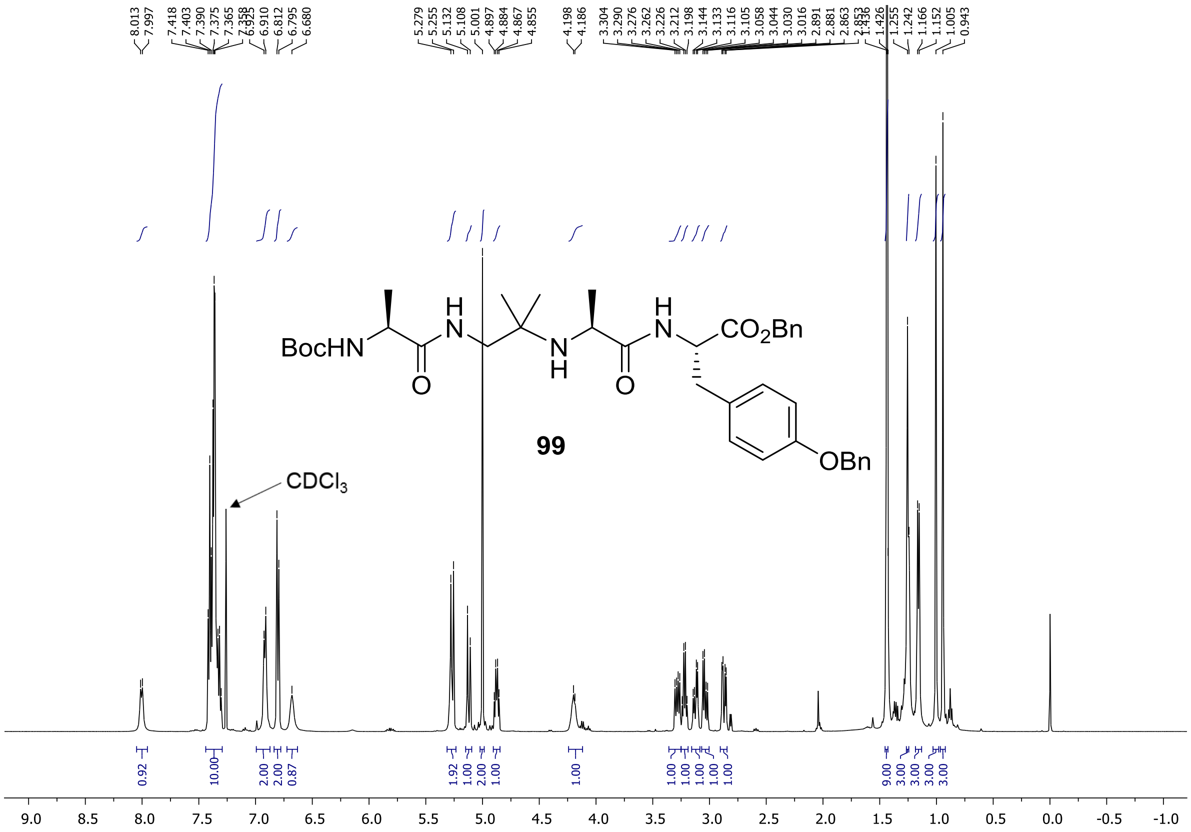

^{13}C NMR (126 MHz, CDCl$_3$)

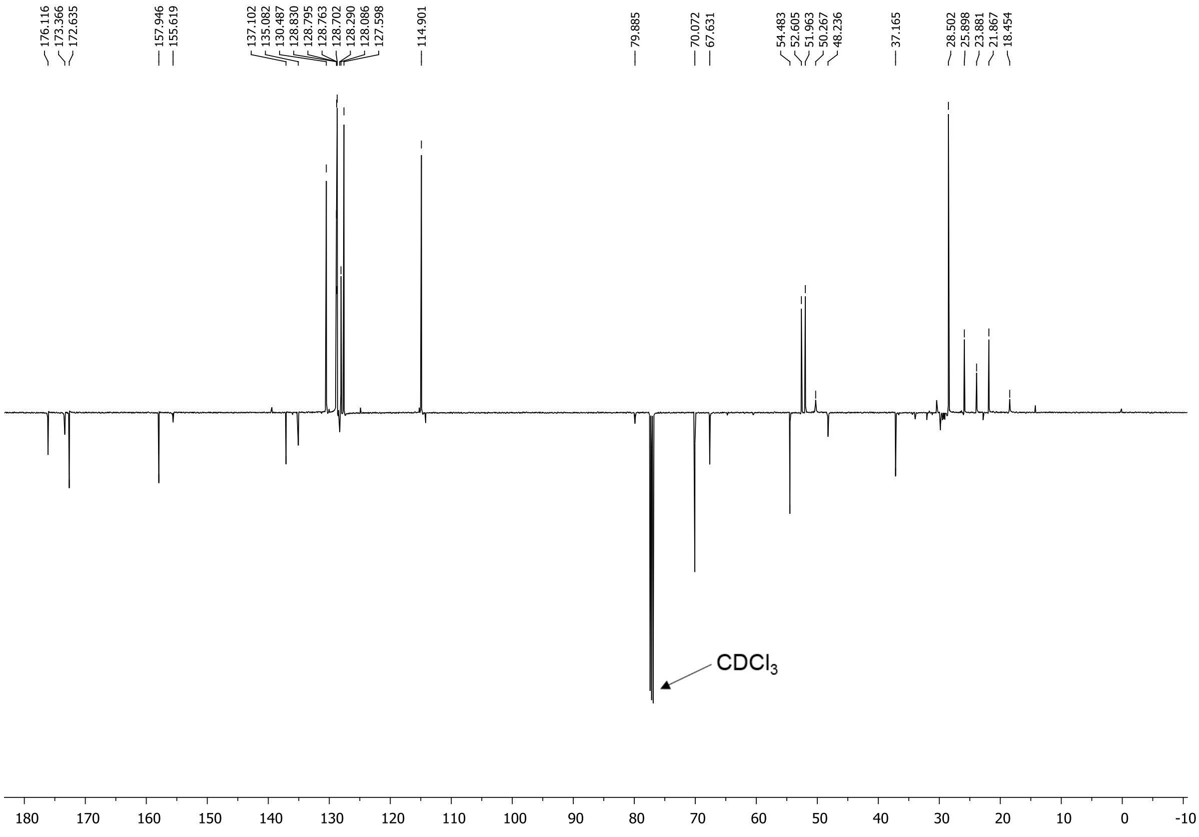

Cbz-Leu-Ala-NHCH$_2$CH(Me)$_2$-Ala-Tyr(Bn)-OBn (100)

^{1}H NMR (500 MHz, CDCl$_3$)

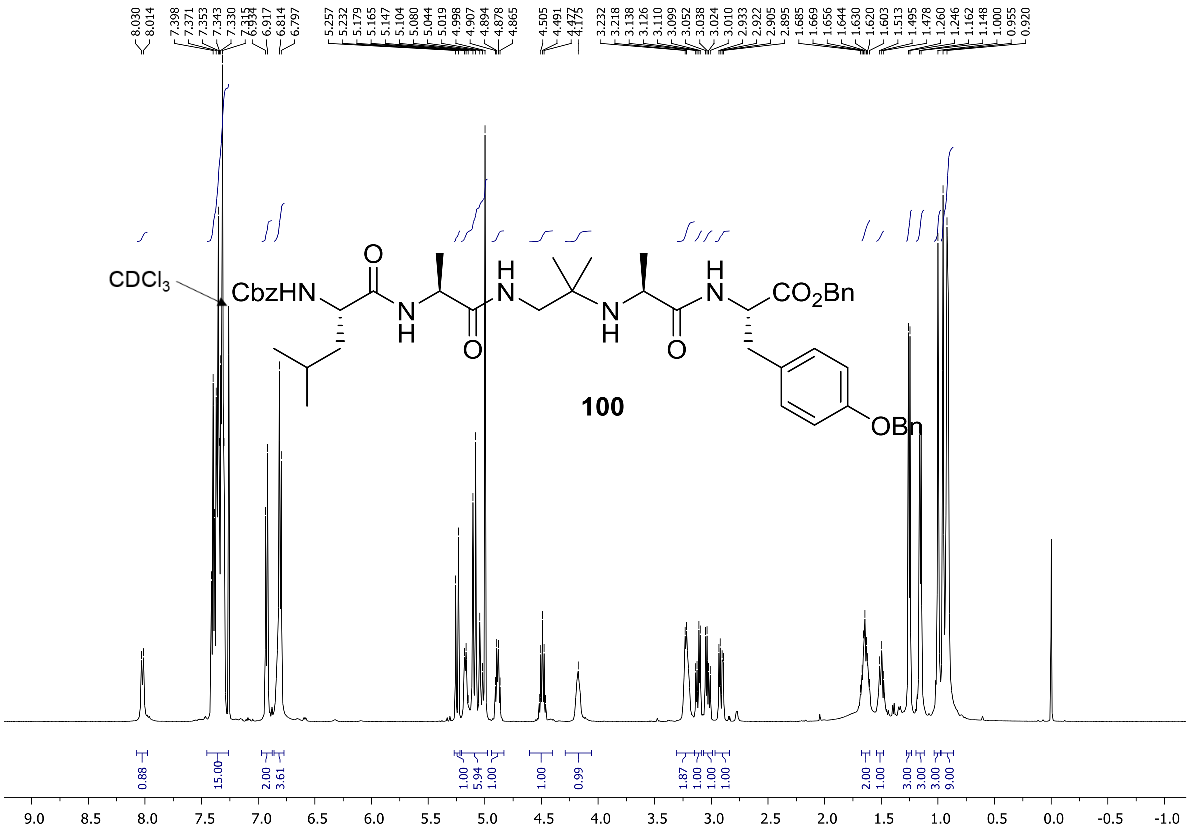

^{13}C NMR (126 MHz, CDCl$_3$)

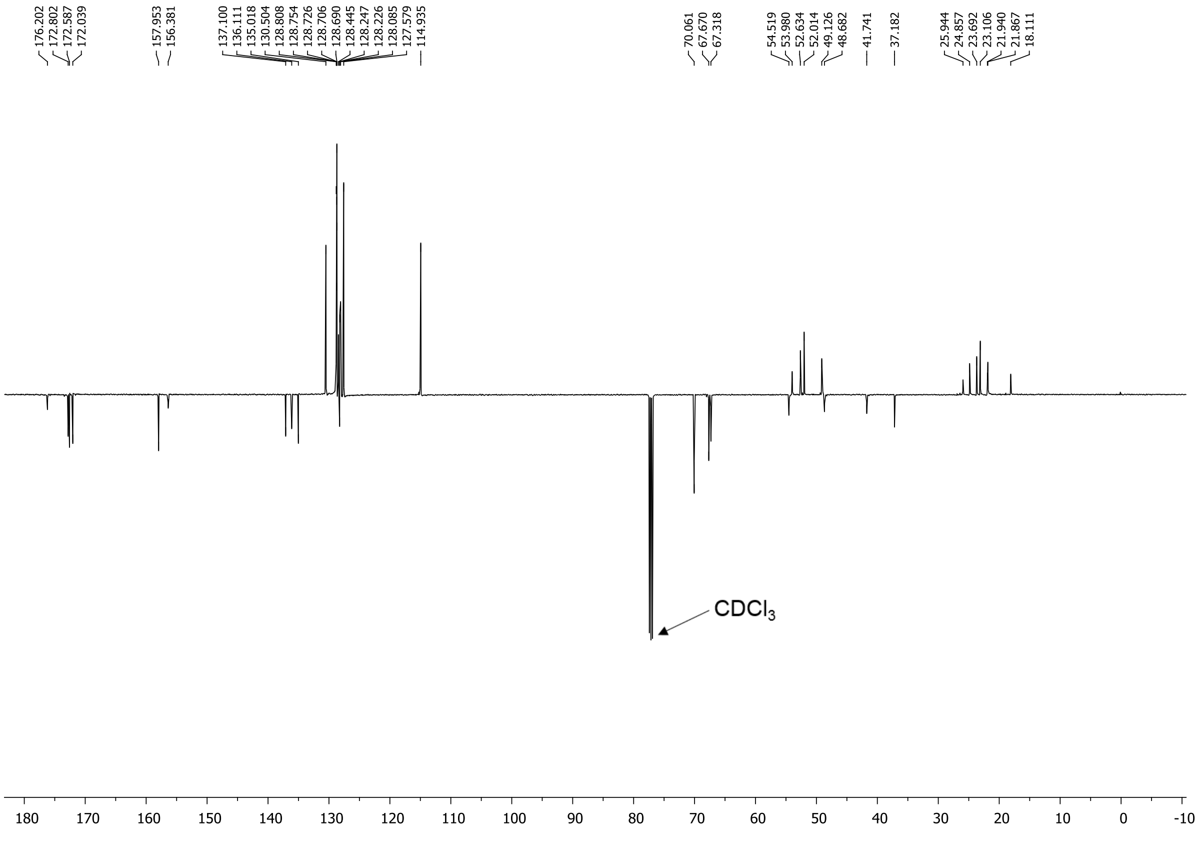

H-Leu-Ala-NHCH$_2$CH(Me)$_2$-Ala-Tyr-OH (101)

^{1}H NMR (500 MHz, CD_3OD)

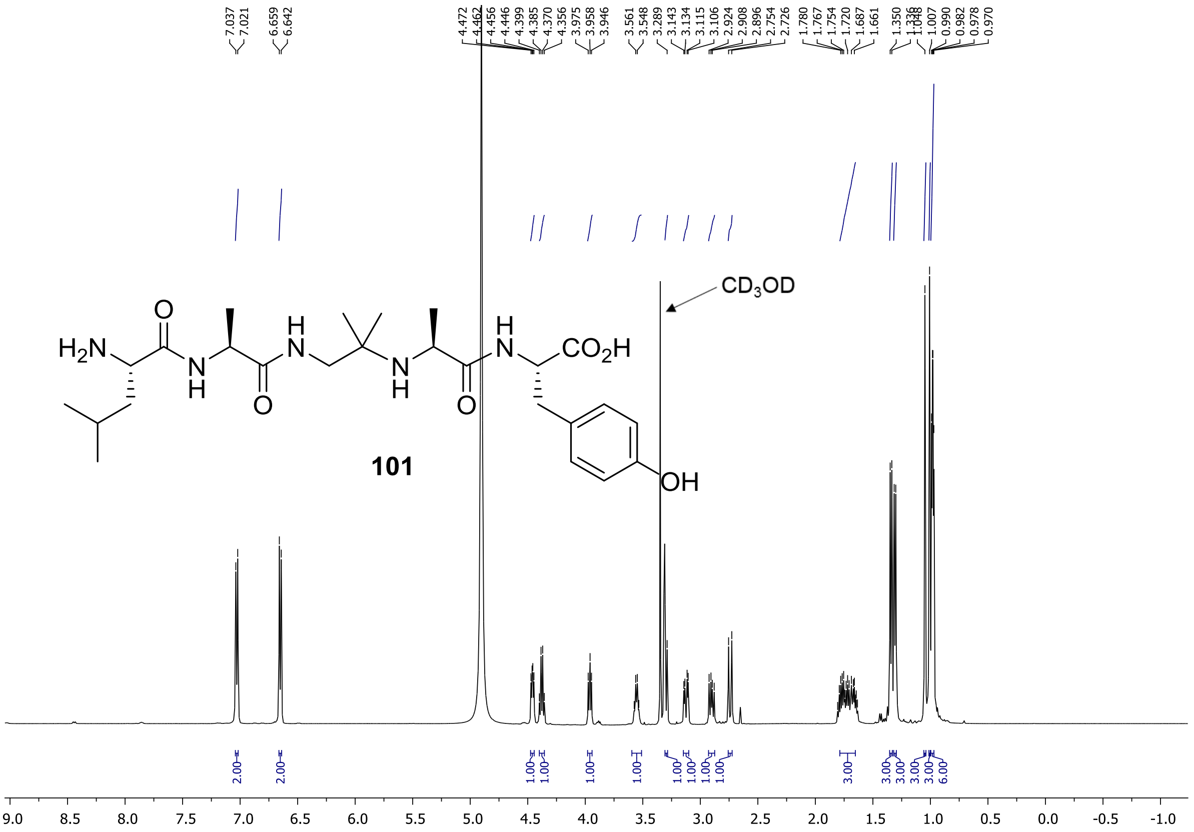

^{13}C NMR (126 MHz, CD_3OD)

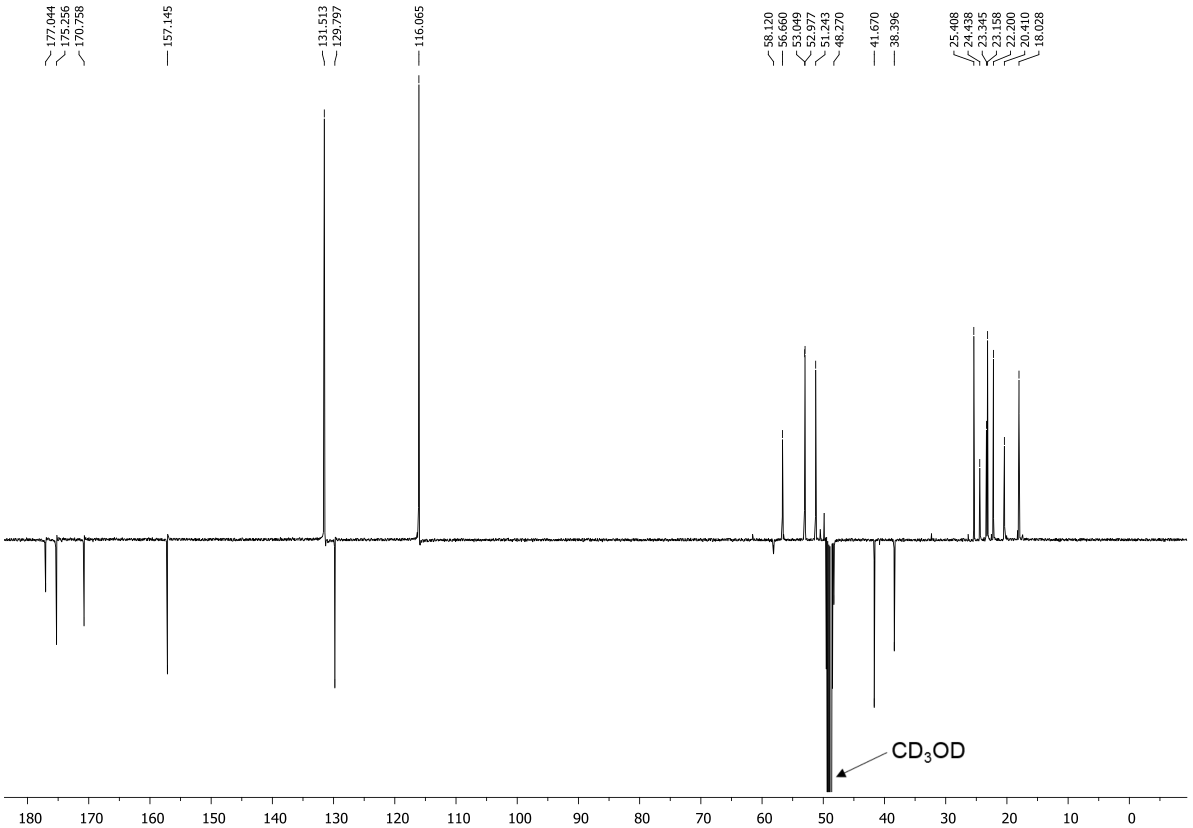

Cyclo(Leu-Ala-NHCH$_2$CH(Me)$_2$-Ala-Tyr) (102)

^{1}H NMR (500 MHz, CD$_3$OD)

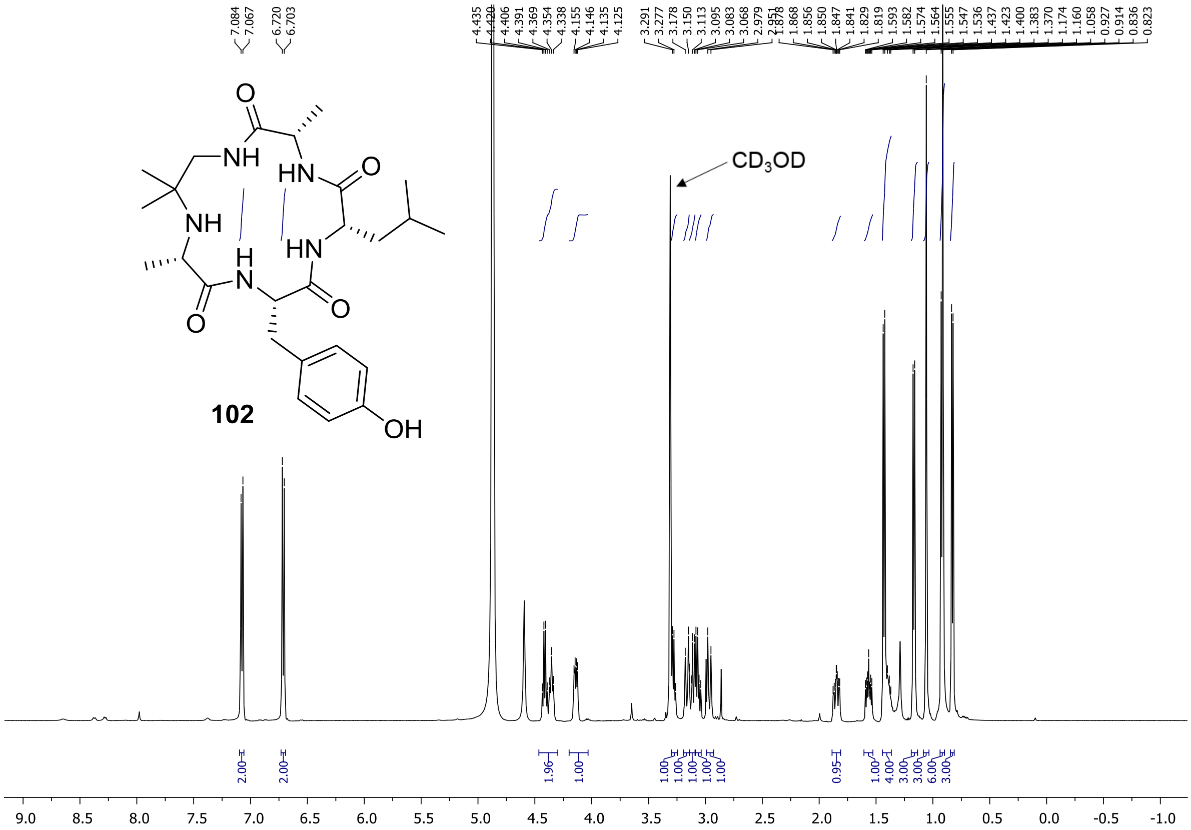

^{13}C NMR (126 MHz, CD$_3$OD)

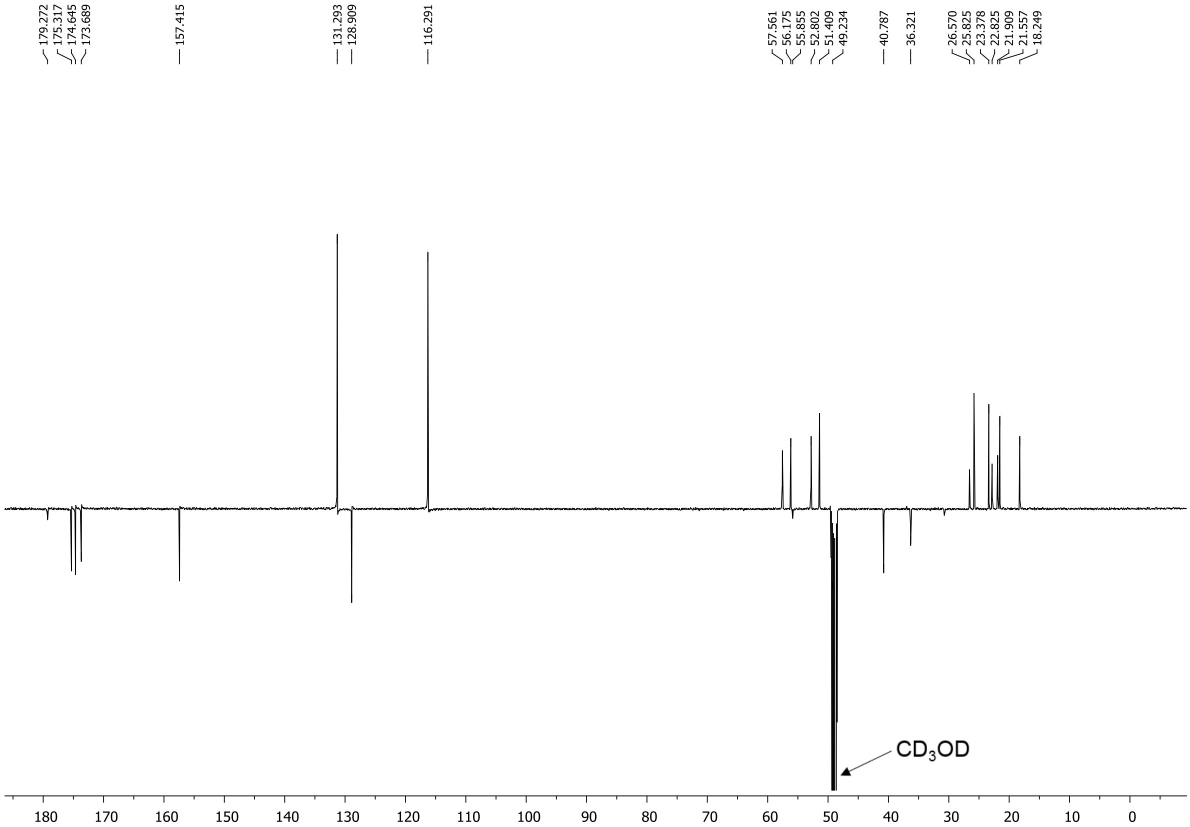

Boc-βAla-Ala-Tyr(Bn)-OBn (103)

^{1}H NMR (500 MHz, DMSO-*d6*)

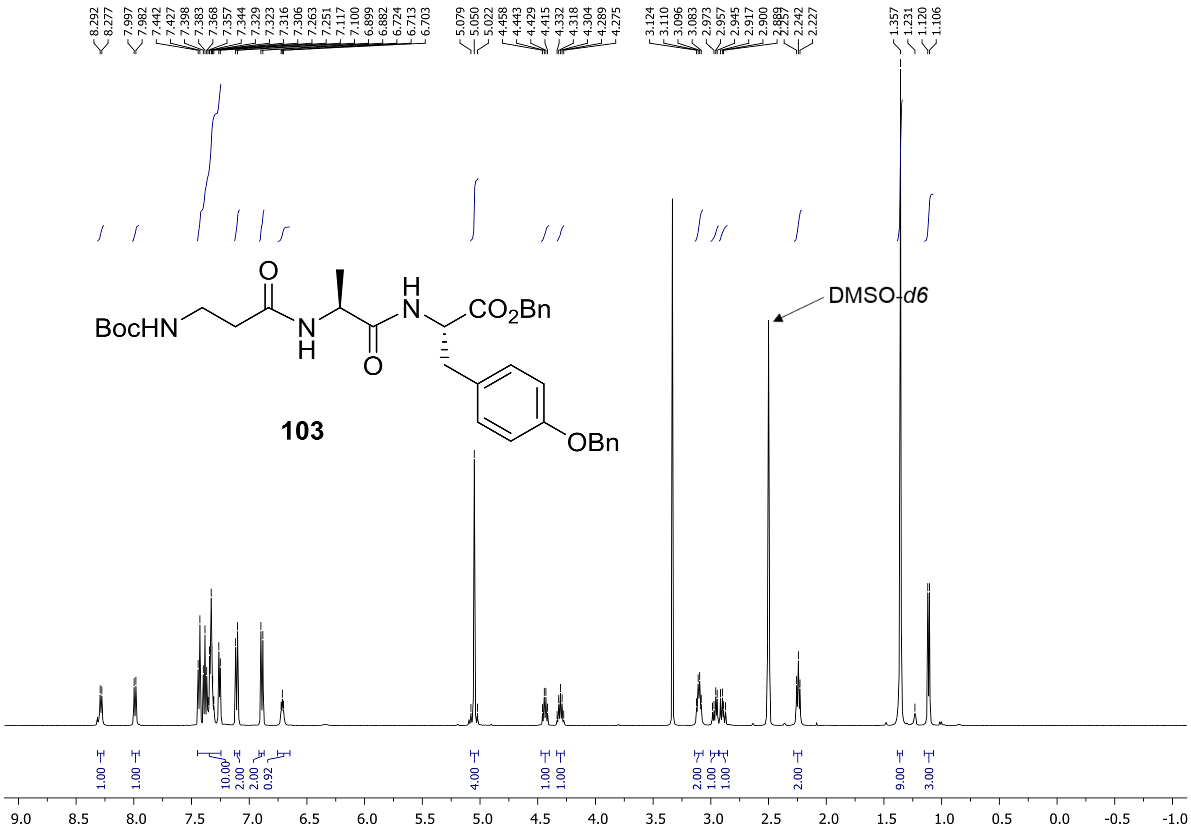

^{13}C NMR (126 MHz, DMSO-*d6*)

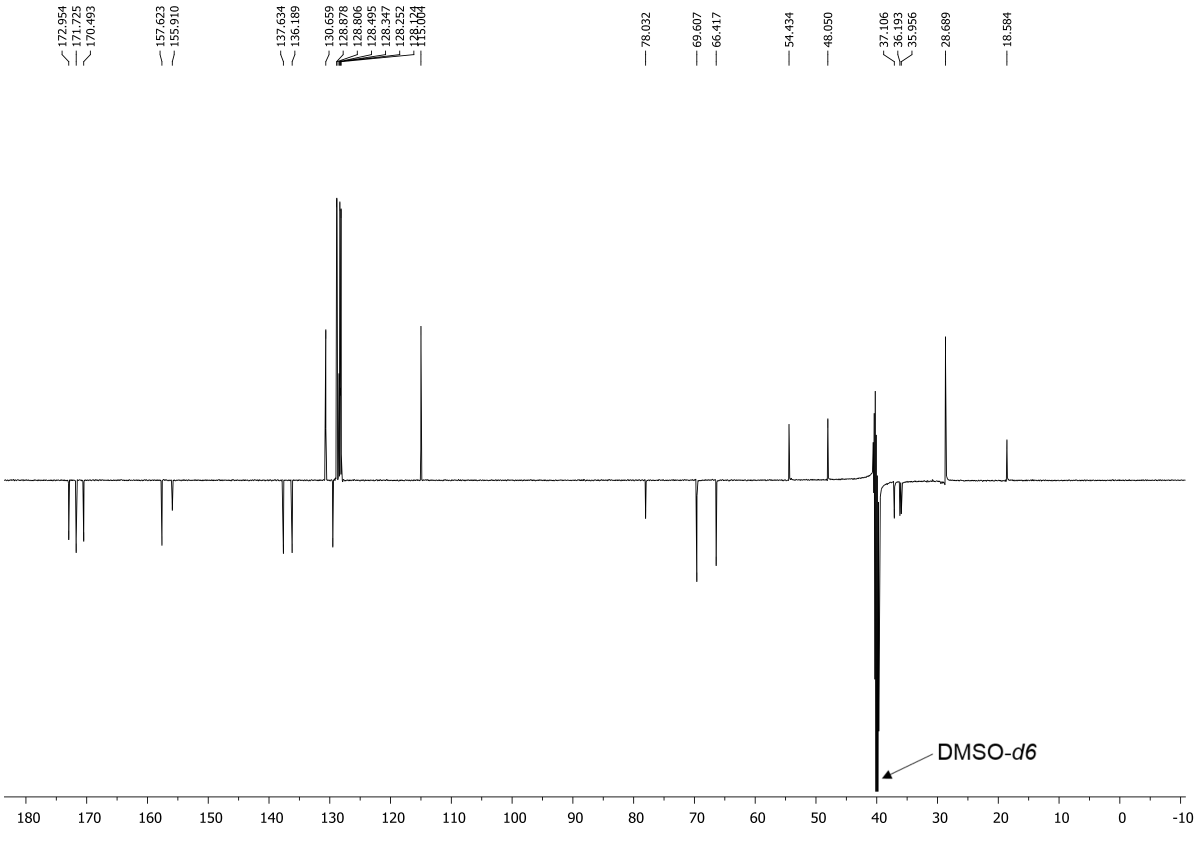

Boc-Ala-βAla-Ala-Tyr(Bn)-OBn (104)

^{1}H NMR (500 MHz, CD_2Cl_2)

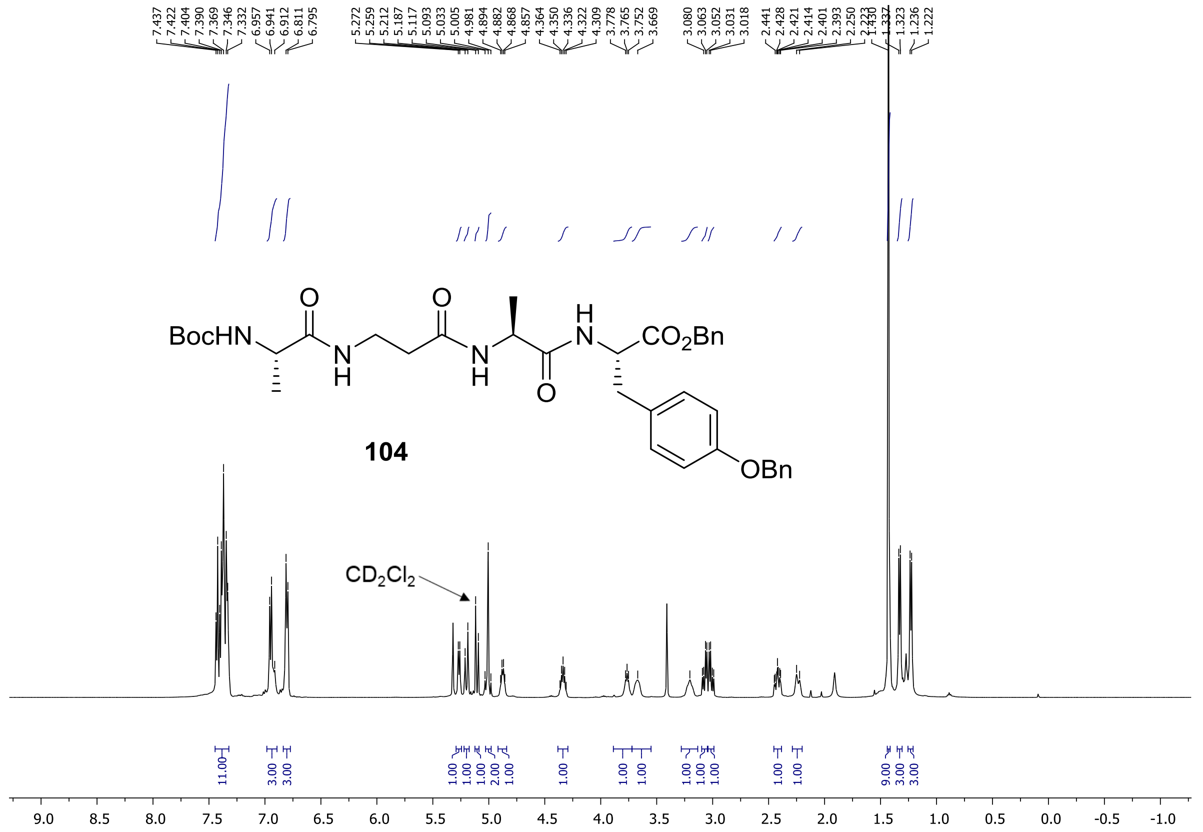

^{13}C NMR (126 MHz, CD_2Cl_2)

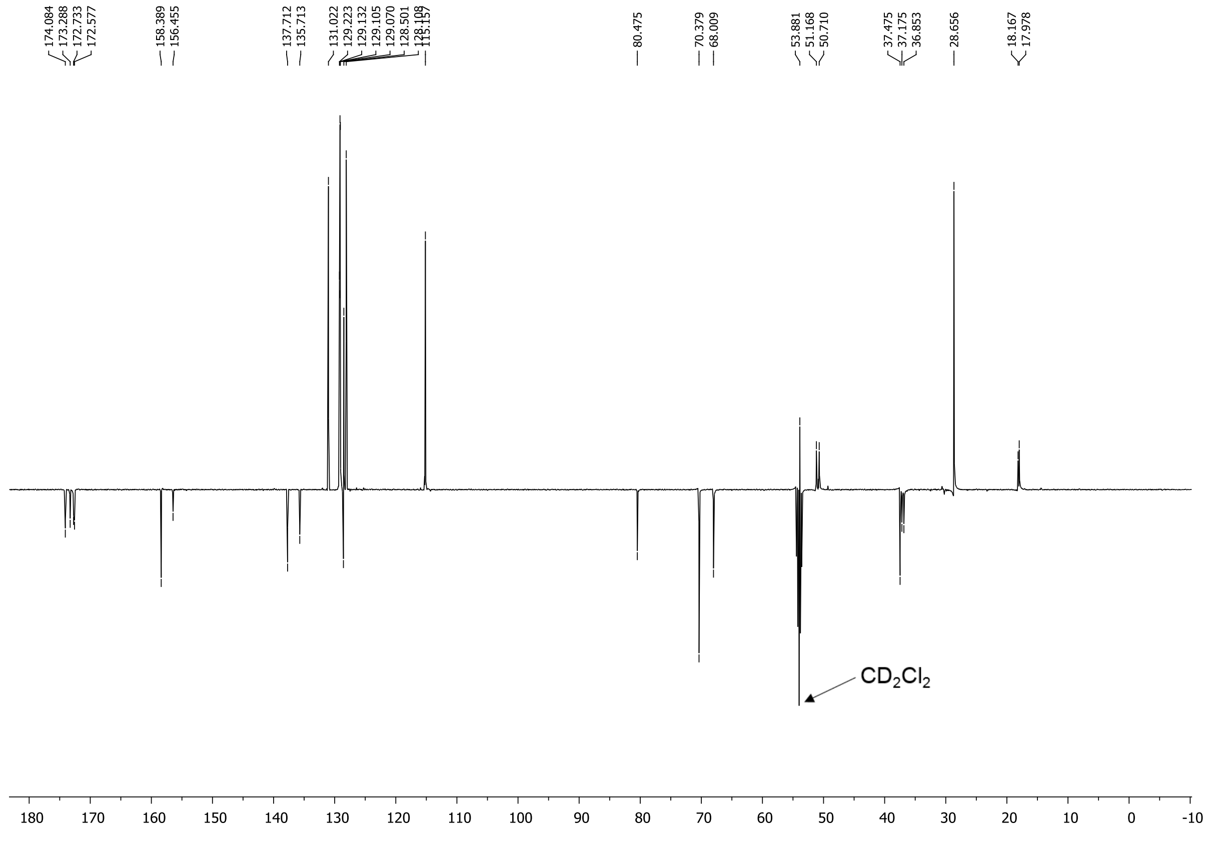

Cbz-Leu-Ala-βAla-Ala-Tyr(Bn)-OBn (105)

^{1}H NMR (500 MHz, DMSO-*d6*)

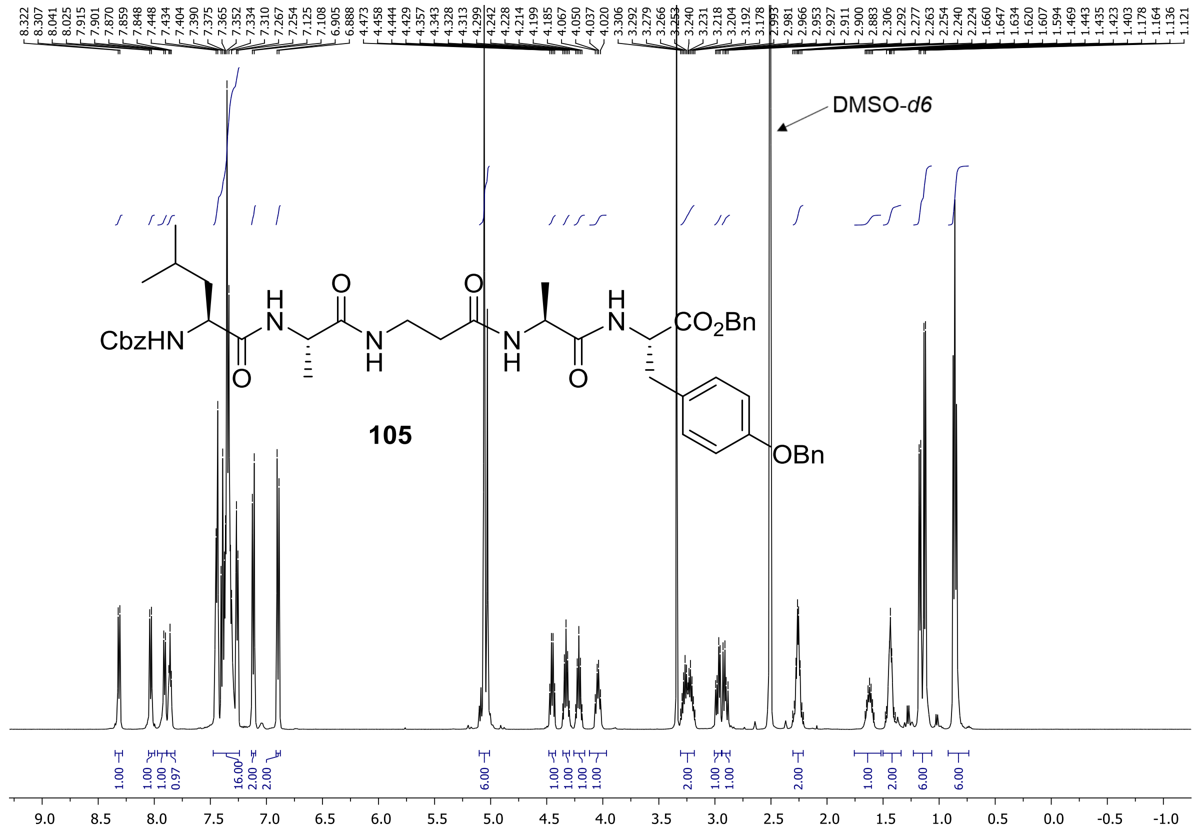

^{13}C NMR (126 MHz, DMSO-*d6*)

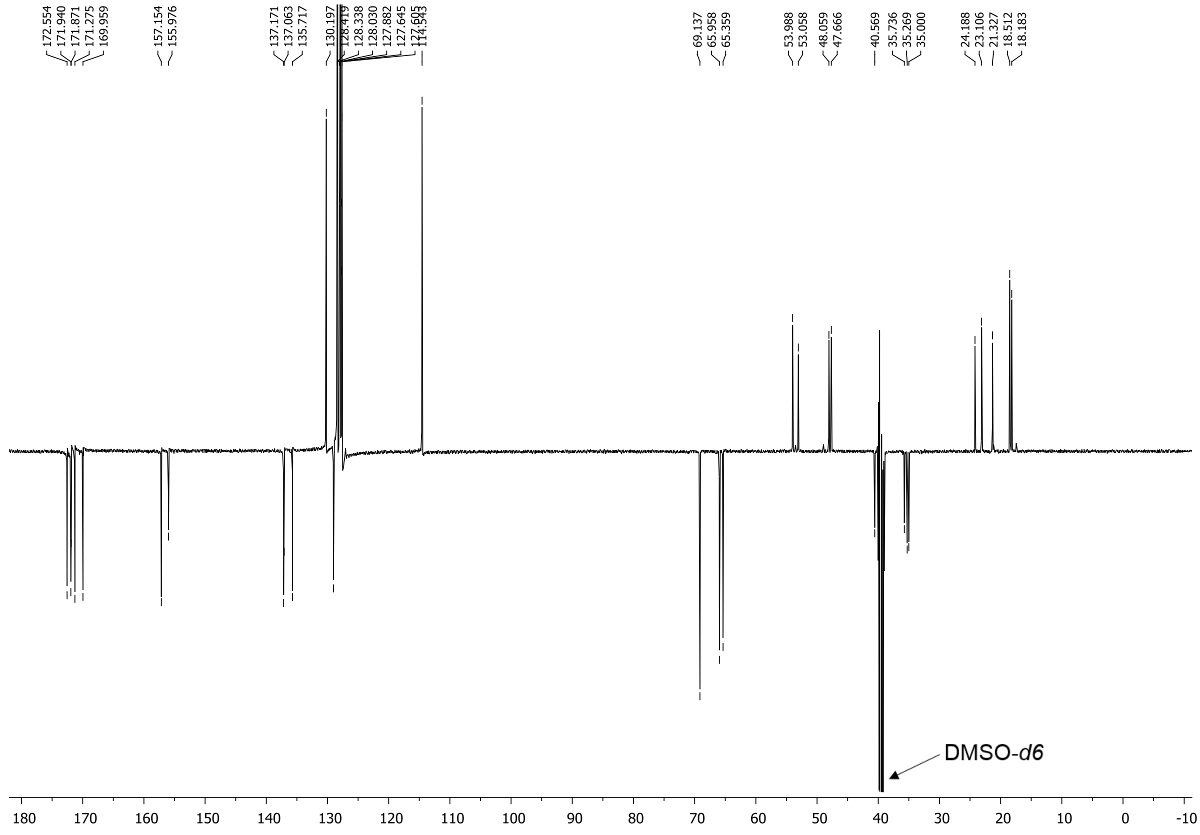

H-Leu-Ala-βAla-Ala-Tyr-OH (106)

^{1}H NMR (500 MHz, CD_3OD)

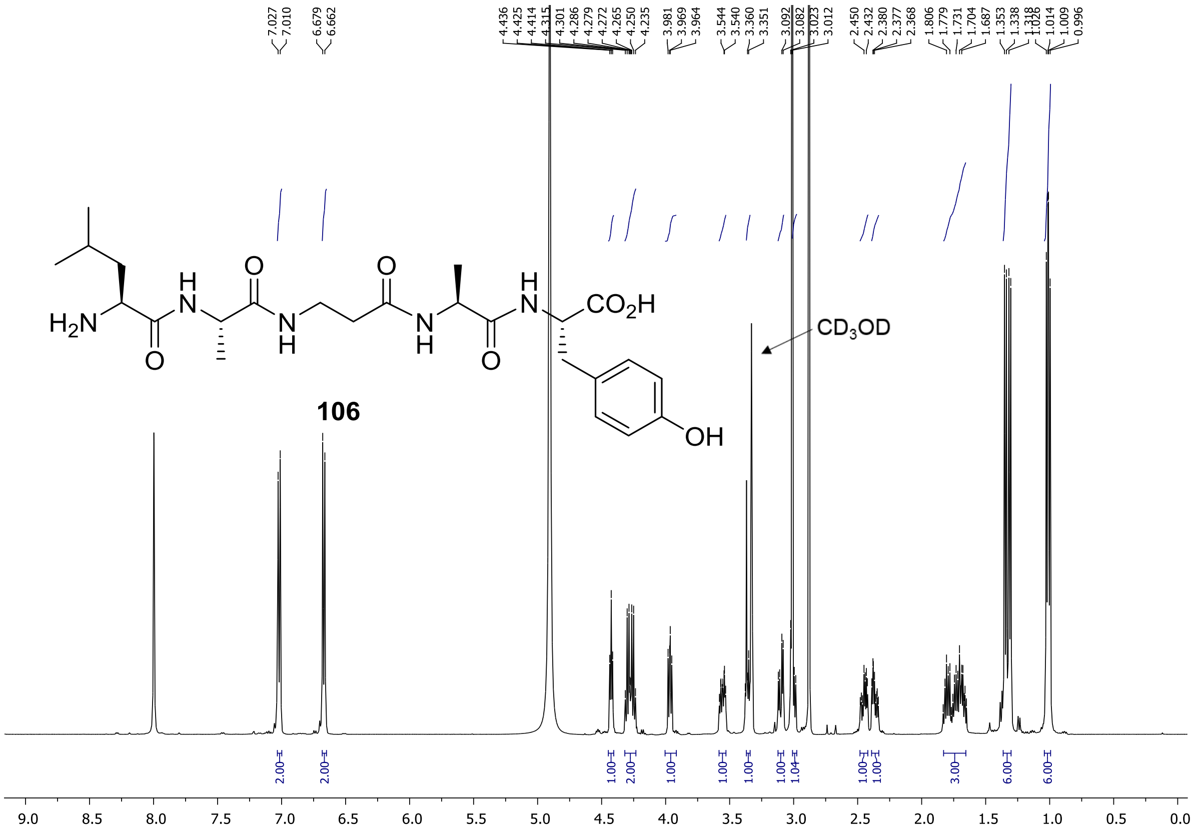

^{13}C NMR (126 MHz, CD_3OD)

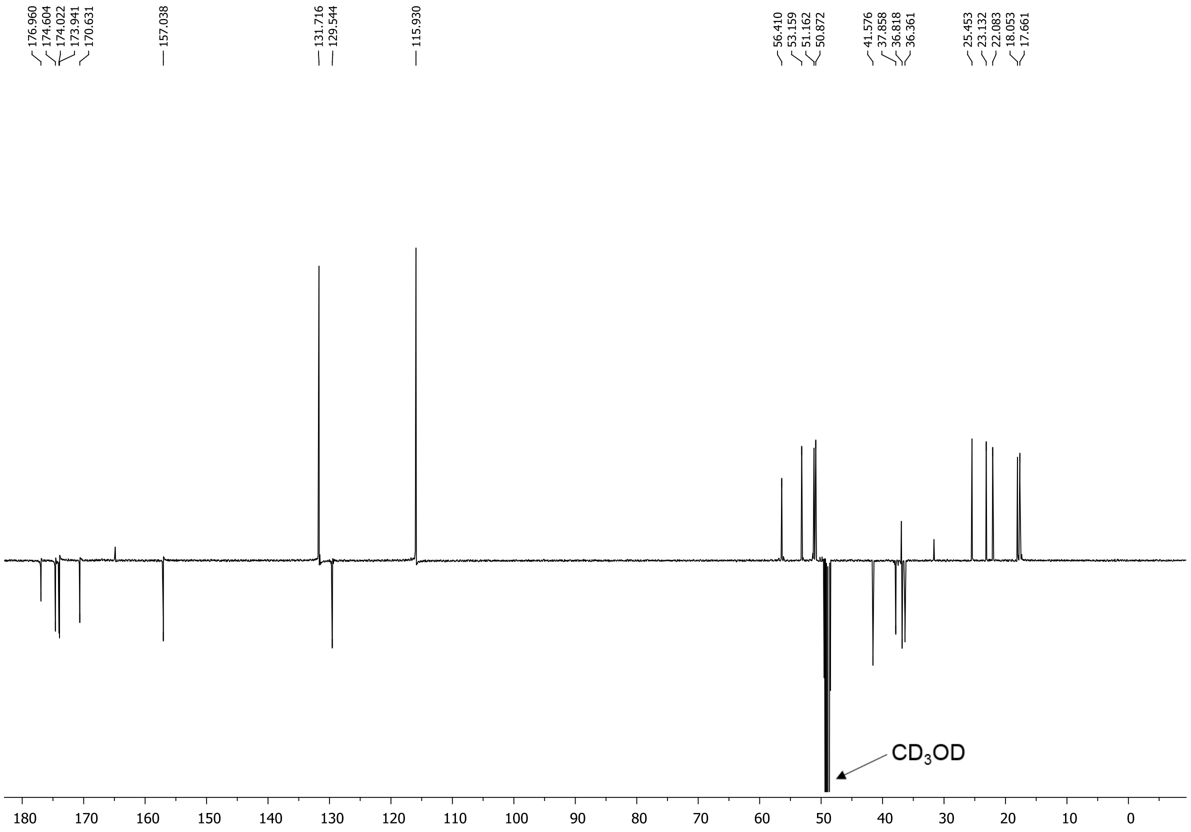

Cyclo(Leu-Ala-βAla-Ala-Tyr) (107)

^{1}H NMR (500 MHz, CD_3OD)

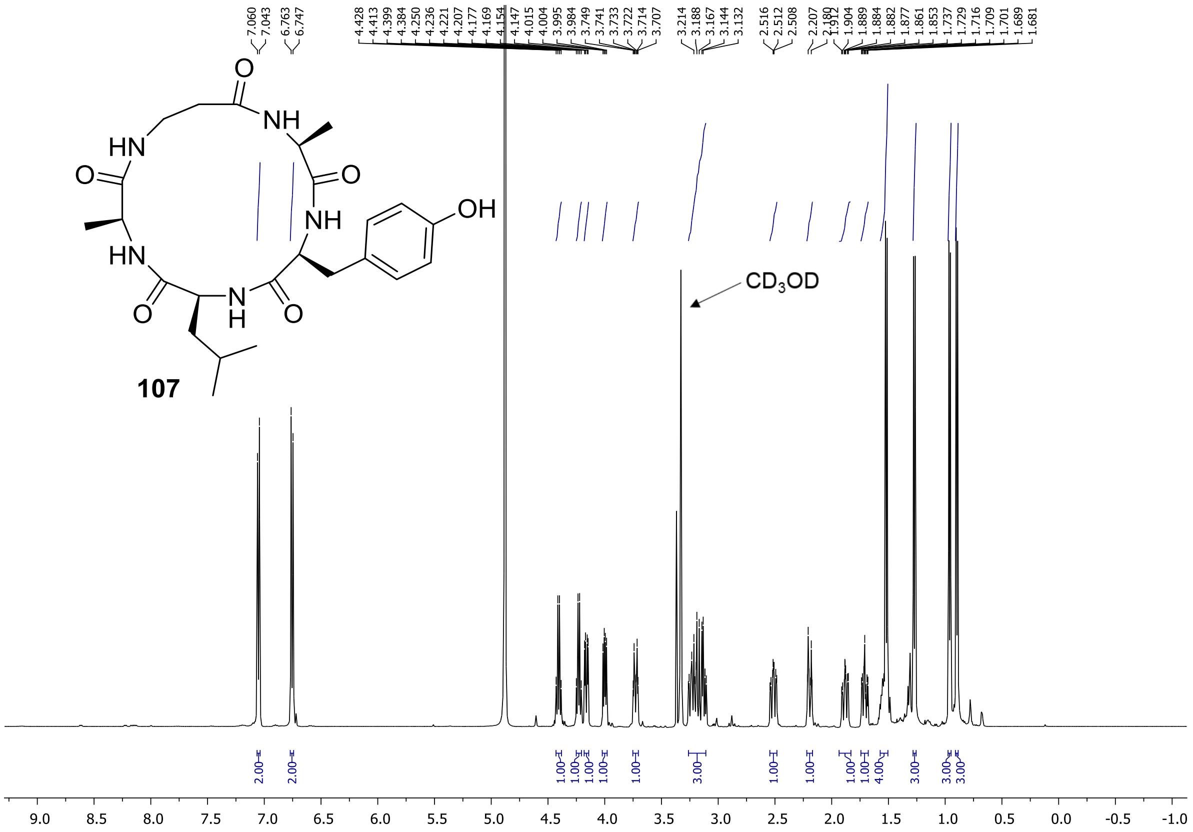

^{13}C NMR (126 MHz, CD_3OD)

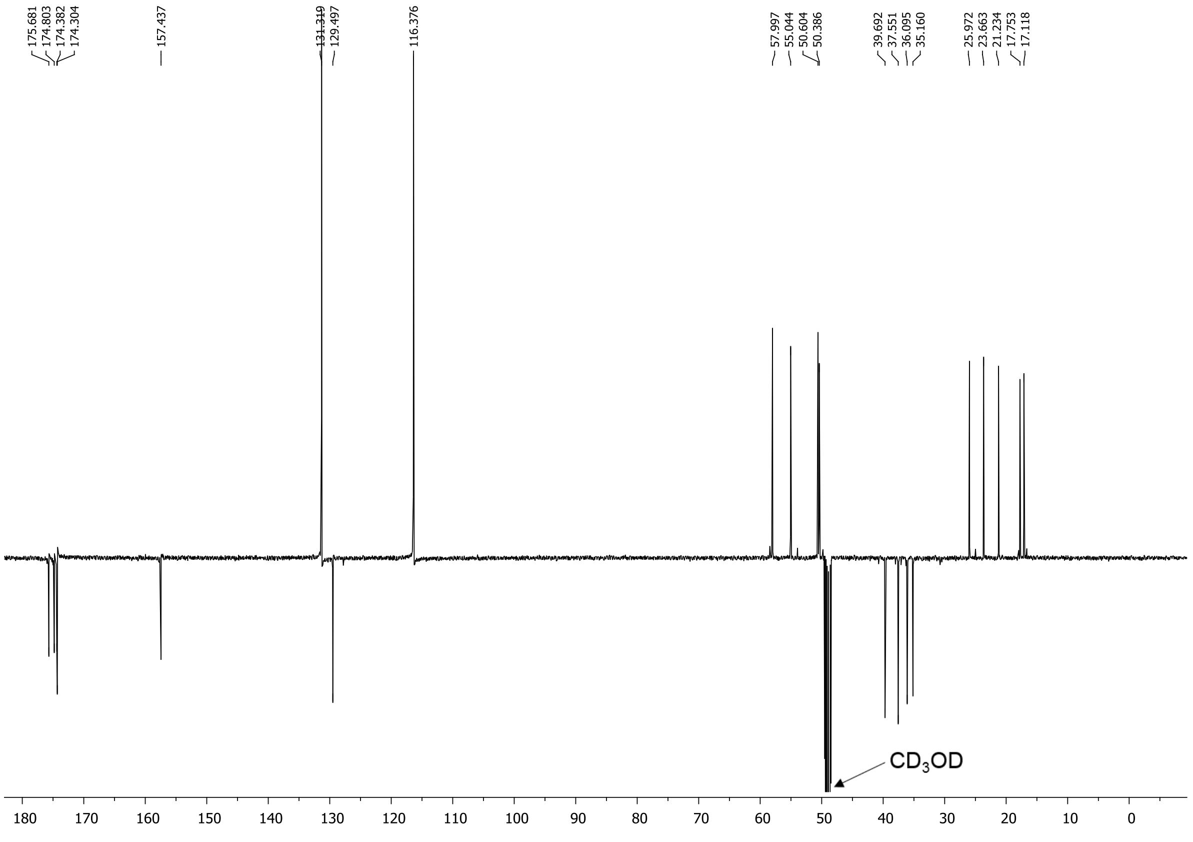

Boc-Aib-Ala-Tyr(Bn)-OBn (108)

^{1}H NMR (500 MHz, $CDCl_3$)

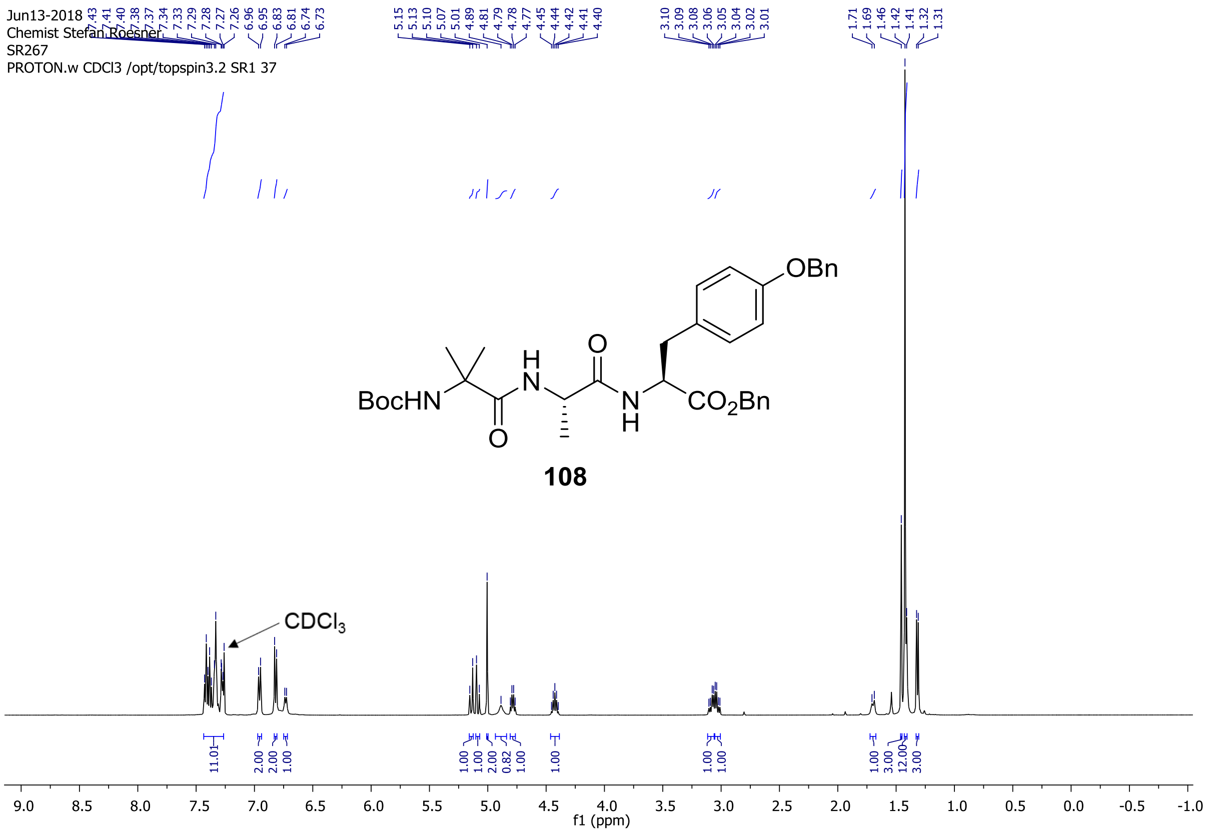

^{13}C NMR (126 MHz, $CDCl_3$)

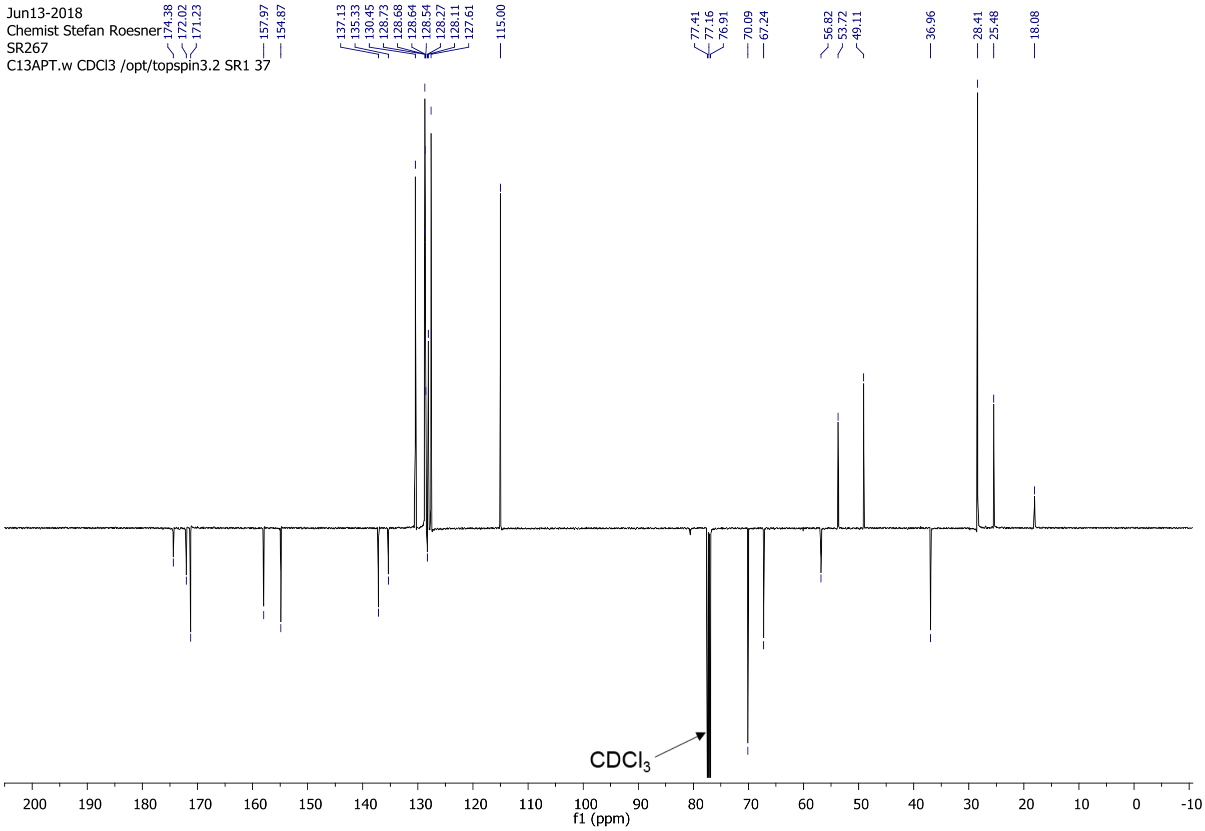

Boc-Ala-Aib-Ala-Tyr(Bn)-OBn (109)

^{1}H NMR (500 MHz, $CDCl_3$)

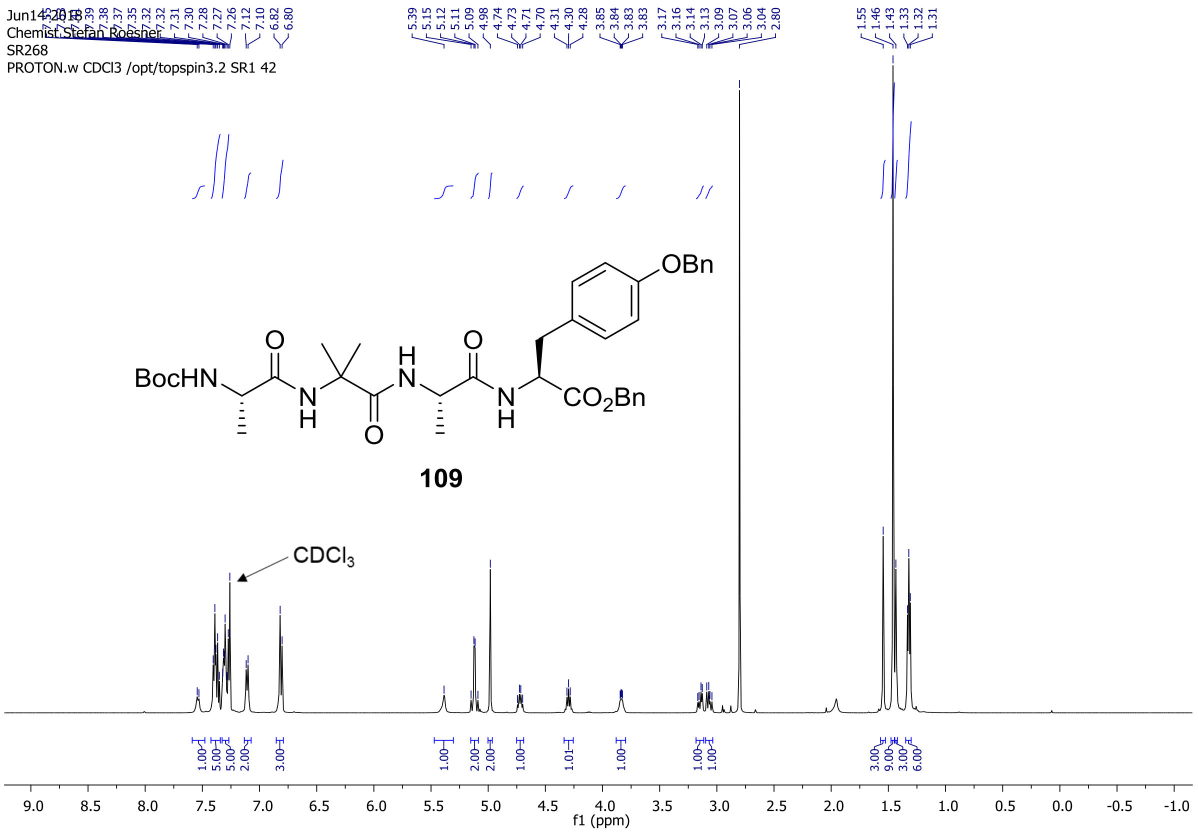

^{13}C NMR (126 MHz, $CDCl_3$)

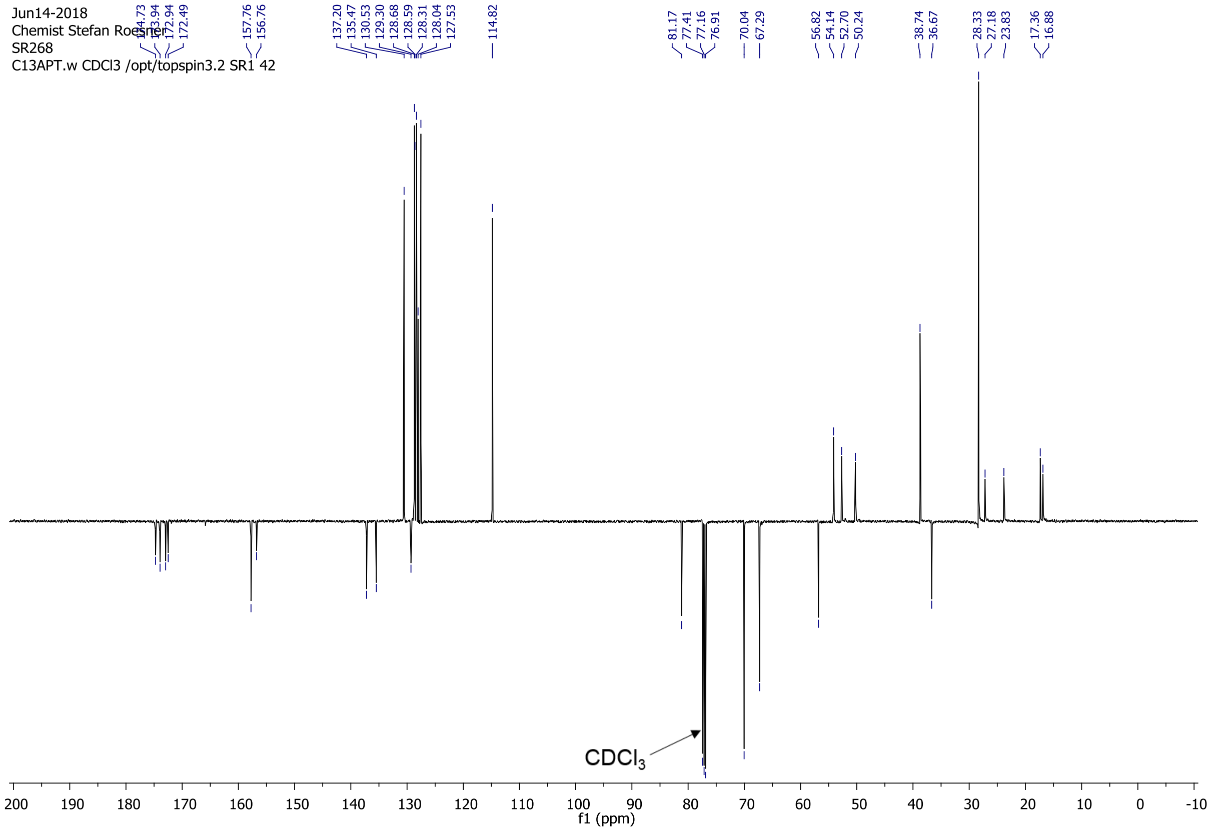

Cbz-Leu-Ala-Aib-Ala-Tyr(Bn)-OBn (110)

^{1}H NMR (500 MHz, $CDCl_3$)

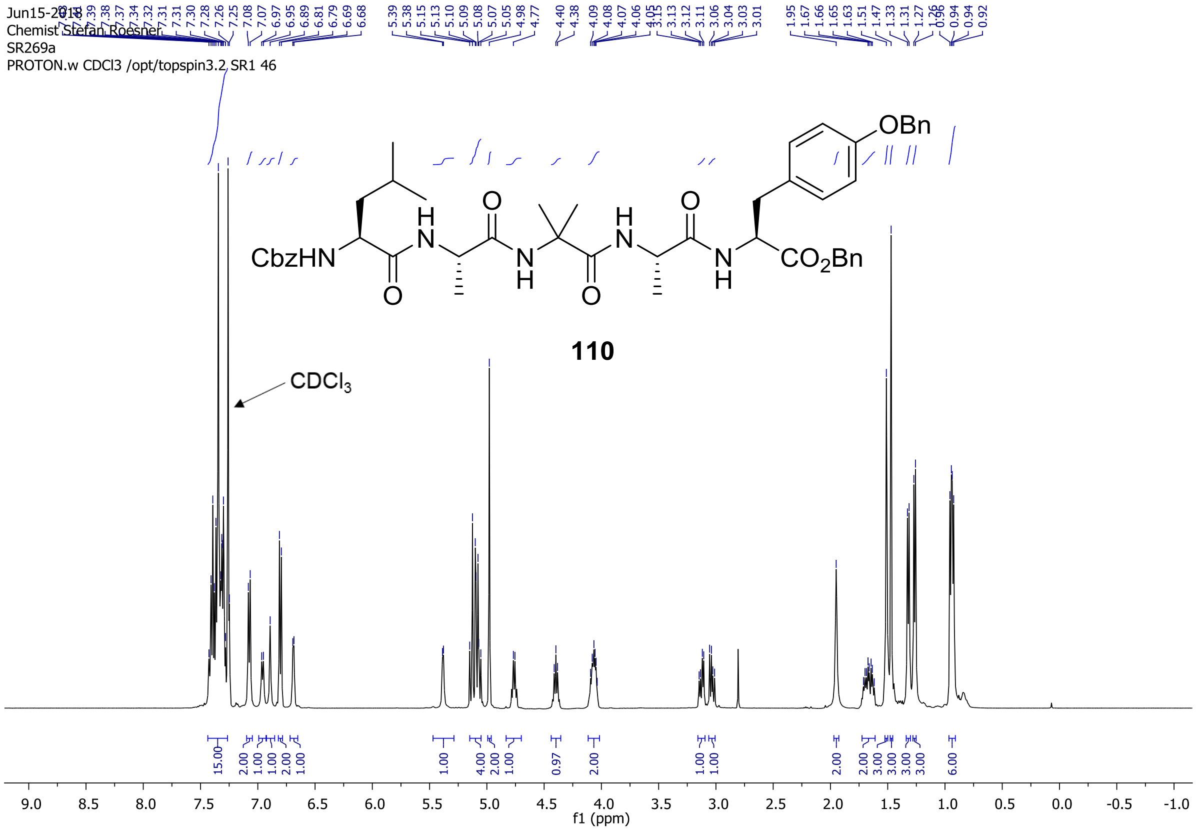

^{13}C NMR (126 MHz, $CDCl_3$)

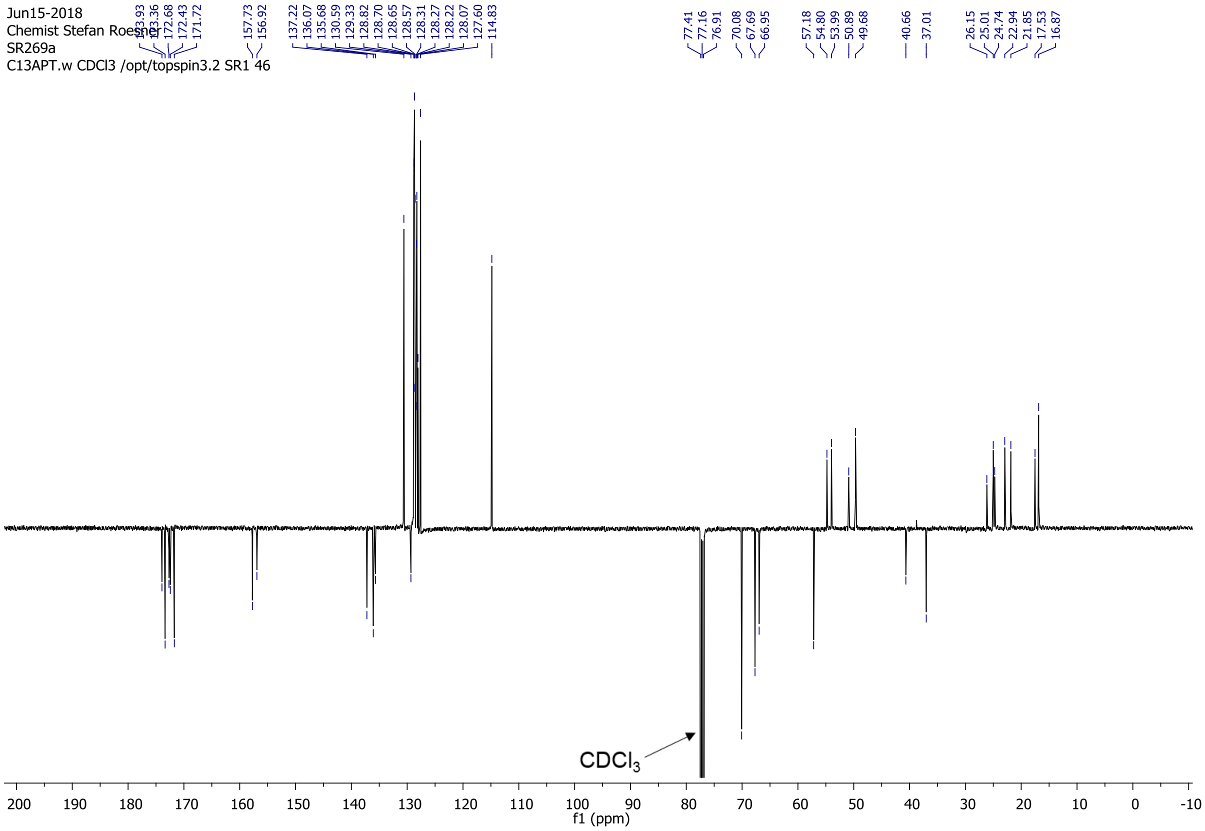

H-Leu-Ala-Aib-Ala-Tyr-OH (111)

^{1}H NMR (500 MHz, CD_3OD)

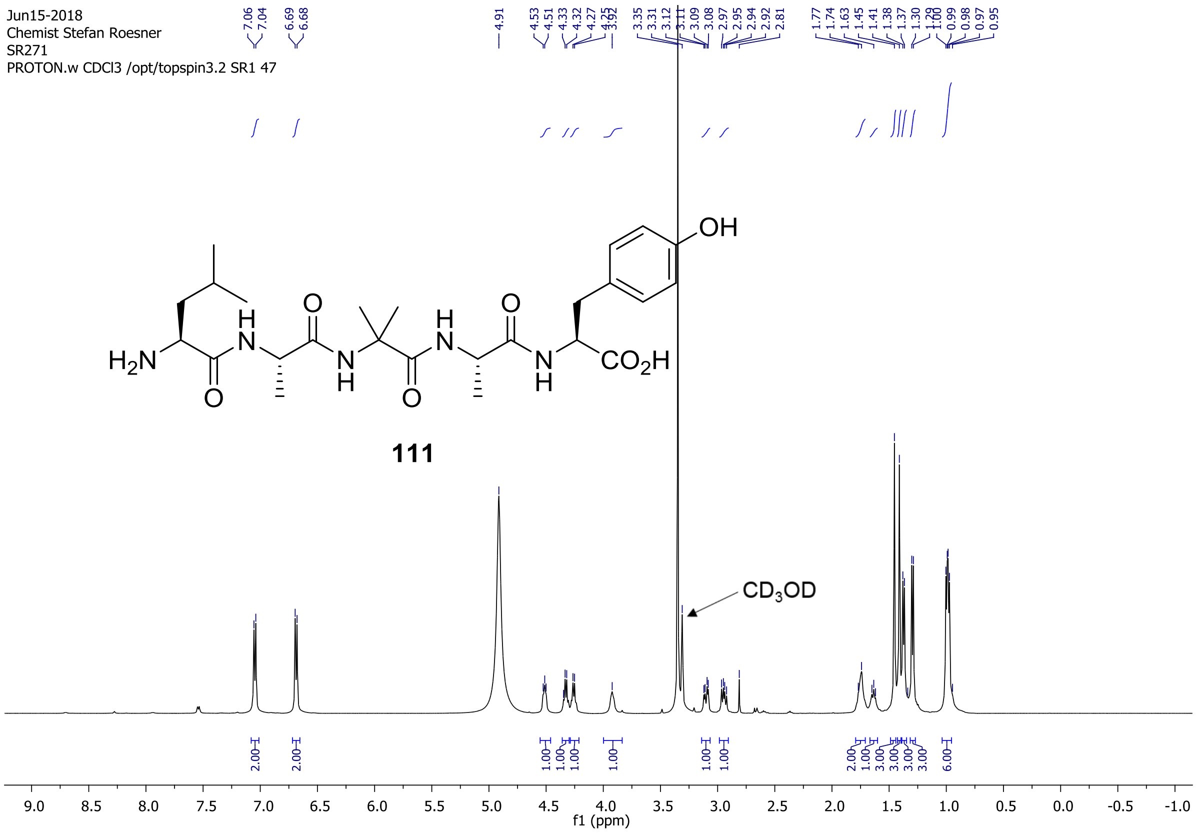

^{13}C NMR (126 MHz, CD_3OD)

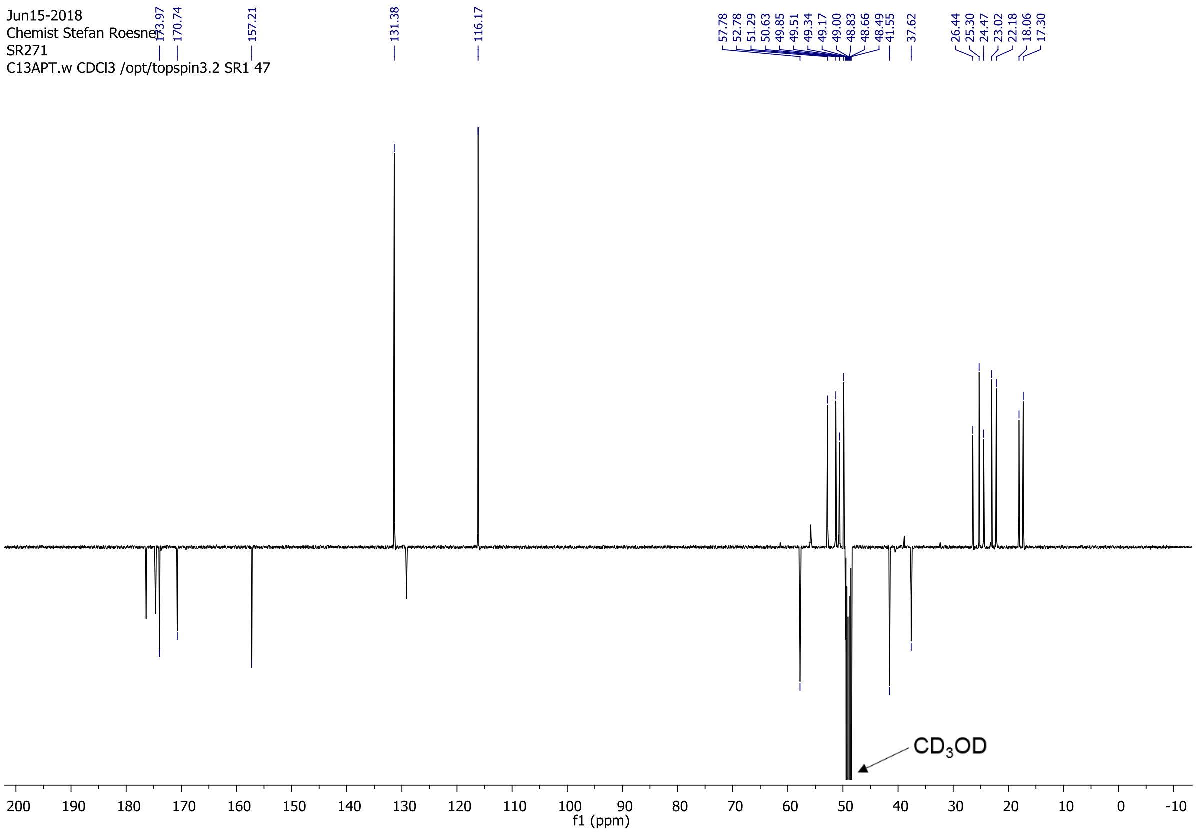

Cyclo(Leu-Ala-Gly-Aib-Tyr) (112)

^{1}H NMR (400 MHz, CD_3OD)

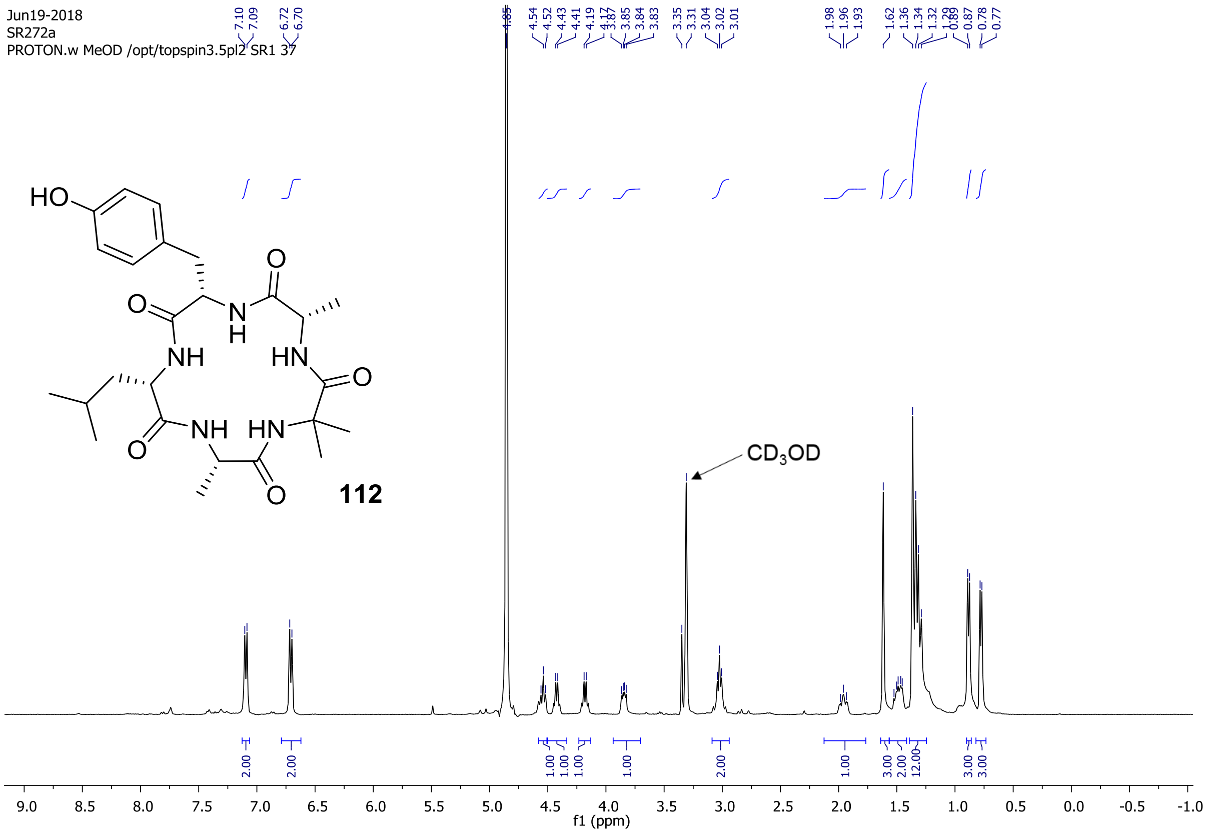

^{13}C NMR (126 MHz, CD_3OD)

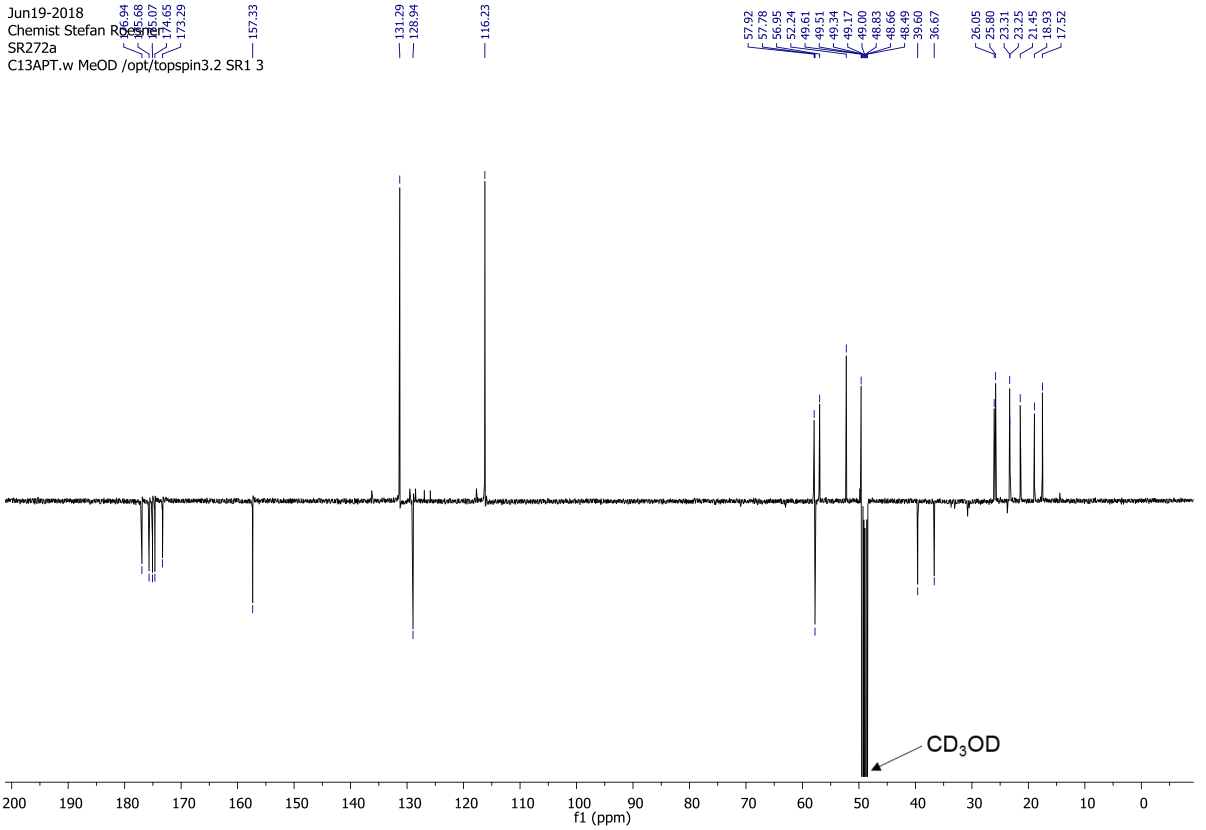

Fmoc-Thr(*t*Bu)-OCumyl (113)

^{1}H NMR (500 MHz, $CDCl_3$)

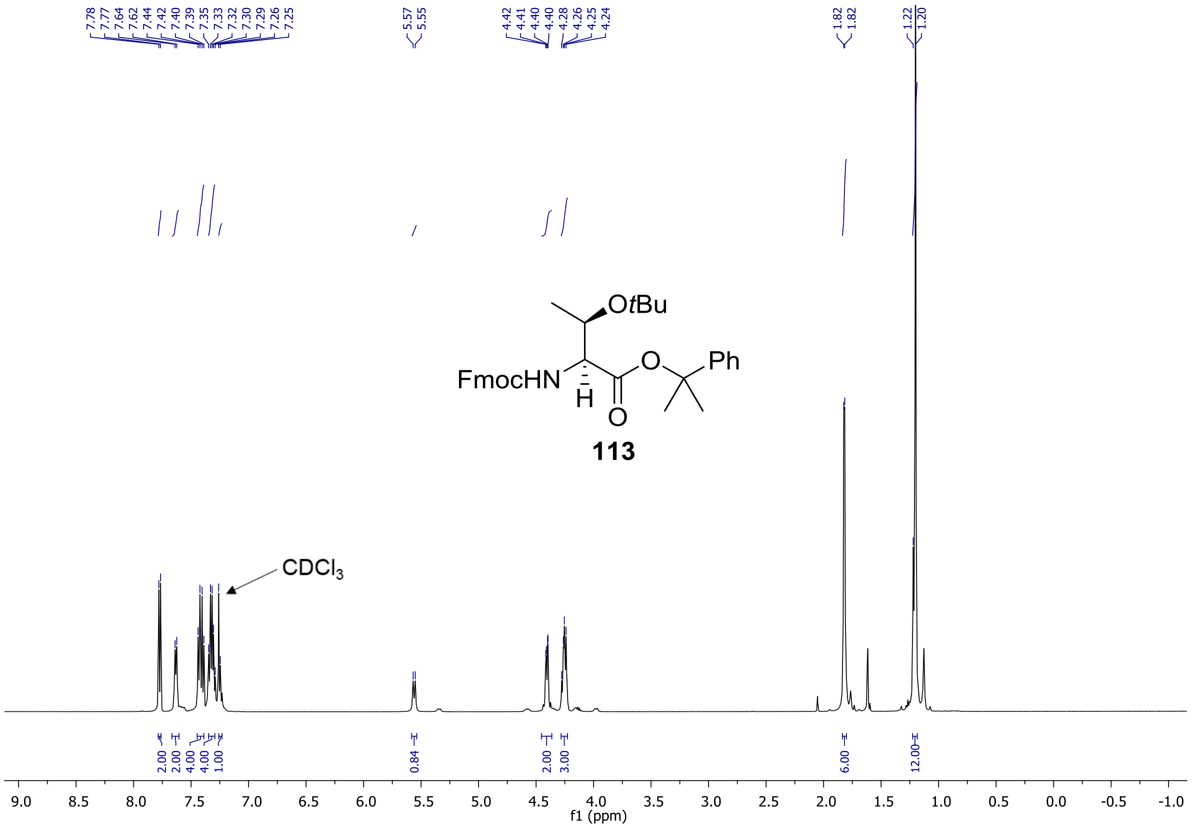

^{13}C NMR (126 MHz, $CDCl_3$)

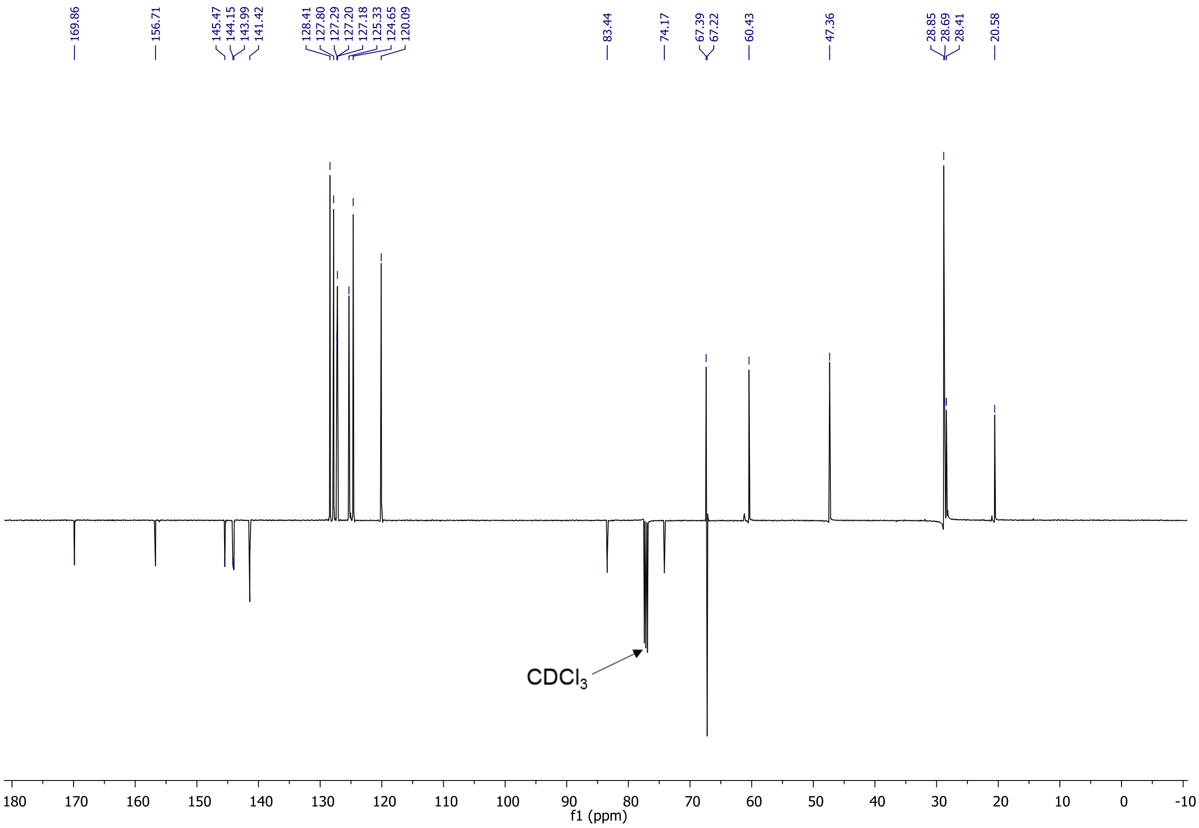

NO_2-GOx-Thr(*t*Bu)-OCumyl (114)

^{1}H NMR (500 MHz, $CDCl_3$)

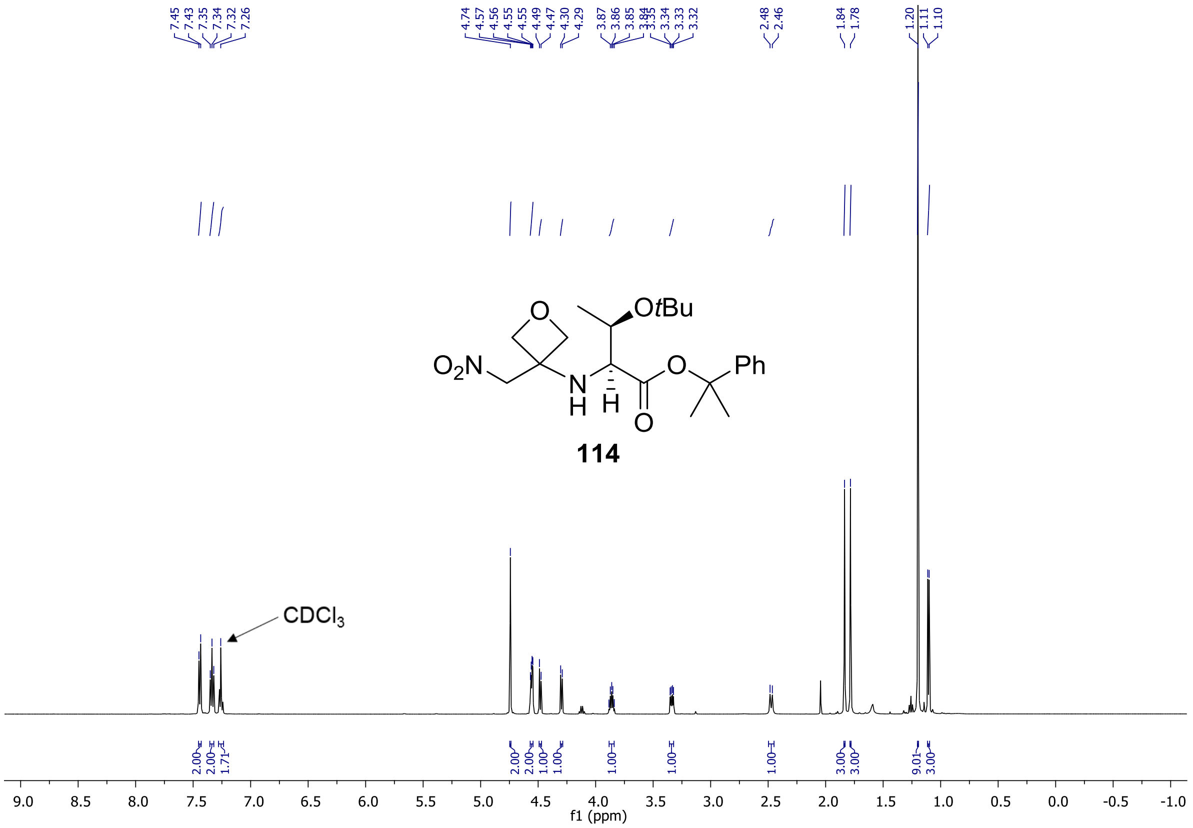

^{13}C NMR (126 MHz, $CDCl_3$)

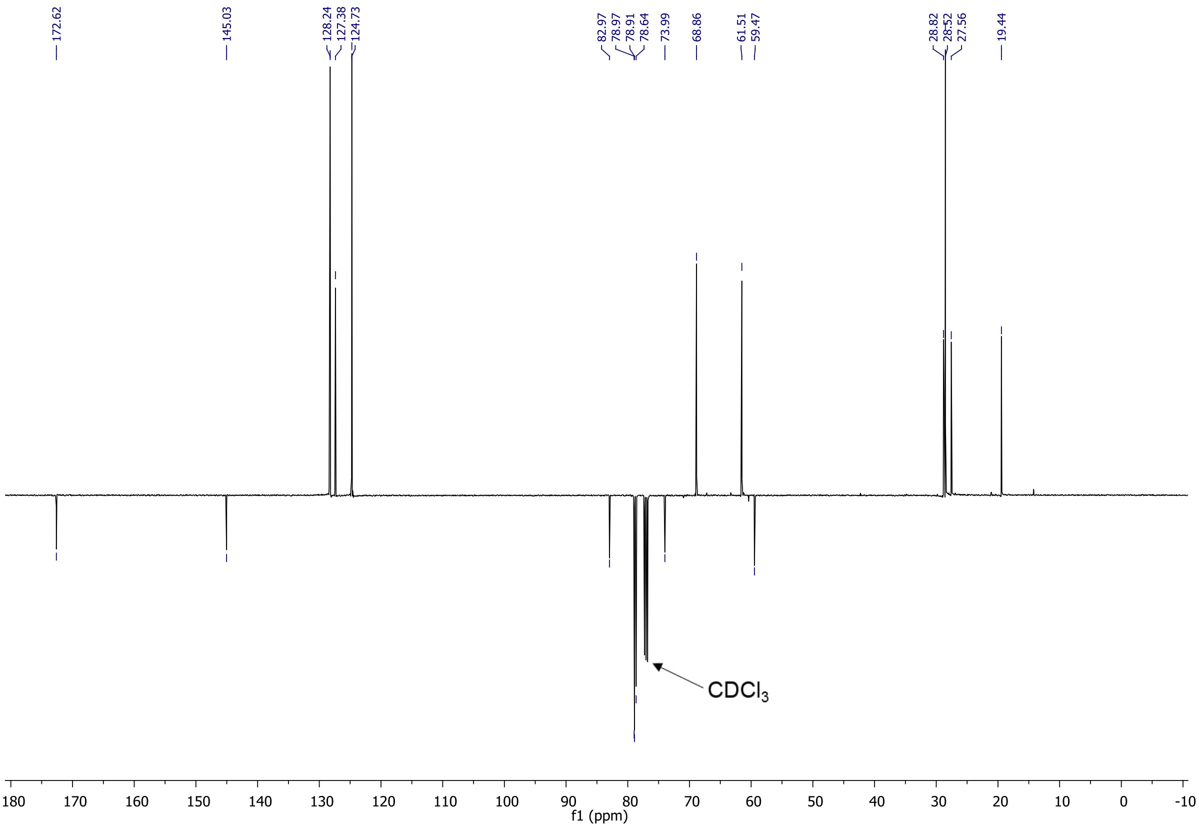

Fmoc-Val-GOx-Thr(*t*Bu)-OCumyl (115)

^{1}H NMR (500 MHz, $CDCl_3$)

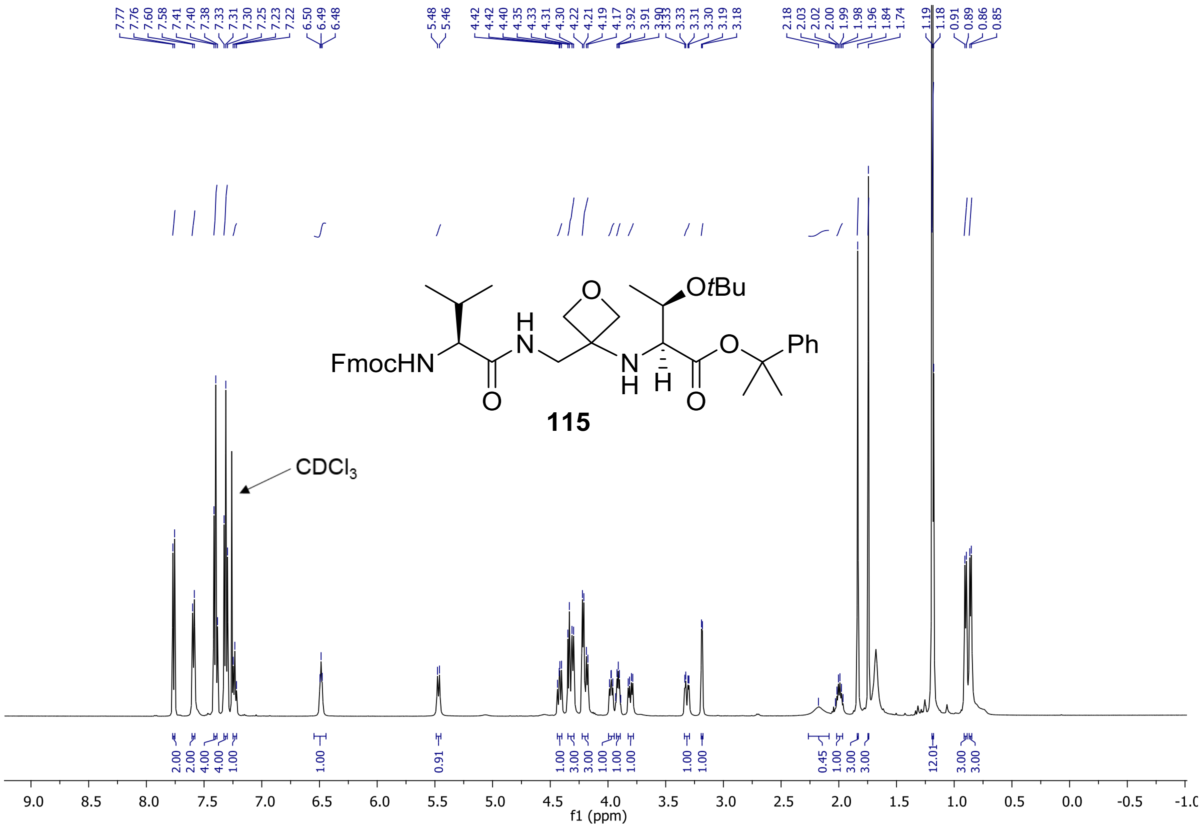

^{13}C NMR (126 MHz, $CDCl_3$)

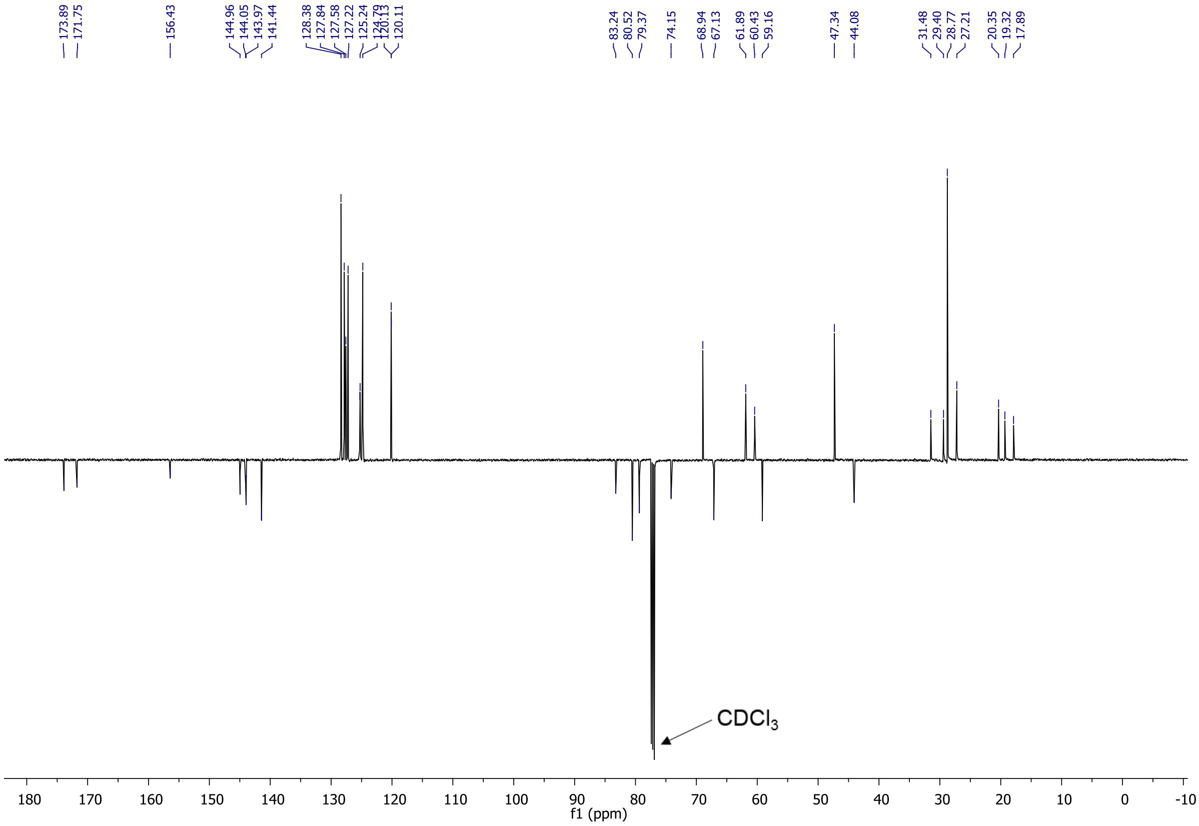

Fmoc-Val-GOx-Thr(*t*Bu)-Phe-OBn (116)

^{1}H NMR (500 MHz, $CDCl_3$)

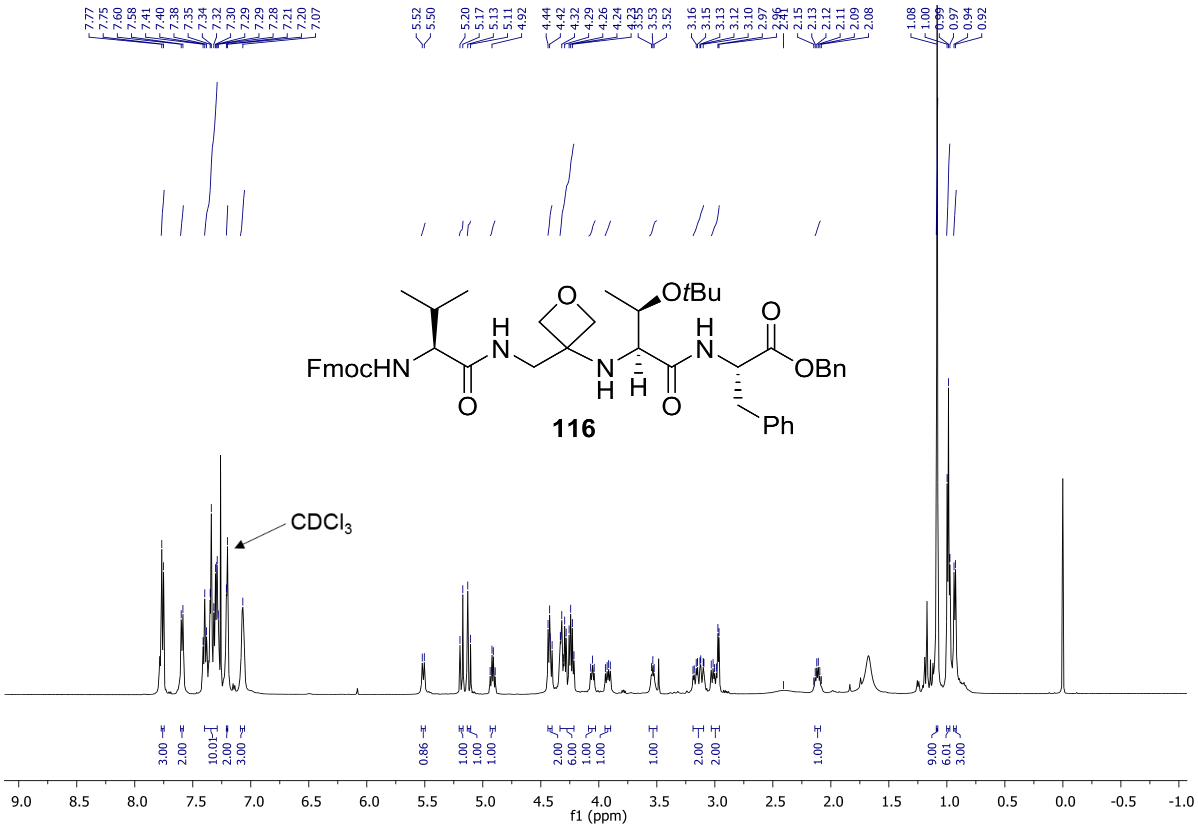

^{13}C NMR (126 MHz, $CDCl_3$)

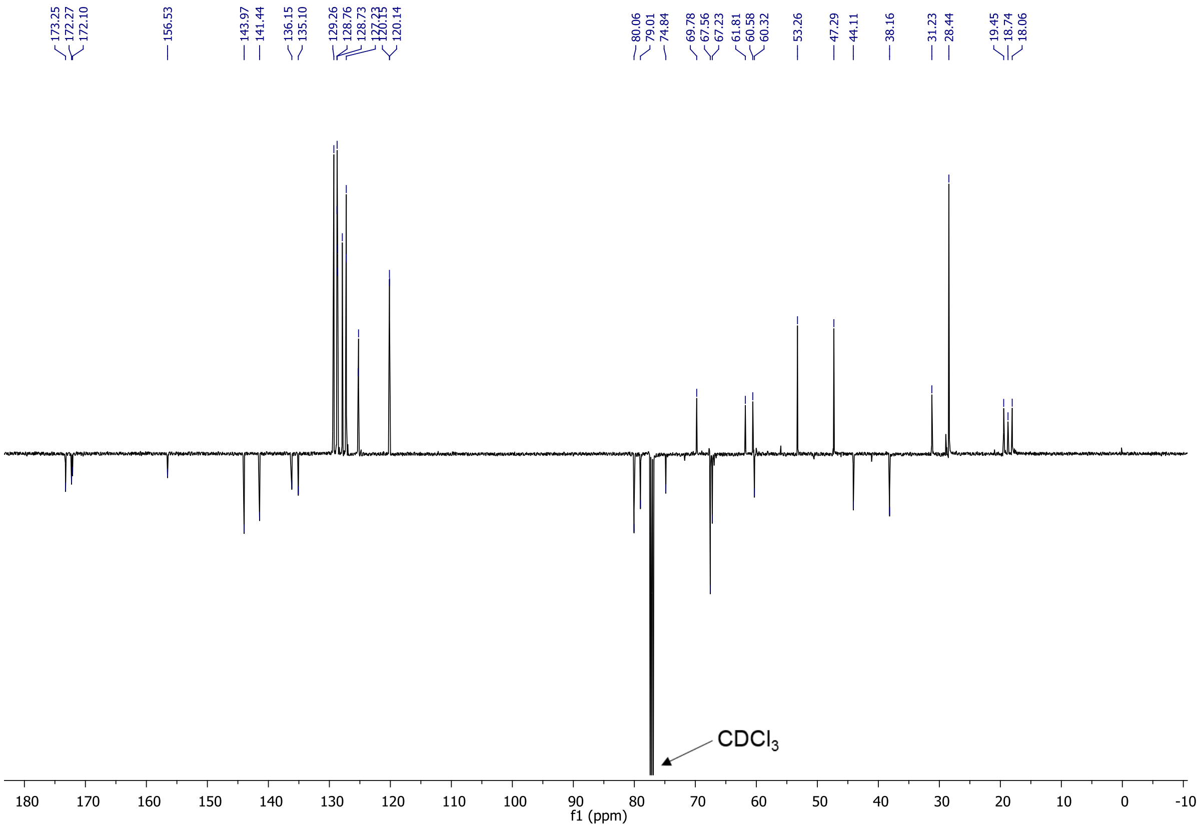

Fmoc-Tyr(*t*Bu)-Val-GOx-Thr(*t*Bu)-Phe-OBn (117)

^{1}H NMR (500 MHz, $CDCl_3$)

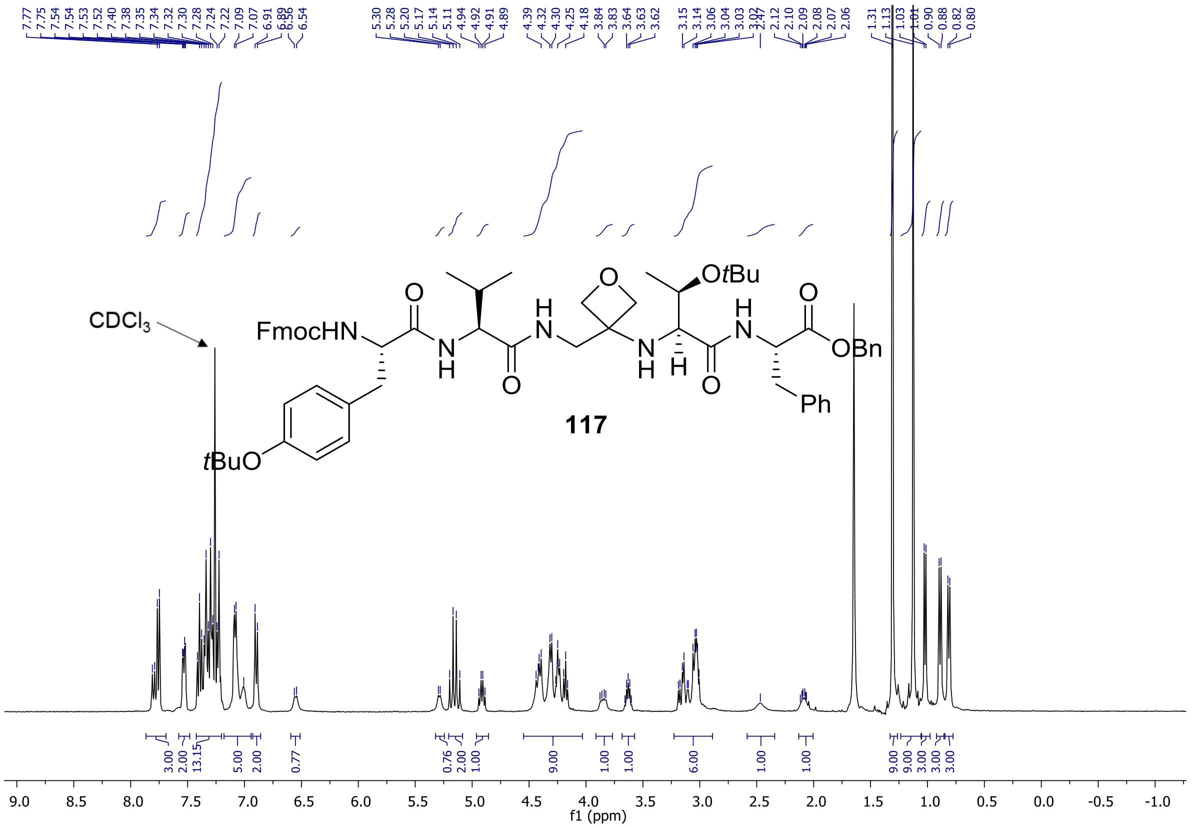

^{13}C NMR (126 MHz, $CDCl_3$)

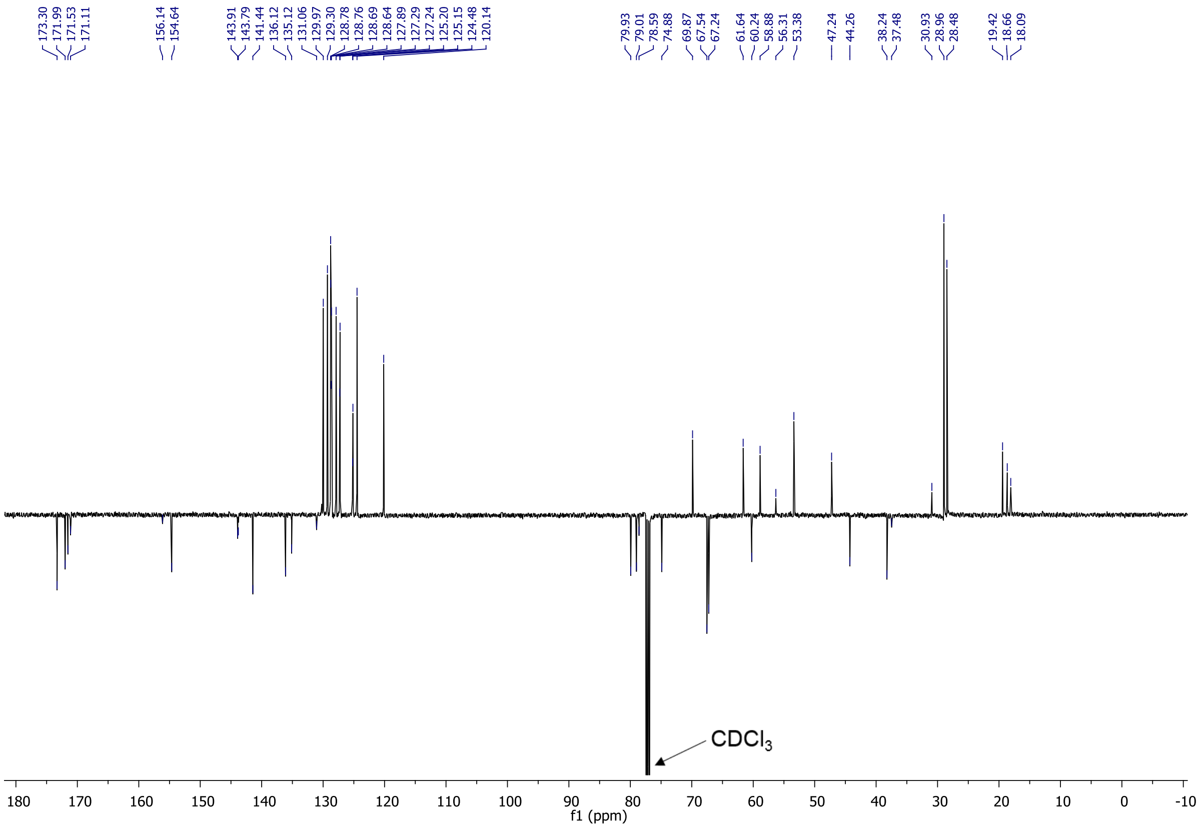

Cbz-Leu-Tyr(*t*Bu)-Val-GOx-Thr(*t*Bu)-Phe-OBn (118)

^{1}H NMR (500 MHz, $CDCl_3$)

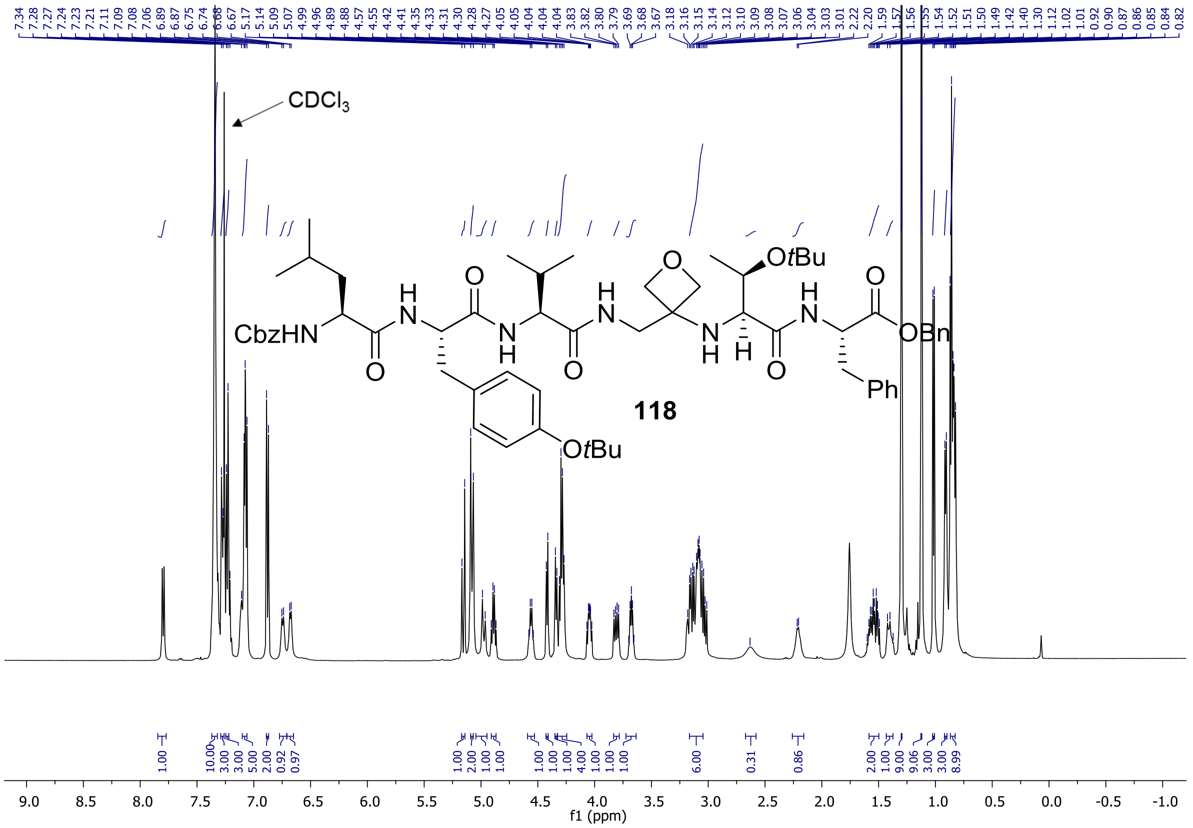

^{13}C NMR (126 MHz, $CDCl_3$)

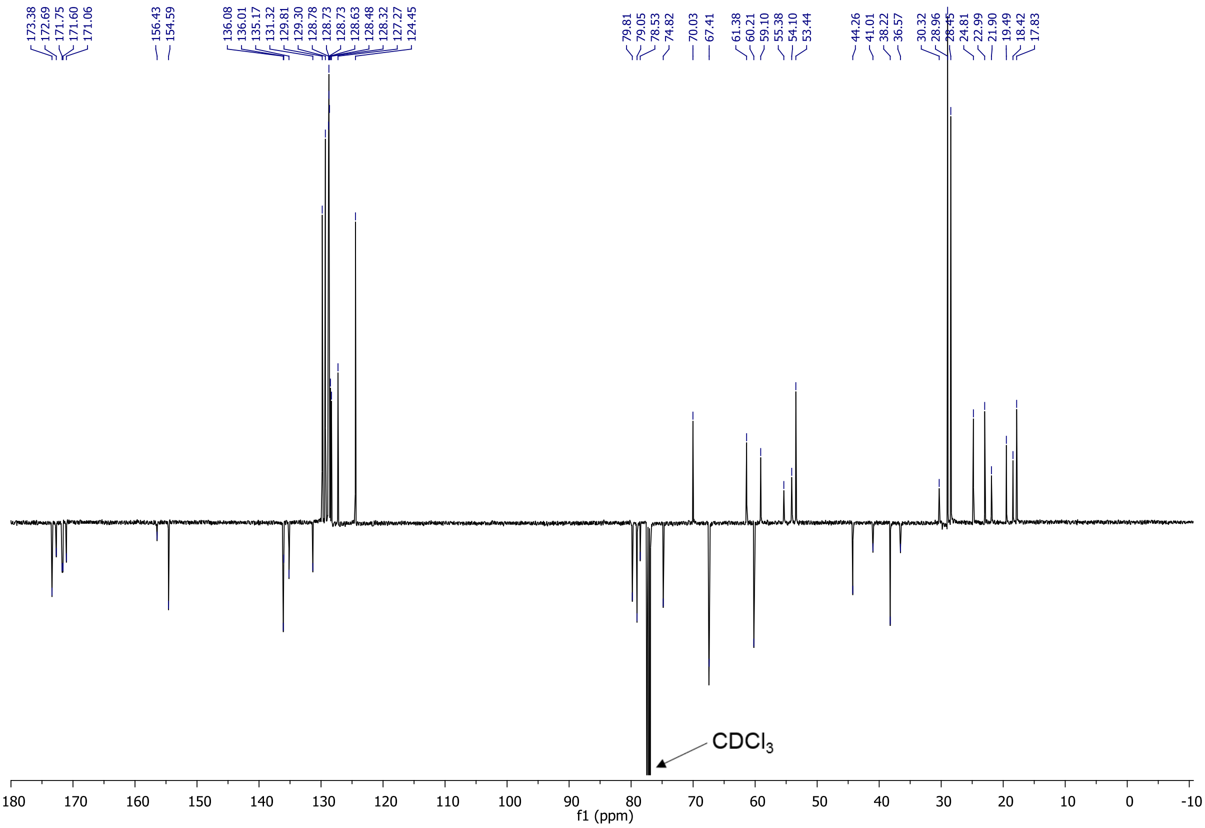

H-Leu-Tyr(*t*Bu)-Val-GOx-Thr(*t*Bu)-Phe-OH (119)

^{1}H NMR (500 MHz, CD_3OD)

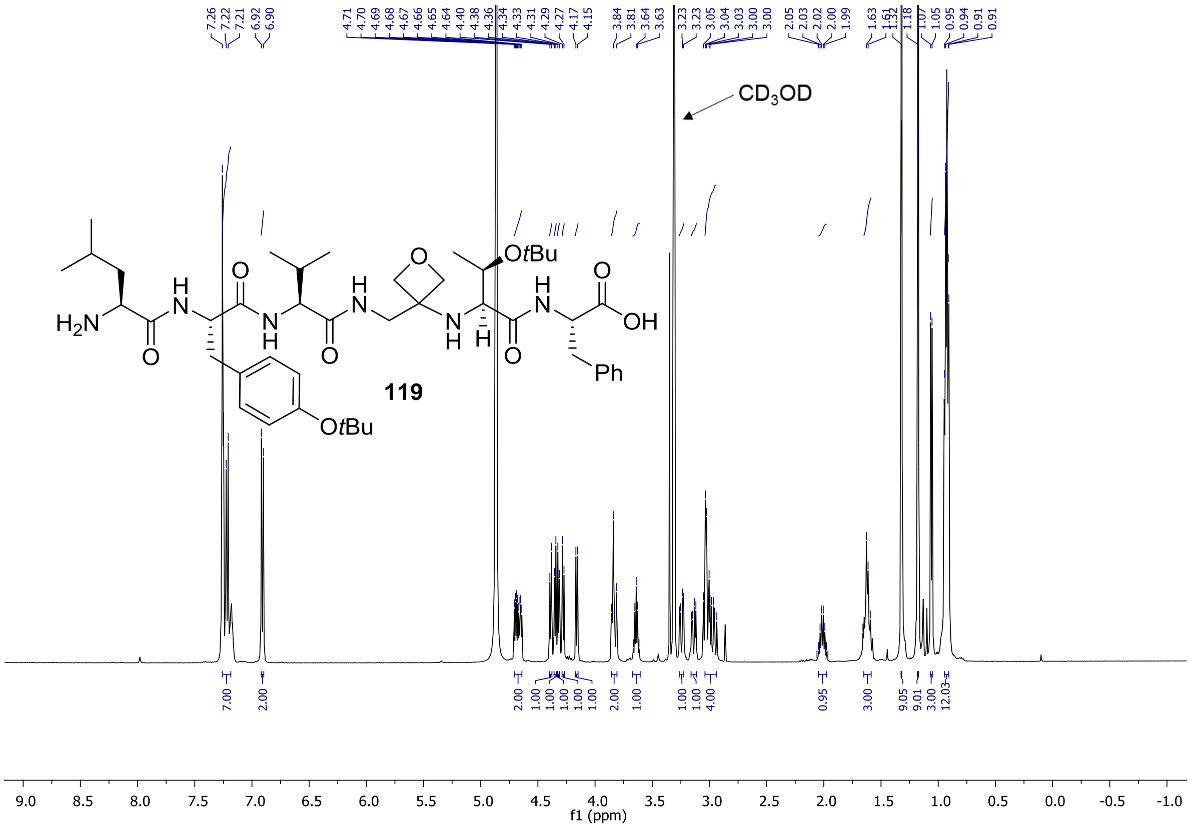

^{13}C NMR (126 MHz, CD_3OD)

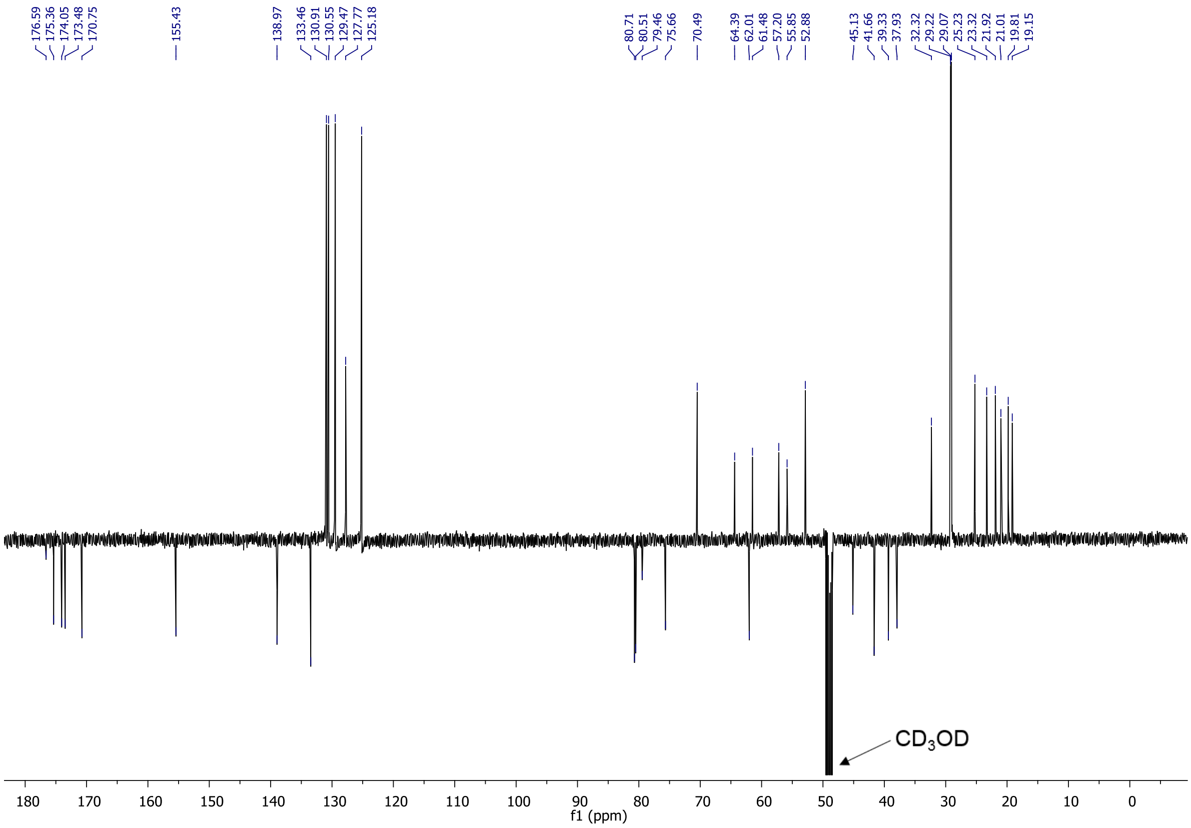

Cyclo(Leu-Tyr(tBu)-Val-GOx-Thr(tBu)-Phe) (14)

^1H NMR (500 MHz, CD_3OD)

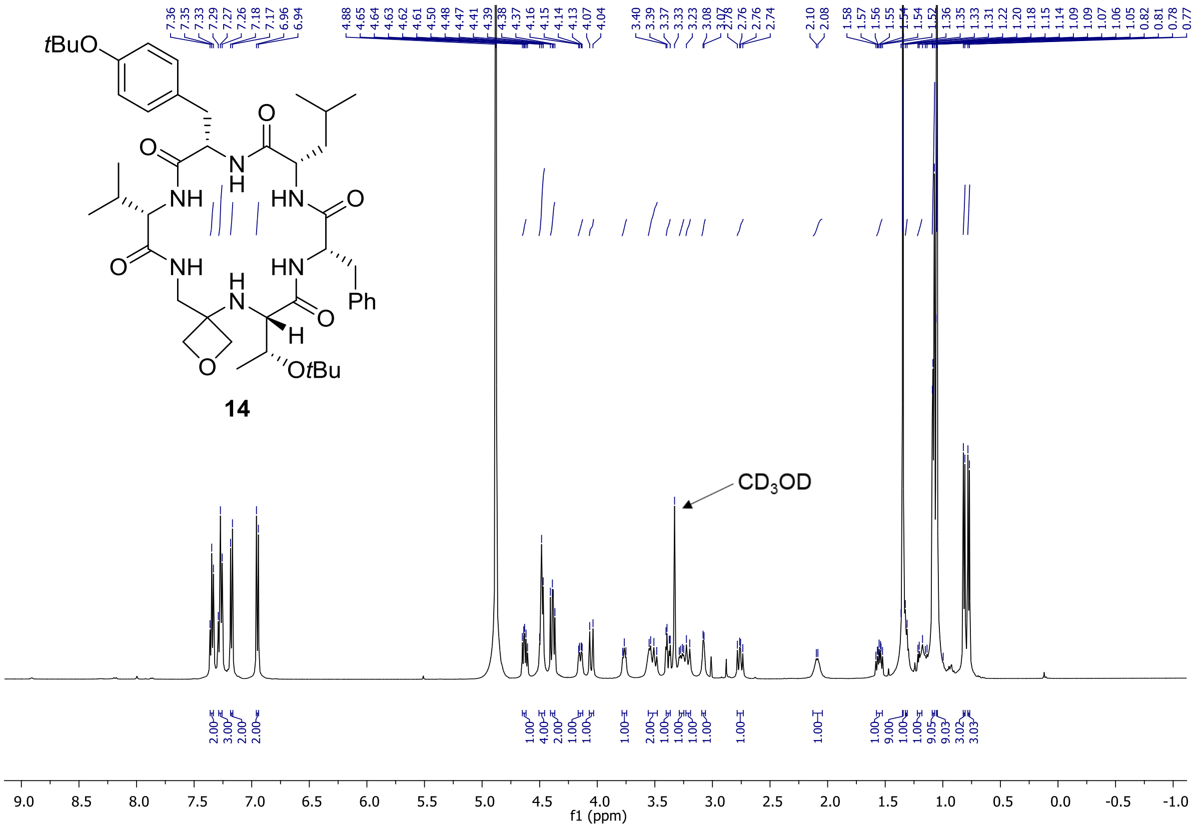

^{13}C NMR (126 MHz, $CDCl_3$)

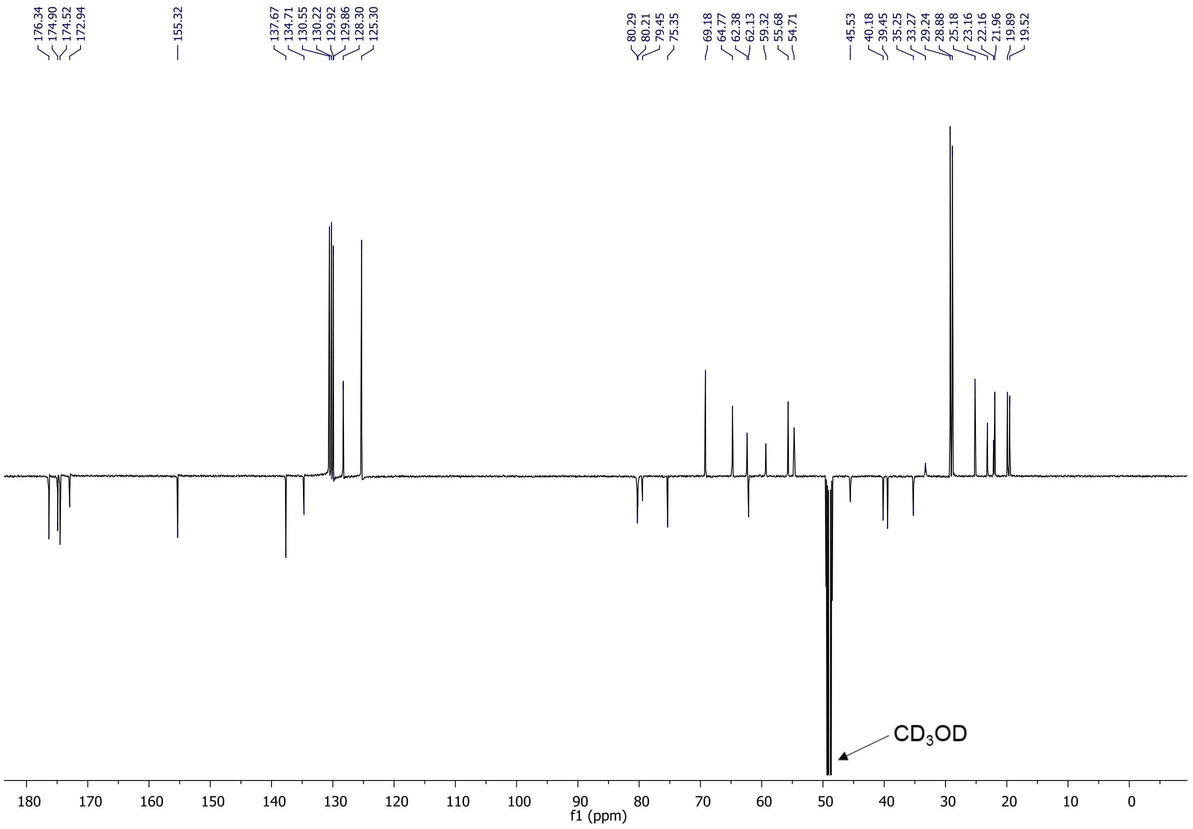

Fmoc-Gly-OCumyl (120)

^{1}H NMR (500 MHz, $CDCl_3$)

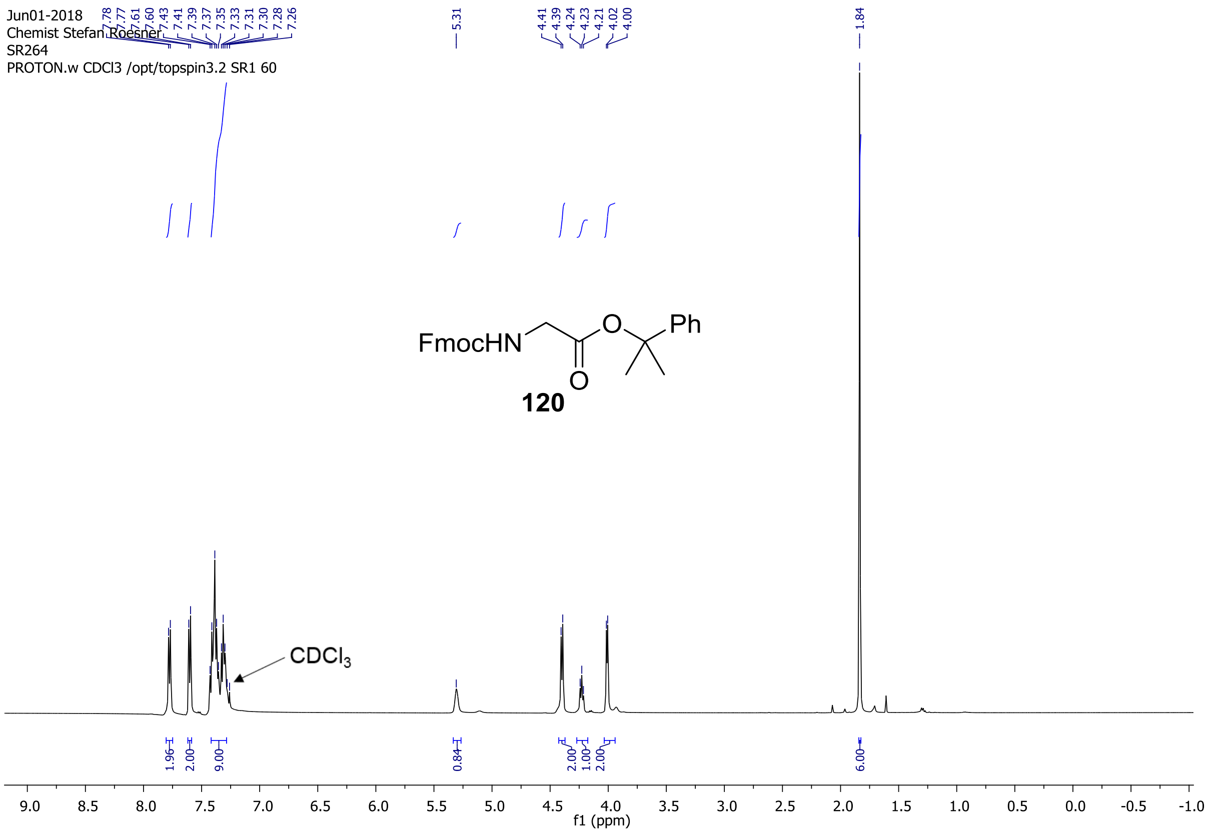

^{13}C NMR (126 MHz, $CDCl_3$)

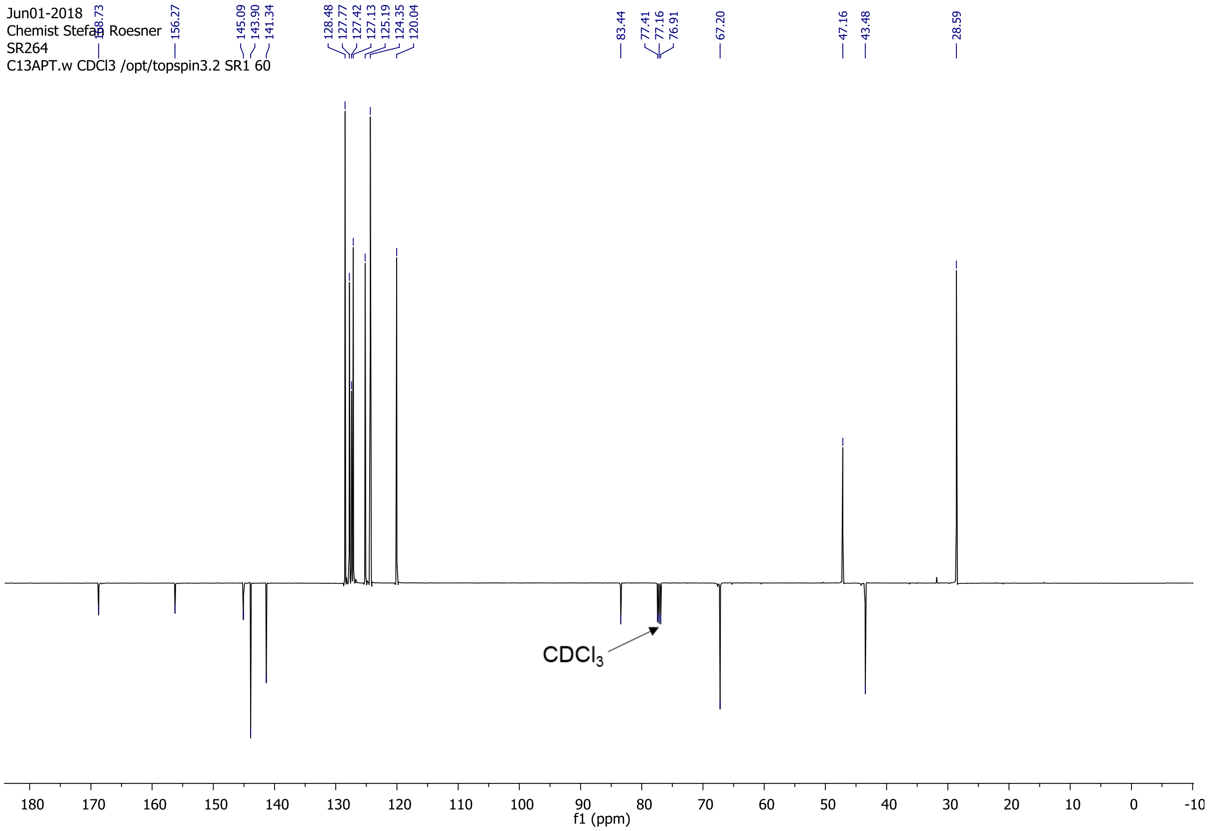

O_2N-GOx-Gly-OCumyl (121)

^{1}H NMR (400 MHz, $CDCl_3$)

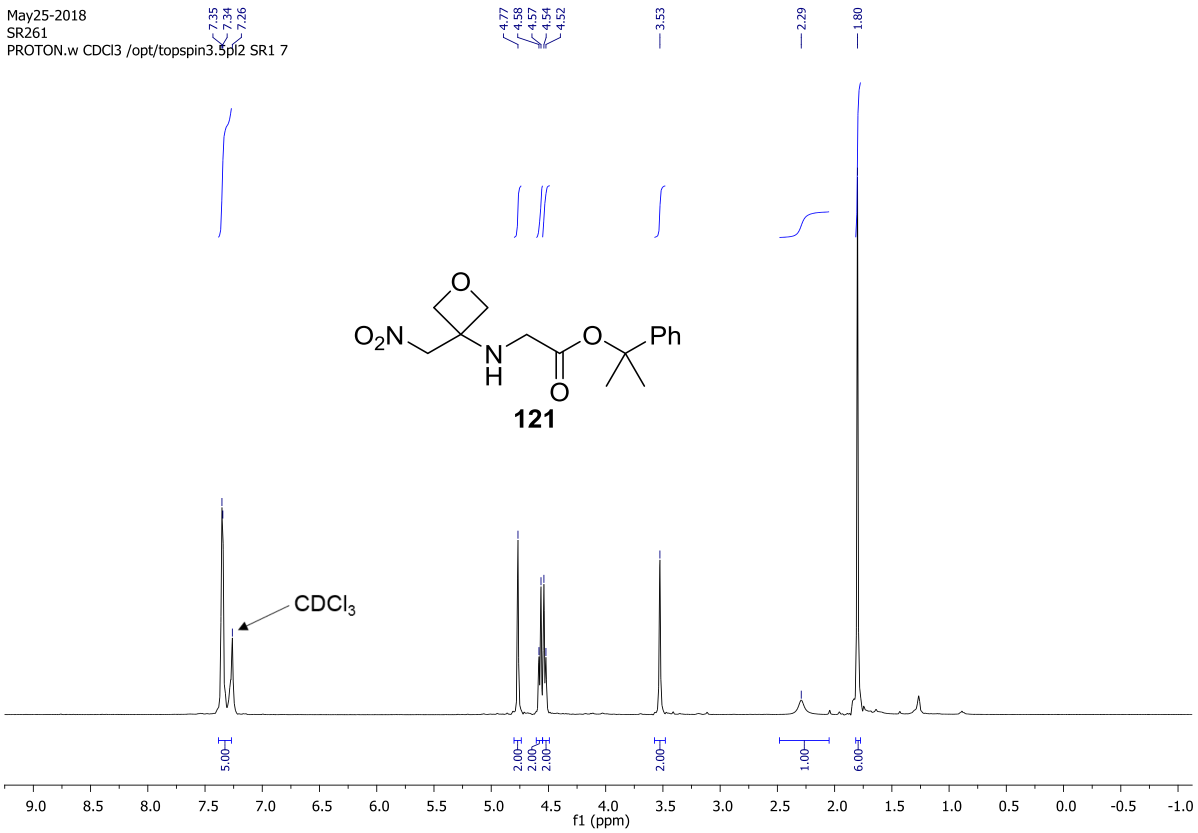

^{13}C NMR (101 MHz, $CDCl_3$)

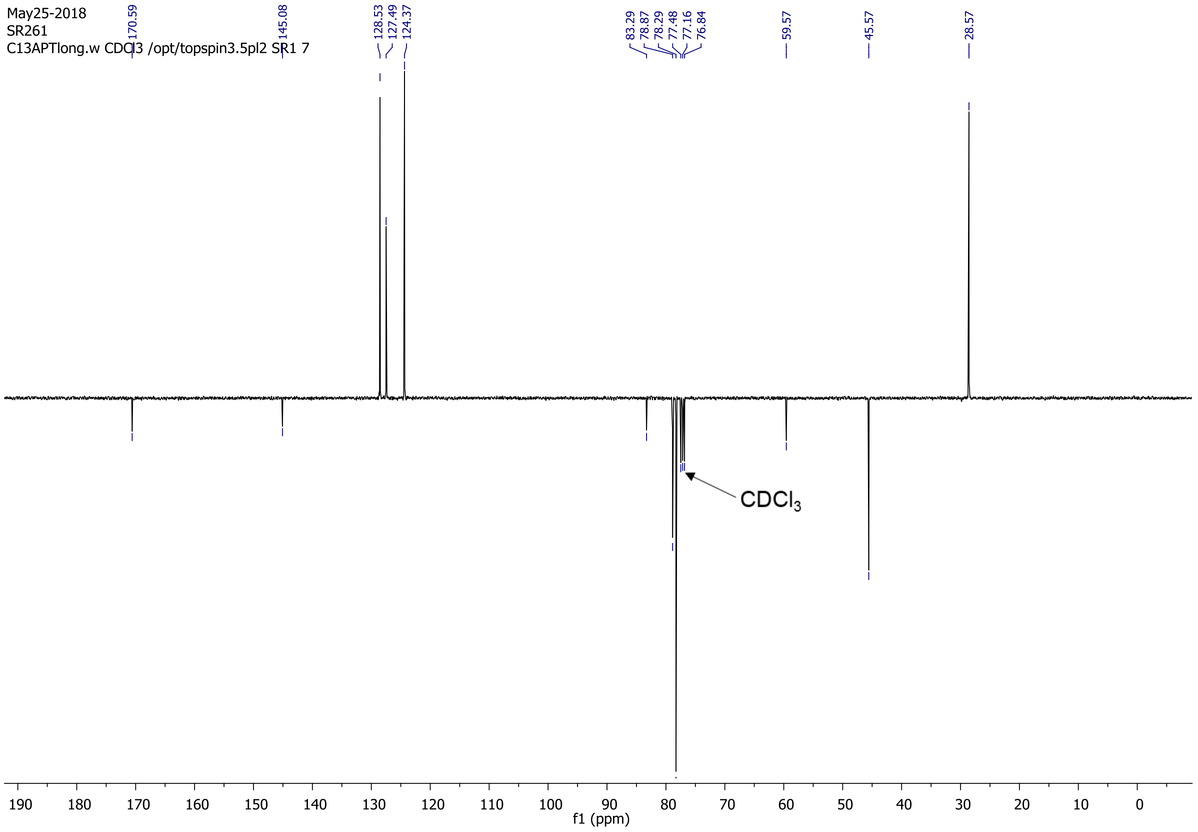

Fmoc-GOx-Gly-OCumyl (122)

^{1}H NMR (400 MHz, $CDCl_3$)

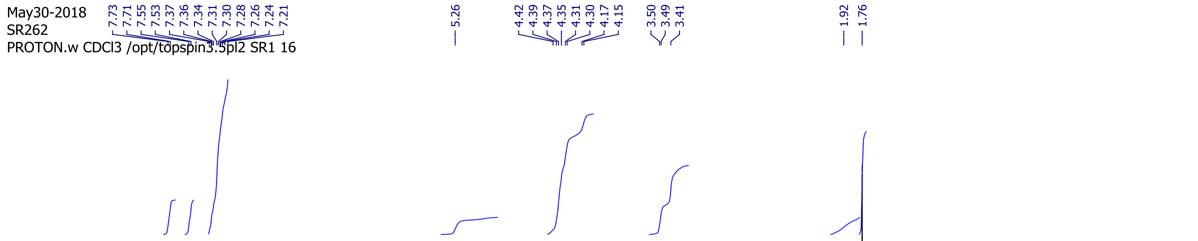

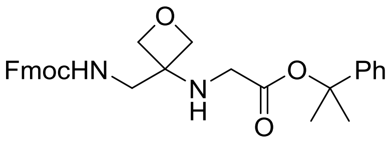

122

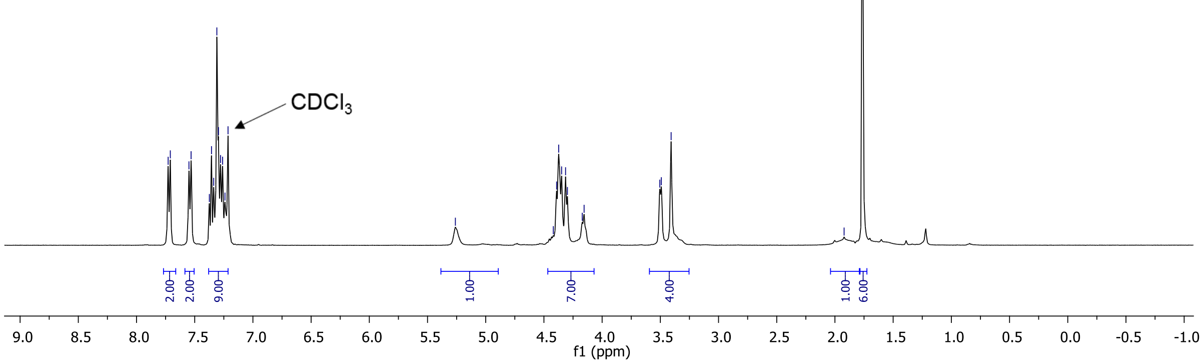

^{13}C NMR (101 MHz, $CDCl_3$)

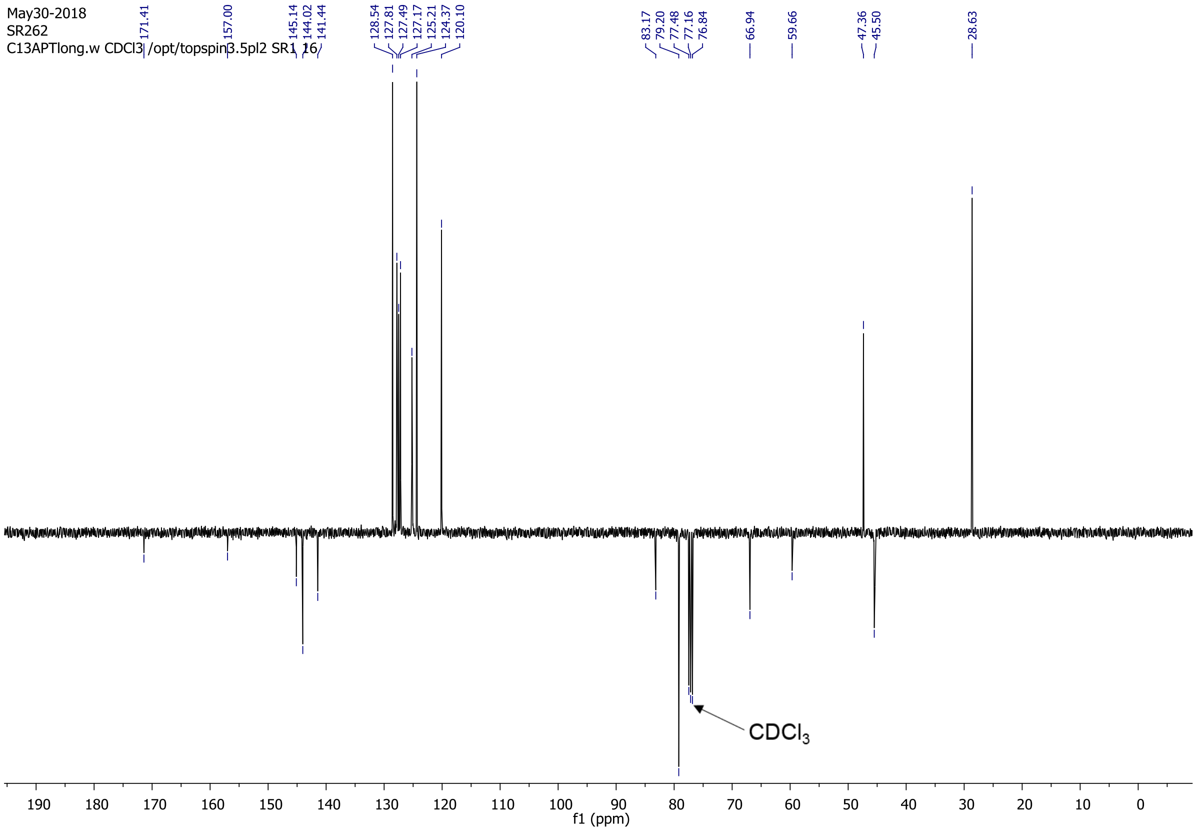

Cyclo(Leu-GOx-Gly-Trp(Boc)) (18)

^{1}H NMR (500 MHz, DMSO-*d6*)

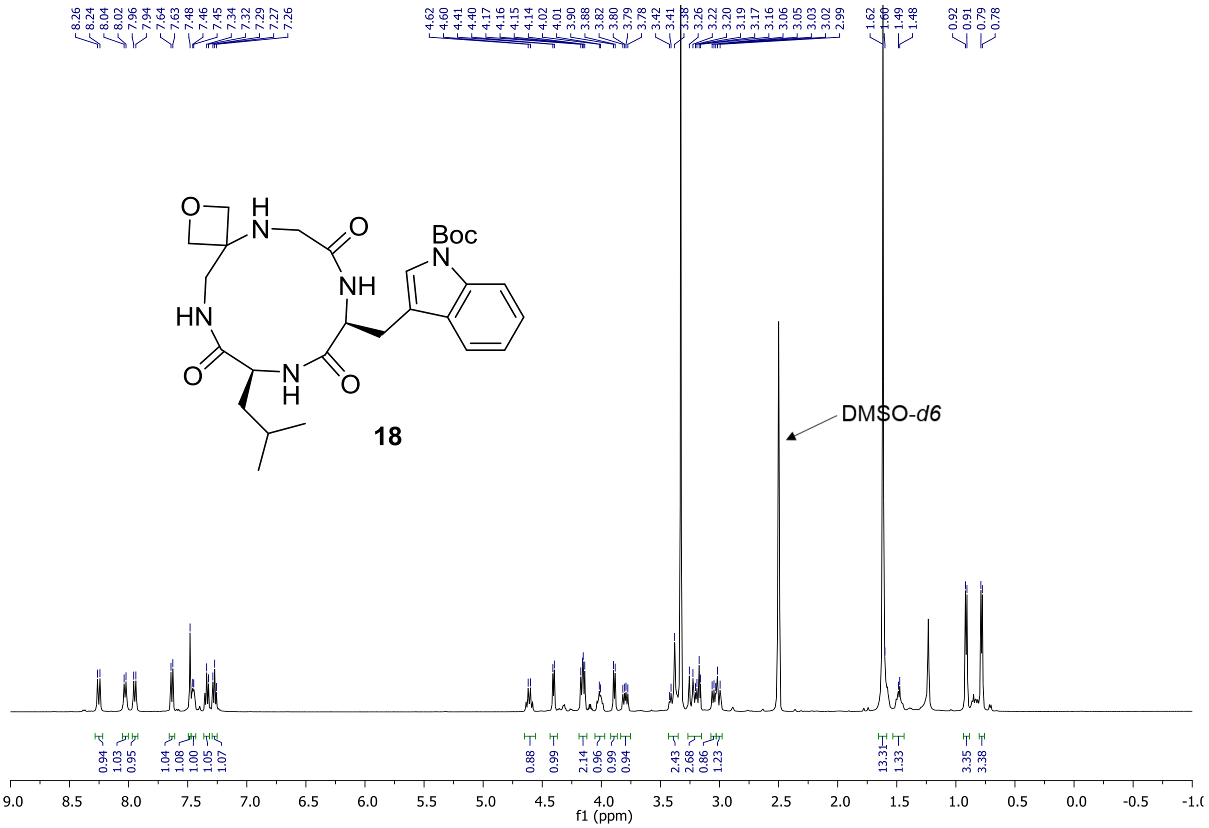

^{13}C NMR (126 MHz, DMSO-*d6*)

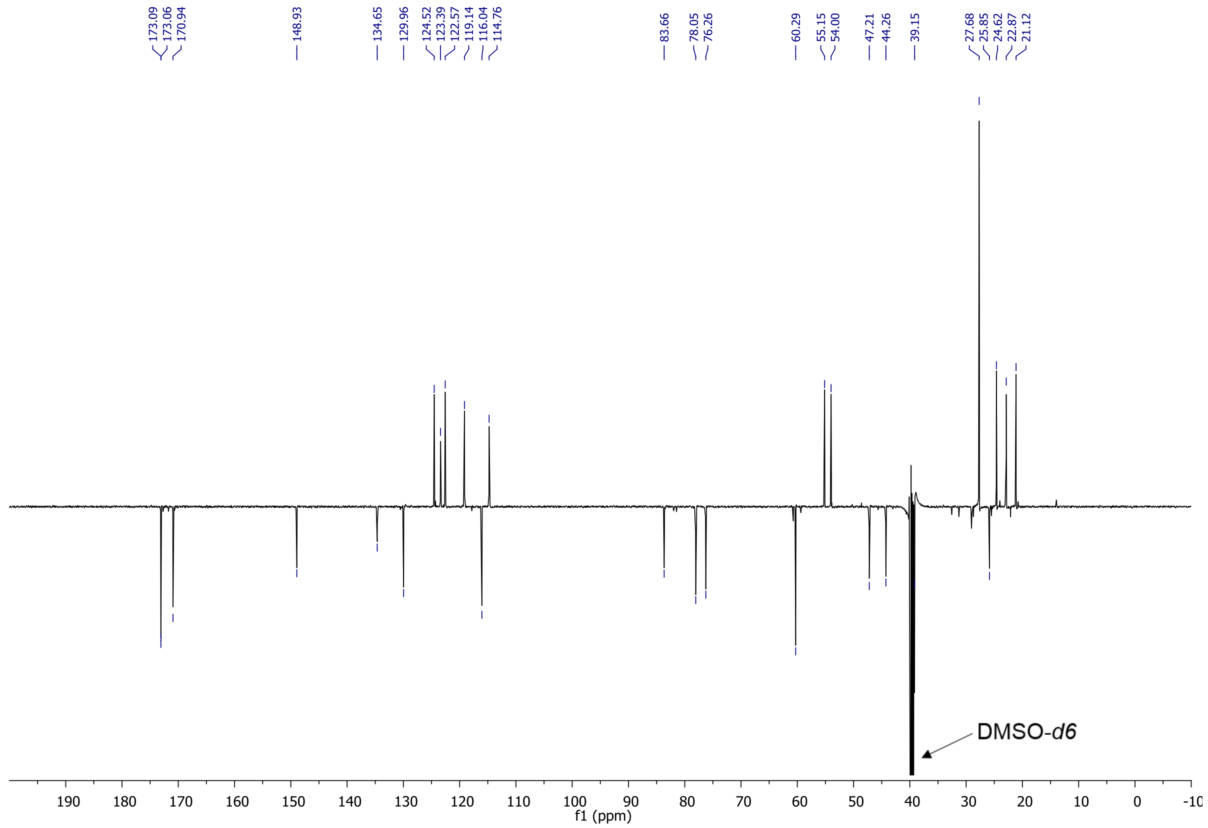

Fmoc-Arg(Pbf)-OCumyl (123)

^{1}H NMR (400 MHz, $CDCl_3$)

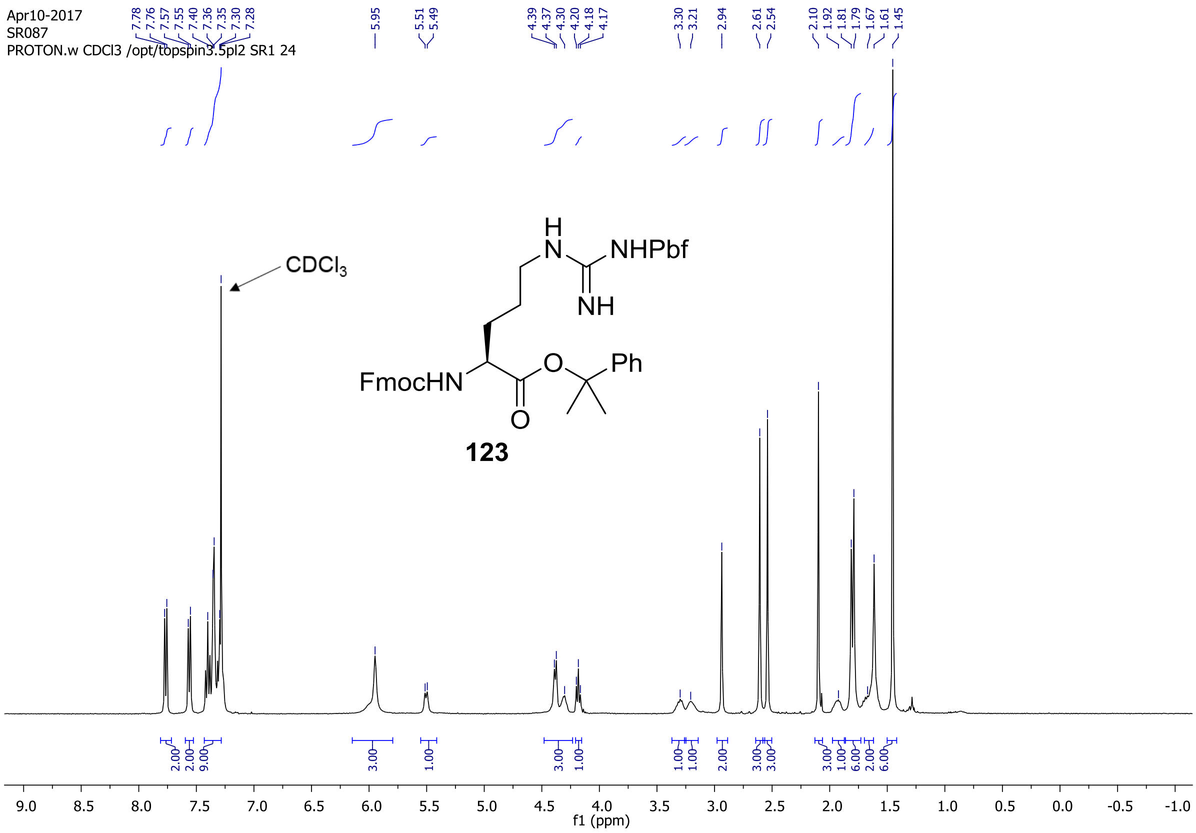

^{13}C NMR (101 MHz, $CDCl_3$)

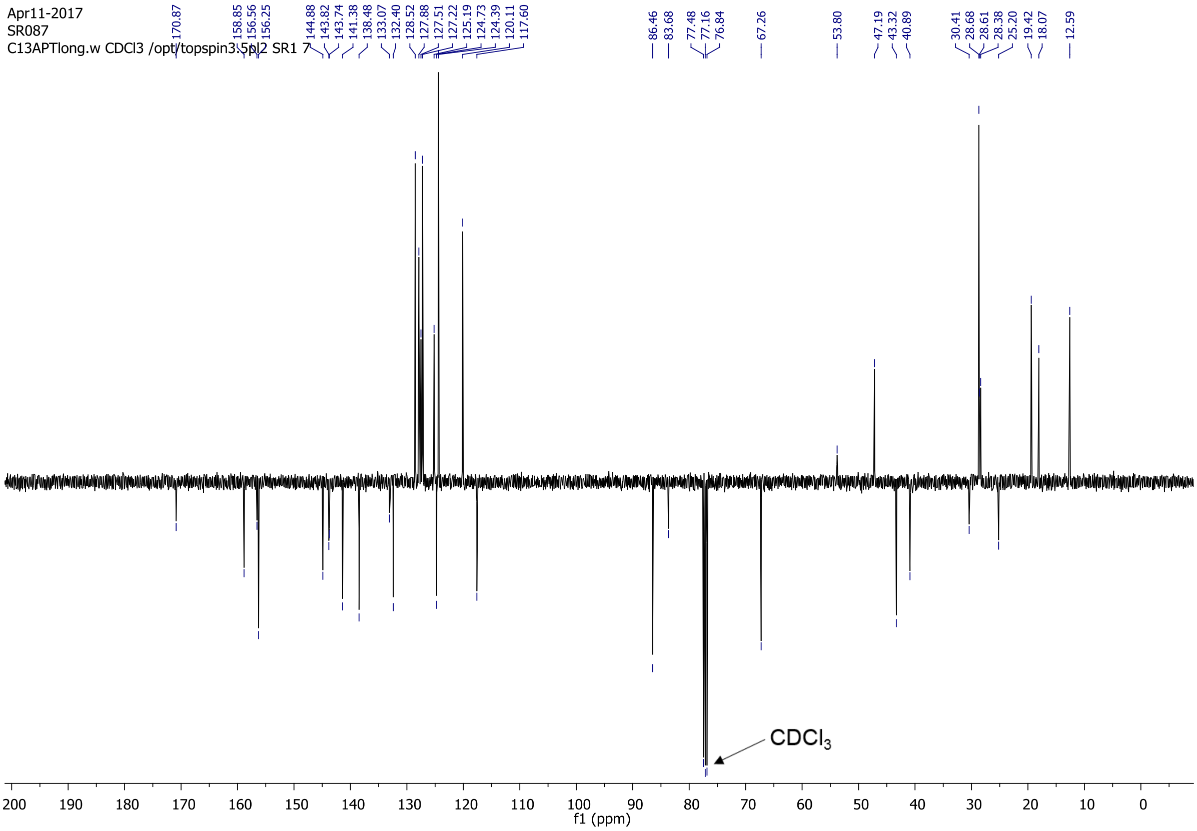

NO_2-GOx-Arg(Pbf)-OCumyl (124)

^{1}H NMR (400 MHz, $CDCl_3$)

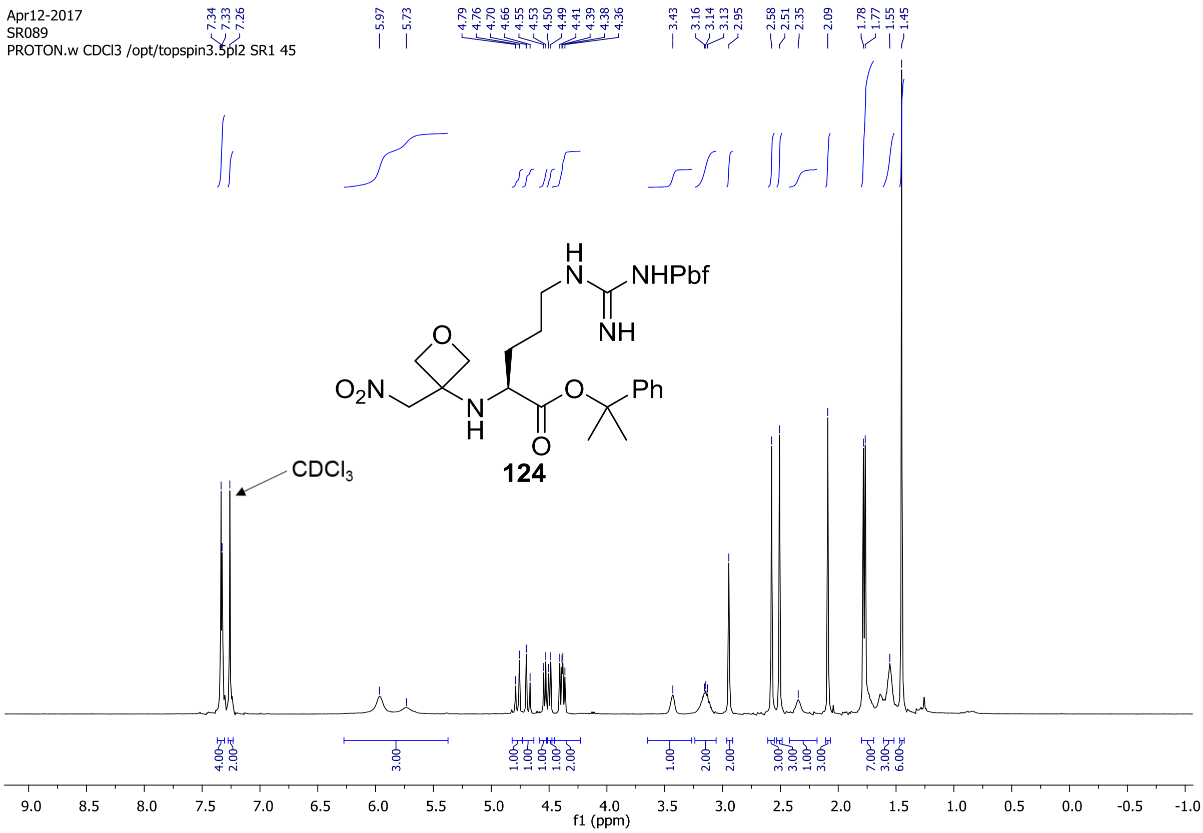

^{13}C NMR (101 MHz, $CDCl_3$)

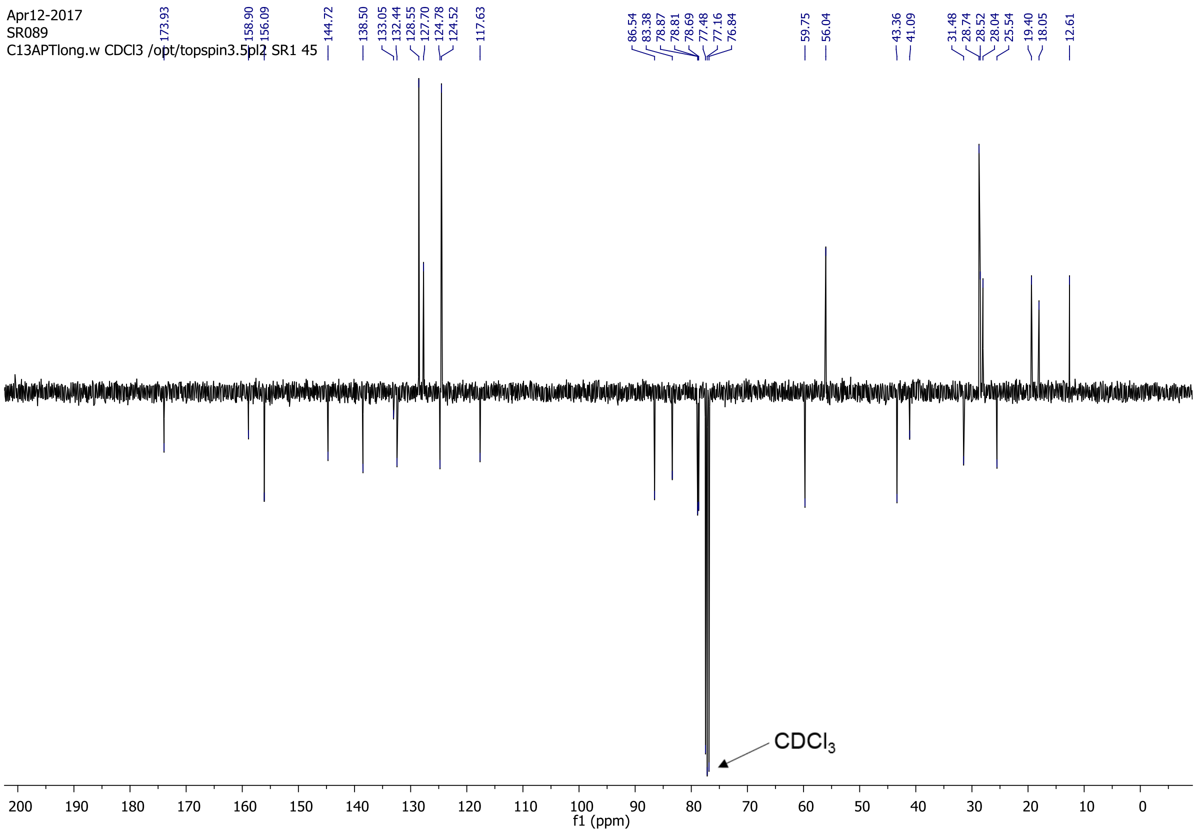

Fmoc-GOx-Arg(Pbf)-OCumyl (125)

^{1}H NMR (600 MHz, $CDCl_3$ @ 323 K)

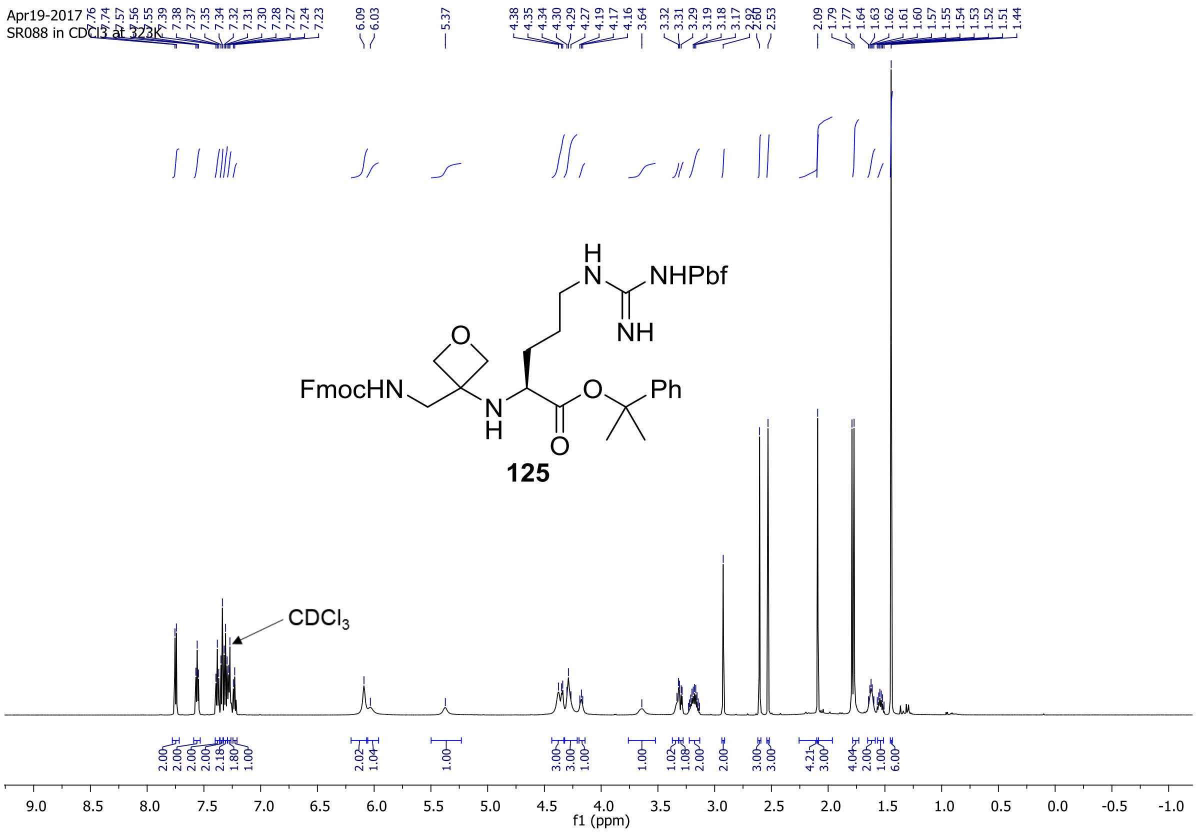

^{13}C NMR (151 MHz, $CDCl_3$ @ 323 K)

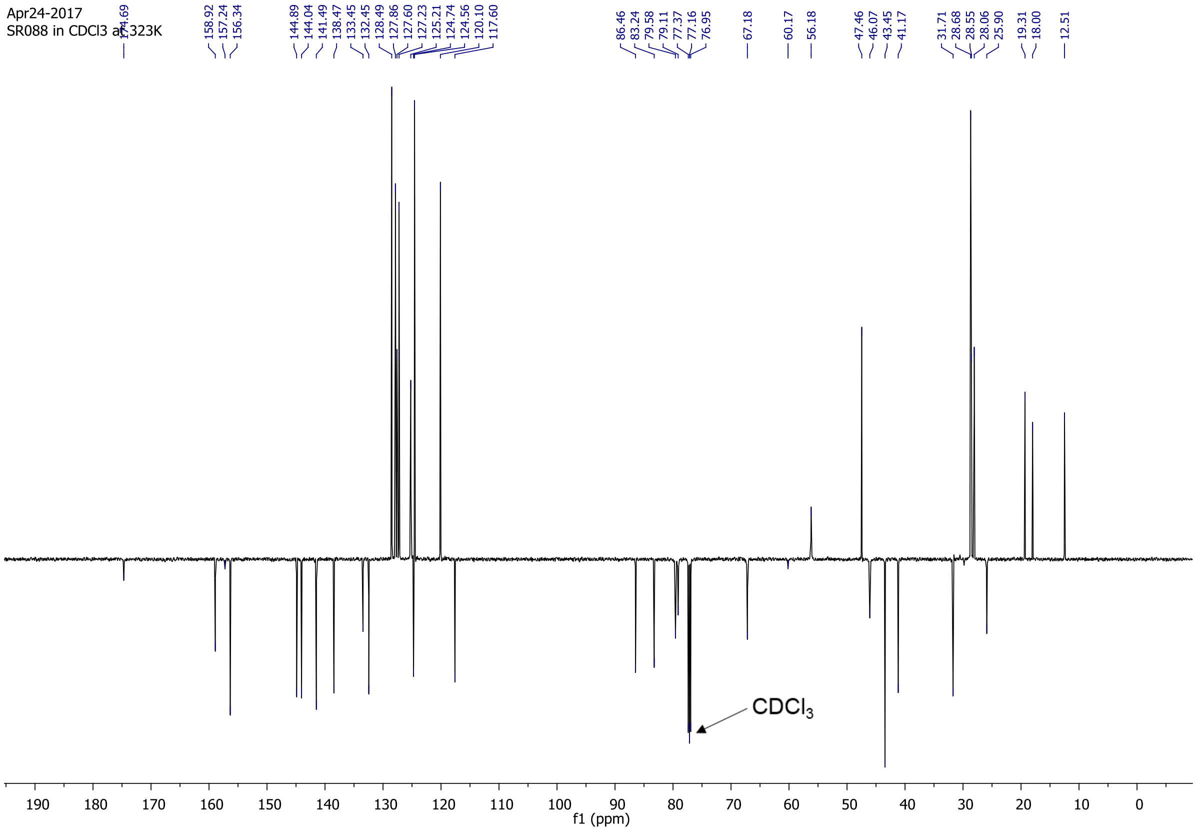

Fmoc-GOx-Arg(Pbf)-Cys(Trt)-O*t*Bu (126)

^{1}H NMR (500 MHz, $CDCl_3$)

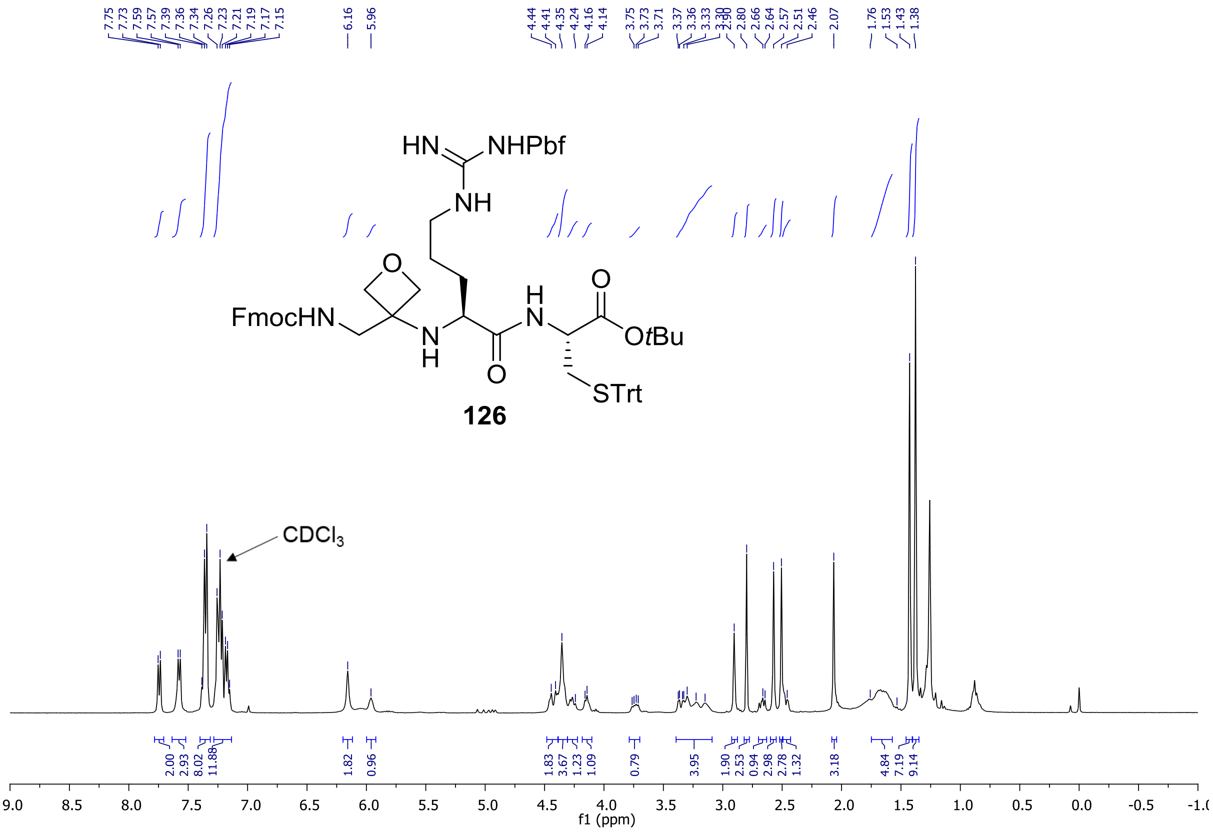

^{13}C NMR (126 MHz, $CDCl_3$)

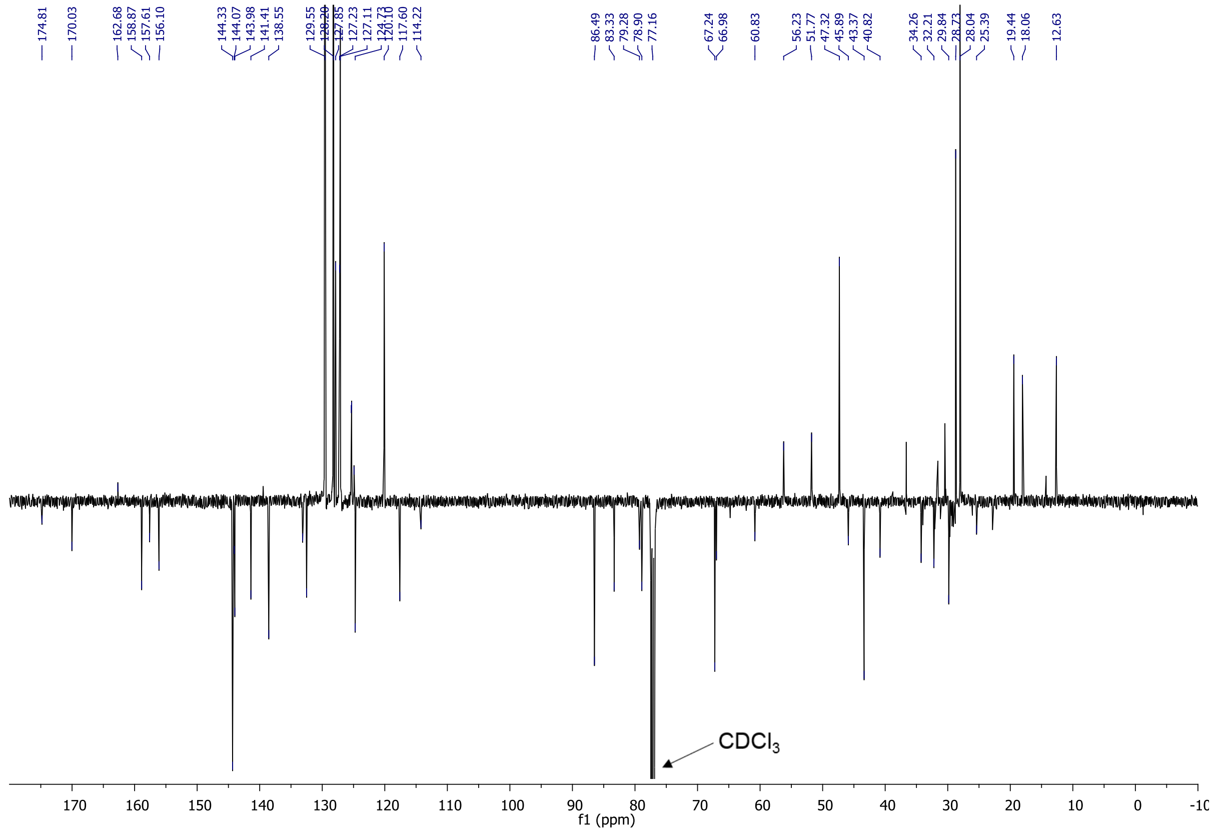

Fmoc-Asn(Trt)-GOx-Arg(Pbf)-Cys(Trt)-O*t*Bu (127)

^{1}H NMR (500 MHz, $CDCl_3$)

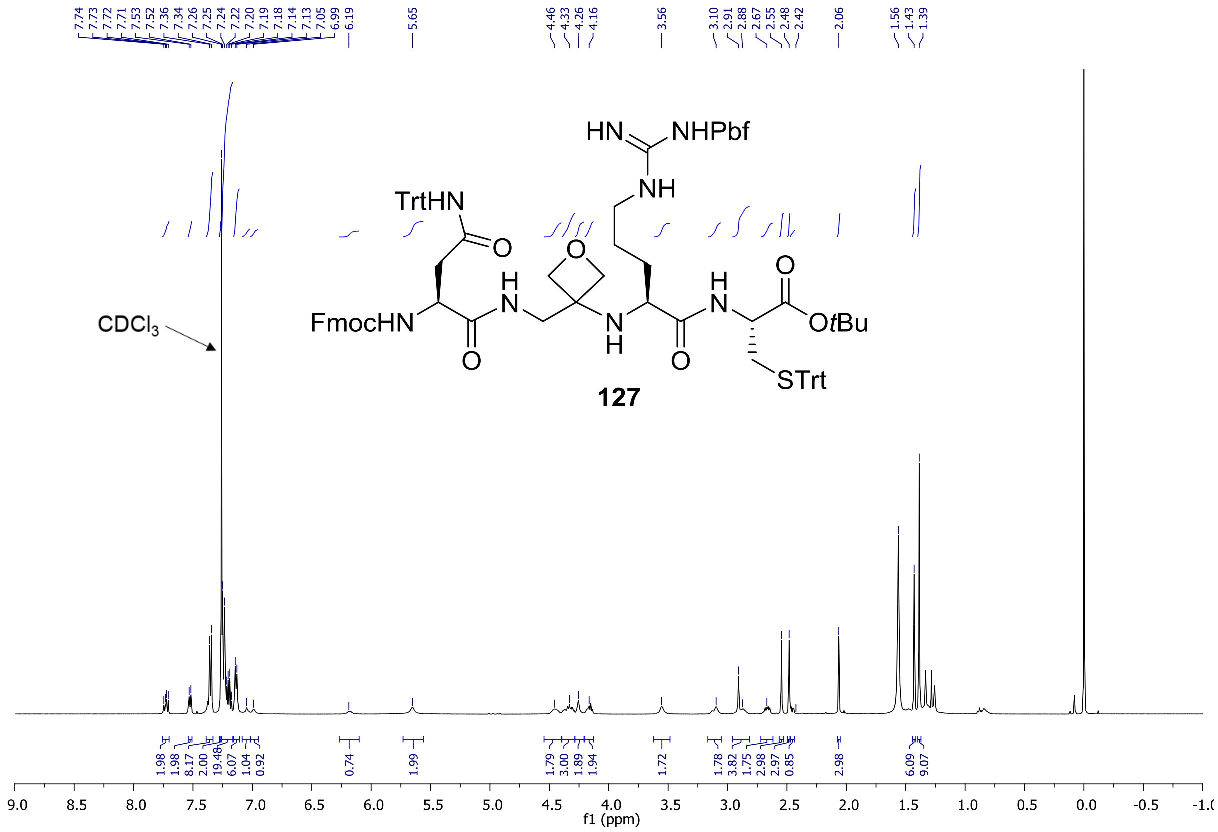

^{13}C NMR (126 MHz, $CDCl_3$)

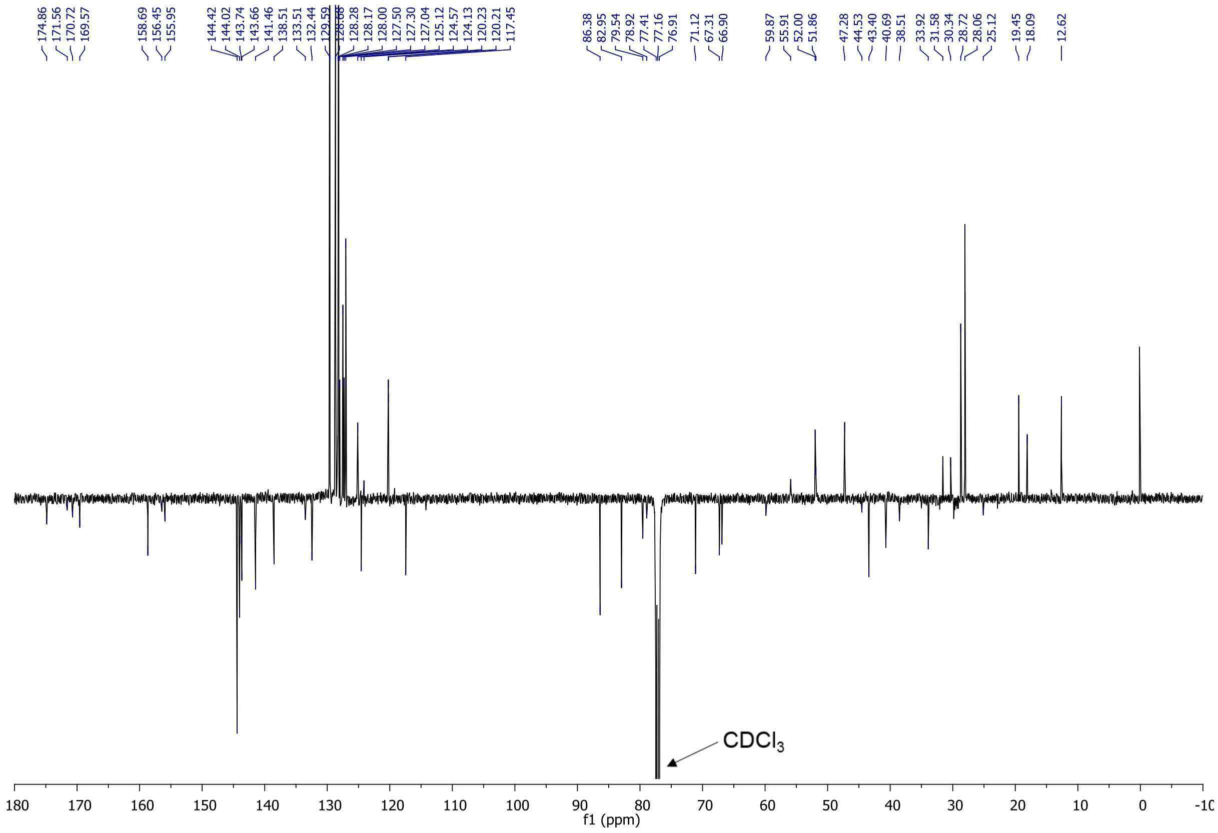

Fmoc-Cys(Trt)-Asn(Trt)-GOx-Arg(Pbf)-Cys(Trt)-O*t*Bu (2)

^{1}H NMR (500 MHz, CDCl3)

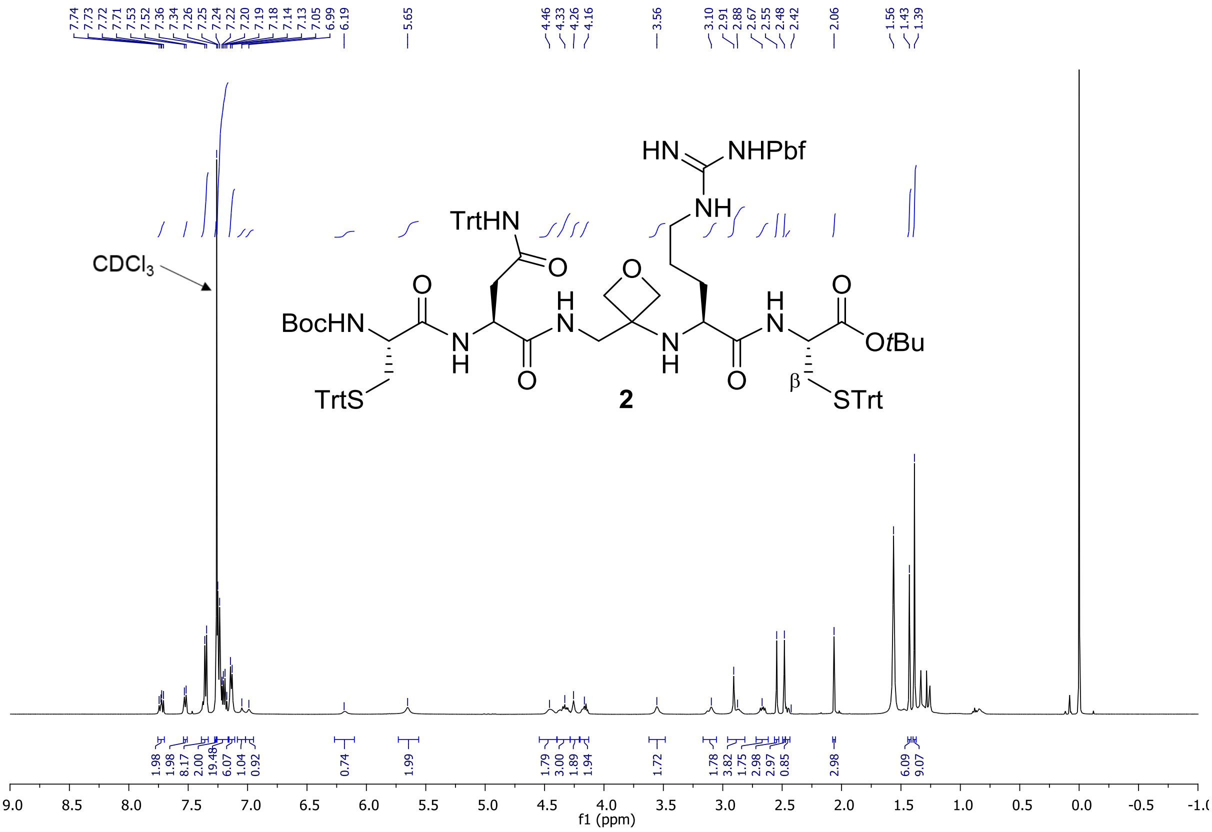

^{13}C NMR (126 MHz, CDCl3)

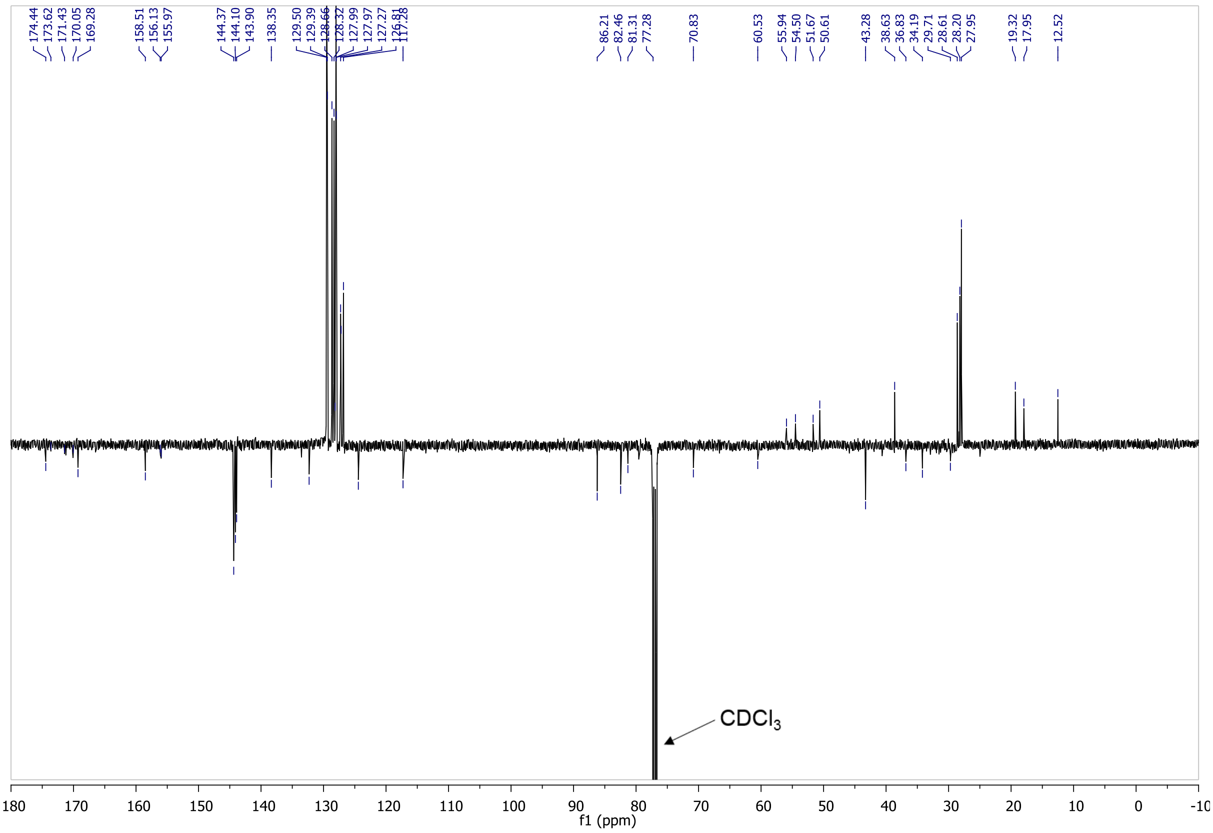

Cyclo(H-Cys-Asn-GOx-Arg-Cys-OH (22)

^{1}H NMR (500 MHz, D_2O @ 323 K)

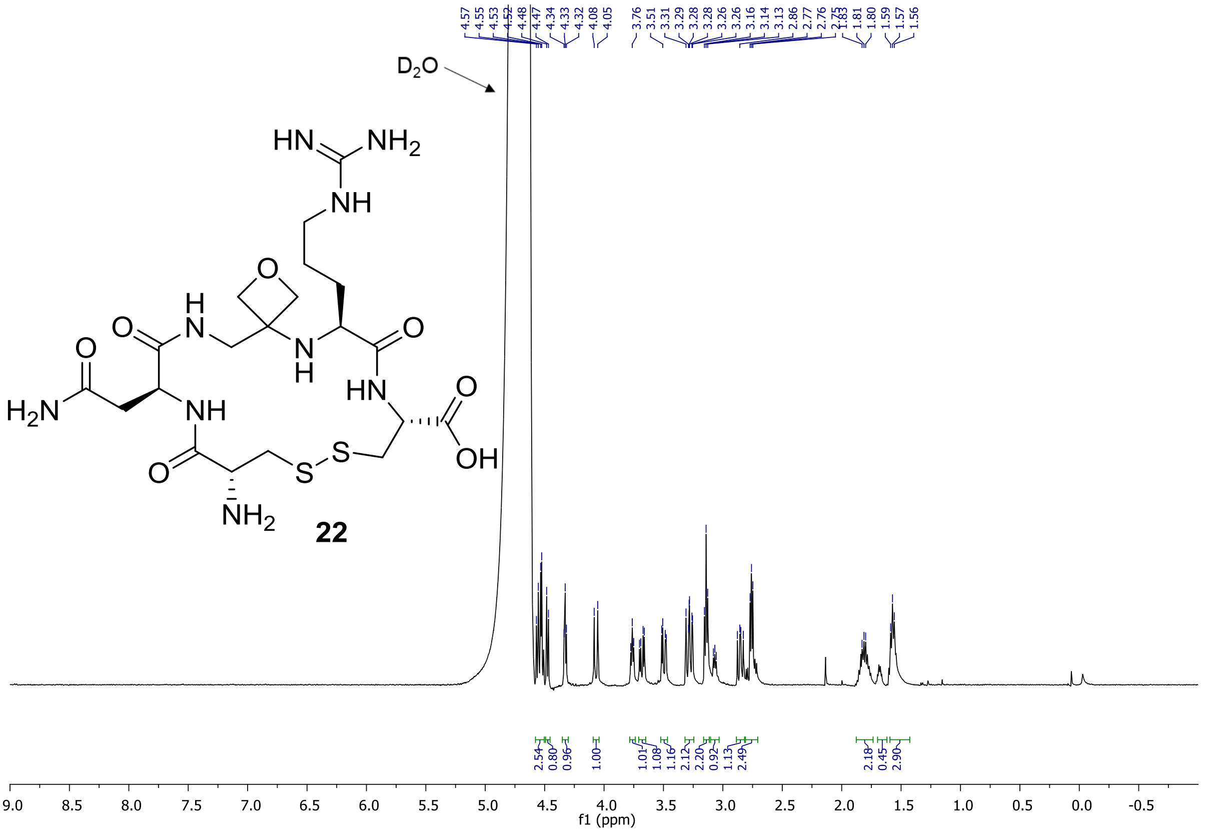

^{13}C NMR (126 MHz, D_2O @ 323 K)

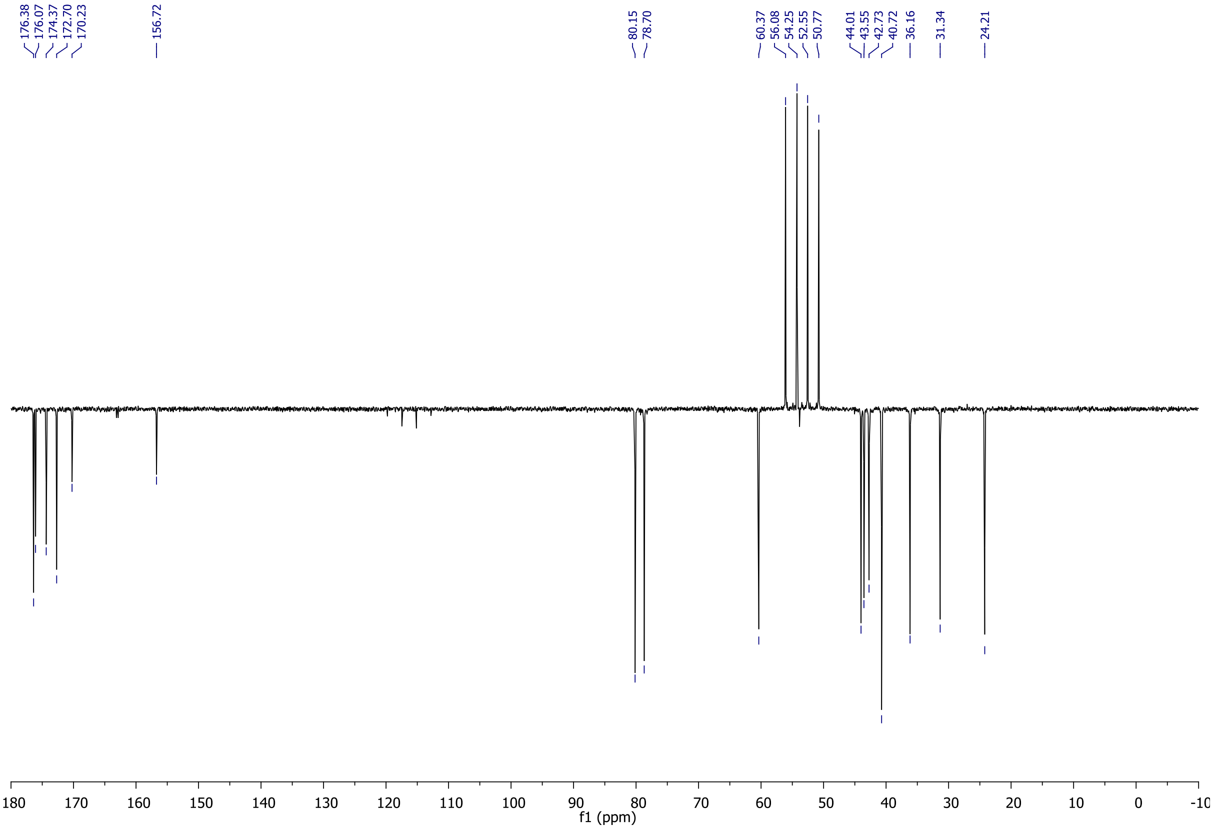

Fmoc-Cys(Trt)-O*t*Bu (128)

^{1}H NMR (400 MHz, $CDCl_3$)

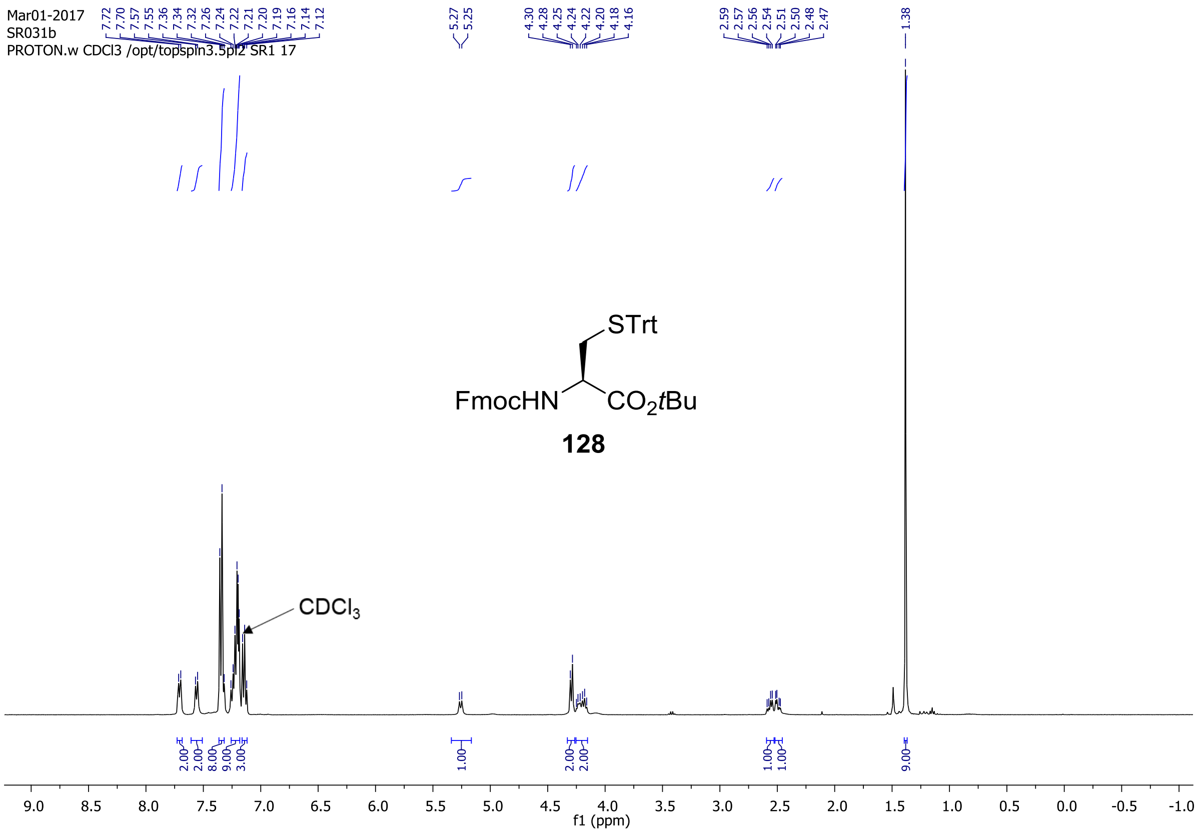

^{13}C NMR (101 MHz, $CDCl_3$)

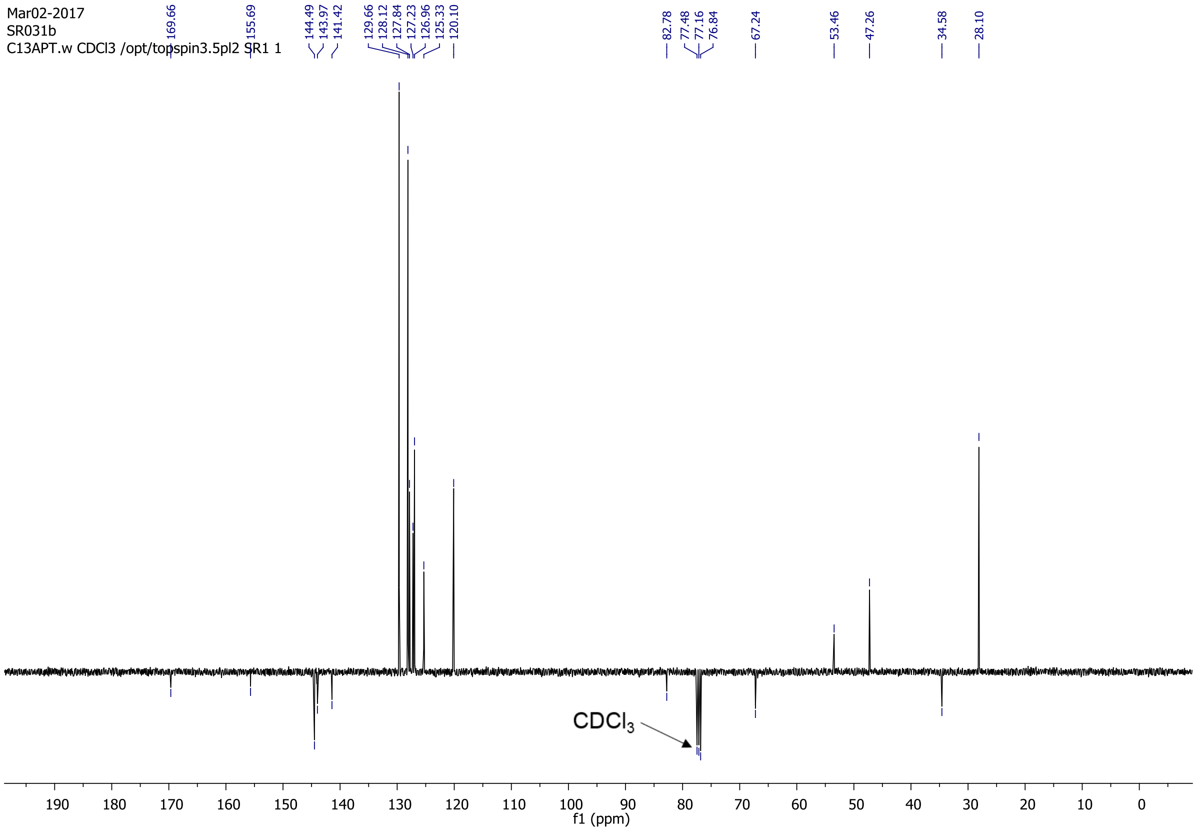

NO_2-GOx-Cys(Trt)-O*t*Bu (129)

^{1}H NMR (400 MHz, $CDCl_3$)

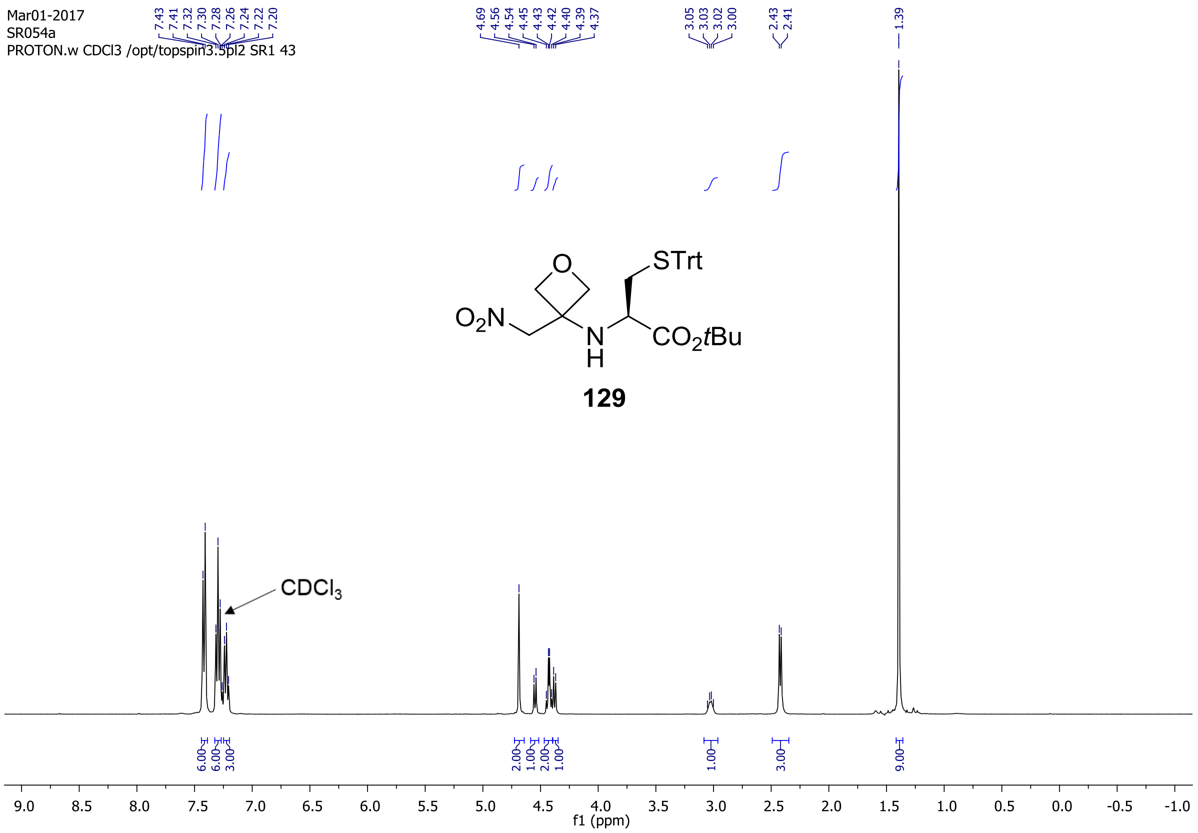

^{13}C NMR (101 MHz, $CDCl_3$)

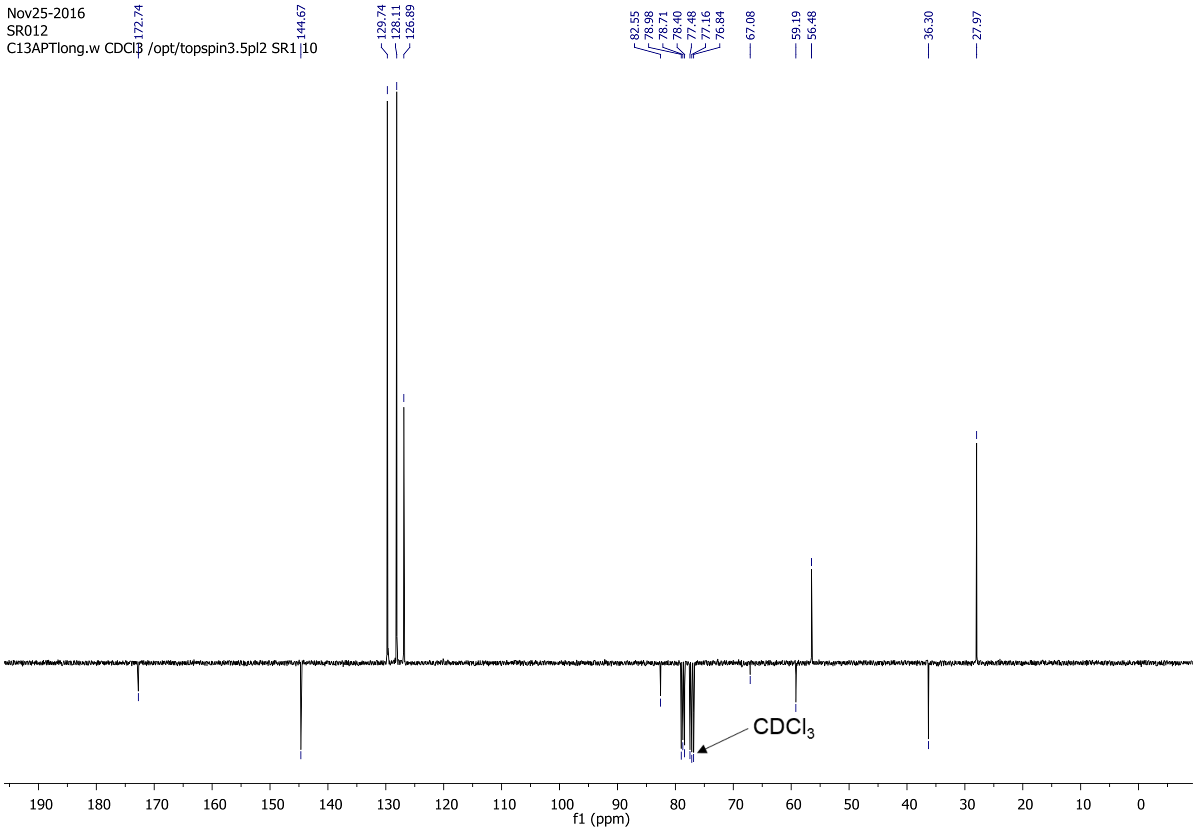

Boc-Cys(Trt)-GOx-Cys(Trt)-O*t*Bu (130)

^{1}H NMR (600 MHz, $CDCl_3$ @ 323 K)

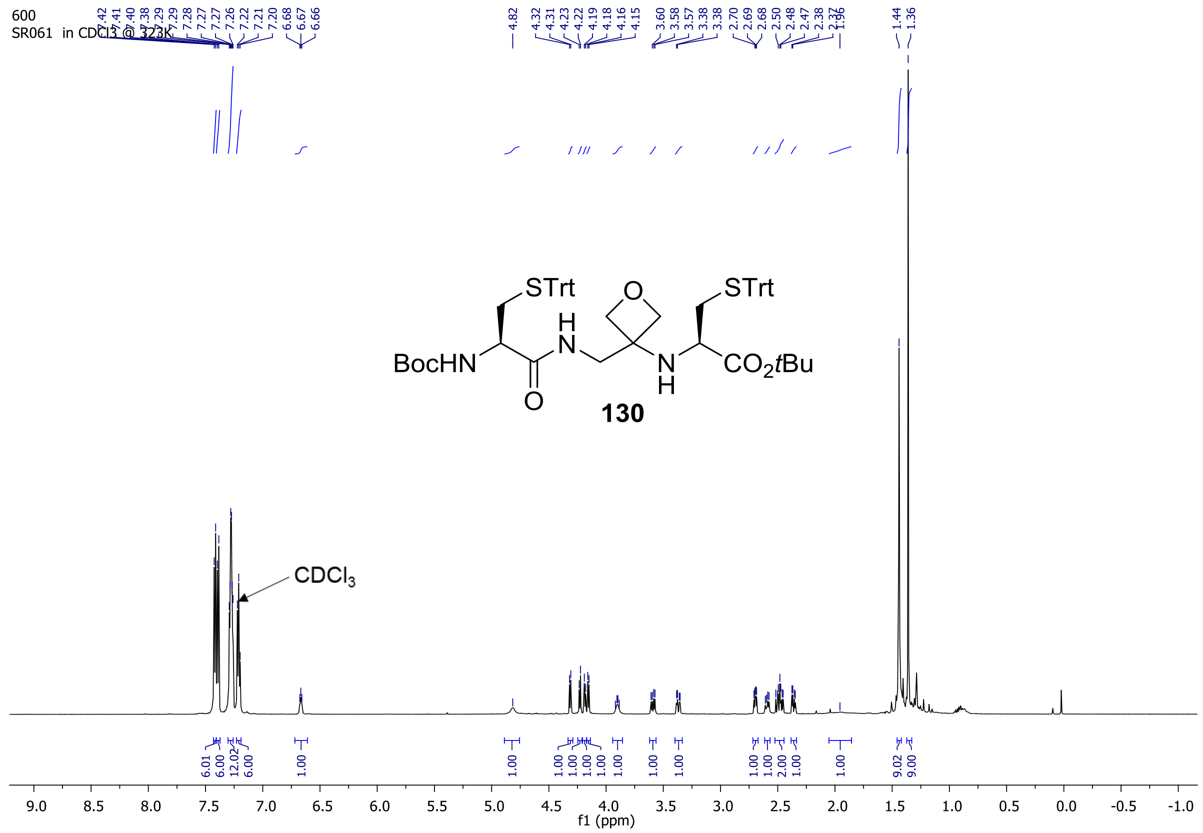

^{13}C NMR (151 MHz $CDCl_3$ @ 323 K)

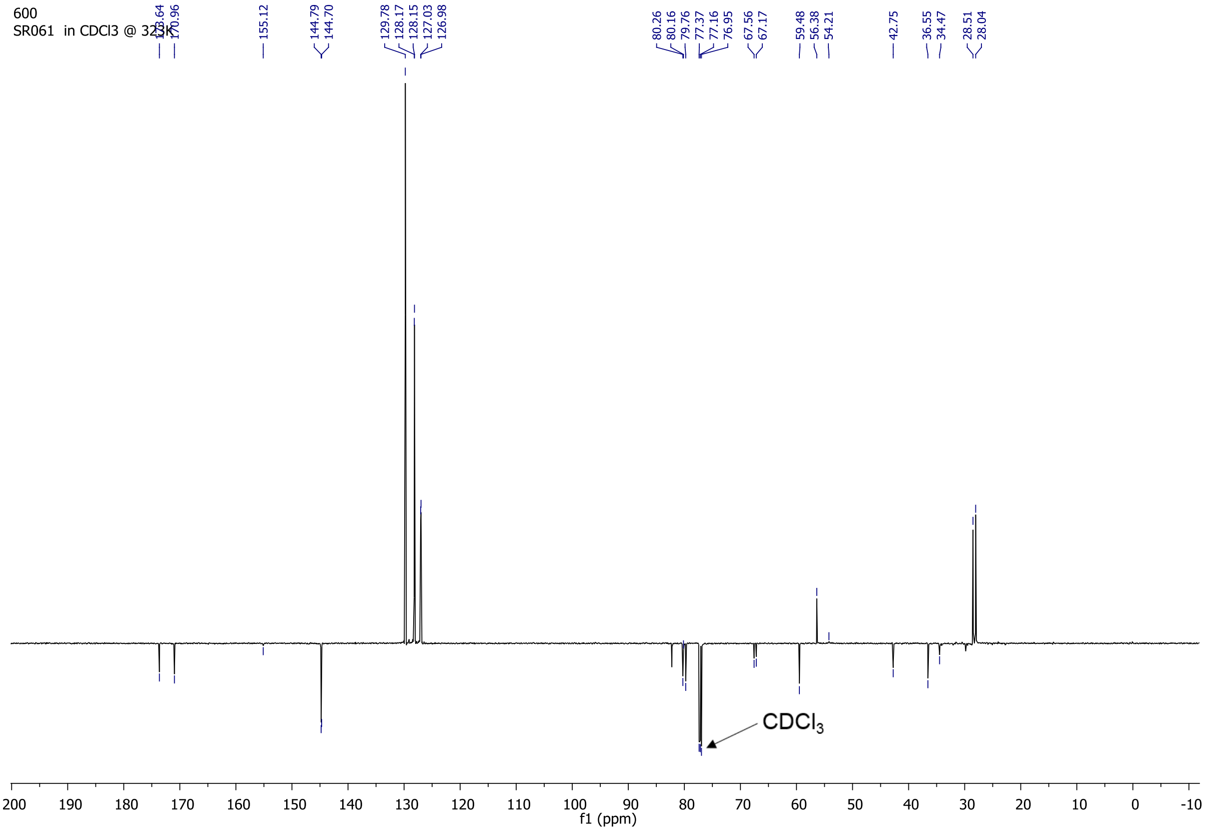

Cyclo(Boc-Cys-GOx-Cys-O*t*Bu) (23)

^{1}H NMR (600 MHz, CDCl$_3$ @ 323 K)

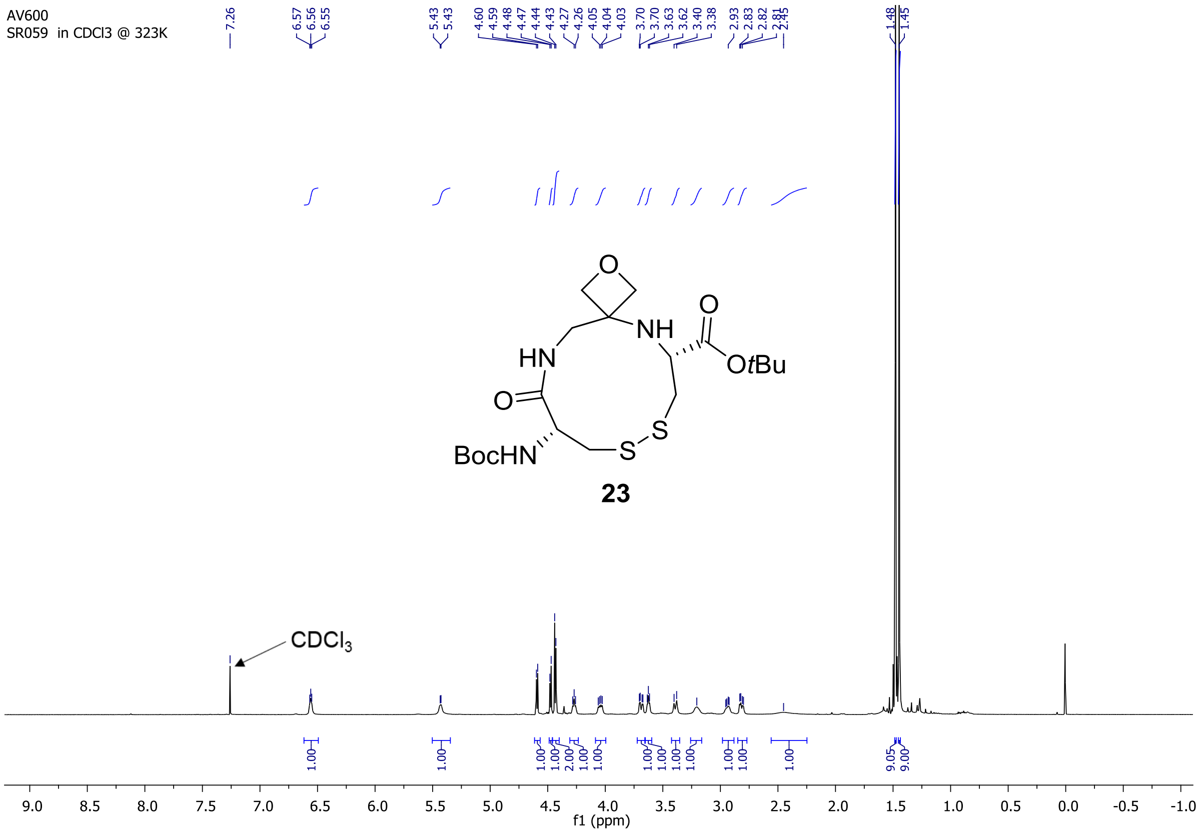

^{13}C NMR (151 MHz CDCl$_3$ @ 323 K)

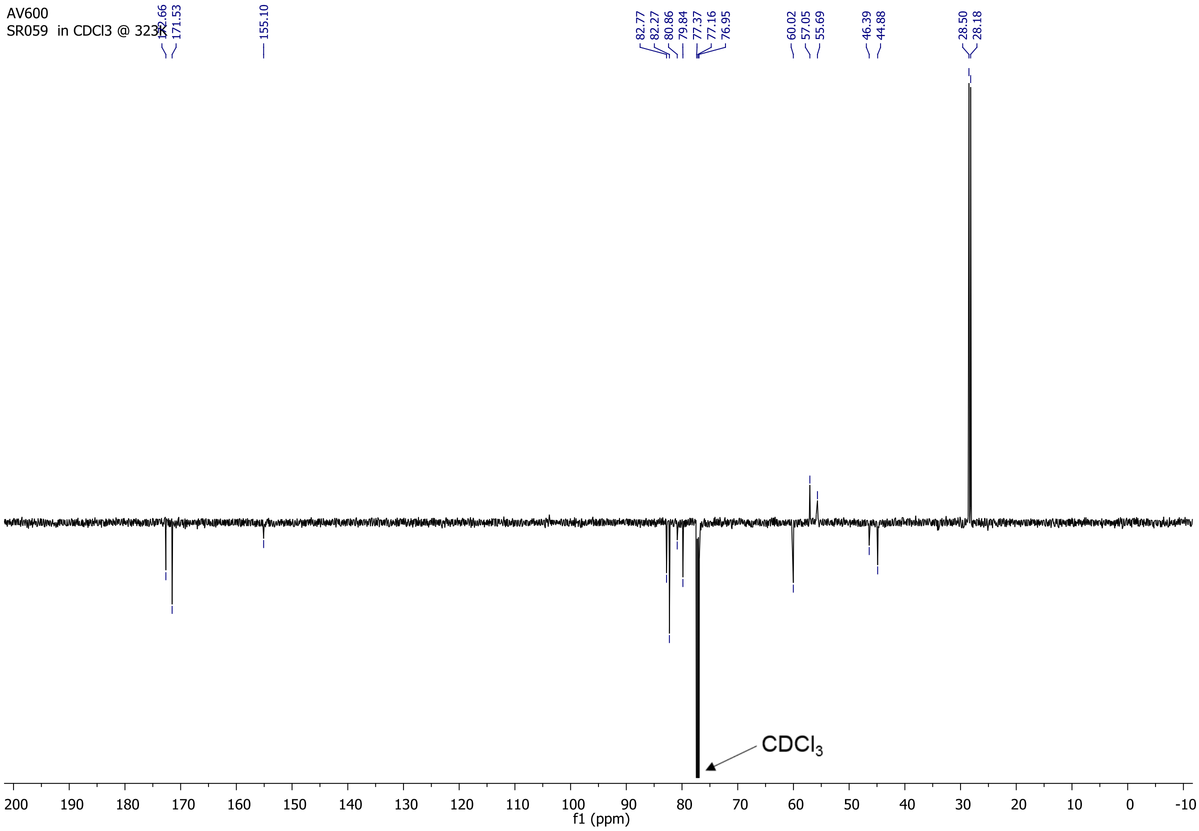

Fmoc-Pro-GOx-Cys(Trt)-O*t*Bu (131)

^{1}H NMR (600 MHz, toluene-*d8* @ 353 K)

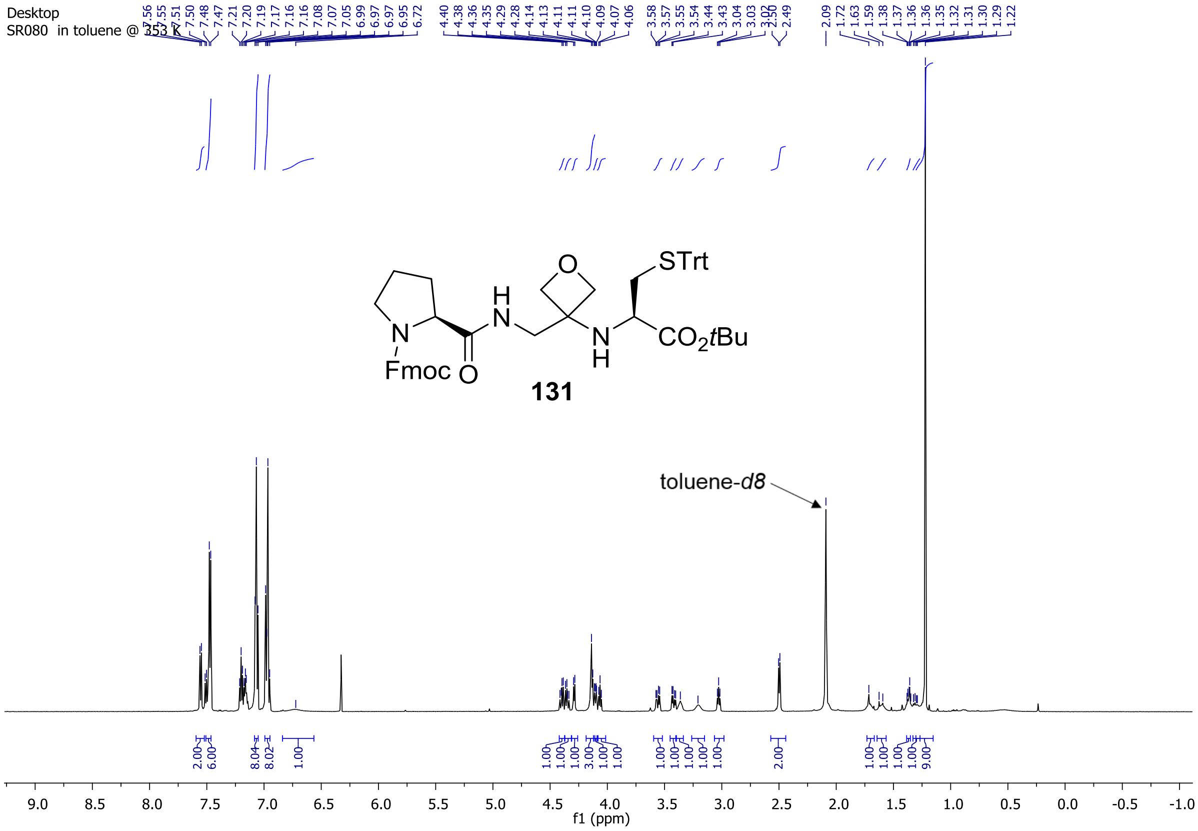

^{13}C NMR (151 MHz, toluene-*d8* @ 353 K)

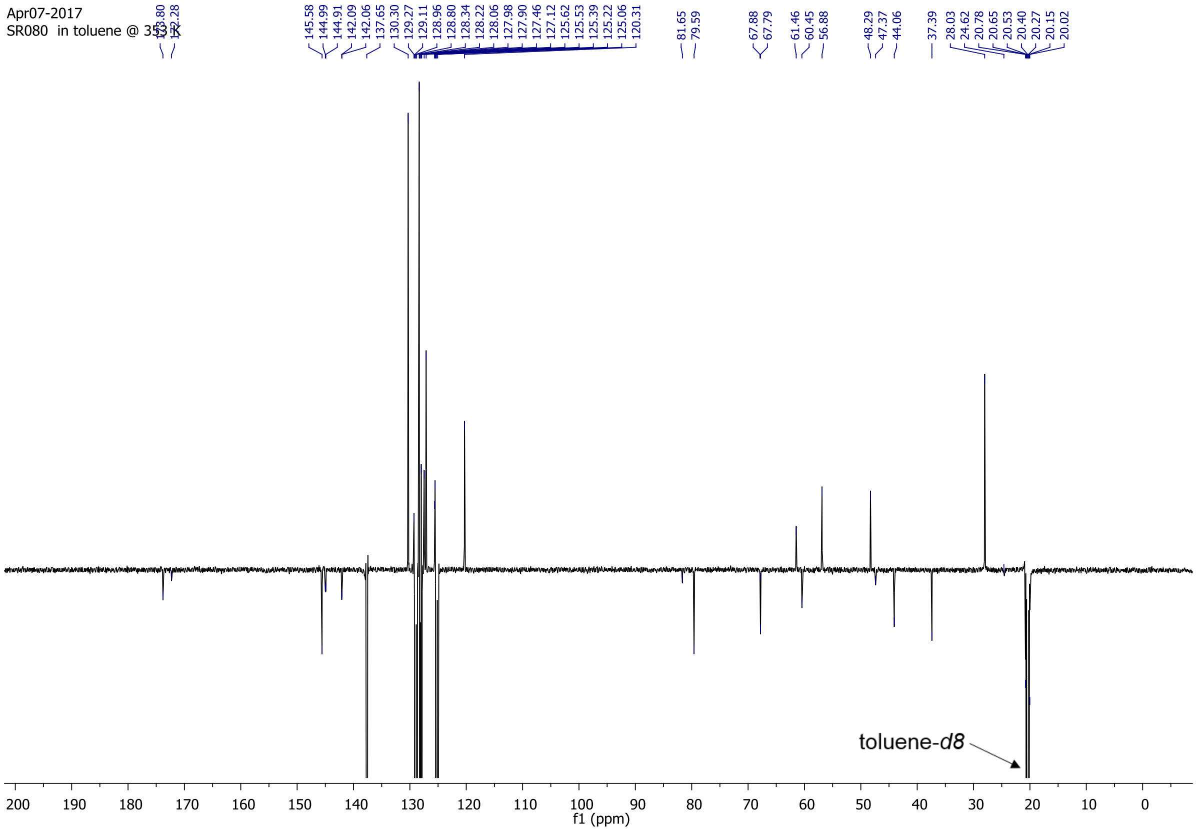

Boc-Cys(Trt)-Pro-GOx-Cys(Trt)-O*t*Bu (132)

^{1}H NMR (600 MHz, $CDCl_3$)

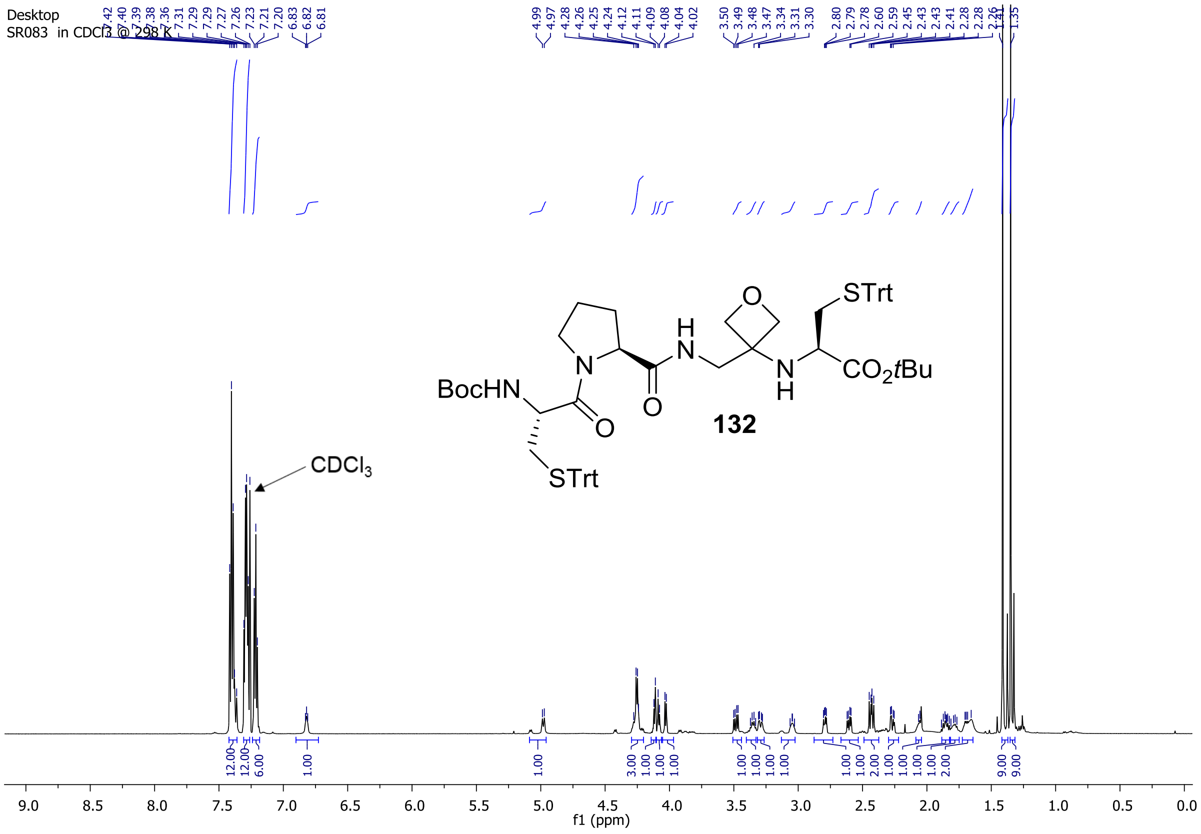

^{13}C NMR (126 MHz $CDCl_3$)

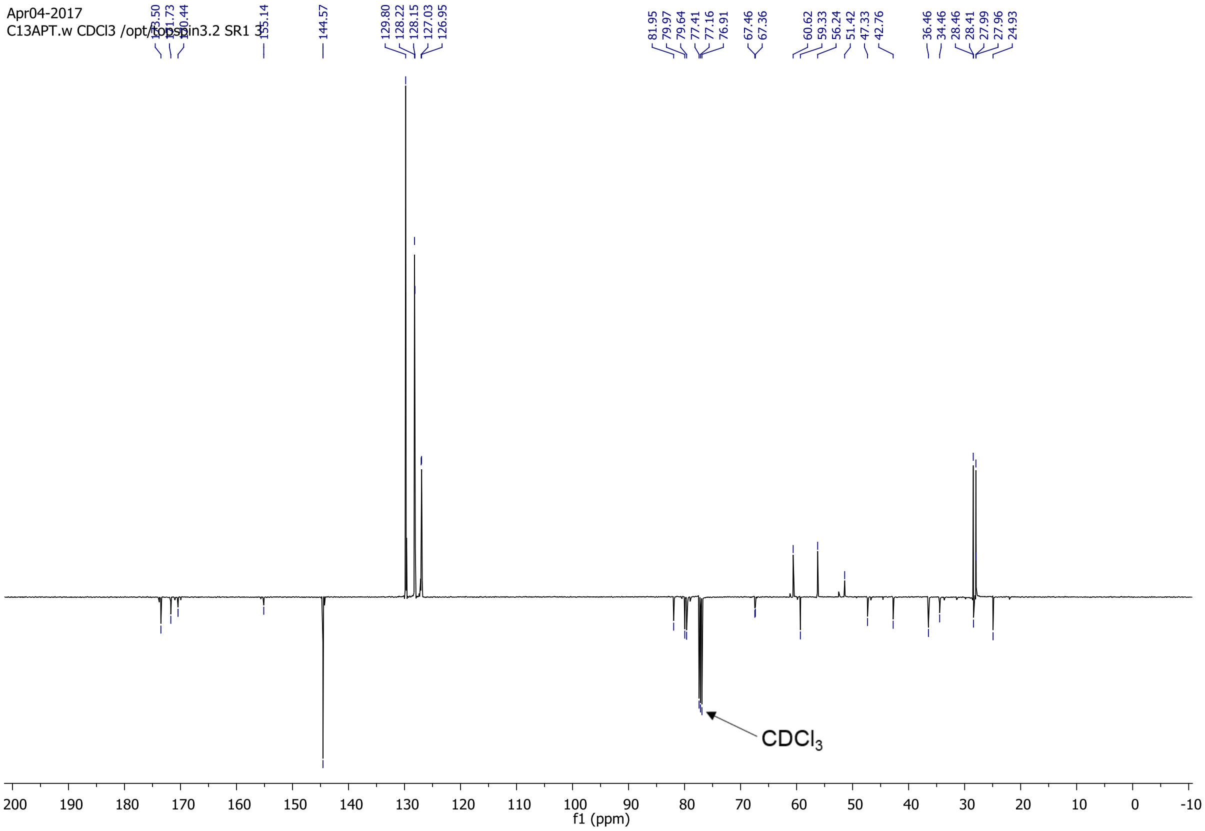

Cyclo(Boc-Cys-Pro-GOx-Cys-O*t*Bu) (24)

^{1}H NMR (400 MHz, CDCl$_3$)

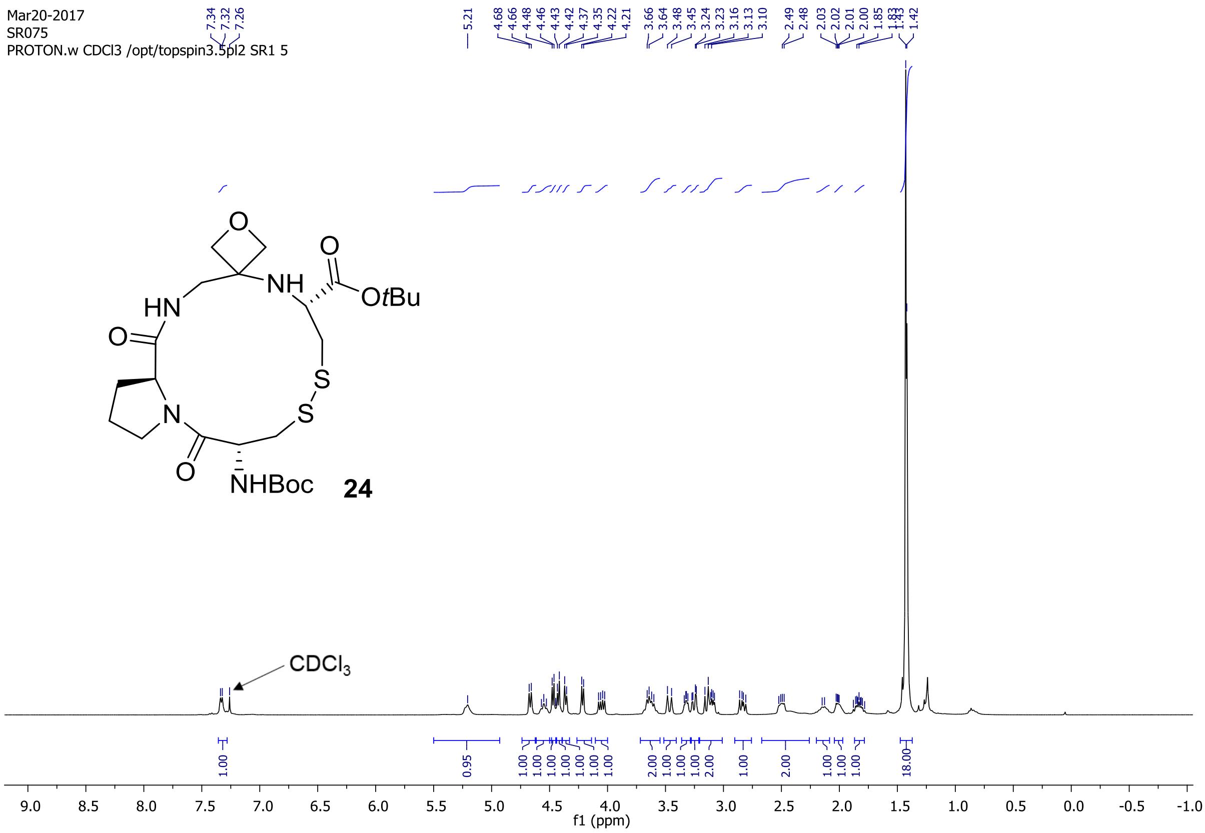

^{13}C NMR (101 MHz CDCl$_3$)

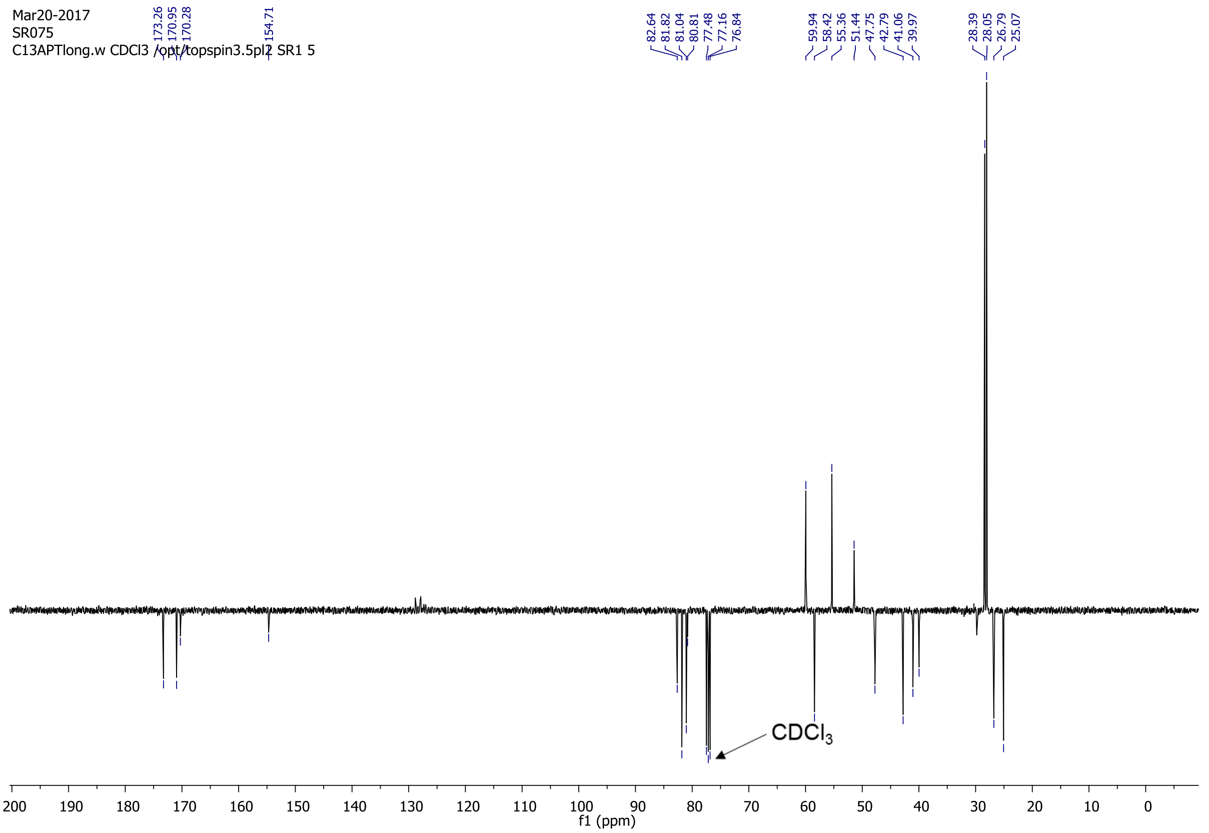

Fmoc-Gly-Cys(Trt)-O*t*Bu (133)

^{1}H NMR (400 MHz, $CDCl_3$)

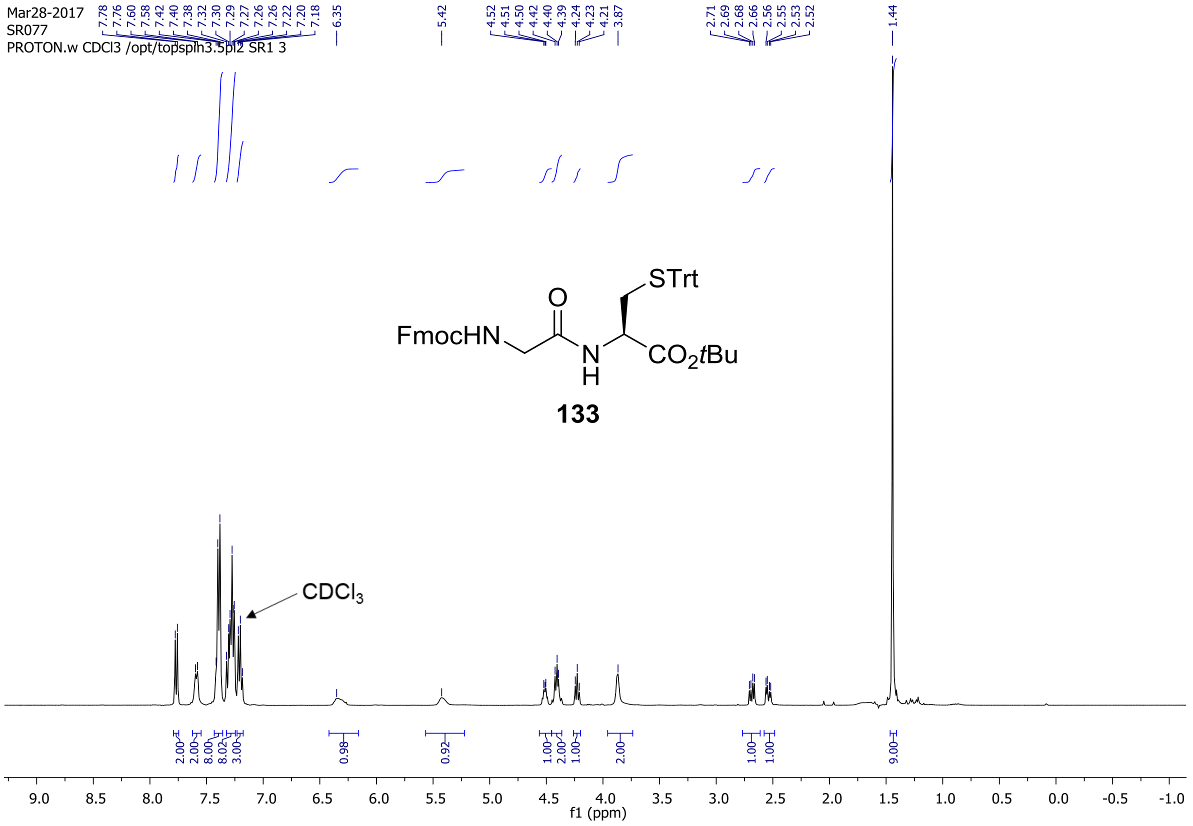

^{13}C NMR (101 MHz, $CDCl_3$)

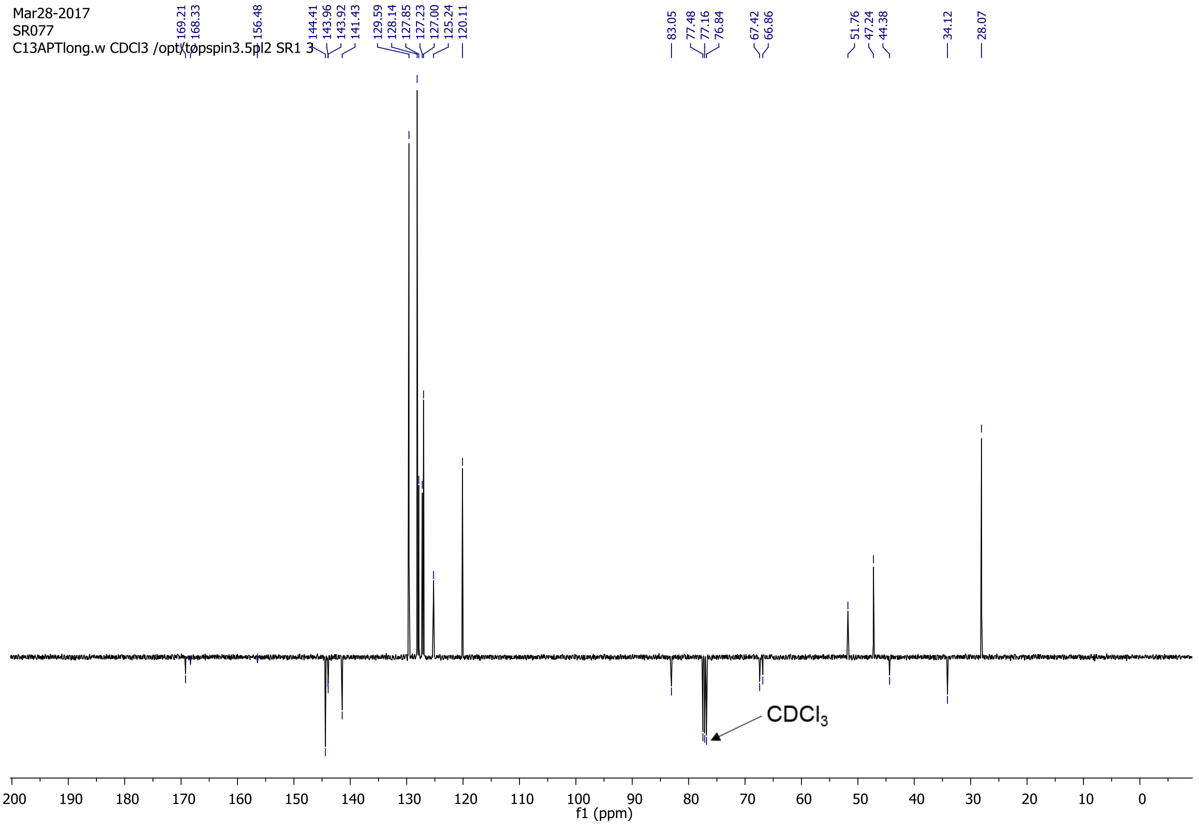

Fmoc-Pro-Gly-Cys(Trt)-O*t*Bu (134)

^{1}H NMR (600 MHz, toluene-*d8* @ 353 K)

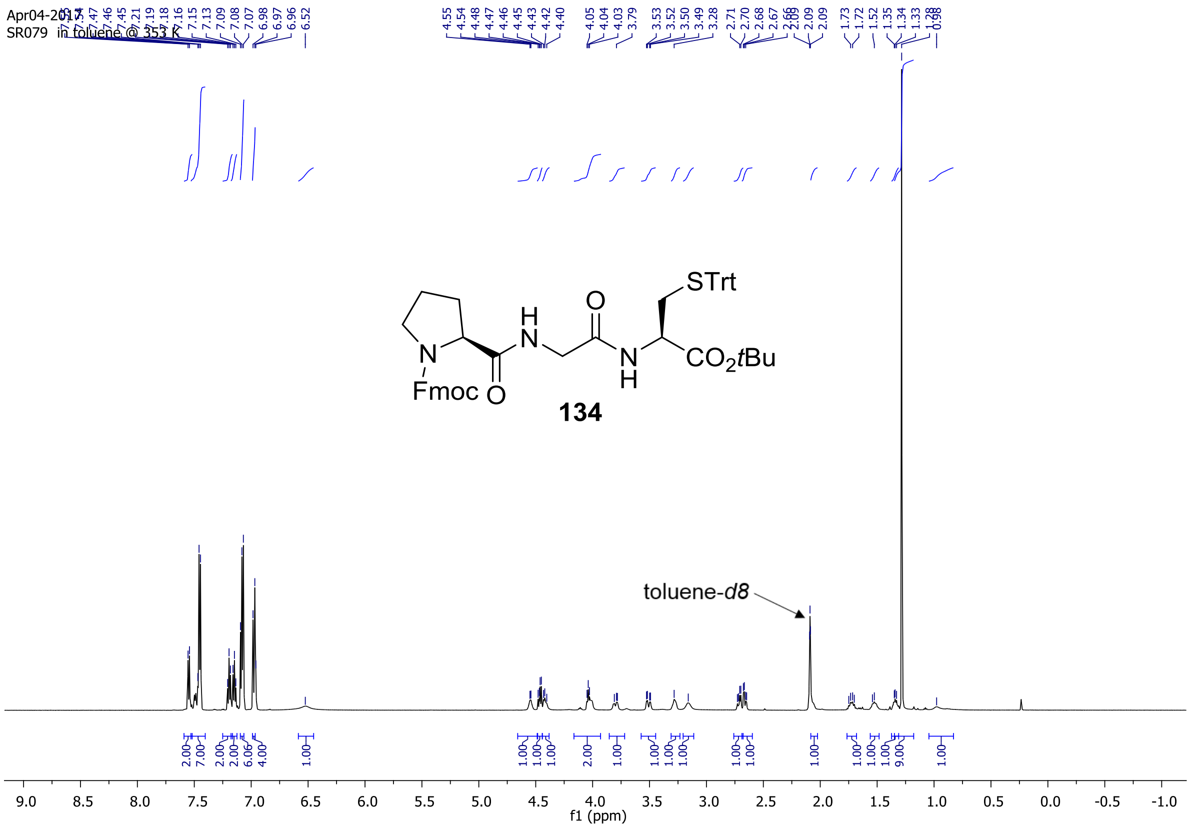

^{13}C NMR (151 MHz, toluene-*d8* @ 353 K)

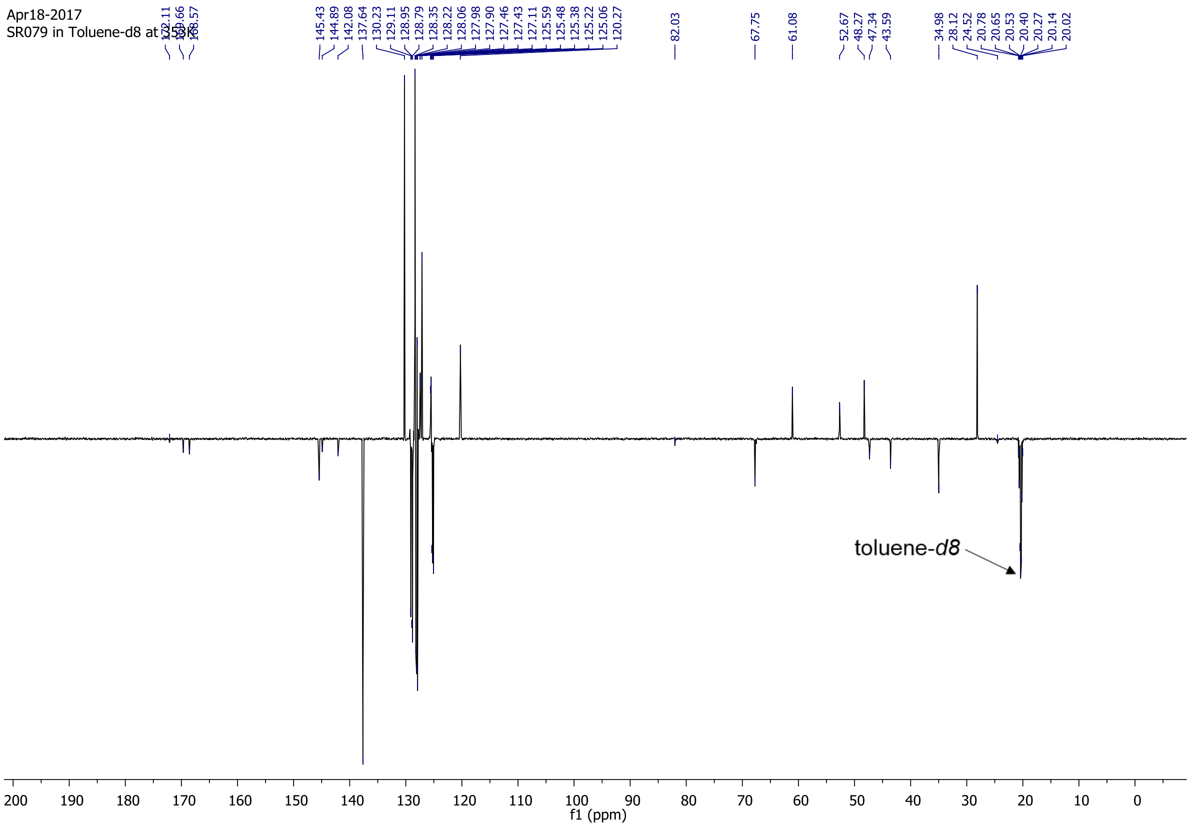

Boc-Cys(Trt)-Pro-Gly-Cys(Trt)-O*t*Bu (135)

^{1}H NMR (600 MHz, $CDCl_3$ @ 313 K)

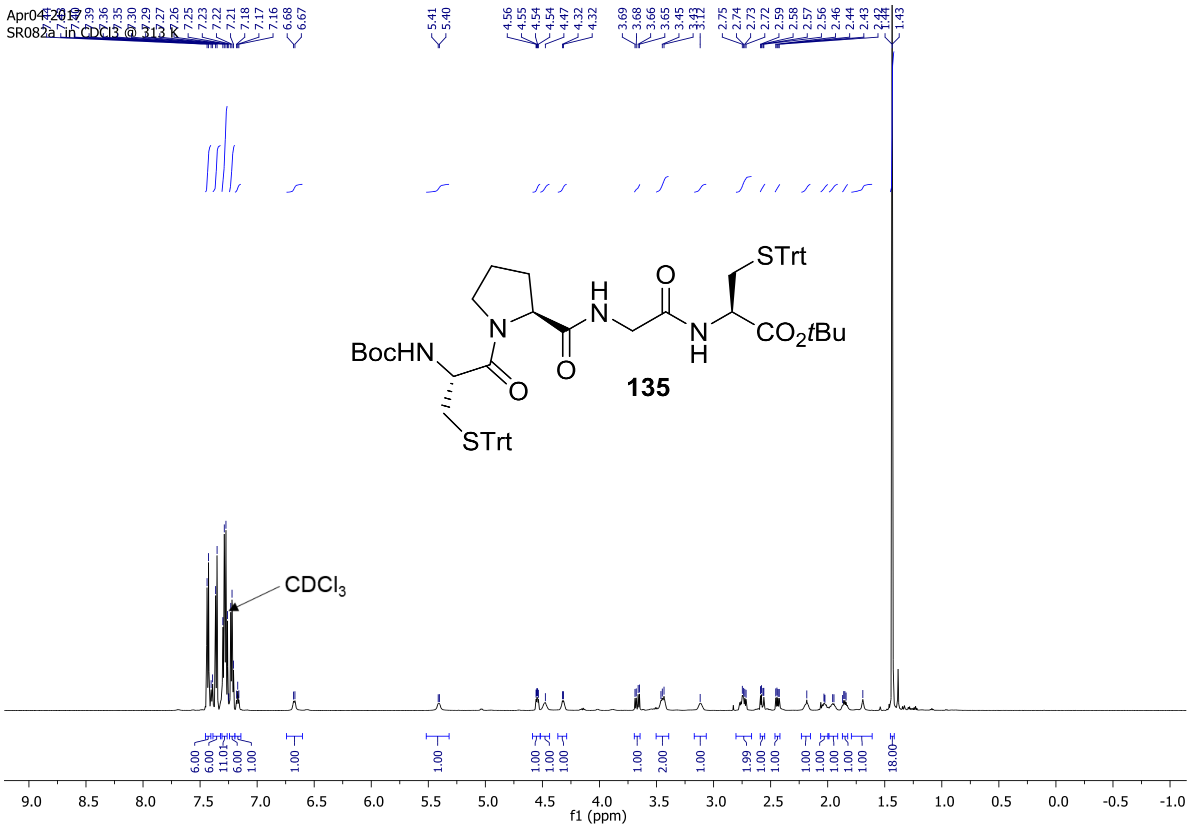

^{13}C NMR (151 MHz, $CDCl_3$ @ 313 K)

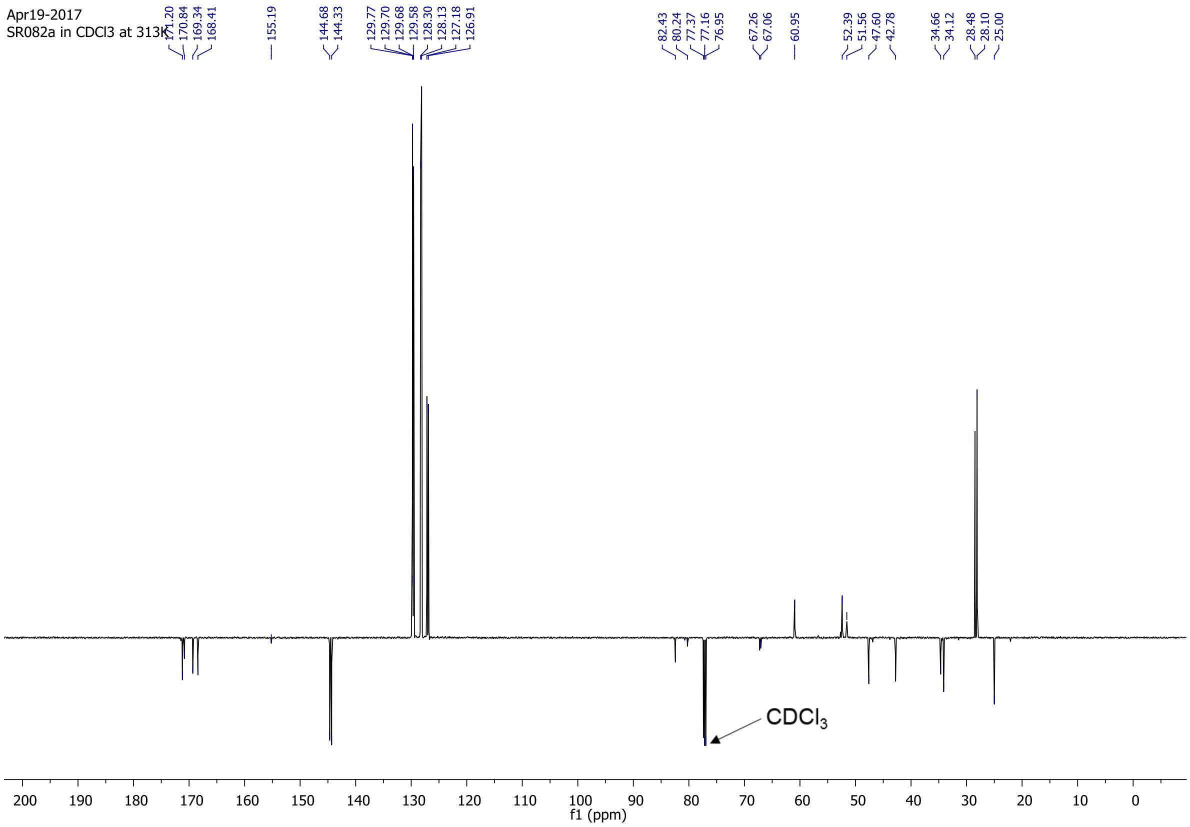

Cyclo(Boc-Cys-Pro-Gly-Cys-O*t*Bu) (136)

^{1}H NMR (400 MHz, DMSO-*d6*)

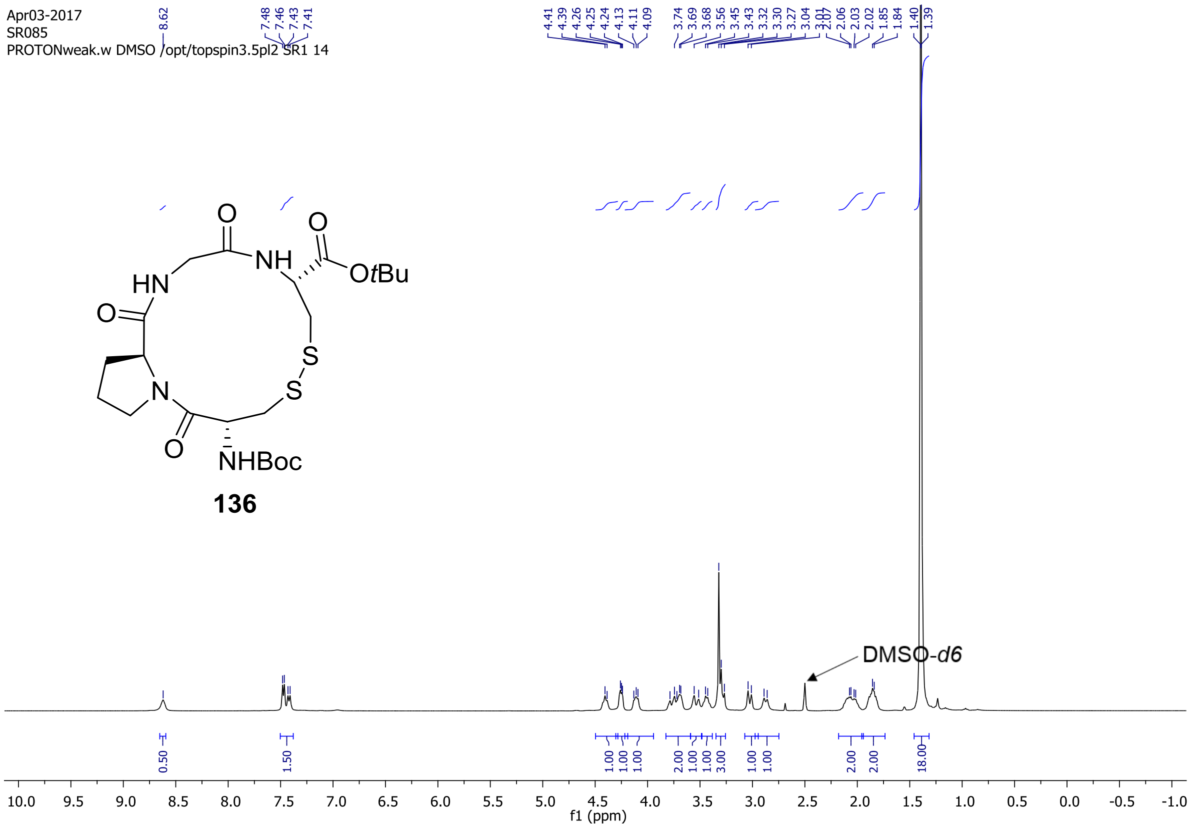

^{13}C NMR (101 MHz, DMSO-*d6*)

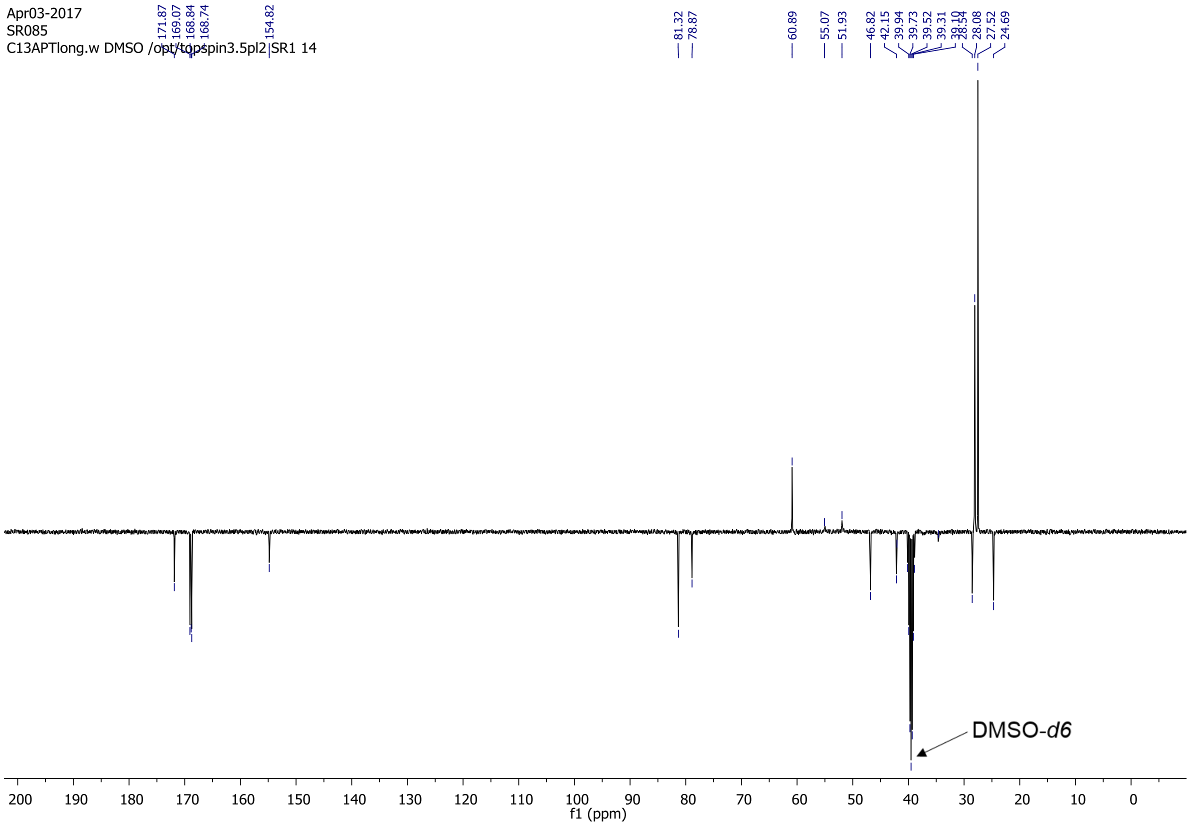

H-Leu-Ala-Gly-Ala-Tyr-OMe (137)

^{1}H NMR (700 MHz, DMSO-*d6*) with water-suppression

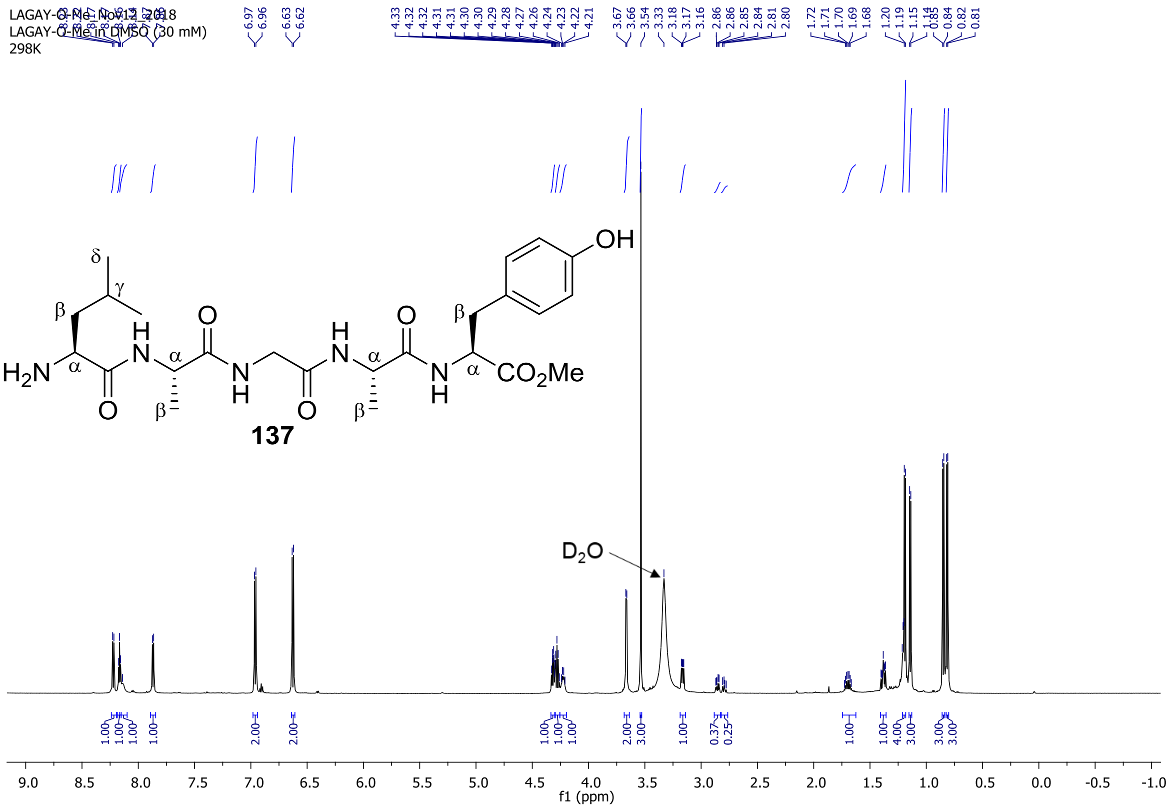

^{13}C NMR (126 MHz, DMSO-*d6*)

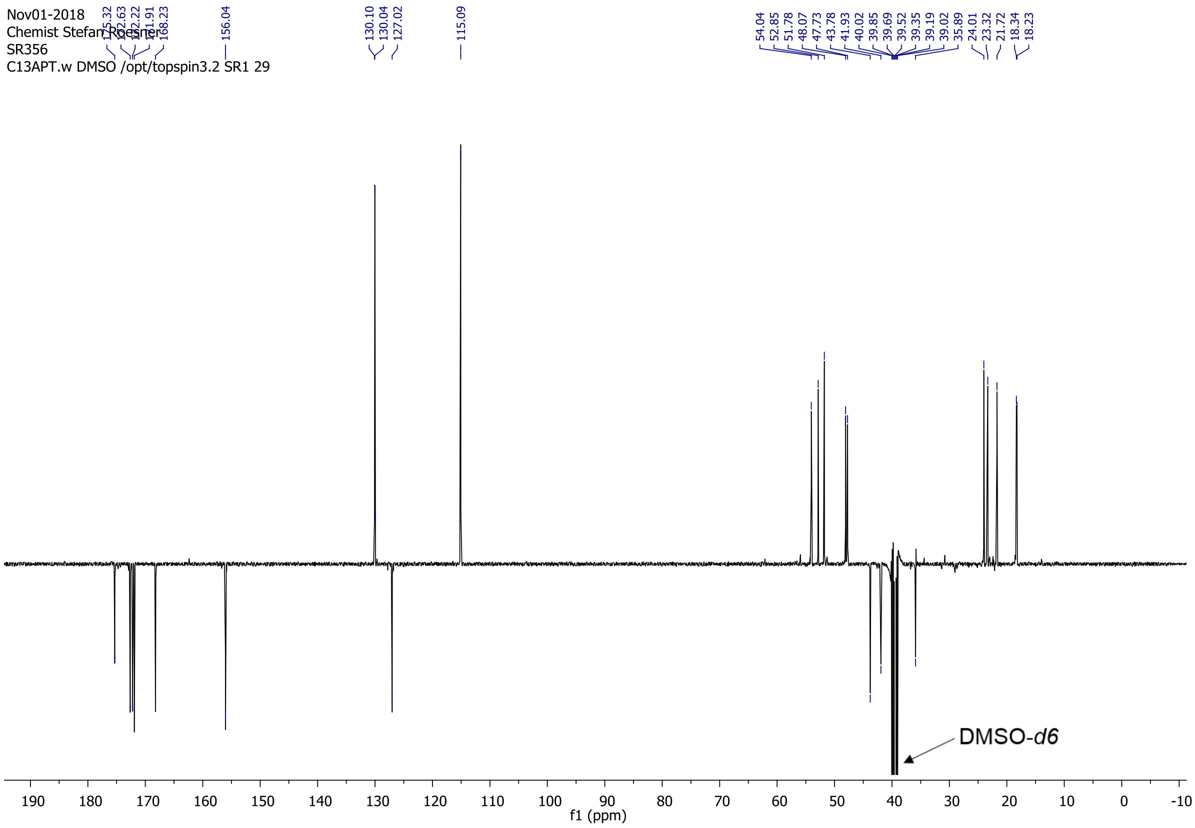

H-Leu-Ala-GOx-Ala-Tyr-OMe (138)

^{1}H NMR (700 MHz, DMSO-*d6*) with water-suppression

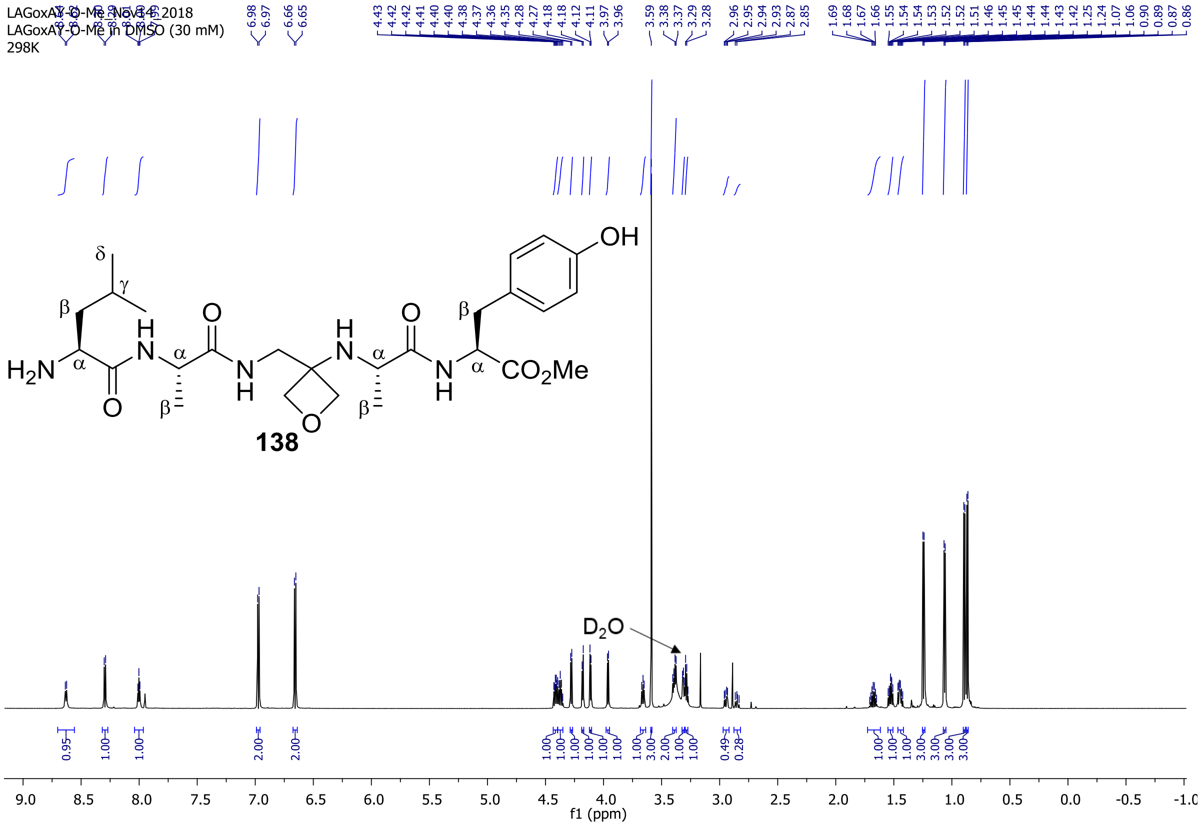

^{13}C NMR (126 MHz, DMSO-*d6*)

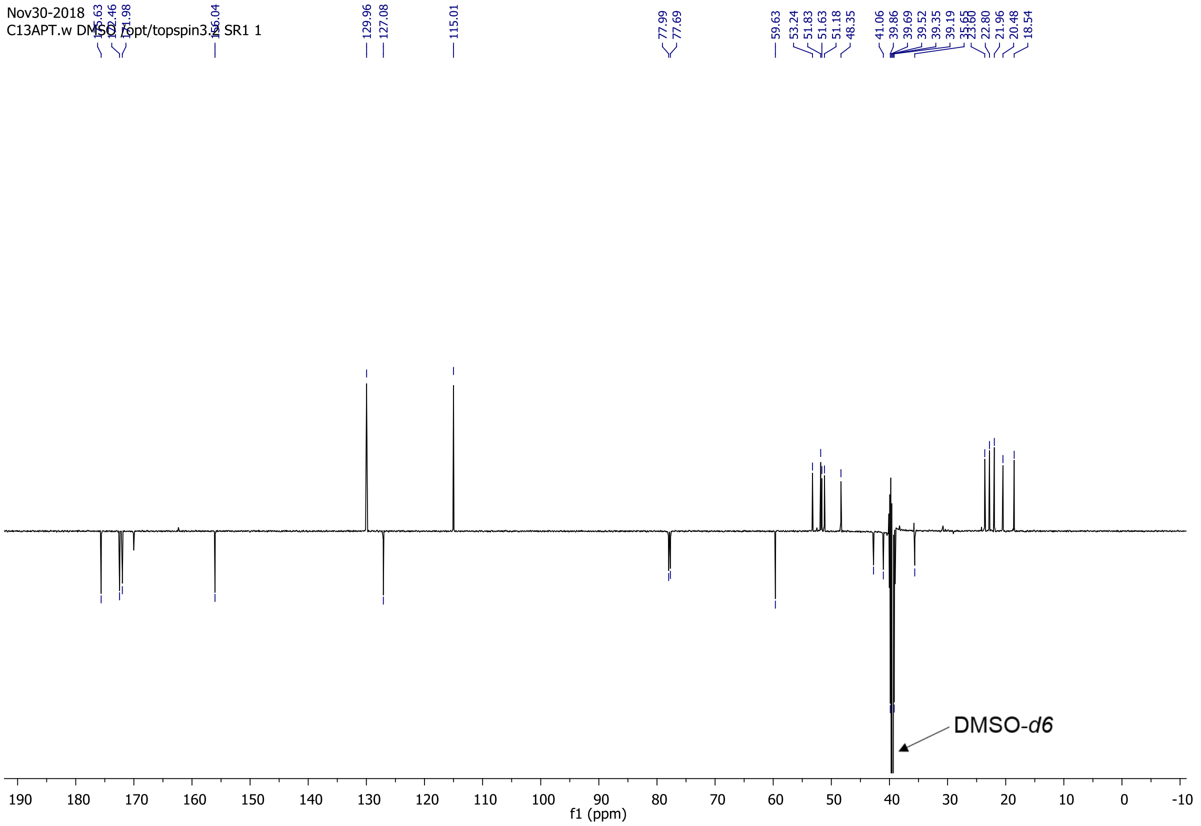

Supplement: Supplementary file 1 [file SC-010-C8SC05474F-s001.pdf]
